# A computational triage approach to the synthesis of novel difluorocyclopentenes and fluorinated cycloheptadienes using thermal rearrangements†
†Electronic supplementary information (ESI) available: Computational methodology, Cartesian coordinates for intermediates and transition states, synthetic procedures and characterisation spectra for novel compounds and further discussion on points highlighted in the text. See DOI: 10.1039/c6sc01289b



**DOI:** 10.1039/c6sc01289b

**Published:** 2016-06-16

**Authors:** David Orr, Jonathan M. Percy, Zoë A. Harrison

**Affiliations:** a WestCHEM Department of Pure and Applied Chemistry , University of Strathclyde , Thomas Graham Building, 295 Cathedral Street , Glasgow , G1 1XL , UK . Email: jonathan.percy@strath.ac.uk ; Fax: +44 (0)141 548 4898; b Refractory Respiratory Inflammation DPU , GlaxoSmithKline Medicines Research Centre , Gunnels Wood Road , Stevenage , SG1 2NY , UK

## Abstract

Electronic structure calculations have been used for the effective triage of substituent effects on difluorinated vinylcyclopropane precursors and their ability to undergo vinyl cyclopropane rearrangements (VCPR). Groups which effectively stabilised radicals, specifically heteroarenes, were found to result in the lowest energy barriers. Ten novel precursors were synthesised to test the accuracy of computational predictions; the most reactive species which contained heteroarenes underwent thermal rearrangements at room temperature to afford novel difluorocyclopentenes and fluorinated benzocycloheptadienes through competing VCPR and [3,3]-rearrangement pathways, respectively. More controlled rearrangement of ethyl 3-(1′(2′2′-difluoro-3′-benzo[*d*][1,3]dioxol-5-yl)cyclopropyl)propenoate (**22**) allowed these competing pathways to be monitored at the same time and activation energies for both reactions were determined; *E*_a(VCPR)_ = (23.4 ± 0.2) kcal mol^–1^ and *E*_a([3,3])_ = (24.9 ± 0.3) kcal mol^–1^. Comparing our calculated activation energies with these parameters showed that no single method stood out as the most accurate for predicting barrier heights; (U)M05-2X/6-31+G* methodology remained the best for VCPR but M06-2X/6-31G* was better for the [3,3]-rearrangement. The consistency observed with (U)B3LYP/6-31G* calculations meant that it came closest to a universal method for dealing with these systems. The developed computational design model correctly predicted the observed selectivity of rearrangement pathways for both our system and literature compounds.

## Abstract 2

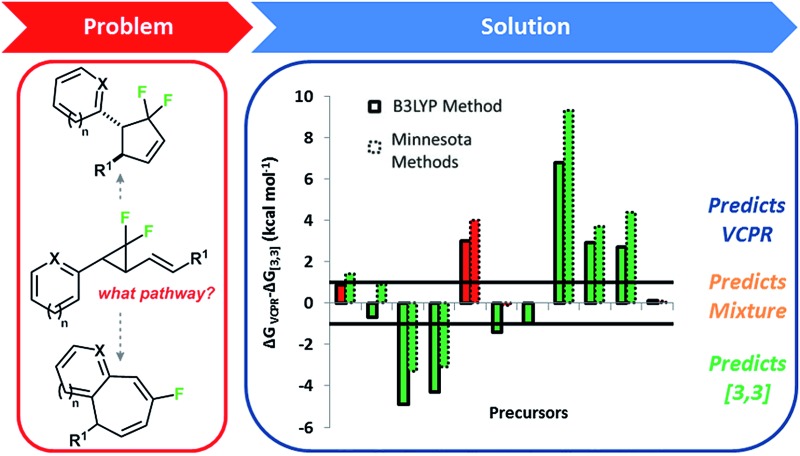
Electronic structure calculations have been used for the effective triage of substituent effects on difluorinated vinylcyclopropane precursors and their ability to undergo vinyl cyclopropane rearrangements (VCPR).

## Introduction

The continual rise in the processing power of modern computer systems has allowed the computational chemist to perform ever larger and more accurate calculations over shorter and shorter periods of time. Calculations are routinely performed for synthetically interesting reactions in order to characterise pathways in detail and rationalise experimental observations.^1^ Typically, these calculations are performed after considerable synthetic optimisation, which can be time consuming and expensive. Computational evaluation of reactions prior to the commitment of experimental resource is now becoming less rare having been shown to streamline investigations into a range of organic transformations.^2^

**Scheme 1:**
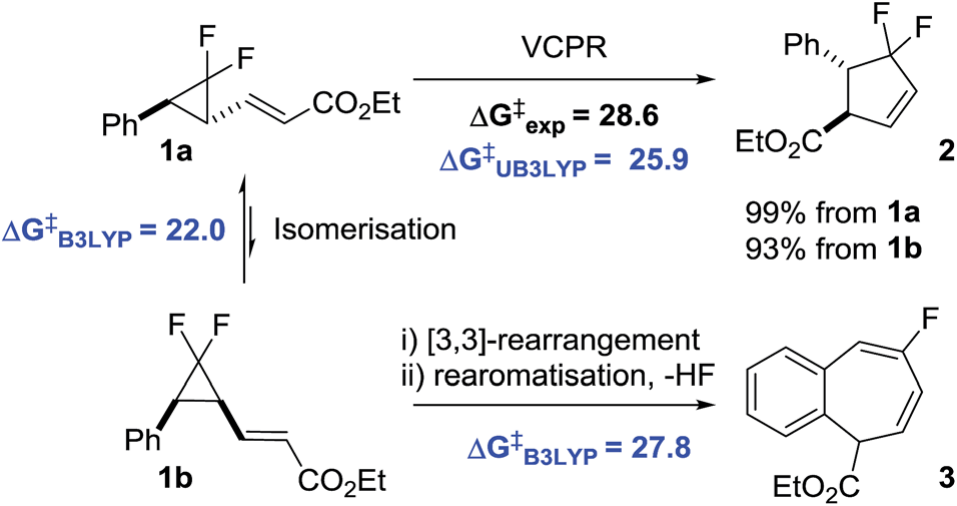
Correlation between experimental and calculated rates for ordering the rearrangement pathways of **1a** (Δ*G* ^‡^ values (blue) calculated using UB3LYP/6-31G*, gas phase, 298 K, Gaussian'09, all energy values are in kcal mol^–1^).

We found that electronic structure calculations were extremely helpful in developing our understanding of the rearrangement of **1a** to difluorocyclopentene **2** (Scheme 1).^3^

We observed good agreement between calculated (29.8 kcal mol^–1^, UM05-2X/6-31G*, with the conformationally simpler Me ester) and experimental (28.6 kcal mol^–1^) VCPR activation energies, but only the less expensive UB3LYP/6-31G* calculations predicted the order of reactivity between competing cyclopropane stereoisomerisation and [3,3]-sigmatropic rearrangement correctly. The system is most unusual in that reactions involving open shell singlets and triplet species are concurrent with a more classically concerted pericyclic rearrangement to **3**. Calculations also revealed the unexpected role that the alkene stereochemistry played in controlling rearrangement pathways; *E*-alkenoates underwent VCPR whereas *Z*-alkenoates reacted *via* initial [3,3]-rearrangement.

**Fig. 1:**
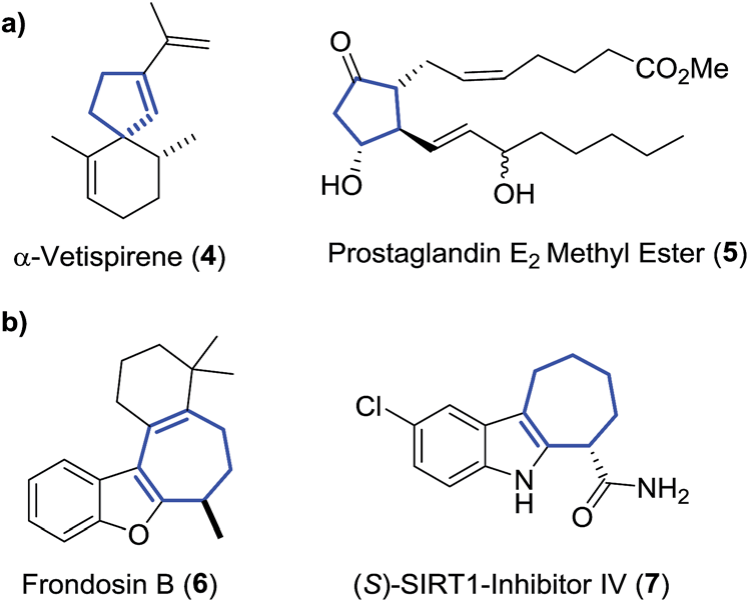
Natural products and active pharmaceutical compounds synthesised using (a) VCPR and (b) aromatic-vinylcyclopropane rearrangements (key ring structures formed during the rearrangements are highlighted in blue).

The two ring expansion rearrangements observed in our system have both been used successfully by other groups in total synthesis projects. VCPRs were utilised as key steps in securing the 5-membered cyclic cores for a range of complex structures,^4^ including α-vetispirene **4** from the sesquiterpene family of compounds,^5^ as well as the prostaglandin E_2_ methyl ester **5** (Fig. 1a).^6^ There have also been reports of related [3,3]-sigmatropic rearrangements,^7^ including divinylcyclopropane to cycloheptadiene rearrangements,^8^ being used in a similar fashion to generate molecular complexity. However, despite being utilised in the successful synthesis of marine sponge derived natural product frondosin B **6** ^9^ and the highly potent SIRT-inhibitor **7**,^10^ the [3,3]-rearrangements from our system are much less common (Fig. 1b).

**Scheme 2:**
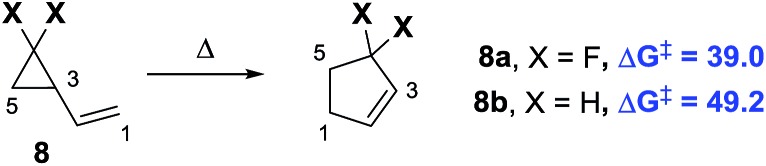
Effect of fluorine atom substitution on VCPR activation energies (Δ*G* ^‡^ experimental values, 298 K, re-calculated from activation parameters, kcal mol^–1^).^16^

The key rearrangements in the synthesis of **4** and **5** required high and very high temperatures of 190 °C and 560 °C, respectively. Substrate modifications^11^ and the utilisation of transition metal catalysts^12^ have started to allow access to room temperature rearrangements. Fluorine atom substitution on the cyclopropane ring has also been shown to have a beneficial effect.^13^ The ring strain energy is ∼10 kcal mol^–1^ higher for *gem*-difluorinated VCP **8a** than that for the parent hydrocarbon **8b** (Scheme 2).^14^ This induces regiospecific ring opening in **8a**, due to the weakened distal C_3_–C_5_ bond, and lowers the activation energy for VCPR (ΔΔ*G* ^‡^ = 10.2 kcal mol^–1^ between **8a** and **8b**).^13b,15^

Our VCPR of precursor **1a** exploited this effect, alongside stabilisation of the open shell singlet transition state through benzylic resonance, to lower the activation energy further (ΔΔ*G* ^‡^ = 20.6 kcal mol^–1^ from **1a** to **8b**). This system offers a highly atom-efficient route into a novel difluorocyclopentene at practical rearrangement temperatures (100 °C).^3^ Despite recent synthetic advances for securing difluorinated 5-membered ring structures,^17^ difluorinated cyclopentenes are still rare motifs in the literature.^18^ To our knowledge, there are currently no reported routes for the synthesis of structures similar to fluorinated benzocycloheptadiene **3**. Since both of these desirable fluorinated compounds were accessible from difluoro-VCP **1a**, our system represents an exciting building block class for accessing new fluorinated chemical space.^19^

**Fig. 2:**
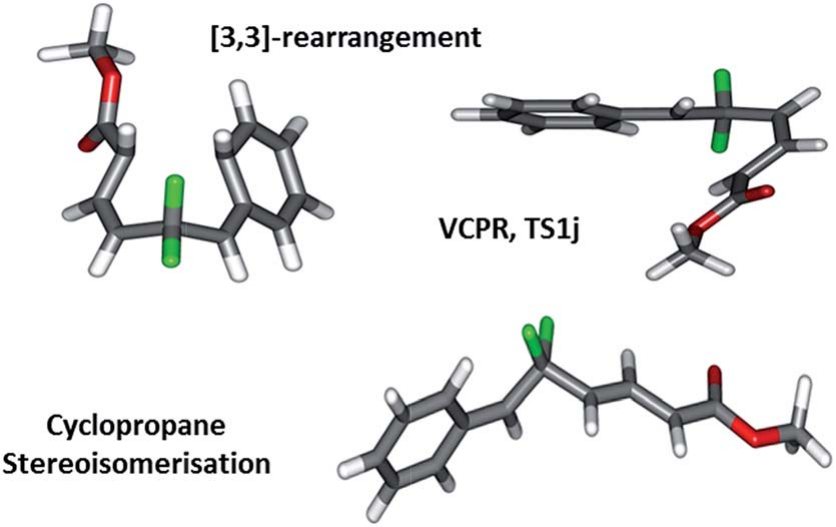
Geometries found representing the open shell singlet-VCPR, closed-shell [3,3]-sigmatropic rearrangement, and triplet cyclopropane stereoisomerisation transition states derived from VCP-**1a**/**1b**.

We now look to understand more fully what effect changing the functional groups around our difluorinated precursors has on the balance between these two rearrangement pathways; we wish to be able to design precursors which rearrange at relatively low temperatures. By securing accurate transition structures for all three possible rearrangement pathways (Fig. 2), we have ensured a relatively unique opportunity to start our study by assessing the scope and limitations of our system using electronic structure calculations before committing to any synthetic chemistry.

The computational screening would start by separately predicting the effect different substituents, attached either to the difluorocyclopropane or the alkene portions of the precursor, would have on the barrier for VCPR. A selection of interesting compounds based on the predicted ease of rearrangement would then be synthesised to assess the accuracy of the theoretical predictions. As the overall goal is to identify computational models that can be easily implemented by synthetic chemists, off-the-peg and less computationally-intensive methods would be preferred over calculations which required bespoke methods and were more demanding in terms of computational resource.^20^

## Results and discussion

### Effect of difluorocyclopropane substitution

**Fig. 3:**
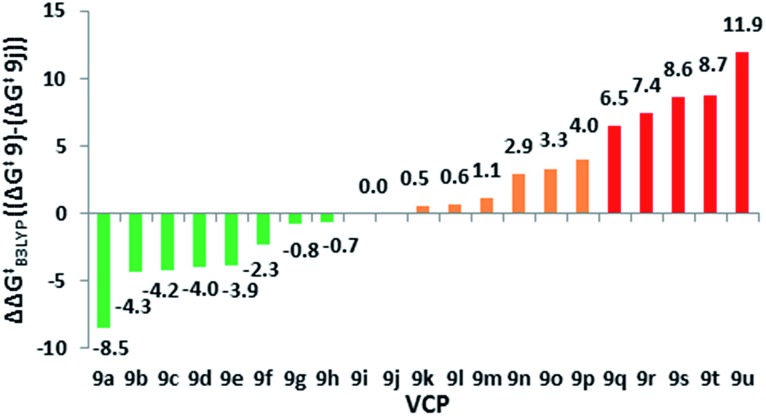
Difference in free energies of activation (Δ*G*‡B3LYP) between cyclopropane-substituted difluoro-VCP and reference **9j**. Green = lower Δ*G* ^‡^ (<25.3 kcal mol^–1^), orange = 0–5 kcal mol^–1^ higher Δ*G* ^‡^ and red = >5 kcal mol^–1^ increase in Δ*G* ^‡^.

We reported that the most cost effective method for calculating VCPR activation energies was an unrestricted B3LYP^21^ (UB3LYP) method with the 6-31G* basis set.^3^ This method was used to assess the impact of modification of the left-hand side of difluoro-VCP **9**, with calculated activation barriers ranked alongside phenyl-substituted precursor **9j** (Table 1 and Fig. 3).

**Table 1:** Effects of substitution on difluorocyclopropane precursors **9** (UB3LYP/6-31G*, gas phase, 298 K, Spartan'10)

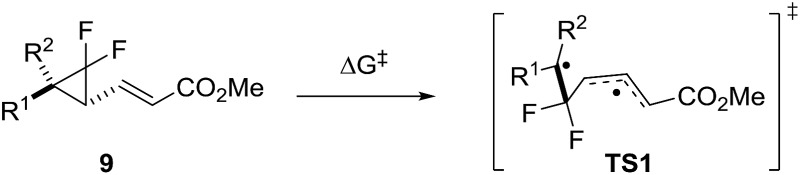				
VCP	R^1^	R^2^	Δ*G*‡B3LYP	ΔΔ*G*‡B3LYP^*a*^
**9a**	2-Pyrrolyl	H	16.8	–8.5
**9b**	2-Furyl	H	21.0	–4.3
**9c**	4-Pyridyl-*N*-oxide	H	21.1	–4.2
**9d**	2-Thiophenyl	H	21.3	–3.8
**9e**	2-*N*-Boc-pyrroyl	H	21.4	–3.9
**9f**	5-Benzo[*d*][1,3]dioxolyl	H	23.0	–2.3
**9g**	H_2_C=CH_2_ (vinyl)	H	24.5	–0.8
**9h**	3-Thiophenyl	H	24.6	–0.7
**9i**	2-Thiazolyl	H	25.3	0.0
***9j***	*Ph*	*H*	*25.3*	*0.0*
**9k**	Ph	Ph	25.8	+0.5
**9l**	HC≡C (alkynyl)	H	25.9	+0.6
**9m**	4-Pyridyl	H	26.4	+1.1
**9n**	2-Pyridyl	H	28.2	+2.9
**9o**	2,6-Dimethylphenyl	H	28.6	+3.3
**9p**	CN	H	29.3	+4.0
**9q**	Me	Me	31.8	+6.5
**9r**	–CH_2_(CH_2_)_3_CH_2_–		32.7	+7.4
**9s**	Me	H	33.9	+8.6
**9t**	C_6_H_11_	H	34.0	+8.7
**9u**	H	H	37.2	+11.9

^*a*^ΔΔ*G*‡B3LYP = (Δ*G*‡B3LYP**9x**) – (Δ*G*‡B3LYP**9j**).

We observed a dramatic rise in calculated activation energy when there was no additional substitution on the difluoro-VCP (**9u**, +11.9 kcal mol^–1^). Compounds with no aromatic functionality but one (**9s** and **9t**) or two (**9q** and **9r**) alkyl substituents were also found to have higher barriers for rearrangement (ranging from +6.5 to +8.6 kcal mol^–1^). We had observed previously that temperatures of 180 °C facilitated a [3,3]-rearrangement pathway with a calculated activation energy of 32.9 kcal mol^–1^ (Δ*G*‡B3LYP, UB3LYP/6-31G*, gas phase, 298 K, Gaussian'09),^3^ suggesting that low temperature rearrangement with alkyl substituents would be unlikely. Higher reaction temperatures also have the potential to activate a competitive [1,5]-hydride shift pathway^22^ so these substitution patterns were ruled out of our synthetic study.

**Fig. 4:**
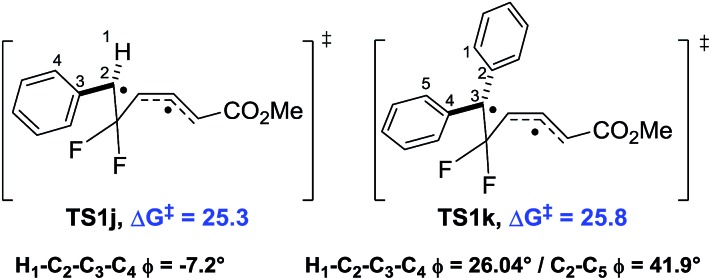
Aromatic ring planarity effects on benzyl radical stabilisation in **TS1j** and **TS1k** (dihedral angles represented by *φ*, Δ*G* ^‡^ values (blue) calculated using UB3LYP/6-31G*, gas phase, 298 K, Spartan'10, all energy values are in kcal mol^–1^).

We observed a higher activation energy for 2,6-dimethylphenyl **9o** (+3.3 kcal mol^–1^) due to steric interactions between the methyl protons and the proton (2.26 Å) and fluorine atoms (2.23 and 2.25 Å) attached to the cyclopropane ring in the transition state. These small steric interactions are tolerated in the transition state to ensure the benzylic radical remains coplanar with the aromatic ring. Unlike the bis-alkyl substituents, no barrier-lowering was observed for bis-phenyl **9k** (+0.5 kcal mol^–1^). Steric repulsion between the *ortho*-protons in **TS1k** induces a slight rotation of each ring, forcing stabilising aryl groups out of the plane of the benzylic radical (Fig. 4). Sustmann and co-workers investigated this twist angle effect in more detail,^23^ but no further investigations into bis-arylated difluoro-VCP systems were undertaken because the Δ*G*‡B3LYP values were so similar to those for **9j**.

A wider range of effects was observed when R^1^ was an heteroaryl group; 2- and 4-pyridyl groups raised the predicted barrier. 2-Furyl, 2-pyrrolyl and 2-thiophenyl groups lowered it significantly, consistent with their known effects of free radical stabilisation as quantified by Creary.^24^ A piperonyl group also activated the system (see the ESI† for a wider discussion of the systems screened and the substituent effects).

### Effect of alkene substitution

**Fig. 5:**
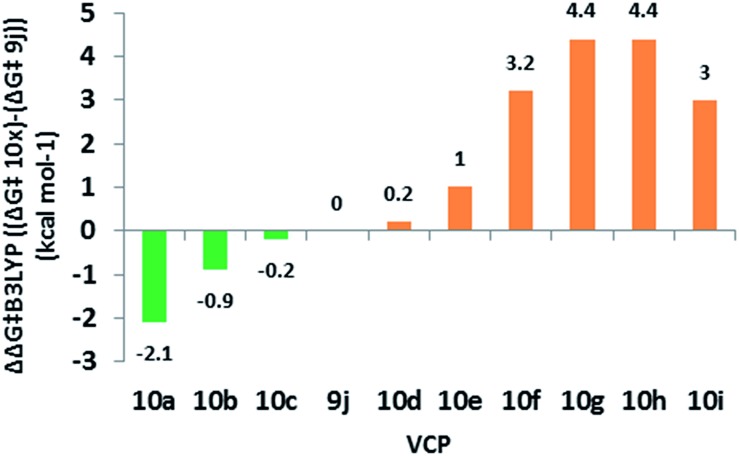
Difference in free energy (Δ*G*‡B3LYP) between alkene substituted difluorovinylcyclopropanes and reference **9j**. Green = lower Δ*G* ^‡^ (<25.3 kcal mol^–1^) and orange = 0–5 kcal mol^–1^ greater Δ*G* ^‡^.

We used **10** as a template for investigating modifications to the alkene fragment of the precursors, focusing on the effects of alkene configuration (R^1^ *versus* R^2^) and radical stabilising substituents (Table 2 and Fig. 5).

**Table 2:** Effects of alkene substitution on VCP **10** (UB3LYP/6-31G*, gas phase, 298 K, Spartan'10)

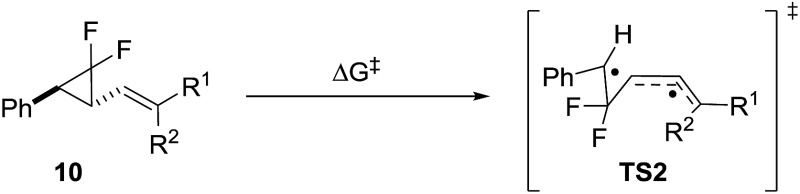					
Entry	VCP	R^1^	R^2^	Δ*G*‡B3LYP	ΔΔ*G*‡B3LYP^*a*^
1	**10a**	CN	H	23.2	–2.1
2	**10b**	CON(Me)OMe	H	24.4	–0.9
3	**10c**	CH_2_OH	H	25.1	–0.2
*4*	***9j***	*CO*_*2*_ *Me*	*H*	*25.3*	*0.0*
5	**10d**	Me	H	25.5	0.2
6	**10e**	H	H	26.3	1.0
7	**10f**	H	CH_2_OH	28.5	3.2
8	**10g**	H	Me	29.7	4.4
9	**10h**	H	CN	29.7	4.4
10	**10i**	CO_2_Me	Me	28.3	3.0

^*a*^ΔΔ*G*‡B3LYP = (Δ*G*‡B3LYP**10x**) – (Δ*G*‡B3LYP**9j**).

The dramatic reactivity difference between the *E*- and *Z*-alkene isomers of **9j** previously reported^3^ was maintained when a wider range of substituents was examined. Calculated activation energies for *E*-substituted precursors (Table 2, entries 1–5) were lower than for the unsubstituted precursor **10e** (Table 2, entry 6), whilst the barriers for the corresponding *Z*-diastereoisomers were higher (Table 2, entries 7–9). The narrow range of free energies of activation observed for the *E*-diastereoisomers (23.2 to 25.5 kcal mol^–1^) suggests that all of these precursors should rearrange at temperatures close to or below 100 °C. The narrow spread of barrier heights (ΔΔ*G*‡B3LYP = 6.5 kcal mol^–1^) as the alkene substituents vary is half that observed when the similar changes were made on the cyclopropane (see Table 1). This suggests that a wider range of substituents could be tolerated on the alkene fragment since the radical is already stabilised through allylic resonance.

The transition states for the *Z*-diastereoisomers of Weinreb amide **10b** and methyl ester **10i** failed to optimise, but the alcohol (**10f**, +3.2 kcal mol^–1^), methyl (**10g**, +4.4 kcal mol^–1^) and cyanide (**10h**, +4.4 kcal mol^–1^) species all optimised with higher activation energies than the corresponding *E*-series. Disubstituted alkene **10i** (Table 2, entry 10) had a higher free energy of activation than ester **9j** (+3.0 kcal mol^–1^) but the introduction of the ester functionality lowered the activation energy for the rearrangement of *Z*-methyl **10g** (difference of 1.4 kcal mol^–1^ between **10i** and **10g**).

### Synthetic investigations

After the systematic examination of the functional group tolerance of the VCPR using electronic structure calculations, the synthesis of a selection of compounds was undertaken in order to test the computational predictions. While the literature describing the synthesis of difluorocyclopropanes by difluorocarbene transfer is extensive,^17h,25^ reaction substrates often seem to have been selected for electron-richness and low levels of functionality. We have challenged the published methods extensively in securing this set of compounds; our studies are described fully in the ESI.† The approach described below is a pragmatic one, arrived at after extensive optimisation for this focussed set of compounds.

### Synthesis of substituted difluoro-VCP

**Scheme 3:**
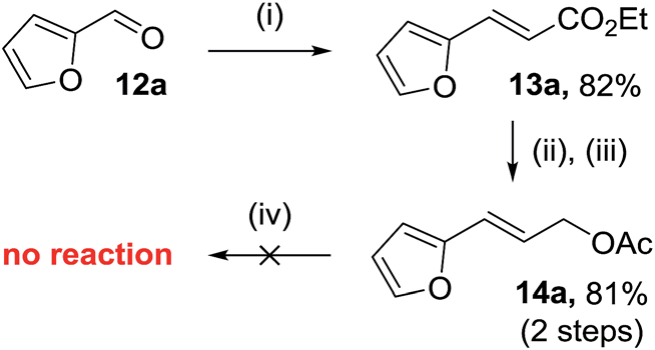
Synthesis and failed difluorocyclopropanation of 2-furyl allyl acetate **14a**. Conditions: (i) (carbethoxymethylene)-triphenylphosphorane (1.1 eq.), DCM, r.t., 17 h (ii) DIBAL (3 eq.), toluene, –78 °C to r.t., 8 h (iii) Ac_2_O (1.2 eq.), pyridine (1.2 eq.), DCM, 45 °C, 5 h (iv) MDFA (2.5 eq.), TMSCl (2.5 eq.), KI (2.8 eq.), diglyme (1.17 eq.), 120 °C, 24 h.

The efficient synthesis of phenyl-VCP **1a** (73% over three steps) previously reported, relied on the successful difluorocyclopropanation of commercially available cinnamyl acetate with methyl 2,2-difluoro-2-(fluorosulfonyl)acetate (MDFA, **11**). It was believed that the more electron-rich heteroarene substituents would help the cyclopropanation reaction by raising the nucleophilicity of the alkene. However, only decomposition was observed when **13a** was exposed to the MDFA conditions (Scheme 3).

**Scheme 4:**
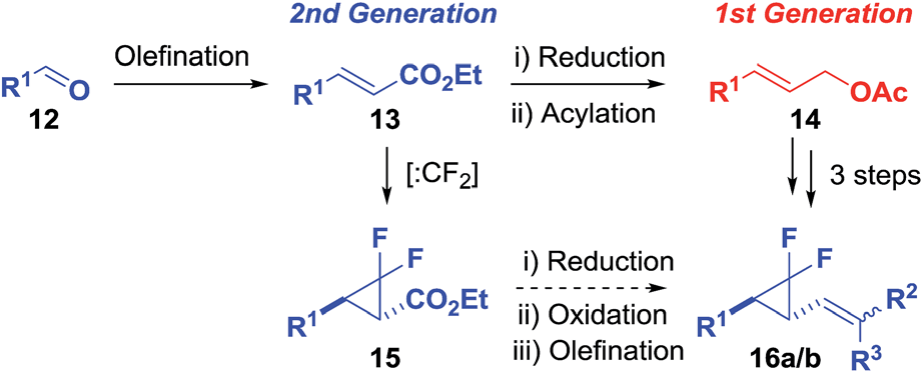
1^st^ and 2^nd^ generation synthetic routes for accessing difluoro-VCP **16**.

A 2^nd^ generation synthesis of precursor **16** from alkenoate **13** was proposed (Scheme 4); alkenoates **13** are easily accessible on a gram scale from the corresponding commercial or easy-to-prepare aldehydes and are more stable olefins for the high temperature reactions involving electron-rich aromatic substituents than allyl acetate **14**.

Wittig reactions of aldehydes **12a–h** (see the ESI†) as described previously afforded the desired alkenoates **13a–h** in good to excellent yields (45–98%, Table 3, entries 1–10).

**Table 3:** Synthesis of difluorocyclopropyl allyl alcohols **17a–h** from aldehydes **12a–h** ^*a*^

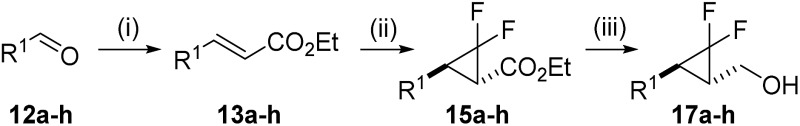						
Entry	R^1^	x	**13**	**15**		**17**
			Yield^*b*^ (%)	Conv.^*c*^ (%)	Yield^*b*^ (%)	Yield^*b*^ (%)
1	2-Furyl	**a**	82	66	40	94
2	Ph	**b**	—	28^*d*^	—	—
3			—	29^*d*^ ^,^ ^*e*^	n.d.^*f*^	—
4			73	37	n.d.^*f*^	—
5	2-Thiophenyl	**c**	98	77	71	50
6	5-Benzo[*d*][1,3]-dioxole	**d**	94	50	43	75
7	2-Pyridyl	**e**	87	0	—	—
8	2-*N*-Boc-pyrrolyl	**f**	60	92	54	6
9	2-Thiazolyl	**g** ^*g*^	74^*h*^	0	—	—
10	3-Me-2-furyl	**h** ^*i*^	45^*j*^	80	45	84

^*a*^Conditions: (i) (carbethoxymethylene)triphenylphosphorane (1.1–1.3 eq.), DCM, r.t., 6–20 h (ii) MDFA (2.5 eq.), TMSCl (2.5 eq.), KI (2.8 eq.), diglyme (1.17 eq.), 120 °C, 4 h (iii) DIBAL (3 eq.), toluene or DCM, –78 °C to r.t., 8 h.

^*b*^Isolated yields.

^*c*^Determined by ^1^H NMR.

^*d*^Reaction time of 24 h.

^*e*^Starting from commercial ethyl cinnamate **13b**.

^*f*^ **13b** and **15b** were inseparable *via* column chromatography or distillation.

^*g*^Aldehyde **12g** was synthesised from thiazole and used crude in the olefination reaction (see ESI for details).

^*h*^Calculated over two steps from thiazole, 4 : 1 mixture of *E* : *Z*-isomers.

^*i*^Aldehyde synthesised *in situ* from the oxidation of 2-hydroxymethyl-3-methyl furan (see ESI for details).

^*j*^Calculated over two steps from methyl 3-methyl-2-furoate.

Furyl alkenoate **13a** proved to be more stable under MDFA-mediated difluorocyclopropanation conditions than acetate **14a** but only when a shorter reaction time of 4 hours was used (Table 3, entry 1); prolonged reaction times of 24 h resulted in a decreased conversion to ester **15a** (66% compared with 50%, respectively). The conversion to **15a** could be increased to 87% by using sodium chlorodifluoroacetate (Na-CDFA, 10 eq.) conditions,^25e,26^ but a drop in isolated yield was observed (22%) and attributed to product decomposition at the higher reaction temperature of 180 °C over a longer reaction time of 25 hours. Reactions with ethyl cinnamate **13b** (Table 3, entries 2–4) showed that this 2^nd^ generation route is less efficient than our previously published entry from cinnamyl acetate, due to the alkene reactivity rather than the conditions used to access alkenoates.

A wider range of heteroarene functionalised difluorocyclopropyl esters could be isolated in moderate to good yields (40–71%) using our shorter difluorocyclopropanation conditions from the corresponding alkenoates (Table 3). 2-Pyridyl **13e** and 2-thiazoyl **13g** failed to show any signs of reaction. Successful difluorocyclopropanation of a more reactive vinyl pyridine was reported in the patent literature using higher temperature decomposition of sodium chlorodifluoroacetate (Na-CDFA);^27^ these conditions also failed to cause alkenoate **13e** to react. The lack of reactivity was attributed to side reactions between the substituents and difluorocarbene; a full discussion can be found the ESI.†

DIBAL-mediated reduction of the isolated difluorocyclopropyl esters to the corresponding alcohols gave mixed results with moderate to excellent yields (50–94%) observed for furyl **17a**, thiophenyl **17c**, piperonyl **17d** and 3′-methyl-furyl **17g** analogues (Table 3). Unfortunately, *N*-Boc pyrrolyl **15f** was unstable under reduction conditions and gave a poor 6% yield of **17f**.

**Scheme 5:**
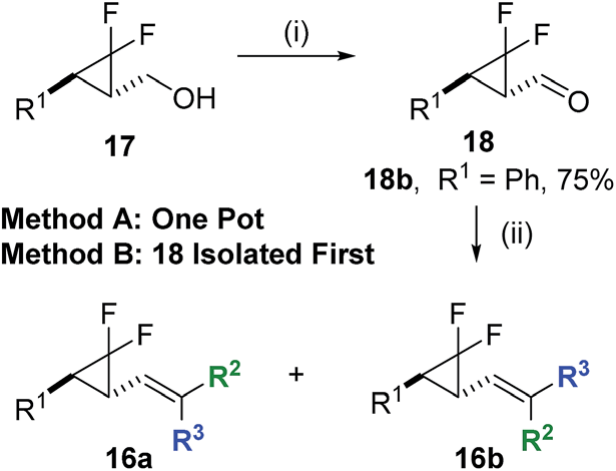
Oxidation and olefination chemistry used to access a range of alkene functionality. Conditions: (i) BAIB (1.1 eq.), TEMPO (0.1 eq.), DCM, r.t., 5–6 h, (ii) Ph_3_P=C(R^1^)R^2^, DCM, r.t., 15–16 h.

Vatele's room temperature tandem oxidation/olefination conditions were utilised as the final step in the precursor synthesis, minimising rearrangements during alkenoate formation.^28^ The one-pot method previously found to be successful with ethyl ester functionalisation only worked with a selection of commercially available phosphoranes (Scheme 5, Method A). However, aldehyde **18b** could be isolated in good yield using the same room temperature oxidation chemistry, allowing direct Wittig reactions to proceed smoothly (Scheme 5, Method B).

Three more alkenes were prepared in good yields from phenyl **17b**; these were *E*-Weinreb amide **19a**, α-methyl ester **20a** and cyanide **21** (Table 4, entry 1–3, respectively). Furthermore, heteroarene-based building blocks all underwent functionalisation successfully but only piperonyl precursor **22a** could be isolated (50%, Table 4, entry 4); the 2-furyl, 2-thiophenyl and 3-methyl-2-furyl congeners all rearranged at ambient temperature before isolation (Table 4, entry 5–10) could be completed.

**Table 4:** Complete synthesis of difluoro-VCP precursors **19–27** ^*a*^

Entry (^*b*^)	R^1^	R^2^	R^3^	VCP	Yield^*c*^ (%)	
					*E*-Alkene (**a**)	*Z*-Alkene (**b**)
1 (A)	Ph	CON(OMe)Me	H	**19**	74	9
2 (A)	Ph	CO_2_Et	Me	**20**	74	0
3 (B)	Ph	H/CN	H/CN	**21**	84^*d*^	
4 (A)	2-Piperonyl	CO_2_Et	H	**22**	50	n.d.^*e*^
5 (A)	2-Furyl	CO_2_Et	H	**23**	Full conversion^*f*^	
6 (A)	2-Thiophenyl	CO_2_Et	H	**24**	Full conversion^*f*^	
7 (A)	2-Furyl	CO_2_Et	Me	**25**	Full conversion^*f*^	
8 (A)	2-Thiophenyl	CO_2_Et	Me	**26**	Full conversion^*f*^	
9 (A)	3-Me-2-furyl	CO_2_Et	H	**27**	Full conversion^*f*^	

^*a*^Compounds represented by numbers and suffix **a** and **b** correspond to *E*- and *Z*-isomers, respectively.

^*b*^Synthetic methodology based on Scheme 5.

^*c*^Isolated yields unless otherwise stated.

^*d*^ **21a** and **21b** could not be separated by column chromatography and instead were isolated as a 3 : 2 mixture, respectively (mixture determined by ^1^H NMR).

^*e*^ **22b** formed during the reaction but could not be separated from a mixture with **22a** (21% isolated yield of **22a**/**22b** mixture).

^*f*^All precursors were successfully formed but reactions resulted in complex mixtures due to competing low temperature rearrangements.

Although 2-furyl **9b** and 2-thiophenyl **9d** had some of the lowest predicted VCPR activation energies (21.0 and 21.3 kcal mol^–1^, respectively), the observed low temperature rearrangements were still surprising; all reactions were conducted at room temperature and only warmed briefly during the evaporation of solvent (maximum temperature 40 °C). A repeated synthesis of VCP-**23** (furan) evaporated the reaction solvent under a stream of nitrogen at ambient temperature and resulted in a similar mixture of products. These results suggest that the rearrangements occurred at room temperature (<20 °C) and not during work up. To our knowledge these are the first examples of VCP precursors undergoing low temperature thermolysis without additional additives; previously only transition metal mediated^12a,29^ or charge accelerated rearrangements^11,30^ occurred at temperatures close to ambient.

Deconvolution of crude oxidation/Wittig reaction mixtures for furyl and thiophenyl substrates was difficult due to the presence of triphenylphosphine oxide side product. All of these reaction mixtures were purified twice, first to remove reaction impurities in order to try to reveal the reaction outcome (see ESI† for crude reaction spectra), then a final separation to isolate products free from phosphine oxide (Table 5).

**Table 5:** Reaction outcomes from furyl and thiophenyl precursors **23–26**

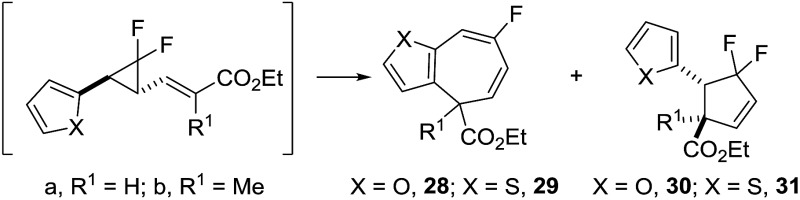							
Entry	VCP	X	R^1^	Crude observations^*a*^ (% conversion)	Product^*b*^ (%)		
					**28**/**29**		**30**/**31**
1	**23**	O	H	**28a** (>95), **30a** (trace)	n.a.^*c*^		
2	**24**	S	H	**29a** major, **31a** minor, evidence of **24**	**29a** (12)	**31a** (3)^*d*^	
3	**25**	O	Me	**28b** (100)	**28b** (48)	**30b** (0)	
4	**26**	S	Me	**29b** (100)	**29b** (55)	**31b** (0)	

^*a*^Percentage conversion determined by ^1^H NMR after 1^st^ purification.

^*b*^Isolated yield.

^*c*^Compound decomposed during purification attempts (see ESI for further information).

^*d*^50% purity (determined by ^1^H NMR) containing **29a**.

Precursor **23** (furyl) was absent after the reaction; instead mixed fractions from the first purification confirmed that mono-fluorinated cycloheptadiene **28a** was the favoured rearrangement product over difluorocyclopentene **30a** (Table 5, entry 1). Attempts at isolating rearrangement product failed due to product decomposition but distinctive ^19^F NMR signals which were consistent with isolated thiophene product were used for assigning rearrangement outcomes.

The synthesis of thiophenyl **24a**/**24b** resulted in more complex fraction mixtures from the first purification, with NMR evidence for VCP **24a** and **24b**, as well as rearrangement products **29a** and **31a** (Table 5, entry 2). Further thermolysis reactions of mixtures containing VCP precursors showed that *E*-isomer **24a** rearranged at 40 °C whereas the corresponding *Z*-isomer **24b** required the higher temperature of 50 °C (see ESI† for full discussion). The major mono-fluorinated cycloheptadiene **29a** could be isolated in 12% yield and minor difluorocyclopentene **31a** in a lower 3% yield (but of only 50% purity because of the presence of cycloheptadiene **29a**).

Previous computational investigations predicted that an α-methyl substituent on the alkene for VCP precursor **10i** would increase the activation barrier for VCPR rearrangement (*vide supra*). It was proposed that similar substitution would enable some temperature control of the rearrangement of highly reactive heterocyclic precursors. However, despite an increase in calculated activation energies for furyl **25a** (Δ*G*‡B3LYP = 23.5 kcal mol^–1^, +2.5 *cf.* **9b**) and thiophenyl **26a** (Δ*G*‡B3LYP = 23.7 kcal mol^–1^, +2.4 *cf.* **9d**), experimental results still gave room temperature rearrangements (Table 5, entries 3–4). Interestingly, both precursors reacted exclusively *via* the [3,3]-pathway, leading to moderate yields of both **28b** (48%) and **29b** (55%).

**Scheme 6:**
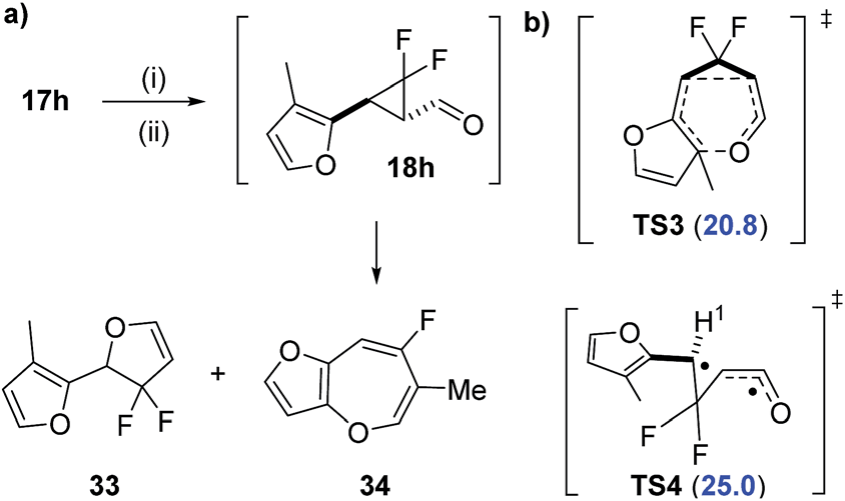
(a) Oxidation/olefination of alcohol **17h** resulting in unexpected rearrangement of aldehyde **18h**. (b) Electronic structure calculations for proposed rearrangement (UB3LYP/6-31G*, Spartan'10, gas phase, kcal mol^–1^). Conditions: (i) BAIB (1.1 eq.), TEMPO (10 mol%), DCM (3 mL), (ii) Ph_3_P=CHCO_2_Et (1.3 eq.) (one pot).

We also attempted the synthesis of 3-methyl furyl precursor **27** expecting that the methyl group would cause unfavourable steric interactions in the [3,3]-transition state and instead favour VCPR (Scheme 6a). However, like all other heteroarene substituted precursors, rearrangement was observed before isolation, but ^19^F NMR reaction monitoring of the tandem oxidation/olefination of alcohol **17h** suggested that rearrangement had occurred from aldehyde **18h** (see ESI† for further discussion).

The two major products were tentatively assigned as dihydrofuran **33** and benzooxepine **34** due to the strong similarities between ^19^F NMR chemical shifts reported by Hammond^31^ and ourselves,^3^ respectively. Electronic structure calculations for the [3,3]-rearrangement *via* **TS3** supported room temperature rearrangement with a low Δ*G*‡UB3LYP value of 20.8 kcal mol^–1^ (Scheme 6b). Analysis of the VCPR of aldehyde **18h** through **TS4** gave a higher calculated barrier for rearrangement (Δ*G*‡UB3LYP = 25.0 kcal mol^–1^), but a low spin operator value (*S* ^2^ = 0.291) suggested that the rearrangement is more likely to be concerted or through a donor–acceptor ring opening/ring closing mechanism.^32^ Purification of the resulting crude reaction mixture failed, due to either decomposition or volatility of the proposed products. Further synthetic and computational investigations into these appealing fluorinated products are underway but they do not contribute to the development of the computational model in this study.

The observed experimental results with heterocyclic precursors link well to the calculated low activation energies for VCPR. However the lack of control, and in some cases dominance of the [3,3]-pathway, was surprising because temporary dearomatisation is required. Experimental activation energies were required from more controlled rearrangements in order to screen for the best computational methods for assessing this competing pathway.

### Thermal rearrangement of isolated difluoro-VCP

**Scheme 7:**
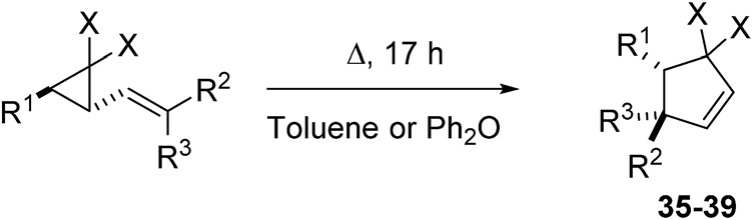
Rearrangement conditions for isolated-VCP.

The rearrangement temperatures for precursors which could be isolated were optimised to give full consumption of VCP after 17 hours (±5 °C) (Scheme 7). Normalising the reaction temperature against a fixed reaction time allows for the role that different substituents play on rearrangement rates (Table 6) to be appreciated more readily.

**Table 6:** Thermal rearrangement of isolated VCP

Entry	VCP precursor					Temp. (°C)	VCPR product		
	#	X	R^1^	R^2^	R^3^		#	Conv.^*a*^ (%)	Yield^*b*^ (%)
1	**19a**	F	Ph	CON(Me)OMe	H	95	**35**	100	97
2	**19b**	F	Ph	H	CON(Me)OMe	160		0^*c*^	—^*d*^
3	**20a**	F	Ph	CO_2_Et	Me	155	**36**	0^*c*^	—^*e*^
4	**21a**/**b** ^*f*^	F	Ph	H/CN	H/CN	90	**37**	71^*g*^	—
5^*h*^	**21b**	F	Ph	H	CN	160		100	48^*i*^
6	**47**	H	Ph	CO_2_Et	H	220	**38**	100	40^*j*^
7	**19a**	F	Piperonyl	CO_2_Et	H	70	**39**	42^*k*^	18^*l*^

^*a*^Conversion to product (determined by ^1^H or ^19^F NMR).

^*b*^Isolated yields unless otherwise stated.

^*c*^Full conversion of VCP precursor was observed.

^*d*^Decomposition was observed.

^*e*^Clean product could not be isolated from crude reaction mixture.

^*f*^3 : 2 mixture of **21a** and **21b**, respectively.

^*g*^Crude mixture also contains 26% **21b** and 3% *cis*-**42** (determined by ^19^F NMR).

^*h*^Using crude reaction mixture from entry 4.

^*i*^6 : 1 ratio of difluorocyclopentene **37** and alkene isomer **43** (by ^1^H NMR).

^*j*^22% of *cis*-cyclopentene **38b** was also isolated.

^*k*^Crude reaction mixture also contains 58% of [3,3]-product (**44**, by ^19^F NMR).

^*l*^11% of **44** was also isolated.

**Fig. 6:**
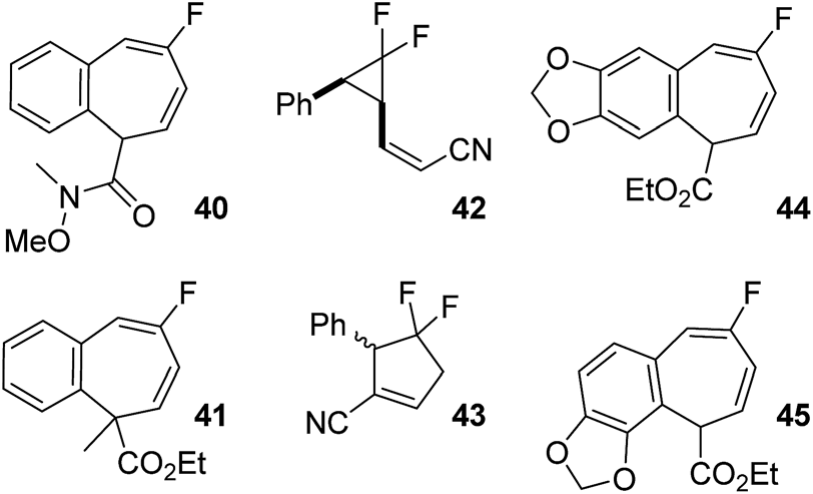
Potential side products from the rearrangement of difluorinated VCP discussed in Table 6.

Weinreb amide **19a** rearranged at 95 °C, 5 °C lower than the corresponding ethyl ester **1a**, to afford a near quantitative yield of difluorocyclopentene **35** (Table 6, entry 1), consistent with the slightly lower calculated activation energy (Δ*G* ^‡^ = –0.9 kcal mol^–1^). Minor stereoisomer **19b** required higher temperatures to induce rearrangement, favouring what seemed to be a [3,3]-pathway over VCPR, consistent with previously investigated *Z*-alkenoates. Diene **40** was proposed as one of the major products (Fig. 6) but these harsher conditions resulted in some decomposition and poor recovery of observed products (Table 6, entry 2). Despite the slightly lower temperature of 155 °C required for methylated alkenoate **20a** (Table 6, entry 3), decomposition was again observed and only firm ^19^F NMR identification of diene **41** could be achieved (see ESI† for further discussion). The higher temperature required for rearrangement of **20a** compared with ester **1a** was entirely consistent with an increased calculated barrier height (+3.0 kcal mol^–1^, *vide supra*).

It was predicted that the *E*-isomer (**21a**) would rearrange more rapidly in the isolated mixture of nitriles-**21a** and **21b** since the calculated activation energy for **21a** was 6.5 kcal mol^–1^ lower than that of the *Z*-isomer (**21b**). Thermolysis of the mixture at 90 °C over 17 hours gave full conversion of **21a** to difluorocyclopentene **37**; **21b** only showed cyclopropane stereoisomerisation to *cis*-**42** at this temperature (Table 6, entry 4). Only when the resulting mixture was re-heated to 160 °C was full conversion of the *Z*-isomers observed (Table 6, entry 5), affording difluorocyclopentene **37** in a 48% yield in a 6 : 1 ratio with alkene regioisomer **43**.

**Scheme 8:**
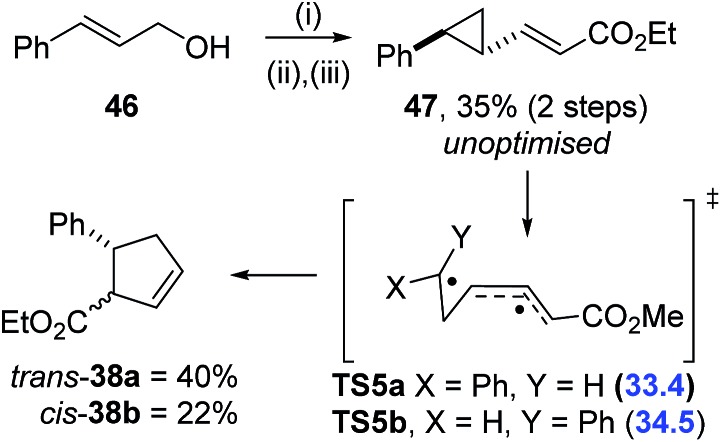
Two step synthesis of non-fluorinated VCP-**47**; activation energies to **TS5** (Δ*G* ^‡^ value (in blue) were calculated using (U)B3LYP/6-31G* from intermediate **47**, gas phase, 298 K, Spartan'10, using conformationally simpler Me ester, units are kcal mol^–1^). Conditions: (i) ZnEt_2_ (1 M in hexane, 5 eq.), CH_2_I_2_ (10 eq.), 0 °C to r.t., 2.5 h; (ii) TEMPO (0.1 eq.), BAIB (1.1 eq.), DCM, r.t., 3.5 h; (iii) Ph_3_P=C(H)CO_2_Et (1.3 eq.), 20 h.

The computational triage was not limited to fluorinated precursors; VCP **47** had a calculated activation barrier of 33.4 kcal mol^–1^ for rearrangement through **TS5** (Scheme 8). Simmons–Smith cyclopropanation^33^ of commercially available cinnamyl alcohol **46**, followed by tandem oxidation/olefination, afforded the desired precursor **47** in an unoptimised 35% yield over two steps. A much higher temperature of 220 °C was required for full conversion of **47** to cyclopentene **38** over 17 hours (Table 6, entry 6), 120 °C higher than required for the corresponding difluorinated precursor **1a**. A mixture of *trans*-**38a** and *cis*-**38b** diastereoisomers was observed and chromatographic separation gave 40% and 22% yields of the two products, respectively. Electronic structure calculations showed that transition states representing the VCPR from both *trans*-**47** (**TS5a**, Δ*G*‡B3LYP = 33.4 kcal mol^–1^) and the corresponding *cis*-isomer (**TS5b**, Δ*G*‡B3LYP = 34.5 kcal mol^–1^) had similar energies and could therefore be competitive at high temperatures.

This result conclusively shows the accelerative effect of *gem*-difluorination, and justifies the decision not to invest time in the synthesis of precursors predicted to have activation energies greater than 30 kcal mol^–1^. In fact, experimental results from compounds **20a** and **21b** suggest that the maximum temperature for synthetically useful VCPR with fluorinated precursors could be much lower than expected.

**Scheme 9:**
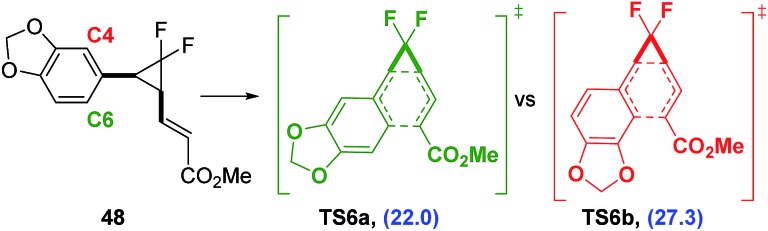
Electronic structure calculations used to predict the favoured [3,3]-rearrangement pathway from **48** (Δ*G* ^‡^ values are relative to **48** (blue) and calculated using UB3LYP/6-31G*, gas phase, 298 K, Spartan'10, units are in kcal mol^–1^).

Piperonyl species **22a** had a much lower predicted activation energy (Δ*G*‡B3LYP = 23.0 kcal mol^–1^) and subsequently rearranged at much lower optimised temperature of 70 °C (Table 6, entry 7). However, like compounds containing the highly activating heteroarene substituents, both difluorocyclopentene **39** and heptadiene **44** were observed and could be isolated after preparative HPLC in low yields (18% and 11% respectively). Diene **44** was the exclusive product of the [3,3]-rearrangement, despite the possibility of the formation of regioisomer **45** *via* reaction at aromatic carbon centre C4 (Scheme 9). Electronic structure calculations were consistent with experimental results, showing that the observed pathway through **TS6a** had a lower activation energy than that through **TS6b** (ΔΔ*G* ^‡^ = 5.3 kcal mol^–1^).

The greater thermal control observed from the rearrangement of **22a**, allowed the competition between VCPR and [3,3]-pathways to be examined fully and for the first time, using VT ^19^F NMR spectroscopy (344–373 K in [*D*_8_]toluene (see the ESI† for experimental details and a full discussion on kinetic modelling)). Unlike the VCPR of phenyl **1a** monitored previously, no evidence of the *cis*-VCP was observed during thermolysis because the [3,3]-pathway was now competitive with VCPR. At this higher rearrangement temperature (100 °C), full consumption of **22a** was observed after 30 minutes, contrasting with the 10 hours required for full conversion of **1a** and providing further experimental evidence that the activation energy for the latter was higher, as predicted. An Arrhenius determination of activation parameters was carried out; the value for VCPR *E*_a_ from piperonyl **22a** of (23.4 ± 0.2) kcal mol^–1^ was very close to the calculated Δ*G*‡B3LYP (23.0 kcal mol^–1^). A slightly higher *E*_a_ value of (24.9 ± 0.3) kcal mol^–1^ was calculated for the [3,3]-pathway; these values were used to screen for the best computational method for treating this manifold of reactions.

### Predictive electronic structure calculations

We carried out an extensive set of calculations to determine the most appropriate methods for the treatment of VCPR and competing [3,3]-rearrangement. The study is reported fully in the ESI† and only the main findings will be summarised here. The UM05-2X/6-31+G* method gave the closest agreement between experimental values for the VCPR of **1a** and **22a**, while the M06-2X/6-31G* method was the best for the [3,3]-rearrangement of **48**; these Minnesota functionals were selected as the most accurate methods for assessing the two pathways. Because of its consistency of performance, the (U)B3LYP/6-31G* was also retained as a low cost method able to handle both rearrangements at a useful level of accuracy. The calculated Δ*G* ^‡^ values for VCPR using each of these methods were corrected by the average error (Δ*G* ^‡^) from **9f** and **9j**; these values are +2.7 kcal mol^–1^ for UB3LYP and –1.2 kcal mol^–1^ for UM05-2X. Since the experimental activation energy for the [3,3]-rearrangement could only be determined for **48**, the correcting values are based solely on this compound's Δ*G* ^‡^ values, which are +2.4 kcal mol^–1^ for B3LYP and +0.5 kcal mol^–1^ for M06-2X.

The differences in corrected Δ*G*‡VCPR and corrected Δ*G*‡[3,3] values ((Δ*G*‡VCPRΔ) – (Δ*G*‡[3,3])) were then used to predict which rearrangement pathway would be favoured by either B3LYP or Minnesota methods; values greater than +1.0 kcal mol^–1^ would favour VCPR and values less than –1.0 kcal mol^–1^ would favour sigmatropic rearrangement. Small differences between these values could result from computational error (±0.5 kcal mol^–1^) and would represent the limit of our ability to predict the composition of rearrangement product mixtures or the identity of the dominant pathway (Table 7).

**Table 7:** Predicting thermal rearrangement pathways (VCPR or [3,3]) from VCPs using corrected free energies of activation (Δ*G* ^‡^)

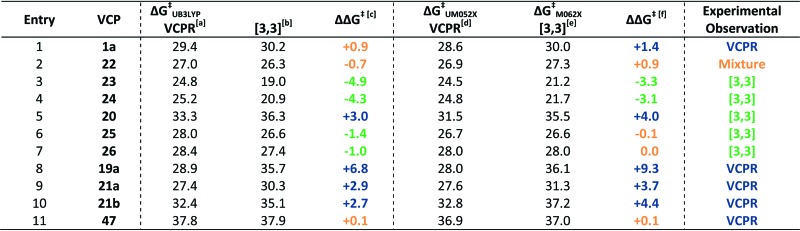

^*a*^Calculated Δ*G* ^‡^ + 2.7 kcal mol^–1^ (UB3LYP/6-31G*).

^*b*^Calculated Δ*G* ^‡^ + 2.4 kcal mol^–1^ (B3LYP/6-31G*).

^*c*^Δ*G* ^‡^ = (corrected VCPR Δ*G*‡UB3LYP) – (corrected [3,3] Δ*G*‡B3LYP).

^*d*^Calculated Δ*G* ^‡^ – 1.2 kcal mol^–1^ (UM05-2X/6-31+G*).

^*e*^Calculated Δ*G* ^‡^ + 0.5 kcal mol^–1^ (M06-2X/6-31G*).

^*f*^Δ*G* ^‡^ = (corrected VCPR Δ*G*‡UM052X) – (corrected [3,3] Δ*G*‡M062X). All calculations were performed in gas phase at 298 K using Gaussian'09, units are in kcal mol^–1^. Blue values predict VCPR pathway, orange values predict a mixture of pathways and green values predict [3,3]-rearrangement.

**Fig. 7:**
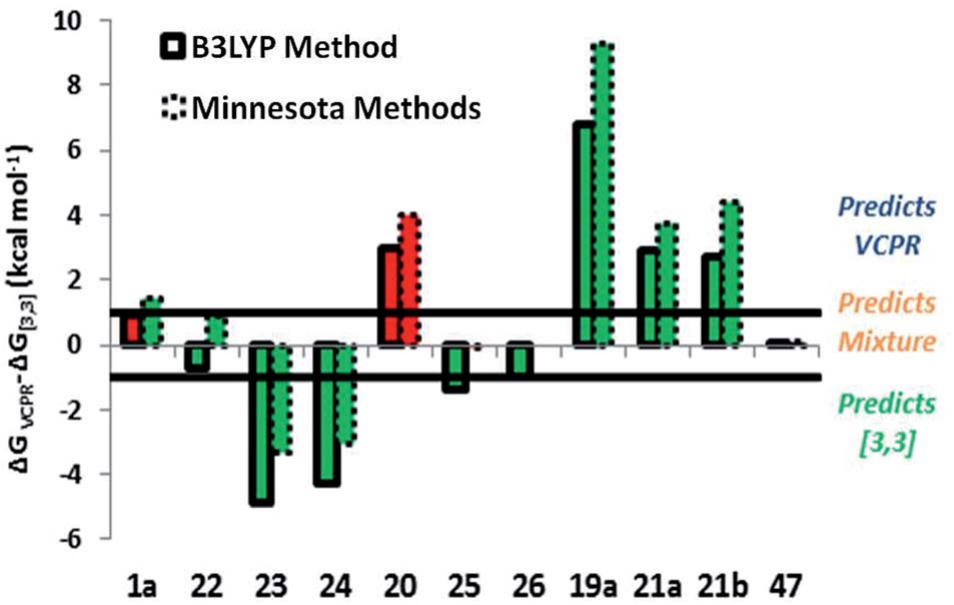
Prediction plot of the difference between corrected Δ*G*‡VCPR (UB3LYP/6-31G* or UM05-2X/6-31+G*) and corrected Δ*G*‡[3,3] (B3LYP/6-31G* or M06-2X/6-31G*, respectively) for synthesised VCP. Colour used to represent the correct (green) or incorrect (red) predictions compared with experimental observations.

Since the correction factors were derived from their experimental values, it was no surprise that phenyl **1a** was correctly predicted to undergo VCPR and that piperonyl **22a** was predicted to give a mixture of products (Table 7, entry 1 and 2, respectively). More pleasingly, the major rearrangement pathways for the nine out of the ten novel difluoro-VCP systems examined were all predicted correctly using B3LYP/6-31G* calculations; for phenyl **1a**, the VCPR pathway was correctly predicted as the major one but is within computational error (Δ*G* ^‡^ = +0.9 kcal mol^–1^, Fig. 7).

**Fig. 8:**
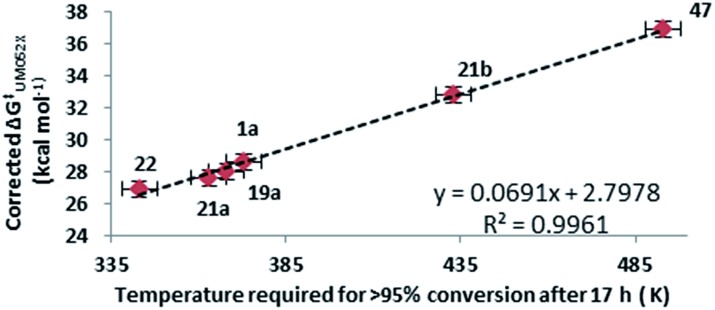
Correlation between corrected Δ*G*‡UM052X values (kcal mol^–1^) for VCPR and the optimised reaction temperatures (K) which gave 100% conversion of VCP. Error in Δ*G*‡UM052X values = ±0.5 kcal mol^–1^. Error in temperature = ±5 K.

Both methods failed to deal with the non-fluorinated system, predicting a mixture of rearrangement products instead of only the VCPR observed experimentally. The Minnesota functionals also failed to deal with more sterically hindered heteroarene VCPs **25** and **26**, but it is unknown whether the errors arise from the VCPR or [3,3]-rearrangement calculations, or from a combination of both. These results strongly suggest that the lowest cost method is comparable and in some case better than the more expensive Minnesota methods (we refer to implementation in Spartan), consistent with studies carried out by Simón and Goodman.^34^ Since all experimental rearrangements were optimised to give full conversion of VCP after 17 hours, a strong trend was apparent between the corrected Δ*G* ^‡^ values for VCPR with reaction temperatures using either M05-2X/6-31+G* (Fig. 8) or B3LYP/6-31G* methods (see ESI†).

As more compounds are synthesised, the error associated with these models may be reduced. However, from the small set of varied difluorinated vinylcyclopropanes that were examined, the most effective computational models look reliable enough to be used with confidence in the design and assessment of new precursors before any synthetic commitments are required.

**Fig. 9:**
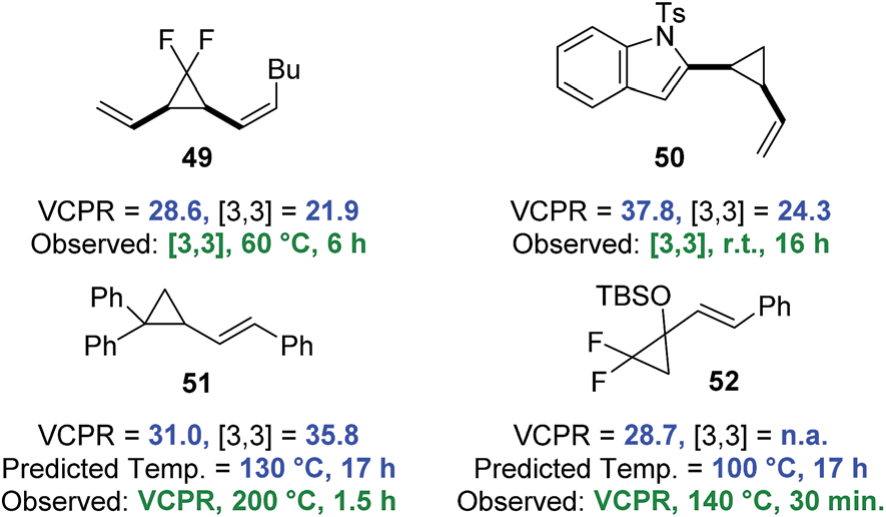
Testing the predictive capability of electronic structure models against compounds which undergo selective VCPR or [3,3]-rearrangement (simplified computational models were used for **50** (Ts replaced with Ms) and **52** (TBS replaced with TMS)). Free energies of activation (Δ*G* ^‡^) calculated on Gaussian'09 using UB3LYP/6-31G* (gas phase, 298 K) and quoted in kcal mol^–1^. Predicted temperature derived from the straight line equation *y* = 0.0721*x* + 2.0341 from a plot of corrected Δ*G*‡B3LYP against VCPR rearrangement temperatures (see ESI†).

A set of VCP from the literature were selected to test the predictive capability of the developed computational models and the rearrangement pathway for all four compounds was successfully predicted using the lower cost UB3LYP/6-31G* method (Fig. 9). The indole-vinylcyclopropane rearrangement of **50** ^8*b*^ and divinylcyclopropane rearrangement of **49** ^8*c*^ were both favoured over VCPR, whereas Sustmann and co-workers bis-aryl VCP **51** was correctly predicted to undergo VCPR.^35^ The calculated Δ*G*‡VCPR value of 31.0 ± 0.5 kcal mol^–1^ for **51** is within error of the reported experimental activation energy of 32.8 ± 1.6 kcal mol^–1^ (corrected to 298 K). Our temperature prediction suggests that the rearrangement could give full conversion after 17 hours at 130 °C, 70 °C lower than the reported conditions (reaction time of 1.5 hours). Difluoro-VCP **52** could only undergo VCPR and a calculated Δ*G*‡VCPR value of 28.7 kcal mol^–1^ was very similar to phenyl-VCP **1a** and predicted to rearrange at the same temperature of 100 °C. These results strongly suggest that the Ni-catalyst present during the reaction of **52** at 140 °C does not facilitate VCPR, a factor that was not obvious from experimental observations.^17*h*^

## Conclusion

A low cost computational assessment (UB3LYP/6-31G*) of substituent effects on the VCPR of difluorinated vinylcyclopropanes was used to guide the synthesis of a test set of novel difluorocyclopropanes. The VCPR system examined is most unusual in that reactions involving open shell singlets and triplet species are concurrent with a more classically concerted pericyclic rearrangement, posing a significant challenge to available computational methods.

Radical stabilising groups, specifically heteroarenes, were found to lower calculated free energies of activation more when bound directly to the cyclopropane instead of to the alkene, consistent with the open shell singlet mechanism for VCPR, and bringing about rearrangements at room temperature in some cases. The most reactive systems based on heteroarenes, underwent rearrangements at unexpectedly low temperatures, through competing VCPR and aromatic-vinylcyclopropane rearrangements to give access to both novel difluorocyclopentenes and fluorinated benzocycloheptadienes, respectively. Optimised rearrangement temperatures for isolated precursors showed a good trend with calculated activation energies, allowing estimates of rearrangement temperatures to be made before synthesis. Comparison of predictions made by electronic structure calculations with experimental activation energies for piperonyl **22a** and literature examples showed that the (U)M05-2X/6-31+G* method remained the most accurate for assessing VCPR, but M06-2X/6-31G* calculations were better for the aromatic-vinylcyclopropane rearrangement. No single method stood out overall but the consistency in error observed with (U)B3LYP/6-31G* calculations for both pathways, meant that it came closest to a universal method for dealing with the reaction manifold. There was no simple relationship between the amount of HF-exchange and the accuracy of the predictions. The selectively for rearrangement pathways could be predicted accurately using electronic structure calculations, either with the Minnesota functionals or lower cost DFT methods ((U)B3LYP/6-31G*). The computational design model developed was tested against literature compounds and was found to predict observed experimental results correctly.

The ability to determine whether a VCP molecule will rearrange thermally at a synthetically useful temperature, and to predict which pathway it will take, is an extremely powerful development, and shows that effective triage of synthetic chemistry programmes is not only effective, but also practicable by non-specialists.
