# Supplementary material for: A computational triage approach to the synthesis of novel difluorocyclopentenes and fluorinated cycloheptadienes using thermal rearrangements
Source: Chem Sci. 2016 Jun 16;7(10):6369–80. doi: 10.1039/c6sc01289b (PMC5355944; doi:10.1039/c6sc01289b)
Supplement: Supplementary file 1 [file SC-007-c6sc01289b-s001.pdf]

# A Computational Triage Approach into the Synthesis of Novel Difluorocyclopentenes and Fluorinated Heptadienes using Thermal Rearrangements

*David Orr, Prof. Dr. Jonathan. M. Percy, and Dr. Zoë A. Harrison*

## Supporting Information


| | |
|---|---|
| **General Experimental** | **S1** |
| **Computational Methodology** | **S3** |
| **Computational Triage Discussion** | **S4** |
| Hammett Plot Data | **S4** |
| Radical Stabilising Effects | **S6** |
| Computational Searches involving Phenolate **S7** | **S8** |
| Regioselectivity with Heterearene Precursors | **S9** |
| **Synthetic Procedures** | **S10** |
| General Procedure A: Synthesis of Alkenoates from Aldehydes | **S10** |
| General Procedure B: Difluorocyclopropanation of Alkenoates | **S16** |
| General Procedure C: Reduction of Difluorocyclopropyl Esters | **S21** |
| General Procedure D: Tandem Oxidation/Olefination of Alcohols | **S25** |
| General Procedure E: Thermal Rearrangement of VCP | **S34** |
| **Characterisation Spectra** | **S41** |
| **Difluorocyclopropanation Optimisation** | **S132** |
| NMR Evidence of Decomposition of Furyl Vinyl Acetate **14a** | **S133** |
| Difluorocyclopropanation of Ethyl Cinnamate with TFDA | **S134** |
| Evidence of Difluorocarbene-Digylme Reaction | **S142** |
| Plot of Conversion vs HOMO Energies | **S143** |
| Functional Group Tolerance: Additive Experiment | **S145** |
| **Crude Spectra for Low Temperature Rearrangement Precursors** | **S148** |
| **Rearrangement of Isolated VCP Precursors – Crude Spectra** | **S155** |
| **Piperonyl 22a Rearrangement: Kinetic Modelling** | **S162** |
| VT $^{19}$F NMR Procedure for Piperonyl **22a** | **S162** |
| Raw Kinetic Data for Piperonyl **22a** | **S164** |
| Rearrangement Reaction Profiles for **22a** (343-373 K) | **S168** |
| NMR Simulation | **S169** |
| Arrhenius Plot for Piperonyl **22a** | **S173** |
| **Computational Methodology Refinement** | **S176** |
| Identification of the most appropriate Method for Electronic Structure Calculations: an Overview | **S176** |
| Basis Set Effect on **22a** VCPR Activation Energies | **S182** |
| Method Screening for VCPR | **S183** |
| Assessment of Solvent Effects | **S184** |
| Investigations into Alternative [3,3]-Diradical/Triplet Pathways | **S186** |
| Arrhenius Plot for divinyl-difluorocyclopropane **49** | **S188** |
| [3,3]-Sigmatropic Computational Methodology Screening | **S189** |
| Error Associated with T1 and G3(MP2) Calculations | **S193** |
| Raw Data for Isodesmic Reactions | **S194** |
| **Free Energy Values for Computational Predictions** | **S196** |
| **VCPR Temperature Predictions** | **S197** |
| **References** | **S198** |
| **Cartesian Coordinates** | **S200** |
| Triage: Cyclopropane Substitution and Alkene Substitution | **S200** |
| Other Synthesised VCP Precursors | **S246** |
| Isodesmic Compounds | **S255** |
| Remaining Species for Prediction Work | **S260** |

| | |
|---|---|
| Literature Prediction | **S277** |
| Supporting Information Compounds | **S287** |


**General Experimental**

NMR spectra were recorded on Bruker DPX-400, AV-500 Avance-II+ 600 spectrometers. $^1$H, $^{19}$F and $^{13}$C NMR spectra were recorded using the deuterated solvent as the lock and the residual solvent as the internal reference. The multiplicities of the spectroscopic data are presented in the following manner: s = singlet, d = doublet, dd = double doublet, ddd = doublet of double doublets, etc up to ddddd, dt = doublet of triplets, ddt = doublet of double triplet, dddt = doublet of double double triplet, dq = doublet of quartet; t = triplet, td = triple doublet; tt = triple triplet, q = quartet, ABq = AB system quartet, m = multiplet and br. = broad. Unless stated otherwise, all couplings refer to $^3J$ homocouplings. All $^1$H spectra are fully assigned as much as possible. Relevant 2D-NMR spectra are supplied to confirm assignment.

IR spectra were recorded on a Shimadzu IRAffinity-1 FT-IR Spectrophotometer using a MIRacle™ Single Reflection Horizontal ATR Accessory.

GC/MS spectra were obtained on an Agilent 7890A GC System fitted with a DB5-type column (30 m × 0.25 µm) running a 40–320 °C temperature program, ramp rate 20 °C min$^{-1}$ with helium carrier gas flow at 1 cm$^3$ min$^{-1}$. Chemical ionisation (CI) (methane) mass spectra were recorded on an Agilent Technologies 5975C mass spectrometer.

HRMS measurements were obtained from a Waters GCT Premier MS (CI-MS), Fiinigan MAT 95 XP (EI-MS and/or APCI-MS) or Thermo Scientific LTQ Orbitrap XL via Advion TriVersa NanoMate infusion (NSI-ES) spectrometers (EPSRC National Mass Spectrometry Service Centre, Swansea).

Melting points were recorded on a Griffin melting point apparatus and are uncorrected.

Thin layer chromatography was performed on pre-coated aluminium-backed silica gel plates (E.Merck, A.G.Darmstadt, Germany. Silica gel 60 F254, thickness 0.2 mm). Visualisation was achieved using potassium permanganate or UV detection at 254 nm. Column

chromatography was performed on silica gel (Zeochem, Zeoprep 60 HYD, 40-63 µm) using a Büchi Sepacore system. Hexane was distilled before chromatography.

Preparative HPLC was performed using Grace Reveleris PREP purification system with a Kromasil 100-10-C18 column (L = 250 mm, ID = 200 mm). Compound was loaded in a minimum volume of 1:1 DMSO:MeOH in a 5 mL sample loop. Flow rates varied depending on separation (5-30 ml/min) and peaks determined using ELSD and UV detection.

Commercial furfural (black) was percolated through a pad of alumina to afford an orange solution which was used immediately in reactions. 2-Formyl-pyrrole-1-carboxylic acid tert-butyl ester **12f** was synthesised from NaH and Boc anhydride using conditions described by Carreira and co-workers.[S1] 2-thiazolyl-carboxaldehyde **12g** was synthesised from thiazole, BuLi and DMF using conditions described by Glorius and co-workers.[S2] Due to the volatility of the aldehyde, crude product was used directly in subsequent Wittig olefinations.

Diglyme was distilled from $CaH_2$ (60 °C/23 mbar) and stored under nitrogen over $CaH_2$. Trimethylsilyl chloride was distilled from $CaH_2$ (60 °C/430 mbar) and stored under nitrogen over $CaH_2$ in the refrigerator. Methyl 2,2-difluoro-2-(fluorosulfonyl)acetate (MDFA) and trimethylsilyl 2,2-difluoro-2-(fluorosulfonyl)acetate (TFDA) were purchased from Fluorochem and stored under a headspace of nitrogen.Potassium iodide (Sigma Aldrich) and magnesium sulfate ($MgSO_4$, VWR Chemicals) were dried in the oven (150 °C) before use. DCM (for tandem oxidation/olefination, acetylation and cyclopropanation reactions) and toluene (for reduction reactions) were dried using a PureSolv system from Innovative Technology, Inc..

All other chemicals were purchased from Sigma Aldrich, Apollo Scientific, Alfa Aesar, or Fluorochem and used as received.

**Computational Methodology**

Structures were built in Spartan'10 using previously published compounds as models and geometry optimisation calculations were carried out for all closed shell compounds. For known diradical compounds unrestricted methodology was invoked using the keywords MIX and SCF=UNRESTRICTED, with CONVERGE deprecated. In cases where SCF convergence was difficult, the keyword NODIIS was used. All calculations were performed *in vacuo*. Spartan'10 was run on a Dell Precision T7500 (2 x Intel E5530 processors, four cores each, 2.40 GHz) with 24 GB RAM Debian GNU/Linux 5.

The calculation methods used in Spartan have been documented in: Y. Shao, L.F. Molnar, Y. Jung, J. Kussmann, C. Ochsenfeld, S.T. Brown, A.T.B. Gilbert, L.V. Slipchenko, S.V. Levchenko, D.P. O'Neill, R.A. DiStasio Jr., R.C. Lochan, T. Wang, G.J.O. Beran, N.A. Besley, J.M. Herbert, C.Y. Lin, T. Van Voorhis, S.H. Chien, A. Sodt, R.P. Steele, V.A. Rassolov, P.E. Maslen, P.P. Korambath, R.D. Adamson, B. Austin, J. Baker, E.F.C. Byrd, H. Dachsel, R.J. Doerksen, A. Dreuw, B.D. Dunietz, A.D. Dutoi, T.R. Furlani, S.R. Gwaltney, A. Heyden, S. Hirata, C-P. Hsu, G. Kedziora, R.Z. Khalliulin, P. Klunzinger, A.M. Lee, M.S. Lee, W.Z. Liang, I. Lotan, N. Nair, B. Peters, E.I. Proynov, P.A. Pieniazek, Y.M. Rhee,J. Ritchie, E. Rosta, C.D. Sherrill, A.C. Simmonett, J.E. Subotnik, H.L. Woodcock III, W. Zhang, A.T. Bell, A.K. Chakraborty, D.M. Chipman, F.J. Keil, A.Warshel, W.J. Hehre, H.F. Schaefer, J. Kong, A.I. Krylov, P.M.W. Gill and M. Head-Gordon, *Phys. Chem. Chem. Phys*., **2006**, *8*, 3172.

Full reference for the Gaussian09 programme: Gaussian 09, Revision A.1, Frisch, M. J.; Trucks, G. W.; Schlegel, H. B.; Scuseria, G. E.; Robb, M. A.; Cheeseman, J. R.; Scalmani, G.; Barone, V.; Mennucci, B.; Petersson, G. A.; Nakatsuji, H.; Caricato, M.; Li, X.; Hratchian, H. P.; Izmaylov, A. F.; Bloino, J.; Zheng, G.; Sonnenberg, J. L.; Hada, M.; Ehara, M.; Toyota, K.; Fukuda, R.; Hasegawa, J.; Ishida, M.; Nakajima, T.; Honda, Y.; Kitao, O.; Nakai, H.; Vreven, T.; Montgomery, Jr., J. A.; Peralta, J. E.; Ogliaro, F.; Bearpark, M.; Heyd, J. J.; Brothers, E.; Kudin, K. N.; Staroverov, V. N.; Kobayashi, R.; Normand, J.; Raghavachari, K.; Rendell, A.; Burant, J. C.; Iyengar, S. S.; Tomasi, J.; Cossi, M.; Rega, N.; Millam, N. J.; Klene, M.; Knox, J. E.; Cross, J. B.; Bakken, V.; Adamo, C.; Jaramillo, J.; Gomperts, R.; Stratmann, R. E.; Yazyev, O.; Austin, A. J.; Cammi, R.; Pomelli, C.; Ochterski, J. W.; Martin, R. L.; Morokuma, K.; Zakrzewski, V. G.; Voth, G. A.; Salvador, P.; Dannenberg, J. J.; Dapprich, S.; Daniels, A. D.; Farkas, Ö.; Foresman, J. B.; Ortiz, J. V.; Cioslowski, J.; Fox, D. J. Gaussian, Inc., Wallingford CT, **2009**.

**Hammett Plot Data**

The lower activation energy calculated for the more electron rich phenyl-substituted **9f** (-2.3 kcal $mol^{-1}$) prompted further investigations into the electronic effects of phenyl ring substitution. The VCPR activation energies for a range of precursors with electron-donating and -withdrawing groups were assessed (**Table S1**), focusing solely on *para*-substituents, since these show the greatest effects for resonance stabilisation of benzylic radicals. Substrates containing electron-donating groups were predicted to undergo faster rearrangement (**Table S1**, Entries 1-6) than unsubstituted **9j** (**Table S1**, Entry 7), whereas substrates bearing electron-withdrawing groups only showed a modest increase in activation energy (**Table S1**, Entries 8-12). The Hammett plot[S3] (**Figure S1**) constructed from this data gave an extremely low $\rho$ value of 0.05 and supported a radical mechanism. A variety of free radical substituent constants ($\sigma^{\bullet}$) have been reported in the literature[S4-7] but only those reported by Creary and co-workers ($\sigma_C^{\bullet}$) are applicable to the more commonly used substituents.[S8] Our calculated activation energies correlated poorly when $\sigma_C^{\bullet}$ constants were used ($R^2$ = 0.4259) but still gave a $\rho$ value close to zero (**Figure S1**). The relatively small increases in activation barriers, suggest that even substrates bearing electron-deficient phenyl rings should undergo VCPR at or close to 100 °C.

Both Hammett plots were generated using Excel from calculated free energies of activation ((U)B3LYP/6-31G*, gas phase, 298 K, kcal $mol^{-1}$) manipulated into $\log(\Delta G^{\ddagger}(X)/\Delta G^{\ddagger}(H))$ values and literature σ values[S3] (**Figure S1**) or $\sigma^{\bullet}$ values[S8] (**Table S1** and **Figure S2**). Graphs were generated on Excel and a straight line fitted using the trendline function for **Figure S1** and **Figure S2**.

**Table S1:** Calculated free energies of activation (298 K) and σ• data used to generate Hammett Plot in **Figure S1**.

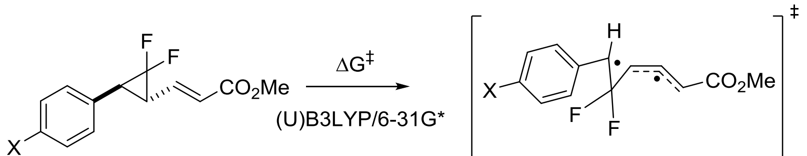


**S1** **S-TS1**

| VCP | X | $\Delta G^{\ddagger}$ | $\Delta\Delta G^{\ddagger}$ | log (ΔG(X)/ΔG (X=H)) | $\sigma^{S3}$ | σ• ref. S8 |
|---|---|---|---|---|---|---|
| **a** | $N(Me)_2$ | 21.2 | -4.1 | -0.056 | -0.83 | 0.9 |
| **b** | $NH_2$ | 21.9 | -3.4 | -0.042 | -0.66 | 0.7 |
| **c** | OH | 23.5 | -1.8 | -0.011 | -0.37 | 0.26 |
| **d** | OMe | 23.3 | -2.0 | -0.015 | -0.27 | 0.27 |
| **e** | NHC(O)Me | 23.7 | -1.6 | -0.007 | -0.17 | 0.34 |
| **f** | Me | 24.5 | -0.8 | -0.072 | 0 | 0.16 |
| **9j** | H | 25.3 | 0 | 0.000 | *0* | - |
| **i** | Cl | 25.3 | 0 | 0.021 | 0.23 | 0.11 |
| **j** | Br | 25.4 | 0.1 | 0.023 | 0.23 | 0.11 |
| **k** | $CF_3$ | 25.4 | 0.1 | 0.023 | 0.54 | 0.05 |
| **l** | CN | 25.6 | 0.3 | 0.026 | 0.66 | 0.47 |
| **m** | $NO_2$ | 25.9 | 0.6 | 0.031 | 0.78 | 0.57 |

[a] $\Delta\Delta G^{\ddagger}_{B3LYP} = (\Delta G^{\ddagger}_{B3LYP}$ **S1x**$)-(\Delta G^{\ddagger}_{B3LYP}$ **9j**$)$

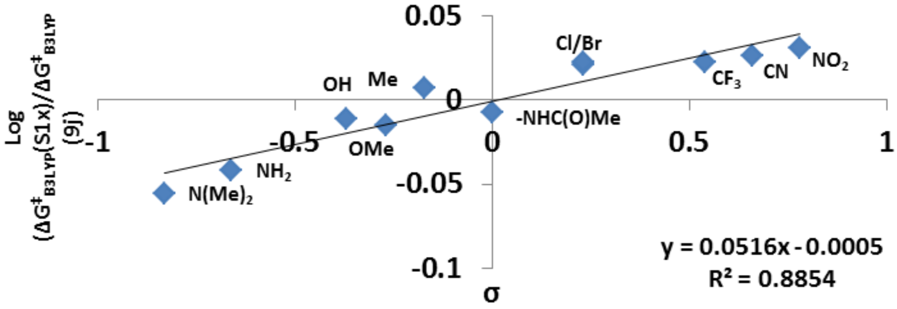


**Figure S1:** Hammett plot generated from calculated free energies of activation and σ values.

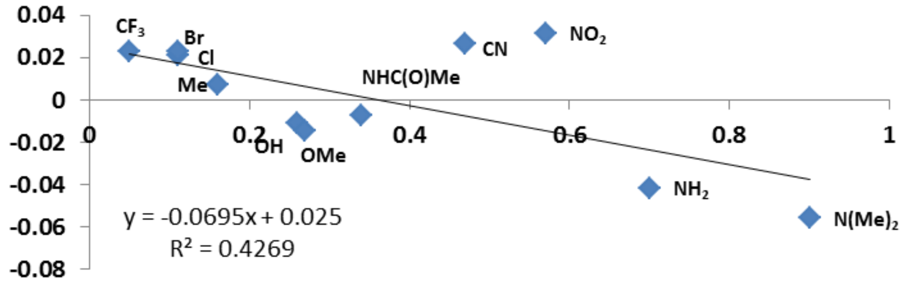


**Figure S2:** Hammett plot generated from calculated free energies of activation and $\sigma^{\bullet}$ values.

## Radical Stabilising Effects

As our prime goal was to lower VCPR reaction temperatures, our screening was directed towards vinyl, (hetero)aryl and alkynyl substituents because the most effective mechanism for radical stabilisation is delocalisation through adjacent π-systems.[S9,10] The lower activation energy observed for **9g** (ethenyl, 24.5 kcal mol$^{-1}$) compared with **9j** (phenyl, 25.3 kcal mol$^{-1}$) and **9l** (ethynyl, 25.9 kcal mol$^{-1}$) showed good correlation with the increased radical stabilisation energy reported for these substituents (calculated as 17.6, 14.6 and 14.5 kcal mol$^{-1}$, respectively).[S10] The higher activation energy observed for **9p** (cyano, 29.3 kcal mol$^{-1}$) can be rationalised by a decreased radical stabilisation energy (7.9 kcal mol$^{-1}$),[S10] which arises from the higher electronegativity of the nitrogen atom. These calculations suggested that *bis*-vinyl VCP-**9g** has the potential to rearrange at temperatures lower than 100 °C, but electronic structure calculations predicted that *cis*-isomer **S2** would favour divinylcyclopropane rearrangement via **S-TS2** ($\Delta G^{\ddagger}_{B3LYP}$ = 13.5 kcal mol$^{-1}$, VCPR vs [3,3] difference of 11.5 kcal mol$^{-1}$, **Scheme S1a**). Erbes and Boland showed experimentally that a similar dialkenyl-substituted *gem*-difluorocyclopropane (**S3**) exclusively favoured the [3,3]-pathway through to heptadiene **S4** (**Scheme S1b**).[S11] Dialkenyl-**9g** was omitted from any subsequent synthetic investigations due to the unlikelihood of selective VCPR.

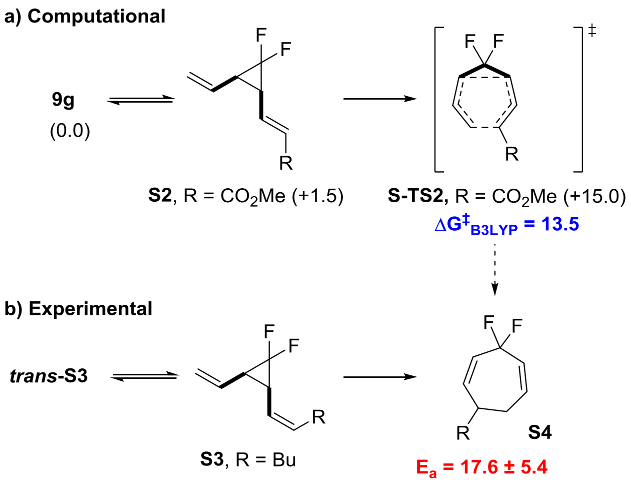


**Scheme S1:** a) Predicted lower energy [3,3]-pathway for bis-vinyldifluorocyclopropane **S2** (free energies are relative to **9h**). b) Experimental example of preferential divinylcyclopropane rearrangement from bis-vinyl-difluorocyclopropane **S3** to difluoroheptadiene **S4**[S11] (experimental activation energy ($E_a$) is derived from an Arrhenius determination of reported kinetic data[S11]).

Of the heteroaromatic substituents examined, only the 2- and 4-pyridyl species gave higher VCPR barriers compared with **9j** (**9o**, +2.9 kcal/mol$^{-1}$ and **9m**, +1.1 kcal mol$^{-1}$); the nitrogen atom cannot help stabilise radicals in these cases. Creary and co-workers reported similar results during investigations into the radical rearrangement of methylenecyclopropane **S5** to isopropylidenecyclopropane **S6** (**Scheme S2**).[S8,12]

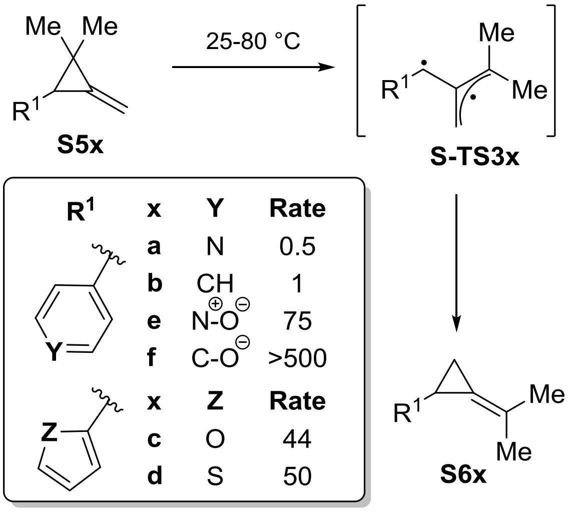


**Scheme S2:** Substituent group radical stability determined from the rate of rearrangement of **S5** to **S6**.[S8]

They reported higher rearrangement rates when heterocycles **S5c-e** and phenolate **S5f** were present. These substituents were dubbed "super radical stabilisers". Unfortunately all computational attempts at incorporating the most strongly-activating 4-phenoxide

substituent into a VCPR failed; a diradicaloid transition state could not be obtained. Ring opening occurred via a donor-acceptor mechanism when the rearrangement precursor was optimised[S13,14] (*vida infra*).

**Computational Searches Involving Phenolate S7**

Intermediate VCP **S7** and transition state **S-TS4** were built from previously optimised structure of phenyl VCP **9j** and **TS1j** and optimised using B3LYP/6-31G* or UB3LYP/6-31G*, respectively (Spartan'10, gas phase, 298 K, multiplicity = 1). It was observed that closed shell intermediate **S7** optimised to ring opened cyclopropane intermediate **S8** (Scheme **S3a**); desired VCP **S7** was never observed after optimisation. An optimised transition state structure could be obtained from **S-TS4** ($\nu_i$ = i107 $cm^{-1}$) but the lack of diradical character ($S^2$ = 0.0001) suggested that the process had a donor-acceptor mechanism rather than VCPR (Scheme **S3b**).

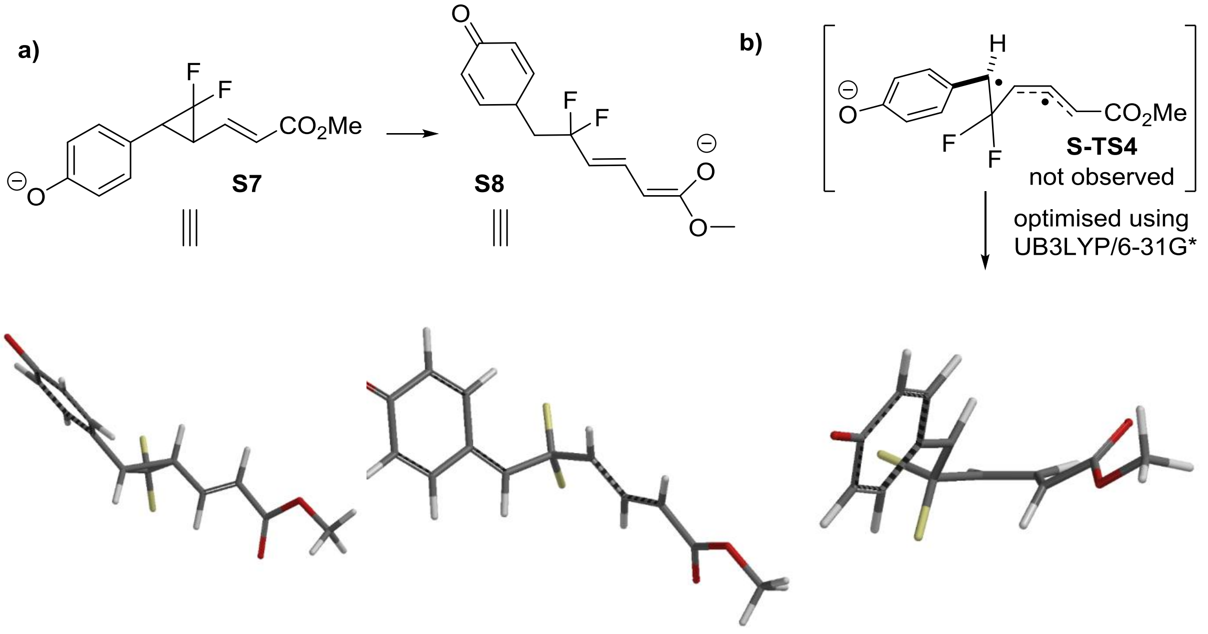


**Scheme S3:** Failed attempts at obtaining optimised structures for a) intermediate **S7** and b) transition state **STS4**. The resulting structures strongly suggest the process proceeds *via* a donor-acceptor mechanism.

## Regioselectivity with Heteroarene Precursors

When, the heteroarenes were embedded in our VCPR system, the calculated activation energies (for **9b-9d**) were consistently lower (ranging from -5.3 to -4.2 kcal mol$^{-1}$ lower) than for phenyl **9j**. Two regioisomeric transition-states exist for the unsymmetrical species; the 2-pyrrolyl and 2-thiophenyl species favoured **TS1a** whilst the 2-furyl favoured **TS1b** (**Figure S3a**).

a)

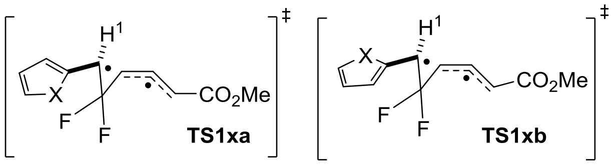


| X | x | ΔG‡ |
|---|---|---|
| NH | a | 16.8 |
| O | b | 23.1 |
| S | d | 21.3 |

| X | x | ΔG‡ | X...H¹ (Å) |
|---|---|---|---|
| NH | a | 19.7 | n.d. |
| O | b | 21.0 | 2.56 |
| S | d | 21.7 | 2.88 |

b)

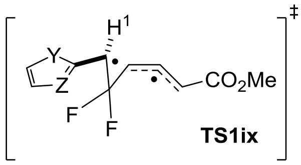


| Y | Z | x | ΔG‡ | X...H¹ (Å) |
|---|---|---|---|---|
| N | S | a | 24.1 | 2.60 |
| S | N | b | 27.1 | 2.88 |

**Figure S3:** Two alternative VCPR transition states available for difluoro-VCP substituted with heteroarenes ($\Delta G^{\ddagger}$ energies calculated on Spartan'10, (U)B3LYP/6-31G*, 298 K, gas phase, energies quoted in kcal mol$^{-1}$).

We propose that the lower energies observed for **TS1bb** compared with **S-TS1ba** arose from a complementary polar interaction between the $\delta^{+}$ proton ($H^1$) and the oxygen atom in the furan ring (calculated distances are within the sum of the Van der Waals radii used in Spartan'10). The absence of additional stabilisation observed in **S-TS1cb** over **S-TS1ca** can be attributed to lower strength S...H interactions. This was consistent with calculated activation energies for thiazolyl **TS1i**, favouring the stronger N...H interaction over S...H (**Figure S3b**).[S15] Furthermore, 2-pyrrolyl **9a** favoured **S-TS1xa** due to an unfavourable N-H...H interaction in **S-TS1ab.** The extra stabilisation experience by furyl **9b** over thiophenyl **XXc** offered some explanation as to why our calculated rearrangement rates were ordered differently from those reported by Creary and co-workers. Our lowest calculated activation energy was observed with 2-pyrrolyl **9a** (-8.5 kcal mol$^{-1}$), a substituent which to our knowledge has not previously been reported as a strong radical stabiliser.

**Synthetic Procedures**

**General Procedure A:** Synthesis of Alkenoates from Commercial Aldehydes

**Preparation of ethyl 3-(2'-furyl) prop-2*E*-enoate (13a)**

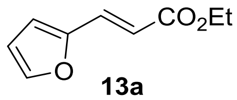


**13a**

(Carbethoxmethylene)triphenylphosphorane (3.7 g, 10.4 mmol) was added to a solution of furfural **12a** (0.79 mL, 9.5 mmol) in anhydrous DCM (50 mL) and the reaction mixture stirred at room temperature under nitrogen for 7 hours. An aliquot was taken and TLC confirmed full conversion of aldehyde. The reaction mixture was concentrated under reduced pressure and column chromatography on silica gel (1:9 $Et_2O$ in hexane) afforded alkenoate **13a** (1.36 g, 82%) as a yellow oil. $R_f$ = 0.31 (1:4 $Et_2O$ in hexane); $^1$H NMR (400 MHz, $CDCl_3$): δ = 7.49 (d, *J* = 1.8 Hz; Ar*H*, 1H), 7.45 (d, *J* = 15.8 Hz, ArC*H*=CH, 1H), 6.62 (d, *J* = 3.4 Hz, Ar*H*, 1H), 6.48 (dd, *J* = 3.4, 1.8 Hz, Ar*H*, 1H), 6.33 (d, *J* = 15.8 Hz, ArCH=C*H*, 1H), 4.26 (q, *J* = 7.1 Hz, $OCH_2CH_3$, 2H), 1.34 ppm (t, *J* = 7.1 Hz, $OCH_2CH_3$, 3H); $^{13}$C NMR (100 MHz, $CDCl_3$): δ = 166.5, 150.5, 144.1, 130.4, 115.5, 114.0, 111.7, 59.9, 13.8 ppm; ν̄/(film) = 2361, 2342, 1703, 1638, 1302, 1258, 1207, 1159, 1017 cm$^{-1}$; MS (CI): m/z (%): 195 (12) $[M+C_2H_5]^+$, 167 (100) $[M+H]^+$, 139 (61), 121 (80) $[M-OEt]^+$; $t_R$ (GC) = 9.62 minutes. The data was in agreement with that reported by Lebel and Davi.[S16]

**Preparation of (*E*)-3-(furan-2-yl)allyl acetate (14a)**

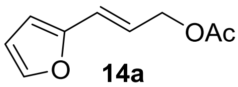


**14a**

Diisobutylaluminium hydride (14.0 mL of a 1.1 M solution in cyclohexane, 15.0 mmol)) was added dropwise to a solution of ester **13a** (1.12 g, 5.0 mmol) in anhydrous toluene (40 mL) under nitrogen at 0 °C for 10 minutes. The reaction mixture was allowed to warm to room temperature and stirred for a further 8 hours, cooled to 0 °C and quenched by dropwise addition of $H_2O$ (1 mL), 0.1 M aqueous NaOH (1 mL) and $H_2O$ (1 mL) in that order. $MgSO_4$ was added to quenched mixture until the solid was free flowing and the mixture was left overnight at room temperature (17 h). The resulting white emulsion was washed with EtOAc (3 x 50 mL) and the organic extracts were combined and concentrated under reduced

pressure. $^{1}$H NMR spectrum confirmed full conversion to the corresponding alcohol (603 mg, 97%) which was directly acetylated without further purification. Acetic anhydride (0.78 ml, 8.3 mmol) was added dropwise to a solution of crude alcohol (860 g, 6.9 mmol) and pyridine (0.67 ml, 8.3 mmol) in DCM (7 mL) under nitrogen and the reaction mixture was heated to 45 °C for 5 hours. The TLC of the reaction mixture showed complete consumption of alcohol. $H_2O$ (20 mL) was added to the reaction mixture and the organic layer separated. The aqueous layer was extracted with DCM (2 x 10 mL) and the organic layer and extracts were combined and backwashed with HCl (2 x 20 mL of a 1 M aqueous solution), NaOH (3 x 20 mL of a 10 M aqueous solution) and aqueous NaCl (20 mL) in that order. The resulting organic layer was dried ($MgSO_4$) and concentrated under reduced pressure. Any remaining pyridine in the resulting oil was removed by co-evaporation with toluene (2 x 25 mL) to afford acetate **14a** as a colourless oil (950 g, 83%). $R_f$ = 0.43 (1:4 $Et_2O$ in hexane); $^{1}$H NMR (400 MHz, $CDCl_3$): δ = 7.37 (d, *J* = 1.8 Hz, Ar*H*, 1H), 6.49 (dt, *J* = 15.8 Hz, $^{4}J$ = 1.3 Hz, ArC*H*=CHC$H_2$, 1H), 6.40 (dd, *J* = 3.5, 1.8 Hz, Ar*H*, 1H), 6.30 (d, *J* = 3.4 Hz, Ar*H*, 1H), 6.23 (dt, *J* = 15.8, 6.5 Hz, CH=C*H*C$H_2$, 1H), 4.72 (dd, *J* = 6.4 Hz, $^{4}J$ = 1.3 Hz, C$H_2$OAc, 2H), 2.12 ppm (s, COC$H_3$, 3H); $^{13}$C NMR (100 MHz, $CDCl_3$): δ = 170.3, 151.3, 141.9, 121.6, 121.2, 110.9, 108.4, 64.1, 20.5 ppm; $\bar{\nu}$/(film) = 2359, 1734, 1377, 1360, 1223, 1013 $cm^{-1}$; MS (CI): *m/z* (%): 166 (5) [M], 123 (8) $[M\text{-}COMe]^+$, 107 (100) $[M\text{-}OAc]^+$, 61 (75); $t_R$ (GC) = 10.26 minutes. The data were in agreement with those reported by Iwasaki and co-workers.[S17]

**Preparation of cinnamyl acetate (13b)**

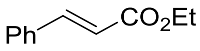

**13b**

Ester **13b** was prepared from benzaldehyde **12b** (1.06 ml, 10 mmol), (carbethoxmethylene)triphenylphosphorane (3.83 g, 11.0 mmol) and DCM (20 ml) according to General Procedure A. Column chromatography on silica gel (1:4 $Et_2O$ in hexane) afforded ester **13b** as a yellow oil (930 mg, 87%). $^{1}$H NMR data was consistent with a commercial sample.

**Preparation of ethyl 3-(2'-thiophenyl)-prop-2*E*-enoate (13c)**

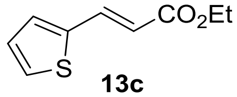

**13c**

Ester **13c** was prepared from thiophene-2-carbaldehyde **12c** (0.92 ml, 10 mmol), (carbethoxmethylene)triphenylphosphorane (4.6 g, 11.2 mmol) and DCM (30 ml) over 20 hours according to General Procedure A. Column chromatography on silica gel (1:4 $Et_2O$ in hexane) afforded ester **13c** as a yellow oil (1.79 g, 98%). $R_f$ = 0.31 (1:1 $Et_2O$ in hexane); $^1$H NMR (400 MHz, $CDCl_3$): δ = 7.81 (d, *J* = 15.7 Hz, ArC*H*=CH, 1H), 7.39 (d, *J* = 5.1 Hz, Ar*H*, 1H), 7.27 (d, *J* = 3.6 Hz, Ar*H*, 1H), 7.07 (dd, *J* = 5.1, 3.6 Hz, Ar*H*, 1H), 6.26 (d, *J* = 15.7 Hz, ArCH=C*H*, 1H), 4.27 (q, *J* = 7.0 Hz, $OCH_2CH_3$, 2H), 1.35 ppm (t, *J* = 7.0 Hz, $OCH_2CH_3$, 3H); $^{13}$C NMR (100 MHz, $CDCl_3$): δ = 166.4, 139.1, 136.5, 130.3, 127.8, 127.6, 116.5, 60.0, 13.8 ppm; $\bar{\nu}$/(film) = 2358, 2342, 1703, 1624, 1369, 1304, 1202, 1159,1042 $cm^{-1}$; MS (CI): m/z (%): 211 (13) $[M+C_2H_5]^+$, 183 (100) $[M+H]^+$, 137 (80) $[M-OEt]^+$; $t_R$ (GC) = 11.85 minutes. The data was in agreement with that reported by Lebel and Davi.[S16]

**Preparation of ethyl 3-(5'-benzo[*d*][1,3]dioxolyl)-prop-2*E*-enoate (13d)**

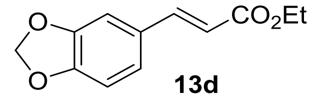

**13d**

Ester **13d** was prepared from benzo[*d*][1,3]dioxole-5-carbaldehyde **12d** (1.39 g, 6.3 mmol), (carbethoxmethylene)triphenylphosphorane (3.47 g, 8.4 mmol) and DCM (30 ml) over 18 hours according to General Procedure A. Column chromatography on silica gel (1:4 $Et_2O$ in hexane) afforded ester **13d** as a white solid (1.88 g, 98%). m.p. = 63-65 °C (obtained from powdered solid isolated after chromatography); $R_f$ = 0.38 (1:4 $Et_2O$ in hexane); $^1$H NMR (400 MHz, $CDCl_3$): δ = 7.63 (d, *J* = 15.9 Hz, ArC*H*=CH, 1H), 7.06 (d, $^4J$ = 1.6 Hz, Ar*H*, 1H), 7.03 (dd, *J* = 8.1, $^4J$ = 1.6 Hz, Ar*H*, 1H), 6.83 (d, *J* = 8.1 Hz, Ar*H*, 1H), 6.29 (d, *J* = 15.9 Hz, ArCH=C*H*, 1H), 6.03 (s, $O_2CH_2$, 2H), 4.28 (q, *J* = 7.1 Hz, $OCH_2CH_3$, 2H), 1.35 ppm (t, *J* = 7.1 Hz, $OCH_2CH_3$, 3H); $^{13}$C NMR (100 MHz, $CDCl_3$): δ = 166.7, 149.1, 147.8, 143.8, 128.4, 123.9, 115.7, 108.1, 106.0, 101.1, 59.9, 13.9 ppm; $\bar{\nu}$/(film) = 2359, 2342, 1701, 1632, 1489, 1445, 1236, 1163 $cm^{-1}$; MS (CI): m/z (%): 249 (19) $[M+C_2H_5]^+$, 221 (95) $[M+H]^+$, 193 (12), 175 (100) $[M-OEt]^+$, 147 (10) $[M-CO_2Et]^+$; $t_R$ (GC) = 13.91 minutes. The data was in agreement with that reported by Lebel and Davi.[S16]

**Preparation of ethyl 3-(2'-pyridyl)-prop-2*E*-enoate (13e)**

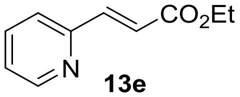

**13e**

Ester **13e** was prepared from picolinaldehyde **12e** (0.57 ml, 6 mmol), (carbethoxmethylene)triphenylphosphorane (2.7 g, 6.6 mmol) and DCM (25 ml) according to General Procedure A. Column chromatography on silica gel (1:1 $Et_2O$ in hexane) afforded ester **13e** as a yellow oil (930 mg, 87%). $R_f$ = 0.34 (1:1 $Et_2O$ in hexane); $^1$H NMR (400 MHz, $CDCl_3$): δ = 8.66 (br. d, *J* = 4.7 Hz, Ar*H*, 1H), 7.72 (td, *J* = 7.8 Hz, $^4J$ = 1.9 Hz, Ar*H*, 1H), 7.70 (d, *J* = 15.7 Hz, ArC*H*=CH, 1H), 7.44 (dt, *J* = 7.8 Hz, $^4J$ = 1.0 *Hz*, Ar*H*, 1H), 7.27 (ddd, *J* = 7.8, 4.7 Hz, $^4J$ = 1.0 Hz, Ar*H*, 1H), 6.93 (d, *J* = 15.7 Hz, ArCH=C*H*, 1H), 4.29 (q, *J* = 7.14 Hz, $OCH_2CH_3$, 2H), 1.35 ppm (t, *J* = 7.14 Hz, $OCH_2CH_3$, 3H); $^{13}$C NMR (100 MHz, $CDCl_3$): δ = 166.3, 152.5, 149.6, 142.8, 136.2, 123.7, 123.6, 122.0, 60.2, 13.8 ppm; ν̄/(film) = 2981, 2359, 2342, 1717, 1201, 1167 $cm^{-1}$; MS (CI): m/z (%): 218 (5) $[M+C_3H_5]^+$, 206 (10) $[M+C_2H_5]^+$, 178 (100) $[M+H]^+$, 150 (15), 132 (20) $[M-OEt]^+$; $t_R$ (GC) = 12.10 minutes. The data was in agreement with that reported by Lebel and Davi.[S16]

**Preparation of ethyl 3-(2'-pyrrolyl-1-carboxylic acid *tert*-butyl ester)-prop-2*E*-enoate (13f)**

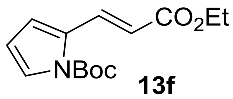

**13f**

Ester (**13f**) was prepared from aldehyde **12f** (3.9 g, 20 mmol), (carbethoxmethylene)triphenylphosphorane (9.25 g, 26 mmol) and DCM (125 ml) according to General Procedure A. Column chromatography (1:19 $Et_2O$ in hexane to 1:4 $Et_2O$ in hexane) afforded alkenoate **13f** as a yellow oil (3.4 g, 60%). $R_f$ = 0.40 (1:4 $Et_2O$ in hexane); $^1$H NMR (400 MHz, $CDCl_3$): δ = 8.30 (d, *J* = 15.9 Hz, ArC*H*=CH, 1H), 7.42 (br. dd, *J* = 3.6 Hz, $^4J$ = 1.6 Hz, Ar*H*, 1H), 6.72-6.70 (m, Ar*H*, 1H), 6.23 (t, *J* = 3.6 Hz, Ar*H*, 1H), 6.22 (d, *J* = 15.9 Hz, CH=C*H*$CO_2$Et, 1H), 4.26 (q, *J* = 7.1 Hz, $OCH_2CH_3$, 2H), 1.65 (s, $OC(CH_3)_3$, 9H), 1.3 ppm (t, *J* = 7.1 Hz, $OCH_2CH_3$, 3H); $^{13}$C NMR (100 MHz, $CDCl_3$): δ = 166.6, 148.5, 134.4, 130.6, 124.3, 116.1, 114.3, 110.9, 84.4, 59.8, 27.5, 13.9 ppm; ν̄/(film) = 2976, 1742, 1705, 1621, 1316, 1299, 1245, 1158, 1117 $cm^{-1}$; MS (CI): m/z (%): 194 (12) $[M+ C_2H_5\text{-Pyrroyl-N-Boc}]^+$, 166 (50) $[M\text{-Pyrroyl-N-Boc}]^+$, 120 (100); $t_R$ (GC) = 12.58 minutes. The data was in agreement with that reported by Jeffrey and co-workers.[S18]

**Preparation of ethyl 3-(2'-thiazolyl)-prop-2*E*-enoate (13g)**

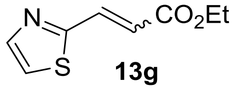

**13g**

Aldehyde **12g** was synthesised from thiazole (1.75 mL, 25 mmol), DMF (3.9 mL, 20 mmol), BuLi (14.3 mL of a 2.1 M in THF, 30 mmol) in THF (100 mL) according to Glorius and co-workers[S2] as a yellow oil. $^1$H NMR confirmed full conversion to aldehyde. Ester **13f** was prepared from crude aldehyde **12g** (assumed 25 mmol), (carbethoxmethylene)triphenylphosphorane (9.75 g, 27 mmol) and DCM (150 ml) according to general procedure A. Column chromatography (7:20 $Et_2O$ in hexane) afforded ester **13f** as a yellow oil (3.38 g, 74%) with a 4:1 mixture of alkene isomers (*E*-isomer major). **Data for Mixture:** $R_f$ = 0.20 (1:4 $Et_2O$ in hexane); $\bar{\nu}$/(film) = 2978, 1708, 1630, 1468, 1480, 1299, 1266, 1175 1030 cm$^{-1}$; HRMS (FTMS): calcd. for $C_8H_{10}O_2N_1S_1$, 184.0423 $[M+H]^+$ found 184.0427. **Data for *E*-13f**: $^1$H NMR (400 MHz, $CDCl_3$): δ = 7.94 (d, *J* = 3.3 Hz, Ar*H*, 1H), 7.82 (d, *J* = 15.9 Hz, ArC*H*=CH, 1H), 7.45 (d, *J* =3.3 Hz, Ar*H*, 1H), 6.77 (d, *J* = 15.9 Hz, CH=C*H*$CO_2$Et, 1H), 4.31 (q, *J* = 7.3 Hz, O$CH_2$$CH_3$, 2H), 1.36 ppm (t, *J* = 7.3 Hz, $CH_2$$CH_3$, 3H); $^{13}$C NMR (100 MHz, $CDCl_3$): δ = 165.3, 162.9, 144.2, 135.2, 122.5, 120.8, 60.3, 13.7 ppm; MS (CI): m/z (%): 224 (17), $[M+C_3H_5]^+$, 212 (25) $[M+C_2H_5]^+$, 184 (100) $[M+H]^+$, 156 (42), 138 (59) [M-OEt]; $t_R$ (GC) = 11.83 minutes. **Data for *Z*-13f**: $^1$H NMR (400 MHz, $CDCl_3$): δ = 7.97 (d, *J* = 3.3 Hz, Ar*H*, 1H), 7.57 (dd, *J* = 3.3 Hz, $^5J$ = 1.0 Hz, Ar*H*, 1H), 7.37 (dd, *J* = 12.7 Hz, $^5J$ = 1.0 Hz, ArC*H*=CH, 1H), 6.15 (d, *J* = 12.7 Hz, CH=C*H*$CO_2$Et, 1H), 4.32 (q, *J* = 7.2 Hz, O$CH_2H_3$, 2H), 1.37 ppm (t, *J* = 7.2 Hz, $CH_2H_3$, 3H); $^{13}$C NMR (100 MHz, $CDCl_3$): δ = 165.3, 160.2, 142.6, 136.4, 123.7, 118.9, 60.2, 13.6 ppm; MS (CI): m/z (%):224 (11), $[M+C_3H_5]^+$, 212 (18) $[M+C_2H_5]^+$, 184 (100) $[M+H]^+$, 156 (20), 138 (97) [M-OEt]; 112 (14); $t_R$ (GC) = 11.59 minutes.

**Preparation of ethyl 3-(2'-furyl-3-methyl)-prop-2*E*-enoate (13h)**

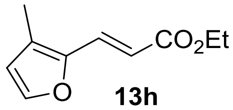

**13h**

Diisobutylaluminium hydride (50 mL of a 0.9 M solution in cyclohexane, 45 mmol)) was added dropwise to a solution of methyl 3-methyl-2-furoate (2.04 g, 14.6 mmol) in anhydrous toluene (120 mL) under nitrogen at -78 °C for 5 minutes. The reaction mixture

was allowed to warm to room temperature and stirred for a further 8 hours, cooled to -78 °C and quenched with aqueous potassium sodium tartrate tetrahydrate (sat. Rochelle salt, 40 mL) and EtOAc (50 mL). The resulting organic layer was separated and collected. The aqueous layer was extracted further with EtOAc (50 mL x 6) and the separated extracts combined with the original organic layer, dried ($MgSO_4$) and concentrated under reduced pressure to afford (3-methylfuran-2-yl)methanol as a yellow oil (1.33 g 81%). $^1$H NMR confirmed full conversion to desired alcohol which was used without further purification. *Bis*(acetoxy)iodobenzene (BAIB, 4.18 g, 13 mmol) was added to a solution of (3-methylfuran-2-yl)methanol (1.33 g, 11.8 mmol), TEMPO (184 mg, 1.18 mmol), in anhydrous DCM (90 mL) and the reaction mixture was stirred at room temperature under nitrogen for 4 hours. The $^1$H NMR spectrum of a reaction aliquot showed complete conversion to corresponding aldehyde **12h**. (Carbethoxmethylene)triphenylphosphorane (5.2 g, 15 mmol) was then added and the reaction mixture stirred for 17 hours. $^1$H NMR of a reaction aliquot showed complete consumption of aldehyde. The resulting orange solution was concentrated under reduced pressure and column chromatography on silica gel (2:25 $Et_2O$ in hexane) afforded alkenoate **13h** as a yellow oil (1.26 g, 45%). $R_f$ = 0.44 (1:10 $Et_2O$ in hexane); $\bar{\nu}$/(film) = 2976, 1703, 1632, 1299, 1253, 1164 cm$^{-1}$; $^1$H NMR (400 MHz, $CDCl_3$): δ = 7.50 (d, *J* = 15.5 Hz, Ar*CH*=CH, 1H), 7.40 (dq, *J* = 1.6 Hz, $^4J$ = 0.5 Hz, Ar*H*, 1H), 6.34 (d, *J* = 1.6 Hz, Ar*H*, 1H), 6.26 (d, *J* = 15.5 Hz, CH=C*H*$CO_2$Et, 1H), 4.27 (q, *J* = 7.1 Hz, OC$H_2$$H_3$, 2H), 2.18 (s, ArC$H_3$, 3H), 1.35 ppm (t, *J* = 7.1 Hz, $CH_2CH_3$, 3H); $^{13}$C NMR (100 MHz, $CDCl_3$): δ = 166.9, 146.6, 143.4, 128.5, 125.1, 114.1, 113.5, 59.7, 13.8, 9.7 ppm; MS (CI): m/z (%): 209 (18), $[M+C_2H_5]^+$, 181 (100), $[M+H]^+$, 153 (12), 135 (72), [M-OEt]; HRMS (NSI): calcd. for $C_{10}H_{13}O_3$, 181.0859 $[M+H]^+$ found 184.0857; $t_R$ (GC) = 11.43 minutes.

**General Procedure B:** Difluorocyclopropanation of Alkenoates **13** with MDFA

**Preparation of ethyl (1S*,3S*)-2,2-difluoro-3-(fur-2-yl)cyclopropane-1-carboxylate (15a)**

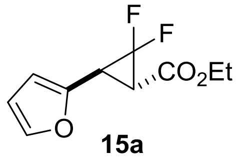

**15a**

An oven dried two-necked round bottom flask containing potassium iodide (1.87 g, 11.2 mmol) was sealed with a SubaSeal, and the salt was stirred and lightly flame dried under an atmosphere of argon. A low boiling point water condenser with a gas outlet connected to an argon/vacuum manifold was fitted to the reaction flask and the atmosphere was purged three times. Alkenoate **13a** (1.48 g, 8.9 mmol) followed by diglyme (1.43 mL) were added and the yellow suspension purged with argon/vacuum once and then heated to 120 °C. Once the reaction temperature had been reached, TMSCl (2.9 mL, 22.3 mmol) and MDFA (2.9 mL, 22.3 mmol) were added dropwise in that order. After 4 hours, the black reaction mixture was allowed to cool to room temperature and the reaction mixture was quenched with aqueous $NH_4Cl$ (10 mL) and diethyl ether (10 mL) was added. The organic layer was separated and the aqueous layer was extracted with diethyl ether (3 x 10 mL). The original organic layer and the extracts were combined, dried ($MgSO_4$) and concentrated under reduced pressure to remove volatile materials. The $^1$H NMR spectrum of the resulting brown oil confirmed 66% conversion to difluorocyclopropyl **15a**. Column chromoatography on silica gel (1:20 $Et_2O$ in hexane) afforded difluorocyclopropyl **15a** as a yellow oil (760 mg, 40%) and recovered starting alkenoate **13a** (340 mg, 23%). $R_f$ = 0.4 (1:19 $Et_2O$ in hexane); $^1$H NMR (400 MHz, $CDCl_3$): δ = 7.39 (dd, $J$ = 2.0 Hz, $^4J$ = 0.8 Hz, Ar*H*, 1H), 6.38 (dd, $J$ = 3.4, 2.0 Hz, Ar*H*, 1H), 6.29 (dd, $J$ = 3.4 Hz, $^4J$ = 0.8 Hz, Ar*H*, 1H), 4.28 (q, $J$ = 7.1 Hz, OC*H₂*CH₃, 2H), 3.48 (ddd, $J_{H-F}$ = 12.3 Hz, $J$ = 7.7 Hz, $^4J_{H-F}$ = 1.8 Hz, ArC*H*$CF_2$, 1H), 2.79 (dd, $J_{H-F}$ = 13.4 Hz, $J$ = 7.7 Hz, $CF_2$C*H*$CO_2$Et, 1H), 1.34 ppm (t, $J$ = 7.2 Hz, $OCH_2CH_3$, 3H); $^{13}$C NMR (100 MHz, $CDCl_3$): δ = 165.5, 145.1, 142.6, 110.6, 108.8 (dd, $^1J_{C-F}$ = 294.6, 289.7 Hz) 108.4, 61.9, 32.1 (t, $^2J_{C-F}$ = 10.9 Hz), 26.7 (dd, $^2J_{C-F}$ = 13.3, 8.9 Hz) 14.1 ppm; $^{19}$F NMR (376 MHz, $CDCl_3$): δ = -133.1 (dd, $^2J$ = 151.7 Hz, $J_{F-H}$ = 13.4 Hz, 1F), -135.1 ppm (dd, $^2J$ = 152.8 Hz, $J_{F-H}$ = 12.2 Hz, 1F); $\tilde{\nu}$/(film) = 2986, 2932, 2359, 2342, 1736, 1447, 1331, 1290, 1153, 1009 cm$^{-1}$; MS (CI): *m/z* (%): 257 (4) $[M+C_3H_5]^+$, 245 (9) $[M+C_2H_5]^+$, 217 (45) $[M+H]^+$, 197 (100) $[M-F]^+$, 189 (58), 171 (42) [M-

$OEt]^+$, 169 (70) $[(M+H)-F-Et]^+$, 143 (20), 125 (16); $t_R$ (GC) = 9.49 minutes; HRMS (APCI): calcd for $C_{10}H_{11}F_2O_3$, 217.0668 $[M+H]^+$ found 217.0671.

**Preparation of ethyl (1S*,3S*)-2,2-difluoro-3-phenylcyclopropane-1-carboxylate (15b)**

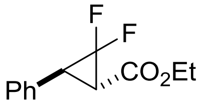

**15b**

The preparation of ester **15b** was attempted from alkenoate **13b** (0.67 mL, 4.03 mmol), MDFA (2.6 mL, 10 mmol), TMSCl (2.6 mL, 10 mmol), potassium iodide (1.87 g, 11.2 mmol) and diglyme (1.4 mL) according to General Procedure B with a total reaction time of 24 hours. The resulting $^1$H NMR of the crude reaction mixture after work up confirmed incomplete conversion of **13b** (28% conv. to **15b**). The reaction was repeated using commercial ethyl cinnamate (**13b**) according to general procedure B on twice the scale. The resulting $^1$H spectrum of the resulting brown oil confirmed incomplete conversion of **13b** (29% conv. to **15b**).

**Preparation of ethyl (1S*,3R*)-2,2-difluoro-3-(thiophen-2-yl)cyclopropane-1-carboxylate (15c)**

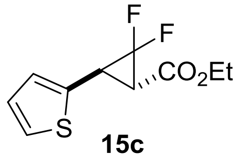

**15c**

Ester **15c** was prepared from alkenoate **13c** (1.45 mg, 8 mmol), MDFA (2.6 mL, 20 mmol), TMSCl (2.6 mL, 20 mmol), potassium iodide (3.68 g, 22.6 mmol) and diglyme (1.3 mL) according to general procedure B with a reaction time of 4 hours. $^1$H NMR of the resulting crude product showed 77% conversion to desired ester **15c**. Column chromatography on silca gel (1:19 $Et_2O$ in hexane) afforded ester **15c** as a yellow oil (1.32 g, 71%) and recovered alkenoate **13c** (310 mg, 21%). $R_f$ = 0.42 (1:24 $Et_2O$ in hexane); $^1$H NMR (500 MHz, $CDCl_3$): δ = 7.26 (dd, $J$ = 3.3, 3.1 Hz, Ar$H$, 1H), 7.00 (d, $J$ = 3.3 Hz, Ar$H$, 1H), 7.00 (d, $J$ = 3.1 Hz, Ar$H$, 1H), 4.27 (q, $J$ = 7.2 Hz, $OCH_2CH_3$, 2H), 3.63 (ddd, $J_{H-F}$ = 13.1, 2.3 Hz, $J$ = 7.7 Hz, ArC$H$, 1H), 2.67 (dd, $J_{H-F}$ = 13.6, $J$ = 7.7 Hz, C$H$$CO_2$Et, 1H), 1.33 ppm (t, $J$ = 7.2 Hz, $OCH_2CH_3$, 3H); $^{13}$C NMR (100 MHz, $CDCl_3$): δ = 165.7, 133.3, 127.0, 126.7, 125.4, 110.4 (dd, $^1J_{C-F}$ = 296.8, 290.3 Hz), 61.9,

34.5 ($^2J_{C-F}$ = 10.9 Hz), 28.6 (dd, $^2J_{C-F}$ = 13.0, 9.2 Hz), 14.1 ppm; $^{19}$F NMR (376 MHz, $CDCl_3$): δ = -132.8 (ddd, $^2J_{F-F}$ = 151.7 Hz, $J_{F-H}$ = 13.6, 2.4 Hz, 1F), -134.2 ppm (dd, $^2J_{F-F}$ = 151.2 Hz, $J_{F-H}$ = 13.3 Hz, 1F); $\bar{\nu}$/(film) = 2965, 2359, 2342, 1732, 1466, 1431, 1325, 1285, 1215, 1152, 1009 $cm^{-1}$; MS (CI): *m/z* (%): 261 (7) $[M+C_2H_5]^+$, 233 (33) $[M+H]^+$, 213 (100) $[M-F]^+$, 205 (43), 187 (36), 185 (66) $[M-(F+Et)]^+$, 159 (16), 139 (18); HRMS (APCI): calcd for $C_{10}H_{14}F_2O_2S_1N_1$, 250.0708 $[M+NH_4]^+$ found 250.0706; $t_R$ (GC) = 11.15 minutes.

**Preparation of ethyl (1S*,3S*)-3-(benzo[d][1,3]dioxol-5-yl)-2,2-difluorocyclopropane-1-carboxylate (15d)**

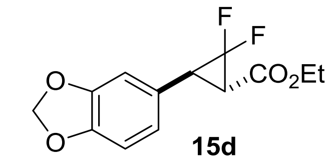

**15d**

Ester **15d** was prepared from alkenoate **13d** (1.66 g, 7.5 mmol), MDFA (2.4 mL, 18.5 mmol), TMSCl (2.4 mL, 18.5 mmol), potassium iodide (3.45 g, 20.7 mmol) and diglyme (1.2 mL) according to general procedure B. $^1$H NMR of the resulting crude product showed 50% conversion to desired ester **15d**. Column chromatography on silca gel (1:9 $Et_2O$ in hexane) afforded ester **15d** as a yellow oil (880 mg, 43%) and recovered alkenoate **13d** (700 mg, 42%). $R_f$ = 0.59 (1:4 $Et_2O$ in hexane); $^1$H NMR (400 MHz, $CDCl_3$): δ = 6.82-6.73 (m, Ar*H*, 3H), 6.00 (s, OC*H₂*O, 2H), 4.28 (q, *J* = 7.2 Hz, $OCH_2CH_3$, 2H), 3.44 (ddd, $J_{H-F}$ = 13.2, 2.9 Hz, *J* = 7.9 Hz, ArC*H*, 1H), 2.65 (dd, $J_{H-F}$ = 13.2 Hz, *J* = 7.9 Hz, C*H*$CO_2$Et, 1H), 1.34 (t, *J* = 7.2 Hz, $OCH_2CH_3$, 3H); $^{13}$C NMR (100 MHz, $CDCl_3$): δ = 165.8, 147.5, 146.9, 124.1, 121.3, 110.2 (t, $^1J_{C-F}$ = 294.2, 287.2 Hz), 107.9 (represents 2 carbons), 100.8, 61.3, 32.4 (dd, $^2J_{C-F}$ = 11.2, 8.8 Hz), 32.0 (t, $^2J_{C-F}$ = 11.1 Hz) 13.6 ppm; $^{19}$F NMR (376 MHz, $CDCl_3$): δ = -133.1 (dd, $^2J_{F-F}$ = 151.9 Hz, $J_{F-H}$ = 13.6 Hz, 1F), -134.1 (ddd, $^2J_{F-F}$ = 151.3 Hz, $J_{F-H}$ = 13.5, 2.7 Hz, 1F), $\bar{\nu}$/(film) = 2359, 2342, 1732, 1506, 1466, 1449, 1290, 1242, 1213,1152, 1038, 1013, 989 $cm^{-1}$; MS (CI): *m/z* (%): 299 (19) $[M+C_2H_5]^+$, 271 (44) $[M+H]^+$, 251 (94) $[M-F]^+$, 223 (75) $[(M+H)-F-OEt]^+$, 206 (24), 197 (26) $[M-CO_2Et]^+$, 177 (100); HRMS (APCI): calcd for $C_{13}H_{13}F_2O_4$, 271.0776 $[M+H]^+$ found 271.0776; $t_R$ (GC) = 13.50 minutes.

**Preparation of ethyl (1S*,3S*)-2,2-difluoro-3-(pyridin-2-yl)cyclopropane-1-carboxylate (15e)**

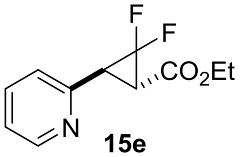

**15e**

The preparation of ester **15e** was attempted from alkenoate **13e** (400 mg, 2.3 mmol), MDFA (0.74 mL, 5.7 mmol), TMSCl (0.74 mL, 5.7 mmol), potassium iodide (1.1 g, 6.6 mmol) and diglyme (0.45 mL) according to general procedure B with a total reaction time of 4 hours. The resulting $^1$H NMR of the resulting crude black solid showed diglyme and diethyl ether with trace amounts of alkenoate **13e**. The desired product **15e** was not observed (0% conversion).

**Preparation of ethyl (1S*,3S*)-2,2-difluoro-3-(2'-pyrrolyl-1-carboxylic acid *tert*-butyl ester)cyclopropane-1-carboxylate (15f)**

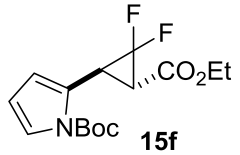

**15f**

Ester **15f** was prepared from alkenoate **13f** (2.12 g, 8 mmol), MDFA (2.6 mL, 20 mmol), TMSCl (2.6 mL, 20 mmol), potassium iodide (3.74 g, 22.4 mmol) and diglyme (1.6 mL) according to general procedure B. $^1$H NMR of the resulting crude product showed 92% conversion to desired product. Column chromatography on silica gel (1:20 $Et_2O$ in hexane) afforded ester **15f** as a yellow oil (1.37 g, 54%) and recovered alkenoate **13f** (140 mg, 7%). $R_f$ = 0.35 (1:4 $Et_2O$ in hexane); $^1$H NMR (400 MHz, $CDCl_3$): δ = 7.29 (ddd, $J$ = 3.3 Hz, $^4J$ = 1.7 Hz, $^5J$ = 0.7 Hz, Ar$H$, 1H), 6.17-6.15 (br. m, Ar$H$, 1H), 6.14 (t, $J$ = 3.4 Hz, Ar$H$, 1H), 4.28 (ABq, $J$ = 7.4 Hz, $^2J$ = 3.1 Hz, $OCH_aH_bCH_3$, 2H), 3.73 (br. dd, $J_{H\text{-}F}$ = 12.8 Hz, $J$ = 7.8 Hz, ArC$H$, 1H), 2.63 (dd, $J$ = 13.4 Hz, $J$ = 7.8 Hz, $HCCO_2Et$, 1H), 1.63 (s, $C(CH_3)_3$, 9H), 1.34 (t, $J$ = 7.2 Hz, $CH_2CH_3$, 3H); $^{13}$C NMR (100 MHz, $CDCl_3$): δ = 165.7, 148.4, 124.0, 122.1, 112.9, 110.3 (dd, $^1J_{C\text{-}F}$ = 297.3, 287.2 Hz), 109.6, 84.1, 61.1, 31.7 (t, $^2J_{C\text{-}F}$ = 10.9 Hz), 27.6 (t, $^2J_{C\text{-}F}$ = 12.9, 8.0 Hz), 27.5, 13.7 ppm; $^{19}$F NMR (376 MHz, $CDCl_3$): δ = -133.4 (dd, $^2J$ = 149.8 Hz, $J_{F\text{-}H}$ = 12.8 Hz, $CF_aF_b$, 1F), -134.9 ppm (dd, $^2J$ = 149.8 Hz, $J_{F\text{-}H}$ = 13.4 Hz, $CF_aF_b$, 1F); $\bar{\nu}$/(film) = 2980, 1742, 1459, 1325, 1158, 1128

cm$^{-1}$; MS (CI): $m/z$ (%): 224 (17) [M-(F+$CO_2$Et)+H]$^+$, 196 (100) [M-(F+Boc)+H]$^+$, 168 (29), 150 (72) [M-(*N*-Boc-pyrrolyl)+H]$^+$; HRMS (APCI): calcd for $C_{15}H_{23}F_2N_2O_4$, 333.1620 [M+$NH_4$]$^+$ found 333.1623; $t_R$ (GC) = 12.07 minutes.

**Preparation of ethyl (1S*,3S*)-2,2-difluoro-3-(2'-thiazolyl)cyclopropane-1-carboxylate (15g)**

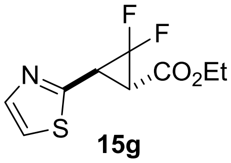

**15g**

The preparation of ester **15g** was attempted from alkenoate **13g** (1.18 g, 6.4 mmol), MDFA (2.1 mL, 16.2 mmol), TMSCl (2.1 mL, 16.2 mmol), potassium iodide (3.0 g, 18 mmol) and diglyme (1.28 mL) according to general procedure B with a total reaction time of 4 hours. The resulting $^1$H NMR of the resulting crude black solid showed diglyme and diethyl ether the desired product **15g** was not observed (0% conversion).

**Preparation of ethyl (1S*,3S*)-2,2-difluoro-3-(2'-furyl-3-methyl) cyclopropane-1-carboxylate (15h)**

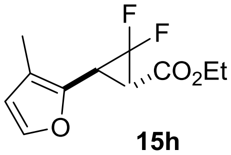

**15h**

Ester **15h** was prepared from alkenoate **13h** (721 mg, 4.0 mmol), MDFA (1.3 mL, 10 mmol), TMSCl (1.3 mL, 10 mmol), potassium iodide (1.84 g, 12 mmol) and diglyme (0.7 mL) according to general procedure B. $^1$H NMR of the resulting crude product showed 80% conversion to desired product. Column chromatography on silica gel (1:20 $Et_2O$ in hexane) afforded ester **15h** as a yellow oil (417 mg, 45%) and recovered alkenoate **13h** (136 mg, 19%). $R_f$ = 0.56 (1:4 $Et_2O$ in hexane); $^1$H NMR (400 MHz, $CDCl_3$): δ = 7.26 (d, $J$ = 1.8 Hz, Ar*H*, 1H), 6.23 (d, $J$ = 1.8 Hz, Ar*H*, 1H), 4.26 (q, $J$ = 7.1 Hz, O$CH_2$$CH_3$, 2H), 3.67 (ddd, $J_{H\text{-}F}$ = 12.2, 2.3 Hz, $J$ = 7.7 Hz, ArC*H*, 1H), 2.92 (dd, $J_{H\text{-}F}$ = 13.5 Hz, $J$ = 7.7 Hz, *H*C$CO_2$Et, 1H), 2.05 (s, Ar$CH_3$, 3H), 1.33 ppm (t, $J$ = 7.1 Hz, $CH_2CH_3$, 3H); $^{13}$C NMR (100 MHz, $CDCl_3$): δ = 165.9, 141.4, 139.8, 119.0, 113.4, 110.1 (t, $^1J_{C\text{-}F}$ = 294.1, 287.4 Hz), 61.9, 30.8 (t, $J_{C\text{-}F}$ = 11.0 Hz), 25.5 (dd, $J_{C\text{-}F}$ = 13.5, 9.5 Hz), 14.1, 9.6 ppm; $^{19}$F NMR (376 MHz, $CDCl_3$): δ = -132.8 (ddd, $^2J$ = 150.5 Hz, $J_{F\text{-}H}$ =

13.5, 2.3 Hz, C$F_a$$F_b$, 1F), -131.5 ppm (dd, $^2J$ = 150.5 Hz, $J_{F\text{-}H}$ = 12.2 Hz, C$F_a$$F_b$, 1F); $\bar{\nu}$/(film) = 2985, 1738, 1454, 1329, 1290, 1208, 1154, 1091, 1013, 989 cm$^{-1}$; MS (CI): *m/z* (%): 259 (6) $[M+C_2H_5]^+$, 231 (25) $[M+H]^+$, 211 (100) $[M-F]^+$, 203 (23), 185 (25) $[M-OEt]^+$, 183 (28); HRMS (APCI): calcd for $C_{11}H_{11}F_2O_3$, 229.0671 $[M-H]^+$ found 229.0676; $t_R$ (GC) = 10.61 minutes.

**General Procedure C:** Reduction of Difluorocyclopropyl Ethyl Esters

**Preparation of ethyl ((1S*,3S*)-2,2-difluoro-3-(furan-2-yl)cyclopropyl)methanol (17a)**

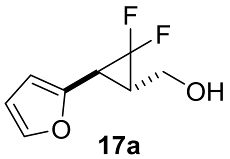


**17a**

Diisobutylaluminium hydride (24 ml of a 1.1 M solution in cyclohexane, 23.7 mmol) was added dropwise to a solution of ester **15a** (1.71 g, 7.9 mmol) in anhydrous toluene (70 mL) under nitrogen at -78 °C over 10 minutes. The reaction mixture was allowed to warm to room temperature and stirred for a further 8 hours, then cooled to 0 °C and quenched by dropwise addition of $H_2O$ (2 mL), 0.1 M aqueous NaOH (2 mL) and $H_2O$ (2 mL) in that order. $MgSO_4$ was added to the quenched mixture until the solid was free flowing and the mixture was left overnight at room temperature (14-17 h). The resulting white emulsion was washed with EtOAc (4 x 50 mL) and the organic extracts were combined and concentrated under reduced pressure to afford alcohol **17a** as a pale yellow oil (1.3 g, 94%). $R_f$ = 0.19 (1:2 $Et_2O$ in hexane); $^1$H NMR (400 MHz, $CDCl_3$): δ = 7.37 (dd, *J* = 1.9 Hz, $^4J$ = 0.8 Hz, Ar*H*, 1H), 6.38 (dd, *J* = 3.3, 1.9 Hz, Ar*H*, 1H), 6.21 (br. d, *J* = 3.3 Hz, Ar*H*, 1H), 3.99-3.91 (br. m, C$H_a$$H_b$OH, 1H), 3.90-3.83 (br. m, C$H_a$$H_b$OH, 1H), 2.64 (dd, $J_{H\text{-}F}$ = 13.1 Hz, *J* = 7.3 Hz, ArC*H*, 1H), 2.32-2.23 (m, C*H*$CH_2$O, 1H), 1.62 ppm (br. s, $CH_2$O*H*, 1H); $^{13}$C NMR (100 MHz, $CDCl_3$): δ = 146.6, 141.6, 112.1 (t, $^1J_{C\text{-}F}$ = 289.0 Hz), 110.1, 106.9, 58.6 (d, $J_{C\text{-}F}$ = 5.9 Hz), 30.4 (t, $^2J_{C\text{-}F}$ = 9.5 Hz), 24.2 ppm (dd, $^2J_{C\text{-}F}$ = 13.6, 10.6 Hz); $^{19}$F NMR (376 MHz, $CDCl_3$): δ = -135.5 (ddd, $^2J$ = 158.7 Hz, $J_{F\text{-}H}$ = 13.6, 2.0 Hz, 1F), -138.3 ppm (dd, $^2J$ = 158.8 Hz, $J_{F\text{-}H}$ = 13.1 Hz, 1F); $\bar{\nu}$/(film) = 2926, 2359, 2342, 1458, 1263, 1167, 1009, 736 cm$^{-1}$; MS (CI): *m/z* (%): 175 (13) $[M+H]^+$, 157 (100) $[M-OH]^+$, 139 (13), 127 (12), 109 (18); HRMS (TOF): calcd for $C_8H_8O_2F_2$, 174.0492 [M] found 174.0496; $t_R$ (GC) = 9.39 minutes.

**Preparation of ((1S*,3R*)-2,2-difluoro-3-(thiophen-2-yl)cyclopropyl)methanol (17c)**

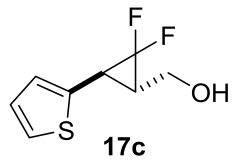

**17c**

Alcohol **17c** was prepared from ester **15c** (272 mg, 0.85 mmol), DIBAL (2.3ml of a 1.1 M solution in cyclohexane, 2.55 mmol) and DCM (6 mL) according to general procedure B. $^1$H NMR of the result crude reaction mixture confirmed 83% conversion. Column chromatography on silica gel (2:3 $Et_2O$ in hexane) afforded alcohol **17c** as a yellow oil (81 mg, 50%). $R_f$ = 0.19 (1:2 $Et_2O$ in hexane); $^1$H NMR (400 MHz, $CDCl_3$): δ = 7.23 (dd, $J$ = 5.2, $^4J$ = 1.3 Hz, Ar*H*, 1H), 6.99 (dd, $J$ = 5.2, 3.6 Hz, Ar*H*, 1H), 6.95 (br. d, $J$ = 3.6 Hz, Ar*H*, 1H), 3.94 (dddd, $^2J$ = 12.1 Hz, $J$ = 6.6, 1.1 Hz, $^4J_{H\text{-}F}$ = 2.7 Hz, C$H_a$H$_b$OH, 1H), 3.84 (ddd, $^2J$ = 12.1 Hz, $J$ = 7.8, 1.6 Hz, CH$_a$$H_b$OH, 1H), 2.77 (dd, $J_{H\text{-}F}$ = 13.6 Hz, $J$ = 7.3 Hz, ArC*H*, 1H), 2.33 (br. s, $CH_2OH$, 1H), 2.17 ppm (ddddd, $J_{H\text{-}F}$ = 13.4 Hz, $J$ = 7.8, 7.4, 6.6 Hz, $^4J$ = 0.9 Hz, C*H*$CH_2OH$, 1H); $^{13}$C NMR (100 MHz, $CDCl_3$): δ = 135.1, 126.5, 125.4, 124.2, 112.7 (t, $^1J_{C\text{-}F}$ = 290.1 Hz), 58.7 (d, $J_{C\text{-}F}$ = 5.7 Hz), 32.9 (t, $^2J_{C\text{-}F}$ = 9.6 Hz), 26.1 ppm (dd, $^2J_{C\text{-}F}$ = 12.5, 11.1 Hz); $^{19}$F NMR (376 MHz, $CDCl_3$): δ = -135.4 (ddd, $^2J$ = 158.0 Hz, $J_{F\text{-}H}$ = 13.5 Hz, $^4J_{F\text{-}H}$ = 2.7 Hz, 1F), -137.5 ppm (dd, $^2J$ = 158.0 Hz, $J_{F\text{-}H}$ = 13.5 Hz, 1F); $\bar{\nu}$/(film) = 3324, 2930, 2885, 1472, 1435, 1245, 1080, 1037, 1007 $cm^{-1}$; MS (CI): $m/z$ (%): 219 (6) $[M+C_2H_5]^+$, 191 (26) $[M+H]^+$, 173 (100) $[M-OH]^+$, 153 (42) $[M-F_2]^+$, 123 (22); HRMS (ASAP): calcd for $C_8H_7F_2S_1$, 173.0231 $[M-H_2O+H]^+$ found 173.0229; $t_R$ (GC) = 10.51 minutes.

**Preparation of ((1S*,3R*)-2,2-difluoro-3-( benzo[d][1,3]dioxol-5-yl)cyclopropyl)methanol (17d)**

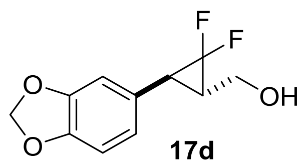

**17d**

Alcohol **17d** was prepared from ester **15d** (550 mg, 2 mmol), DIBAL (1.0 M in cyclohexane, 6.0 mL, 6 mmol) and toluene (30 mL) according to general procedure C. Column chromatography on silica gel (1:1 $Et_2O$ in hexane) afforded desired alcohol **17d** as a colourless oil (342 mg, 75%). $R_f$ = 0.35 (1:1 $Et_2O$ in hexane); $^1$H NMR (400 MHz, $CDCl_3$): δ =

6.80-6.72 (m, Ar*H*, 3H), 5.97 (s, OC*H₂*O, 2H), 3.95-3.83 (m, CHC*H₂*OH, 2H), 2.57 (ddd, $J_{H-F}$ = 11.2 Hz, *J* = 7.6 Hz, $^4J$ = 3.3 Hz, ArC*H,* 1H), 2.12 (ddddd, $J_{H-F}$ = 11.2 Hz, *J* = 7.6, 7.5, 7.1 Hz, $^4J$ = 3.3 Hz, C*H*$CH_2OH$, 1H), 1.92 ppm (br. s, $CH_2OH$, 1H); $^{13}C$ NMR (100 MHz, $CDCl_3$): δ = 147.3, 146.4, 126.1, 121.1, 113.1 (t, $^1J_{C-F}$ = 289.1 Hz), 108.0, 107.8, 100.6, 59.1 (d, $J_{C-F}$ = 4.3 Hz), 30.8 (t, $^2J_{C-F}$ = 9.1 Hz), 30.7 ppm (t, $^2J_{C-F}$ = 11.1 Hz); $^{19}F$ NMR (376 MHz, $CDCl_3$): δ = -136.5 (ddd, $^2J$ = 158.2 Hz, $J_{F-H}$ = 10.8 Hz, $^4J_{F-H}$ = 4.2 Hz, CHC$F_a$$F_b$CH$CH_2$, 1F), -136.6 ppm (ddt, $^2J$ = 157.8 Hz, $J_{F-H}$ = 10.8 Hz, $^4J_{F-H}$ = 3.7 Hz, CH$CF_a$$F_b$CH$CH_2$, 1F). Reported chemical shifts are representative of an AB system; $\bar{\nu}$/(film) = 3292, 1495, 1476, 1437, 1236, 1167, 1036, 1011, 930 $cm^{-1}$; MS (CI): *m/z* (%): 269 (2) $[M+C_3H_5]^+$, 257 (10) $[M+C_2H_5]^+$, 229 (32) $[M+H]^+$, 211 (100) $[M-OH]^+$, 191 (90) $[(M+H)-F_2]^+$, 181 (48), 161 (74), 131 (26); HRMS (APCI): calcd for $C_{11}H_{11}F_2O_3$, 229.0671 $[M+H]^+$ found 229.0668; $t_R$ (GC) = 12.96 minutes.

**Preparation of ((1S*,3R*)-2,2-difluoro-3-(2'-pyrrolyl-1-carboxylic acid *tert*-butyl ester)methanol (17f)**

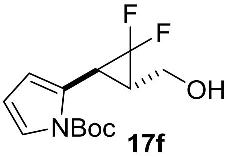

**17f**

Alcohol **17f** was prepared from ester **15f** (600 mg, 1.9 mmol), DIBAL (1.0 M in cyclohexane, 5.7 mL, 5.7 mmol) and toluene (40 mL) according to general procedure C. Column chromatography on silica gel (1:1 $Et_2O$ in hexane) afforded desired alcohol **17f** as a pale yellow oil (30 mg, 6%). $R_f$ = 0.32 (1:1 $Et_2O$ in hexane); $^1H$ NMR (400 MHz, $CDCl_3$): δ = 7.23 (ddd, *J* = 3.4 Hz, $^4J$ = 1.8 Hz, $^5J$ = 0.8 Hz, Ar*H*, 1H), 6.25 (m, Ar*H*, 1H), 6.14 (t, *J* = 3.4 Hz, Ar*H*, 1H), 3.98 (ddd, $^2J$ = 12.0 Hz, *J* = 5.7, 1.8 Hz, CHC$H_a$$H_b$OH, 1H), 3.74 (dt, $^2J$ = 11.9 Hz, *J* = 1.8 Hz, CHC$H_a$$H_b$OH, 1H), 2.96 (br. s, O*H*, 1H), 2.86 (dd, $^3J_{H-F}$ = 15.3 Hz, *J* = 7.3 Hz, ArC*H*, 1H), 1.99 (m, C*H*$CH_2OH$, 1H), 1.63 ppm (s, $OC(CH_3)_3$, 9H); $^{13}C$ NMR (100 MHz, $CDCl_3$): δ = 149.3, 126.5, 121.2, 113.3 (dd, $^1J_{C-F}$ = 290.3, 287.3 Hz), 112.3 (d, $^4J_{C-F}$ = 3,3 Hz), 110.0, 84.2, 59.0 (d, $J_{C-F}$ = 4.4 Hz), 32.9 (t, $^2J_{C-F}$ = 9.3 Hz), 23.5, 23.3 ppm (dd, $^2J_{C-F}$ = 13.0, 9.6 Hz); $^{19}F$ NMR (376 MHz, $CDCl_3$): δ = -132.6 (dd, $^2J$ = 160.0 Hz, $J_{F-H}$ = 13.6 Hz, C$F_a$$F_b$, 1F), -134.7 ppm (dd, $^2J$ = 159.0 Hz, $J_{F-H}$ = 15.1 Hz, $CF_a$$F_b$, 1F); $\bar{\nu}$/(film) = 3443, 2982, 2835, 1740, 1478, 1405, 1372, 1323, 1260, 1164, 1128 $cm^{-1}$; MS (CI): *m/z* (%): 180 (23), 152 (100); $t_R$ (GC) = 10.12 minutes. Due to lack of m/z consistent with product accurate mass analysis was not attempted.

**Preparation of ((1S*,3R*)-2,2-difluoro-3-(2'-furyl-3-methyl)cyclopropyl)methanol (17h)**

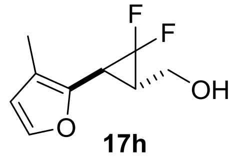

**17h**

Alcohol **17h** was prepared from ester **15h** (234 mg, 1.0 mmol), DIBAL (1.0 M in cyclohexane, 3.0 mL, 3.0 mmol) and toluene (6.2 mL) according to general procedure C to afford desired alcohol **17h** as a pale yellow oil (160 mg, 84%). $R_f$ = 0.21 (1:1 $Et_2O$ in hexane); $^1$H NMR (400 MHz, $CDCl_3$): δ = 7.25 (d, $J$ = 1.8 Hz, Ar$H$, 1H), 6.22 (d, $J$ = 1.8 Hz, Ar$H$, 1H), 3.97-3.84 (br. m., CHC$H_2$OH, 2H), 2.52 (dd, $J_{H\text{-}F}$ = 12.7, $J$ = 7.3 Hz, ArC$H$, 1H), 2.40 (ddddd, $J_{H\text{-}F}$ = 13.9, 2.0 Hz, $J$ = 7.6, 7.3, 6.4 Hz), 2.04 (s, ArC$H_3$, 3H), 1.62 ppm (br. s, O$H$, 1H); $^{13}$C NMR (100 MHz, $CDCl_3$): δ = 141.5, 141.0, 118.2, 113.3, 113.0 (t, $^1J_{C\text{-}F}$ = 289.0 Hz), 59.1 (d, $J_{C\text{-}F}$ = 5.6 Hz), 29.5 (t, $^2J_{C\text{-}F}$ = 9.5 Hz), 23.4 (dd, $^2J_{C\text{-}F}$ = 13.4, 10.8 Hz), 9.6 ppm; $^{19}$F NMR (376 MHz, $CDCl_3$): δ = -134.8 (ddd, $^2J$ = 157.3 Hz, $J_{F\text{-}H}$ = 13.9, 2.2 Hz, C$F_a$F$_b$, 1F), -138.4 ppm (dd, $^2J$ = 157.3 Hz, $J_{F\text{-}H}$ = 12.7 Hz, CF$_a$$F_b$, 1F); $\bar{\nu}$/(film) = 3333, 2928, 2887, 1632, 1455, 1271, 1180, 1041, 1013 cm$^{-1}$; MS (CI): $m/z$ (%): 189 (9) $[M+H]^+$, 171 (100) $[M\text{-}OH]^+$, 153 (16), 121 (14); HRMS (APCI): calcd for $C_9H_9F_2O_1$, 171.0616 $[M\text{-}H_2O+H]^+$ found 171.0615; $t_R$ (GC) = 9.99 minutes.

**Preparation of (1S*,3S*)-2,2-difluoro-3-phenylcyclopropane-1-carbaldehyde (18b)**

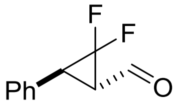

**18b**

BAIB (3.0 g, 9.3 mmol) was added to a solution of ((1S*,3S*)-2,2-difluoro-3-phenylcyclopropyl)methanol (**17b**) (1.58 g, 8.6 mmol) and TEMPO (133 mg, 0.9 mmol) in anhydrous DCM (45 mL) and the reaction mixture was stirred at room temperature under nitrogen for 6 hours. The $^1$H NMR spectrum showed complete conversion to aldehyde **18b**. Kugelrohr distillation (50 °C, 20 mbar) removed iodobenzene side product then (60 °C, 0.1 mbar) afforded aldehyde **18b** as a pale yellow oil (1.17 g, 75%). $R_f$ = 0.26 (1:4 $Et_2O$ in hexane); $^1$H NMR (400 MHz, $CDCl_3$): δ = 9.51 (dd, $J$ = 4.4 Hz, $^4J$ = 2.0 Hz, C(O)$H$, 1H), 7.42-7.34 (m, Ar$H$, 3H), 7.29-7.27 (m, Ar$H$, 2H), 3.63 (ddd, $J_{H\text{-}F}$ = 14.8 Hz, $J$ = 7.6 Hz, $^4J$ = 2.1 Hz, ArC$H$, 1H), 2.97 (ddd, $J_{H\text{-}F}$ = 12.8 Hz, $J$ = 7.5, 4.4 Hz, C$H$C(O)H, 1H); $^{13}$C NMR (100 MHz, $CDCl_3$): δ =

191.8 (d, $J_{C-F}$ = 3.9 Hz), 130.0, 128.4, 127.8, 127.6, 111.0 (t, $^2J_{C-F}$ = 291.3 Hz), 40.3 (t, $J_{C-F}$ = 40.4 Hz), 32.8 ppm (dd, $J_{C-F}$ 12.1, 8.8 Hz); $^{19}$F NMR (376 MHz, $CDCl_3$): δ = -129.4 (dd, $^2J$ = 158.0 Hz, $J_{F-H}$ = 15.0 Hz, 1F), -133.4 ppm (dd, $^2J$ = 157.9 Hz, $J_{F-H}$ = 12.9 Hz, 1F); $\bar{\nu}$/(film) = 1738, 1634, 1059, 980 $cm^{-1}$; MS (CI): *m/z* (%): 183 (11) $[M+H]^+$, 163 (73) $[M-F]^+$, 135 (100) [(M+H)-F-CO], 115 (24) [(M+H)-$F_2$-CO]; HRMS (APCI): calcd for $C_{10}H_9F_2O$, 183.0616 $[M+H]^+$ found 183.0615; $t_R$ (GC) = 10.03 minutes.

**General Procedure D:** Tandem Oxidation/Olefination of Difluorocyclopropyl Alcohols

**Preparation of (*E*)-3-((1R*,3S*)-2,2-difluoro-3-phenylcyclopropyl)-N-methoxy-N-methylacrylamide (19a) and (*Z*)-3-((1R*,3S*)-2,2-difluoro-3-phenylcyclopropyl)-N-methoxy-N-methylacrylamide (19b)**

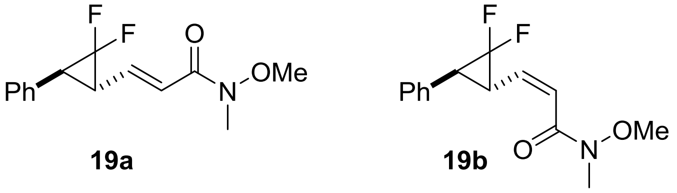

**19a** **19b**

*Bis*(acetoxy)iodobenzene (BAIB, 304 mg, 0.92 mmol) was added to a solution of alcohol **17b** (148 mg, 0.8 mmol) and TEMPO (12 mg, 0.08 mmol) in anhydrous DCM (3.2 mL) and the reaction mixture was stirred at room temperature under nitrogen for 6 hours. The $^1$H NMR spectrum showed complete conversion to the corresponding aldehyde. *N*-methoxy-*N*-methyl(triphenylphosphoranylidene)acetamide (378 mg, 1.04 mmol) was then added, and the reaction mixture stirred for 16 hours until the $^1$H or $^{19}$F NMR spectrum showed complete conversion. The resulting orange solution was concentrated under reduced pressure and column chromatography on silica gel (1:1 to 3:2 diethyl ether in hexane) afforded **19a** (157 mg, 74%) as a pale yellow solid and **19b** (25 mg, 9%) as a yellow oil. Data for **19a**: m.p. = 52-55 °C (chloroform/pentane); $R_f$ = 0.14 (1:1 $Et_2O$ in hexane); $^1$H NMR (400 MHz, $CDCl_3$): δ = 7.39-7.31 (m, Ar*H*, 3H), 7.30-7.25 (m, Ar*H*, 2H), 6.82 (dd, *J* = 15.6, 9.6 Hz, CHC*H*=CH, 1H), 6.69 (d, *J* = 15.4 Hz, CH=C*H*CO, 1H), 3.74 (s, OC*H₃*, 3H), 3.28 (s, NC*H₃*, 3H), 2.91 (dd, $J_{H-F}$ = 14.6 Hz, *J* = 7.1 Hz, ArC*H*$CF_2$CH, 1H), 2.70 ppm (ddd, $J_{H-F}$ = 13.1 Hz, *J* = 9.6, 7.2 Hz, C*H*CH, 1H); $^{13}$C NMR (100 MHz, $CDCl_3$): δ = 165.3, 138.5 (d, $^4J_{C-F}$ = 4.8 Hz), 132.1, 128.2, 127.4, 127.1, 120.6, 112.9 (t, $^1J_{C-F}$ = 292.7 Hz), 61.3, 35.6 (dd, $J_{C-F}$ = 11.2, 9.0 Hz), 33.8 (t, $J_{C-F}$ = 11.5), 31.9 ppm; $^{19}$F NMR (376 MHz, $CDCl_3$): δ = -130.5 (dd, $^2J$ = 156.1 Hz, $J_{F-H}$ = 14.1 Hz, 1F), -135.7 ppm

(dd, $^2J$ = 156.0 Hz, $J_{F-H}$ = 13.5 Hz, 1F); $\bar{\nu}$/(film) = 1661, 1630, 1383, 1273, 1152, 986 cm$^{-1}$; MS (CI): *m/z* (%): 288 (8) [(M-F)+$C_3H_5$]$^+$, 276 (9) [(M-F)+$C_2H_5$]$^+$, 248 (100) [M-F]$^+$; HRMS (APCI): calcd for $C_{14}H_{16}F_2NO_2$, 268.1144 [M+H]$^+$ found 268.1145; $t_R$ (GC) = 11.30 minutes. Data for **19b**: $R_f$ = 0.33 (1:1 $Et_2O$ in hexane); $^1$H NMR (400 MHz, $CDCl_3$): δ = 7.36-7.34 (m, Ar*H*, 4H), 7.32-7.27 (m, Ar*H*, 1H), 6.53 (d, *J* = 11.4 Hz, CH=C*H*CO, 1H), 5.95 (dddd, *J* = 11.4, 10.1 Hz, $^4J_{H-F}$ = 1.4, 1.3 Hz, CHC*H*=CH, 1H), 4.29 (br. ddd, $J_{H-F}$ = 14.0 Hz, *J* = 10.1, 7.2 Hz, CHCH=C*H*, 1H), 3.74 (s, OC*H*$_3$, 3H), 3.27 (s, NC*H*$_3$, 3H), 2.81 (dd, $J_{H-F}$ = 14.9 Hz, *J* = 7.2 Hz, ArC*H*CF$_2$CH, 1H); $^{13}$C NMR (100 MHz, $CDCl_3$): δ = 166.2 (d, $^5J_{C-F}$ = 3.7 Hz), 133.3 (d, $^4J_{C-F}$ = 6.0 Hz), 131.8, 128.1, 127.7, 126.9, 119.0, 113.7 (t, $^1J_{C-F}$ = 291.3 Hz), 61.3, 36.1 (dd, $^2J$ = 11.5, 8.9 Hz), 31.5, 29.8 ppm (dd, $^2J$ = 13.2, 9.8 Hz); $^{19}$F NMR (376 MHz, $CDCl_3$): δ = -132.6 (dd, $^2J$ = 153.9 Hz, $J_{F-H}$ = 14.8 Hz, 1F), -136.3 ppm (dd, $^2J$ = 153.8 Hz, $J_{F-H}$ = 14.0 Hz, 1F); $\bar{\nu}$/(film) = 2982, 2360, 2343, 1656, 1627, 1425, 1354, 1252, 1169, 1001 cm$^{-1}$; MS (CI): *m/z* (%): 296 (12) [M+$C_2H_5$]$^+$, 268 (52) [M+H]$^+$, 248 (100) [M-F]$^+$, 217 (8) [M-(F-OMe)], 187 (8); HRMS (APCI): calcd for $C_{14}H_{16}F_2NO_2$, 268.1144 [M+H]$^+$ found 268.1145; $t_R$ (GC) = 13.37 minutes.

**Preparation of ethyl (*E*)-3-((1R*,3S*)-2,2-difluoro-3-phenylcyclopropyl)-2-methylacrylate (20a)**

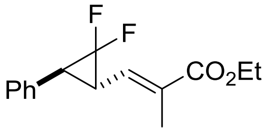

**20a**

Ester **20a** was prepared from alcohol **17b** (340 mg, 1.47 mmol), TEMPO (26 mg, 0.47 mmol), BAIB (512 mg, 1.62 mmol), (carbethoxyethylidene)triphenylphosphorane (692 mg, 1.9 mmol) and anhydrous DCM (10 mL) according to general procedure D with an oxidation time of 4 hours and an olefination time of 17 hours. Column chromatography on silca gel (1:19 $Et_2O$ in hexane) afford ester **20a** as a yellow oil (290 mg, 74%); $R_f$ = 0.47 (1:4 $Et_2O$ in hexane); $^1$H NMR (400 MHz, $CDCl_3$): δ = 7.39-7.30 (m, Ar*H*, 3H), 7.29-7.25 (m, Ar*H*, 2H), 6.59 (ddd, *J* = 9.7 Hz, $^4J_{H-F}$ = 3.0 Hz, $^4J$ = 1.5 Hz, C*H*=CCH$_3$, 1H), 4.25 (q, *J* = 7.2 Hz, OC*H*$_2$CH$_3$, 2H), 2.86 (dd, $J_{H-F}$ = 14.9 Hz, *J* = 7.2 Hz, ArC*H*CF$_2$CH, 1H), 2.70 (ddd, $J_{H-F}$ = 13.3 Hz, *J* = 9.7, 7.3 Hz, C*H*CH=CH$_3$, 1H), 2.00 (d, $^4J$ = 1.2 Hz, CH=C(C*H*$_3$), 3H), 1.34 ppm (t, *J* = 7.2 Hz, OCH$_2$C*H*$_3$, 3H); $^{13}$C NMR (100 MHz, $CDCl_3$): δ = 167.2, 133.4 (d, $^4J_{C-F}$ = 4.9 Hz) 132.6, 130.8, 128.7, 127.9, 127.6, 113.8 (t, $^1J_{C-F}$ = 292.5 Hz), 60.8, 36.2 (dd, $^2J_{C-F}$ = 11.8, 9.6 Hz), 31.2 (t, $^2J_{C-F}$ = 11.2 Hz),

14.3, 13.1 ppm; $^{19}$F NMR (376 MHz, $CDCl_3$): δ = -130.73 (dd, $^2J$ = 154.4 Hz, $J_{F-H}$ = 14.6 Hz, 1F), -135.5 (dd, $^2J$ = 154.8 Hz, $J_{F-H}$ = 13.5 Hz, 1F); $\bar{\nu}$/(film) = 2984, 2359, 2342, 1705, 1441, 1259 $cm^{-1}$; MS (CI): *m/z* (%): 275 (8) $[(M-F)+C_2H_5]^+$, 247 (100), $[M-F]^+$, 219 (11), [(M+H)-F-Et], 201 (20), 173 (39); HRMS (APCI): calcd for $C_{15}H_{17}F_2O_2$, 267.1191 $[M+H]^+$ found 267.1192; $t_R$ (GC) = 12.46 minutes.

**Preparation of ethyl (*E*)-3-((1R*,3S*)-2,2-difluoro-3-phenylcyclopropyl)acrylonitrile (21a) and (*Z*)-3-((1R*,3S*)-2,2-difluoro-3-phenylcyclopropyl)acrylonitrile (21b) mixture**

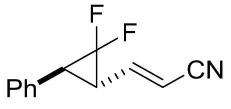

**21a**

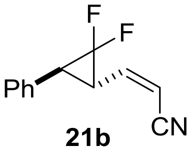

**21b**

(Triphenylphosphoranylidene)acetonitrile (536 mg, 1.8 mmol) was added to a colourless solution of aldehyde **18b** (250 mg, 1.37 mmol) in anhydrous DCM (6 mL) at room temperature under nitrogen. The resulting yellow reaction mixture changed to a red solution after being stirred for 2.5 hours. The reaction mixture was concentrated under reduced pressure and the resulting $^1$H NMR of crude product confirmed complete conversion of **18b**. Column chromatography on silca gel (1:4 $Et_2O$ in hexane) afforded a 3:2 diastereoisomeric mixture of cyano-**21a** and **21b** (237 mg, 84%). Mixed data for **21a** and **21b**: $R_f$ = 0.29 (1:4 $Et_2O$ in hexane); HRMS (APCI): calcd for $C_{12}H_{10}F_2N$, 206.0776 $[M+H]^+$ found 206.0774; $\bar{\nu}$/(film) = 3291, 2912, 1495, 1476, 1437, 1236, 1167, 1103, 1036, 1011, 930 $cm^{-1}$; Extracted data for **21a**: $^1$H NMR (400 MHz, $CDCl_3$): δ = 7.42-7.24 (m, Ar*H*, 5H), 6.53 (ddt, *J* = 16.3, 9.6 Hz, $^4J_{H-F}$ = 1.3 Hz, C*H*=CHCN, 1H), 5.60 (d, *J* = 16.4 Hz, CH=C*H*CN, 1H), 3.00-2.93 (m, ArC*H*, 1H), 2.65 ppm (ddd, $J_{H-F}$ = 12.3 Hz, *J* = 9.6, 7.2 Hz, C*H*CH=CH, 1H); $^{13}$C NMR (100 MHz, $CDCl_3$): δ = 146.9 (d, $^4J_{C-F}$ = 4.9 Hz), 131.5, 128.9, 128.1, 127.9, 116.8, 112.8 (t, $^1J_{C-F}$ = 293.6 Hz), 101.8, 36.4 (dd, $^2J_{C-F}$ = 11.9, 9.6 Hz), 34.2 ppm (t, $^2J_{C-F}$ = 11.6 Hz); $^{19}$F NMR (376 MHz, $CDCl_3$): δ = -129.9 (dd, $^2J$ = 158.1 Hz, $J_{F-H}$ = 15.3 Hz, 1F), -135.5 ppm (dd, $^2J$ = 158.1 Hz, $J_{F-H}$ = 12.10 Hz, 1F); MS (CI): *m/z* (%): 246 (11) $[M+C_3H_5]^+$, 234 (23) $[M+C_2H_5]^+$, 206 (94) $[M+H]^+$, 186 (100) $[M-F]^+$; $t_R$ (GC) = 11.45 minutes. Extracted data for **21b**: $^1$H NMR (400 MHz, $CDCl_3$): δ = 7.42-7.24 (m, Ar*H*, 5H), 6.32 (ddt, *J* = 11.0, 9.8 Hz, $^4J_{H-F}$ = 1.1 Hz, CHC*H*=CHCN, 1H), 5.55 (d, *J* = 11.1 Hz, CH=C*H*CN, 1H), 3.09 (ddd, $J_{H-F}$ = 12.3 Hz, *J* = 9.8, 6.9 Hz, C*H*CH=, 1H), 3.00-2.93 ppm (m, ArC*H*, 1H); $^{13}$C NMR (100 MHz, $CDCl_3$): δ = 146.2 (d, $^4J_{C-F}$ =

5.4 Hz), 131.2, 128.9, 128.1, 127.9, 115.8, 112.8 (t, $^{1}J_{C-F}$ = 293.1 Hz), 101.4, 36.6 (d, $^{2}J_{C-F}$ = 11.8, 9.22), 33.1 ppm (d, $^{2}J_{C-F}$ = 13.3, 10.4); $^{19}$F NMR (376 MHz, $CDCl_3$): δ = -130.0 (dd, $^{2}J$ = 156.9, $J_{H-F}$ = 15.0 Hz, 1F), -135.8 ppm (dd, $^{2}J$ = 156.9, $J_{H-F}$ = 12.2 Hz, 1F); MS (CI): *m/z* (%): 246 (6) $[M+C_3H_5]^+$, 234 (15) $[M+C_2H_5]^+$, 206 (22) $[M+H]^+$, 186 (100) $[M-F]^+$; $t_R$ (GC) = 11.86 minutes.

**Preparation of ethyl (*E*)-3-((1R*,3S*)-3-(benzo[d][1,3]dioxol-5-yl)-2,2-difluorocyclopropyl)acrylate (22a) and of ethyl (*Z*)-3-((1R*,3S*)-3-(benzo[d][1,3]dioxol-5-yl)-2,2-difluorocyclopropyl)acrylate (22b)**

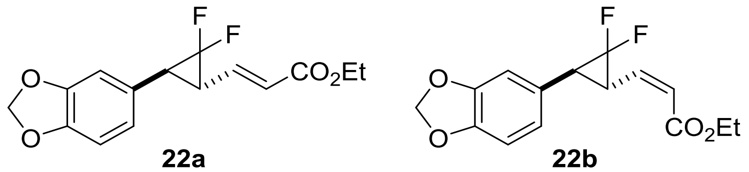


Ester **22a** was prepared from alcohol **17d** (36.5 mg, 0.16 mmol), TEMPO (3.7 mg, 0.02 mmol), BAIB (57 mg, 0.18 mmol), (ethoxycarbonylmethylene)triphenylphosphorane (85 mg, 0.21 mmol) and anhydrous DCM (2 mL) according to General Procedure D with an oxidation time of 4 hours and an olefination time of 4 hours. Column chromatography on silica gel (1:9 diethyl ether in hexane) afforded **22a** (23.5 mg, 50%) and a mixture of **22a** and **22b** (10 mg, 21%) as pale yellow oils. Data for **22a**: $R_f$ = 0.25 (1:4 $Et_2O$ in hexane); $^{1}$H NMR (400 MHz, $CDCl_3$): δ = 6.81-6.72 (m, Ar*H*, 3H), 6.77 (dd, *J* = 15.3, 8.9 Hz, C*H*=$CHCO_2Et$, 1H), 6.08 (d, *J* = 15.5 Hz, CH=C*H*$CO_2Et$, 1H), 5.98 (s, OC*H₂*O, 2H), 4.23 (q, *J* = 7.1 Hz, O$CH_2CH_3$, 2H), 2.84 ($J_{H-F}$ = 14.7 Hz, *J* = 7.1 Hz, ArC*H*, 1H), 2.54 (ddd, $J_{H-F}$ = 13.0 Hz, *J* = 8.9, 7.1 Hz, C*H*CH=CH, 1H), 1.33 (t, *J* = 7.1 Hz, $OCH_2CH_3$, 3H); $^{13}$C NMR (100 MHz, $CDCl_3$): δ = 165.2, 147.5, 146.7, 139.8 (d, $J_{C-F}$ = 4.7 Hz), 125.2, 123.1, 121.2, 112.7 (t, $^{1}J_{C-F}$ = 292.4 Hz), 107.9, 107.7, 100.8, 60.1, 35.2 (dd, $^{2}J_{C-F}$ = 12.1, 9.3 Hz), 33.4 (t, $^{2}J_{C-F}$ = 11.7 Hz), 13.7 ppm; $^{19}$F NMR (376 MHz, $CDCl_3$): δ = -130.6 (dd, $^{2}J$ = 156.5 Hz, $J_{F-H}$ = 14.7 Hz, 1F), -135.5 (dd, $^{2}J$ = 156.2 Hz, $J_{F-H}$ = 13.0 Hz, 1F); $\bar{\nu}$/(film) = 2924, 2359, 2342, 1715, 1505, 1493, 1445, 1256, 1188 1165, 1096, 1038, 1018, 789 $cm^{-1}$; MS (CI): m/z (%): 325 (4) $[M+C_2H_5]^+$, 305 (9) $[(M-F)+C_2H_5]^+$, 277 (100) $[M-F]^+$, 259 (11) $[M-F_2]^+$, 231 (44), 203 (24), 175 (5); HRMS (APCI): calcd for $C_{15}H_{18}F_2O_4N$, 314.1198 $[M+NH_4]^+$ found 314.1199; $t_R$ (GC) = 14.30 minutes. Extracted Data for **22b**: $R_f$ = 0.40 (1:4 $Et_2O$ in hexane); $^{19}$F NMR (376 MHz, $CDCl_3$): δ = -132.5 (dd, $^{2}J$ = 154.4 Hz, $J_{F-H}$ = 14.8 Hz, 1F), -136.1 (dd, $^{2}J$ = 153.6 Hz, $J_{F-H}$ = 13.5 Hz, 1F).

**Attempted preparation of ethyl (*E*)-3-((1R*,3S*)-2,2-difluoro-3-(furan-2-yl)cyclopropyl)acrylate (23a/23b)**

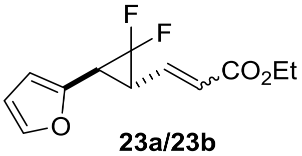

**23a/23b**

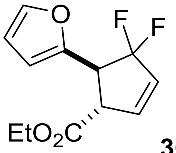

**30a**

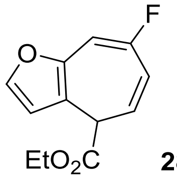

**28a**

The synthesis of ester **23a** was attempted from alcohol **17a** (1.33 g, 7.6 mmol), BAIB (2.67 mg, 8.36 mmol), TEMPO (60 mg, 1.52 mmol), (ethoxycarbonylmethylene)-triphenylphosphorane (5.3 g, 9.8 mmol) and DCM (30 mL) according to general procedure D with an oxidation time of 3 h and olefination time of 16 h. The $^{19}$F NMR of the resulting crude reaction mixture confirmed complete consumption of **17a**. Column chromatography on silica gel (1:19 $Et_2O$ in hexane) afforded a set of fractions containing rearrangement product **28a** with trace amounts of difluorocyclopentene **30a** (1.06 g, 80%) as a pale yellow oil. Further purification attempts failed as isolated compounds showed signs of decomposition (solution turned black). No evidence of ester **23** was observed during the reaction and initial purification, consistent with rearrangement occurring at room temperature. $^1$H and $^{19}$F NMR spectra are consistent with heptadiene **28a** and show similarities with the corresponding thiophene based product (**29a**) which was fully characterised (*vide infra*). Extracted data for **28a**: $R_f$ = 0.38 (1:4 $Et_2O$ in hexane); $^1$H NMR (400 MHz, $CDCl_3$): δ = 7.54 (d, *J* = 2.0 Hz, Ar*H*, 1H), 6.57 (dddd, $J_{H\text{-}F}$ = 15.2 Hz, $^4J$ = 2.0 Hz, $^5J$ = 0.7, 0.5 Hz, C-C*H*=CF, 1H), 6.28 (dd, *J* = 2.0 Hz, $^4J$ = 0.6 Hz, Ar*H*, 1H), 6.17 (dddd, $J_{H\text{-}F}$ = 11.5 Hz, *J* = 10.7 Hz, $^4J$ = 2.0 Hz, 0.5 Hz, (F)CC*H*=CH, 1H), 5.89 (ddd, *J* = 10.7, 7.2 Hz, $^4J_{H\text{-}F}$ = 4.1 Hz, $^5J$ = 0.5 Hz), 4.23 (ABq, *J* = 7.4 Hz, $^2J$ = 1.4 Hz, $OCH_aH_bCH_3$, 2H), 4.18 (br. d, *J* = 7.5 Hz, C(*H*)$CO_2$Et, 1H), 1.29 ppm (t, *J* = 7.2 Hz, $CH_2CH_3$, 3H); $^{19}$F NMR (376 MHz, $CDCl_3$): δ = -105.67 ppm (ddd, $J_{F\text{-}H}$ = 15.2, 11.5 Hz, $^4J_{F\text{-}H}$ = 4.2 Hz).

**Attempted preparation of ethyl (*E*)-3-((1R*,3S*)-2,2-difluoro-3-(thiophen-2-yl)cyclopropyl)acrylate (24a/24b)**

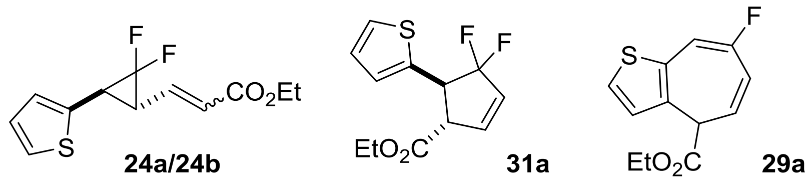

**24a/24b** **31a** **29a**

The synthesis of ester **24a** was attempted from alcohol **17c** (684 mg, 3.6 mmol), BAIB (1.26 g, 3.96 mmol), TEMPO (28 mg, 0.72 mmol), (ethoxycarbonylmethylene)-triphenylphosphorane (1.64 g, 4.68 mmol) and DCM (15 mL) according to general procedure D with an oxidation time of 3 h and an olefination time of 16 h. The $^{1}$H NMR spectrum of the crude reaction mixture confirmed complete consumption of **17c**. Column chromatography on silica gel (1:19 $Et_2O$ in hexane) afforded a set of fractions containing **24b** (12 mg), a mixture of rearrangement products and alkenoate **24a** (420 mg) and a mixture of rearrangement products **29a** and **31a** (212 mg). A solution of crude product containing **24a** in $CDCl_3$ (1 mL) and **24b** in $CDCl_3$ (1 mL) were heated at 40 °C and 50 °C, respectively for 17 h. $^{1}$H and $^{19}$F NMR showed complete consumption of VCP-precursors. Product samples containing a clean mixture of **29a** and **31a** were combined and concentrated under reduced pressure to afford a brown oil (488 mg). HPLC purification on a Kromasil C18 cartridge (2:5 to 1:1 gradient of MeCN containing 0.1% ammonia solution in 10 mM ammonium bicarbonate for 10 minutes then 1:1 to 9.9:10 of the same eluent for 30 minutes) afforded clean heptadiene **29a** (112 mg, 14%) as an orange oil and a brown oil containing a mixture of **29a** and dilfuorocyclopentene **31a** (24 mg, 1:1 by $^{1}$H NMR, the latter characterised by the distinctive $^{19}$F NMR signals). Data for **29a**: $R_f$ = 0.42 (1:4 $Et_2O$ in hexane); $^{1}$H NMR (400 MHz, $CDCl_3$): δ = 7.38 (d, $J$ = 5.2 Hz, Ar*H*, 1H), 6.80 (dd, $J_{H\text{-}F}$ = 15.6 Hz, $^{4}J$ = 1.7 Hz, C-C*H*=CF, 1H), 6.77 (d, $J$ = 5.24 Hz, Ar*H*, 1H), 6.21 (dddd, $J$ = 10.9 Hz, $J_{H\text{-}F}$ = 7.3 Hz, $^{4}J$ = 1.7, 0.8 Hz, CHCH=C*H*-CF, 1H), 6.01 (ddd, $J$ = 10.9, 7.0 Hz, $^{4}J_{H\text{-}F}$ = 4.6 Hz, CH-C*H*=CH, 1H), 4.29 (ABq, $J$ = 7.2 Hz, $^{2}J$ = 1.4 Hz, $OCH_aH_bCH_3$, 2H), 3.98 (d, $J$ = 7.0 Hz, C(*H*)$CO_2$Et, 1H), 1.32 ppm (t, $J$ = 7.2 Hz, $CH_2CH_3$, 3H); $^{13}$C NMR (100 MHz, $CDCl_3$): δ = 170.6, 158.8 (d, $^{1}J_{C\text{-}F}$ = 246 Hz), 132.3 (d, $J_{C\text{-}F}$ = 14.9 Hz), 129.7, 126.8 (d, $^{5}J_{C\text{-}F}$ = 3.1 Hz), 125.8 (d, $J_{C\text{-}F}$ = 13.0 Hz), 125.5, 120.5 (d, $^{2}J_{C\text{-}F}$ = 35.2 Hz), 106.1 (d, $^{2}J_{C\text{-}F}$ = 31.4 Hz), 60.9, 44.7, 13.7 ppm; $^{19}$F NMR (376 MHz, $CDCl_3$): δ = -103.4 ppm (ddd, $J_{F\text{-}H}$ = 15.6, 7.3 Hz, $^{4}J_{F\text{-}H}$ = 4.6 Hz); $\bar{\nu}$/(film) = 2980, 2937, 1733, 1632, 1444, 1402, 1370, 1333,

1308, 1186, 1137 $cm^{-1}$; MS (CI): *m/z* (%): 239 (41) $[M+H]^+$, 219 (35) $[M-F]^+$, 193 (25) $[M-OEt]^+$, 165 (100) $[M-CO_2Et]^+$, 147 (9); HRMS (APCI): calcd for $C_{12}H_{12}FSO_2$, 239.0542 $[M+H]^+$ found 239.0548; $t_R$ (GC) = 12.91 minutes. Extracted data for **31a**: $^1$H NMR (400 MHz, $CDCl_3$): δ = 7.31 (dd, $J$ = 5.1 Hz, $^4J$ = 1.3 Hz, Ar*H*, 1H), 7.09-7.07 (br m, containing d $J$ = 3.7 Hz, Ar*H*, 1H), 7.04 (dd, $J$ = 5.1, 3.7 Hz, Ar*H*, 1H), 6.46 (dt, $J$ = 6.0, 1.9 Hz, $^4J$ = 1.9 Hz, $CF_2$-CH=C*H*, 1H), 6.10 (dd, $J$ = 6.0 Hz, $^4J$ = 2.7 Hz, $CF_2$-C*H*=CH, 1H), 4.34-4.24 (m, under peak for **29a**, ArC*H*, 1H), 4.21 (q, $J$ = 7.2 Hz, $OCH_2CH_3$, 2H), 3.94-3.88 (m, C*H*$CO_2$Et, 1H), 1.28 ppm (t, $J$ = 7.2 Hz, $CH_2CH_3$, 3H); $^{19}$F NMR (376 MHz, $CDCl_3$): δ = -92.4 (ddd, $^2J$ = 250.5 Hz, $J_{F-H}$ = 14.8 Hz, $^4J_{F-H}$ = 7.6 Hz, $CF_aF_b$, 1F), -93.7 ppm (ddd, $^2J$ = 250.5 Hz, $J_{F-H}$ = 14.6 Hz, $^4J_{F-H}$ = 5.4 Hz, $CF_aF_b$, 1F); MS (CI): *m/z* (%): 267 (7) $[M-F+C_2H_5]^+$, 239 (100) $[M-F]^+$, 221 (8) $[M-F_2+H]^+$, 193 (25), 165 (12); $t_R$ (GC) = 12.11 minutes.

**Attempted preparation of ethyl (*E*)-3-((1R*,3S*)-2,2-difluoro-3-(furan-2-yl)cyclopropyl)acrylate (25a/25b)**

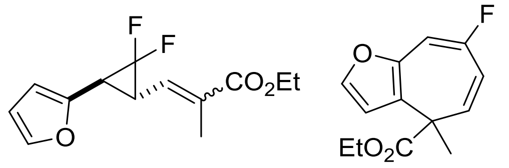


**25a/25b** **28b**

The synthesis of ester **25a** was attempted from alcohol **17a** (52 mg, 0.3 mmol), BAIB (106 mg, 0.33 mmol), TEMPO (5 mg, 0.03 mmol), (carbethoxyethylidene)triphenylphosphorane (142 mg, 0.39 mmol) and DCM (2 mL) according to general procedure D with an oxidation time of 3 h and an olefination time of 14 h. The $^1$H NMR spectrum of the crude reaction mixture confirmed complete consumption of **17a**, forming cycloheptadiene **28b** exclusively. The crude reaction mixture was transferred onto a pad of silica (10 g) in a sinter funnel which had been conditioned with hexane. The pad was eluted with 0:1 to 1:9 $Et_2O$ in hexane to afford cycloheptadiene **28b** as a pale yellow oil (34 mg, 48%). $R_f$ = 0.41(1:4 $Et_2O$ in hexane); $^1$H NMR (400 MHz, $CDCl_3$): δ = 7.53 (d, $J$ = 2.0 Hz, Ar*H*, 1H), 6.57 (dddd, $J_{H-F}$ = 14.9 Hz, $^4J$ = 2.0, 0.7 Hz, $^5J$ = 0.6 Hz, C-C*H*=CF, 1H), 6.28 (dd, $J$ = 2.0 Hz, $^4J$ = 0.7 Hz, Ar*H*, 1H), 6.14 (ddd, $J$ = 11.7 Hz, $J_{H-F}$ = 10.3 Hz, $^4J$ = 2.0 Hz, C(F)-C*H*=CH, 1H), 5.76 (dd, $J$ = 11.7 Hz, $^4J_{H-F}$ = 4.2 Hz, C(F)-CH=C*H*, 1H), 4.18 (q, $J$ = 7.2 Hz, $OCH_2CH_3$, 2H), 1.64 (s, $CH_3$, 3H), 1.25 ppm (t, $J$ = 7.2 Hz, $CH_2CH_3$, 3H); $^{13}$C NMR (150 MHz, $CDCl_3$): δ = 173.9, 157.8 (d, $^1J_{C-F}$ = 241.0 Hz), 146.2 (d,

$^{4}J_{C-F}$ = 15.9 Hz), 144.4 (d, $^{5}J_{C-F}$ = 4.1 Hz), 133.2 (d, $J_{C-F}$ = 13.1 Hz), 120.5 (d, $^{2}J_{C-F}$ = 37.7 Hz), 120.3 (d, $J_{C-F}$ = 3.9 Hz), 109.9, 102.5 (d, $J_{C-F}$ =33.7 Hz), 61.4, 44.9, 24.0, 14.1 ppm; $^{19}$F NMR (376 MHz, $CDCl_3$): δ = -107.05 (ddd, $J_{F-H}$ = 14.9, 10.3 Hz, $^{4}J_{F-H}$ = 4.2 Hz); $\bar{\nu}$/(film) =2980, 2934, 1794, 1731, 1641, 1459, 1405, 1376, 1251, 1216, 1149, 1108, 1017 cm$^{-1}$; MS (CI): *m/z* (%): 237 (33) $[M+H]^+$, 217 (30) $[M-F]^+$, 191 (15) $[M-OEt]^+$, 189 (13) $[M-(F+Et)+H]^+$, 163 (100) $[M-CO_2Et]^+$, 145 (8) $[M-(CO_2Et+F)+H]^+$; HRMS (APCI): calcd for $C_{13}H_{17}F_1N_1O_3$, 254.1187 $[M+NH_4]^+$ found 254.1189; $t_R$ (GC) = 12.05 minutes.

**Attempted preparation of ethyl (*E*)-3-((1R*,3S*)-2,2-difluoro-3-(thiophen-2-yl)cyclopropyl)acrylate (26a/26b)**

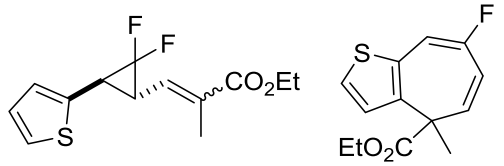

**26a/26b** **29b**

The synthesis of ester **26a** was attempted from alcohol **17c** (93 mg, 0.48 mmol), BAIB (171 mg, 0.53 mmol), TEMPO (7.5 mg, 0.05 mmol), (carbethoxyethylidene)triphenylphosphorane (226 mg, 0.62 mmol) and DCM (3.2 mL) according to general procedure D with an oxidation time of 3 h and an olefination time of 14 h. The $^{1}$H NMR spectrum of the crude reaction mixture confirmed complete consumption of **17c**, forming cycloheptadiene **29b** exclusively. The crude reaction mixture was transferred onto a pad of silica (10 g) in a sinter funnel which had been conditioned with hexane. The pad was eluted with 0:1 to 1:9 $Et_2O$ in hexane to afford cycloheptadiene **29b** as a pale yellow oil (66 mg, 55%). $R_f$ = 0.39 (1:4 $Et_2O$ in hexane); $^{1}$H NMR (400 MHz, $CDCl_3$): δ = 7.39 (d, *J* = 5.4 Hz, Ar*H*, 1H), 6.88 (d, *J* = 5.4 Hz, Ar*H*, 1H), 6.76 (dd, $J_{H-F}$ = 15.4 Hz, $^{4}J$ = 2.0 Hz, C-C*H*=CF, 1H), 6.24 (ddd, *J* = 11.1 Hz, $J_{H-F}$ = 8.2 Hz, $^{4}J$ = 2.0 Hz, CF-C*H*=CH, 1H), 5.80 (dd, *J* = 11.1 Hz, $^{4}J_{H-F}$ = 4.6 Hz, CF-CH=C*H*, 1H), 4.14 (q, *J* = 7.2 Hz, $OCH_2CH_3$, 2H), 1.79 (s, C-C*H*$_3$, 3H), 1.20 ppm (t, *J* = 7.2 Hz, $CH_2CH_3$, 3H); $^{13}$C NMR (100 MHz, $CDCl_3$): δ = 173.8, 158.2 (d, $^{1}J_{C-F}$ = 244.5 Hz), 135.4 (d, $^{4}J_{C-F}$ = 2.0 Hz), 132.9 (d, $J_{C-F}$ = 13.1 Hz), 132.0 (d, $J_{C-F}$ = 13.5 Hz), 126.4 (d, $^{5}J_{C-F}$ = 3.3 Hz), 125.3, 120.0 (d, $^{2}J_{C-F}$ = 36.0 Hz), 105.9 (d, $^{2}J_{C-F}$ = 31.3 Hz), 60.8, 47.6, 23.3, 13.6 ppm; $^{19}$F NMR (376 MHz, $CDCl_3$): δ = -106.10 (ddd, $J_{F-H}$ = 15.4, 8.2 Hz, $^{4}J_{F-H}$ = 4.6 Hz); $\bar{\nu}$/(film) = 2980, 2937, 1733, 1632, 1444, 1402, 1370, 1333, 1308, 1186, 1137 cm$^{-1}$; MS (CI): *m/z* (%): 253 (31) $[M+H]^+$, 233 (53) $[M-F]^+$, 207 (19) $[M-OEt]^+$, 179

(100) [M-$CO_2$Et]$^+$, 161 (8) [M-($CO_2$Et+F)+H]$^+$; HRMS (APCI): calcd for $C_{13}H_{14}F_1S_1O_2$, 253.0693 [M+H]$^+$ found 253.0696; $t_R$ (GC) = 13.17 minutes.

**Attempted preparation of ethyl (*E*)-3-((1R*,3S*)-2,2-difluoro-3-(2'-furyl-3-methyl)cyclopropyl)acrylate (S9a/S9b)**

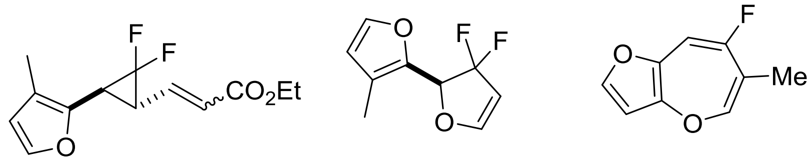


**S9a/S9b** **33** **34**

The synthesis of ester **S9a** was attempted from alcohol **17h** (71 mg, 0.38 mmol), BAIB (144 mg, 0.45 mmol), TEMPO (11 mg, 0.08 mmol), (carbethoxyethylidene)triphenylphosphorane (183 mg, 0.53 mmol) and DCM (3 mL) according to general procedure D with an oxidation time of 6 h and an olefination time of 16 h. Crude $^1$H and $^{19}$F NMR after oxidation suggested rearrangement had occurred before addition of phosphorane. The reaction mixture was concentrated under reduced pressure and crude $^1$H and $^{19}$F NMR showed no new formation of rearrangement products. Attempted chromoatographic purification on silica gel (0:1 to 1:4 $Et_2O$ in hexane) failed to isolated desired rearrangement products either due to product volatility or decomposition on silica. $^{19}$F NMR of crude products showed strong similarities to 3,3-difluoro-4,5-dihydrofurans reported by Hammond and Arimitsu ($^{19}$F NMR: $\delta$ = -84.6 (ddd, $J$ = 248.0, 23.1, 19.8 Hz, 1F) -87.2 ppm (ddd, $J$ = 248.0, 13.2, 13.2 Hz, 1F))[S19] and benzoheptadiene compounds reported previously by ourselves ($^{19}$F NMR: $\delta$ = -100.7 ppm (dt, $J_{F\text{-}H}$ = 18.2, 5.2 Hz, $^4J_{F\text{-}H}$ = 5.2 Hz, 1F).[S20] Due to these similarities the crude products were tentatively assigned as dihydrofuran **33** and ether **34** (1:2.5 ratio respectively by $^{19}$F NMR); the doublet of doublet observed for the latter suggest the methyl group underwent [1,5]-shift during the re-aromatisation of the furan (**Scheme S4**).

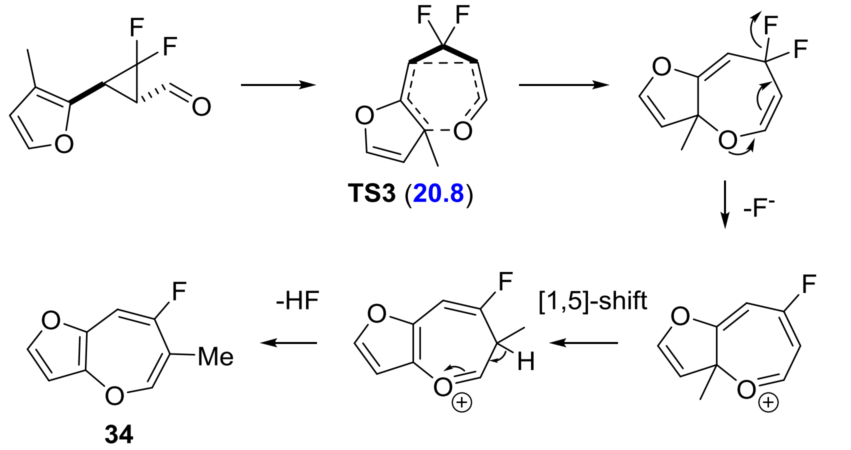


**Scheme S4**: Proposed mechanism rearrangement of **18h** to **34** through rearrangements ($\Delta G^{\ddagger}$ value (**blue**) quoted in kcal $mol^{-1}$)

Data for **33**: $^{19}F$ NMR (376 MHz, $CDCl_3$): δ = -81.9 (dd, $^2J$ = 248.0 Hz, $J_{F\text{-}H}$ = 20.0 Hz, 1F), -89.3 ppm (dt, $^2J$ = 248.0 Hz, $J_{F\text{-}H}$ = 13.8 Hz, 1F). Data for **34**: $^{19}F$ NMR (376 MHz, $CDCl_3$): δ = -99.4 ppm (dd, $J_{F\text{-}H}$ = 34.0 Hz, $^4J_{F\text{-}H}$ = 18.5 Hz).

**General Procedure E**: Thermal Rearrangement of Vinyl Difluorocyclopropanes

**Preparation of (1R*,5R*)-4,4-difluoro-N-methoxy-N-methyl-5-phenylcyclopent-2-ene-1-carboxamide (35)**

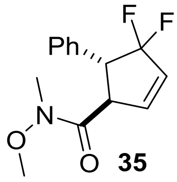

**35**

A solution of **19a** (7.0 mg, 0.026 mmol) in toluene (0.3 mL) was heated to 95 °C in a sealed microwave vial for 17 hours in a DrySyn block. After cooling and venting the vial, $^{19}F$ NMR confirmed complete consumption of **19a**. The reaction mixture was transferred to a round bottom flask using DCM (5 mL) and concentrated under reduced pressure to afford difluorocyclopentene **35** (6.8 mg, 97%) as a yellow oil. $R_f$ = 0.36 (1:1 $Et_2O$ in hexane); $^1H$ NMR (400 MHz, $CDCl_3$): δ = 7.40-7.31 (m, Ar*H*, 5H), 6.40 (dt, *J* = 6.0, 1.6 Hz, $J_{H\text{-}F}$ = 1.6 Hz, =C*H*CHCO, 1H), 6.10 (dd, *J* = 6.0 Hz, $^4J$ = 2.5 Hz, $CF_2CH$=CH, 1H), 4.34 (dddd, $^4J_{H\text{-}F}$ = 8.9, 5.2 Hz, *J* = 6.7, 1.6 Hz, COC*H*, 1H), 4.11 (ddd, $J_{H\text{-}F}$ = 16.1, 15.4 Hz, *J* = 6.7 Hz, PhC*H*, 1H), 3.49 (s, $OCH_3$, 3H), 3.20 ppm (s, $OCH_3$, 3H); $^{13}C$ NMR (100 MHz, $CDCl_3$): δ = 170.5, 139.1 (t, $J_{C\text{-}F}$ = 10.0 Hz), 134.1, 129.6 (t, $^1J_{C\text{-}F}$ = 254.7 Hz), 128.9, 128.3 (dd, $^2J_{C\text{-}F}$ = 30.1, 25.2 Hz), 128.0, 127.3, 60.9, 53.2 (t,

$^{2}J_{C\text{-}F}$ = 24.5 Hz), 51.4 (d, $J_{C\text{-}F}$ = 5.3 Hz), 31.9 ppm; $^{19}$F NMR (376 MHz, $CDCl_3$): δ = -90.2 (ddd, $^{2}J$ = 251.0 Hz, $J_{F\text{-}H}$ = 16.1, $^{4}J_{H\text{-}F}$ = 8.9 Hz, 1F), -92.0 ppm (ddd, $^{2}J$ = 251.1 Hz, $J_{F\text{-}H}$ = 15.4, $^{4}J_{H\text{-}F}$ = 5.2 Hz, 1F); $\bar{\nu}$/(film) = 2963, 2940, 2361, 2342, 1661, 1456, 1418, 1387, 1364, 1350, 1169, 1051, 1034, 1026, 1093 $cm^{-1}$; MS (CI): *m/z* (%): 296 (19) $[(M+H)\text{-}F]^+$, 276 (26) $[(M+H)\text{-}F]^+$, 248 (100) $[M\text{-}F]^+$, 218 (10) $[(M+H)\text{-}OMe\text{-}F]^+$, 187 (16), 161 (6); HRMS (APCI): calcd for $C_{14}H_{15}F_2NO_2Na$, 290.0963 $[M+Na]^+$ found 290.0964; $t_R$ (GC) = 12.97 minutes.

**Thermolysis of (*Z*)-3-((1R*,3S*)-2,2-difluoro-3-phenylcyclopropyl)-N-methoxy-N-methylacrylamide (19b)**

A solution of **19b** (126 mg, 0.47 mmol) in $Ph_2O$ (0.5 mL) was heated at 165 °C for 17 h according to General Procedure E. The crude reaction mixture was loaded onto a silica pad (10 g) conditioned with hexane. $Ph_2O$ was eluted with hexane then crude products were eluted with $Et_2O$, collected and concentrated under reduced pressure to afford a brown oil (53 mg). $^{1}$H and $^{19}$F NMR confirmed full consumption of alkenoate **19b**. Column chromatography on silica gel (1:9 to 2:5 EtOAc in hexane) was used to separated two major unknown products as yellow oils; unknown **S10a** (13 mg) and unknown **S10b** (7 mg). Lack of distinctive Weinreb amide signals in the $^{1}$H NMR suggests decomposition had occurred. Data for **S10a**: $R_f$ = 0.21 (1:1 $Et_2O$ in hexane); $^{19}$F NMR (376 MHz, $CDCl_3$): δ = -100.41 (d, $J_{F\text{-}H}$ = 15.9 Hz). Data for **S10b**: $R_f$ = 0.39 (1:1 $Et_2O$ in hexane); $^{19}$F NMR (376 MHz, $CDCl_3$): δ = -87.39 (s).

**Attempted preparation of ethyl (1R*,5S*)-4,4-difluoro-1-methyl-5-phenylcyclopent-2-ene-1-carboxylate (36)**

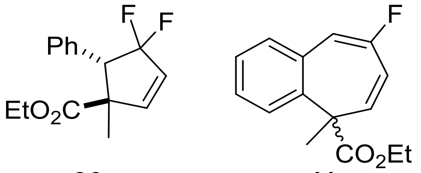


**36** **41**

A solution of **20** (100 mg, 0.37 mmol) in $Ph_2O$ (0.45 mL) was heated at 155 °C for 17 h according to General Procedure E. The crude reaction mixture was loaded onto a silica pad (16 g) conditioned with hexane. $Ph_2O$ was eluted with hexane then crude products were eluted with $Et_2O$, collected and concentrated under reduced pressure to afford crude reaction mixture as a brown oil (100 mg). $^{1}$H and $^{19}$F NMR confirmed full consumption of alkenoate **20**. Column chromatography on silica gel (0:1 to 1:19 EtOAc in hexane) was used

to separated two major unknown products as yellow oils; unknown **S11a** (8 mg) and unknown **S11b** (29 mg). Product assignment could not be determined due to complexity in $^{1}$H NMR spectra. However, the major $^{19}$F NMR signal for **S11b** was consistent with previously observed benzoheptadiene compounds and tentatively assigned as benzoheptadiene **41**. No evidence for VCPR rearrangement to difluorocyclopentene **36**.

Data for **S11a**: $R_f$ = 0.18 (1:19 EtOAc in hexane); $^{19}$F NMR ($CDCl_3$, 376 MHz): $\delta$ = -103.67 (minor peak, dd, $J_{F-H}$ = 12.0, 7.5 Hz), -110.58 ppm (major peak, s). Data for **S11b**: $R_f$ = 0.28 (1:19 EtOAc in hexane); $^{19}$F NMR ($CDCl_3$, 376 MHz): $\delta$ = -95.84 (minor peak, dd, $J_{F-H}$ = 32.9, 15.0 Hz), -104.43 ppm (major peak, dt, $J_{F-H}$ = 17.8, 5.4 Hz, $^{4}J_{F-H}$ = 5.4 Hz).

**Preparation of (1R*,5R*)-4,4-difluoro-5-phenylcyclopent-2-ene-1-carbonitrile (37) and 4,4-difluoro-5-phenylcyclopent-1-ene-1-carbonitrile (43)**

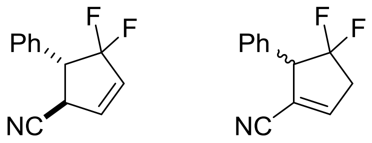


**37** **43**

A solution of mixture **21a**/**21b** (100 mg, 0.48 mmol) in $Ph_2O$ (1.0 mL) was heated to 90 °C in a sealed microwave vial for 17 hours in a DrySyn block. After cooling and venting, the resulting $^{19}$F NMR spectrum confirmed full conversion of **21a**. The microwave vial was resealed and heated to 160 °C for 17 hours in a DrySyn block. After cooling and venting, the resulting $^{19}$F NMR spectrum confirmed full consumption of **21b**. The crude reaction mixture was transferred onto a pad of silica (15 g) in a sinter funnel which has been conditioned with hexane. $Ph_2O$ was eluted using hexane and then crude product fractions were eluted using $Et_2O$, collected and concentrated under reduced pressure to afford a red oil (106 mg). Column chromatography on silica gel (0:1 to 1:9 $Et_2O$ in hexane) afforded a mixture difluorocyclopentenes **37** and **43** (6:1 ratio by $^{1}$H NMR) as a yellow oil (44.7 mg, 48%). Data for mixture of **37** and **43**: $R_f$ = 0.21 (1:9 $Et_2O$ in hexane); $\bar{\nu}$/(film) = 3091, 3069, 3036, 2915, 2249, 2229, 1502, 1457, 1349, 1178 $cm^{-1}$; HRMS (APCI): calcd for $C_{12}H_8F_2N$, 204.0625 [M-H] found 204.0634; Data for **37**: $^{1}$H NMR (400 MHz, $CDCl_3$): δ = 7.47-7.40 (m, Ar*H*, 3H), 7.35-7.32 (m, Ar*H*, 2H), 6.45 (dt, *J* = 6.0, 1.9 Hz, $^{4}J_{H-F}$ = 1.3 Hz, C($F_2$)CH=C*H*, 1H) 6.26 (dd, *J* = 6.0 Hz, $J_{H-F}$ = 2.6 Hz, C($F_2$)-C*H*=CH, 1H), 3.99 (dddd, *J* = 7.6, 2.0 Hz, $^{4}J_{H-F}$ = 9.6, 5.0 Hz, C(*H*)CN, 1H),

3.90 ppm (ddd, $J$ = 7.6 Hz, $J_{H\text{-}F}$ = 14.6, 13.7 Hz, ArC$H$, 1H); $^{13}$C NMR (100 MHz, $CDCl_3$): δ = 134.8 (t, $J_{C\text{-}F}$ = 10.0 Hz), 131.0, 130.4 (dd, $^2J_{C\text{-}F}$ = 30.1, 24.1 Hz), 128.5, 128.4 (represents 2 carbon atoms), 127.4 (t, $^1J_{C\text{-}F}$ = 246.7 Hz), 117.3 (d, $^4J_{C\text{-}F}$ = 5.2 Hz), 5.4 (t, $^2J_{C\text{-}F}$ = 25.4 Hz), 38.3 ppm (d, $J_{C\text{-}F}$ = 6.4 Hz); $^{19}$F NMR (376 MHz, $CDCl_3$): δ = -90.98 (ddd, $^2J$ = 255.6 Hz, $J_{F\text{-}H}$ = 14.9 Hz, $^4J_{F\text{-}H}$ = 9.6 Hz, C$F_a$F$_b$, 1F), -93.00 ppm (ddd, $^2J$ = 255.6 Hz, $J_{F\text{-}H}$ = 13.7 Hz, $^4J_{F\text{-}H}$ = 14.6 Hz, CF$_a$$F_b$, 1F); MS (CI): $m/z$ (%): 246 (10) $[M+C_3H_5]^+$, 234 (20) $[M+C_2H_5]^+$, 206 (77) $[M+H]^+$, 186 (100) $[M-F]^+$; $t_R$ (GC) = 11.31 minutes. Data for **47**: $^1$H NMR (400 MHz, $CDCl_3$): δ = 7.47-7.40 (m, Ar$H$, 3H), 7.24-7.22 (m, Ar$H$, 2H), 6.87 (dtt, $J$ = 5.0 Hz, $^4J_{H\text{-}F}$ = 2.6 Hz, $^4J$ = 2.2 Hz, $CF_2$-$CH_2$-C$H$=, 1H), 4.36 (dddt, $J_{H\text{-}F}$ = 18.0, 7.9 Hz, $^4J$ = 2.2, 1.4 Hz, ArC$H$, 1H), 3.17-3.09 (m, $CF_2$-C$H_2$-CH=,2H); $^{13}$C NMR (100 MHz, $CDCl_3$): δ = 143.9 (t, $J_{C\text{-}F}$ = 4.1 Hz), 129.9, 128.3, 128.2 (represents 2 carbon atoms), 127.1 (t, $^1J_{C\text{-}F}$ = 255.1 Hz), 115.9 (dd, $J_{C\text{-}F}$ = 6.7, 2.8 Hz), 114.1, 58.7 (dd, $^2J_{C\text{-}F}$ = 26.7, 25.4 Hz), 41.9 ppm (dd, $J_{C\text{-}F}$ = 28.9, 28.3 Hz); $^{19}$F NMR (376 MHz, $CDCl_3$): δ = (-90.62)-(-91.40) (m, C$F_a$F$_b$, underneath $^{19}$F signal for major product **37**), (-98.05)-(-98.76) ppm (m, containing d, $^2J$ = 233.1 Hz, CF$_a$$F_b$); MS (CI): $m/z$ (%): 246 (7) $[M+C_3H_5]^+$, 234 (17) $[M+C_2H_5]^+$, 206 (100) $[M+H]^+$, 186 (35) $[M-F]^+$; $t_R$ (GC) = 11.50 minutes.

**Preparation of ethyl (1R,5R)-5-(benzo[d][1,3]dioxol-5-yl)-4,4-difluorocyclopent-2-ene-1-carboxylate (39) and ethyl 8-fluoro-5H-cyclohepta[4,5]benzo[1,2-d][1,3]dioxole-5-carboxylate (44)**

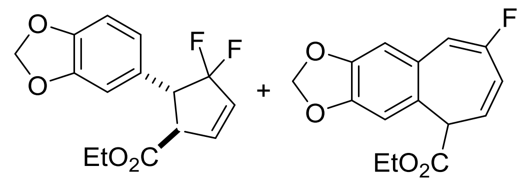


**39** **44**

A solution of **22a** (445 mg, 0.07 mmol) in a toluene (7.5 mL) was heated to 70 °C for 17 h according the General Procedure E. $^1$H and $^{19}$F NMR showed full consumption of **22a** to a mixture of difluorocyclopentene **39** and benzoheptadiene **44** (42:58 respectively, relative ratio by $^{19}$F NMR). HPLC purification on a Kromasil C18 cartridge (3:10 to 9.9:10 gradient of MeCN containing 0.1% ammonia solution in 10 mM ammonium bicarbonate) afford clean fluoroheptadiene **44** (45 mg, 11%) as a white solid and dilfuorocyclopentene **39** (82 mg, 18%). Note: poor yield could be due to partial solubility of crude reaction mixture in loading solvent for HPLC purification (1:1 DMSO/MeOH). Data for **39**: $R_f$ = 0.35 (1:4 $Et_2O$ in hexane);

$^1H$ NMR (400 MHz, $CDCl_3$): δ = 6.81 (s, Ar*H*, 3H), 6.48 (ddd, $J$ = 6.0 Hz, $J_{H\text{-}F}$ = 1.7 Hz, $^4J$ = 1.7 Hz, $CF_2$-C*H*=CH, 1H), 6.07 (dd, $J$ = 6.0, 2.5 Hz, CH=C*H*, 1H), 5.98 (s, $O_2CH_2$, 2H), 4.19 (q, $J$ = 7.0 Hz, $OCH_2CH_3$, 2H), 3.95-3.82 (m, ArC*H* and *H*$CCO_2$Et, 2H), 1.26 ppm (t, $J$ = 7.0 Hz, $CH_2CH_3$, 3H); $^{13}C$ NMR (100 MHz, $CDCl_3$): δ = 170.2 (d, $^4J_{C\text{-}F}$ = 2.7 Hz), 147.3, 146.8, 138.4 (t, $J_{C\text{-}F}$ = 10.4 Hz), 129.0 (t, $^1J_{C\text{-}F}$ = 245.7 Hz), 128.4 (dd, $^2J_{C\text{-}F}$ = 30.8, 24.5 Hz), 127.2, 122.3, 108.8, 107.7, 100.6, 61.0, 54.2 (d, $J_{C\text{-}F}$ = 5.8 Hz), 53.1 (t, $^2J_{C\text{-}F}$ = 24.0 Hz), 13.6 ppm; $^{19}F$ NMR (376 MHz, $CDCl_3$): δ = -90.21 (ddd, $^2J$ = 252.5 Hz, $J_{F\text{-}H}$ = 16.4, $^4J_{F\text{-}H}$ = 8.6 Hz, $CF_aF_b$, 1F), -93.07 ppm (ddd, $^2J$ = 252.5 Hz, $J_{F\text{-}H}$ = 14.1, $^4J_{F\text{-}H}$ = 5.1 Hz, $CF_aF_b$, 1F); $\bar{\nu}$/(film) = 2982, 2904, 1614, 1731, 1506, 1493, 1446, 1245, 1167, 1018 $cm^{-1}$; MS (CI): *m/z* (%): 277 (100) $[M-F]^+$, 231 (16), 203 (15), 175 (4) $[M-C_7O_2H_5]^+$; HRMS (APCI): calcd for $C_{15}H_{14}FO_4$, 277.0876 [M-F] found 277.0905; $t_R$ (GC) = 14.09 minutes. Data for **44**: m.p. = 118-120 °C (chloroform/pentane); $R_f$ = 0.40 (1:4 $Et_2O$ in hexane); $^1H$ NMR (400 MHz, $CDCl_3$): δ = 6.82 (s, Ar*H*, 1H), 6.80 (d, $J_{H\text{-}F}$ = 18.1 Hz, C-C*H*=CF, 1H), 6.59 (s, Ar*H*, 1H), 6.22-6.14 (m, representing CF-C*H*=CH and C(H)-C*H*=CH, 2H), 6.01 (d, $^2J$ = 1.4 Hz, $OCH_aH_bO$, 1H), 5.98 (d, $^2J$ = 1.4 Hz, $OCH_aH_bO$, 1H), 4.30 (ABq, $J$ = 7.2 Hz, $^2J$ = 1.9 Hz, $OCH_aH_bCH_3$, 2H), 3.69 (d, $J$ = 5.2 Hz, *H*$CCO_2$Et, 1H), 1.31 ppm (t, $J$ = 7.2 Hz, $CH_2CH_3$, 3H); $^{13}C$ NMR (100 MHz, $CDCl_3$): δ = 171.0, 158.6 (d, $^2J_{C\text{-}F}$ = 245.5 Hz), 148.6, 146.1, 127.7 (d, $J_{C\text{-}F}$ = 12.5 Hz), 126.0 (d, $J_{C\text{-}F}$ = 11.4 Hz), 125.8, 119.3 (d, $^2J_{C\text{-}F}$ = 35.1 Hz), 113.0 (d, $^2J_{C\text{-}F}$ = 27.9 Hz), 107.0 (d, $^5J_{C\text{-}F}$ = 3.8 Hz), 105.1, 101.1, 60.9, 48.9, 13.7 ppm; $^{19}F$ NMR (376 MHz, $CDCl_3$): δ = -102.97 ppm (ddd, $J_{F\text{-}H}$ = 18.1, 4.8 Hz, $^4J_{C\text{-}F}$ = 4.8 Hz); $\bar{\nu}$/(film) = 2982, 2904, 1733, 1645, 1506, 1485, 1374, 1333, 1305, 1238, 1193 $cm^{-1}$; MS (CI): *m/z* (%): 305 (11) $[M+C_2H_5]^+$, 277 (65) $[M+H]^+$, 257 (41) $[M+H]^+$, 231 (100) $[M-OEt]^+$, 203 (40) $[M-CO_2Et]^+$, 57 (80); HRMS (ASAP+): calcd for $C_{15}H_{14}FO_4$, 277.0876 $[M+H]^+$ found 277.0884; $t_R$ (GC) = 14.97 minutes.

**Preparation of ((1S*,2S*)-2-phenylcyclopropyl)methanol (S12)**

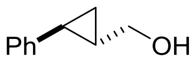


**S12**

Diiodomethane (1 ml, 12.5 mmol) was added dropwise to a solution of diethylzinc (6.25 mL of a 1 M solution in hexane, 6.25 mmol) in DCM (2 mL) at 0 °C over a period of 5 minutes and stirred for 20 minutes under nitrogen. A solution of cinnamyl alcohol (335 mg, 1.25 mmol) in DCM (1.5 mL) was added dropwise and the reaction mixture and stirred for 2.5 hours at 0 °C. The reaction mixture was quenched with saturated aqueous $NH_4Cl$ (10 ml) and

EtOAc (10 ml). The organic layer was separated and the aqueous layer extracted further with EtOAc (3 x 10 ml). The original organic layer and extracts were combined, dried ($MgSO_4$) and concentrated under reduced pressure to remove volatile organic solvent. Column chromatography on silica gel (2:3 EtOAc in hexane) afforded alcohol **S12** as a yellow oil (93 mg, 52%). $R_f$ = 0.23 (1:1 $Et_2O$ in hexane); $^1$H NMR (400 MHz, $CDCl_3$): δ = 7.29-7.26 (m, Ar*H*, 2H), 7.17 (tt, *J* = 7.3 Hz, $^4J$ = 1.2 Hz, Ar*H*, 1H), 7.10-7.08 (m, Ar*H*, 2H), 3.64 (dd, $^2J$ = 11.3 Hz, *J* = 6.8 Hz, $CH_aH_bOH$, 1H), 3.62 (dd, $^2J$ = 11.3 Hz, *J* = 6.8 Hz, $CH_aH_bOH$, 1H), 1.84 (td, *J* = 9.8, 4.9 Hz, PhC*H*, 1H), 1.78 (br. s, O*H*, 1H), 1.50-1.44 (m, $CH_2CH(CH_2)CH$, 1H), 1.00-0.92 ppm (m, $CH_2$, 2H); $^{13}$C NMR (100 MHz, $CDCl_3$): δ = 142.5, 128.4, 125.9, 125.7, 66.5, 25.3, 21.3, 13.8 ppm; $\bar{\nu}$/(film) = 3333, 3026, 3003, 2361, 2342, 1605, 1497, 1018, 743, 696 cm$^{-1}$; MS (CI): *m/z* (%): 159 (14) [(M+H)-OH]$^+$, 131 (100) [M-OH]$^+$; 117 (29), 91 (28); $t_R$ (GC) = 11.10 minutes. Data was in agreement with those reported by Charette and co-workers.[S21]

**Preparation of ethyl (*E*)-3-((1R*,2S*)-2-phenylcyclopropyl)acrylate (47)**

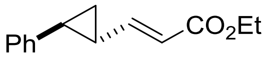


**47**

Ester **47** was prepared from alcohol **S12** (100 mg, 0.67 mmol), TEMPO (10 mg, 0.067 mmol), BAIB (237 mg, 0.74 mmol), (carbethoxyethylidene)triphenylphosphorane (303 mg, 0.87 mmol) and anhydrous DCM (1 mL) according to general procedure D with an oxidation time of 3.5 hours and an olefination time of 20 hours. Column chromatography on silica gel (1:9 $Et_2O$ in hexane) afforded ester **47** as a colourless oil (100 mg, 67%). $R_f$ = 0.42 (1:4 $Et_2O$ in hexane); $^1$H NMR (400 MHz, $CDCl_3$): δ = 7.33-7.28 (m, Ar*H*, 2H), 7.21 (tt, *J* = 7.3 Hz, $^4J$ = 1.3 Hz, Ar*H*, 1H), 7.12-7.09 (m, Ar*H*, 2H), 6.62 (dd, *J* = 15.5, 9.9 Hz, CHC*H*=CH, 1H), 5.92 (d, *J* = 15.5 Hz, CH=C*H*, 1H), 4.21 (q, *J* = 7.2 Hz, $OCH_2CH_3$, 2H), 2.20 (ddd, *J* = 9.5, 6.2, 4.3 Hz, PhC*H*, 1H), 1.84 (dddd, *J* = 9.9, 8.4, 5.4, 4.1 Hz, $CH_2CHCH$=CH, 1H), 1.47 (ddd, $^2J$ = 8.5, *J* = 6.6, 5.2 Hz, $CHCH_aH_bCH$, 1H), 1.36-1.30 ppm (m, (incld. 1.31 ppm, t, *J* = 7.2 Hz, $OCH_2CH_3$, 3H), 4H); $^{13}$C NMR (100 MHz, $CDCl_3$): δ = 166.2, 151.0, 140.3, 128.0, 125.7, 125.4, 118.4, 59.6, 26.4, 26.2, 17.2, 13.8 ppm; $\bar{\nu}$/(film) = 2980, 2359, 2342, 1709, 1643, 1256, 1175, 1036 cm$^{-1}$; MS (CI): *m/z* (%): 245 (6) [M+$C_2H_5$]$^+$, 217 (56) [M+H]$^+$, 171 (50) [M-OEt]$^+$, 143 (100) [M-$CO_2$Et]$^+$, HRMS (APCI): calcd for $C_{14}H_{17}O_2$, 217.1223 [M+H]$^+$ found 217.1224; $t_R$ (GC) = 13.57 minutes.

**Preparation of ethyl (1R*,5R*)-5-phenylcyclopent-2-ene-1-carboxylate (38a) and ethyl (1R*,5S*)-5-phenylcyclopent-2-ene-1-carboxylate (38b)**

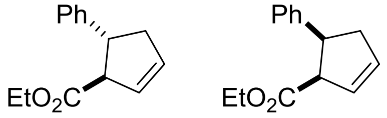

**38a** **38b**

A solution of **47a** (100 mg, 0.46 mmol) in a $Ph_2O$ (0.5 mL) was heated to 220 °C for 17 h according the General Procedure E. The crude reaction mixture was transferred onto a pad of silica (10 g) in a sinter funnel which has been conditioned with hexane. $Ph_2O$ was eluted using hexane and then crude product fractions were eluted using $Et_2O$, collected and concentrated under reduced pressure to afford an orange oil (80 mg). Column chromatography on silica gel (0:1 to 1:9 $Et_2O$ in hexane) afforded a *trans*-**38a** (40 mg, 40%) and *cis*-**38b** (22 mg, 22%, compound started to decompose slowly after isolation to an unknown side product) as pale yellow oils. Data for *trans*-**38a**: $R_f$ = 0.65 (1:4 $Et_2O$ in hexane); $^1$H NMR (400 MHz, $CDCl_3$): δ = 7.35-7.27 (m, Ar*H*, 3H), 7.25-7.21 (m, Ar*H*, 2H), 5.96 (ddt, *J* = 5.7, 2.5 Hz, $^4J$ = 2.3 Hz, $CH_2CH$=C*H*, 1H), 5.79 (ddt, *J* = 5.7, 2.4 Hz, $^4J$ = 2.2 Hz, $CH_2$C*H*=CH, 1H), 4.17 (q, *J* = 7.2 Hz, OC$H_2$$CH_3$, 2H), 3.83 (dt, *J* = 9.2, 6.6 Hz, ArC*H*, 1H), 3.67 (dddd, *J* = 9.2, 2.5 Hz, $^4J$ = 2.5, 2.2 Hz, *H*$CCO_2$Et, 1H), 3.00 (ddddd, $^2J$ = 16.9 Hz, *J* = 9.2, 2.4 Hz, $^4J$ = 2.6, 2.3 Hz, -C$H_a$$H_b$-, 1H), 2.56 (ddddd, $^2J$ = 16.9 Hz, *J* = 6.6, 2.4 Hz, $^4J$ = 2.6, 2.3 Hz, -C$H_a$$H_b$-, 1H), 1.27 ppm (t, *J* = 7.2 Hz, 3H); $^{13}$C NMR (100 MHz, $CDCl_3$): δ = 173.6, 145.0, 132.2, 128.1, 127.7, 126.6, 125.9, 60.2, 59.0, 45.8, 41.1, 13.7 ppm; $\bar{\nu}$/(film) = 3060, 3029, 2980, 2932, 2906, 2854, 1731, 1495, 1457, 1245, 1176 cm$^{-1}$; MS (CI): *m/z* (%): 245 (2) $[M+C_2H_5]^+$, 217 (8) $[M+H]^+$, 171 (19) $[M-OEt]^+$, 143 (100) $[M-CO_2Et]^+$, HRMS (APCI): calcd for $C_{14}H_{17}O_2$, 217.1228 $[M+H]^+$ found 217.1229; $t_R$ (GC) = 12.44 minutes. Data *cis*-**38b**: $R_f$ = 0.52 (1:4 $Et_2O$ in hexane); $^1$H NMR (400 MHz, $CDCl_3$): δ = 7.27-7.28 (m, Ar*H*, 4H), 7.22-7.17 (m, Ar*H*, 1H), 6.15 (ddt, *J* = 5.7, 2.2 Hz, $^4J$ = 2.1 Hz, $CH_2CH$=C*H*, 1H), 5.85 (ddt, *J* = 5.7, 2.3 Hz, $^4J$ = 1.9 Hz, $CH_2$C*H*=CH, 1H), 3.93 (dddt, *J* = 9.1, 2.1 Hz, $^4J$ = 2.0, 1.9 Hz, *H*$CCO_2$Et, 1H), 3.85 (dt, *J* = 9.1, 7.2 Hz, ArC*H*, 1H), 3.78-3.61 (m, OC$H_a$$H_b$$CH_3$, 2H); 2.83 (dddd, *J* = 7.2, 2.3 Hz, $^4J$ = 2.2, 1.9 Hz, CH-C$H_2$-CH=CH-, 2H), 0.85 ppm (t, *J* = 7.0 Hz, $CH_2CH_3$, 3H); $^{13}$C NMR (100 MHz, $CDCl_3$): δ = 172.2, 141.5, 133.8, 128.0, 127.5, 127.4, 126.0, 59.6, 55.9, 46.3, 38.4, 13.2 ppm; $\bar{\nu}$/(film) = 3060, 3029, 2980, 2932, 2904, 1729, 1495, 1457, 1370, 1178 cm$^{-1}$; MS (CI): *m/z* (%): 245 (5) $[M+C_2H_5]^+$, 217 (14) $[M+H]^+$, 171 (20) $[M-OEt]^+$, 143 (100) $[M-CO_2Et]^+$; HRMS (TOF): calcd for $C_{14}H_{17}O_2$, 216.1150 [M] found 216.1155; $t_R$ (GC) = 12.28 minutes.

## Characterisation Spectra

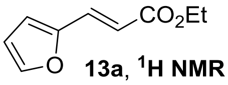


**13a**, $^{1}$H NMR

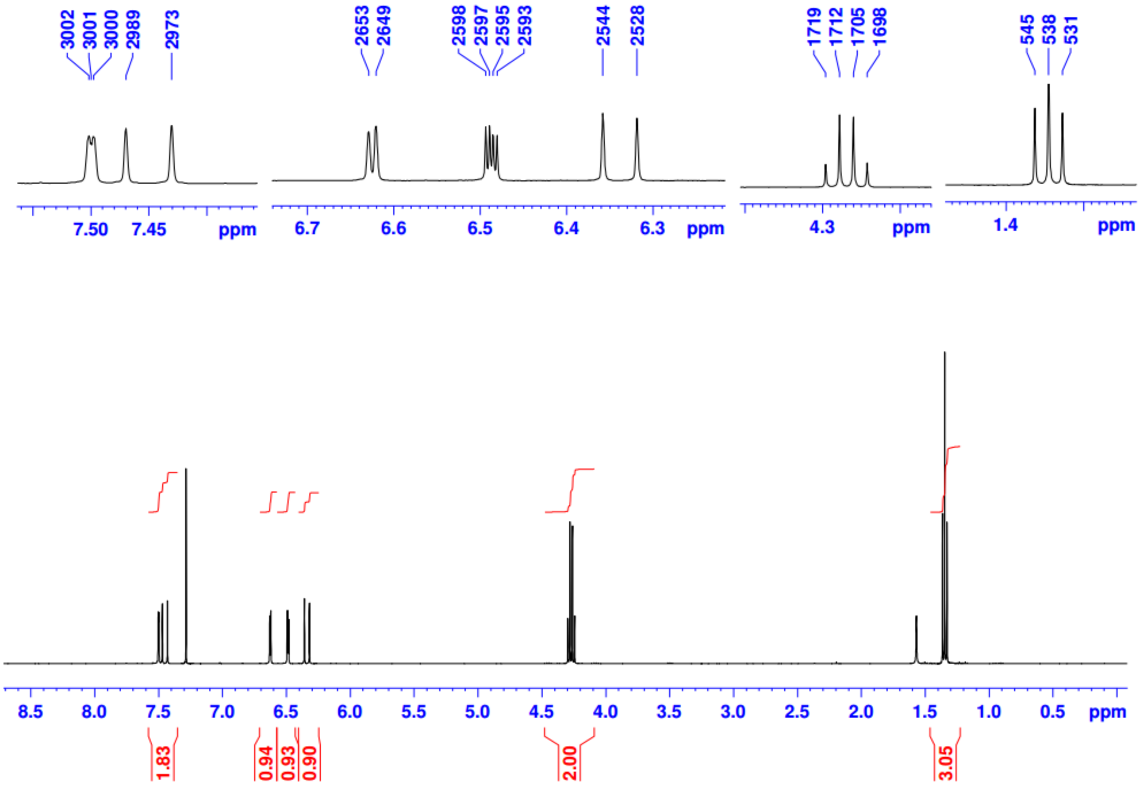


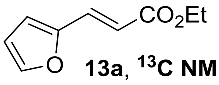


**13a**, $^{13}$C NMR

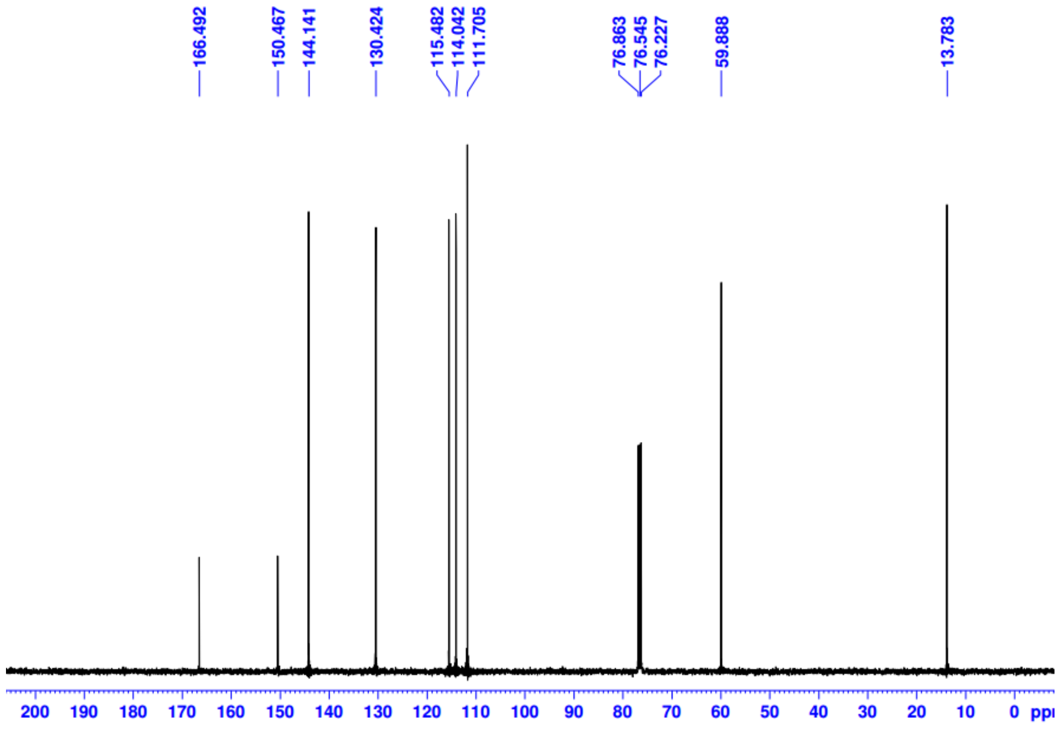

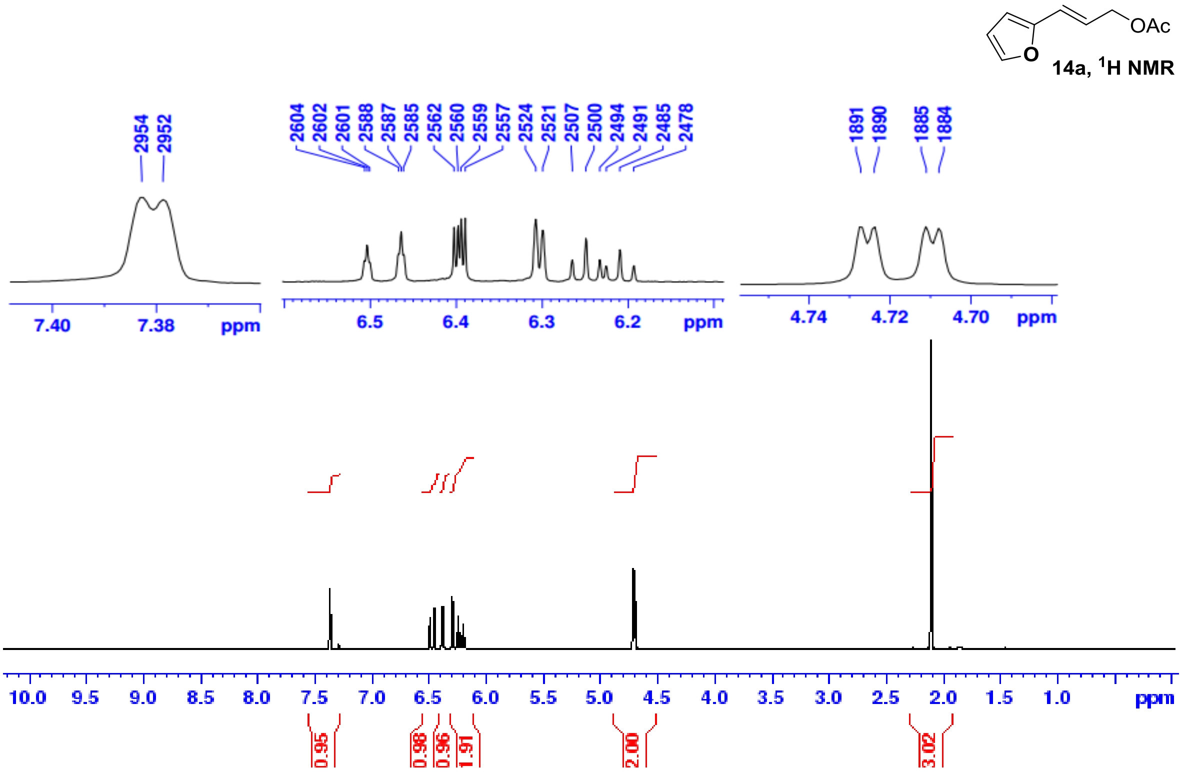
OAc
O
14a, ¹H NMR
ppm
0.95
0.98
0.96
1.91
2.00
3.02

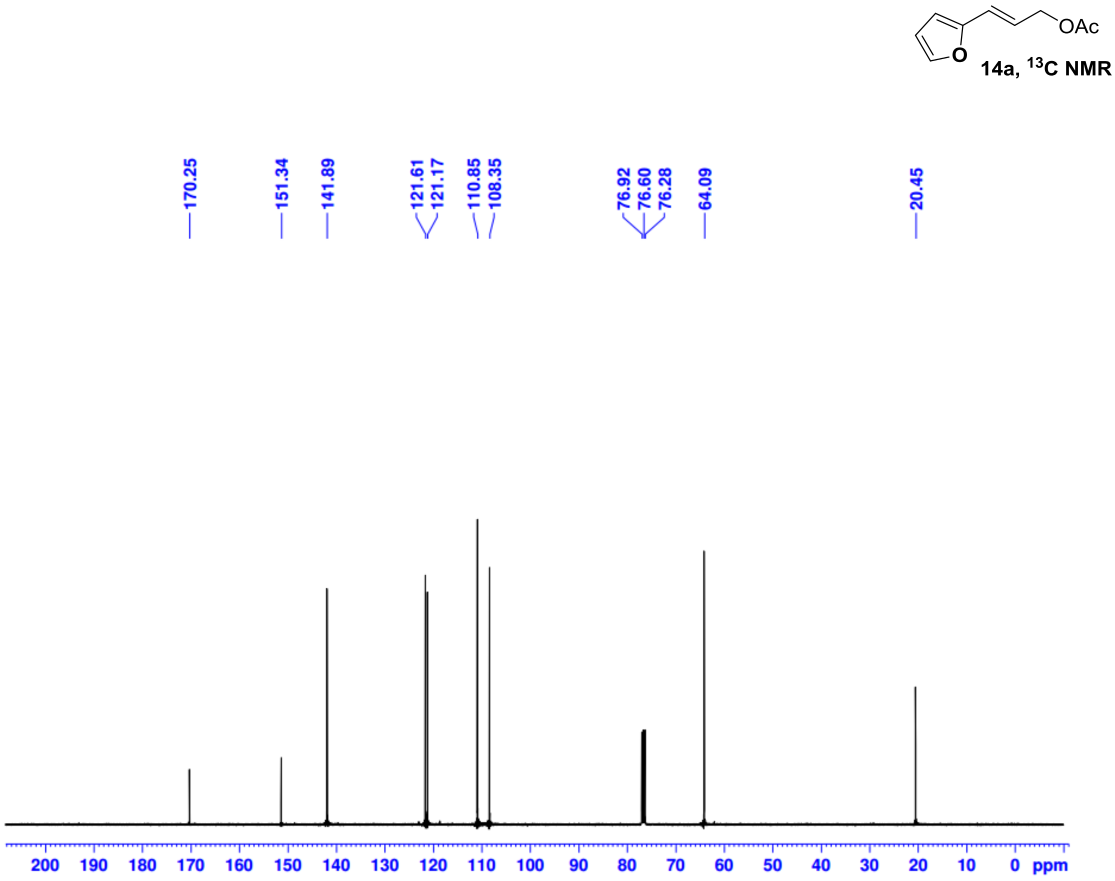
OAc
O
14a, ¹³C NMR
170.25
151.34
141.89
121.61
121.17
110.85
108.35
76.92
76.60
76.28
64.09
20.45
ppm

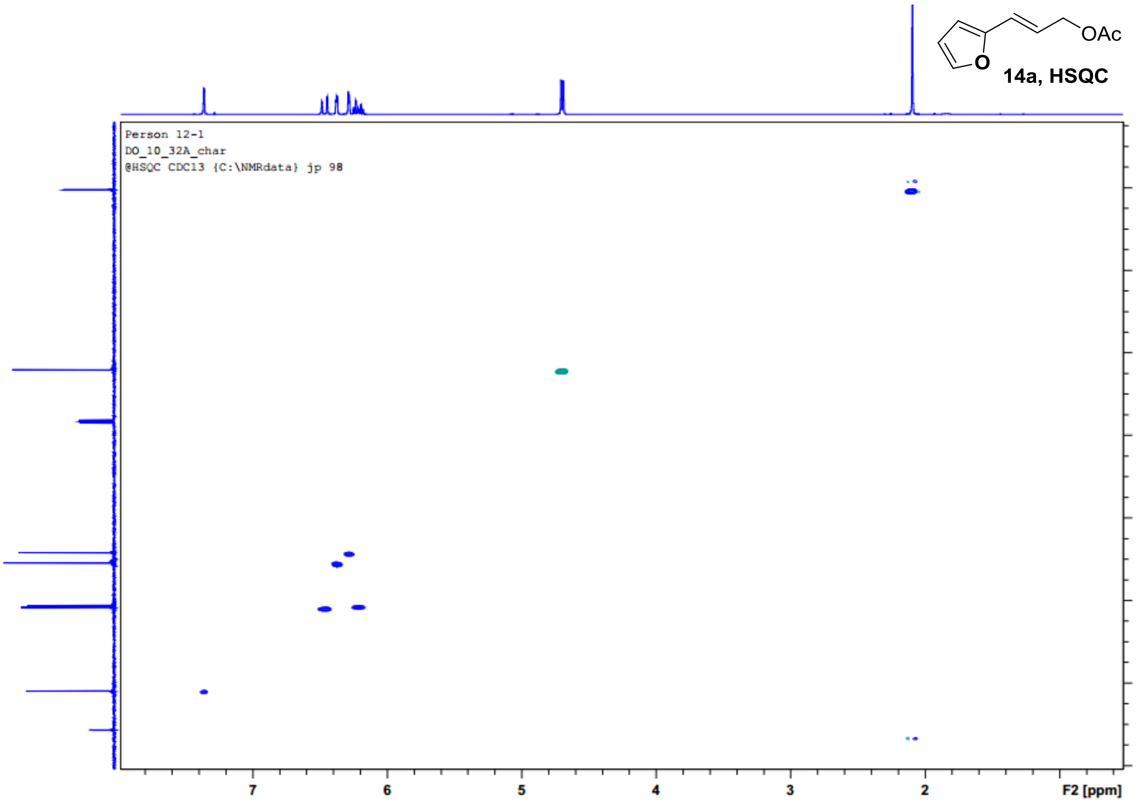
OAc
O
14a, HSQC
Person 12-1
DO_10_32A_char
@HSQC CDCl3 {C:\NMRdata} jp 98
F1 [ppm]
40
60
80
100
120
140
7
6
5
4
3
2
F2 [ppm]

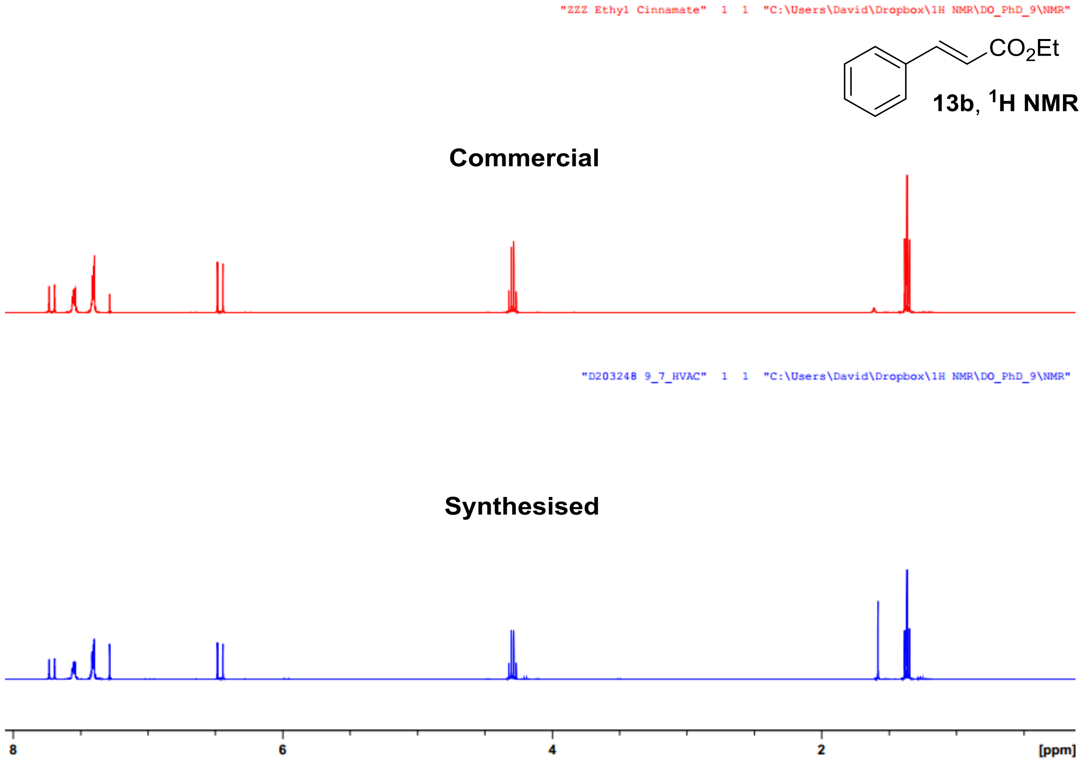
"ZZZ Ethyl Cinnamate" 1 1 "C:\Users\David\Dropbox\1H NMR\DO_PhD_9\NMR"
$CO_2Et$
13b, $^{1}$H NMR
Commercial
"D203248 9_7_HVAC" 1 1 "C:\Users\David\Dropbox\1H NMR\DO_PhD_9\NMR"
Synthesised
8
6
4
2
[ppm]

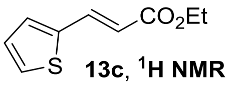

**13c**, $^{1}$H NMR

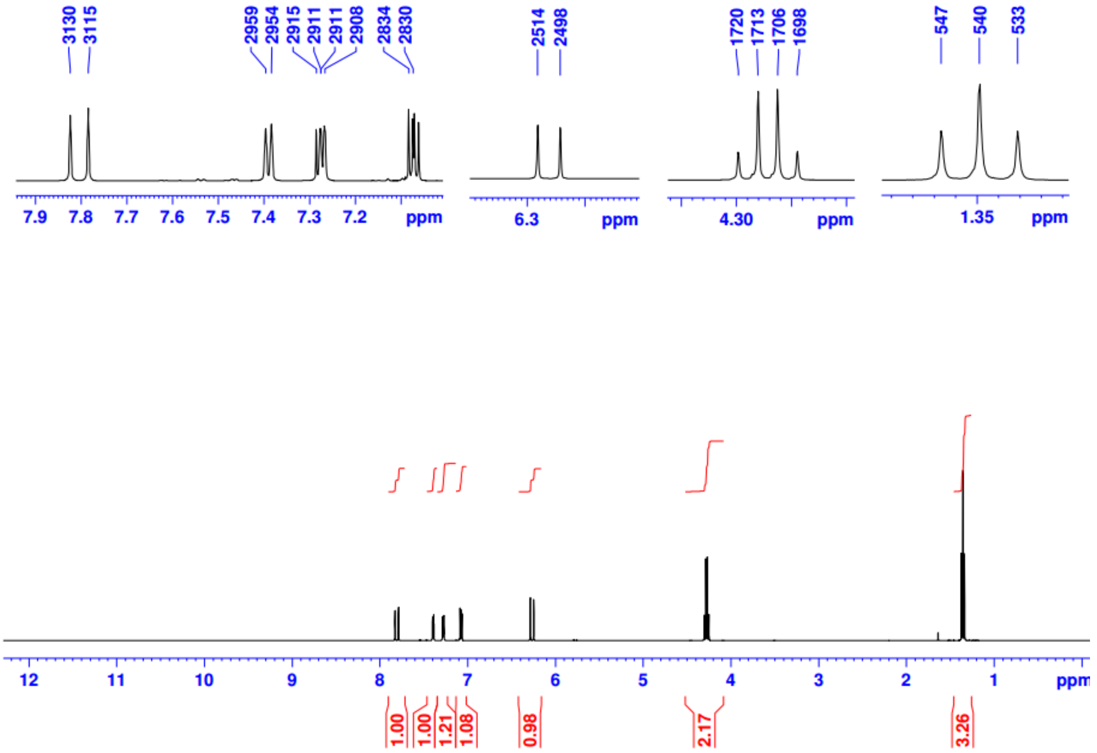
3130
3115
2959
2954
2915
2911
2911
2908
2834
2830
2514
2498
1720
1713
1706
1698
547
540
533
7.9 7.8 7.7 7.6 7.5 7.4 7.3 7.2 ppm
6.3 ppm
4.30 ppm
1.35 ppm
12 11 10 9 8 7 6 5 4 3 2 1 ppm
1.00
1.00
1.21
1.08
0.98
2.17
3.26

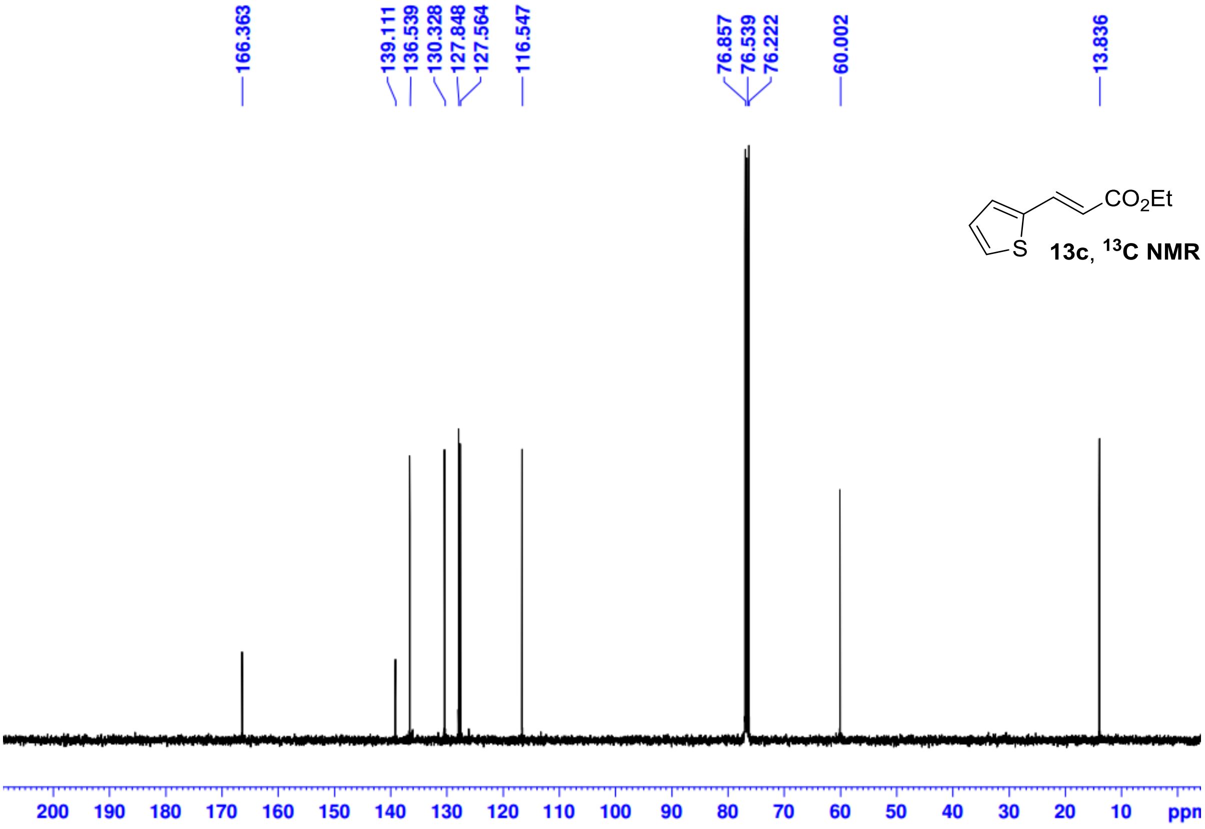
166.363
139.111
136.539
130.328
127.848
127.564
116.547
76.857
76.539
76.222
60.002
13.836
CO2Et
S
13c, 13C NMR
200 190 180 170 160 150 140 130 120 110 100 90 80 70 60 50 40 30 20 10 ppm

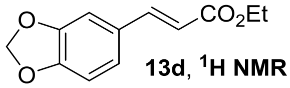


**13d**, **$^{1}$H NMR**

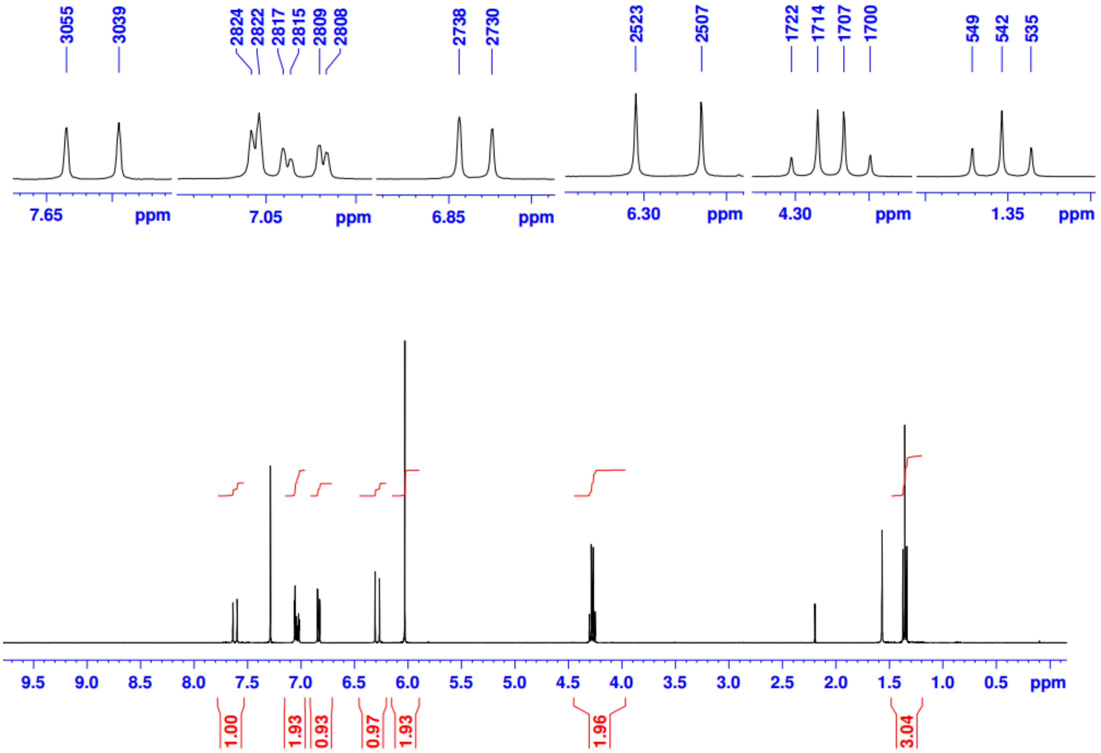


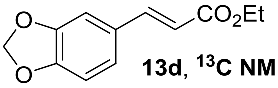


**13d**, **$^{13}$C NMR**

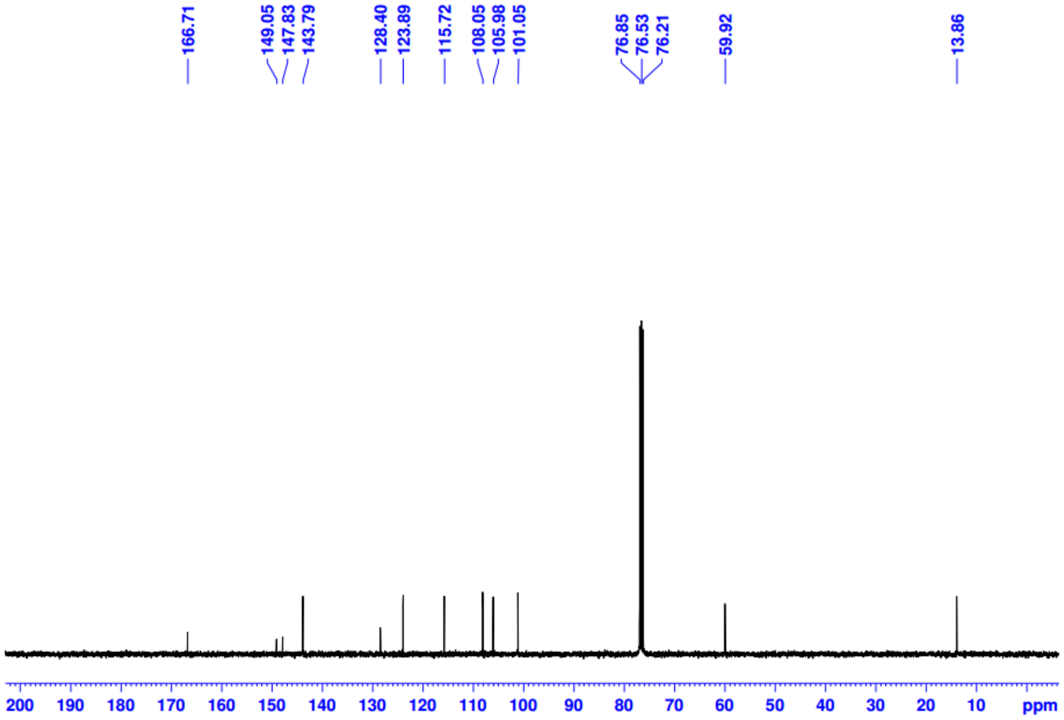

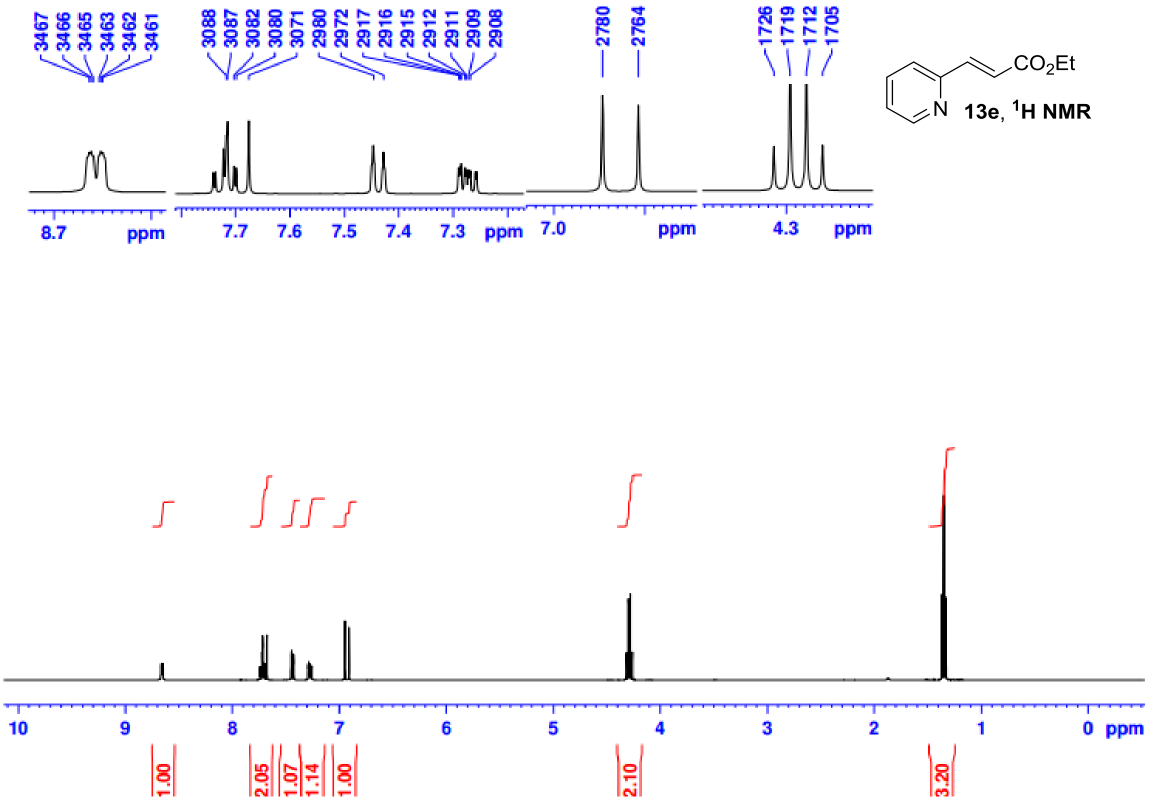


13e, $^{1}H$ NMR

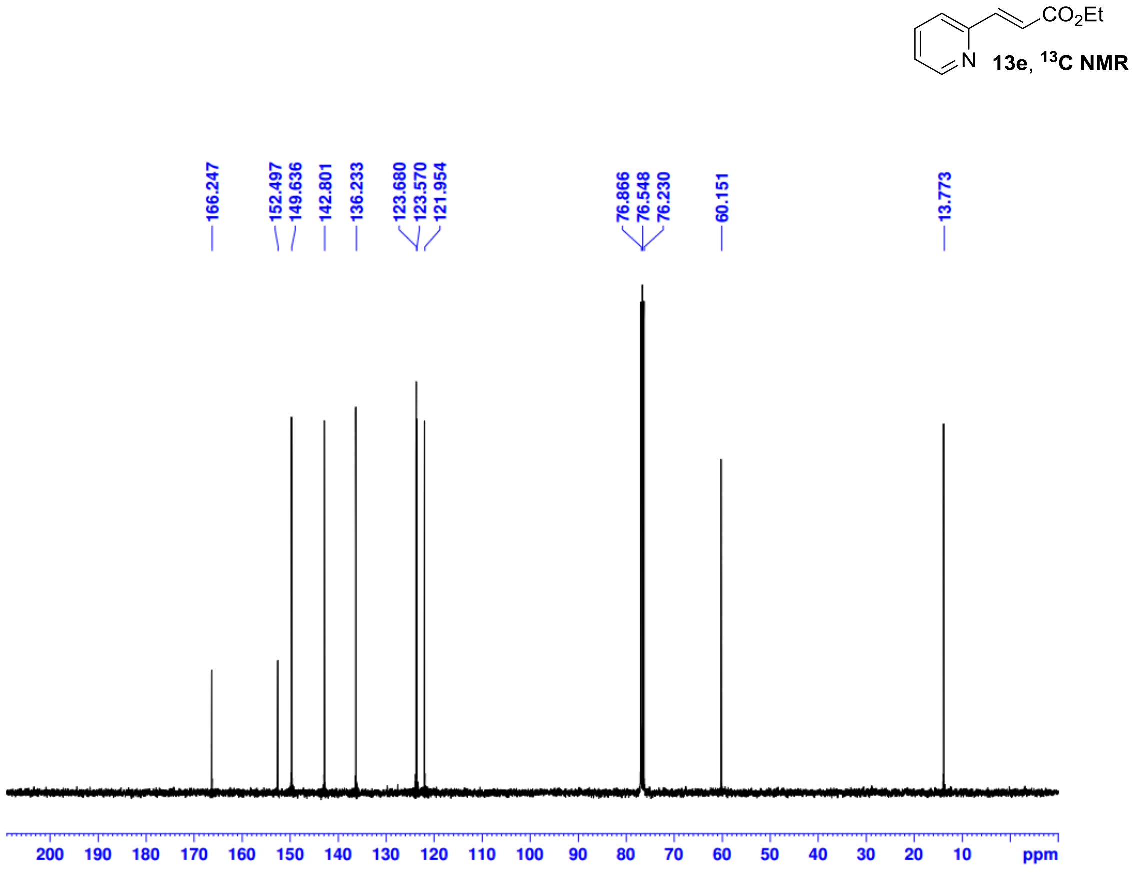


13e, $^{13}C$ NMR

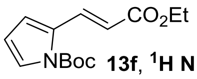


**13f**, $^{1}$**H NMR**

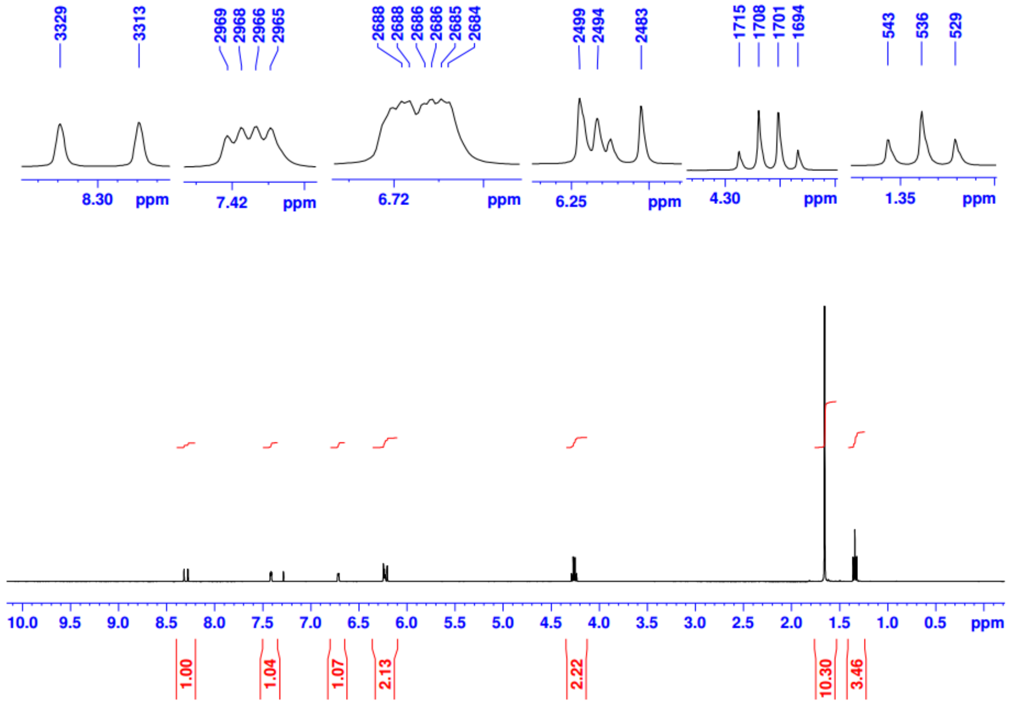


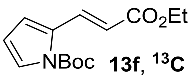


**13f**, $^{13}$**C NMR**

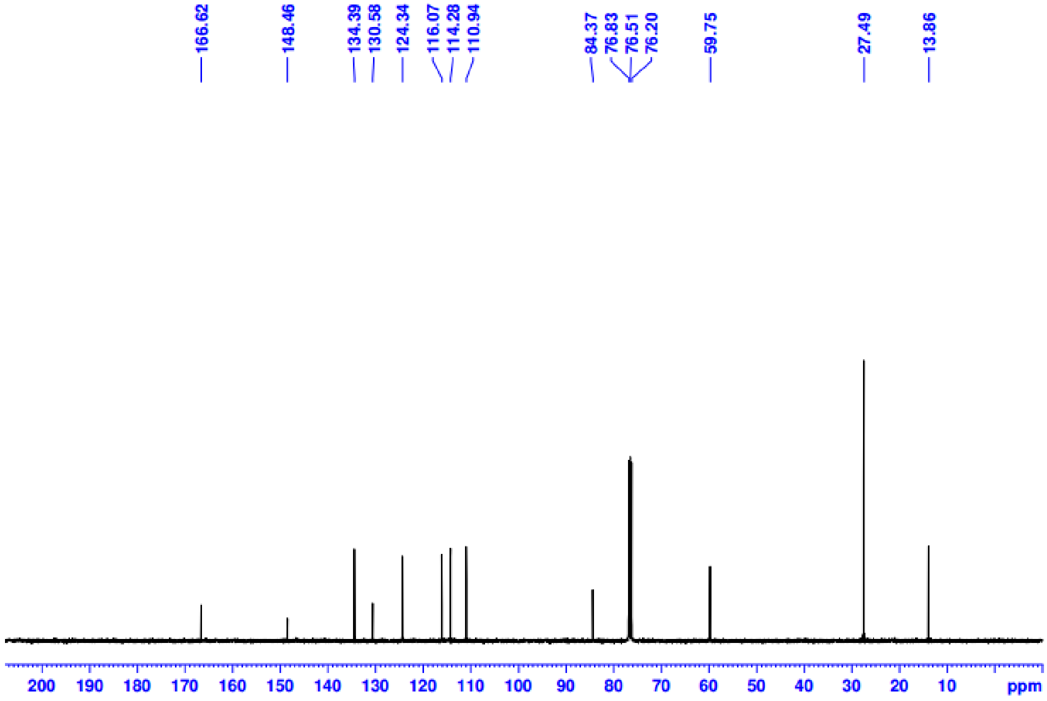

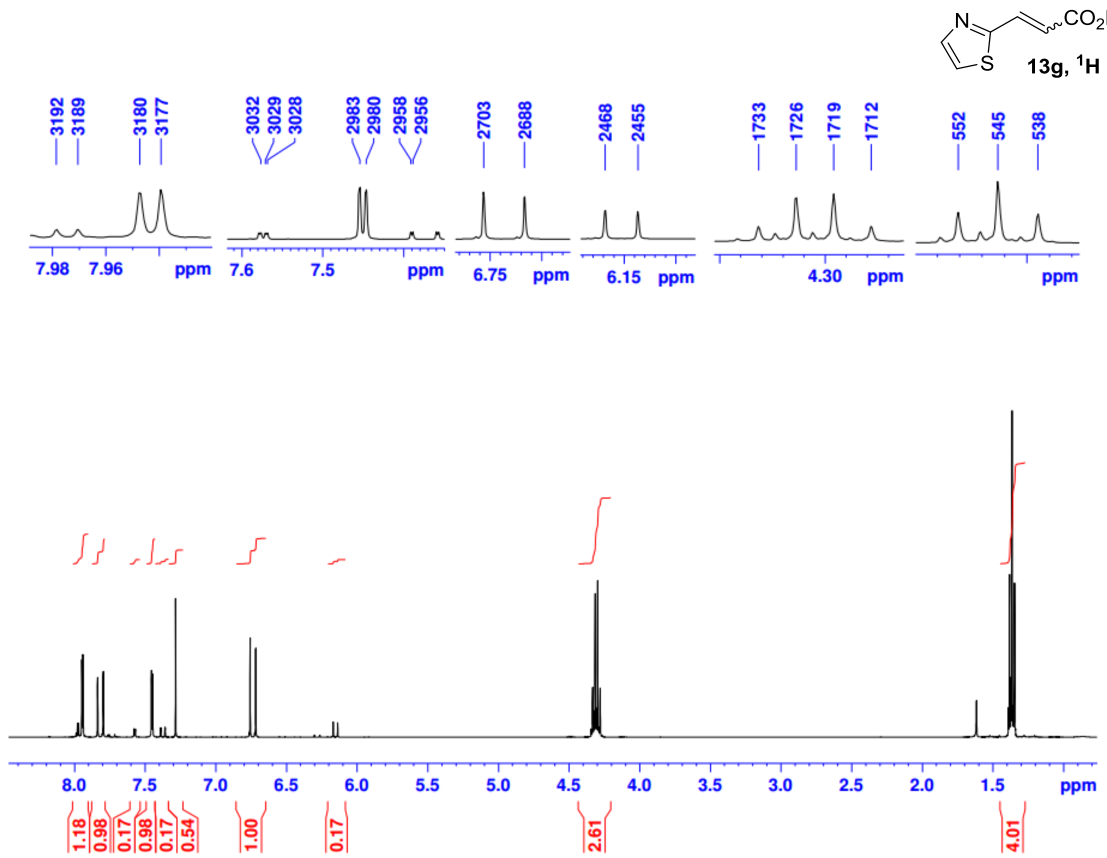


13g, $^1$H NMR

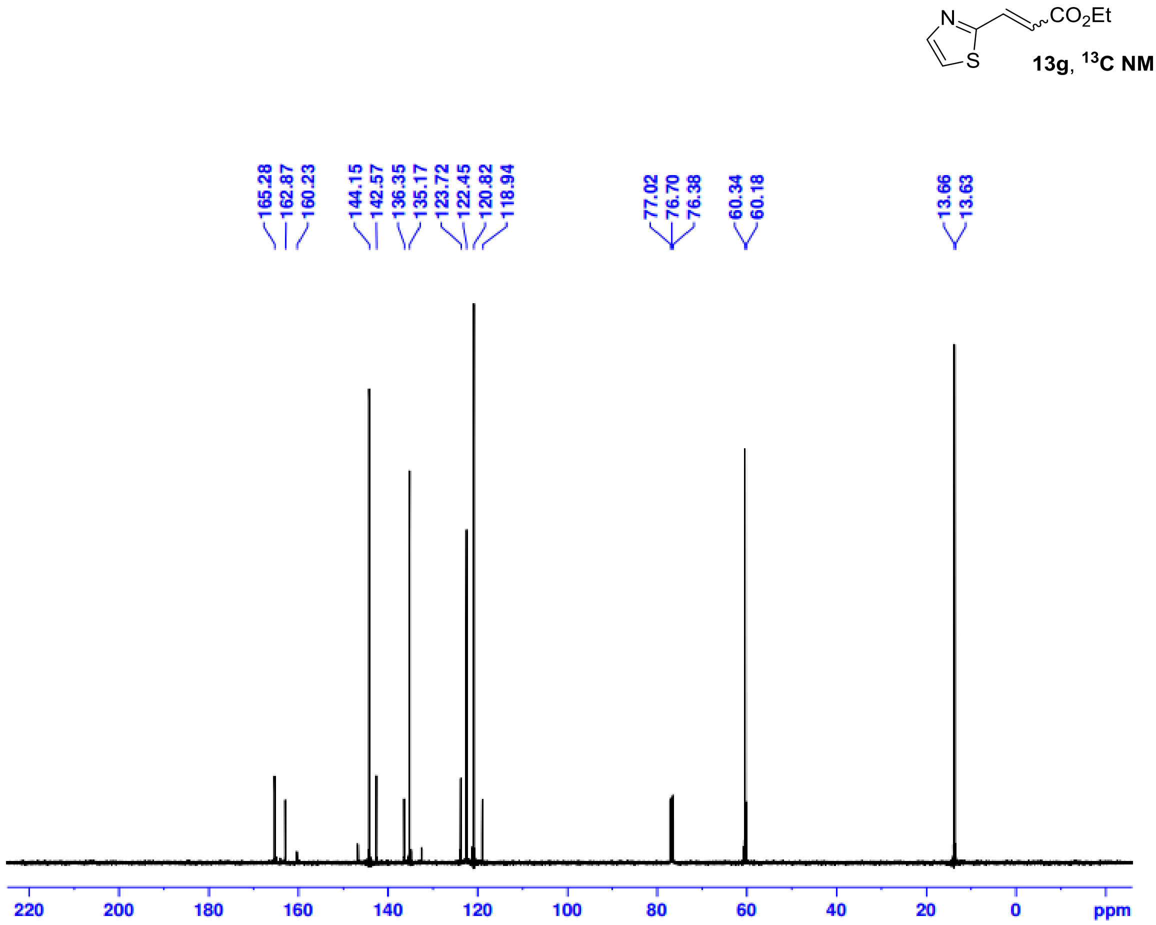


13g, $^{13}$C NMR

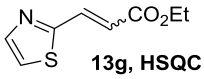

**13g, HSQC**

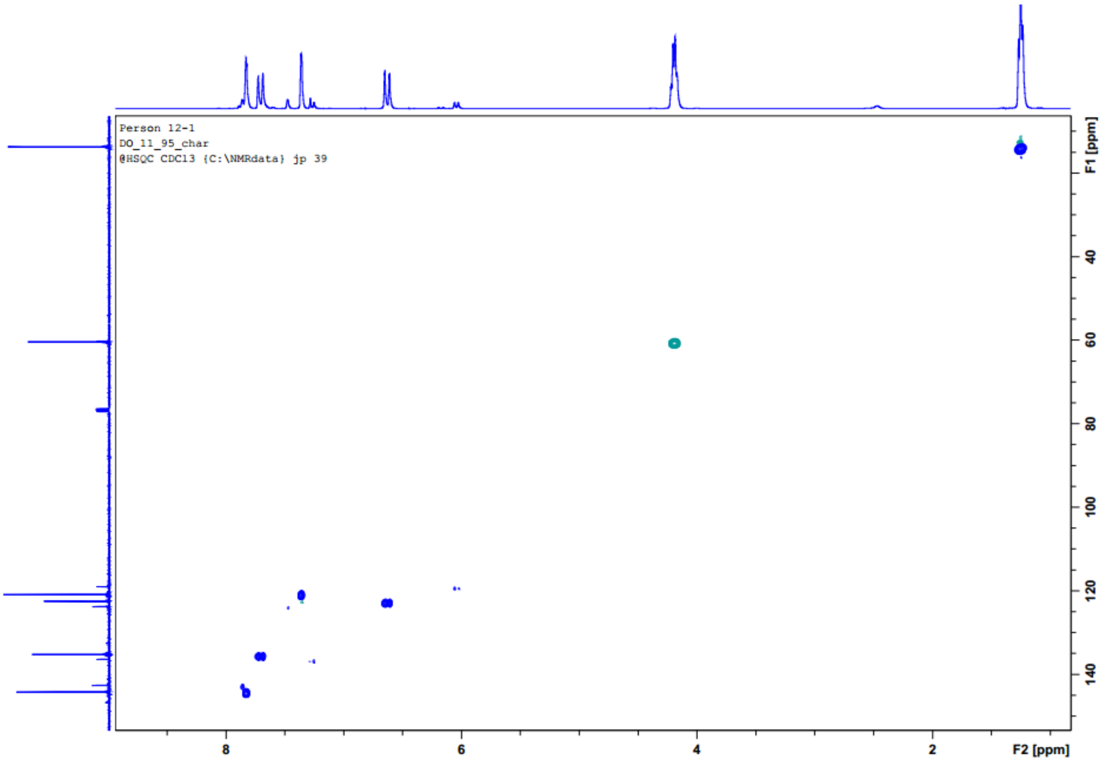


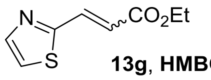

**13g, HMBC**

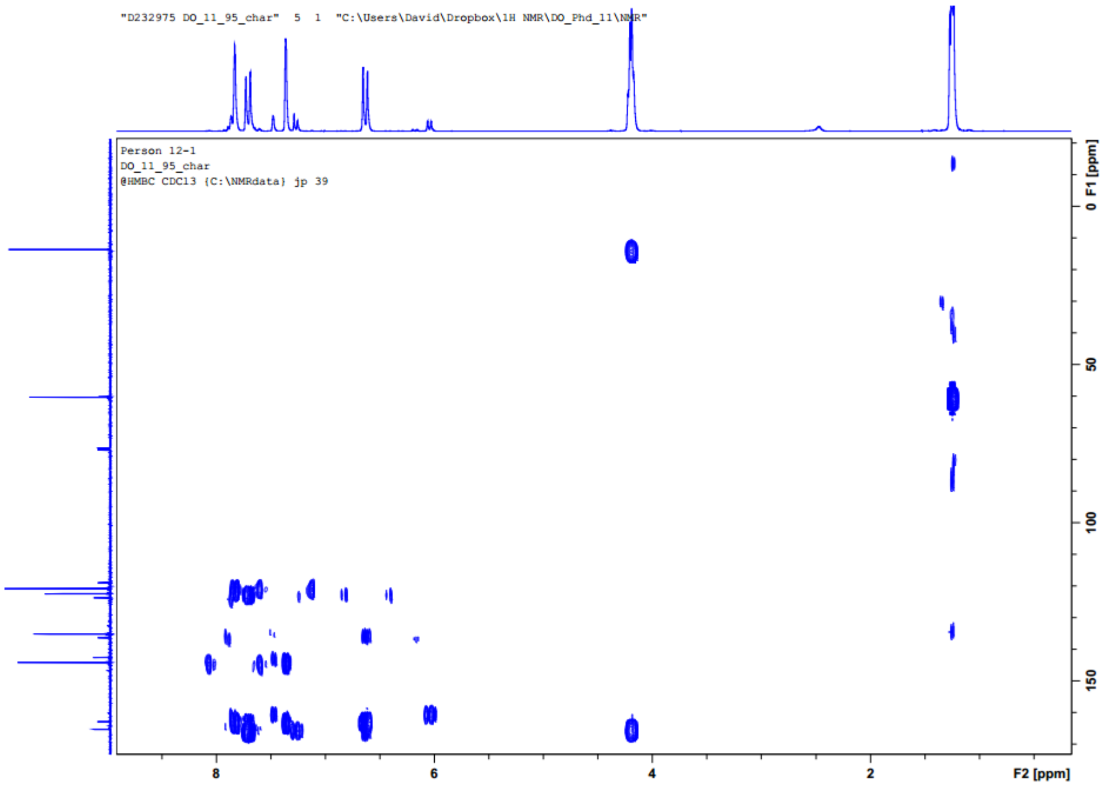

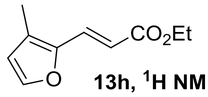


**13h, $^{1}H$ NMR**

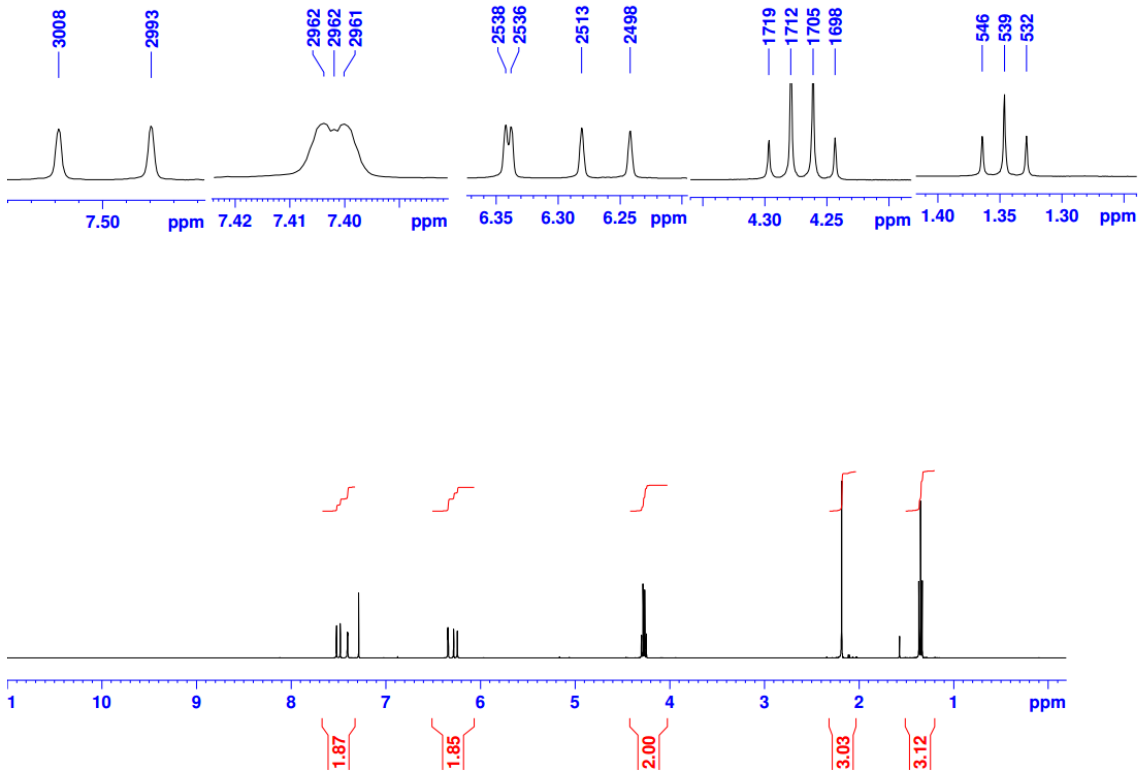


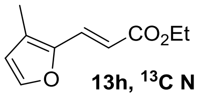


**13h, $^{13}C$ NMR**

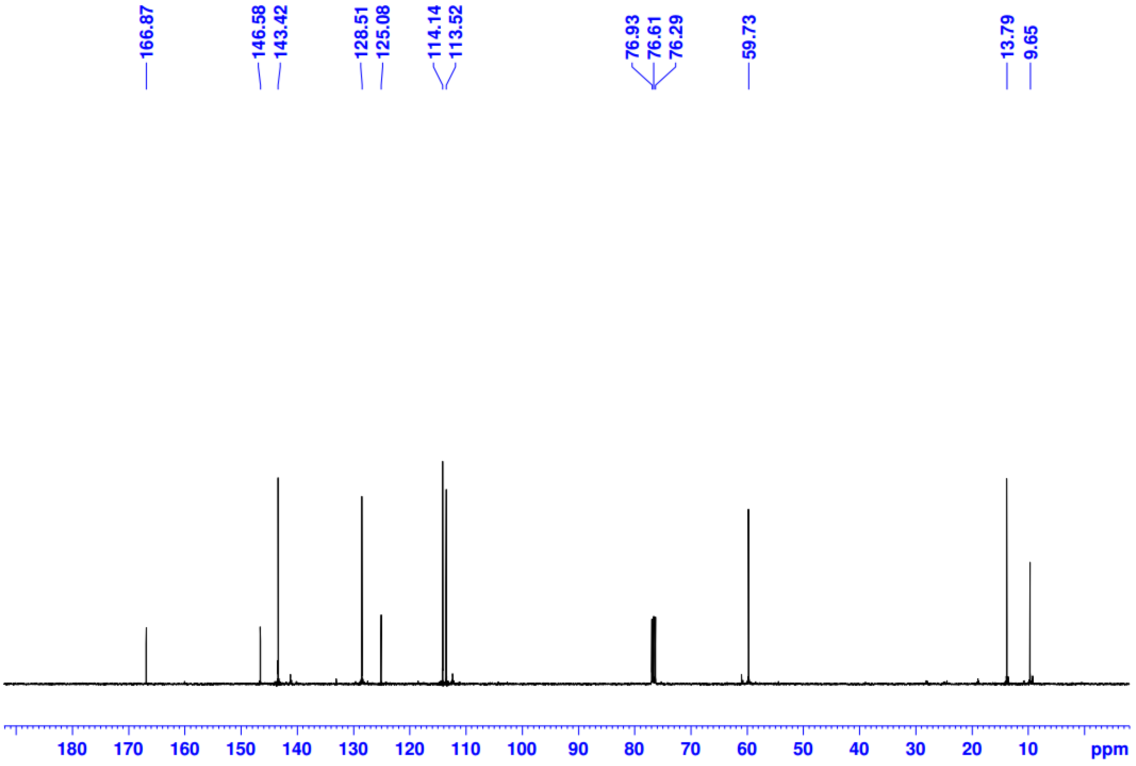

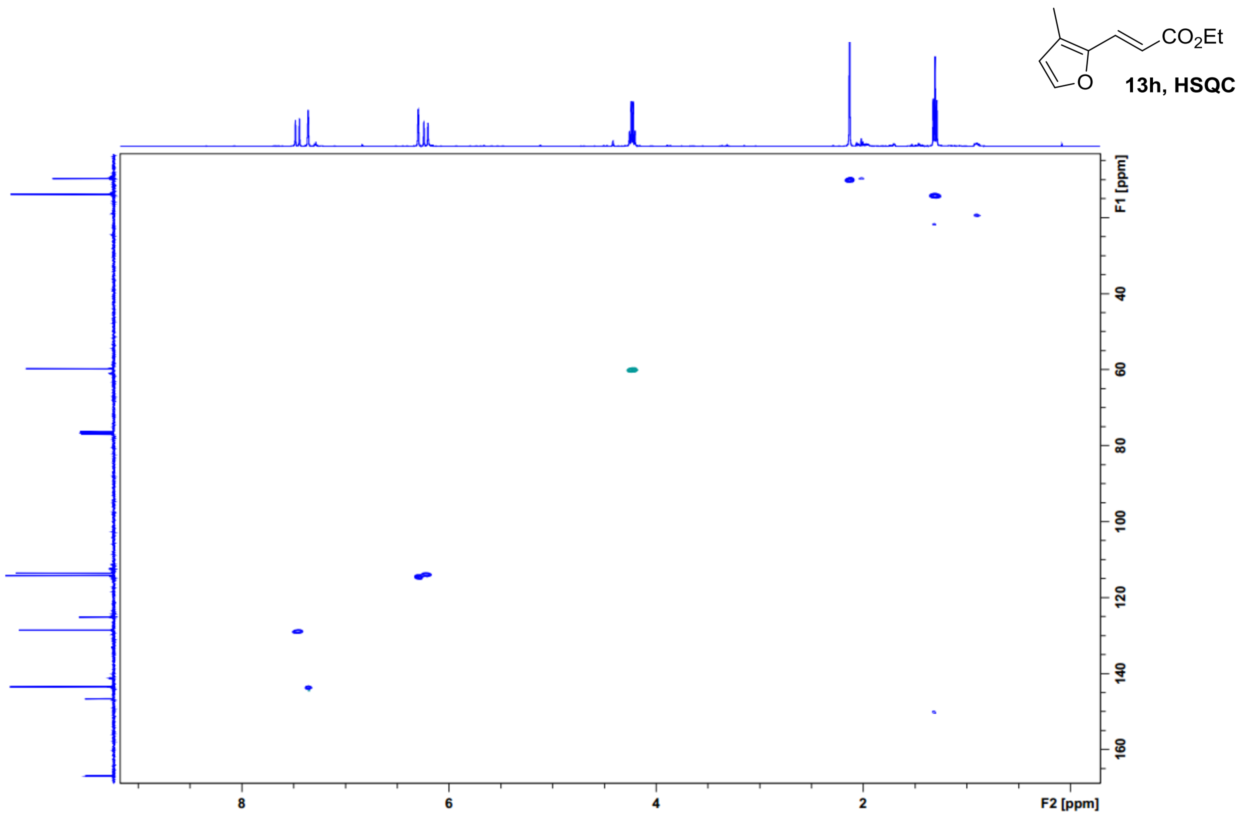
CO2Et
O
13h, HSQC
F1 [ppm]
40
60
80
100
120
140
160
8
6
4
2
F2 [ppm]

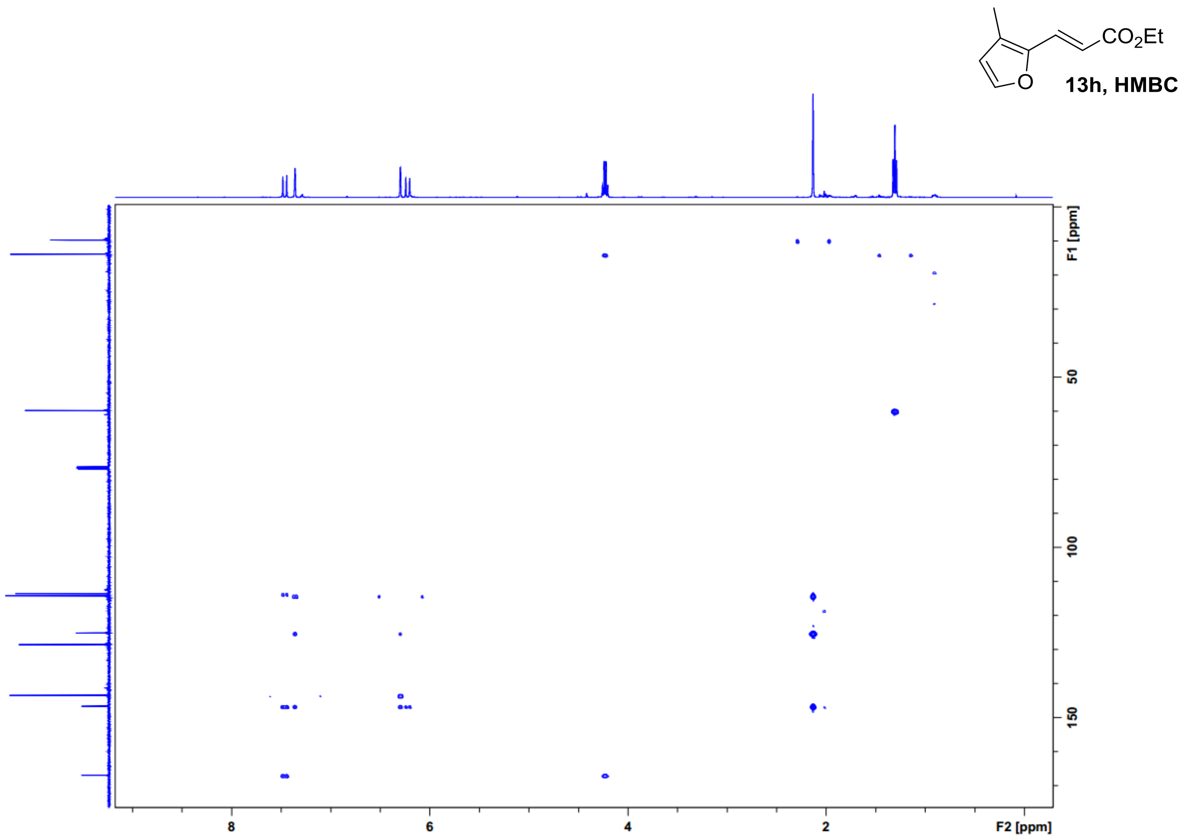
CO2Et
O
13h, HMBC
F1 [ppm]
50
100
150
8
6
4
2
F2 [ppm]

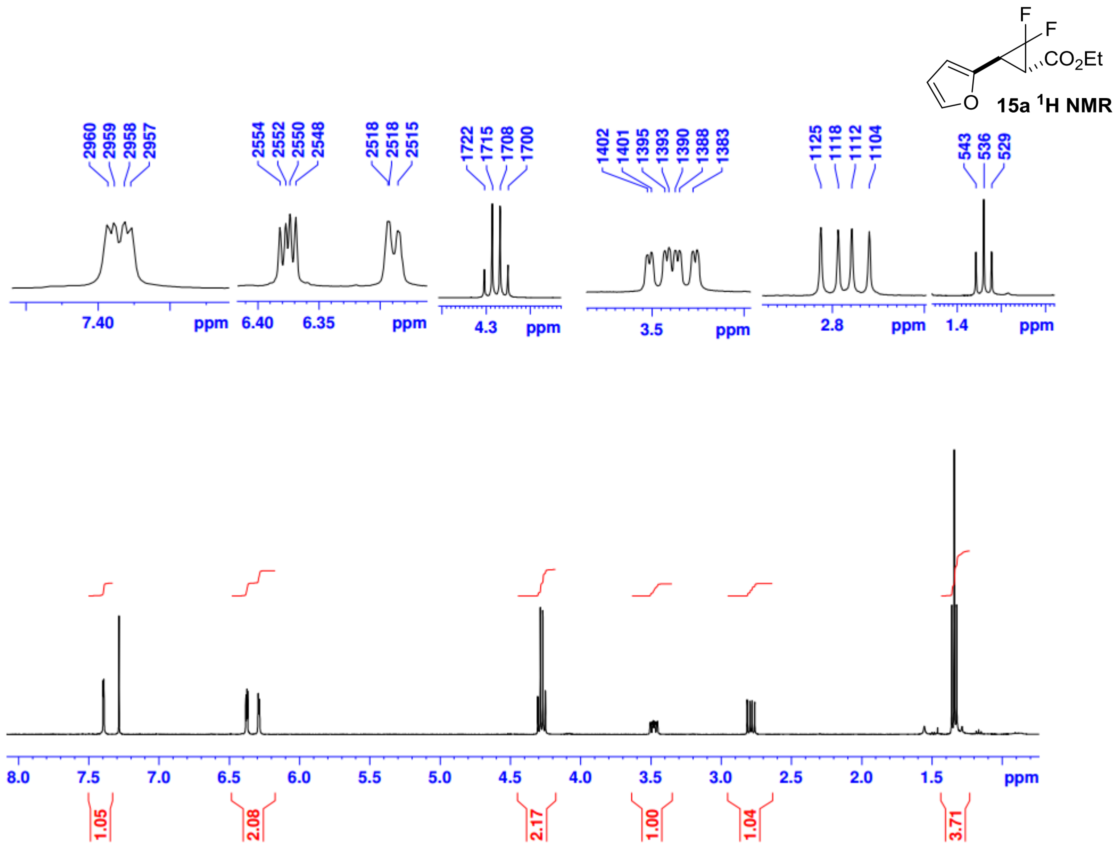
F
F
CO2Et
O
15a 1H NMR

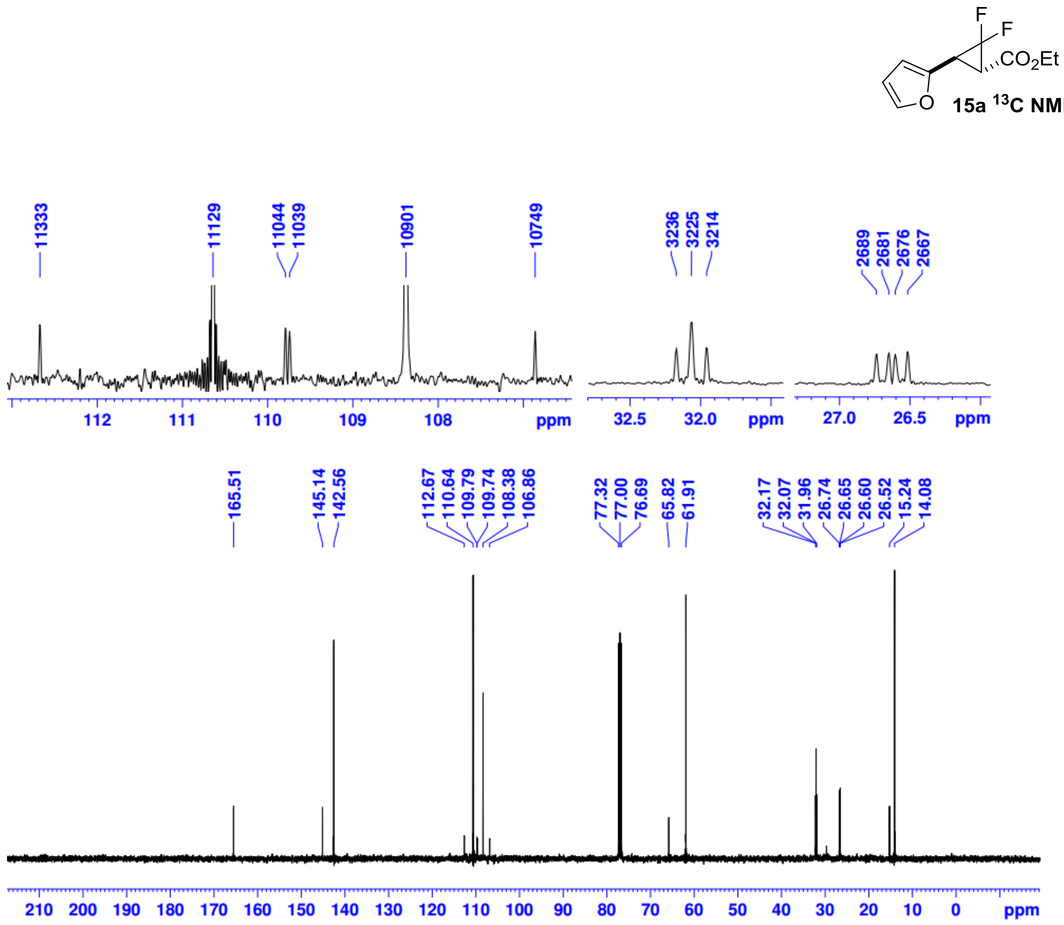
F
F
CO2Et
O
15a 13C NMR

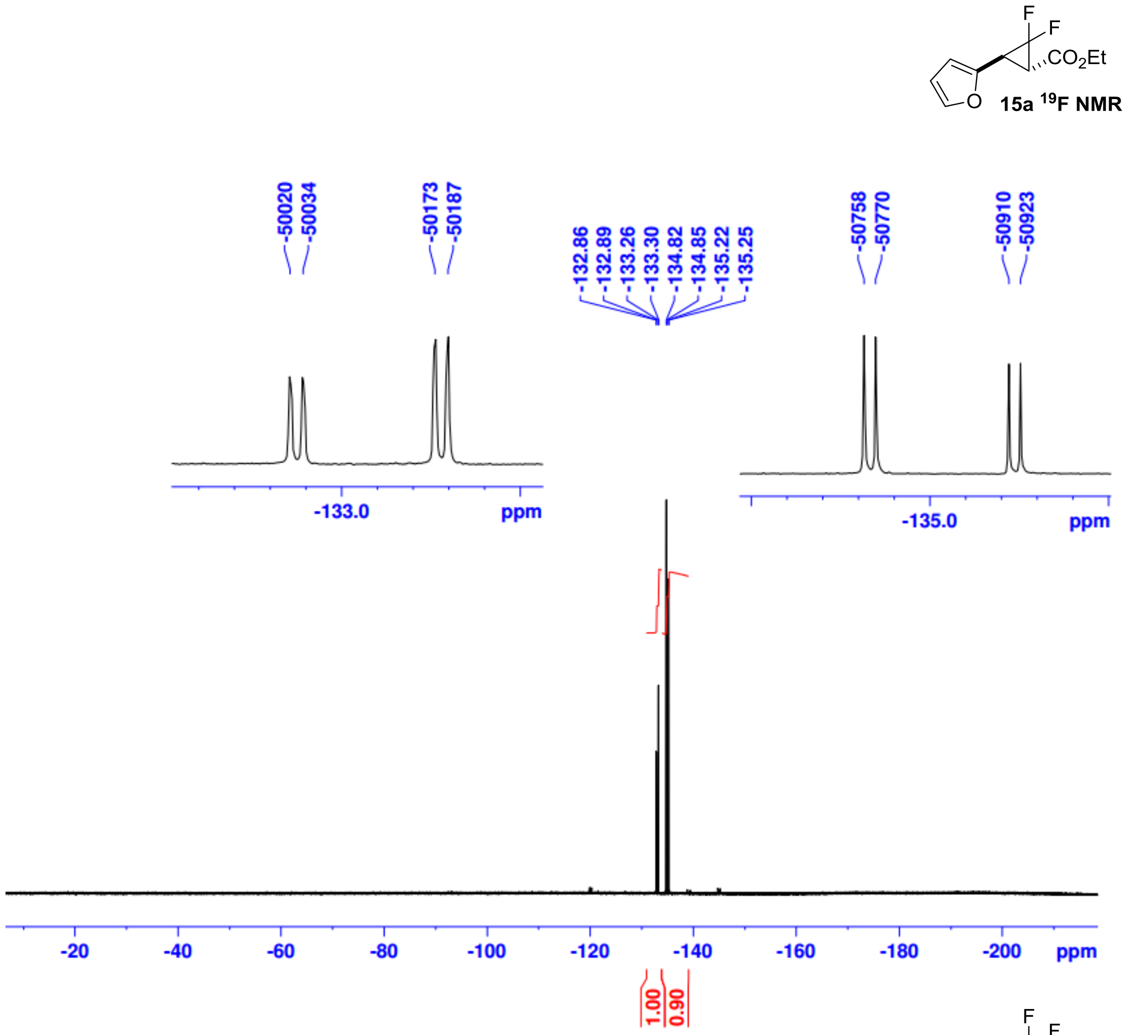
15a $^{19}$F NMR
-50020
-50034
-50173
-50187
-132.86
-132.89
-133.26
-133.30
-134.82
-134.85
-135.22
-135.25
-50758
-50770
-50910
-50923
-133.0
ppm
-135.0
ppm
-20
-40
-60
-80
-100
-120
-140
-160
-180
-200
ppm
1.00
0.90

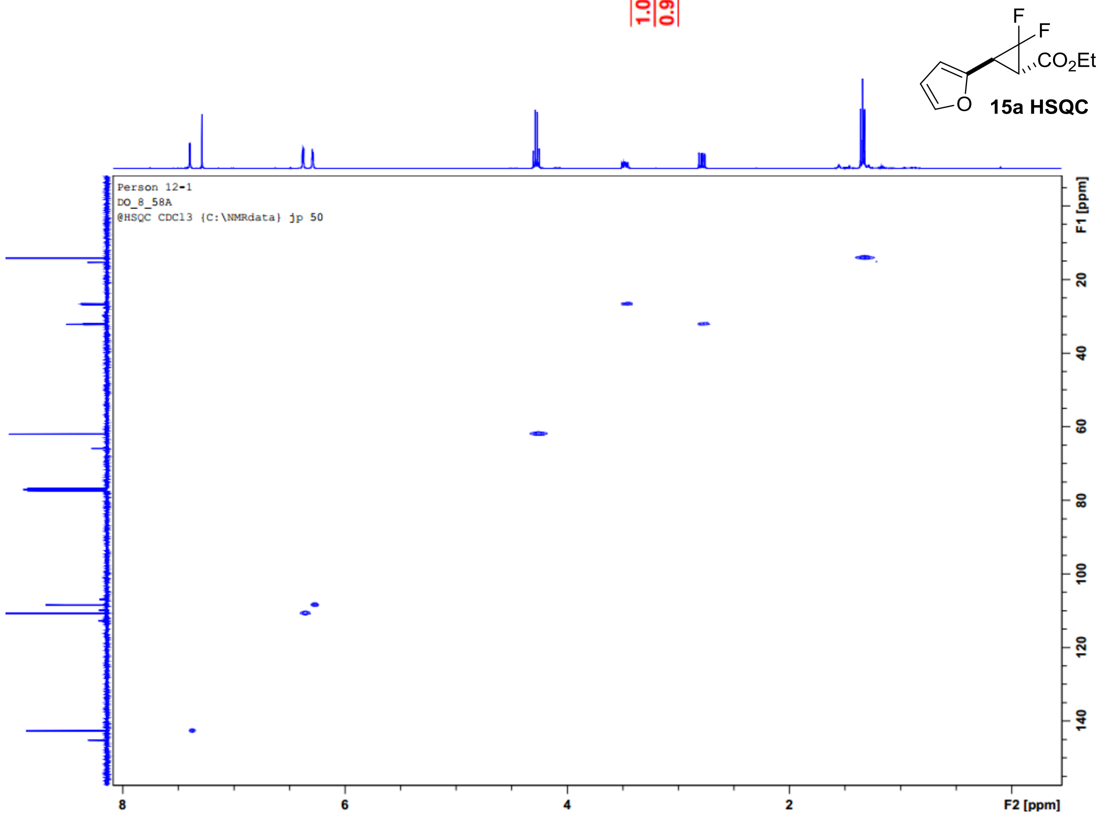
15a HSQC
Person 12-1
DO_8_58A
@HSQC CDCl3 {C:\NMRdata} jp 50
F1 [ppm]
F2 [ppm]

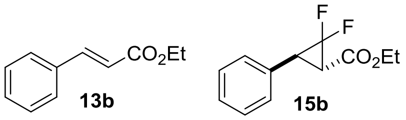

**13b** **15b**

**$^{1}$H crude spectra from incomplete difluorocyclopropanation of 13b.**

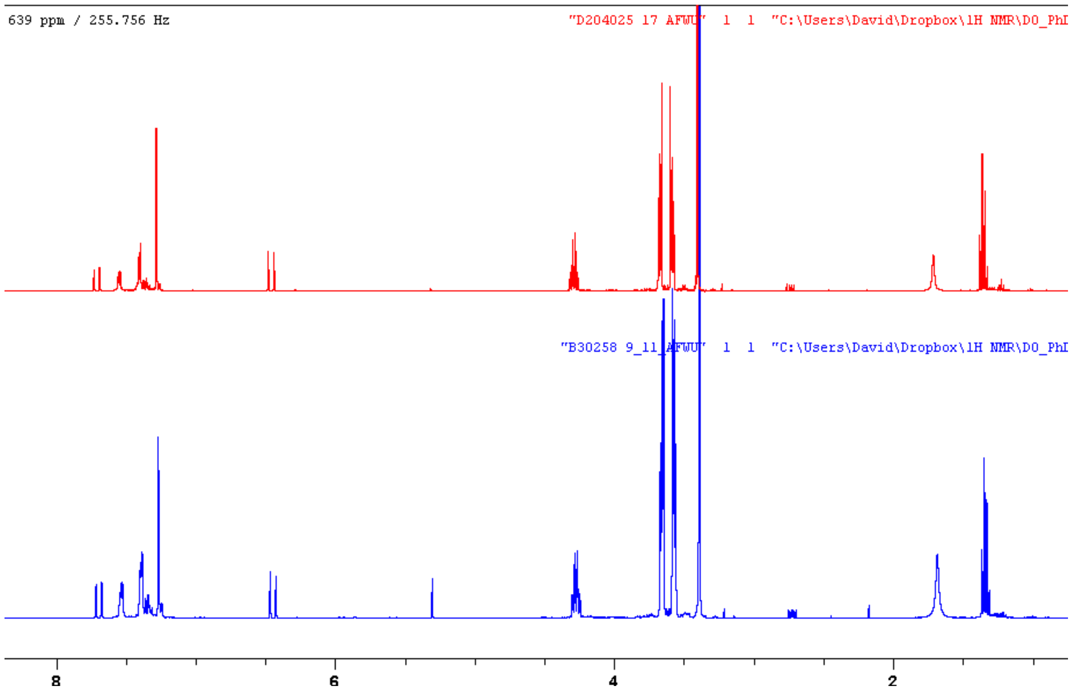


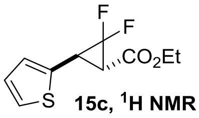

**15c, $^{1}$H NMR**

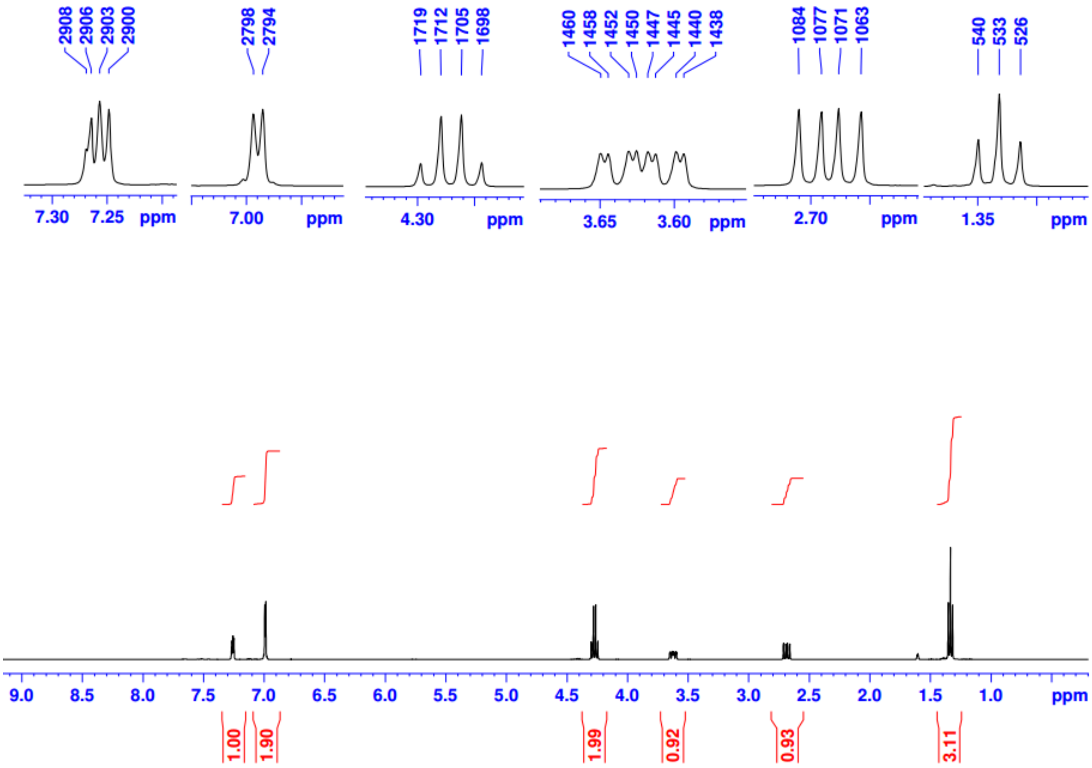

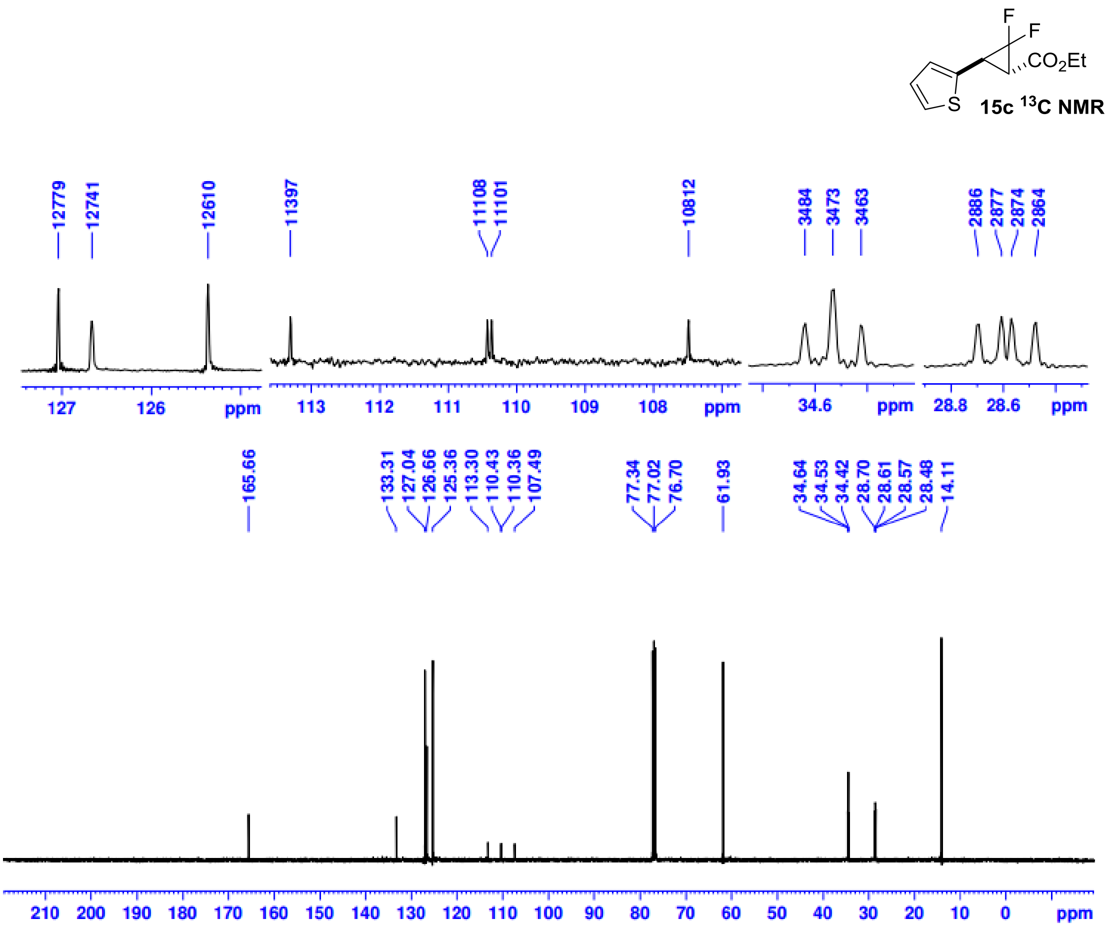
15c ¹³C NMR

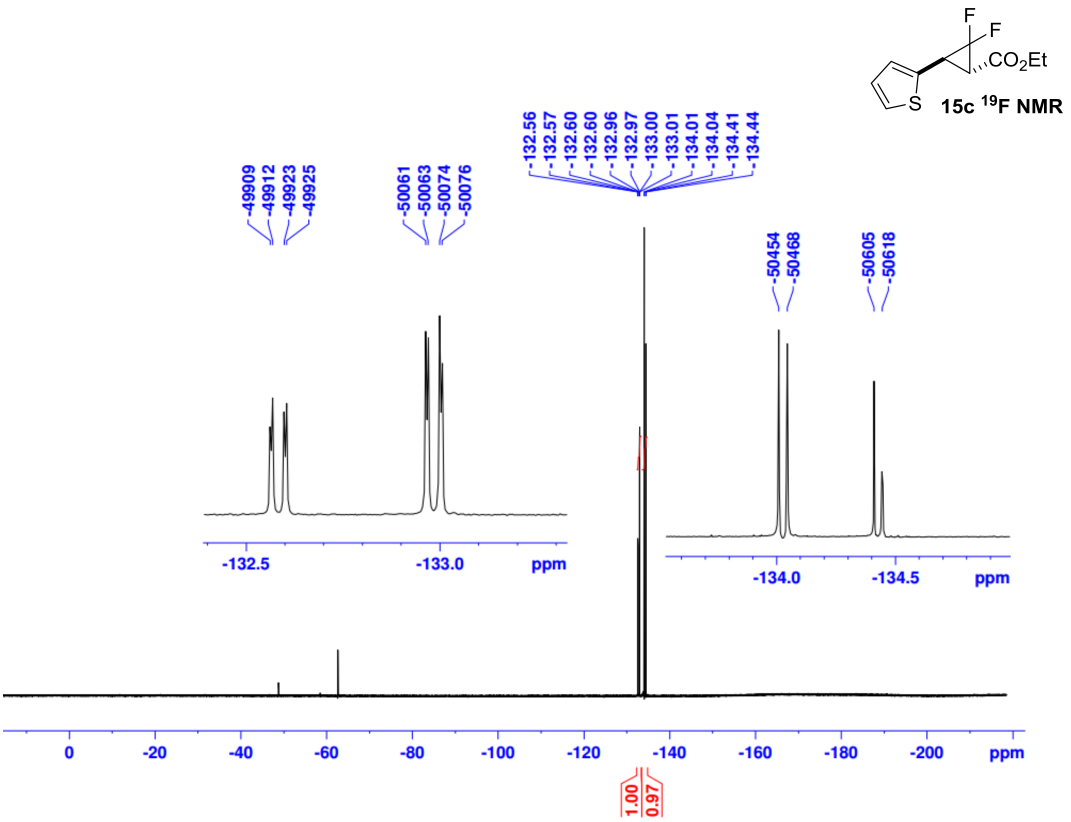
15c ¹⁹F NMR

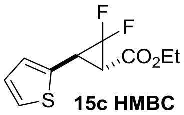


**15c HMBC**

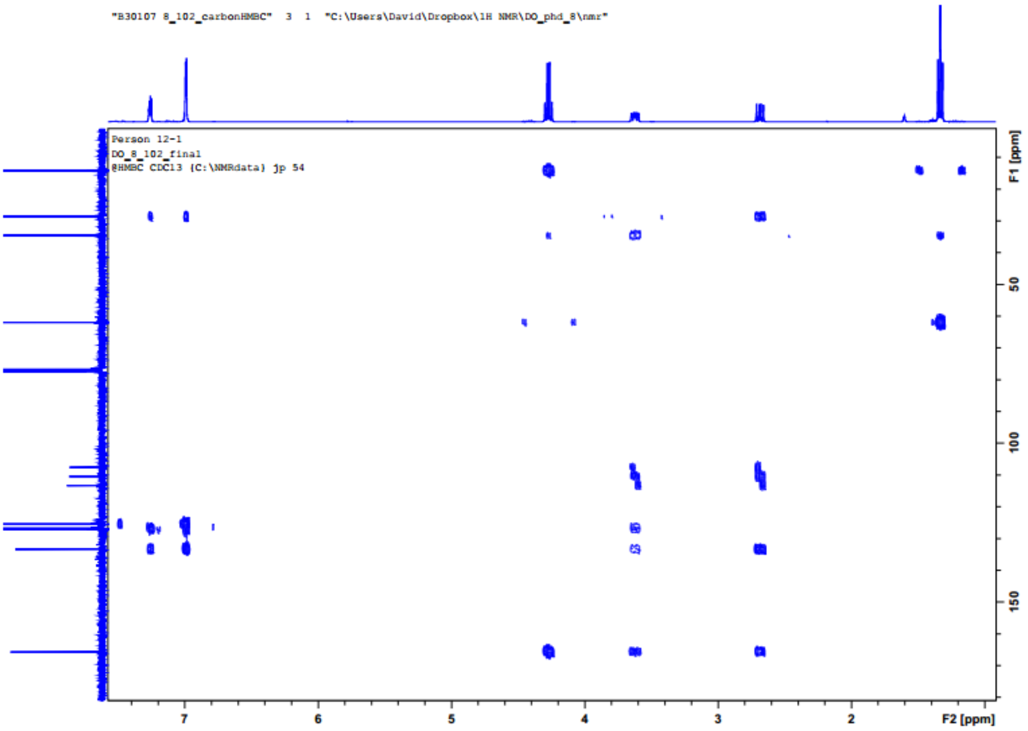


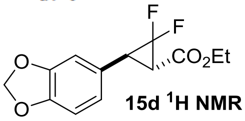


**15d $^1$H NMR**

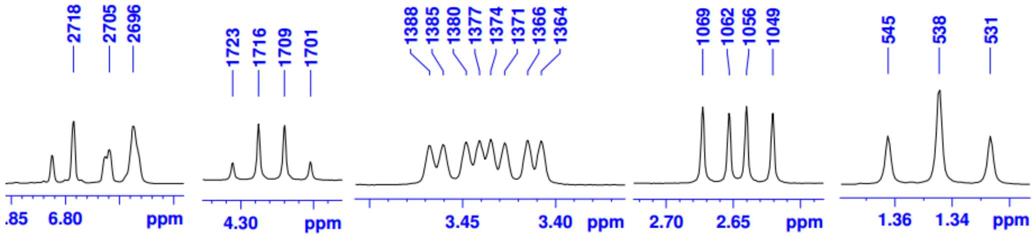


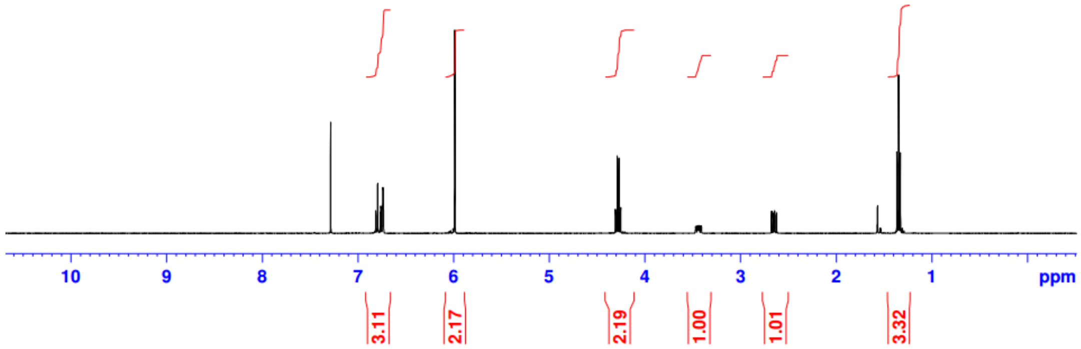

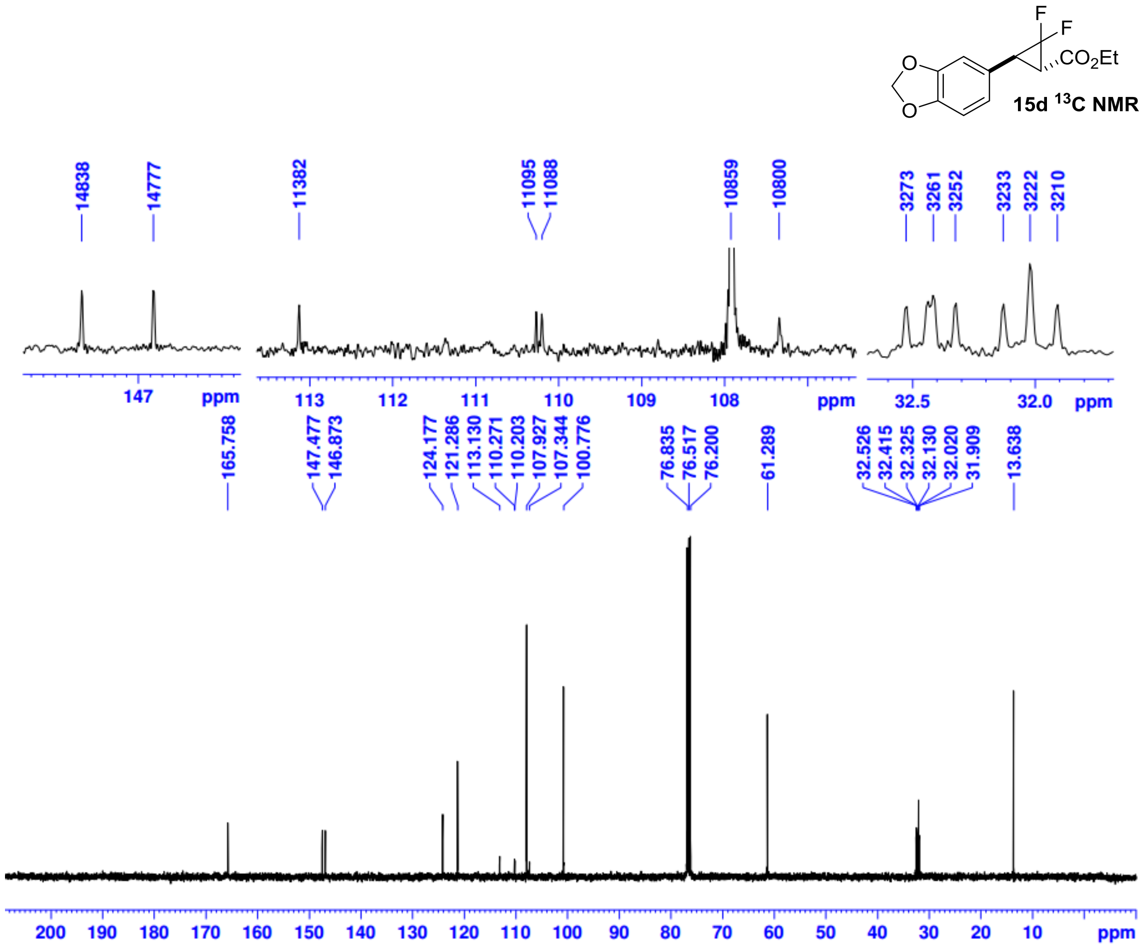
15d $^{13}$C NMR

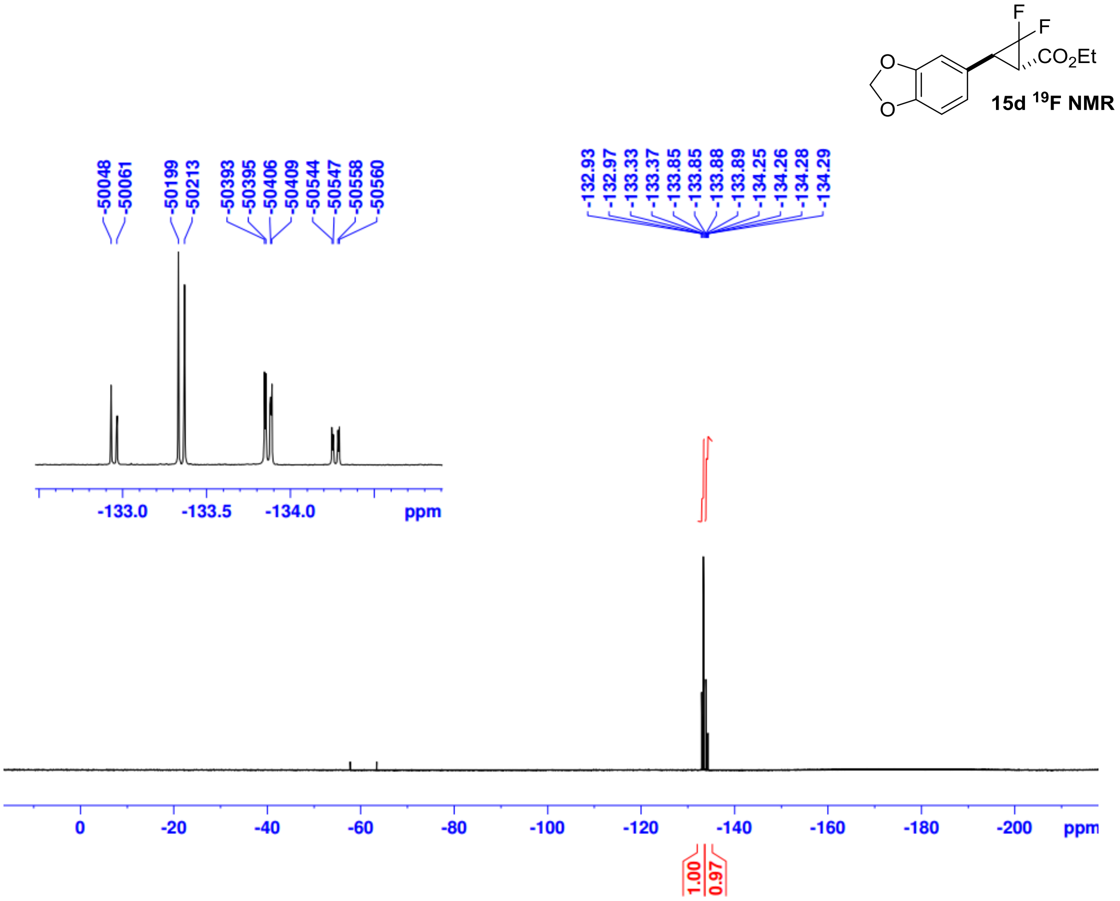
15d $^{19}$F NMR

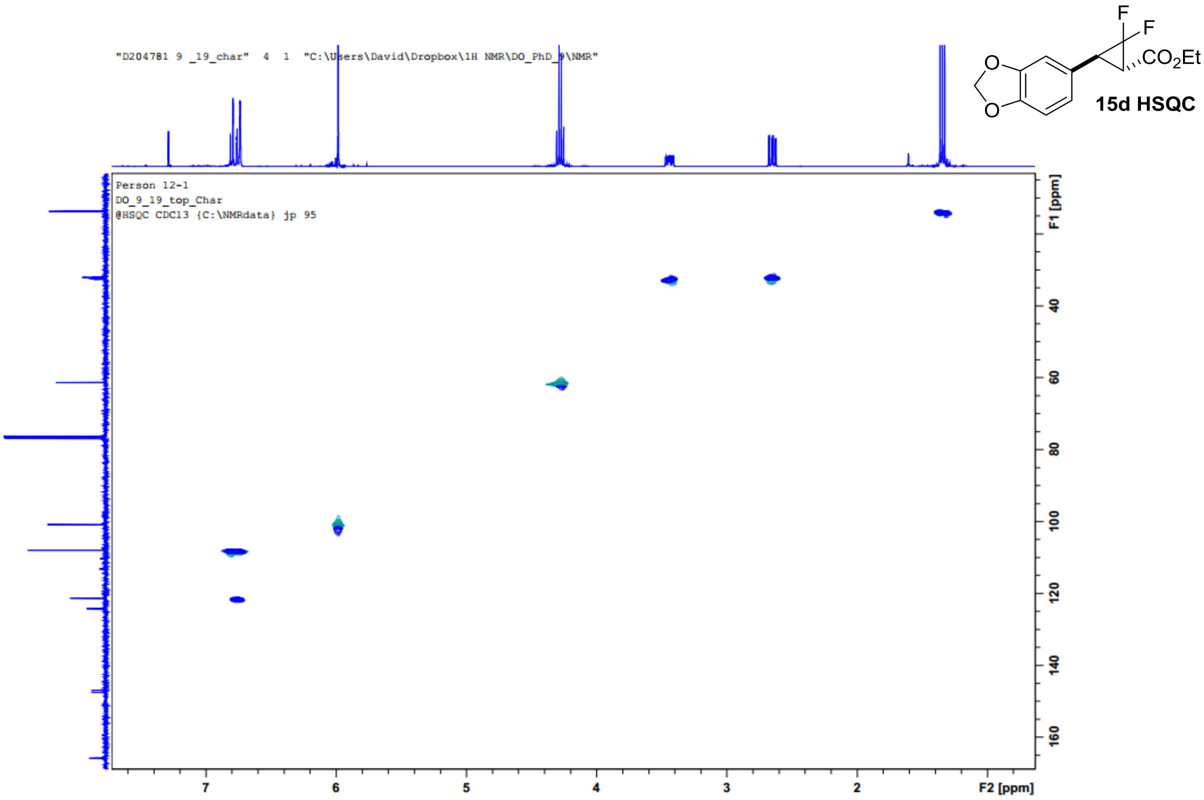


15d HSQC

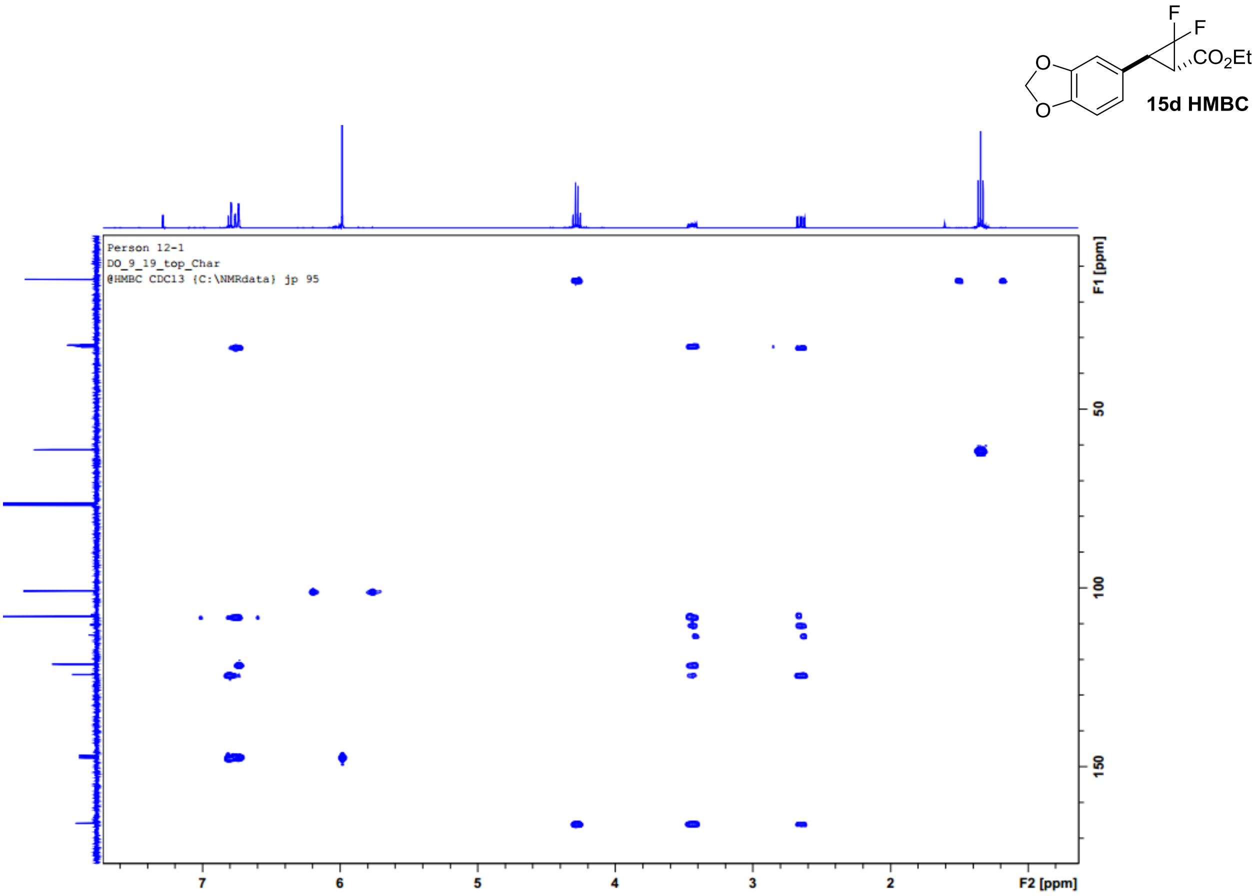


15d HMBC

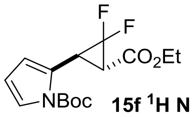


**15f $^{1}$H NMR**

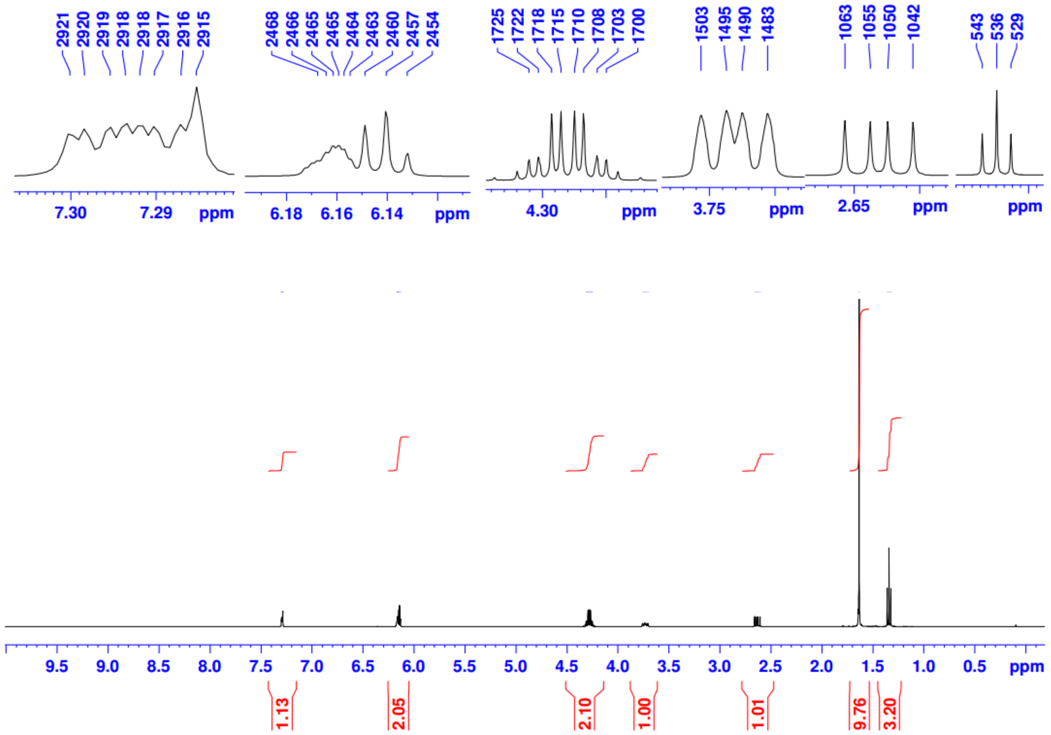


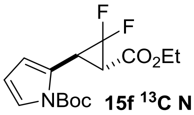


**15f $^{13}$C NMR**

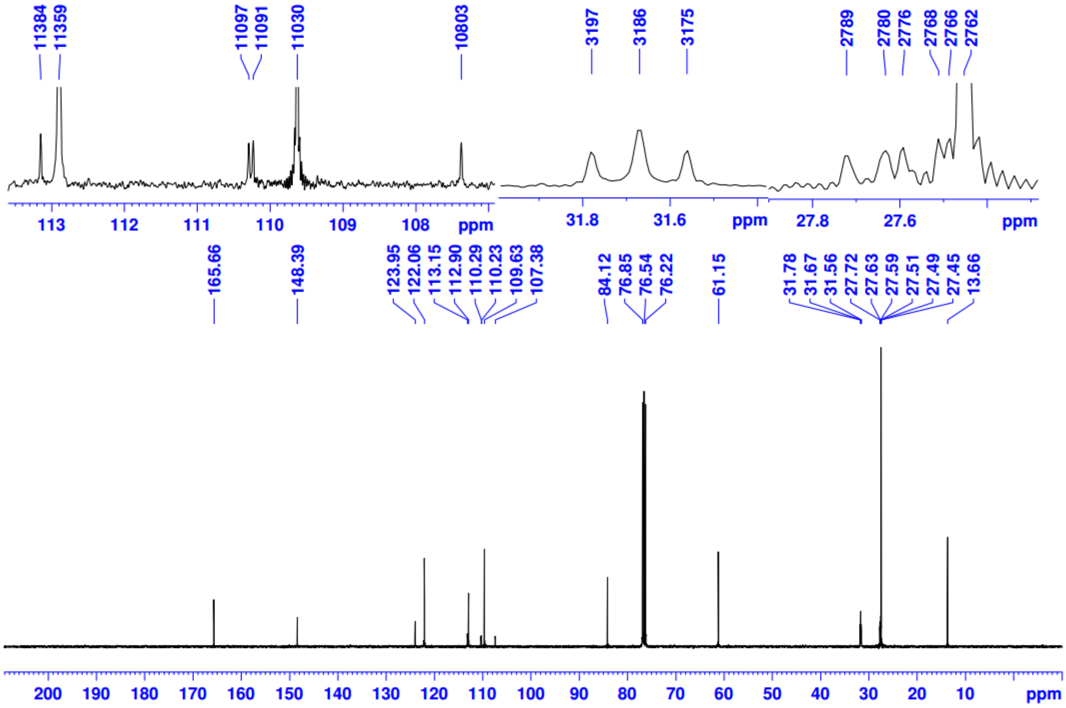

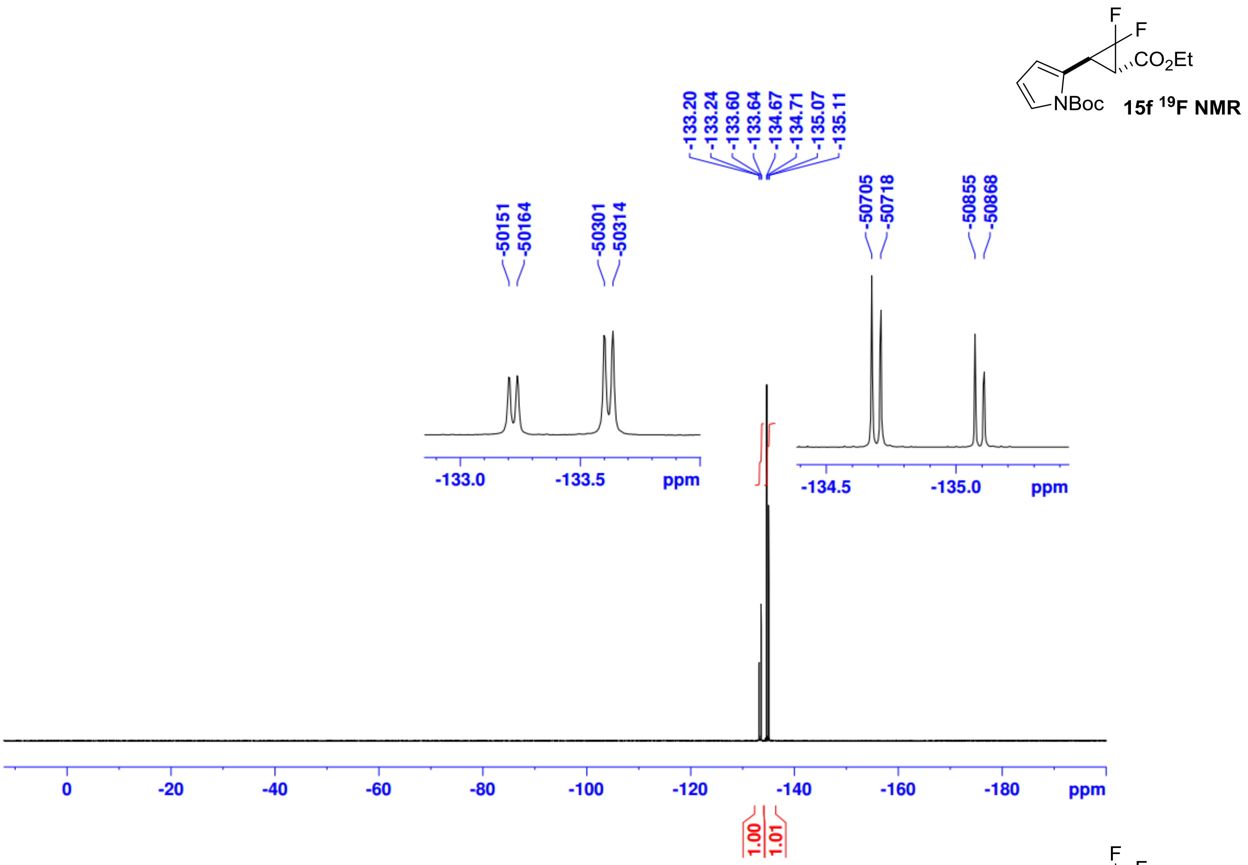
F
F
CO2Et
NBoc
15f 19F NMR
-133.20
-133.24
-133.60
-133.64
-134.67
-134.71
-135.07
-135.11
-50151
-50164
-50301
-50314
-50705
-50718
-50855
-50868
-133.0
-133.5
ppm
-134.5
-135.0
ppm
0
-20
-40
-60
-80
-100
-120
-140
-160
-180
ppm
1.00
1.01

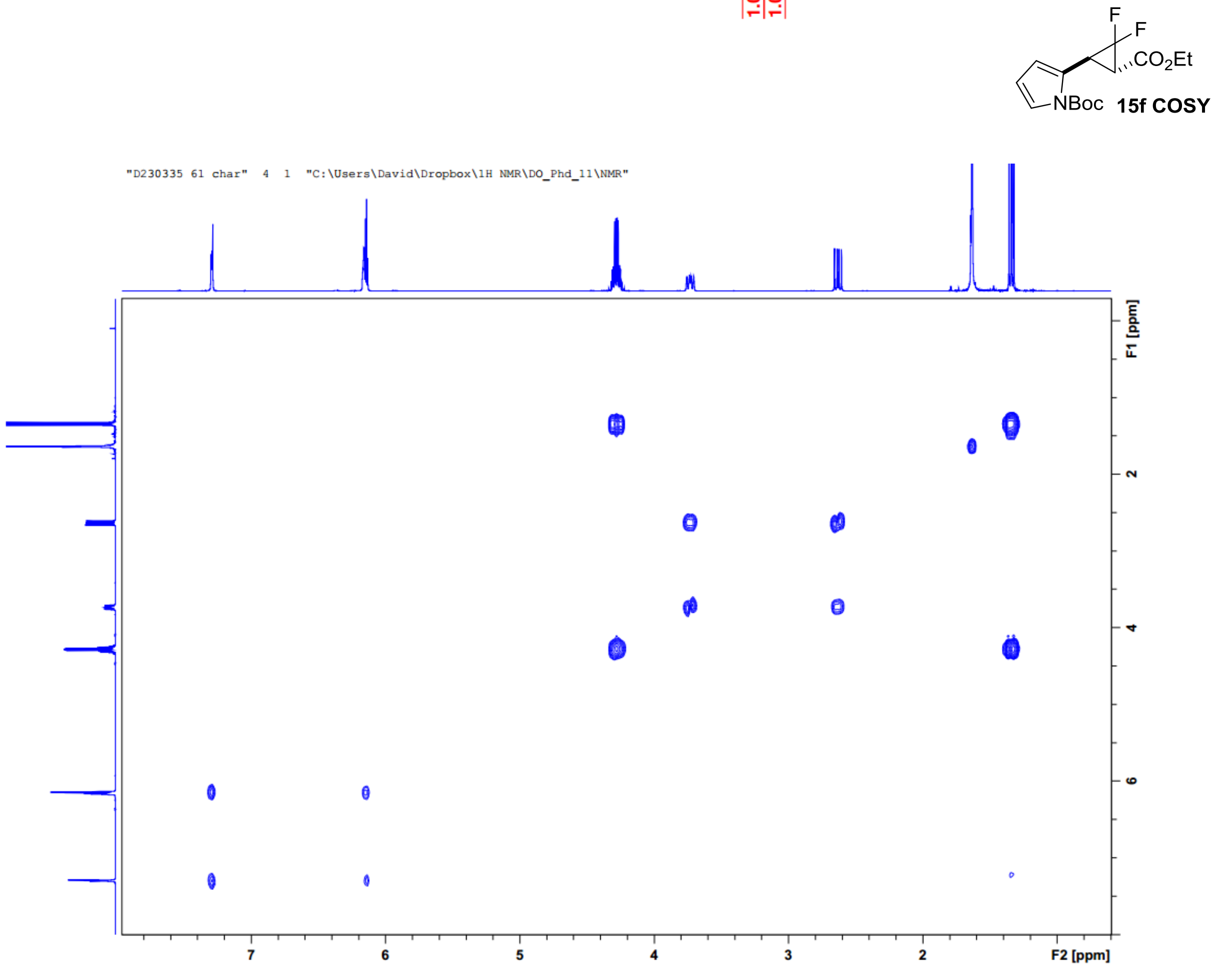
F
F
CO2Et
NBoc
15f COSY
"D230335 61 char" 4 1 "C:\Users\David\Dropbox\1H NMR\DO_Phd_11\NMR"
F1 [ppm]
2
4
6
7
6
5
4
3
2
F2 [ppm]

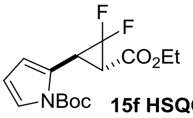


**15f HSQC**

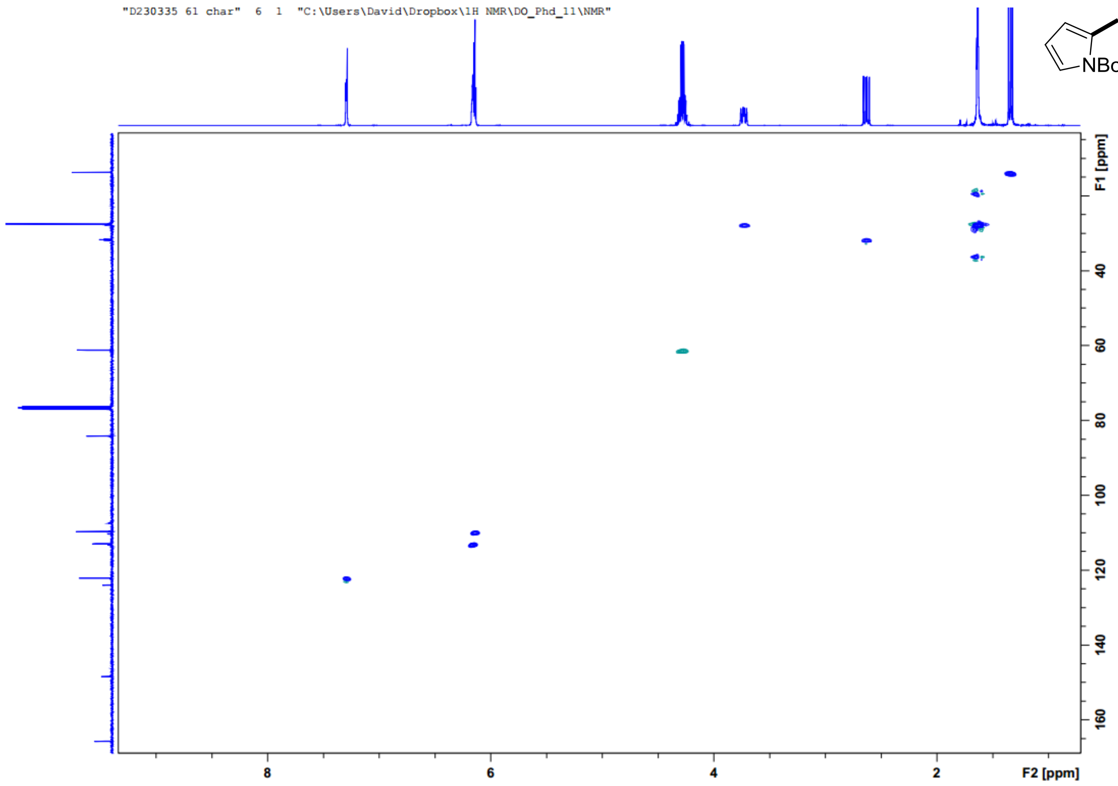


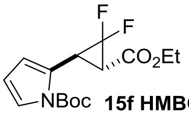


**15f HMBC**

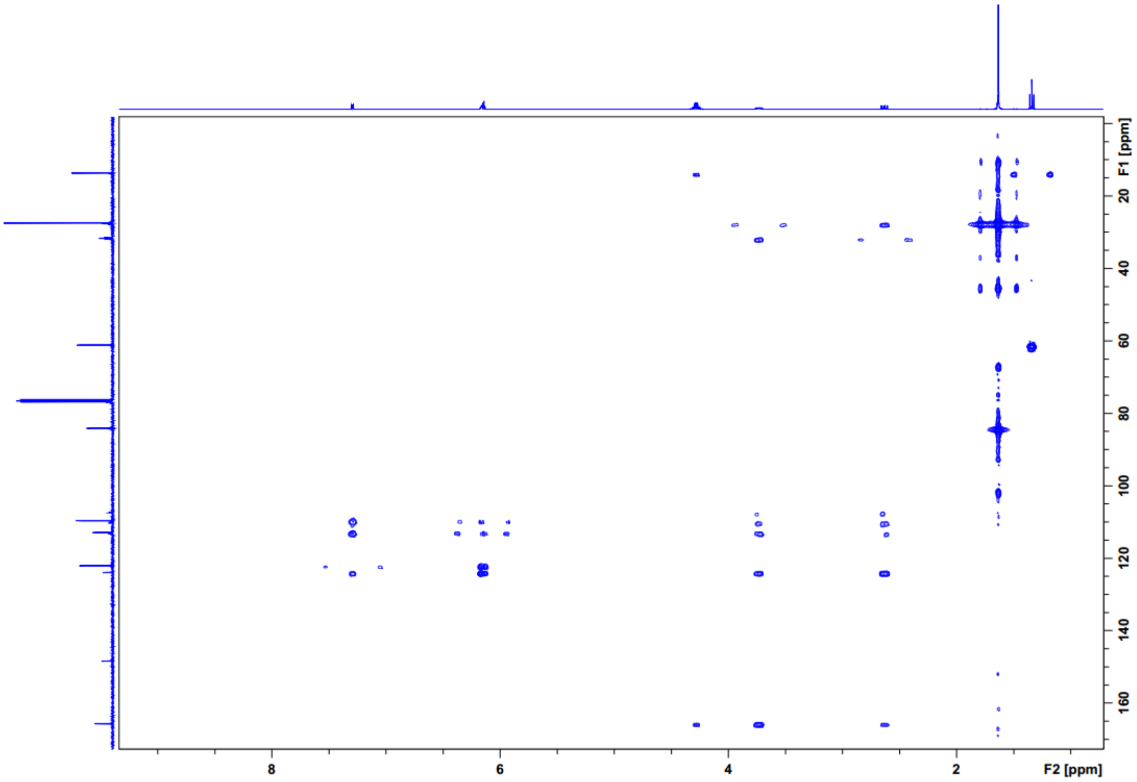

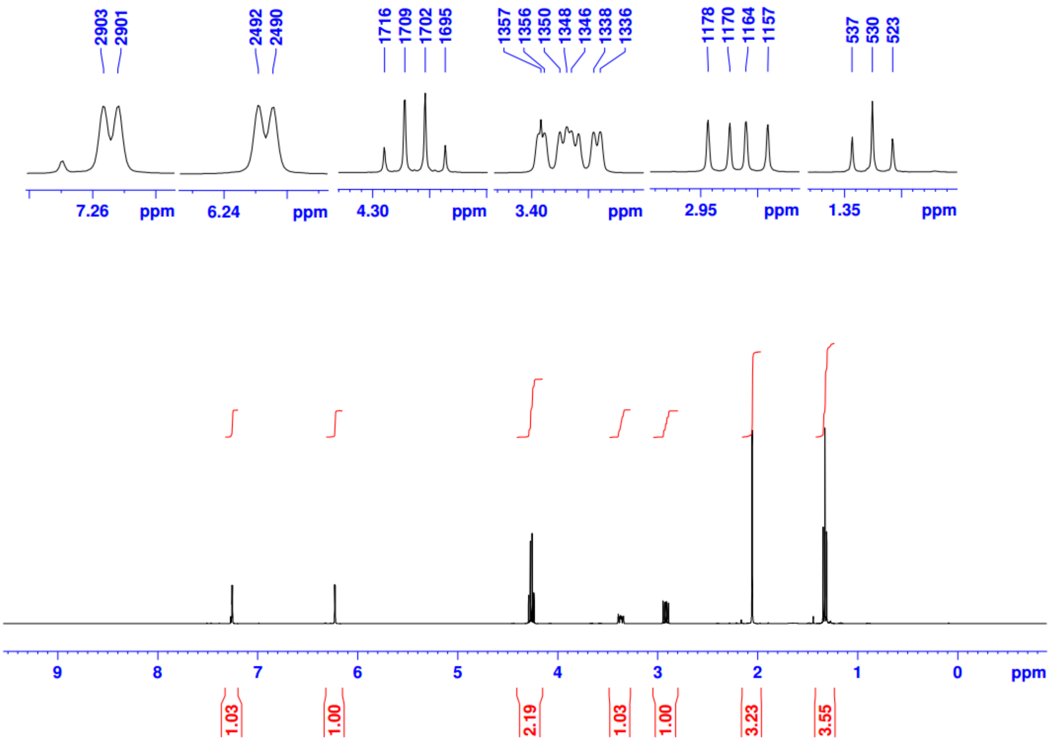


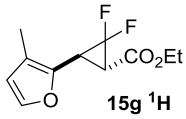


**15g** $^{1}$H

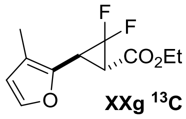


**XXg** $^{13}$C

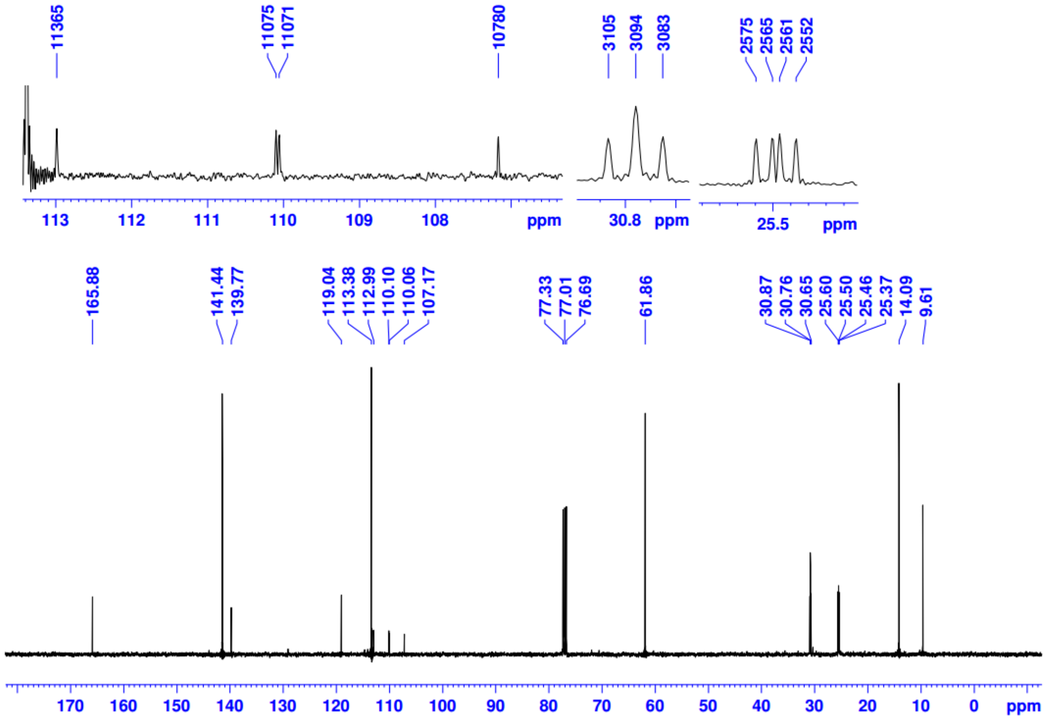

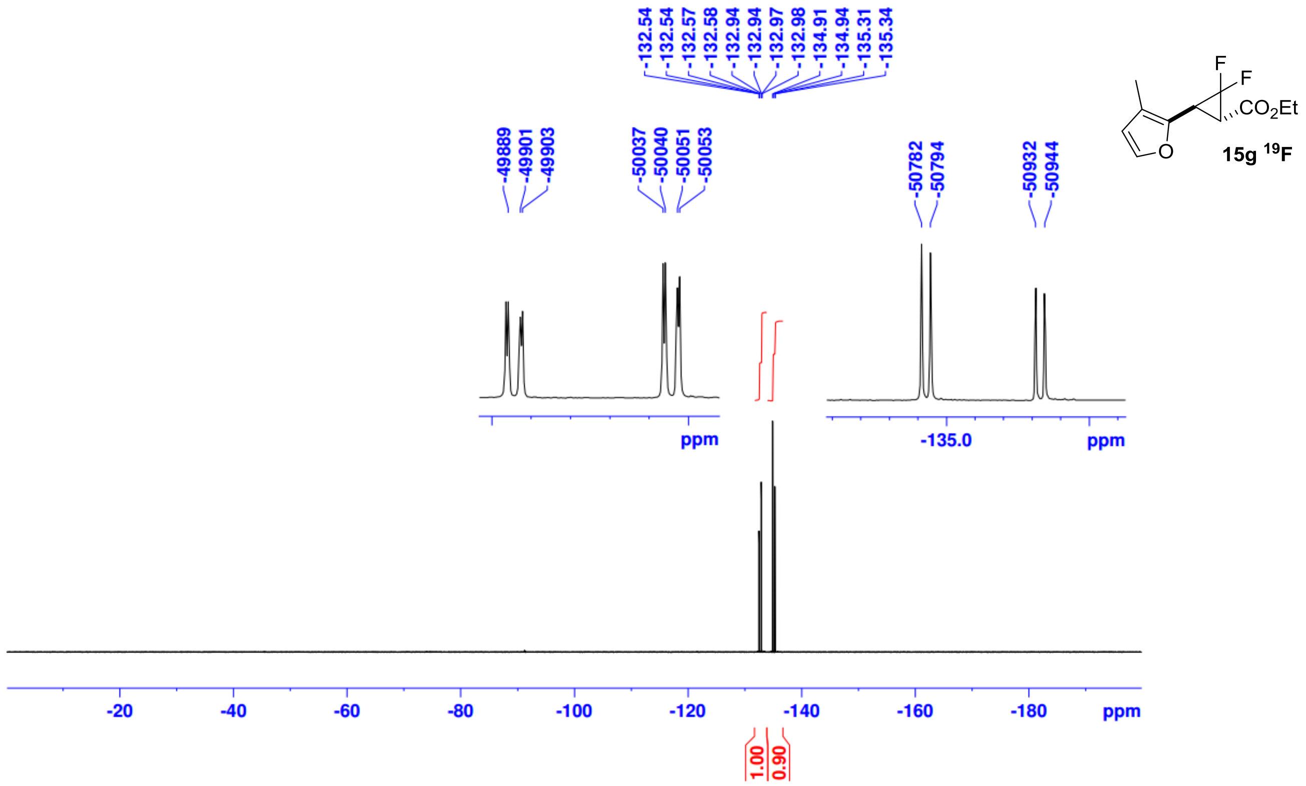


**15g $^{19}$F**

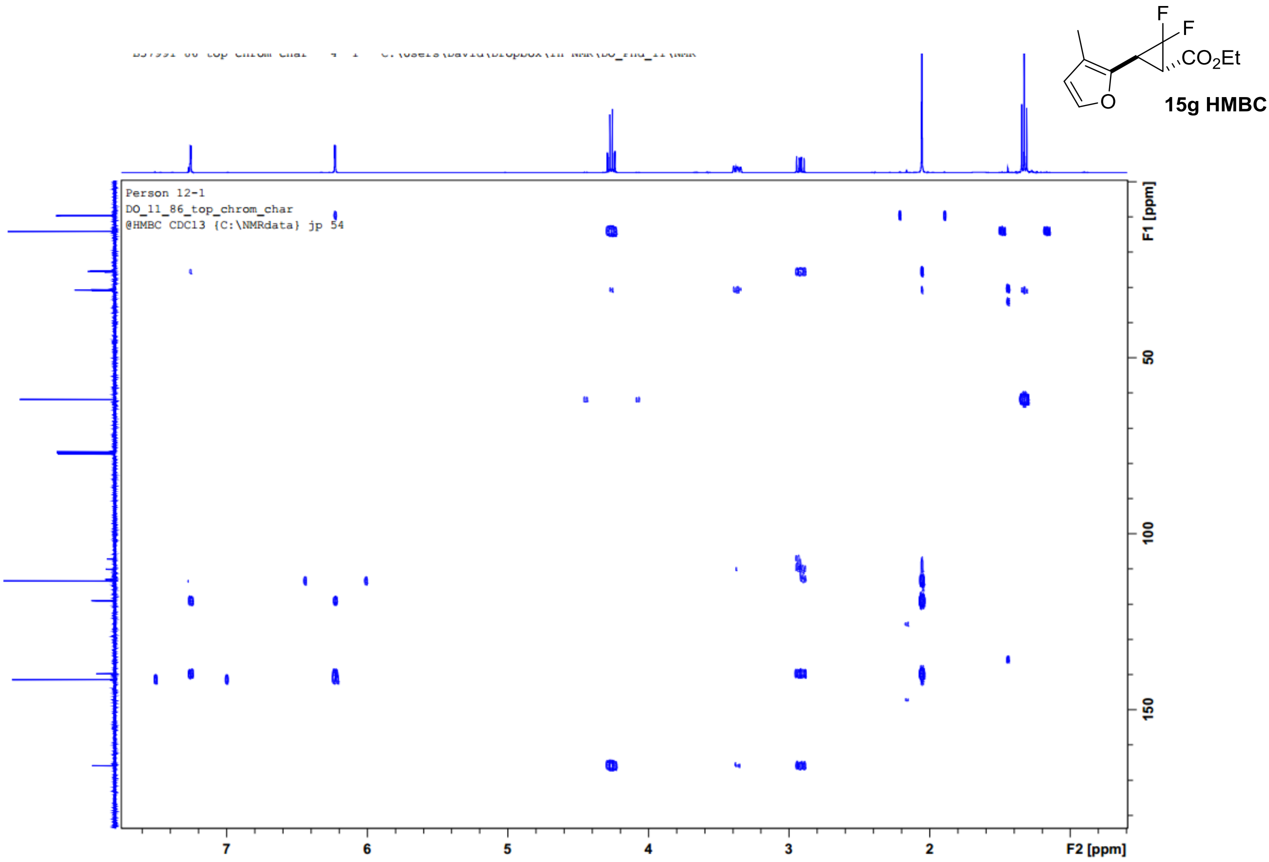


**15g HMBC**

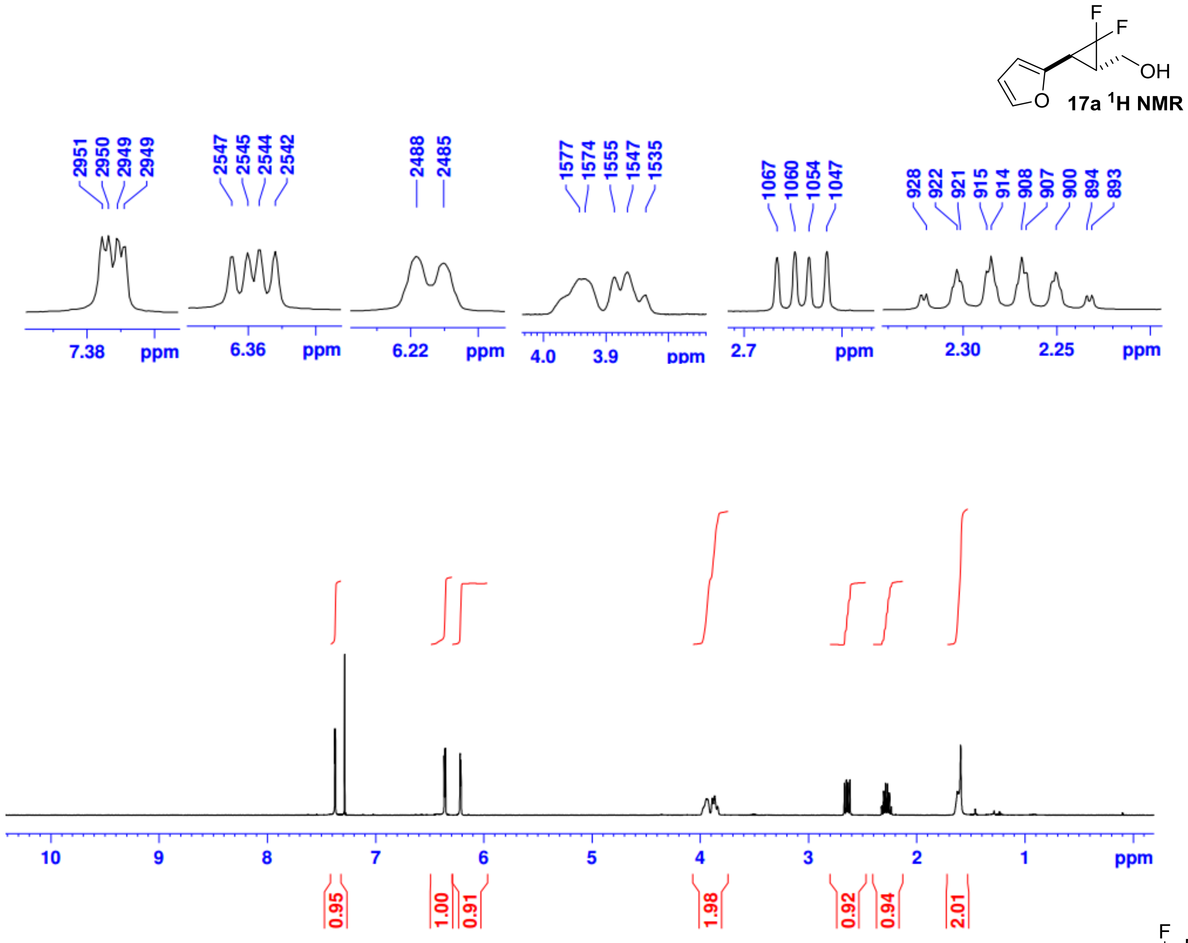


17a $^{1}$H NMR

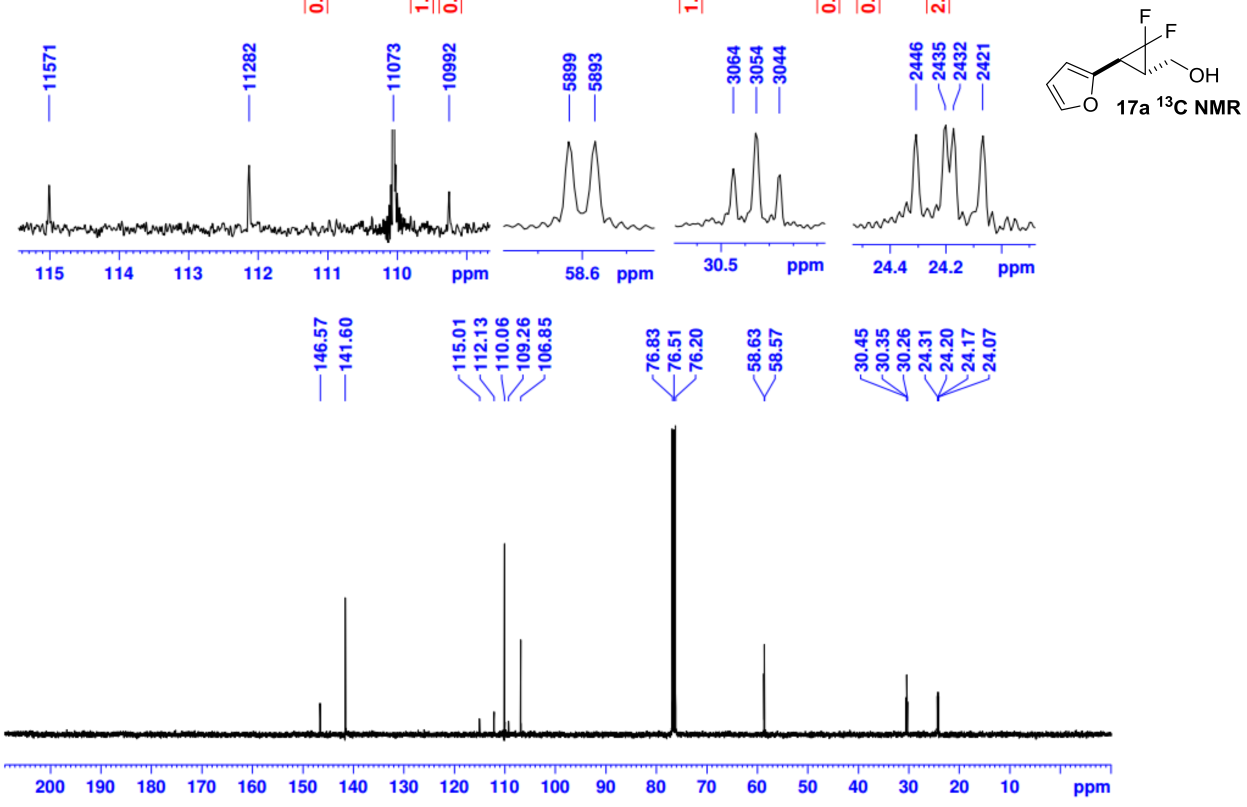


17a $^{13}$C NMR

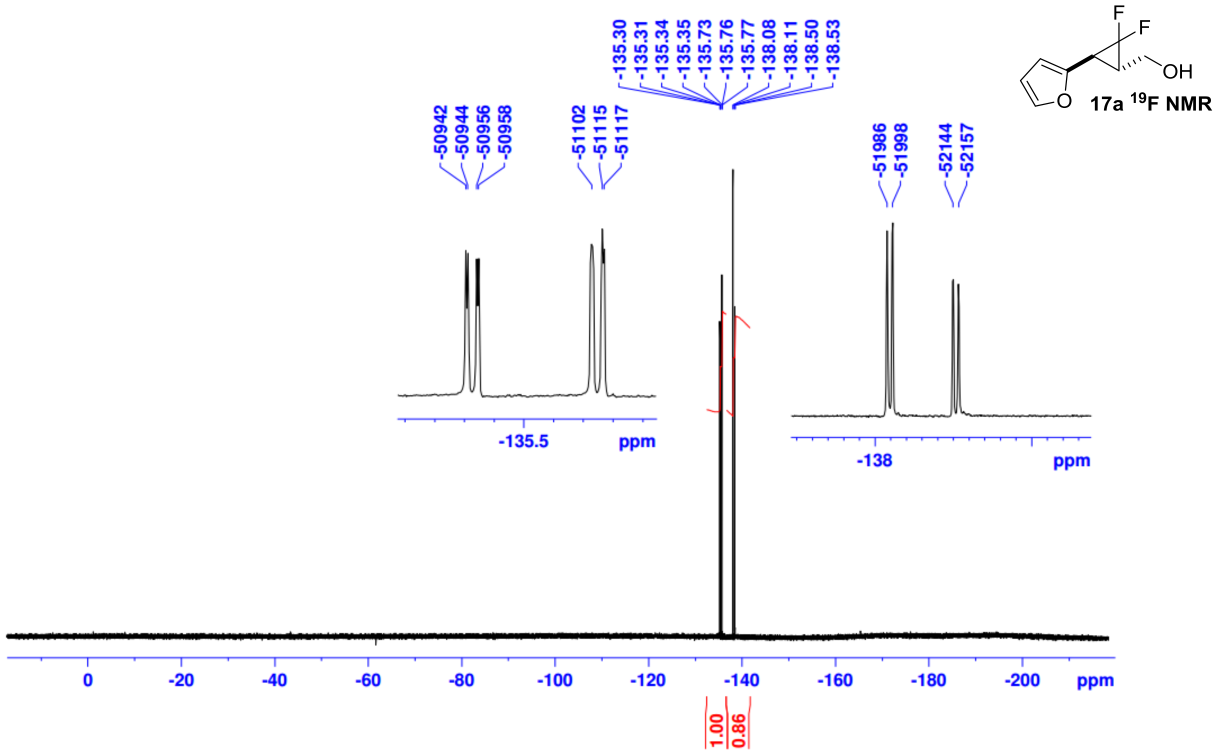
-135.30
-135.31
-135.34
-135.35
-135.73
-135.76
-135.77
-138.08
-138.11
-138.50
-138.53
-50942
-50944
-50956
-50958
-51102
-51115
-51117
-51986
-51998
-52144
-52157
-135.5
ppm
-138
ppm
0
-20
-40
-60
-80
-100
-120
-140
-160
-180
-200
ppm
1.00
0.86
F
F
OH
O
17a ¹⁹F NMR

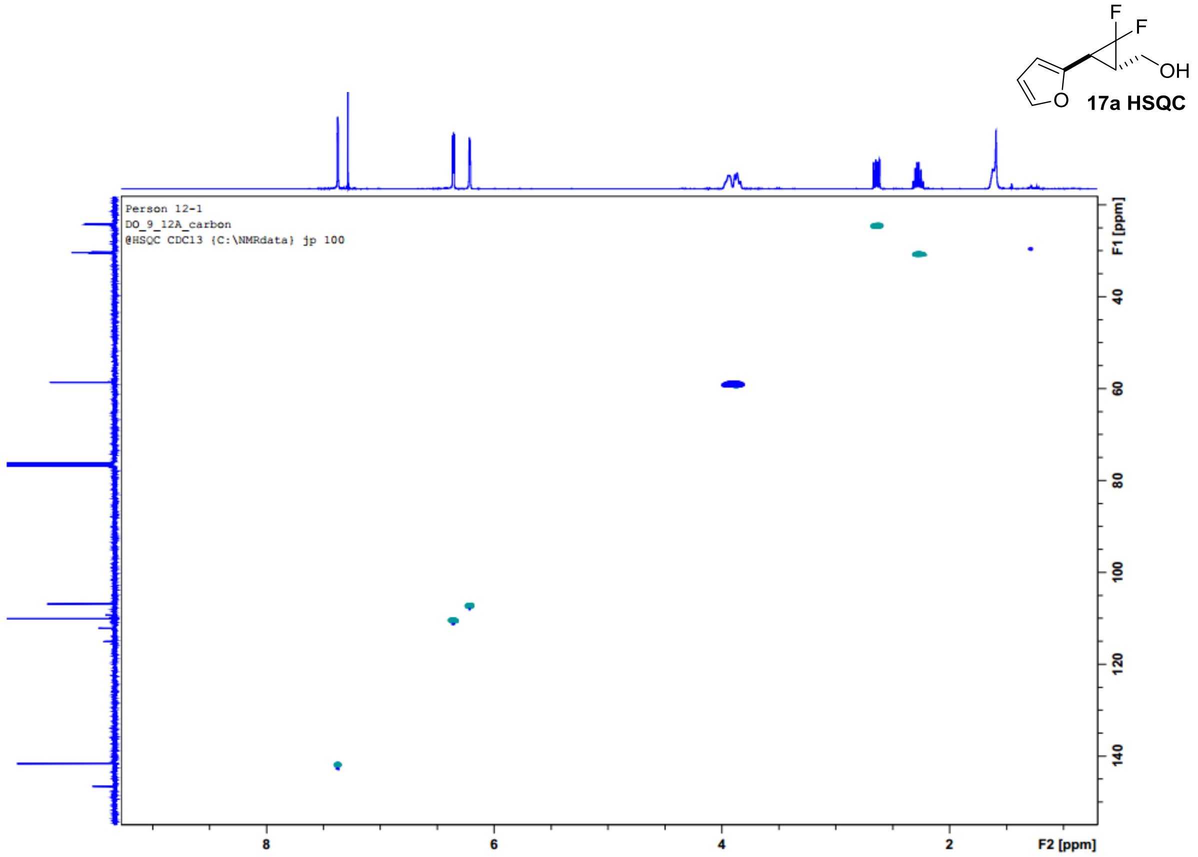
F
F
OH
O
17a HSQC
Person 12-1
DO_9_12A_carbon
@HSQC CDCl3 {C:\NMRdata} jp 100
8
6
4
2
F2 [ppm]
40
60
80
100
120
140
F1 [ppm]

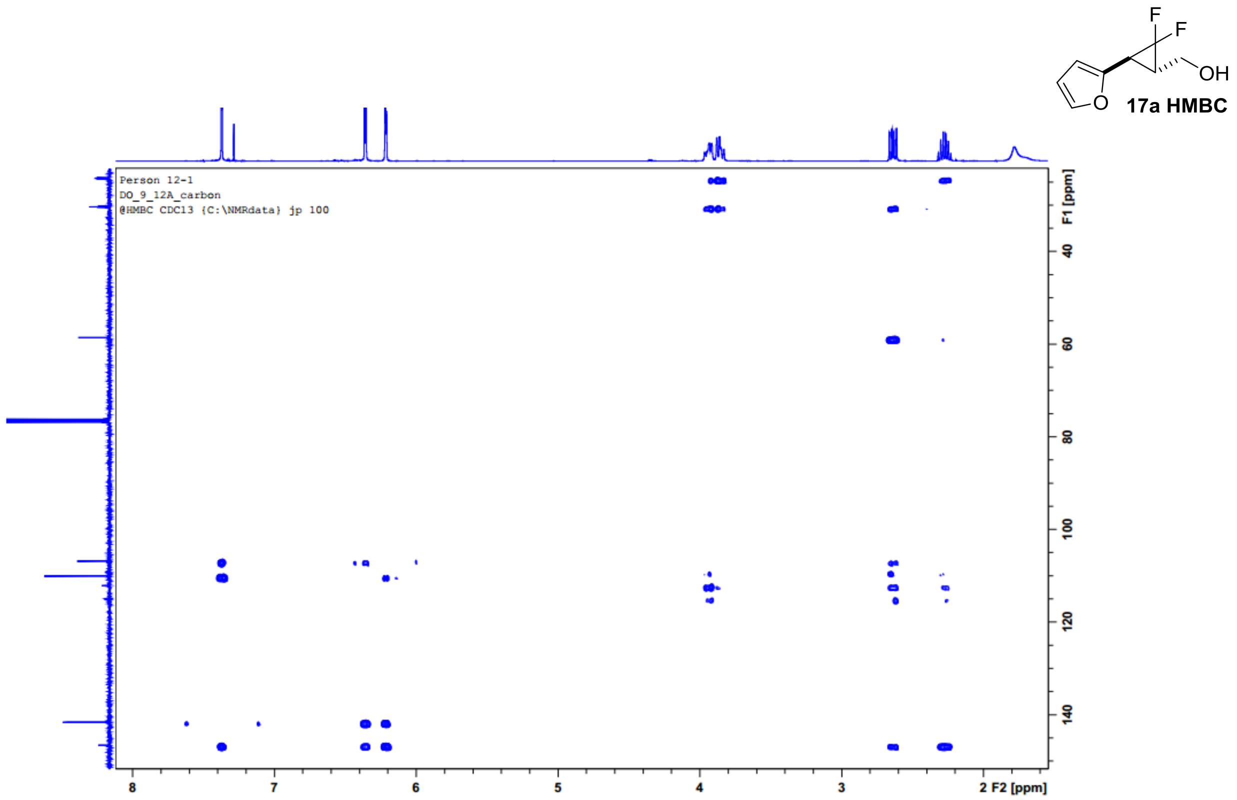


17a HMBC

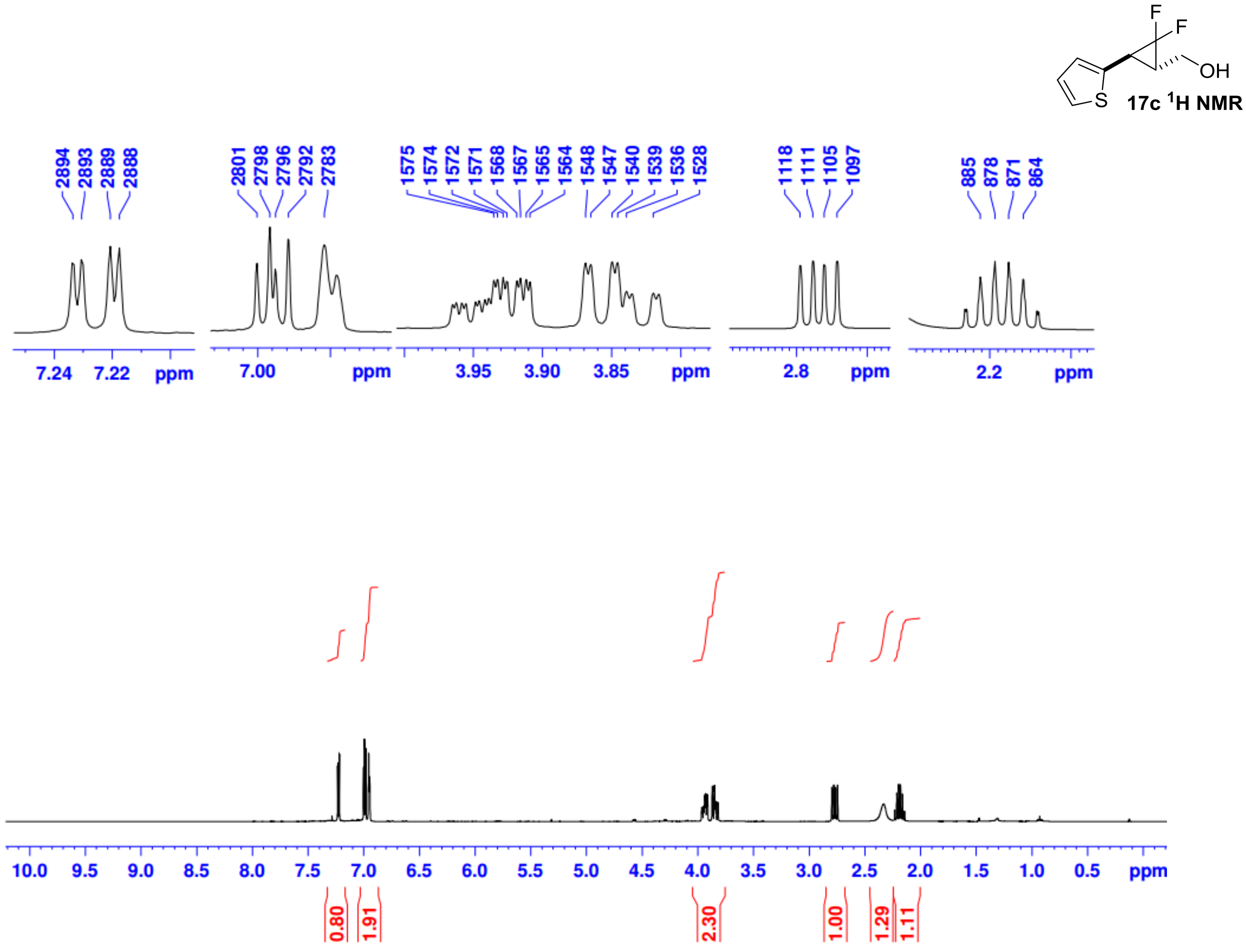


17c $^{1}$H NMR

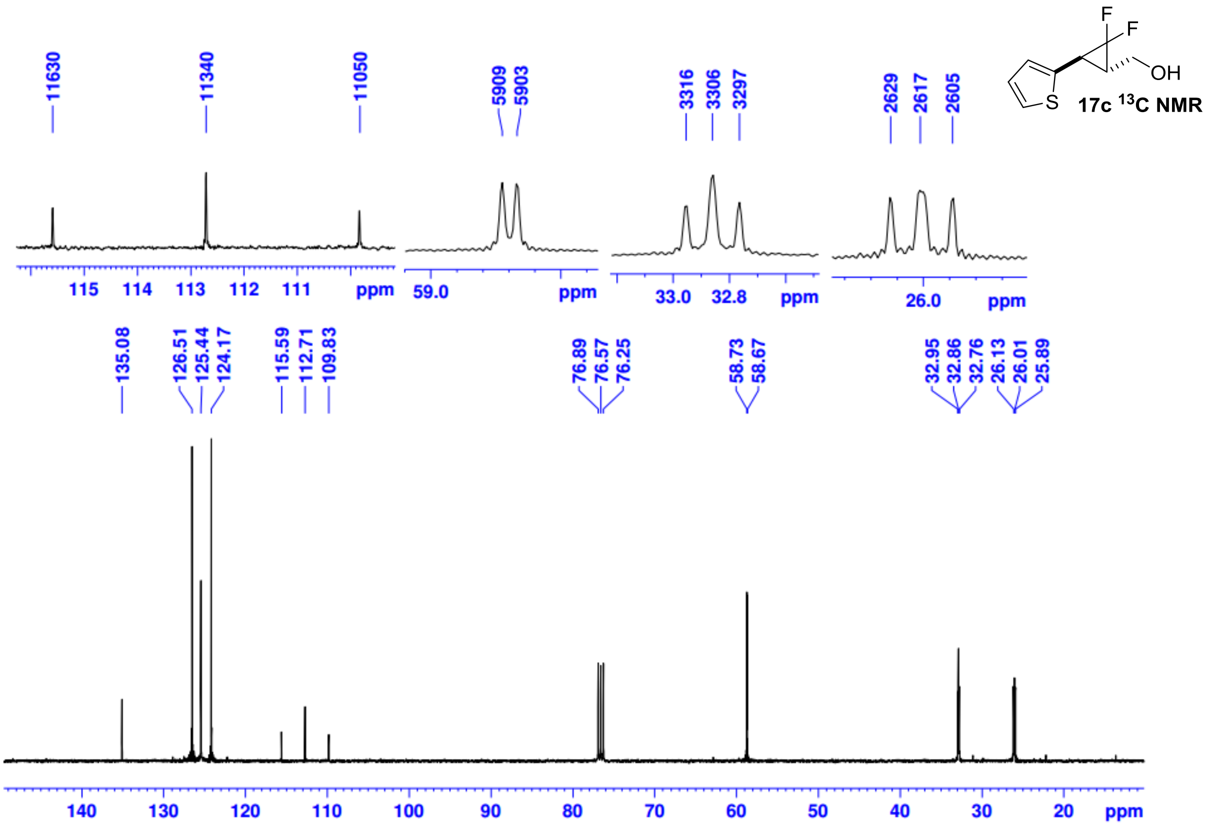
17c $^{13}$C NMR
F
F
OH
S
11630
11340
11050
5909
5903
3316
3306
3297
2629
2617
2605
135.08
126.51
125.44
124.17
115.59
112.71
109.83
76.89
76.57
76.25
58.73
58.67
32.95
32.86
32.76
26.13
26.01
25.89
ppm

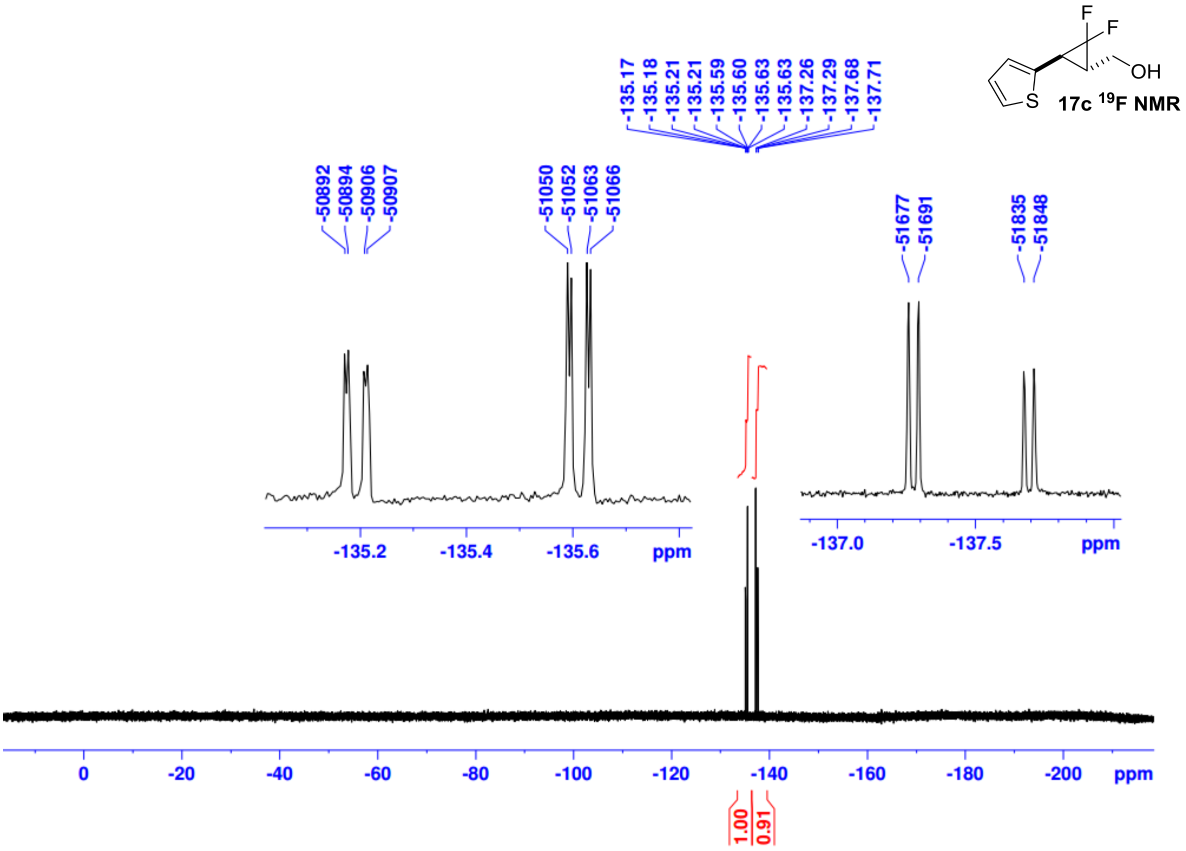
17c $^{19}$F NMR
F
F
OH
S
-135.17
-135.18
-135.21
-135.21
-135.59
-135.60
-135.63
-135.63
-137.26
-137.29
-137.68
-137.71
-50892
-50894
-50906
-50907
-51050
-51052
-51063
-51066
-51677
-51691
-51835
-51848
1.00
0.91
ppm

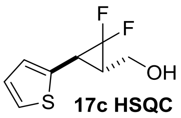

17c HSQC

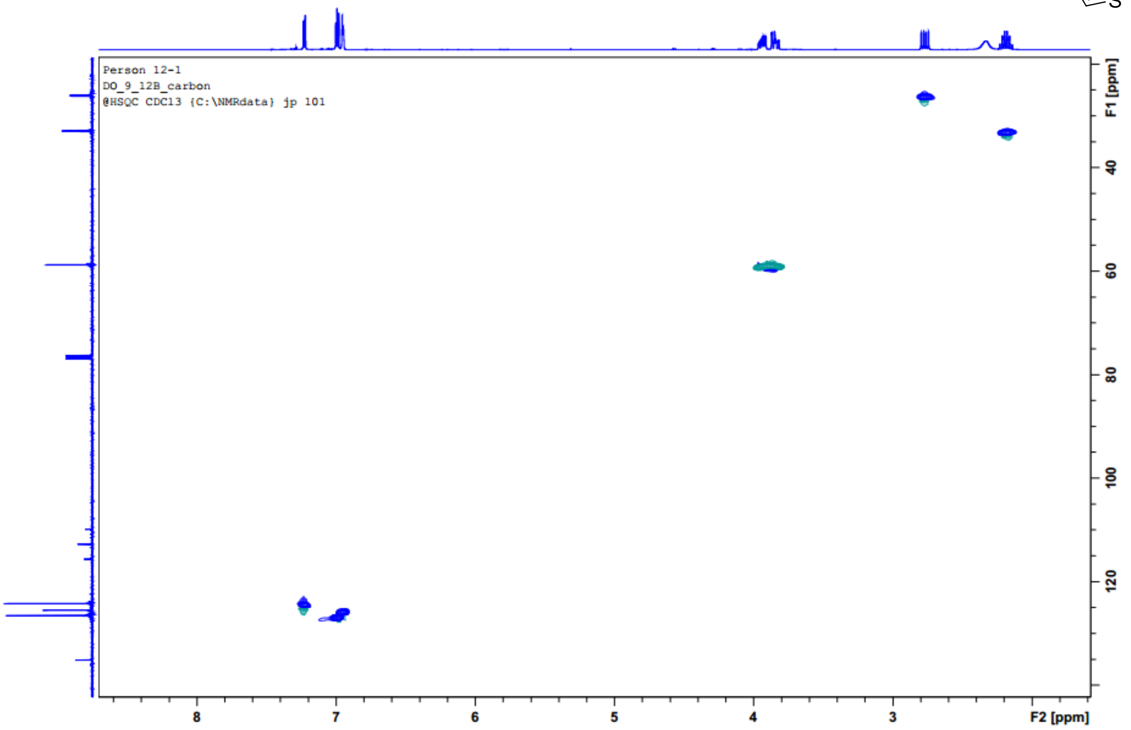


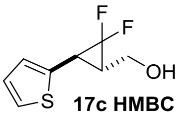

17c HMBC

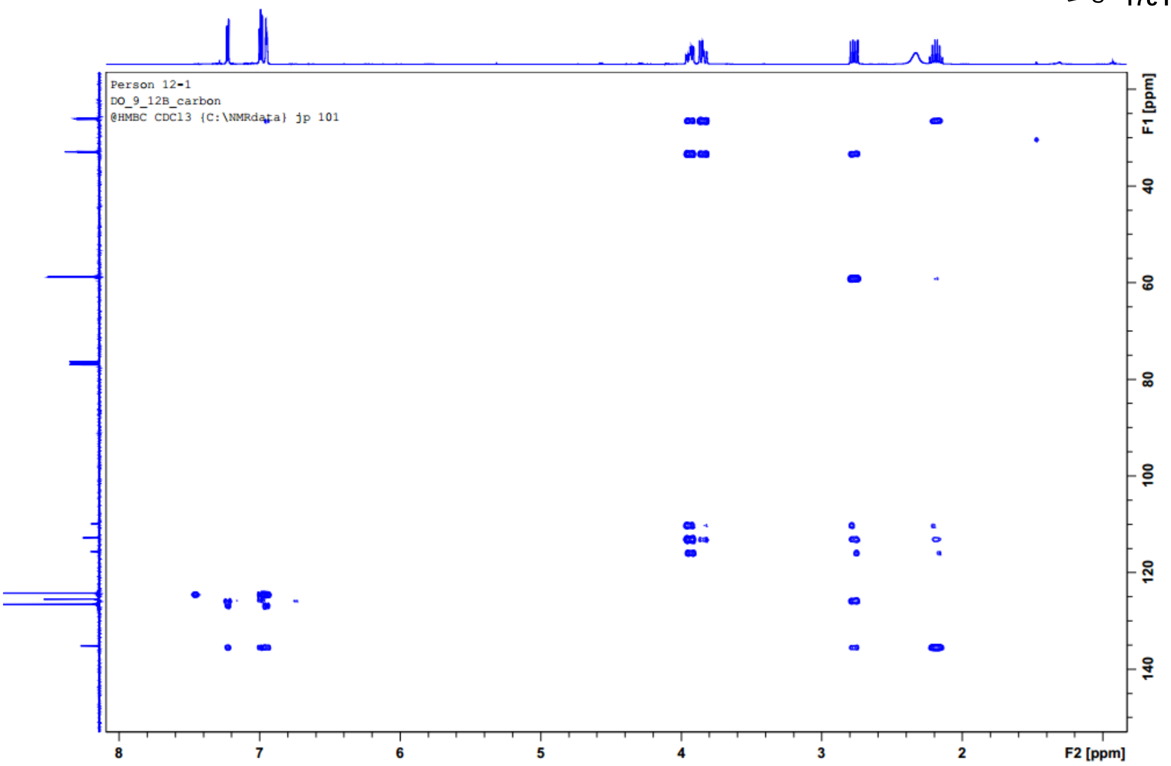

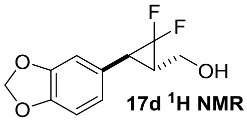

17d $^{1}$H NMR

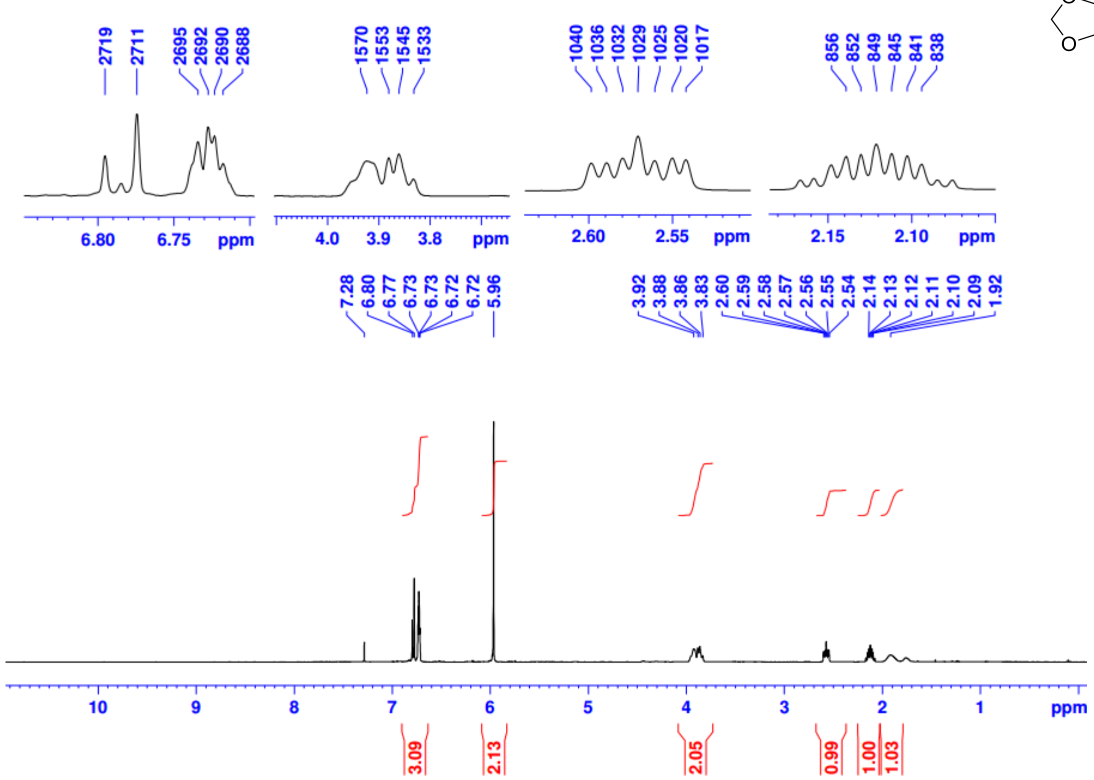

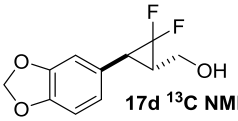

17d $^{13}$C NMR

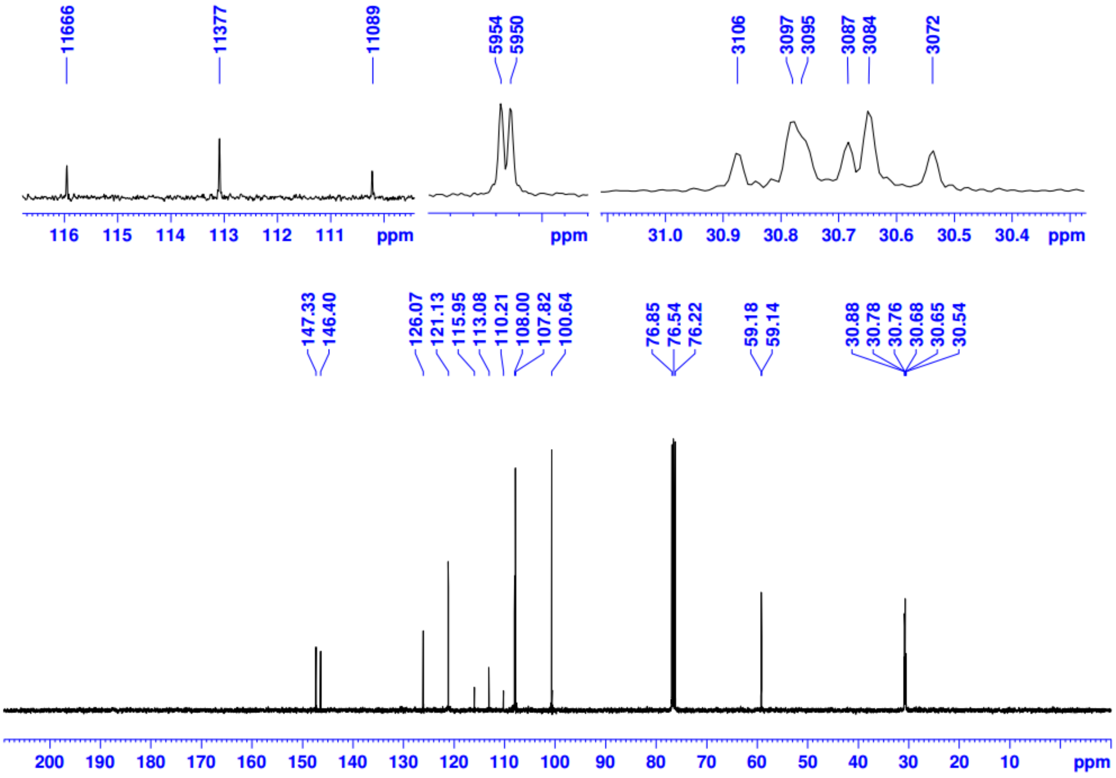

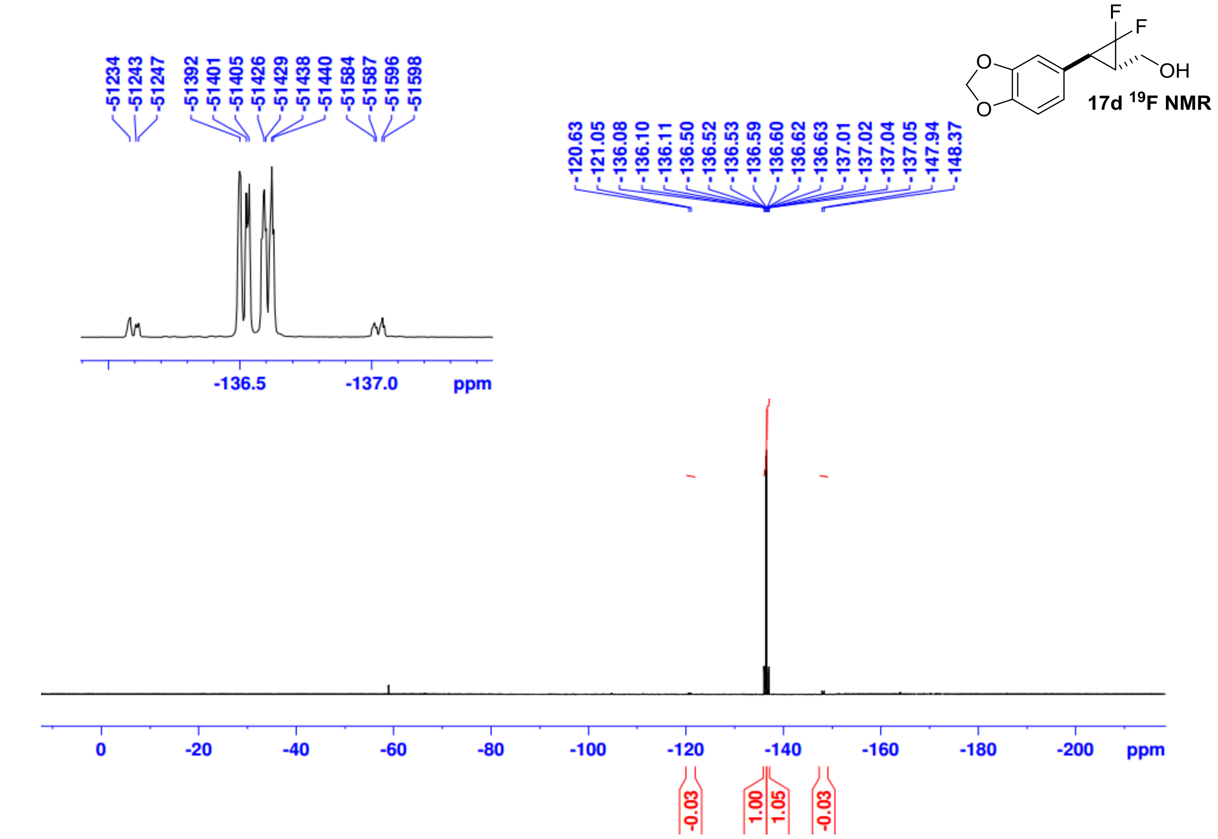
17d 19F NMR
ppm

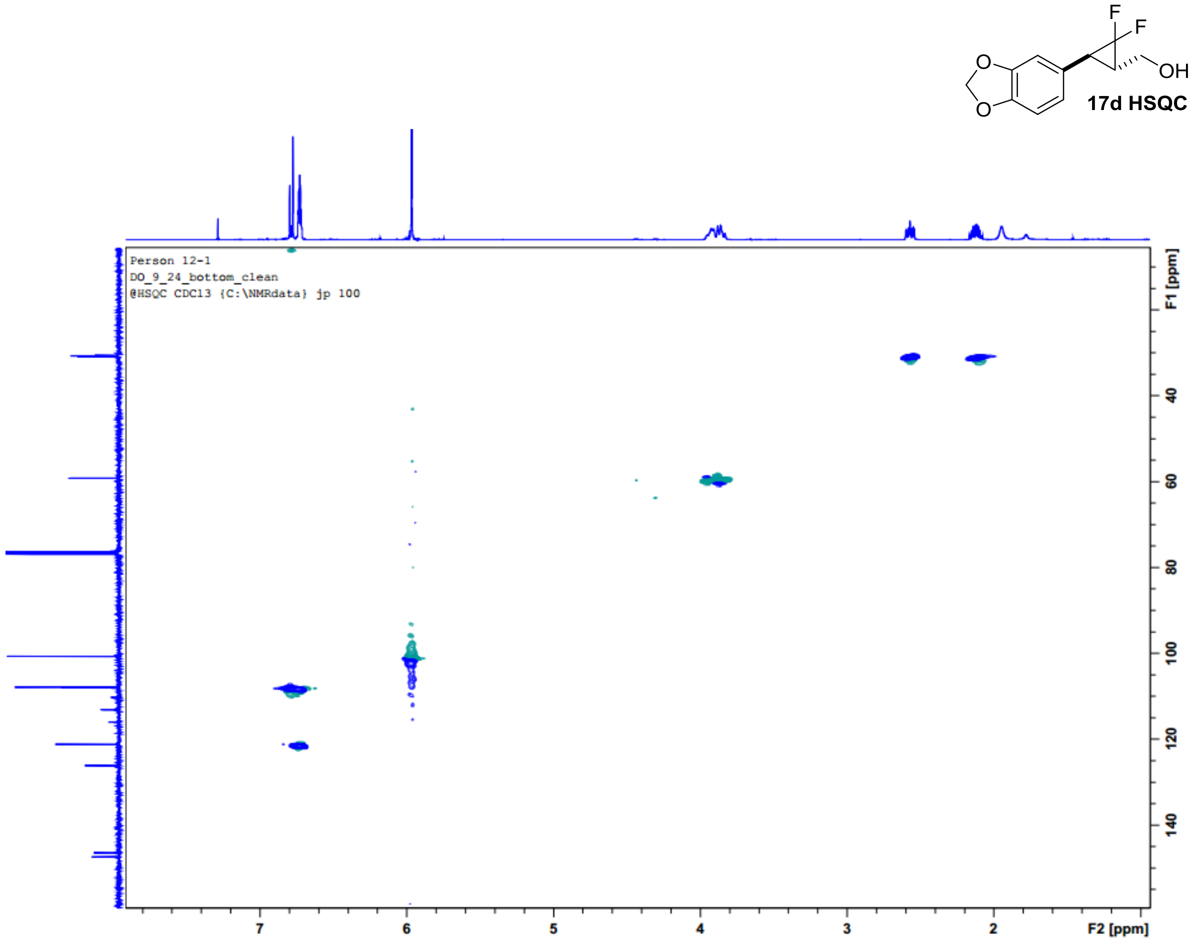
17d HSQC
Person 12-1
DO_9_24_bottom_clean
@HSQC CDCl3 {C:\NMRdata} jp 100
F1 [ppm]
F2 [ppm]

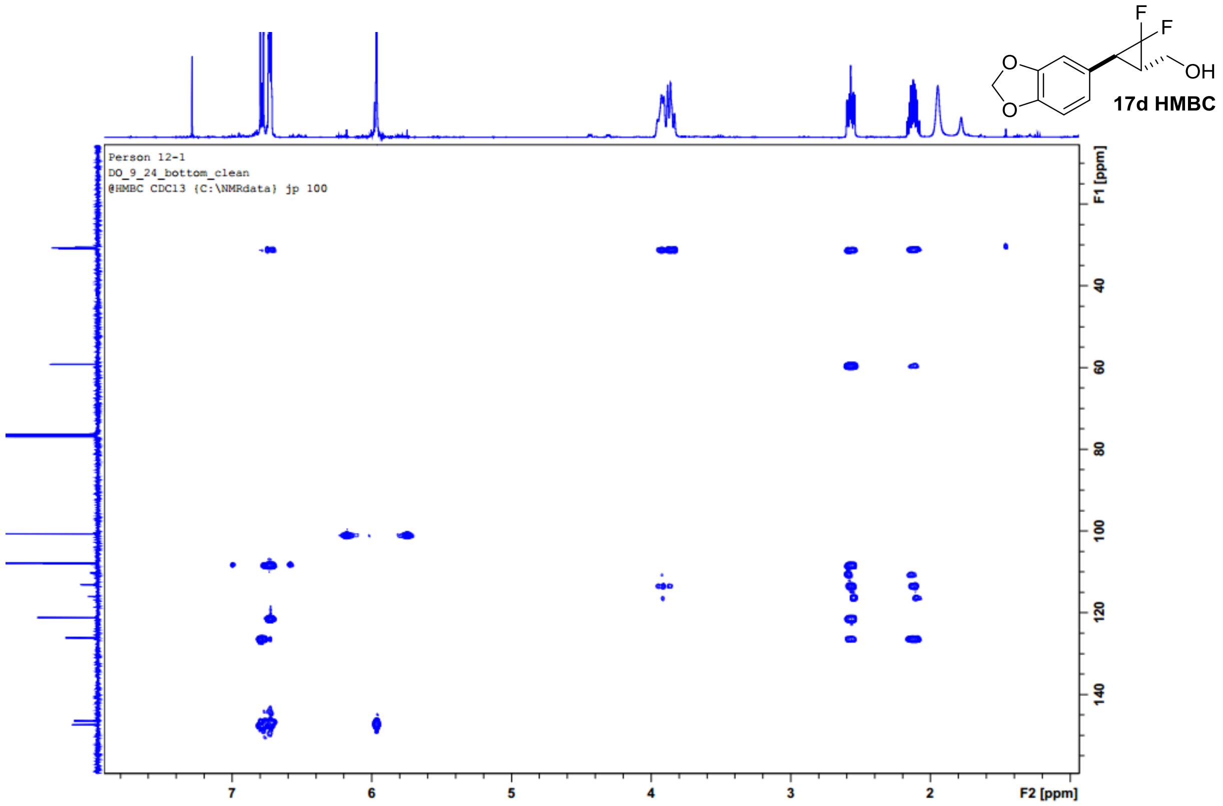


**17d HMBC**

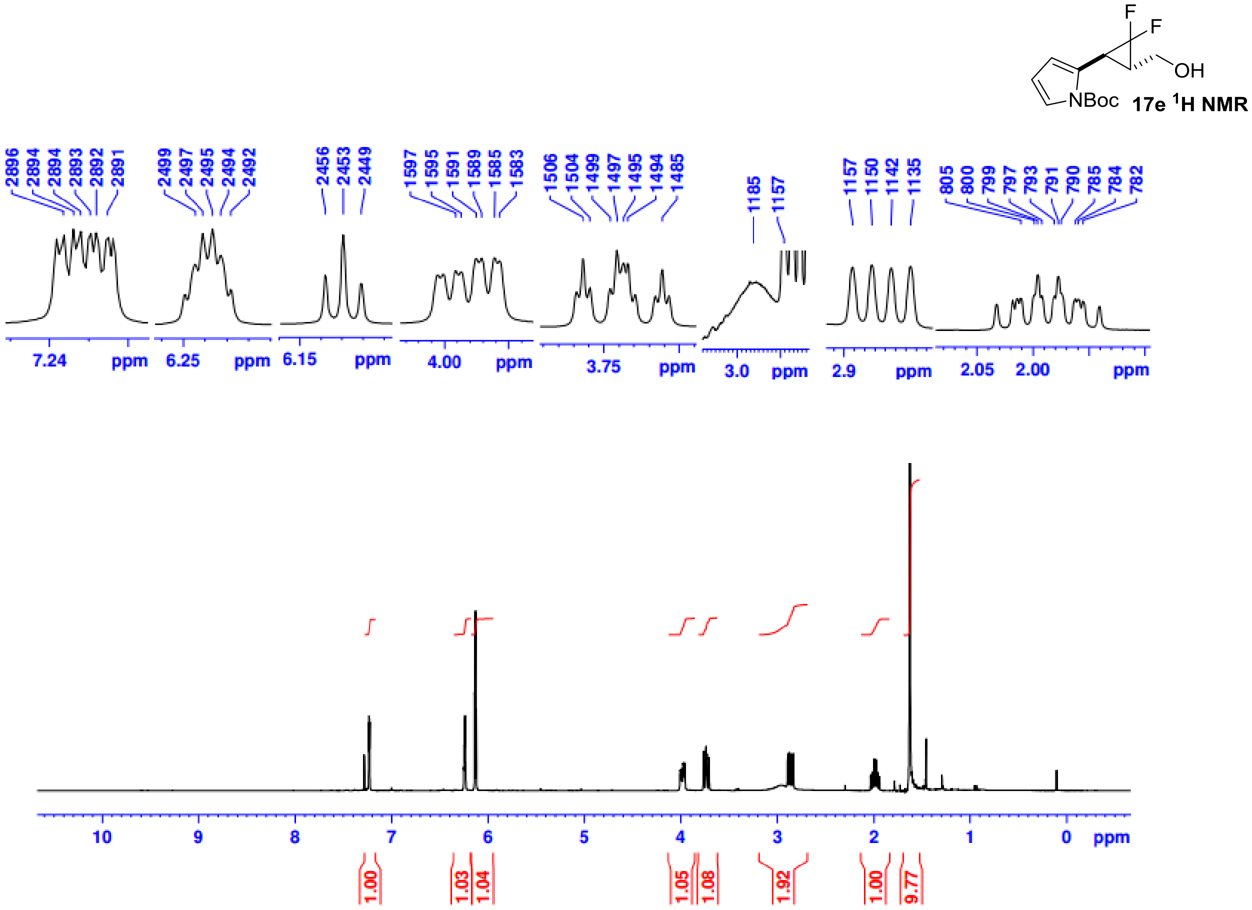


**17e $^{1}$H NMR**

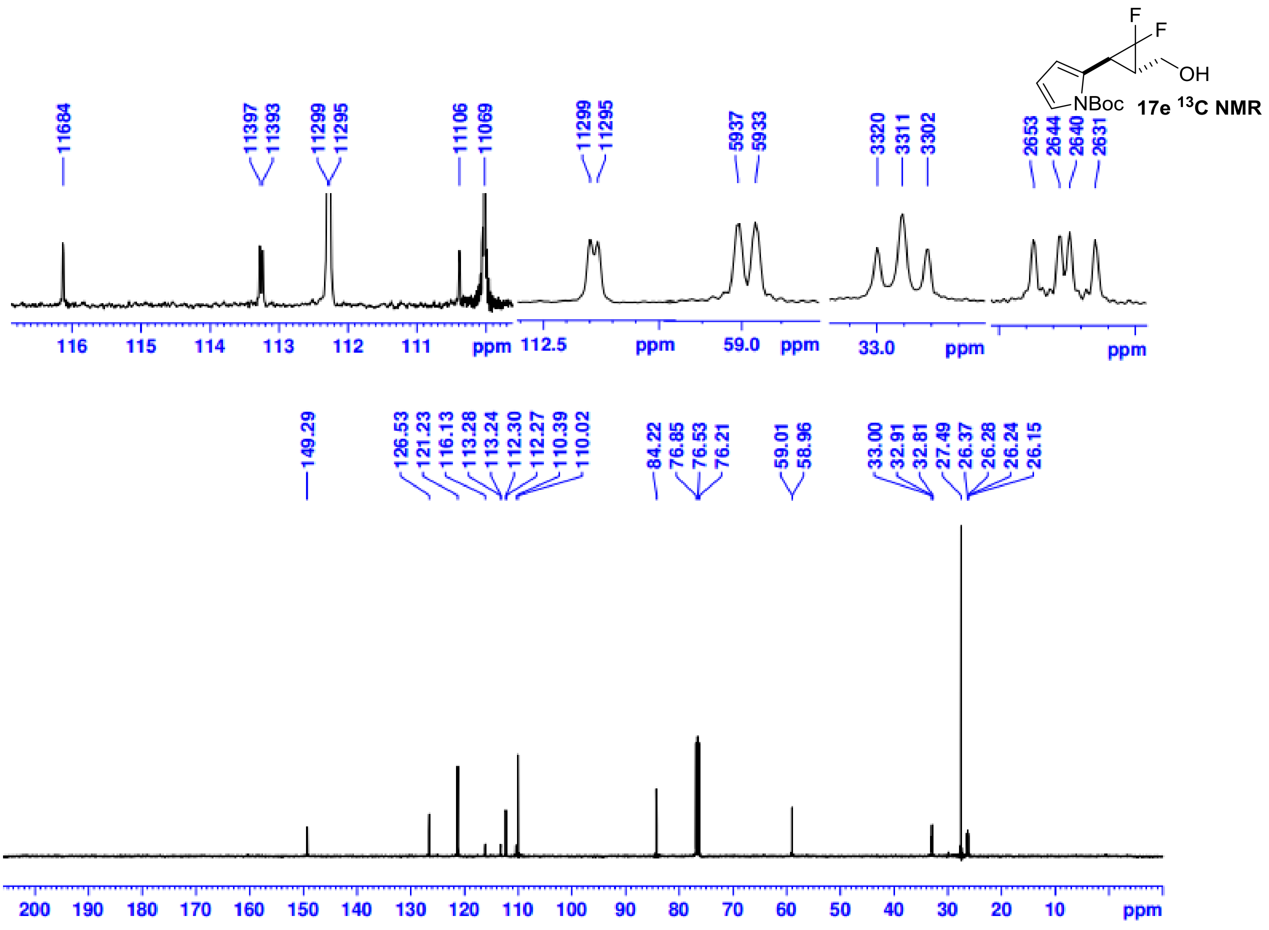
17e $^{13}$C NMR

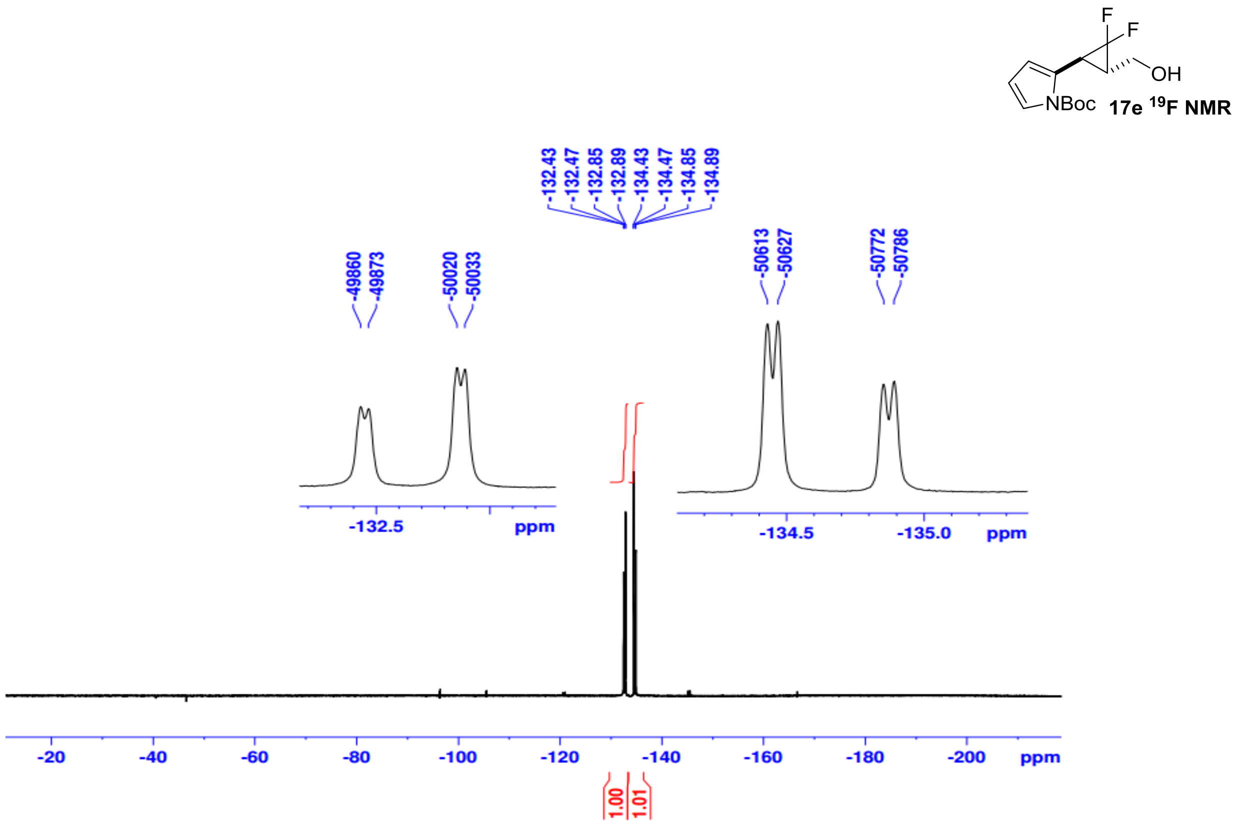
17e $^{19}$F NMR

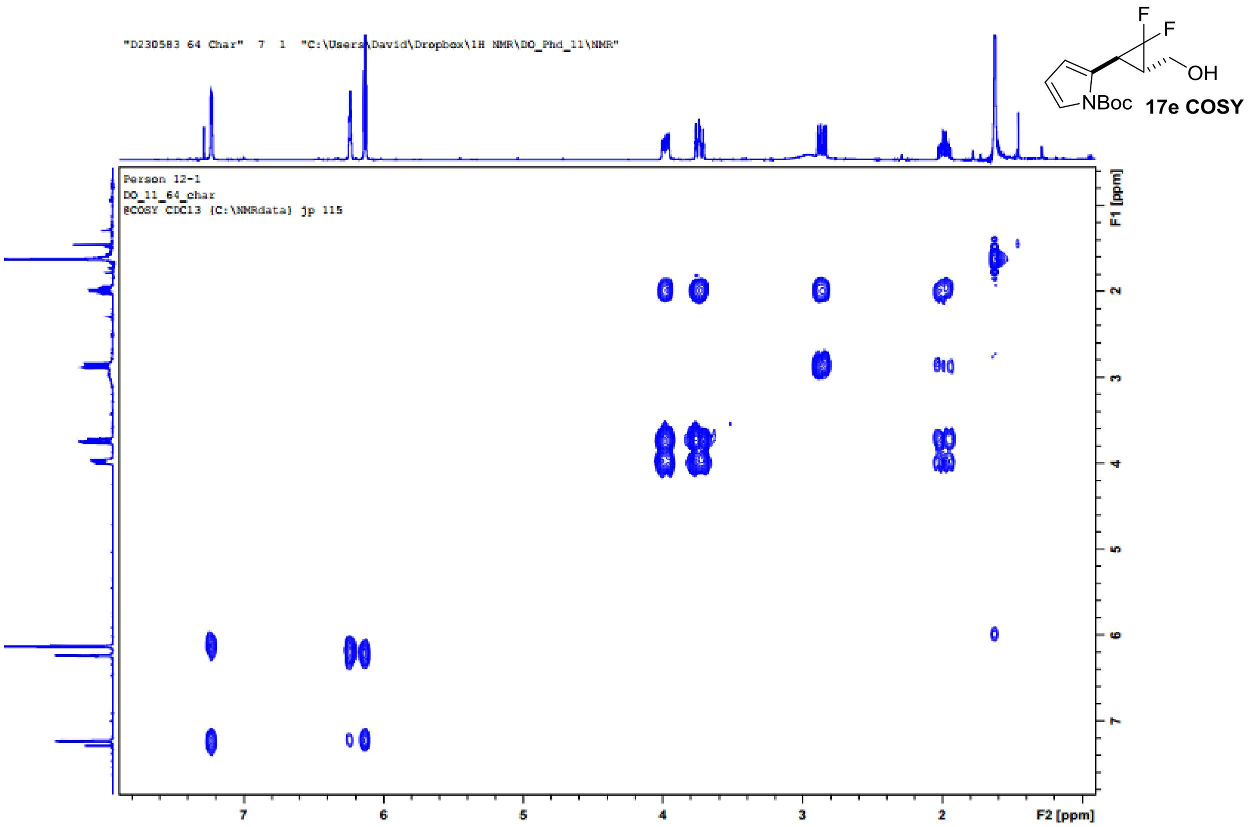


17e COSY

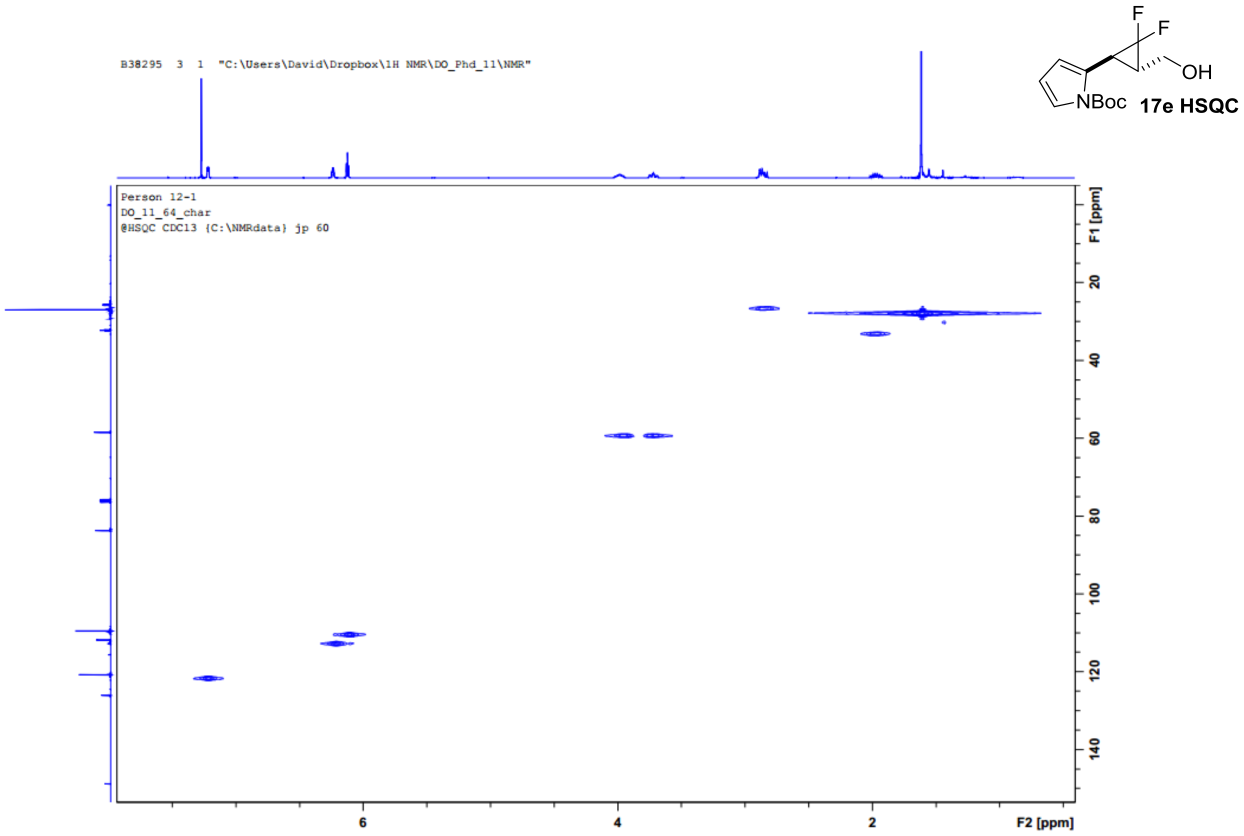


17e HSQC

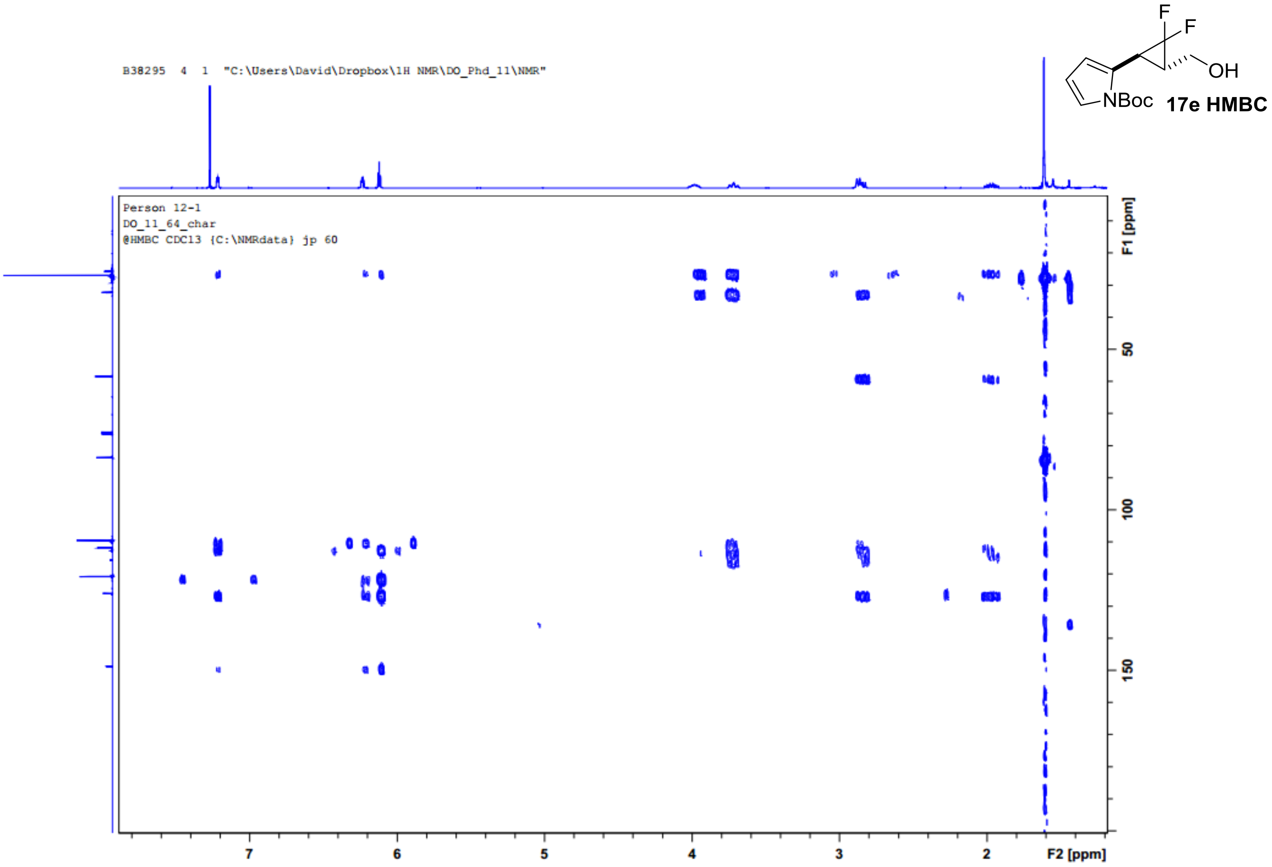


17e HMBC

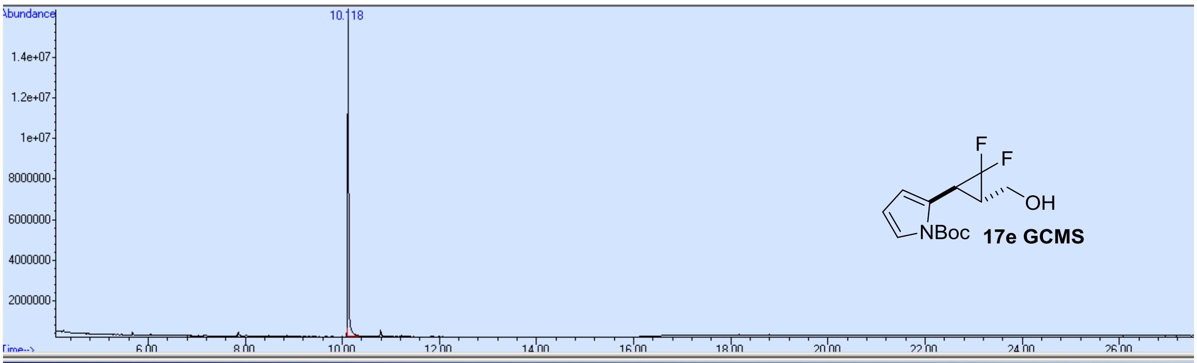


17e GCMS

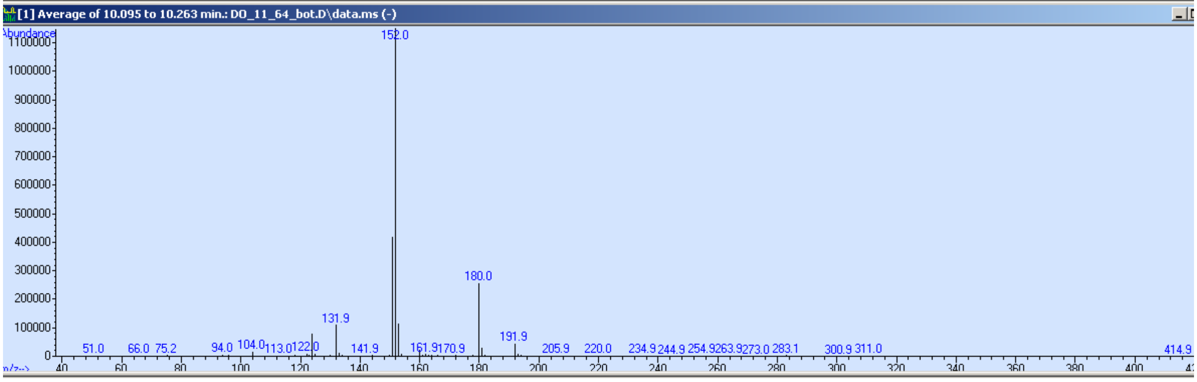

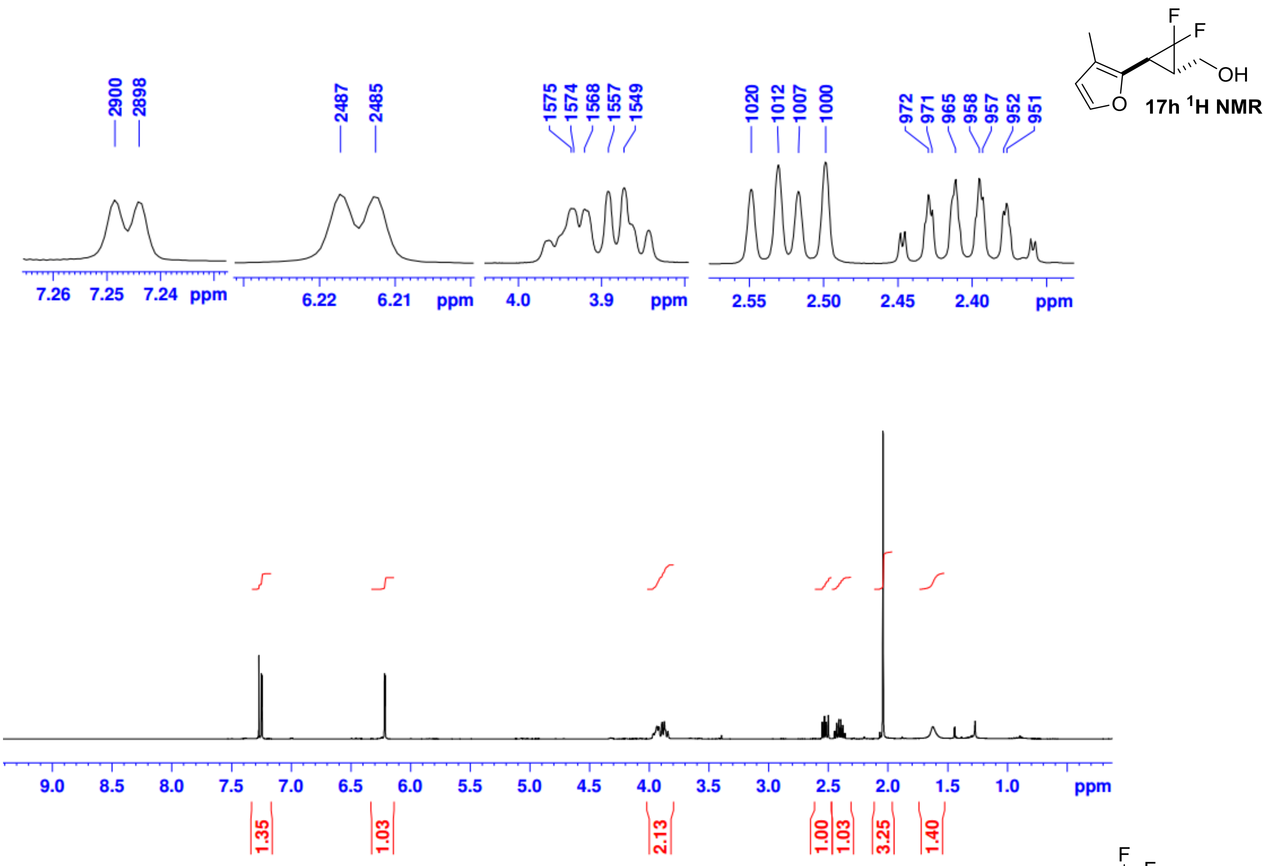
F
F
OH
O
17h ¹H NMR

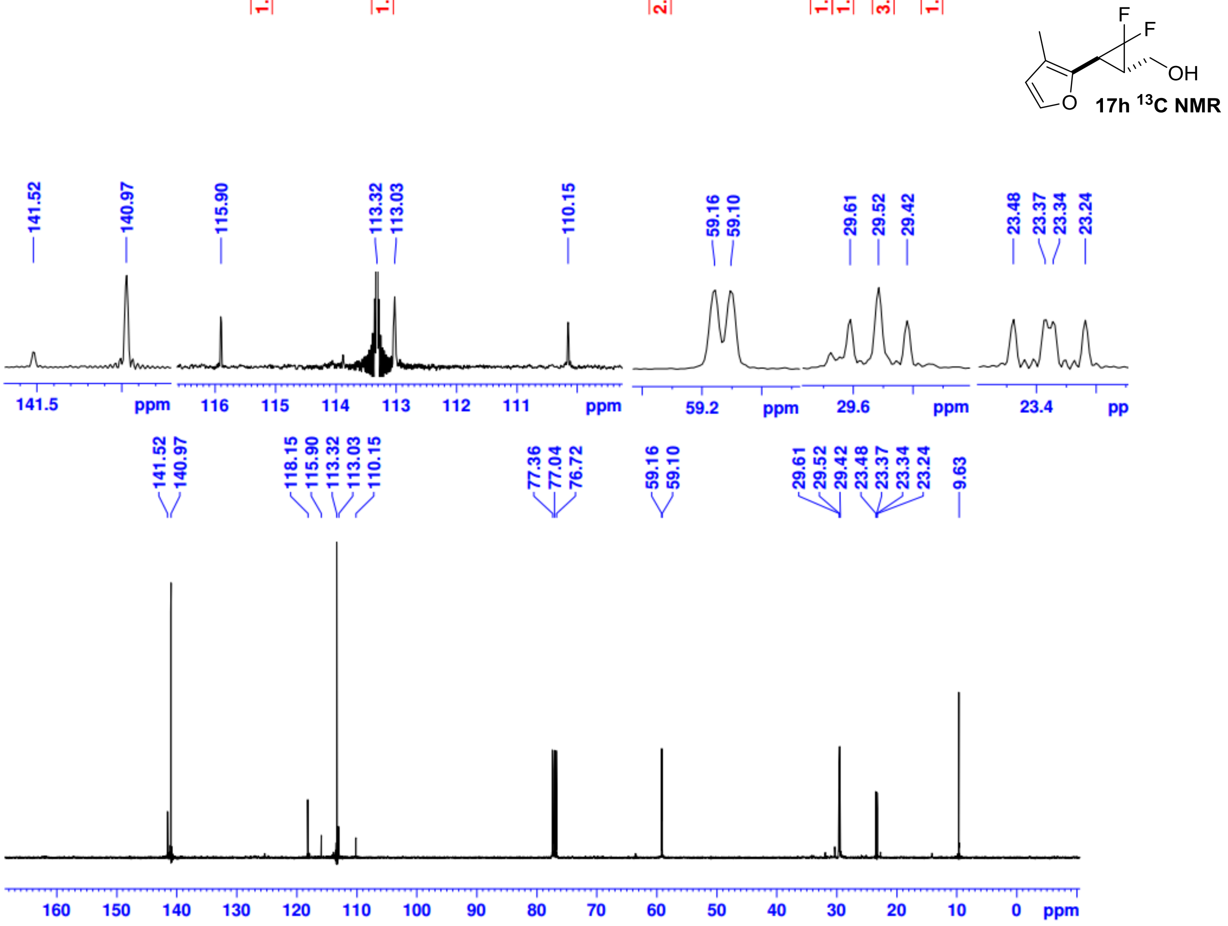
F
F
OH
O
17h ¹³C NMR

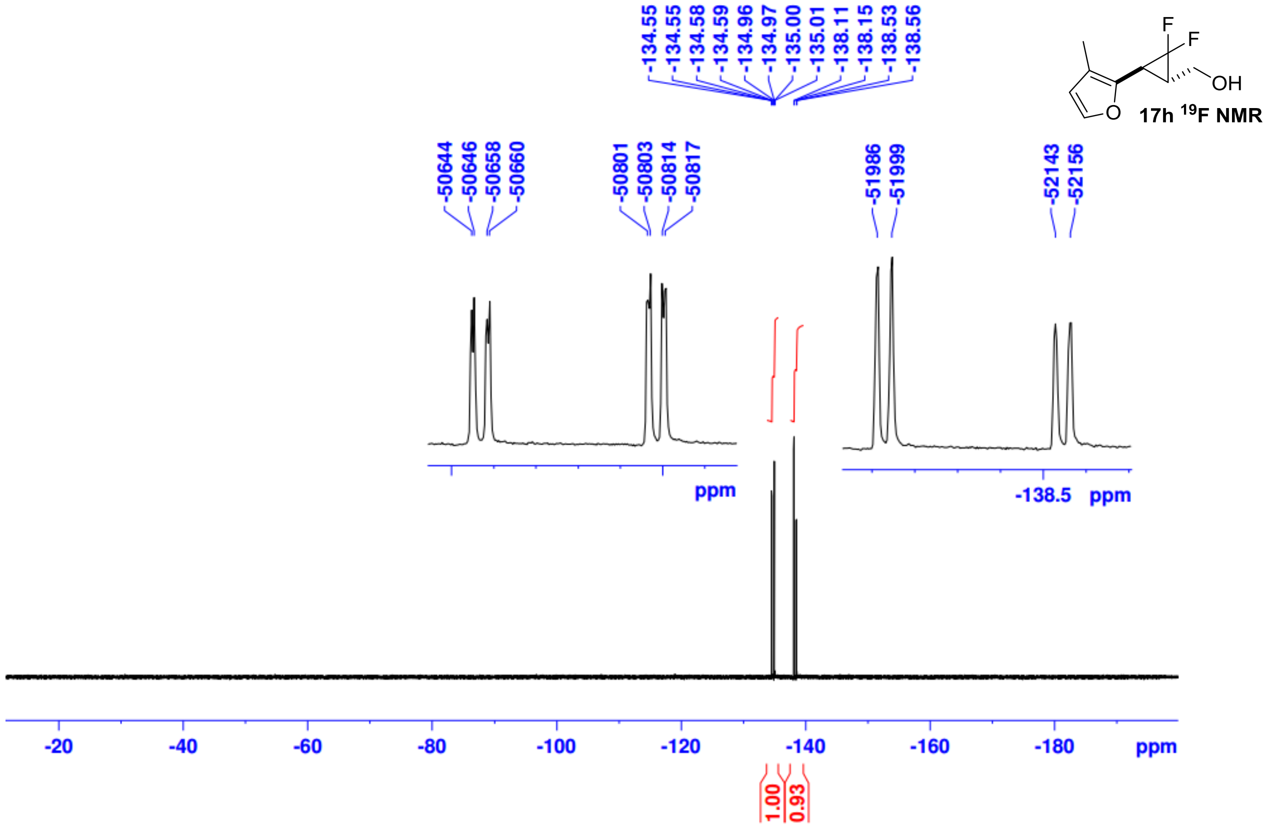
-134.55
-134.55
-134.58
-134.59
-134.96
-134.97
-135.00
-135.01
-138.11
-138.15
-138.53
-138.56
-50644
-50646
-50658
-50660
-50801
-50803
-50814
-50817
-51986
-51999
-52143
-52156
ppm
-138.5 ppm
-20
-40
-60
-80
-100
-120
-140
-160
-180
ppm
1.00
0.93
F
F
OH
O
17h $^{19}$F NMR

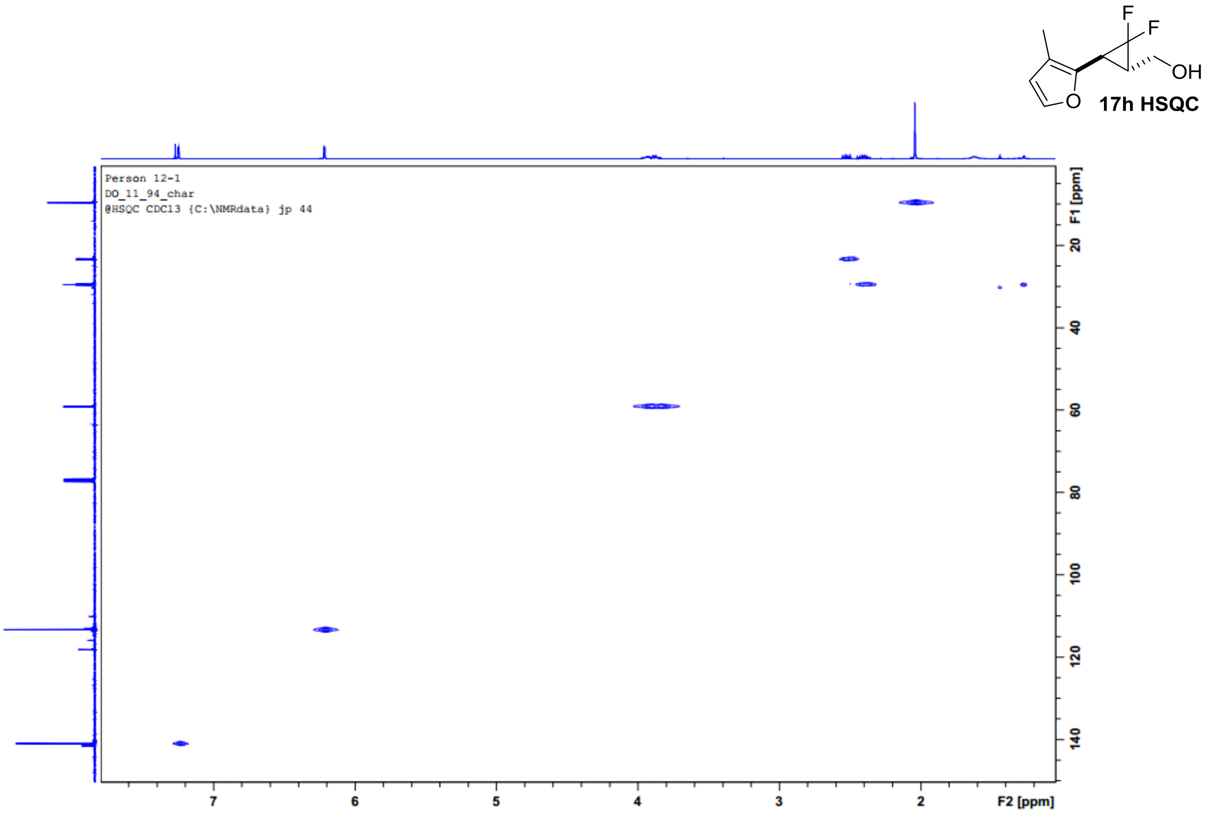
F
F
OH
O
17h HSQC
Person 12-1
DO_11_94_char
@HSQC CDCl3 {C:\NMRdata} jp 44
F1 [ppm]
20
40
60
80
100
120
140
7
6
5
4
3
2
F2 [ppm]

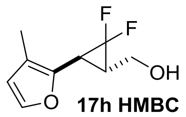

**17h HMBC**

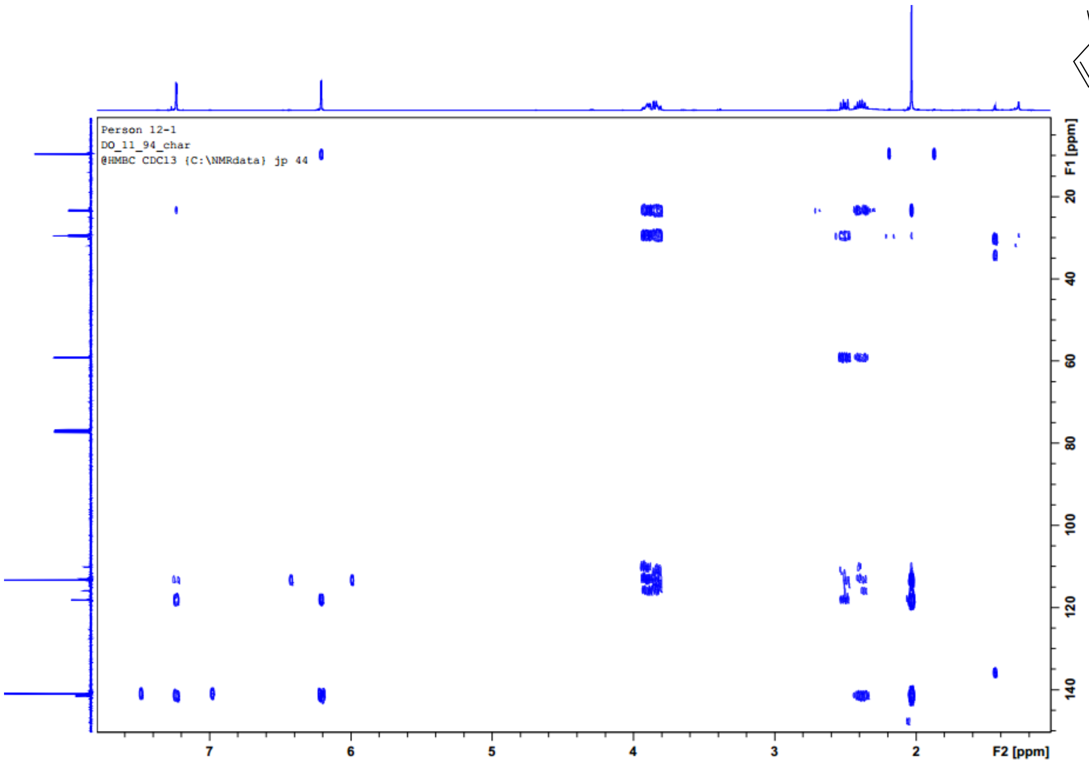


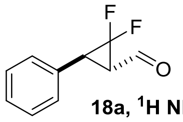

**18a, $^{1}$H NMR**

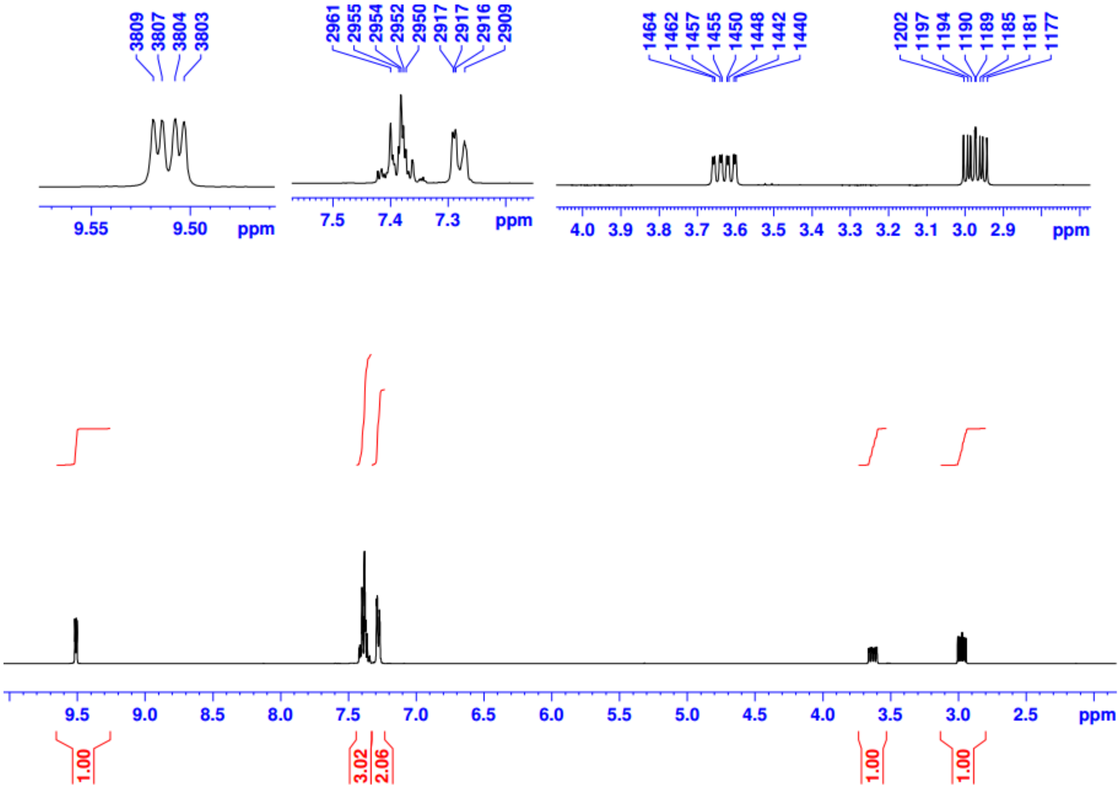

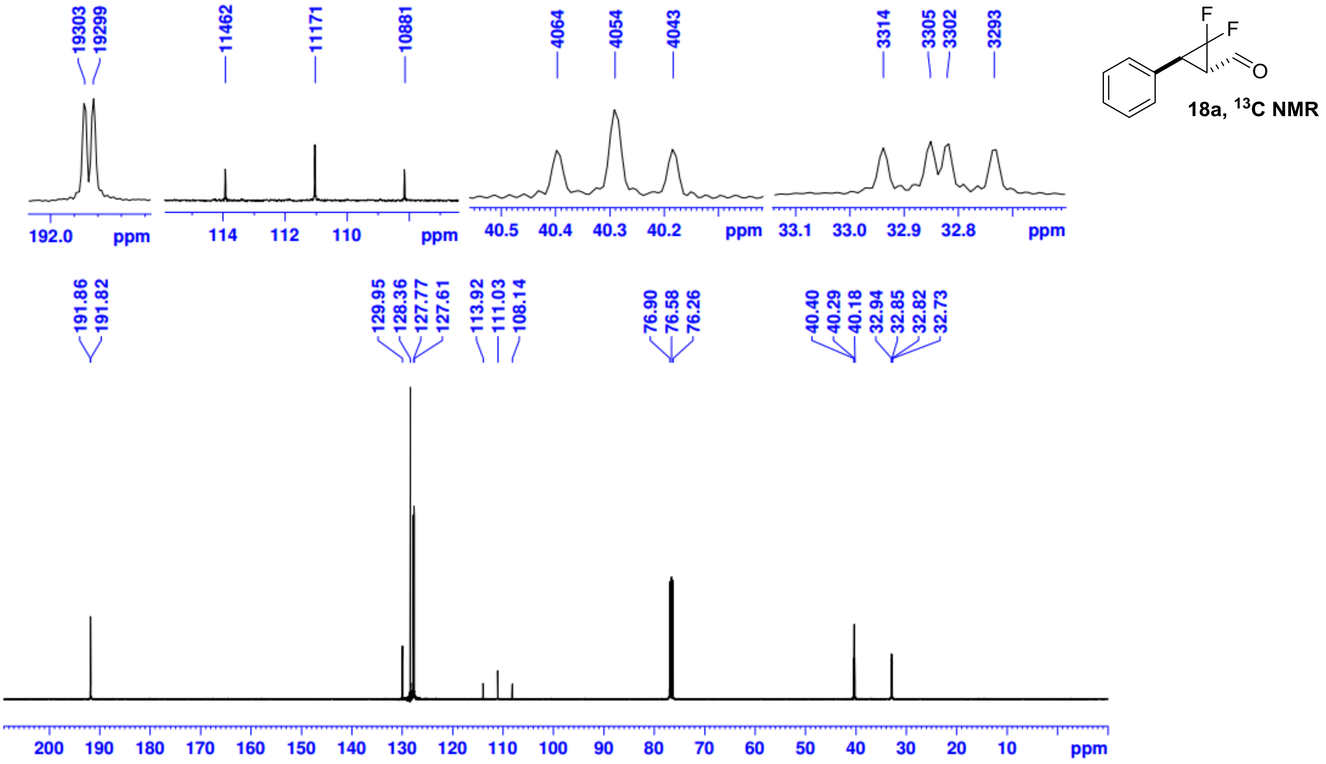


18a, $^{13}$C NMR

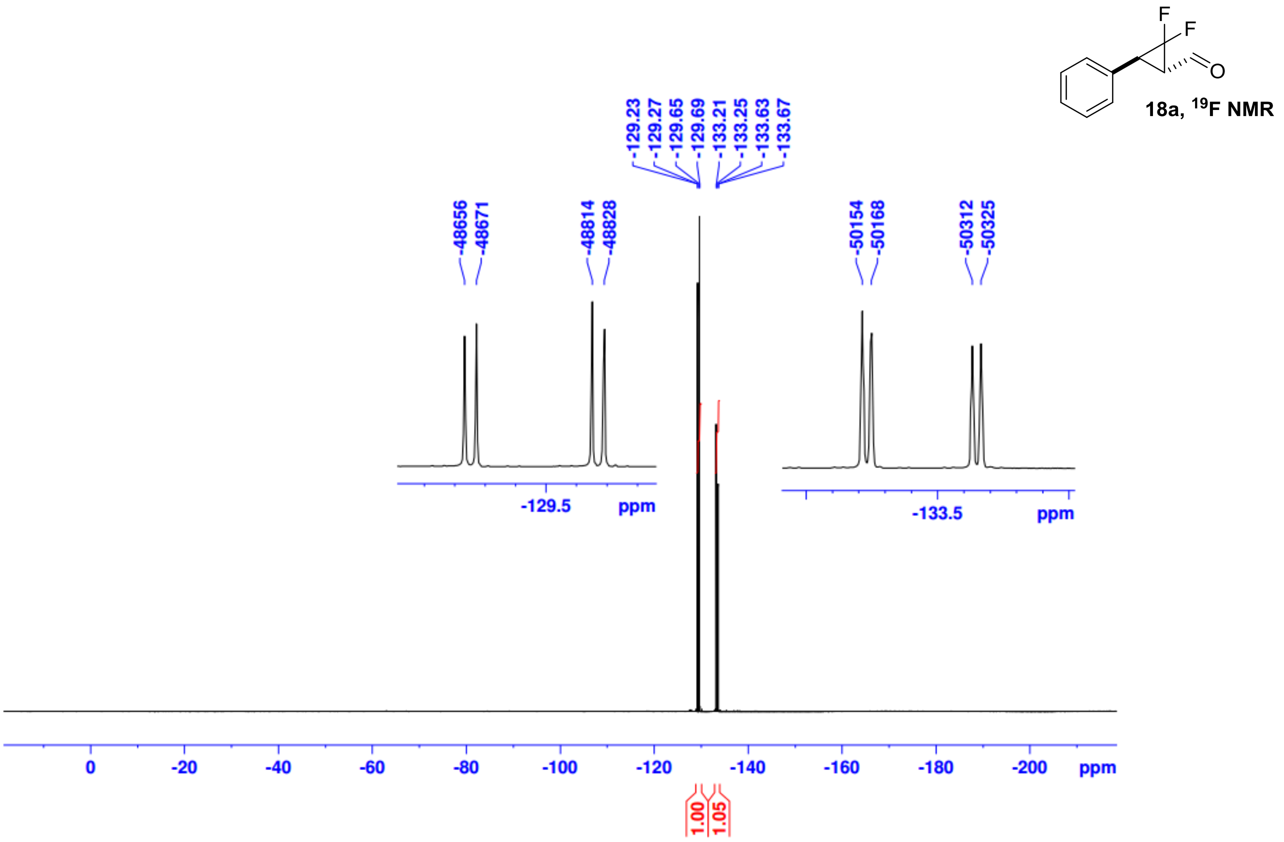


18a, $^{19}$F NMR

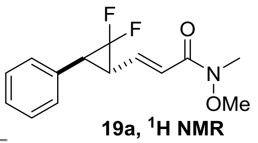


19a, $^{1}$H NMR

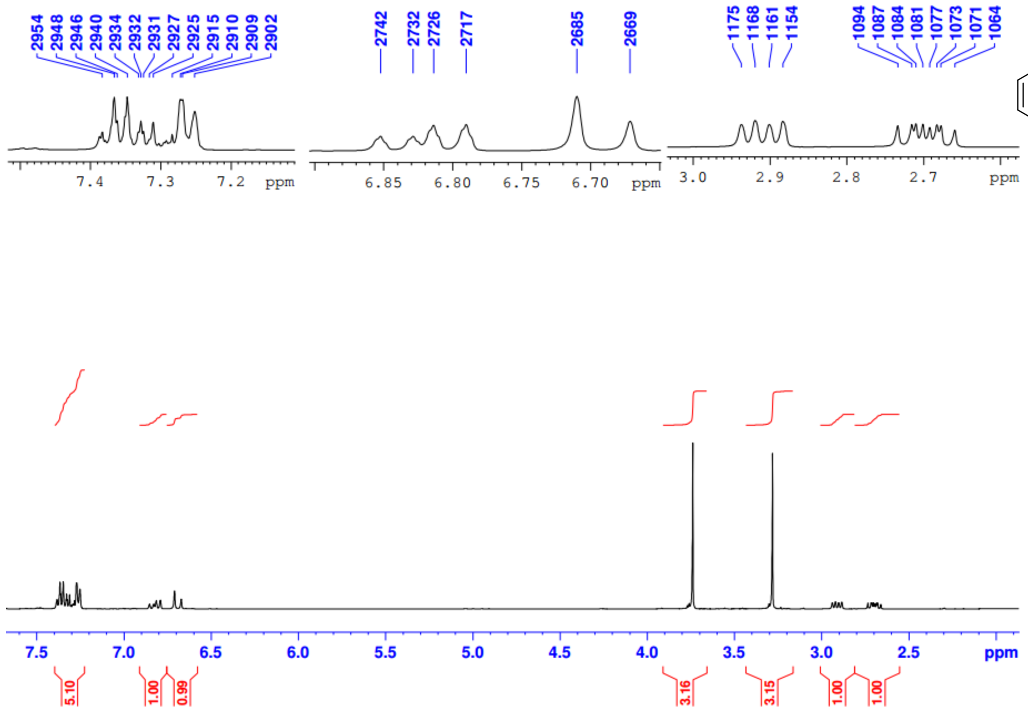


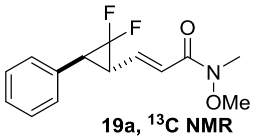


19a, $^{13}$C NMR

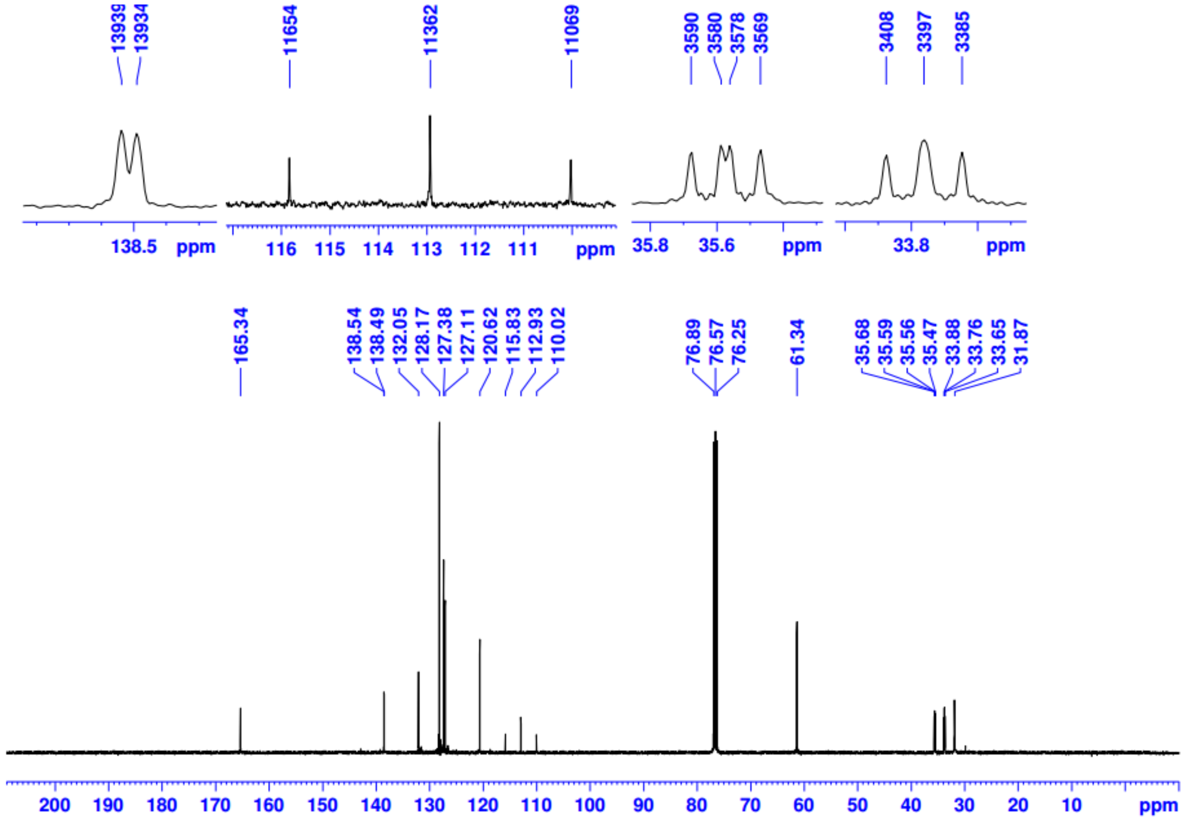

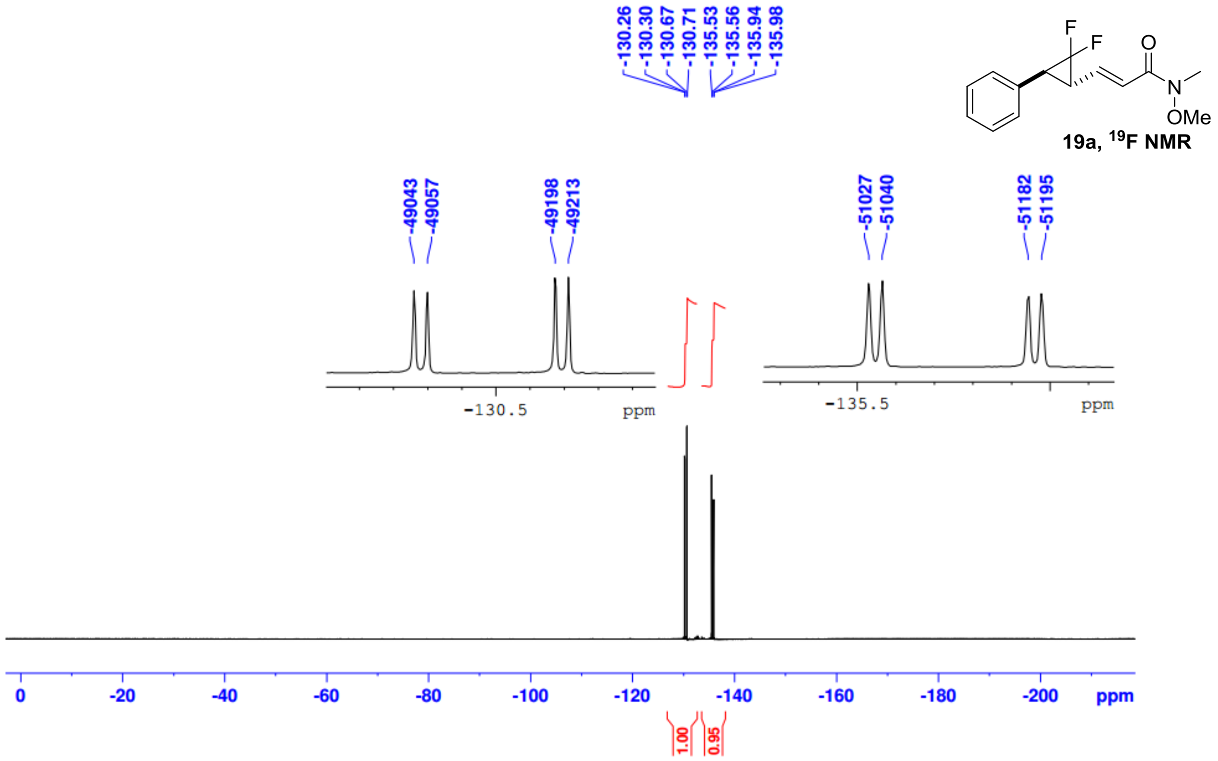

-130.26
-130.30
-130.67
-130.71
-135.53
-135.56
-135.94
-135.98
-49043
-49057
-49198
-49213
-51027
-51040
-51182
-51195
-130.5
ppm
-135.5
ppm
0
-20
-40
-60
-80
-100
-120
-140
-160
-180
-200
ppm
1.00
0.95
F
F
O
N
OMe
19a, 19F NMR


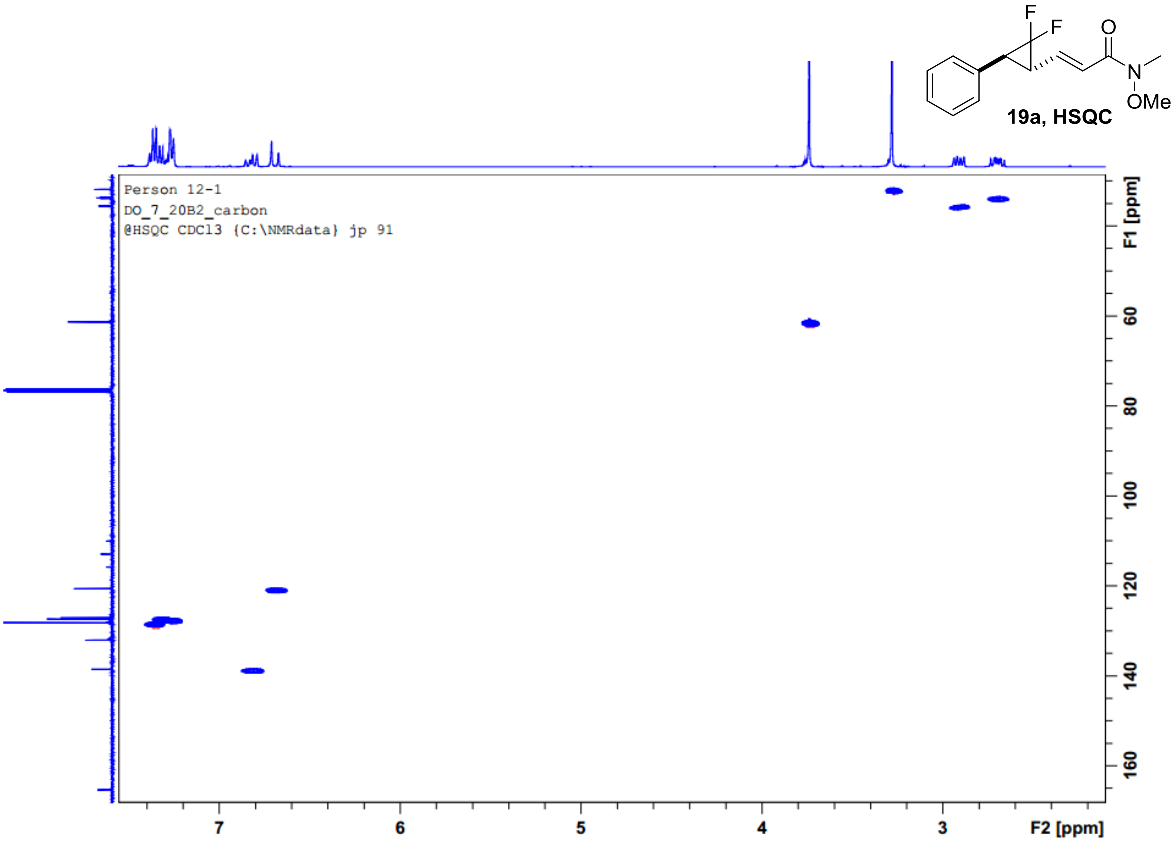

F
F
O
N
OMe
19a, HSQC
Person 12-1
DO_7_20B2_carbon
@HSQC CDCl3 {C:\NMRdata} jp 91
F1 [ppm]
60
80
100
120
140
160
7
6
5
4
3
F2 [ppm]

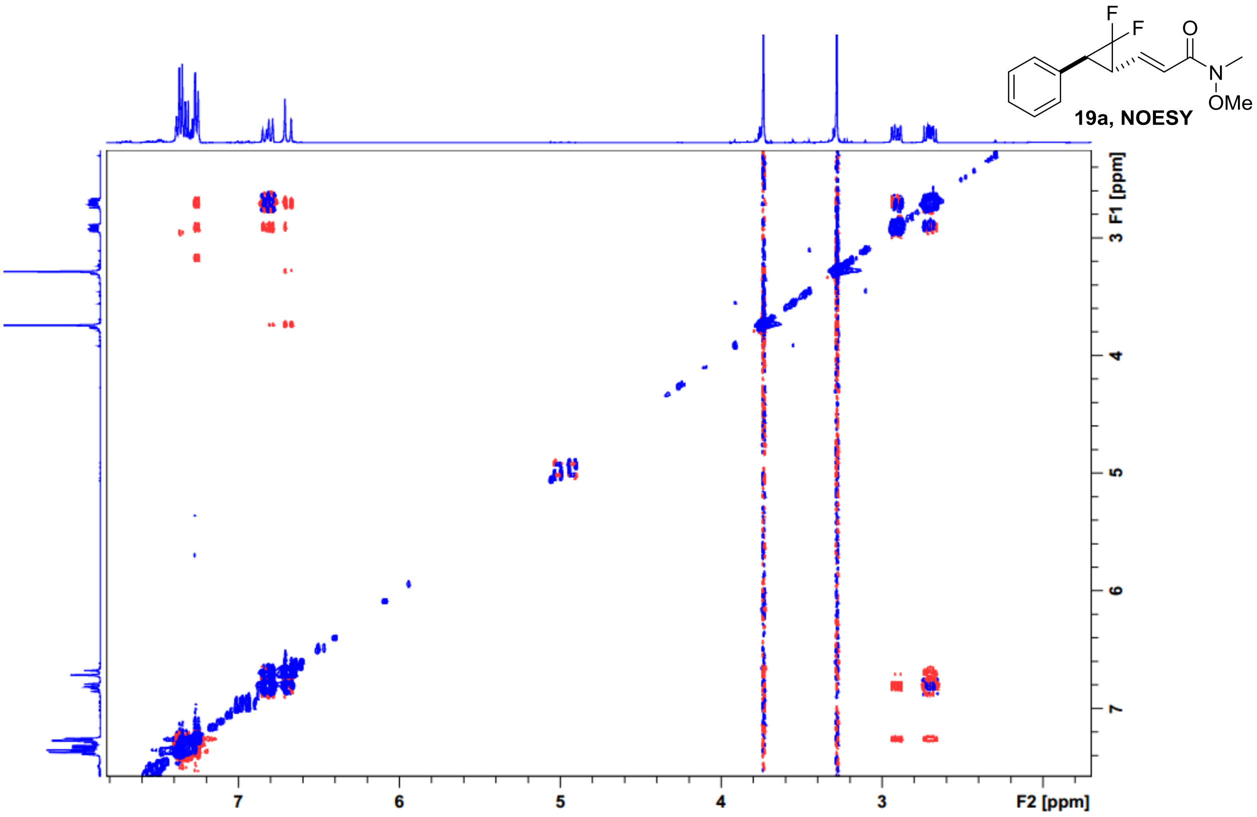
19a, NOESY
F
F
O
N
OMe
F1 [ppm]
F2 [ppm]
7
6
5
4
3

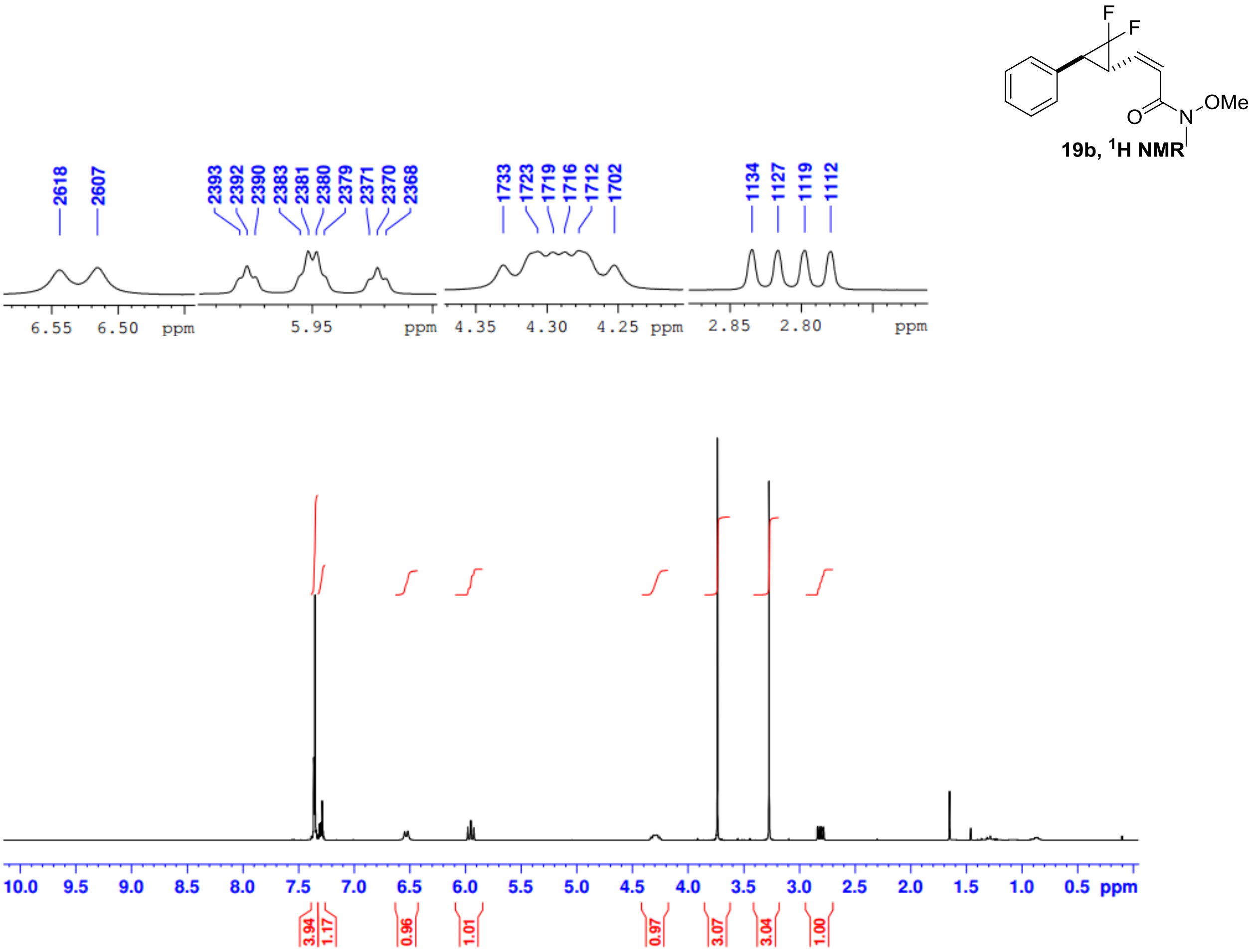
19b, ¹H NMR
F
F
OMe
O
N
2618
2607
2393
2392
2390
2383
2381
2380
2379
2371
2370
2368
1733
1723
1719
1716
1712
1702
1134
1127
1119
1112
6.55
6.50
ppm
5.95
ppm
4.35
4.30
4.25
ppm
2.85
2.80
ppm
10.0
9.5
9.0
8.5
8.0
7.5
7.0
6.5
6.0
5.5
5.0
4.5
4.0
3.5
3.0
2.5
2.0
1.5
1.0
0.5
ppm
3.94
1.17
0.96
1.01
0.97
3.07
3.04
1.00

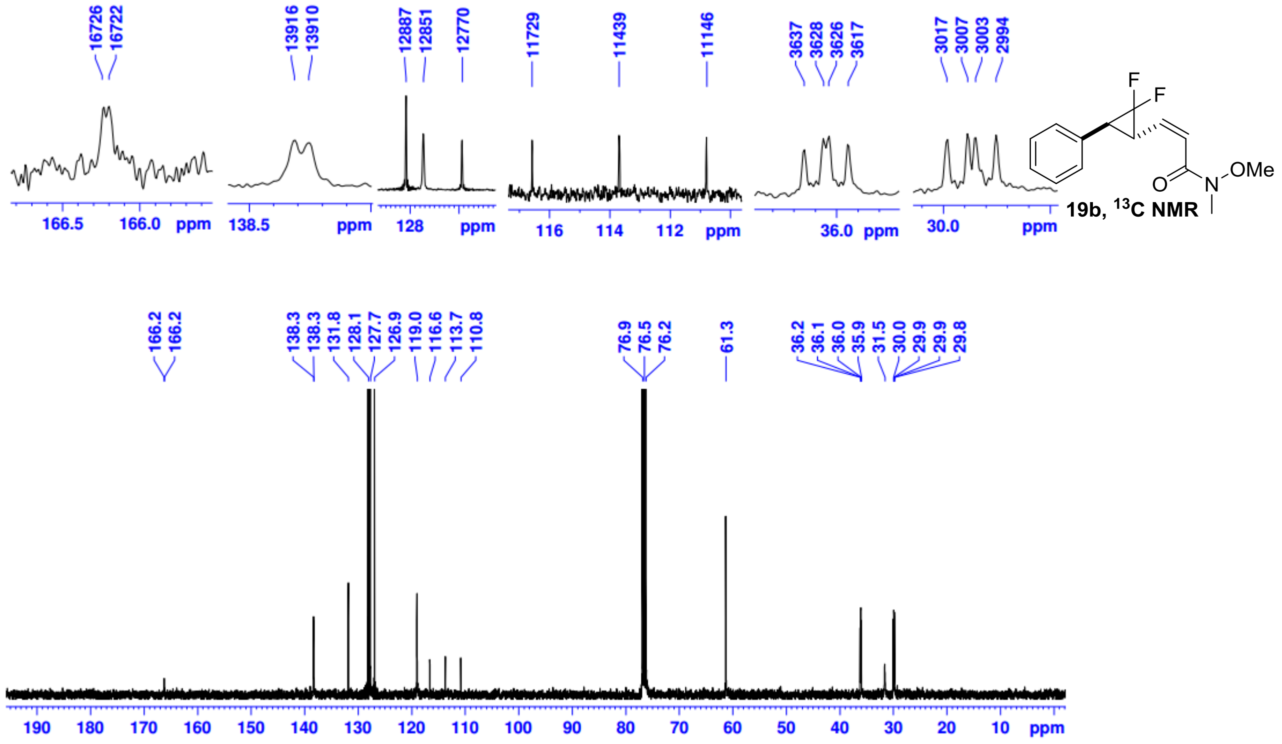
19b, ^13C NMR

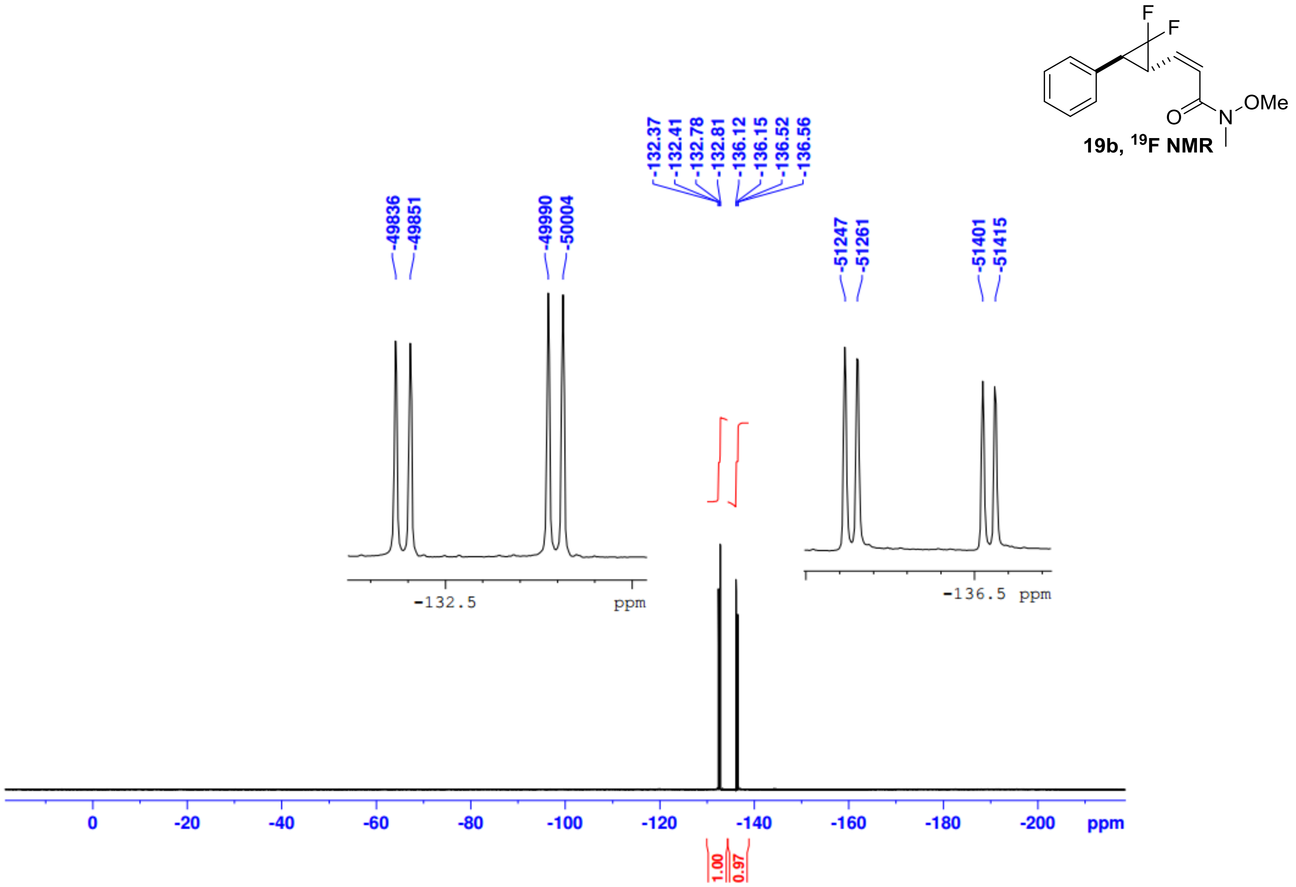
19b, ^19F NMR

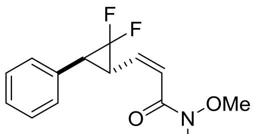


**19b, HSQC**

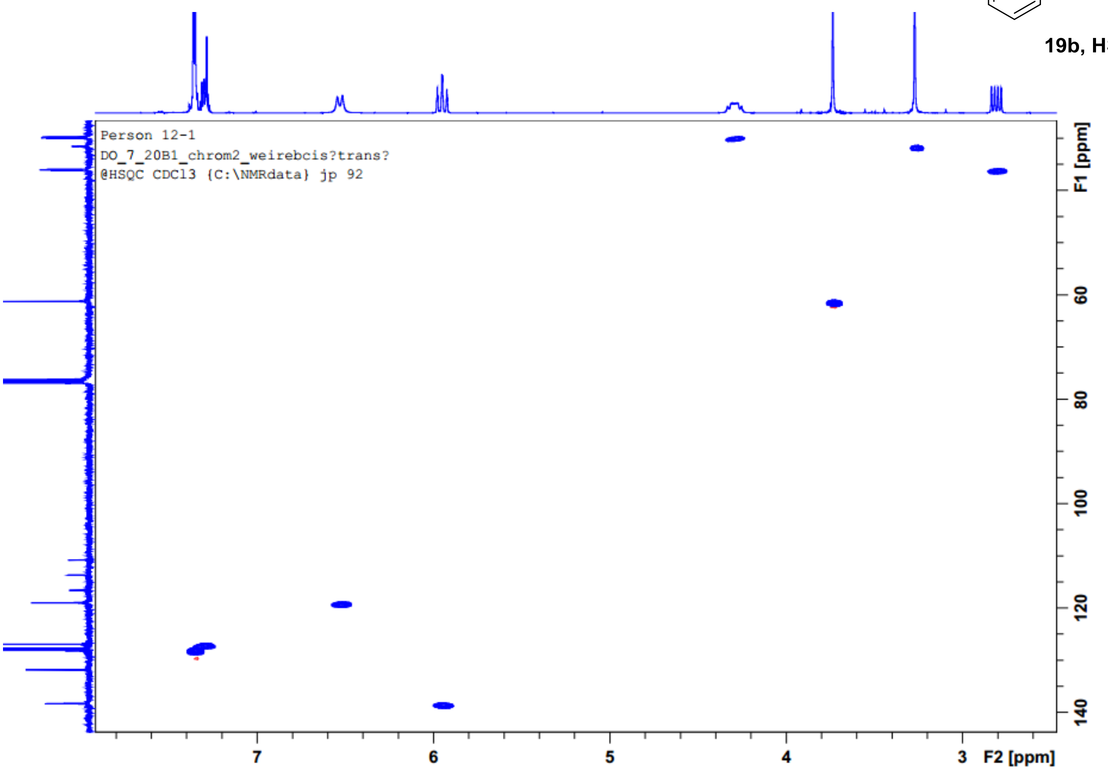


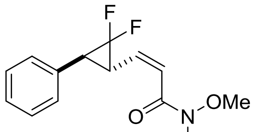


**19b, NOESY**

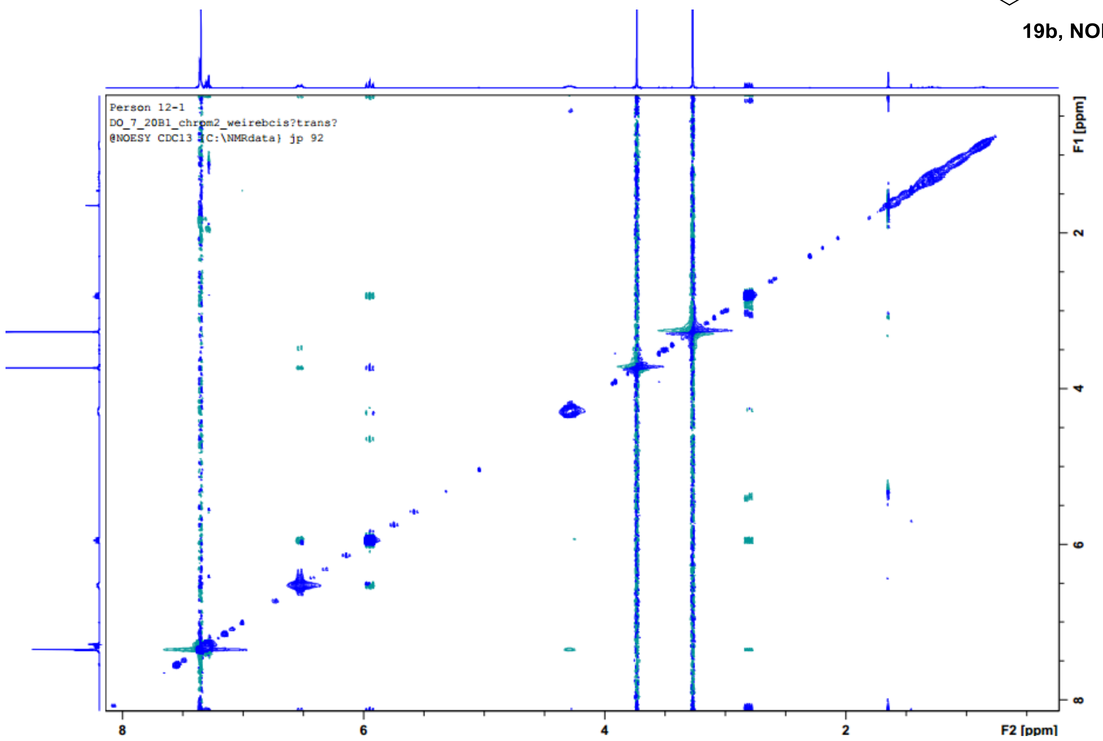

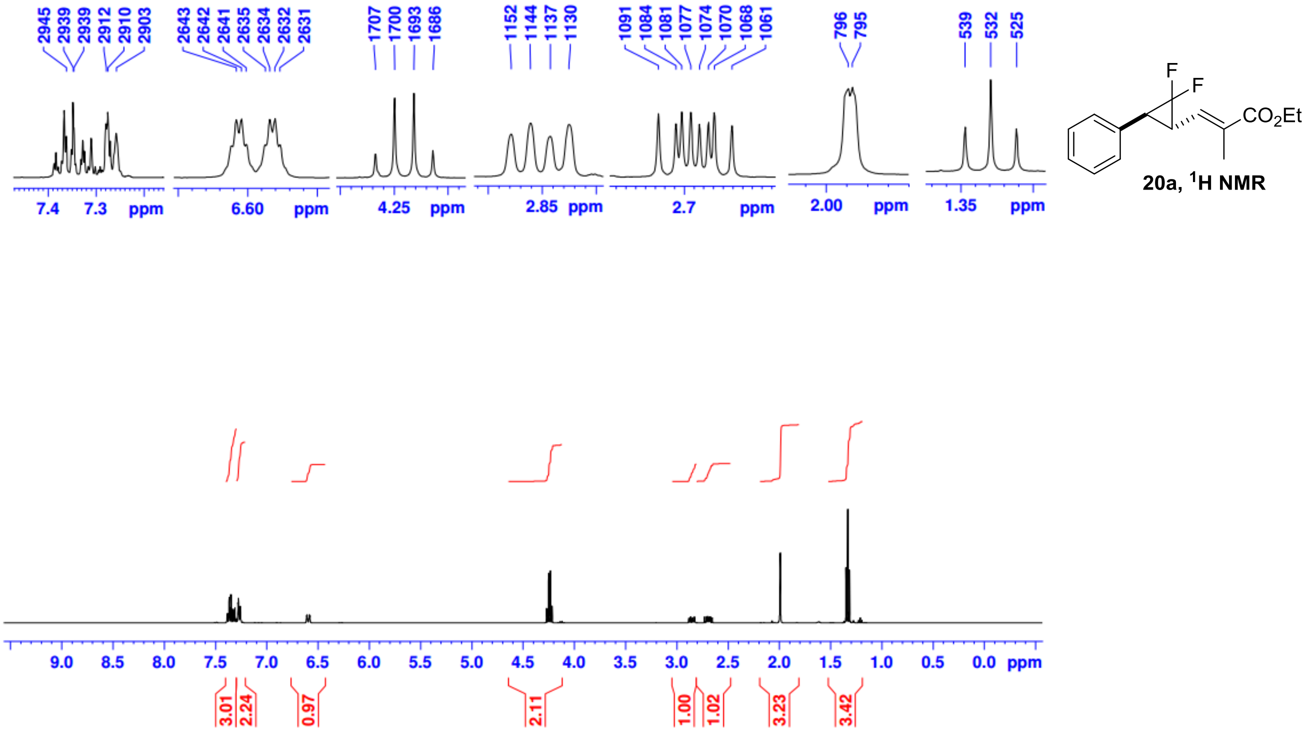


**20a, $^{1}$H NMR**

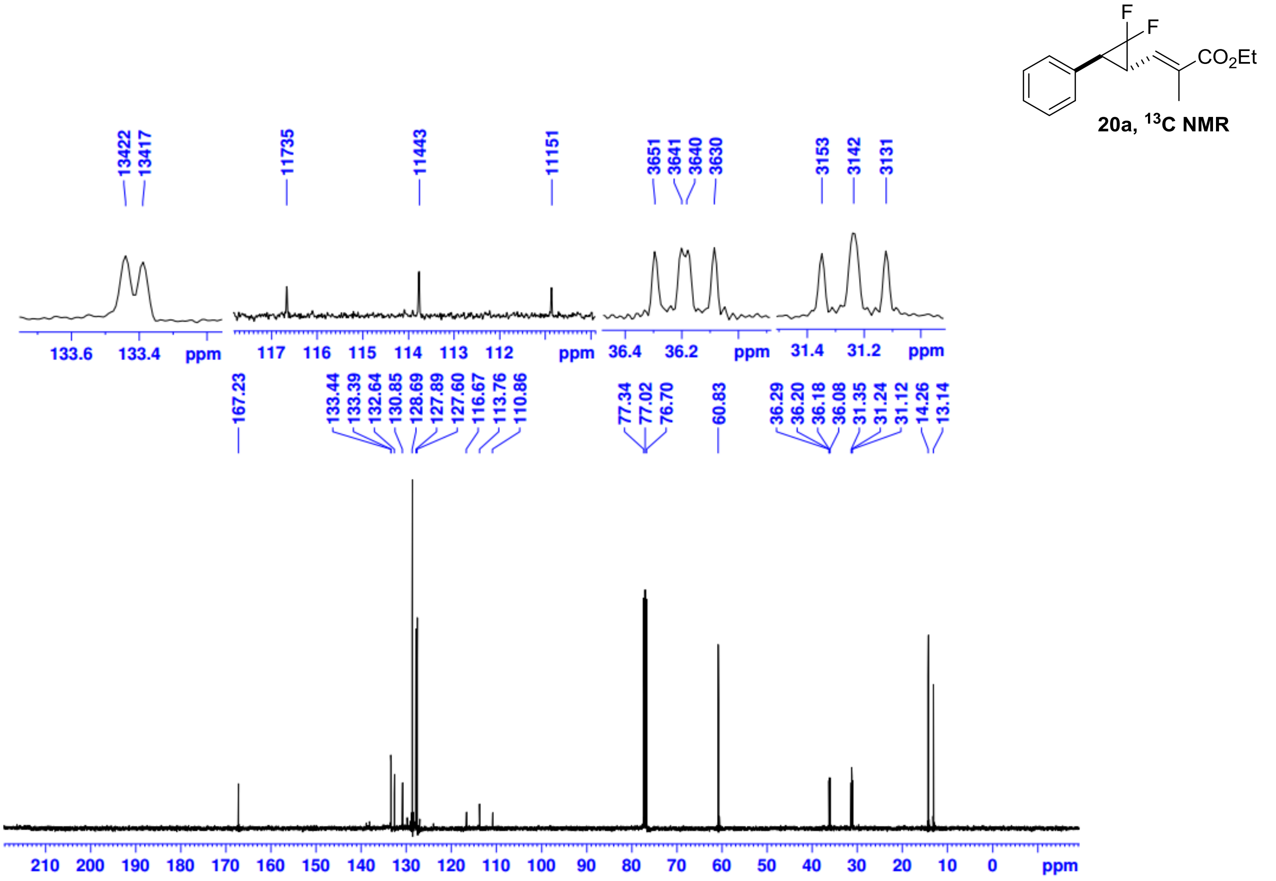


**20a, $^{13}$C NMR**

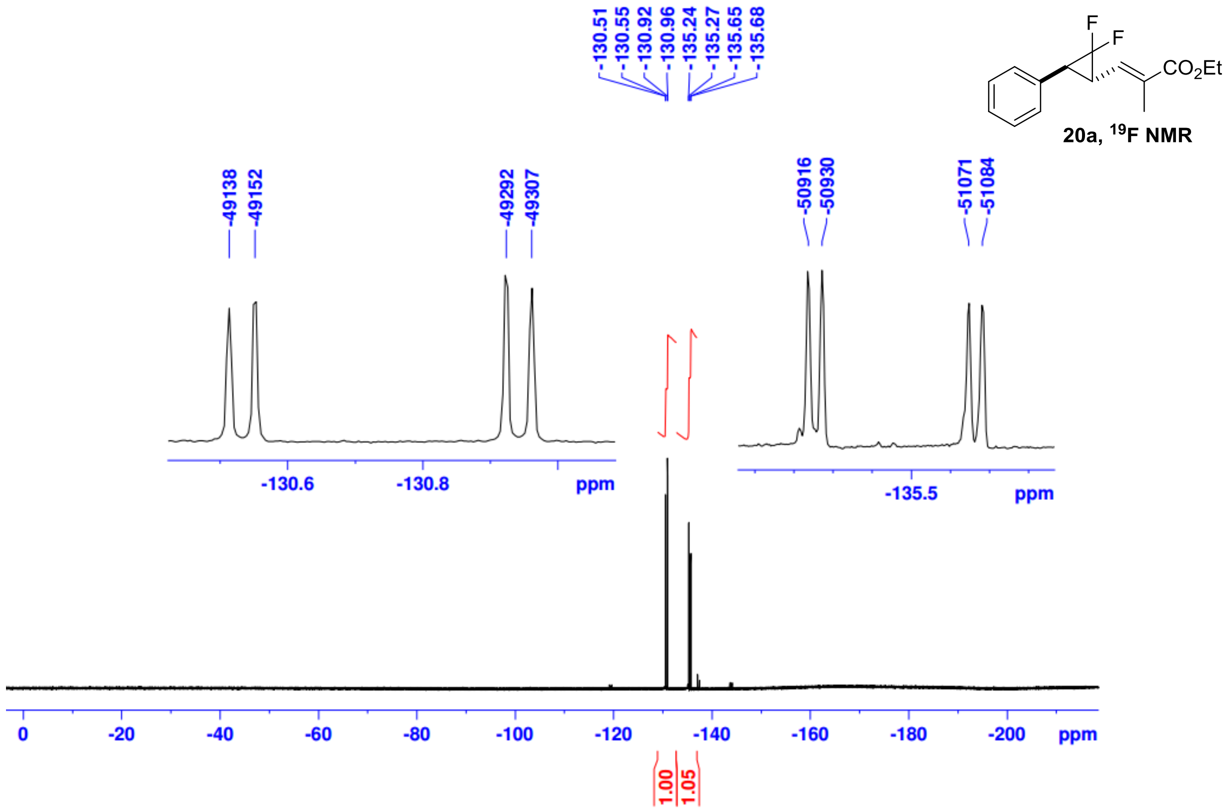

20a, ¹⁹F NMR
-130.51
-130.55
-130.92
-130.96
-135.24
-135.27
-135.65
-135.68
-49138
-49152
-49292
-49307
-50916
-50930
-51071
-51084
-130.6
-130.8
-135.5
ppm
1.00
1.05


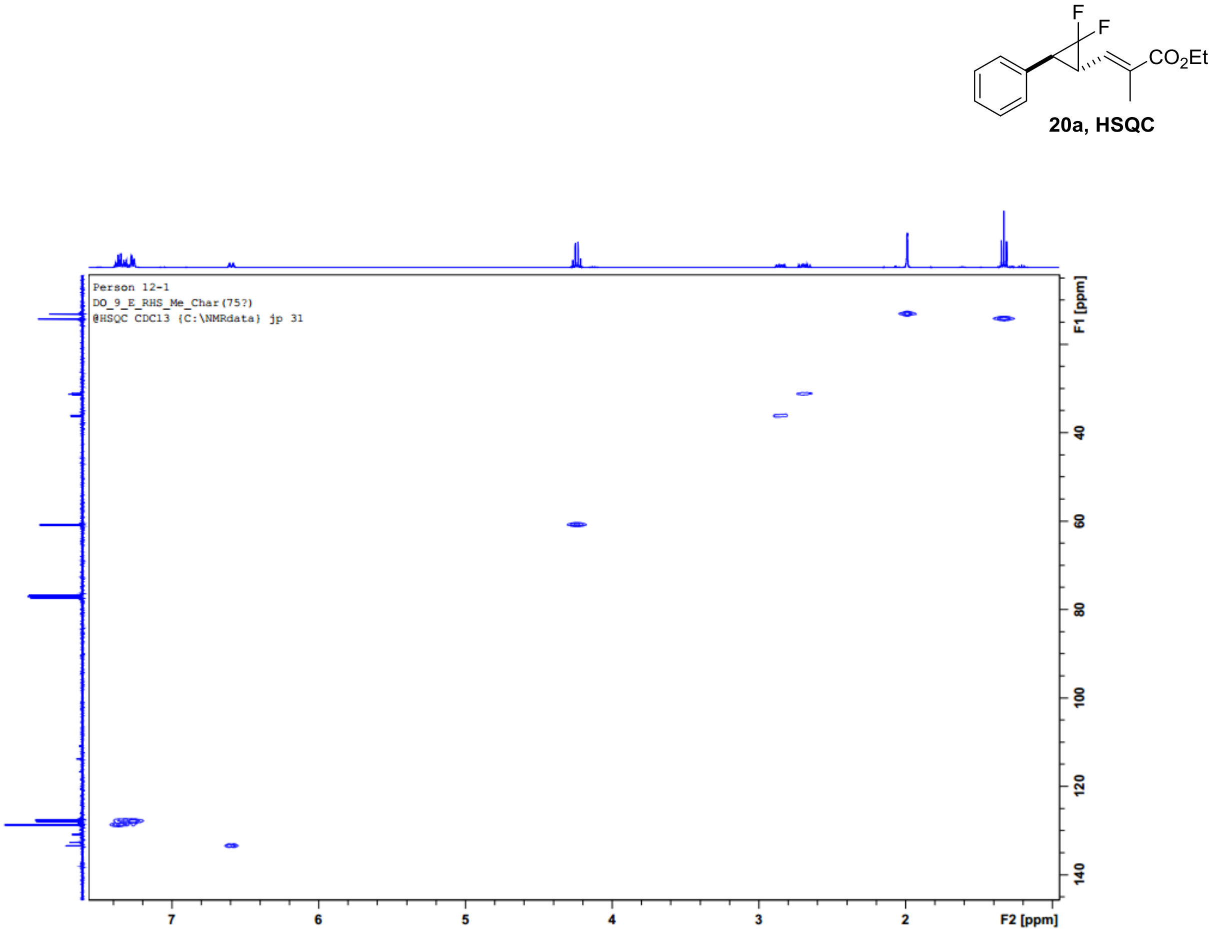

20a, HSQC
Person 12-1
DO_9_E_RHS_Me_Char(75?)
@HSQC CDCl3 {C:\NMRdata} jp 31
F1 [ppm]
F2 [ppm]


C

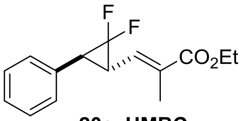


**20a, HMBC**

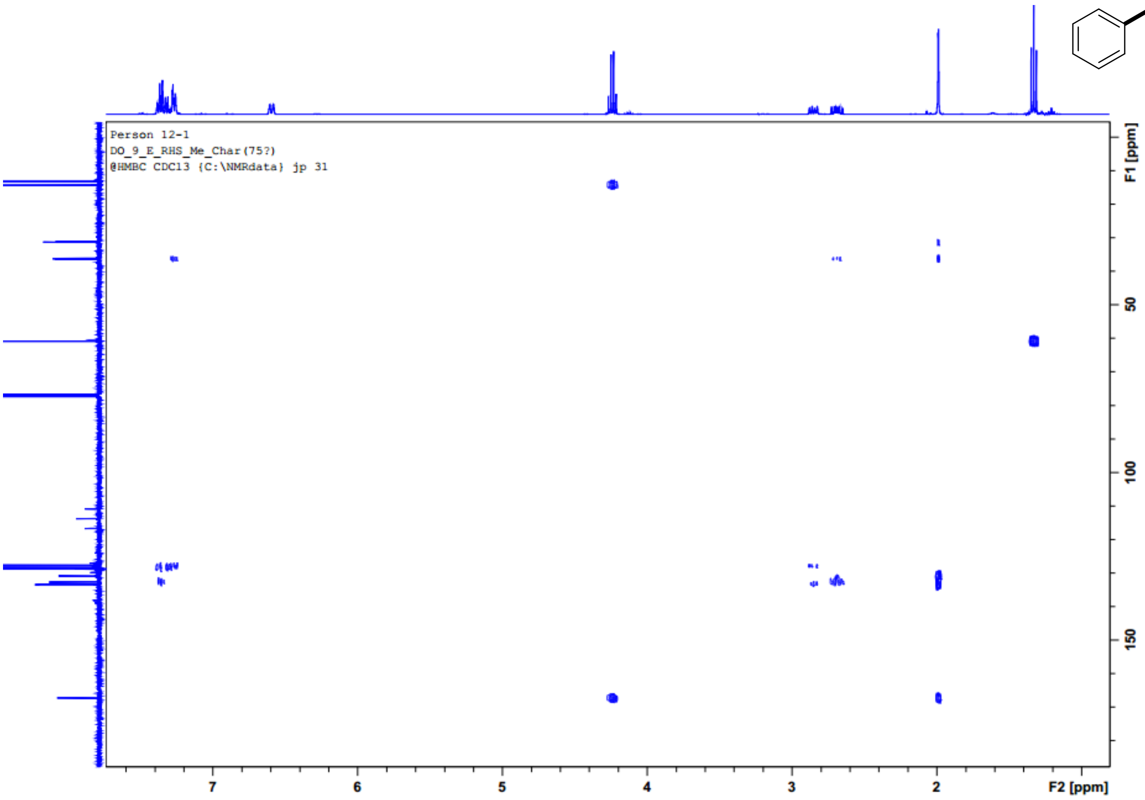


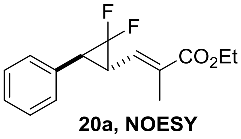


**20a, NOESY**

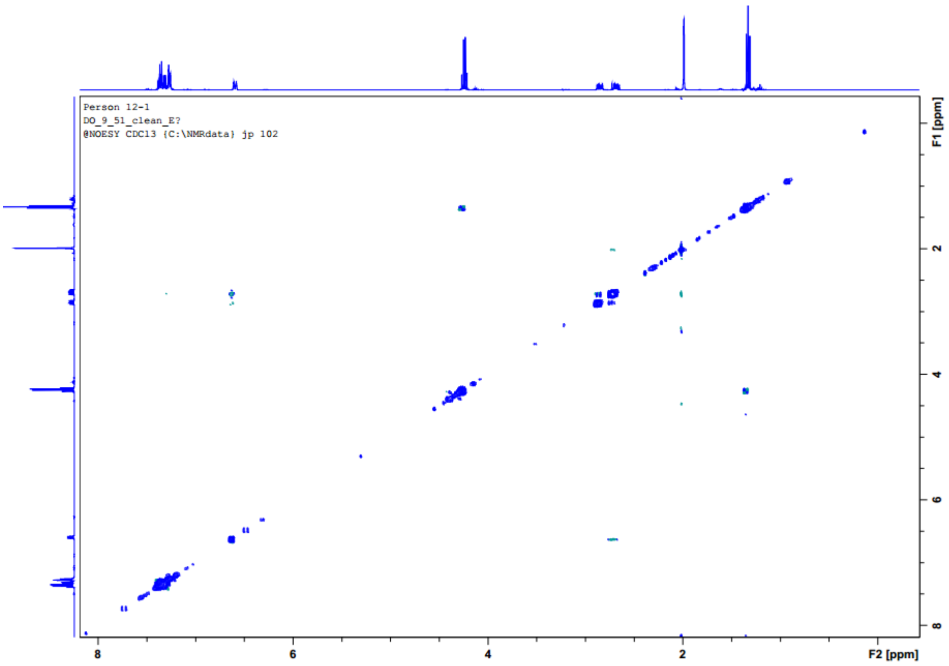

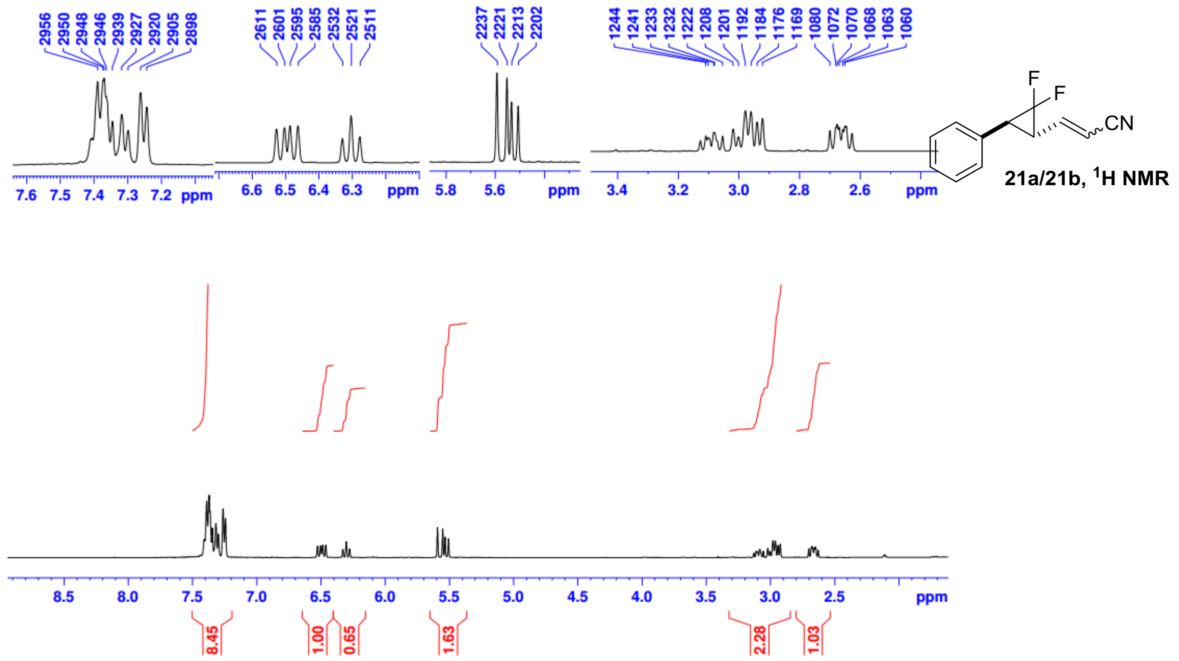
21a/21b, ¹H NMR

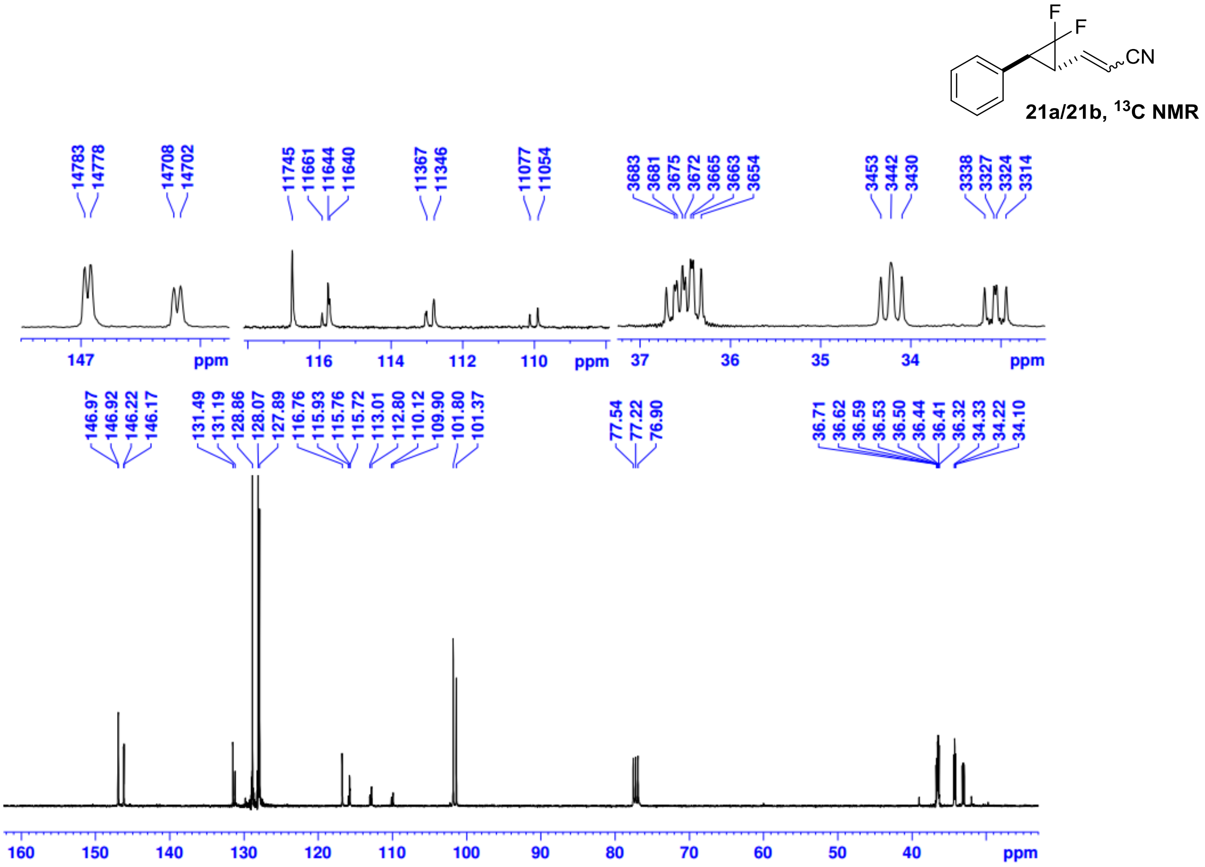
21a/21b, ¹³C NMR

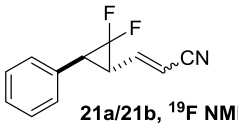

21a/21b, $^{19}F$ NMR

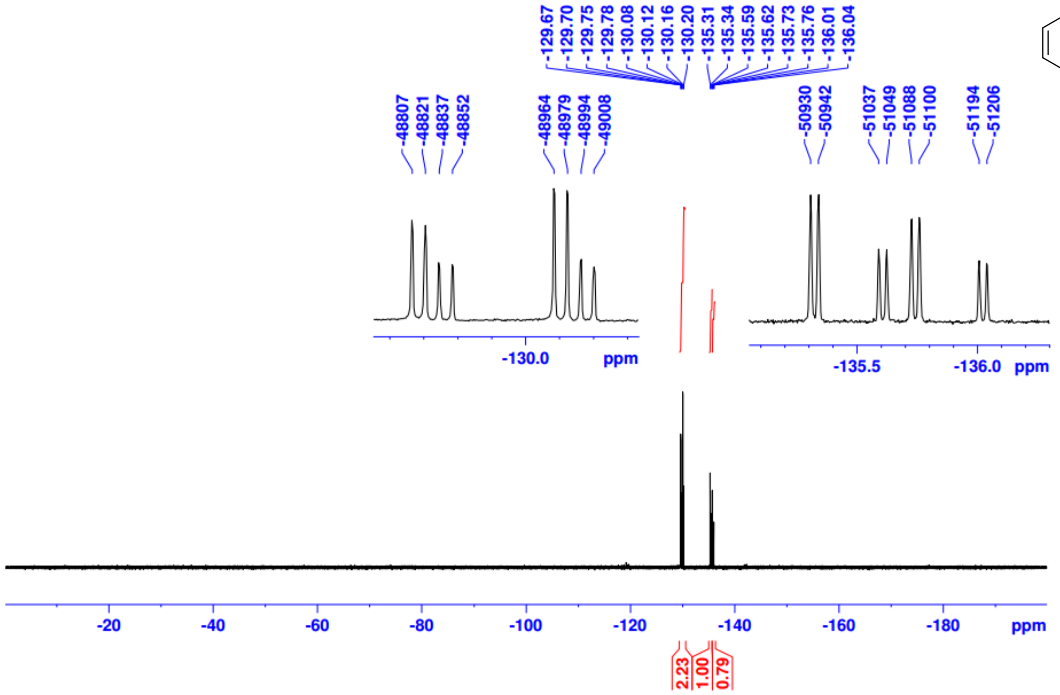

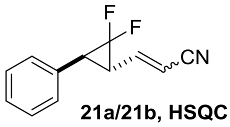

21a/21b, HSQC

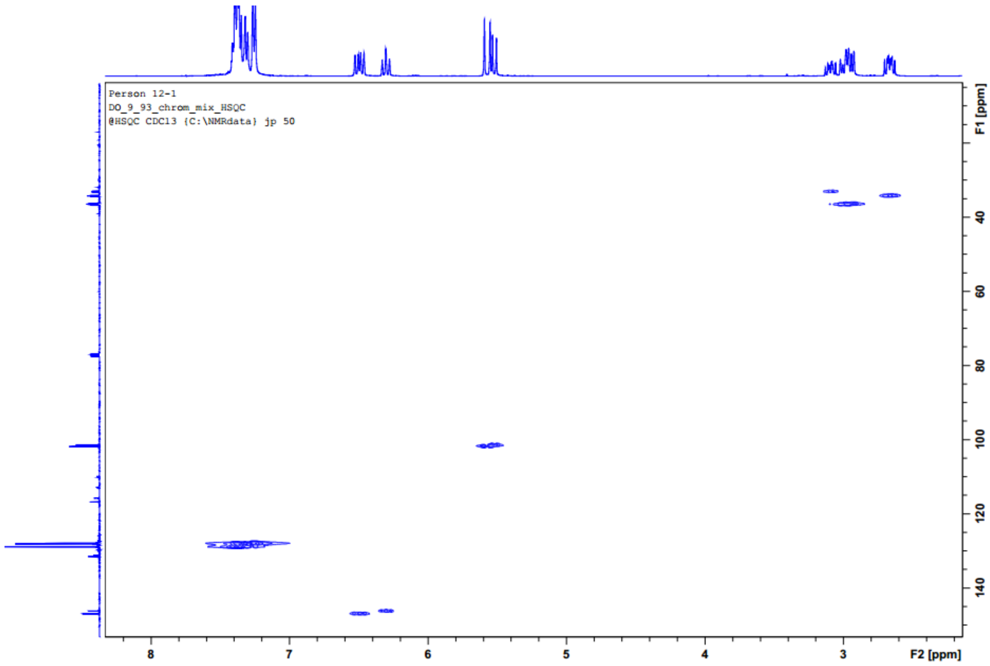

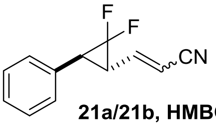

**21a/21b, HMBC**

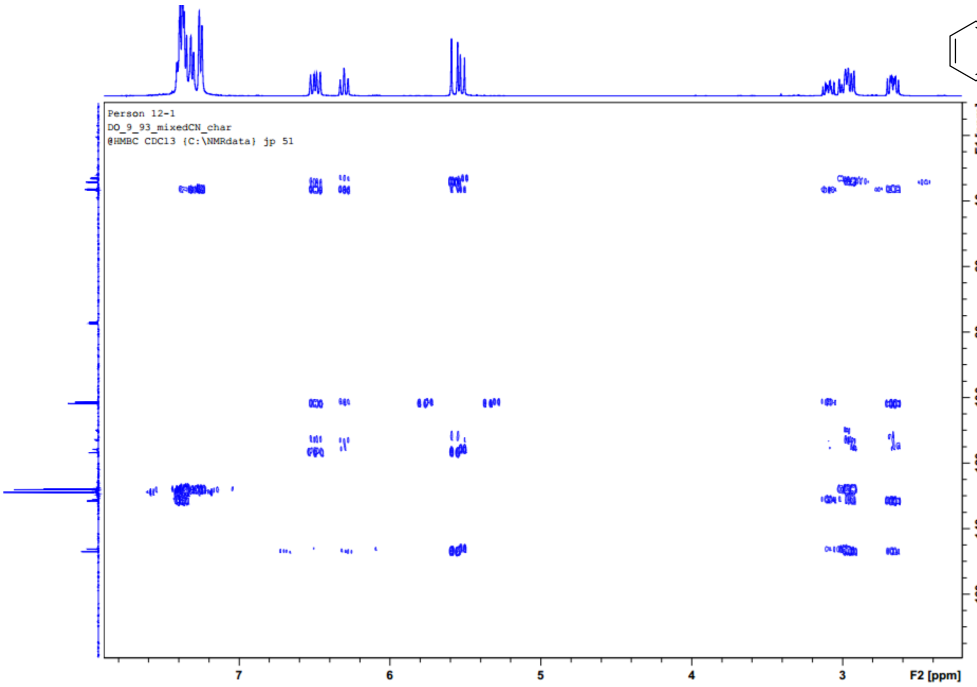


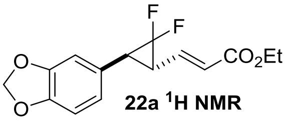

**22a $^{1}$H NMR**

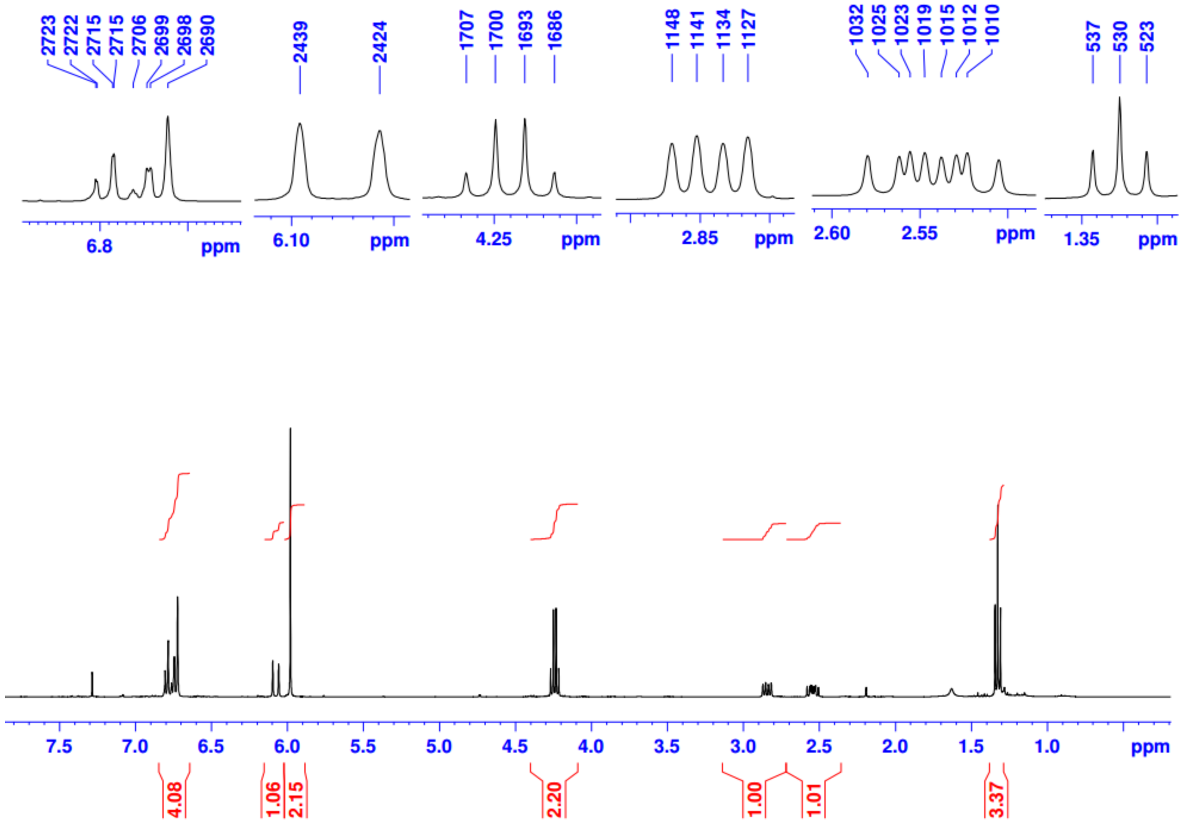

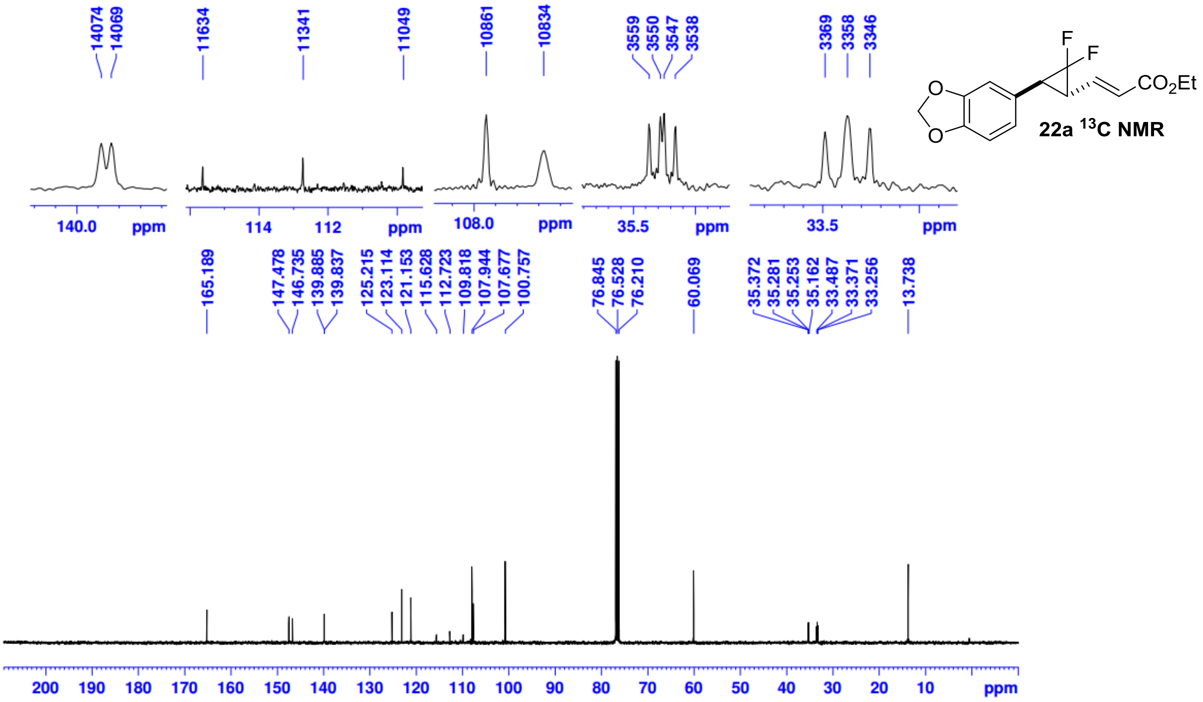
22a 13C NMR
F
F
CO2Et
O
O
14074 14069 11634 11341 11049 10861 10834 3559 3550 3547 3538 3369 3358 3346
165.189 147.478 146.735 139.885 139.837 125.215 123.114 121.153 115.628 112.723 109.818 107.944 107.677 100.757 76.845 76.528 76.210 60.069 35.372 35.281 35.253 35.162 33.487 33.371 33.256 13.738
ppm

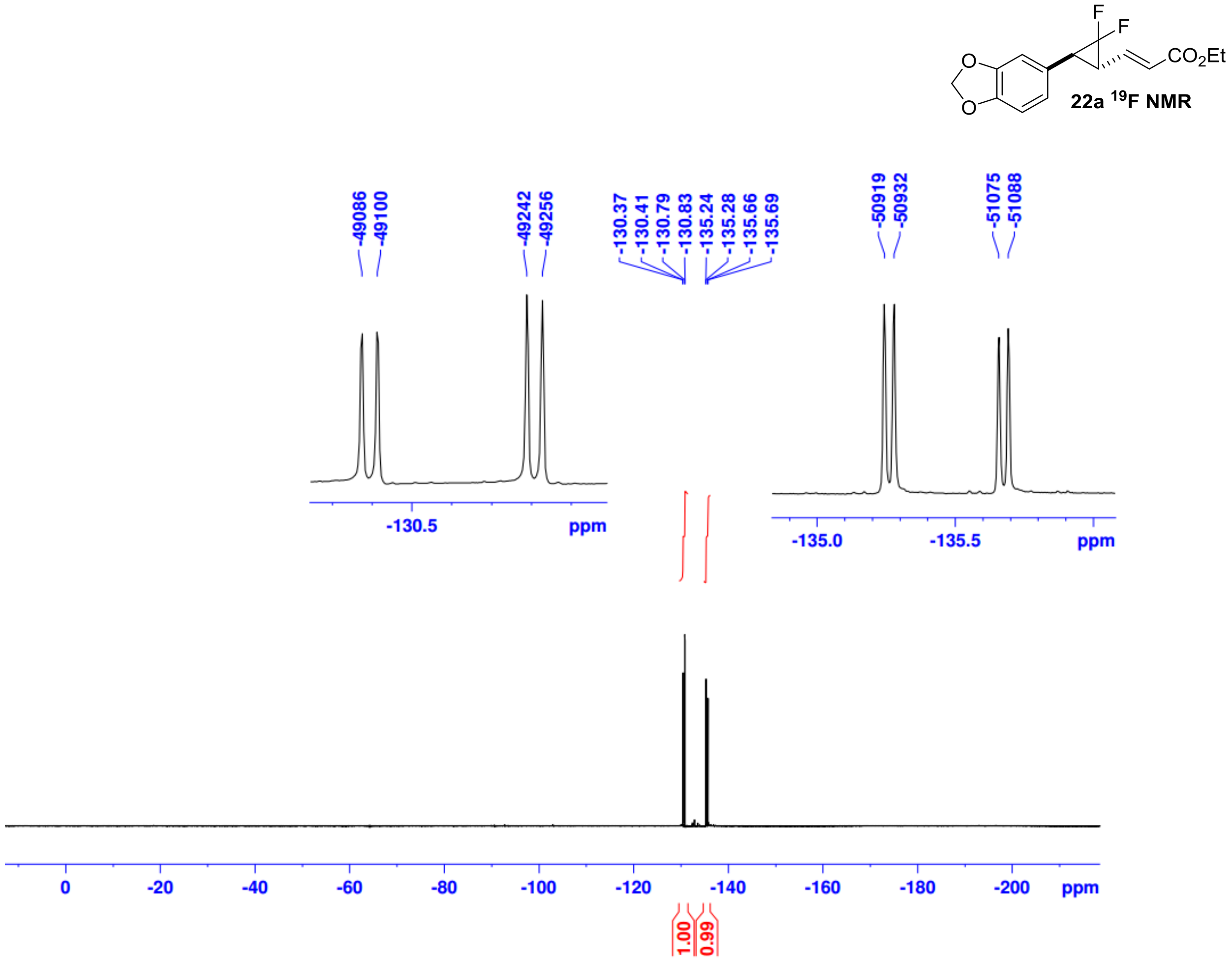
22a 19F NMR
F
F
CO2Et
O
O
-49086 -49100 -49242 -49256 -50919 -50932 -51075 -51088
-130.37 -130.41 -130.79 -130.83 -135.24 -135.28 -135.66 -135.69
1.00 0.99
ppm

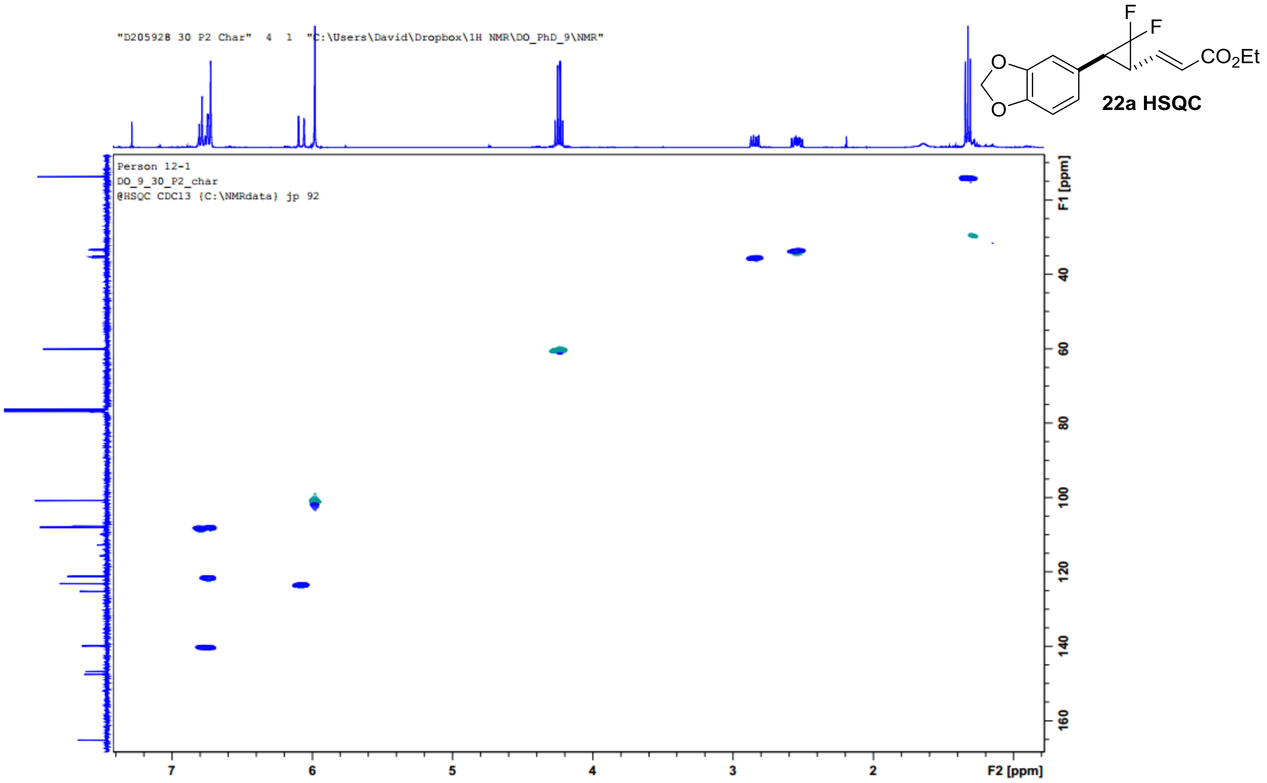


22a HSQC

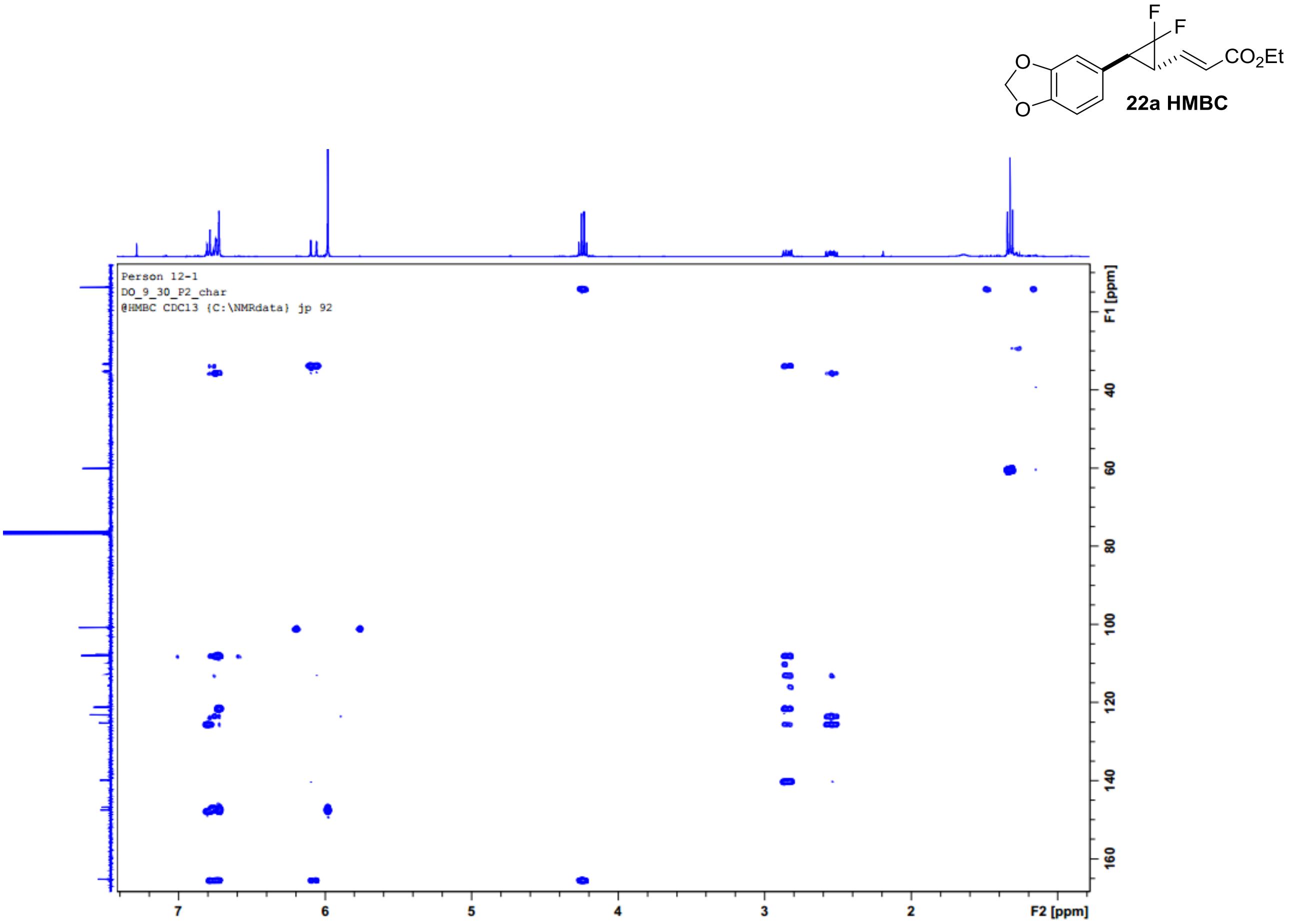


22a HMBC

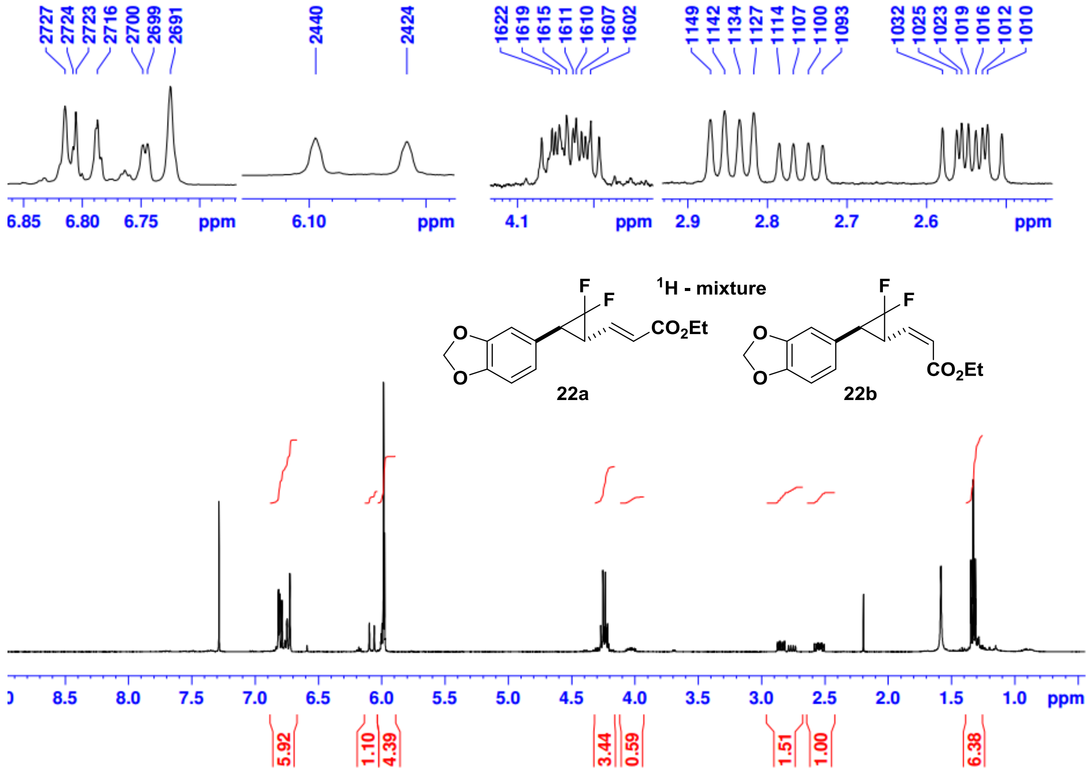
$^{1}H$ - mixture
22a
22b


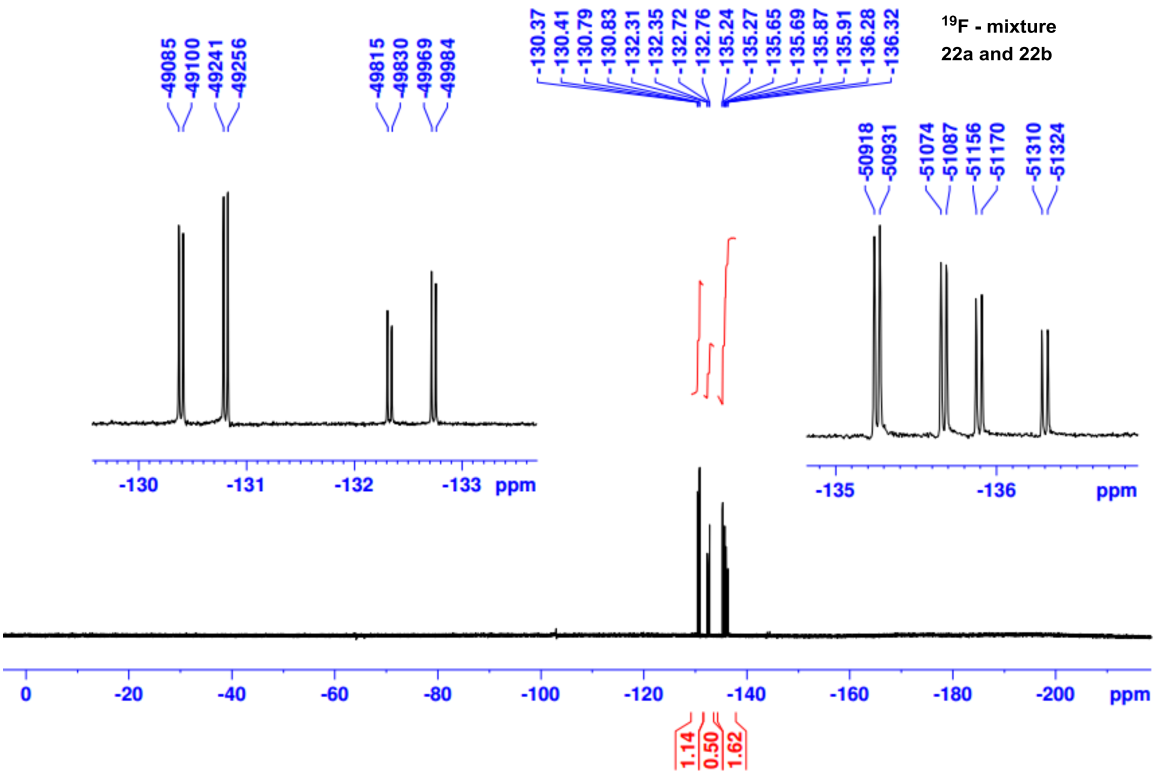
$^{19}F$ - mixture
22a and 22b

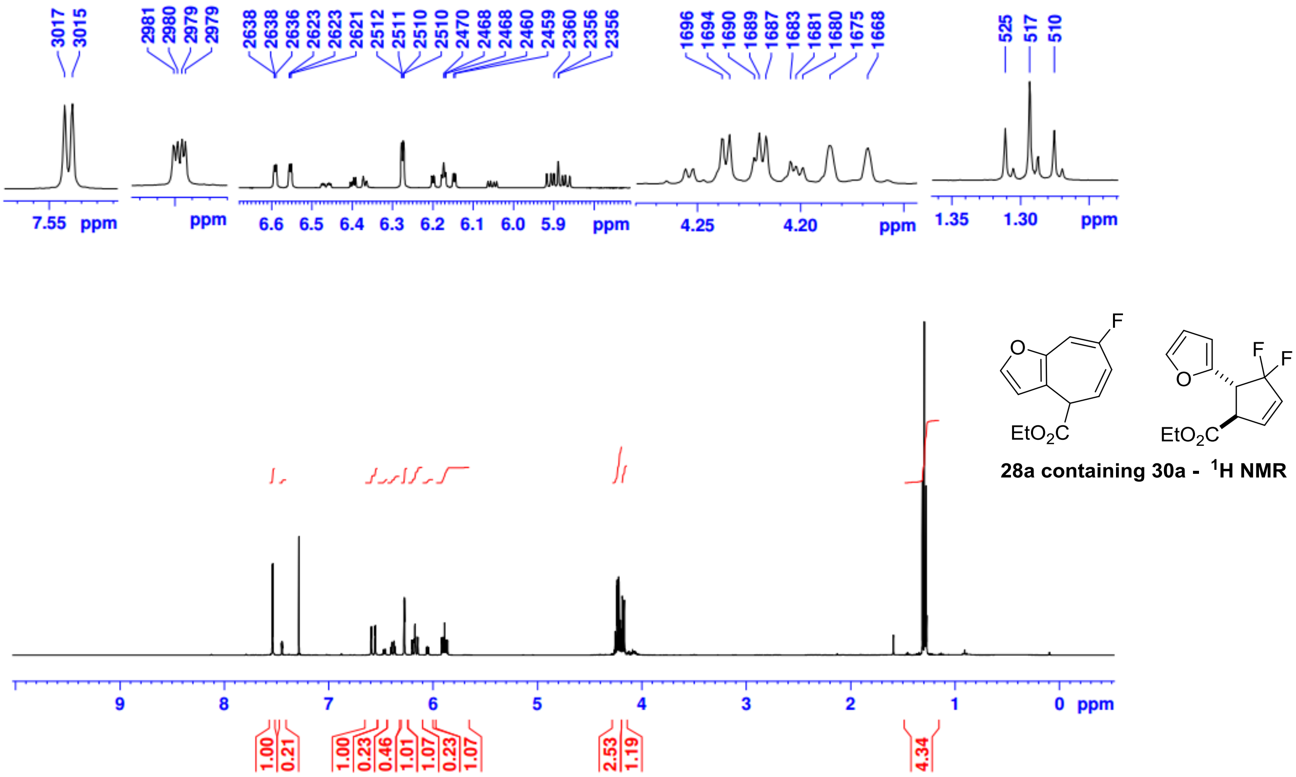

28a containing 30a - $^{1}$H NMR

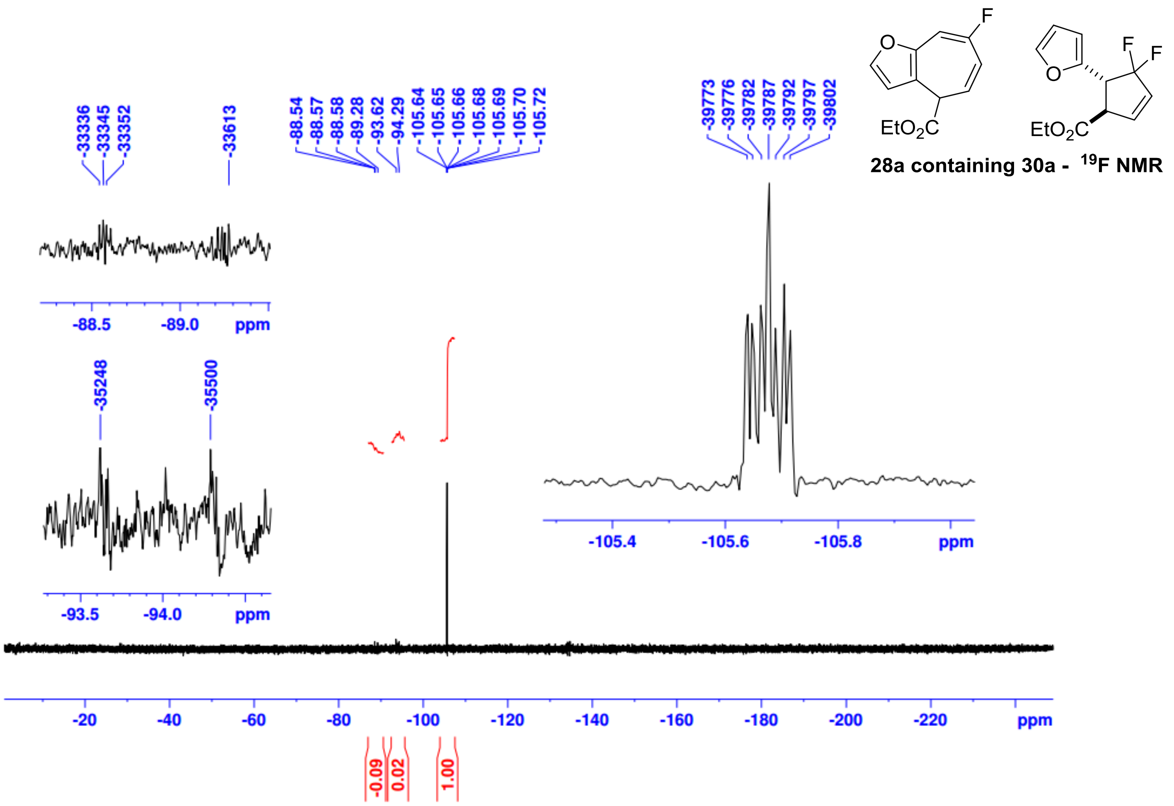

28a containing 30a - $^{19}$F NMR

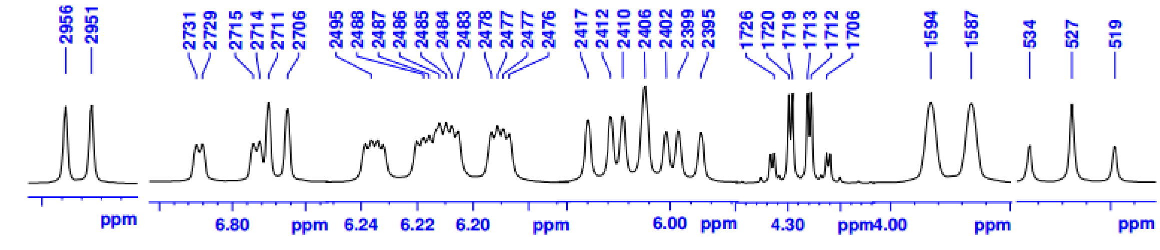


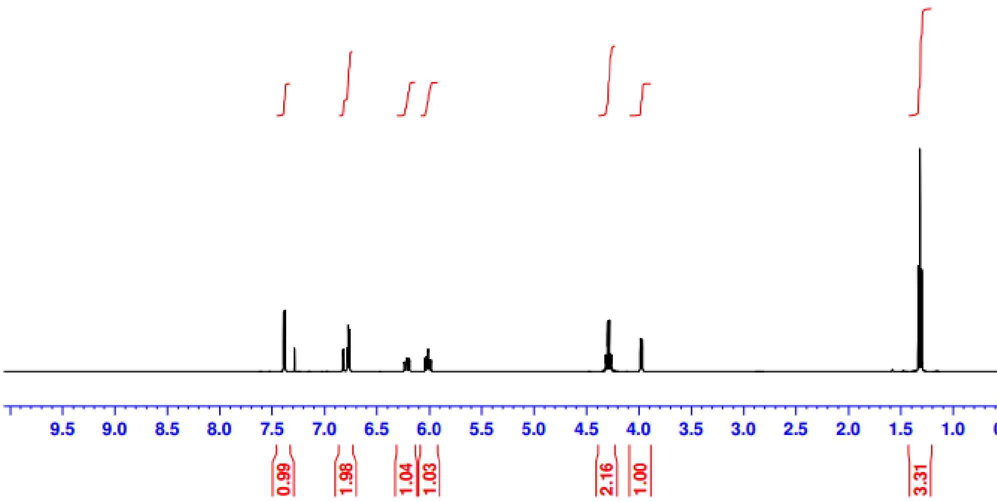


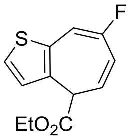


**29a $^{1}$H NMR**

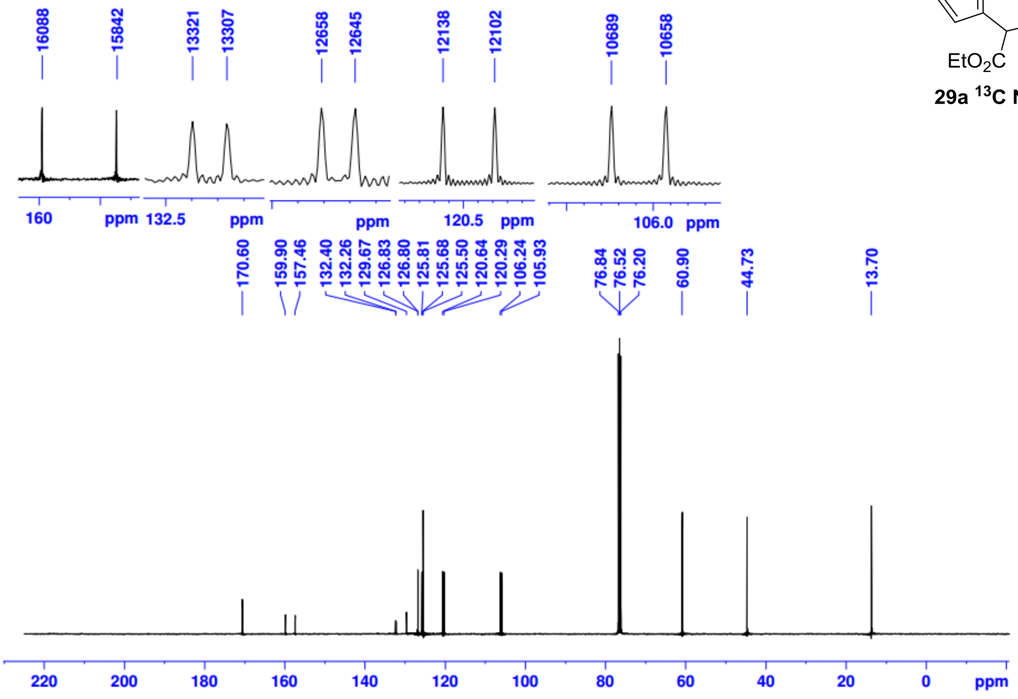


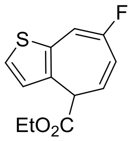


**29a $^{13}$C NMR**

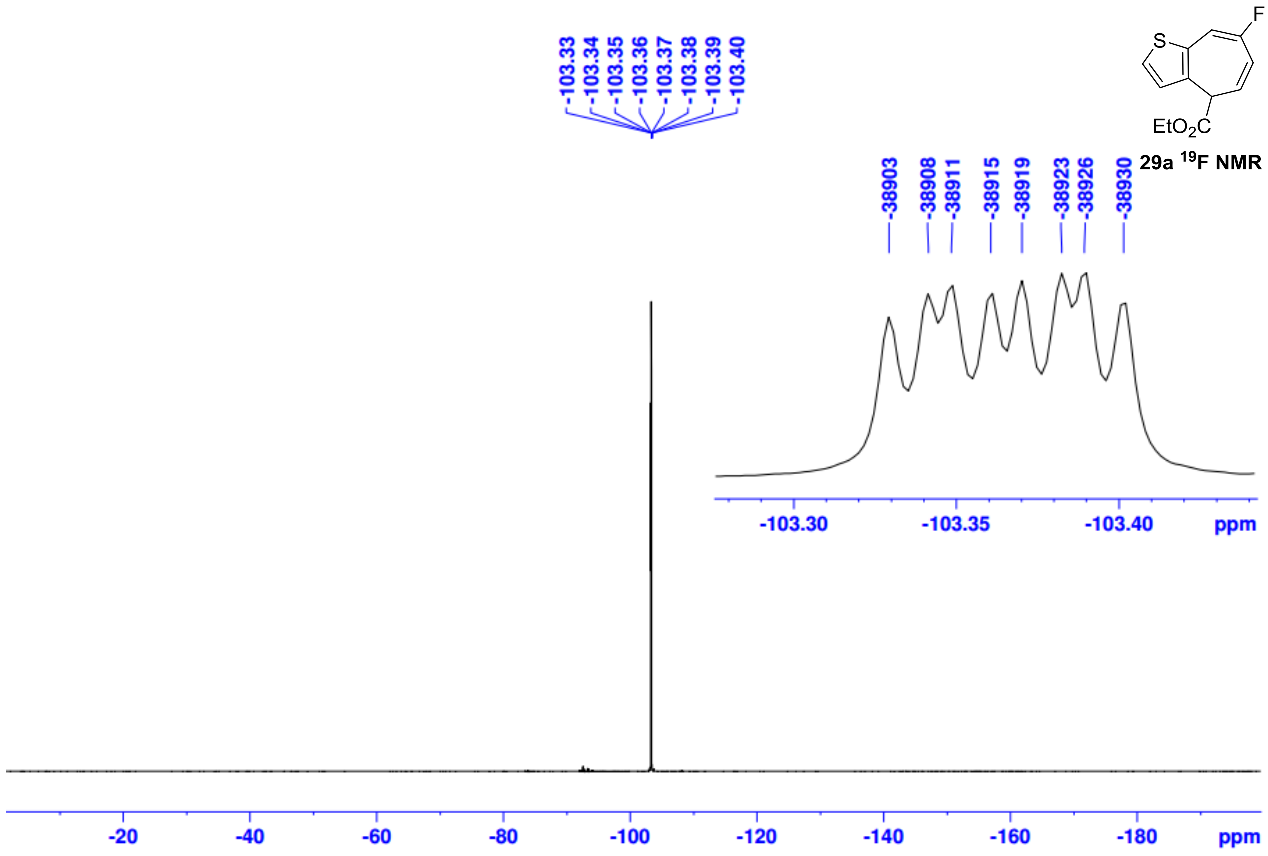


**29a $^{19}$F NMR**

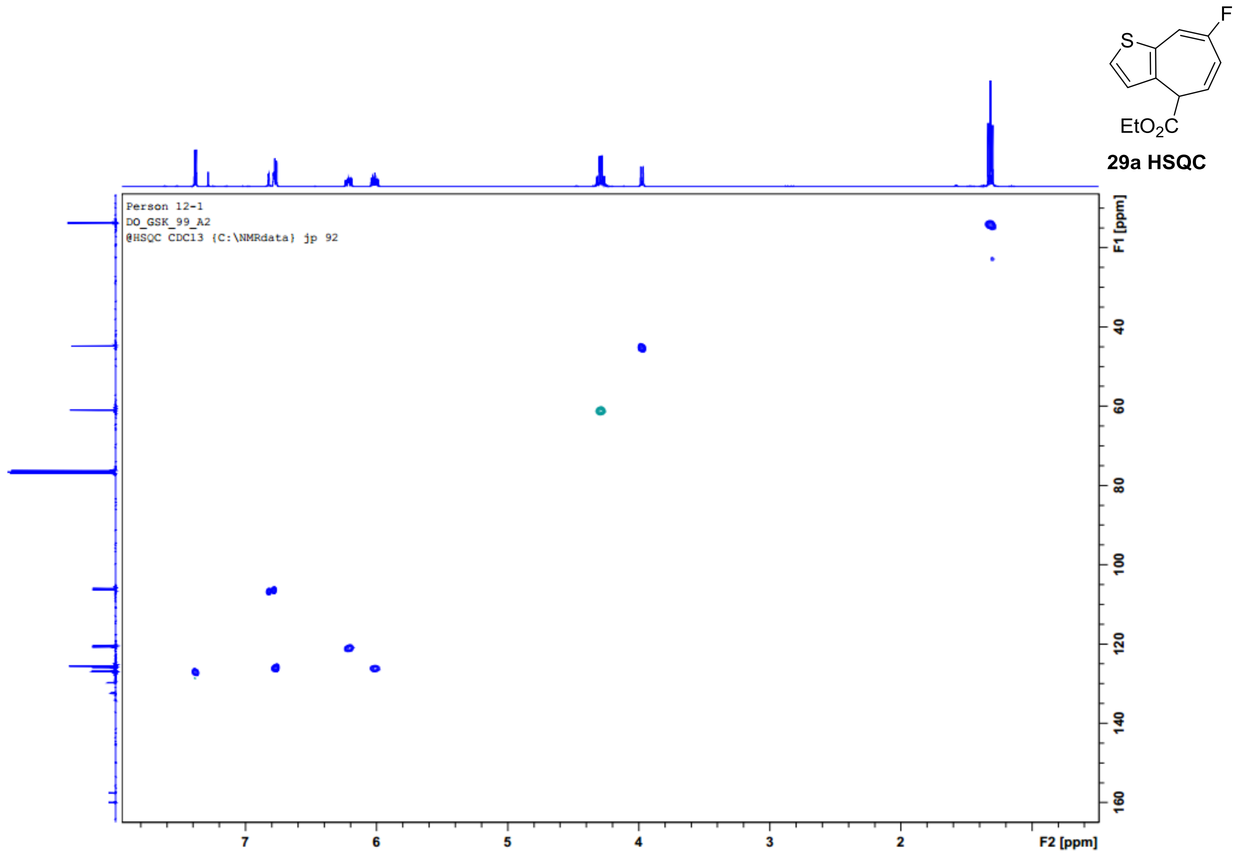


**29a HSQC**

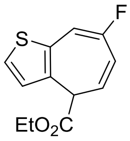


**29a HMBC**

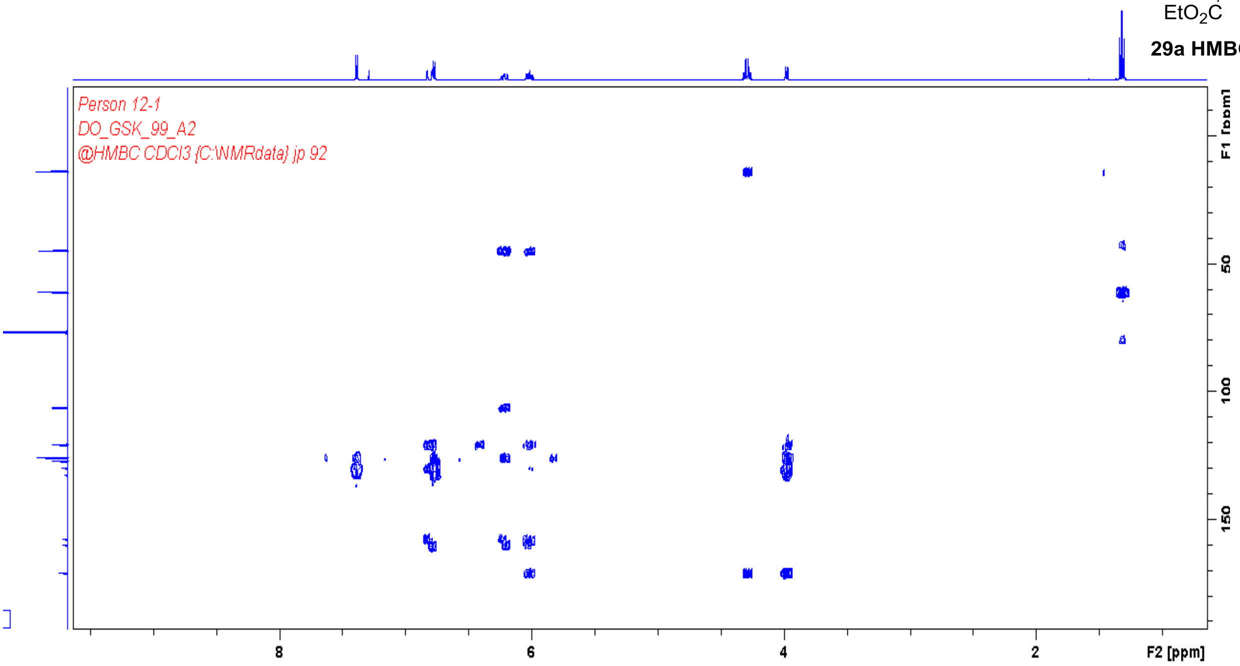


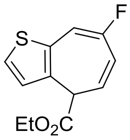


**29a HMBC (expanded)**

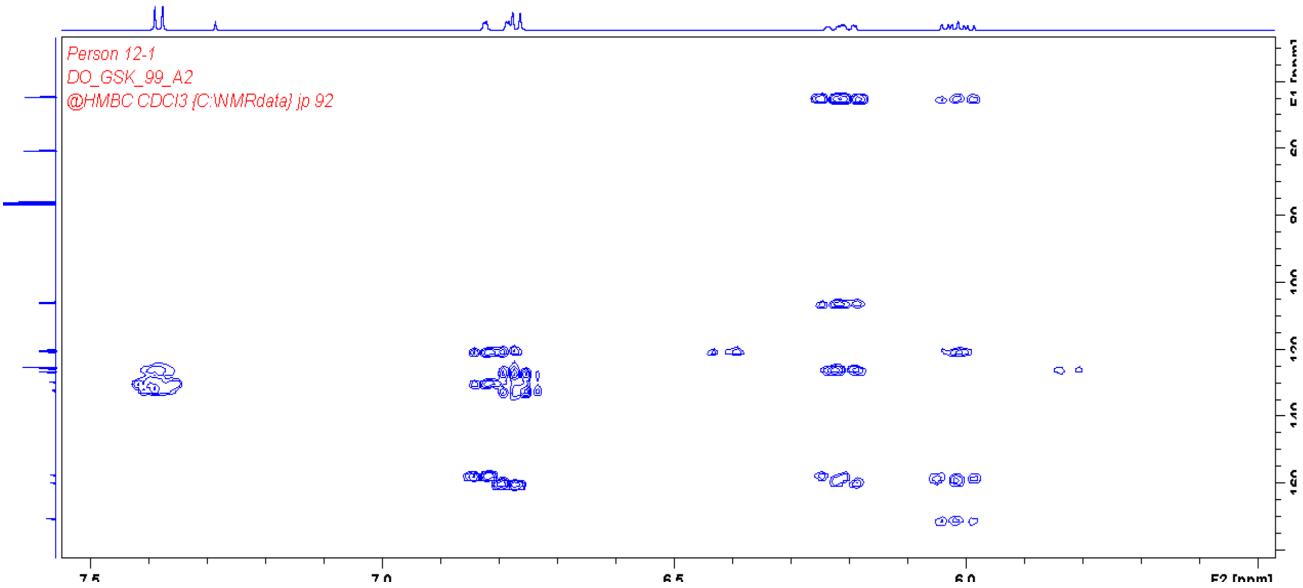

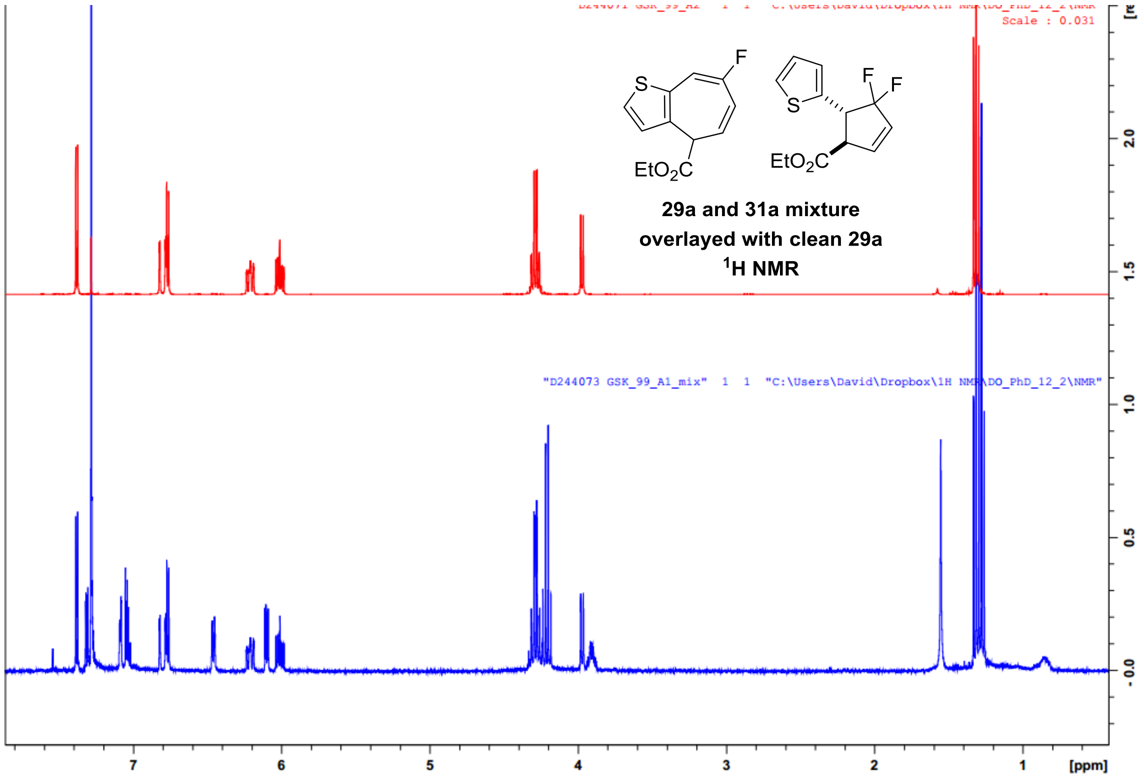
29a and 31a mixture
overlayed with clean 29a
1H NMR
"D244073 GSK_99_A1_mix" 1 1 "C:\Users\David\Dropbox\1H NMR\DO_PhD_12_2\NMR"
Scale : 0.031
[ppm]

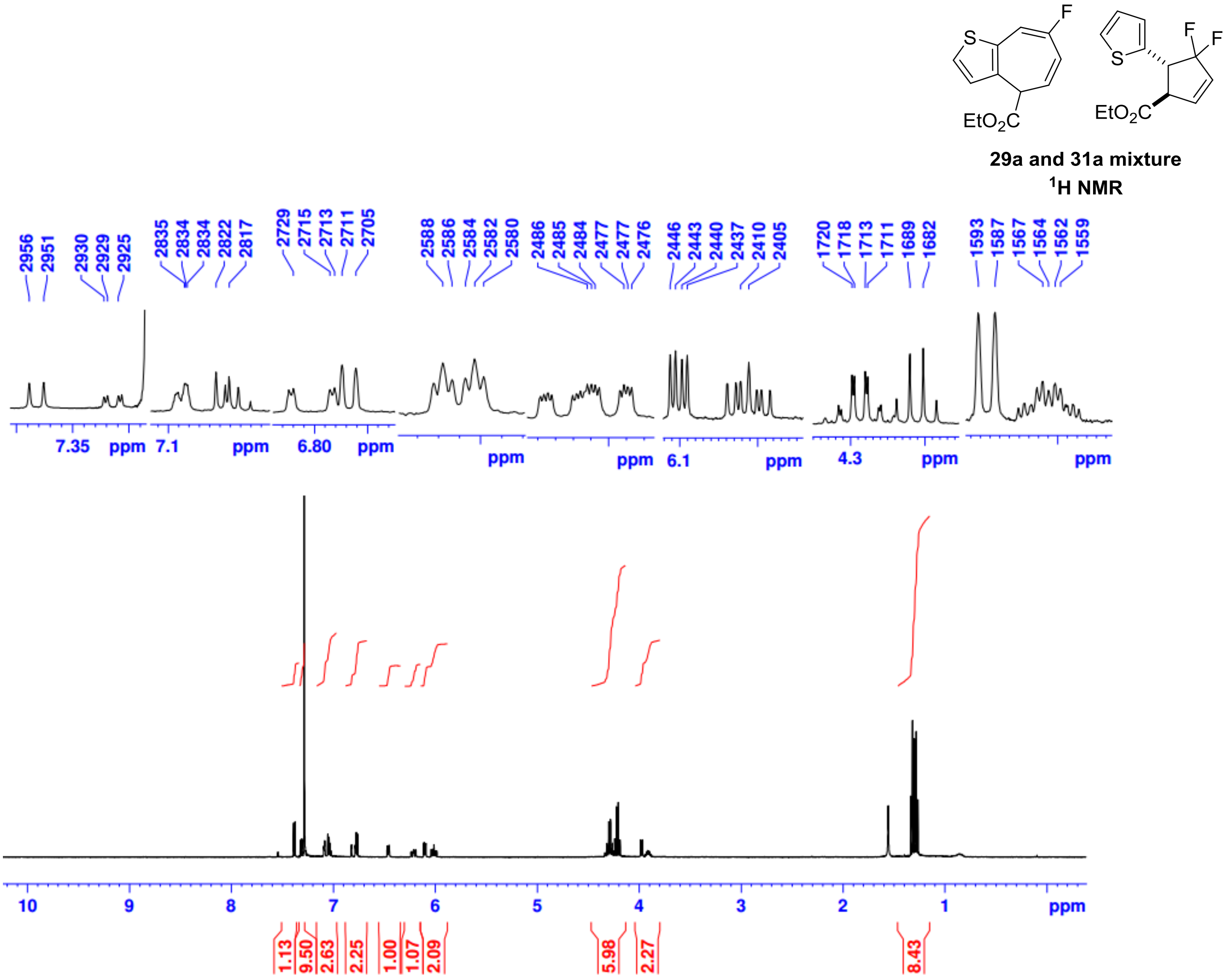
29a and 31a mixture
1H NMR
ppm
1.13
9.50
2.63
2.25
1.00
1.07
2.09
5.98
2.27
8.43

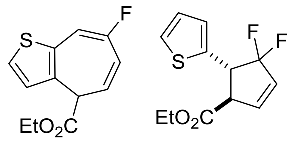


**29b and 31b mixture**
**$^{19}$F NMR**

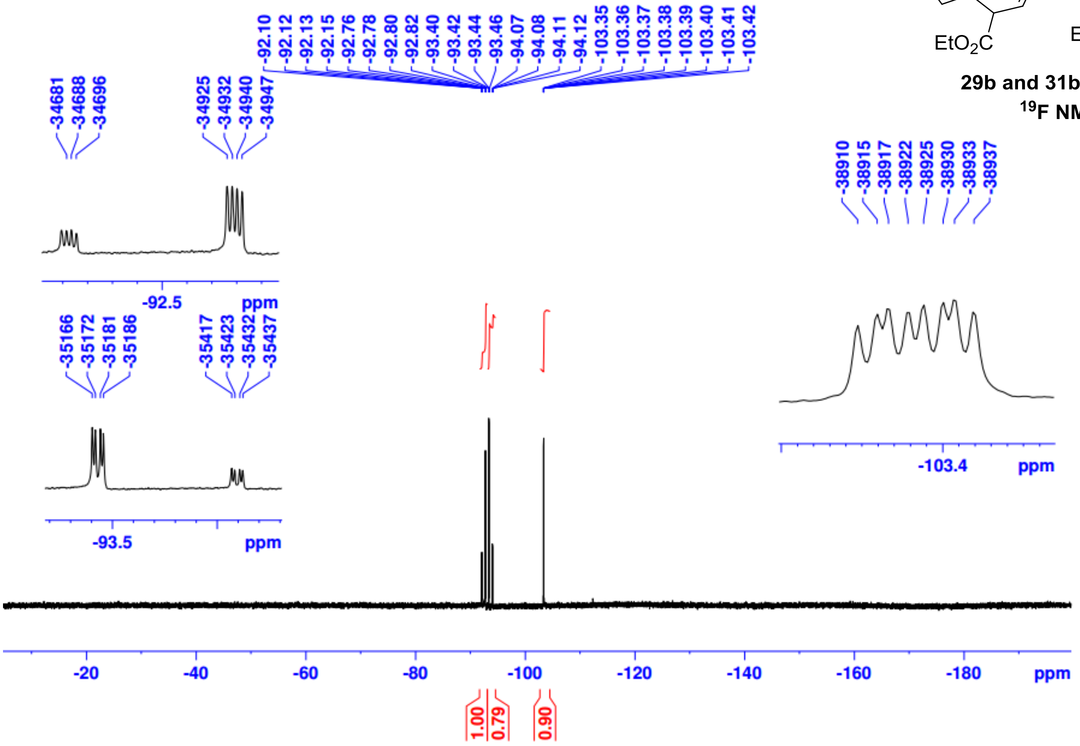


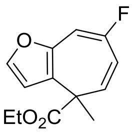


**28b $^{1}$H NMR**

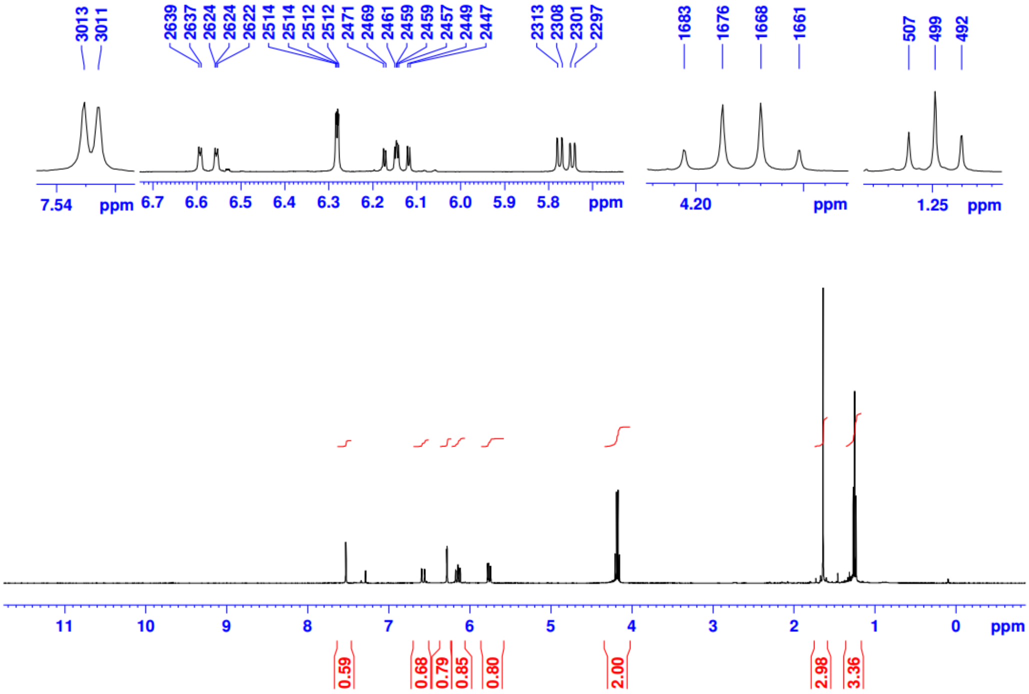

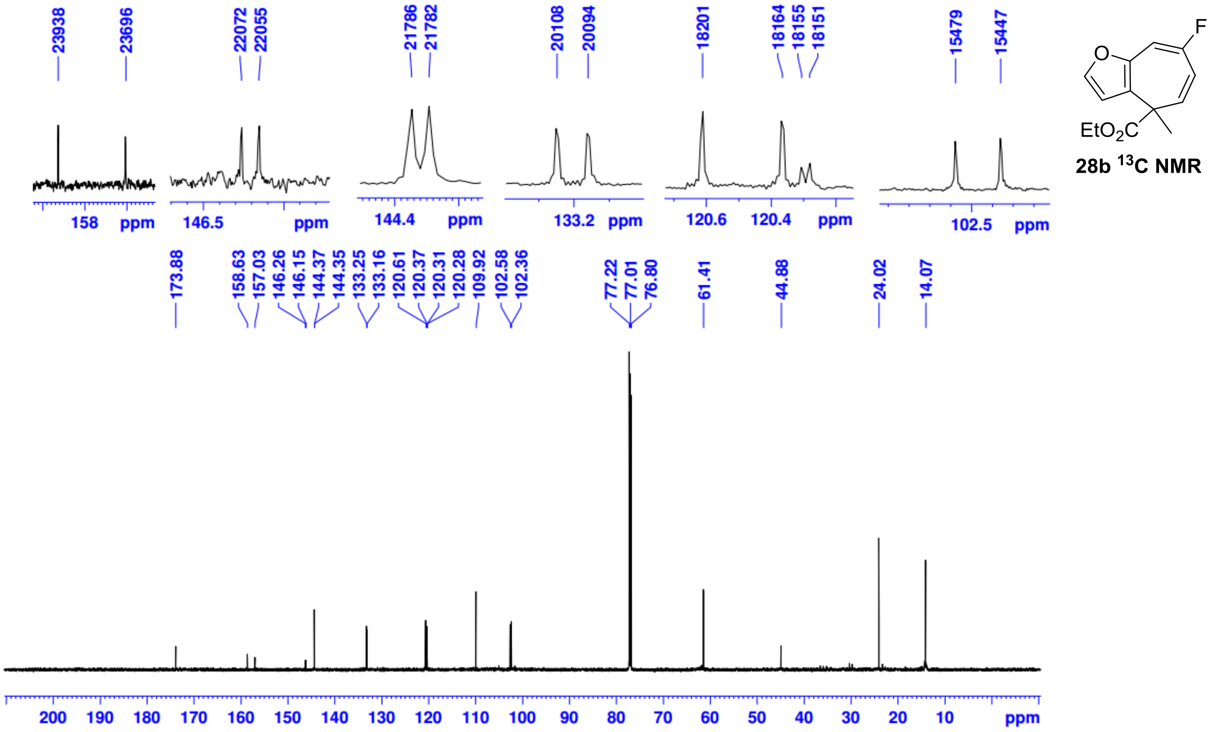


28b $^{13}C$ NMR

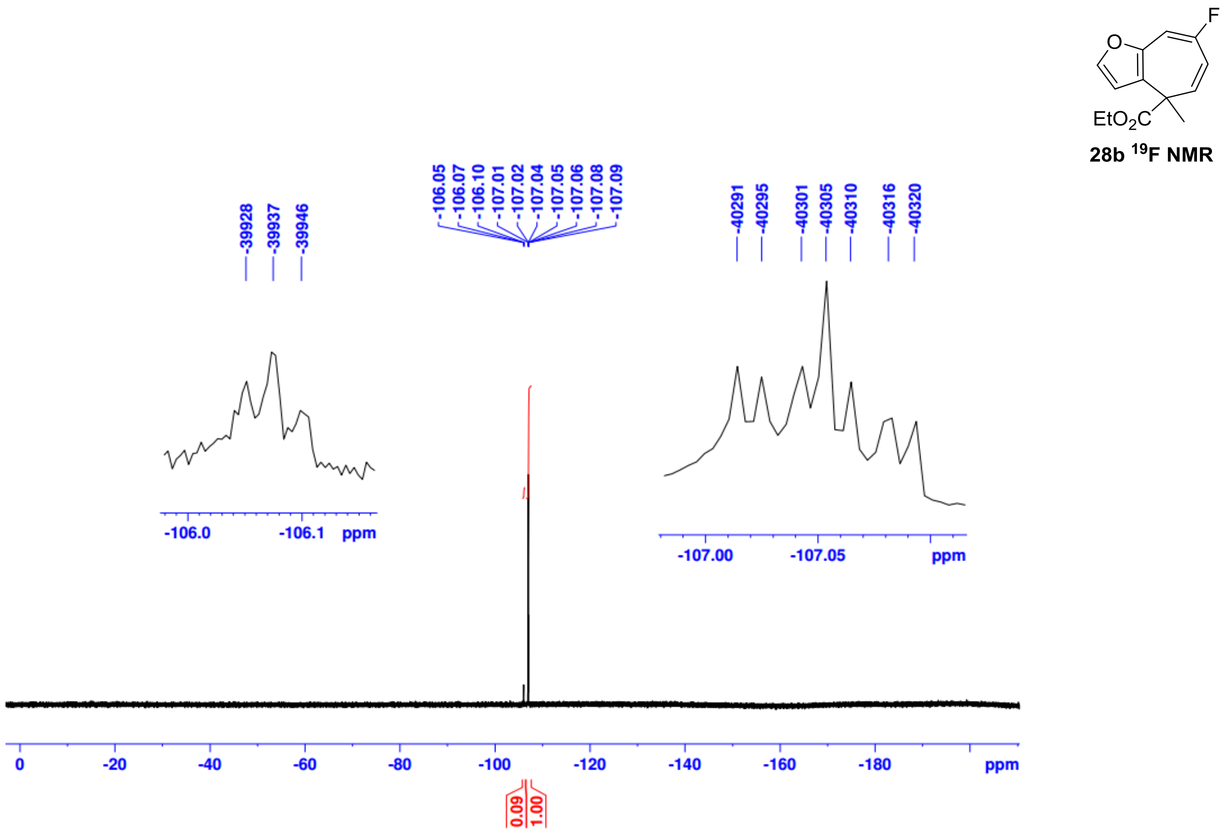


28b $^{19}F$ NMR

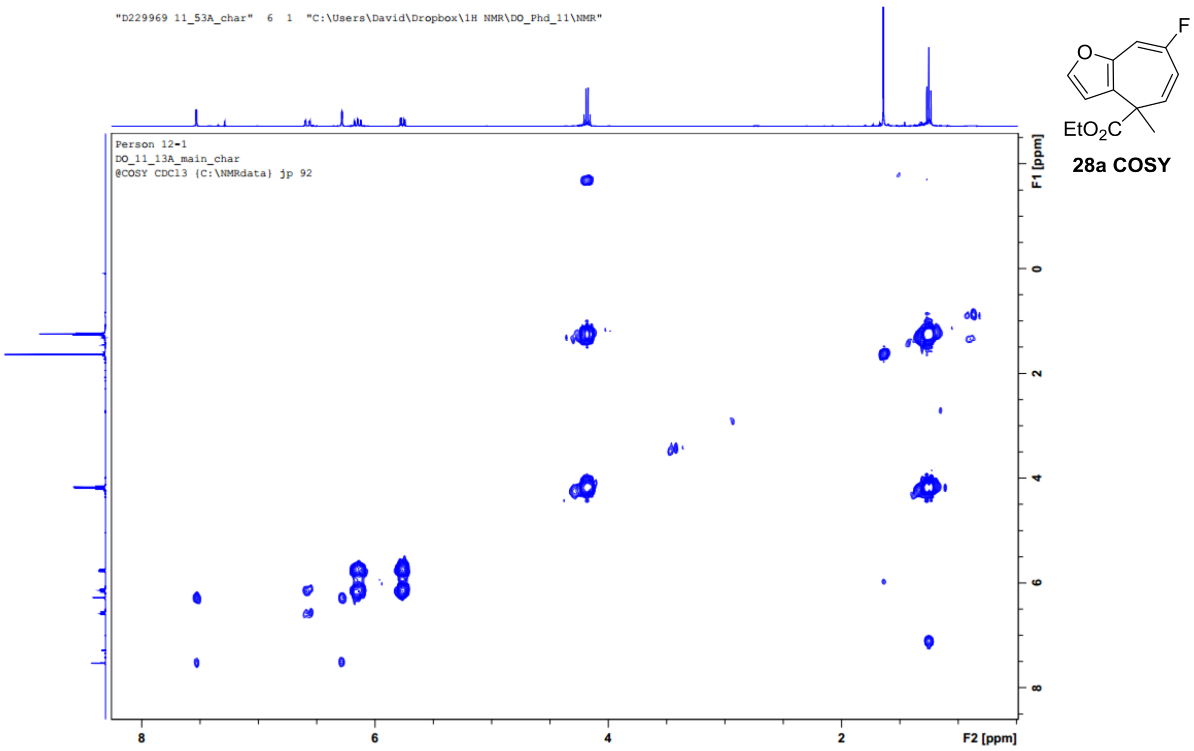


**28a COSY**

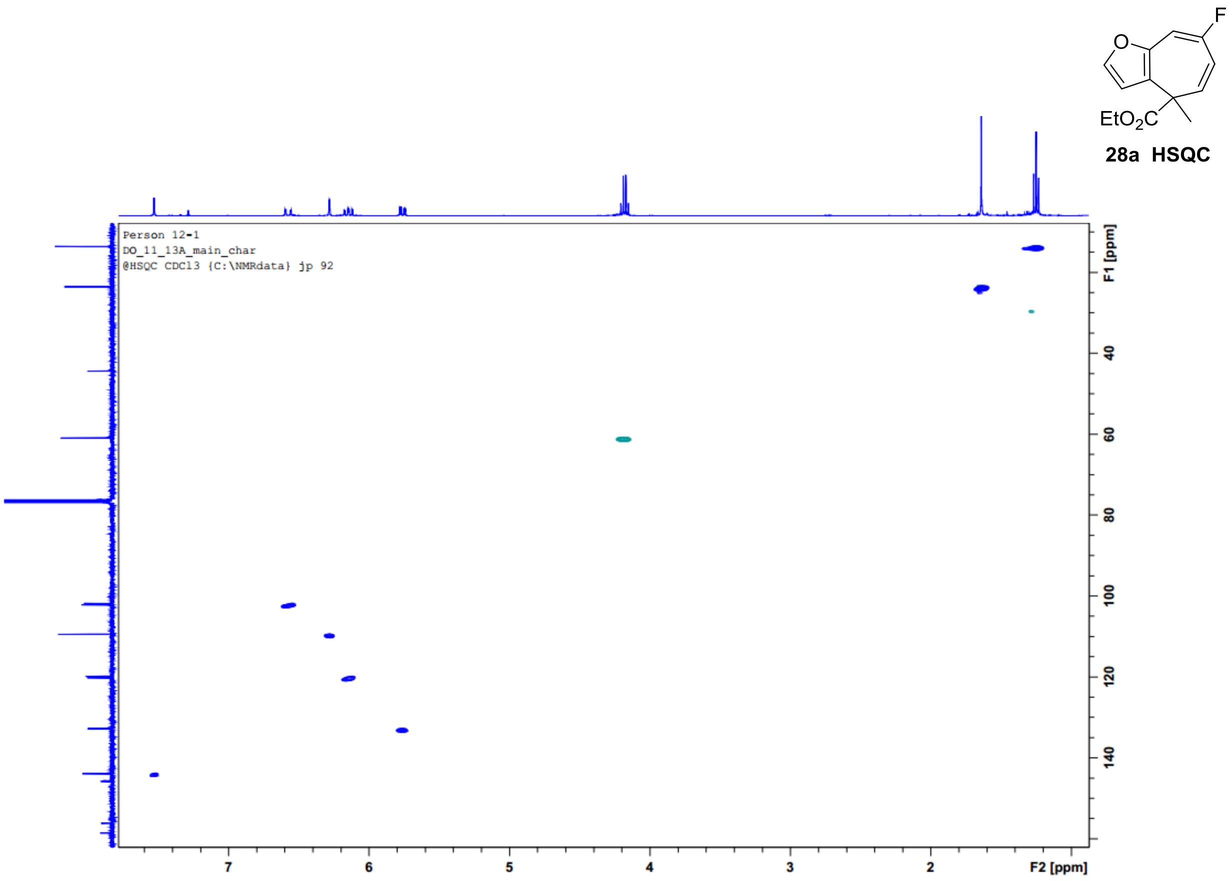


**28a HSQC**

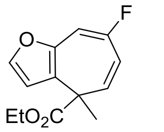


**28a HMBC**

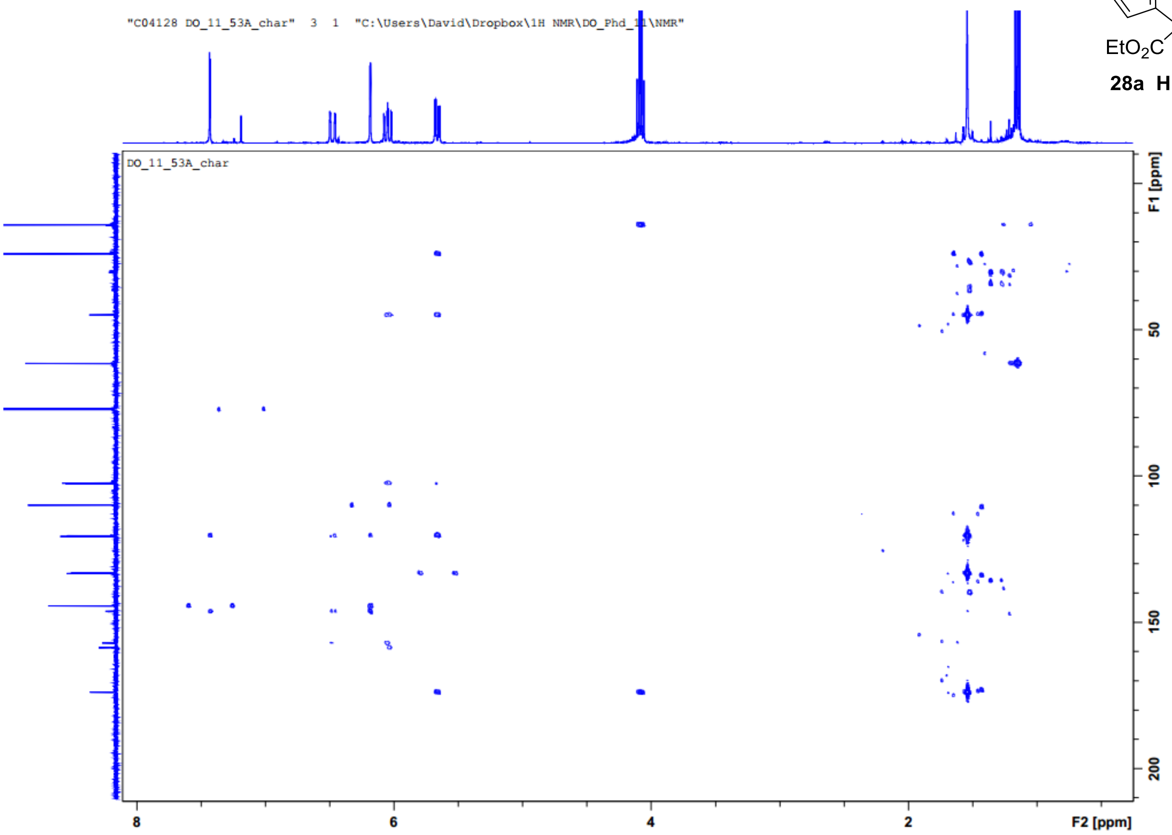


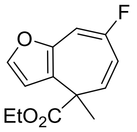


**28a HMBC (expanded)**

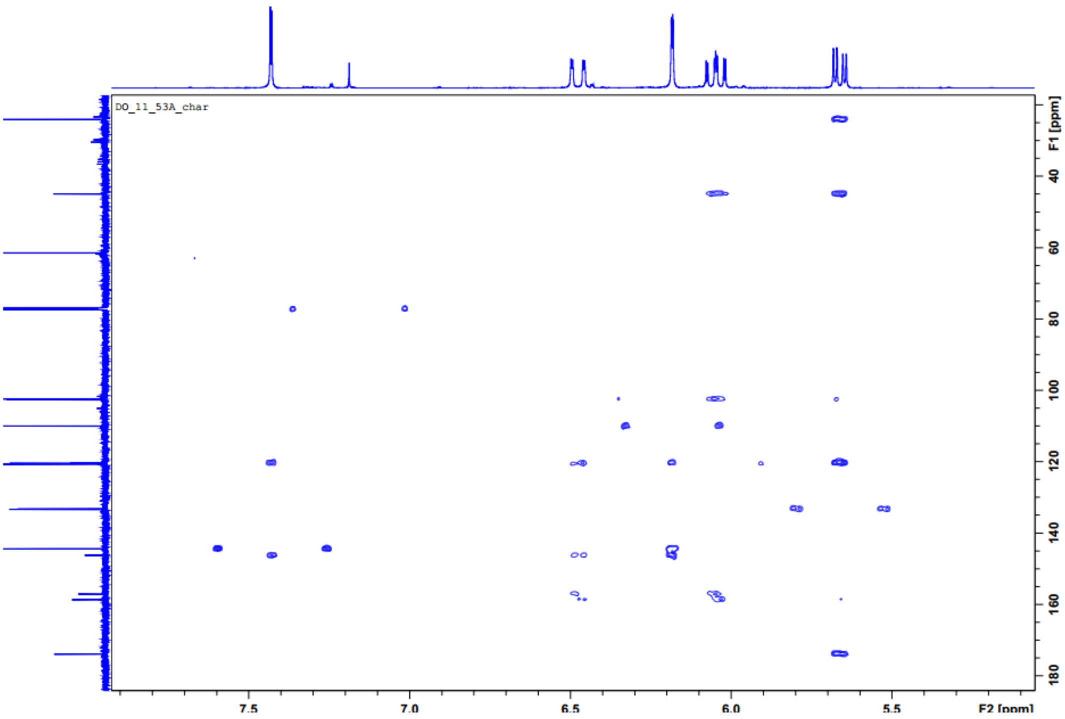

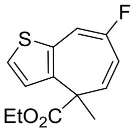


**29b $^1$H NMR**

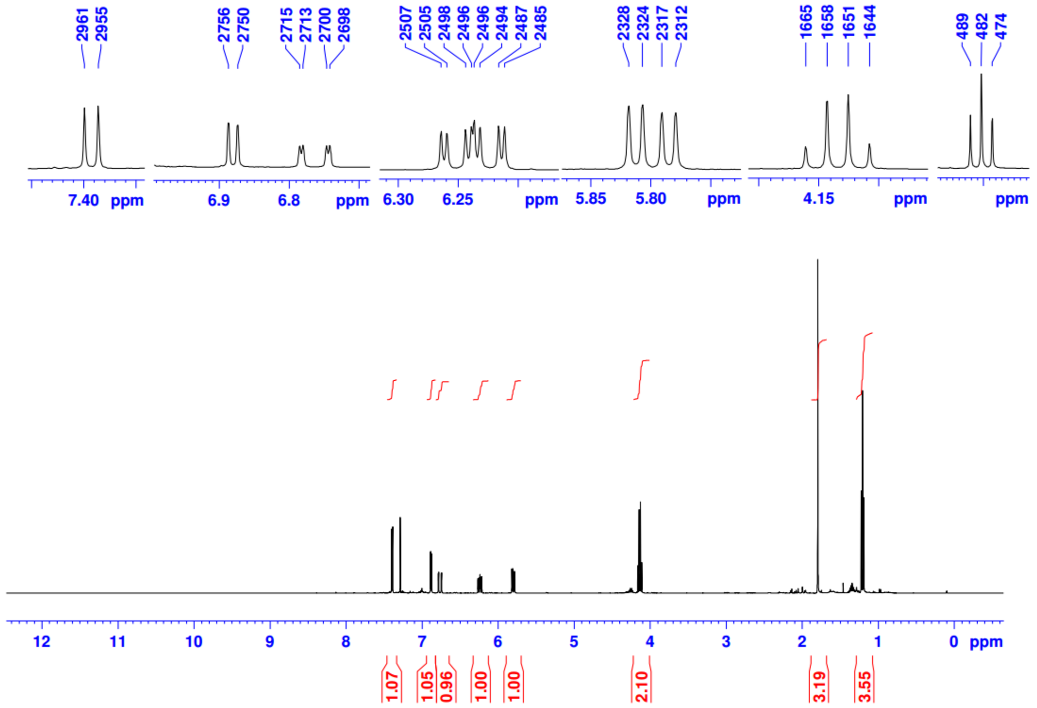


**29b $^{13}$C NMR**

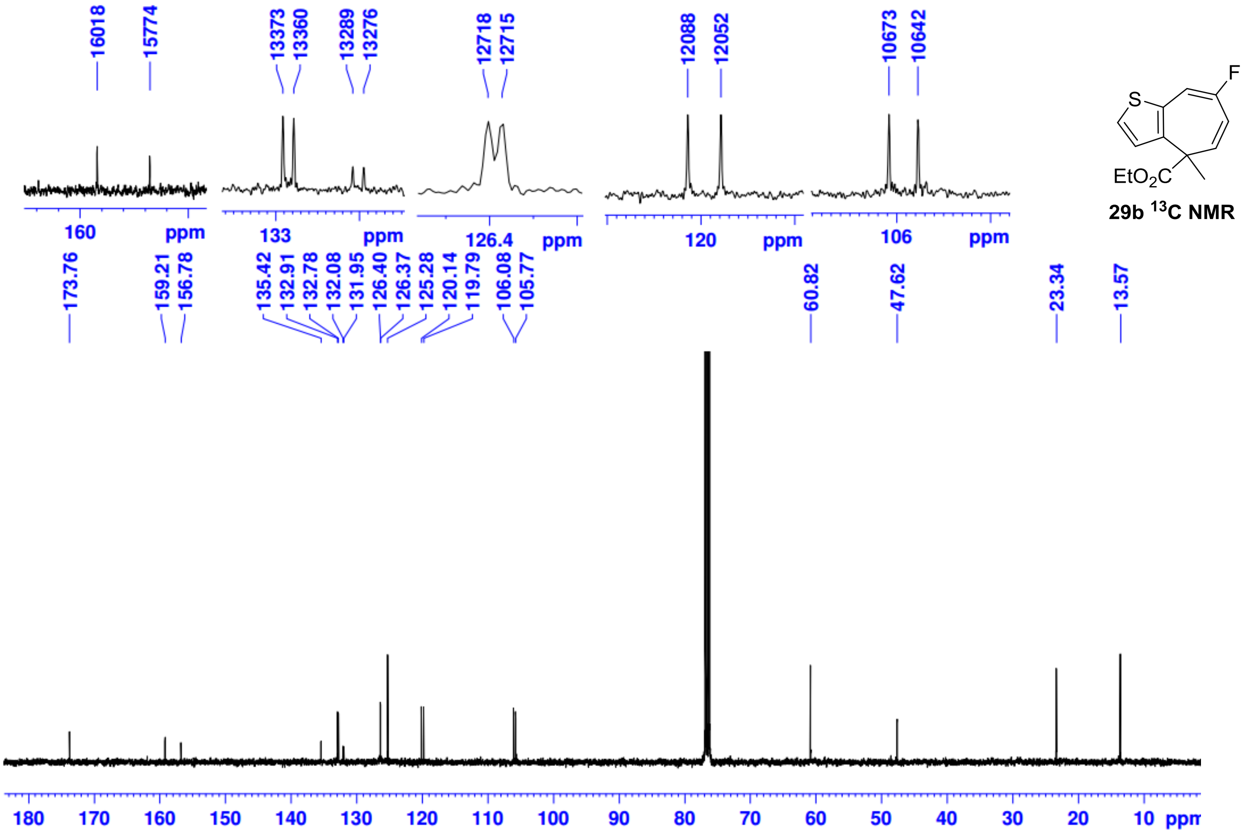

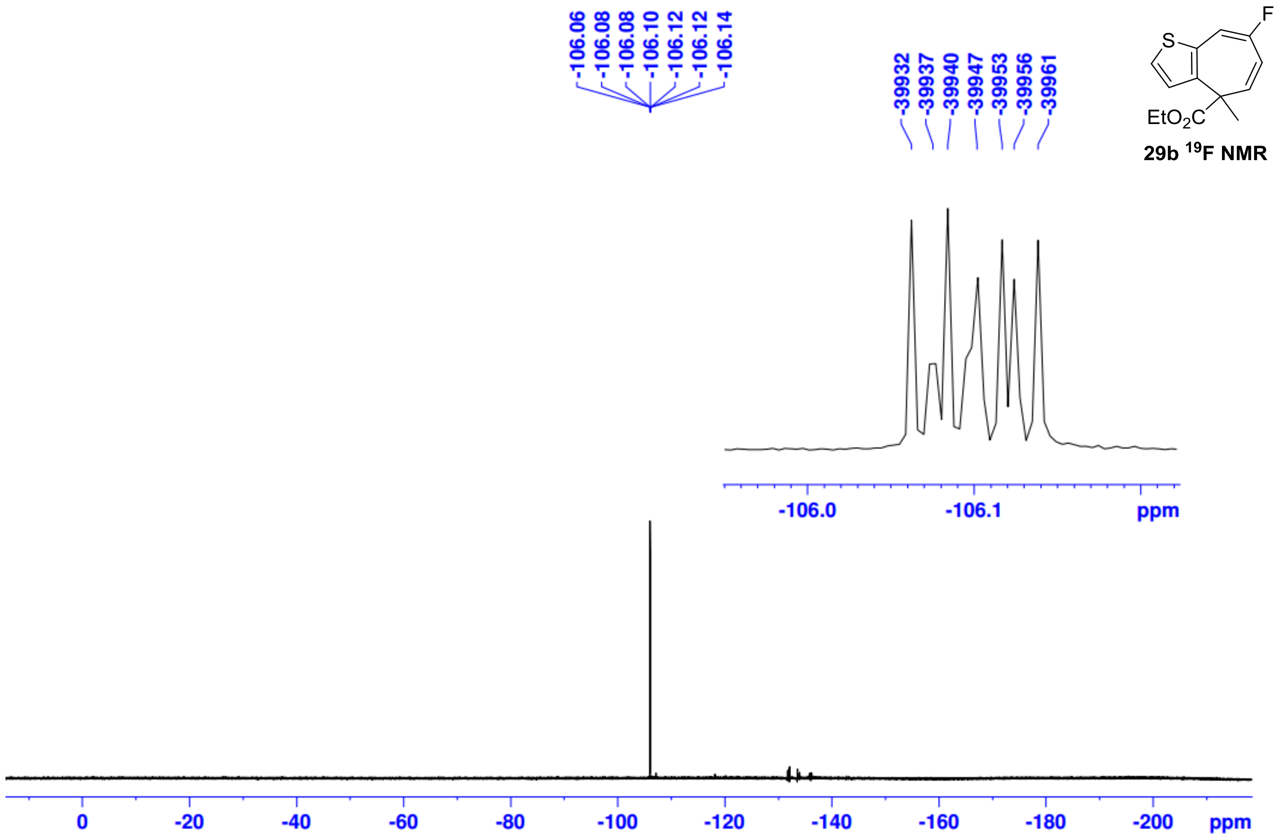


29b $^{19}$F NMR

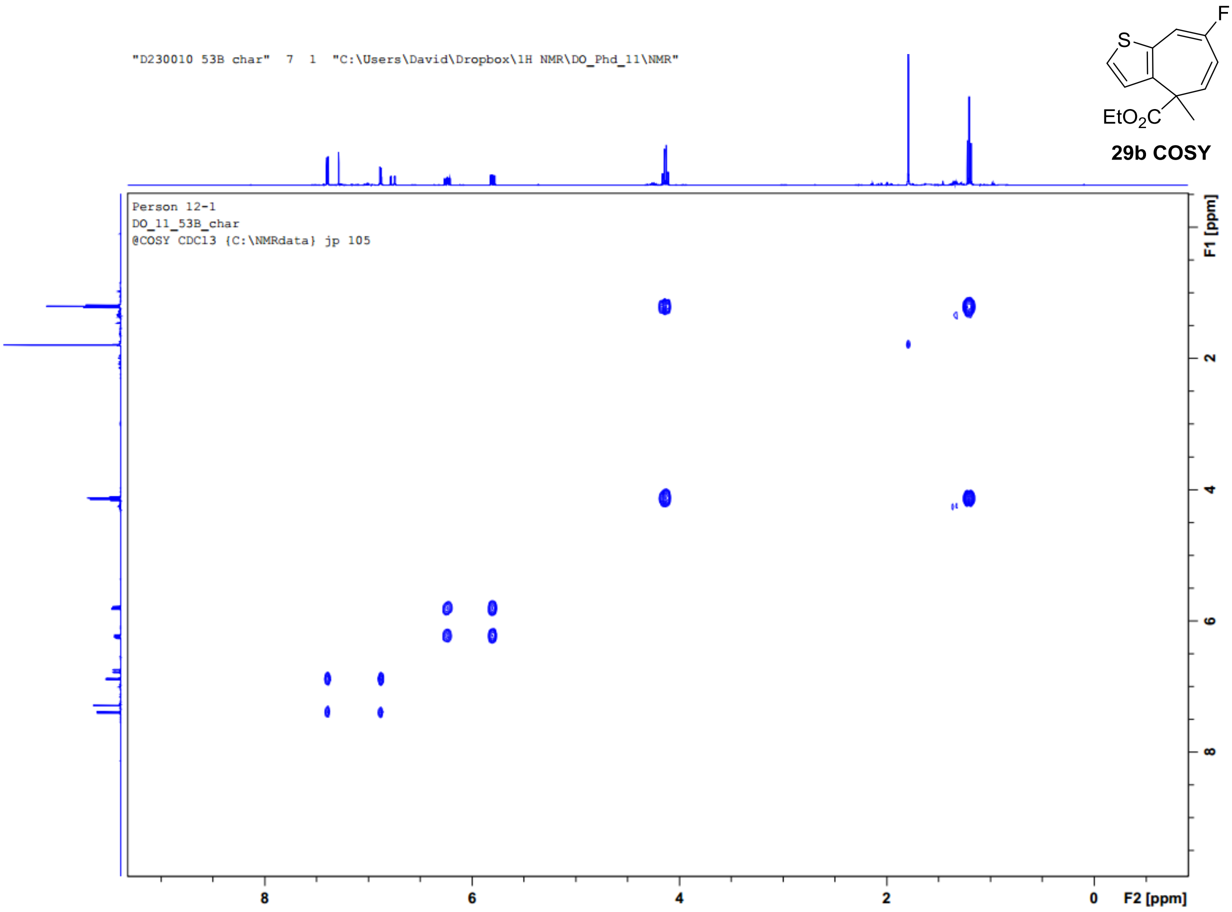


29b COSY

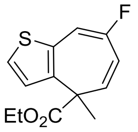


**29b HSQC**

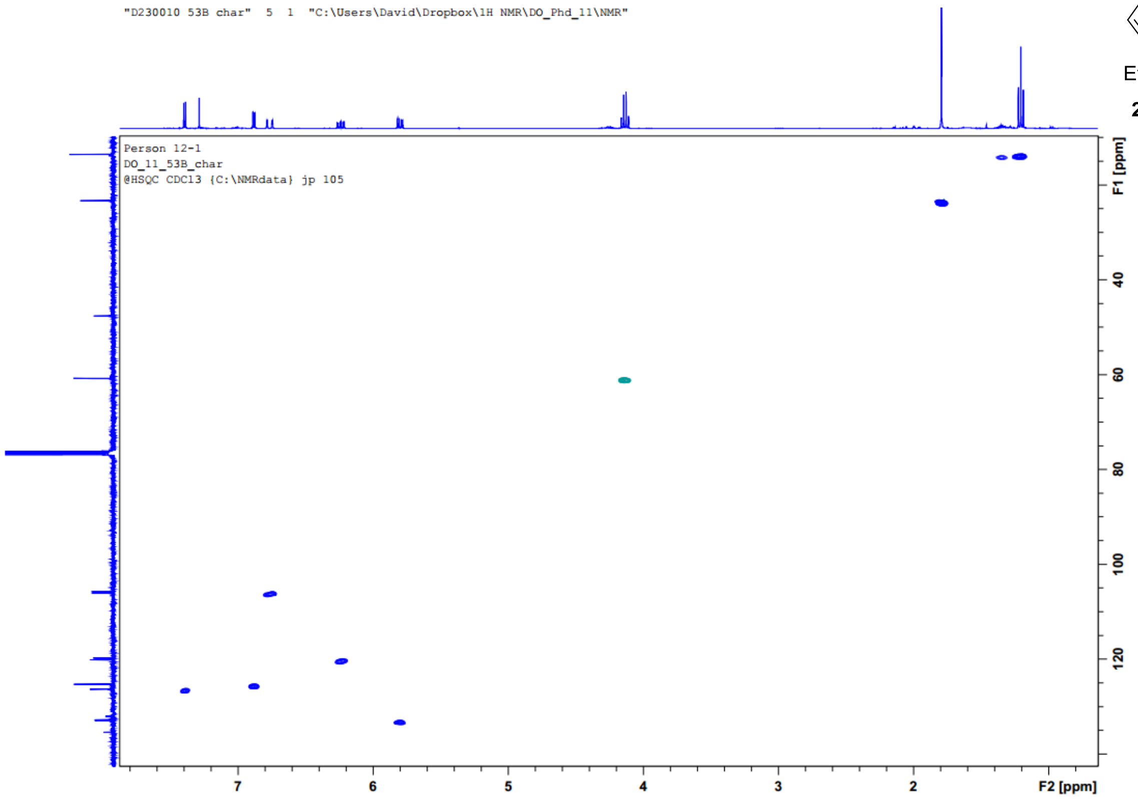


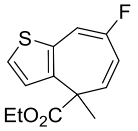


**29b HMBC**

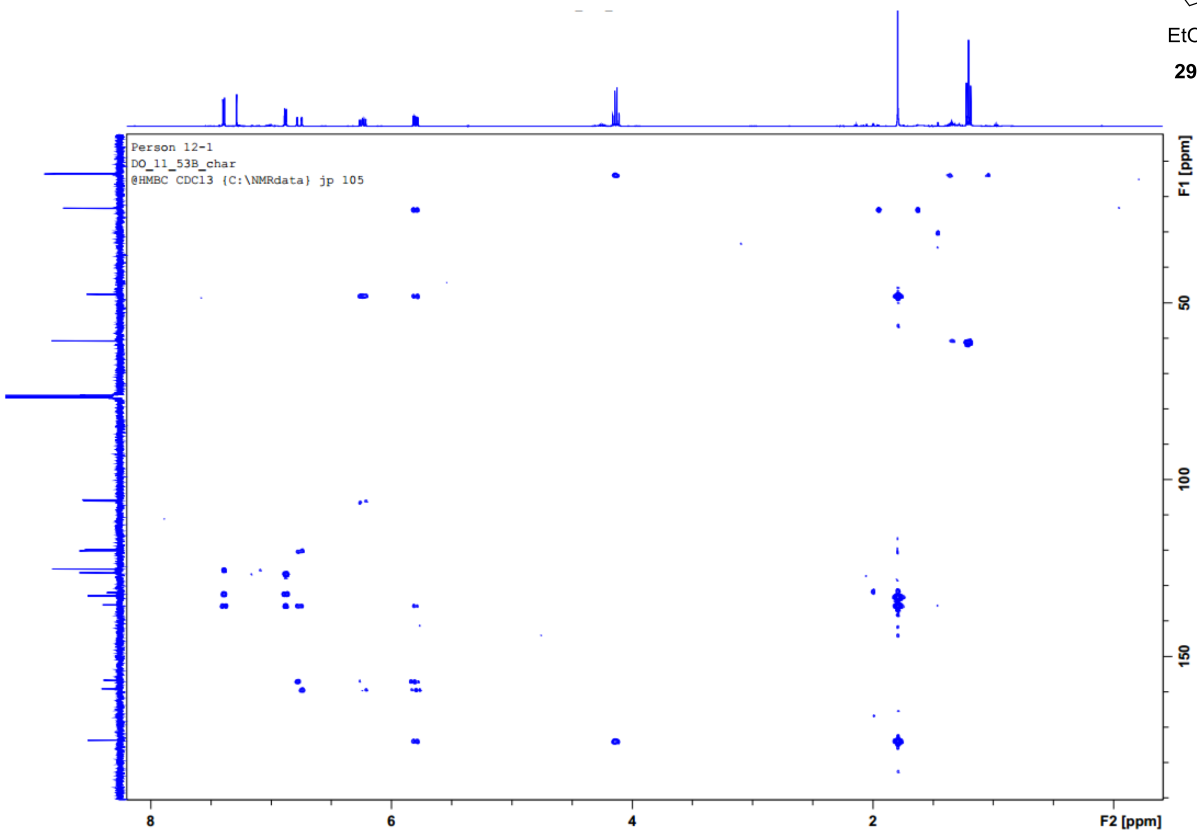

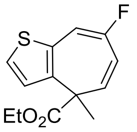


**29b HMBC (expanded)**

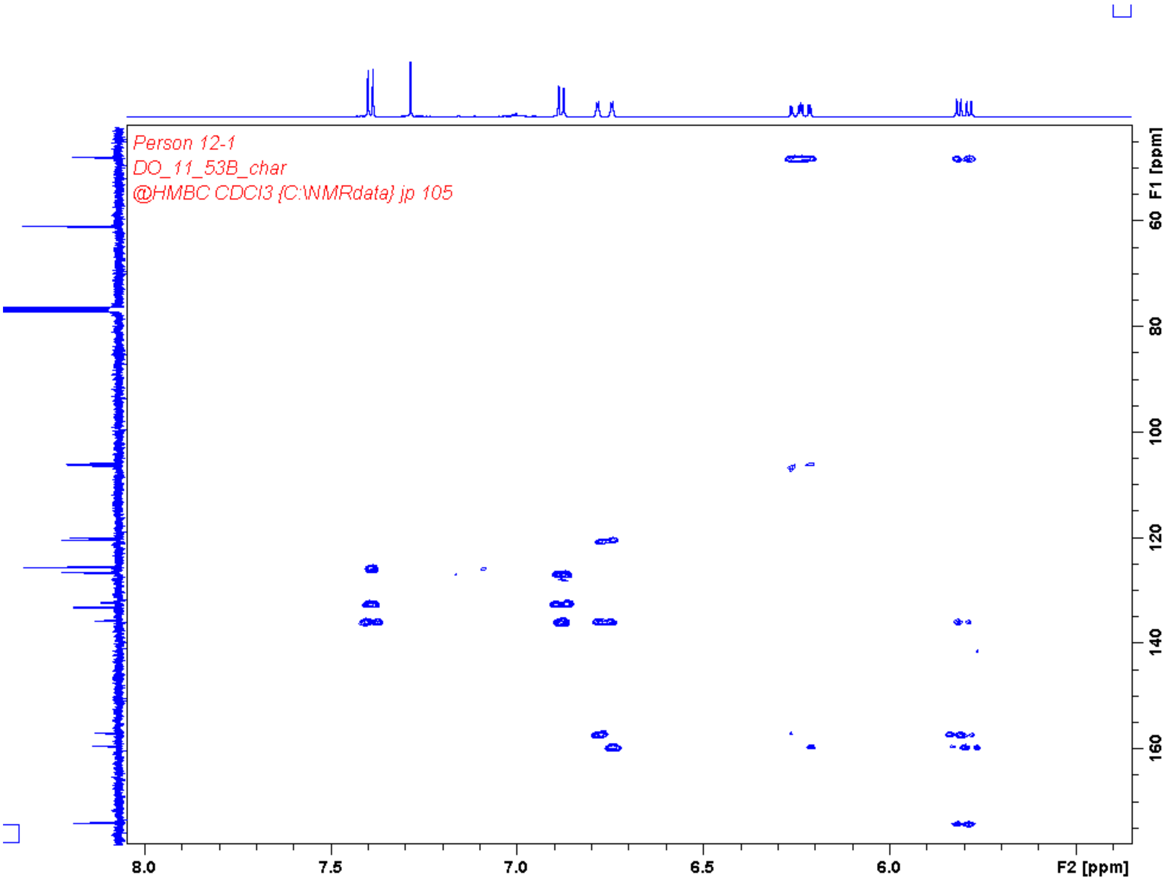


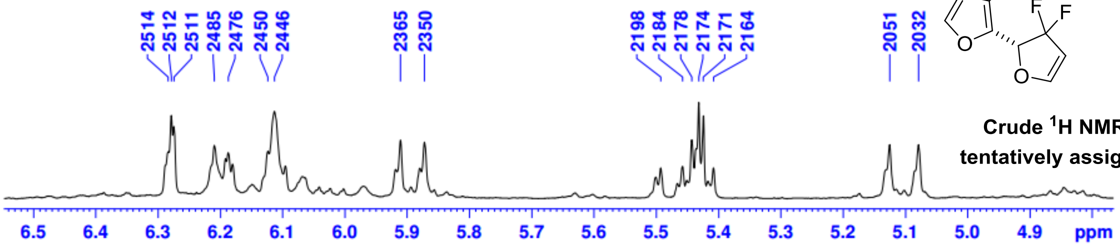


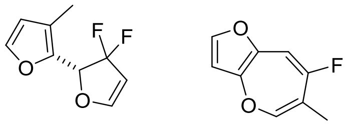


**Crude $^{1}$H NMR containing tentatively assigned 33 and 34**

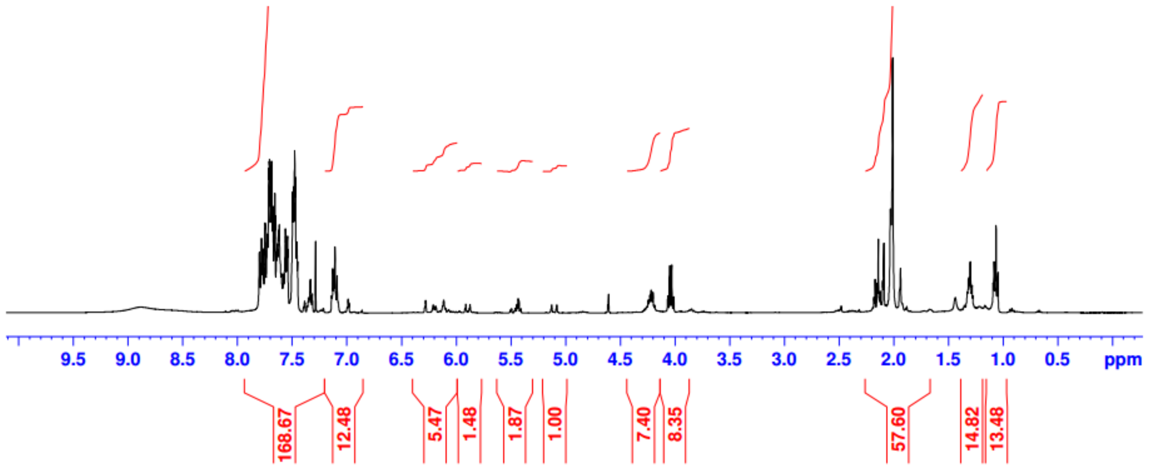

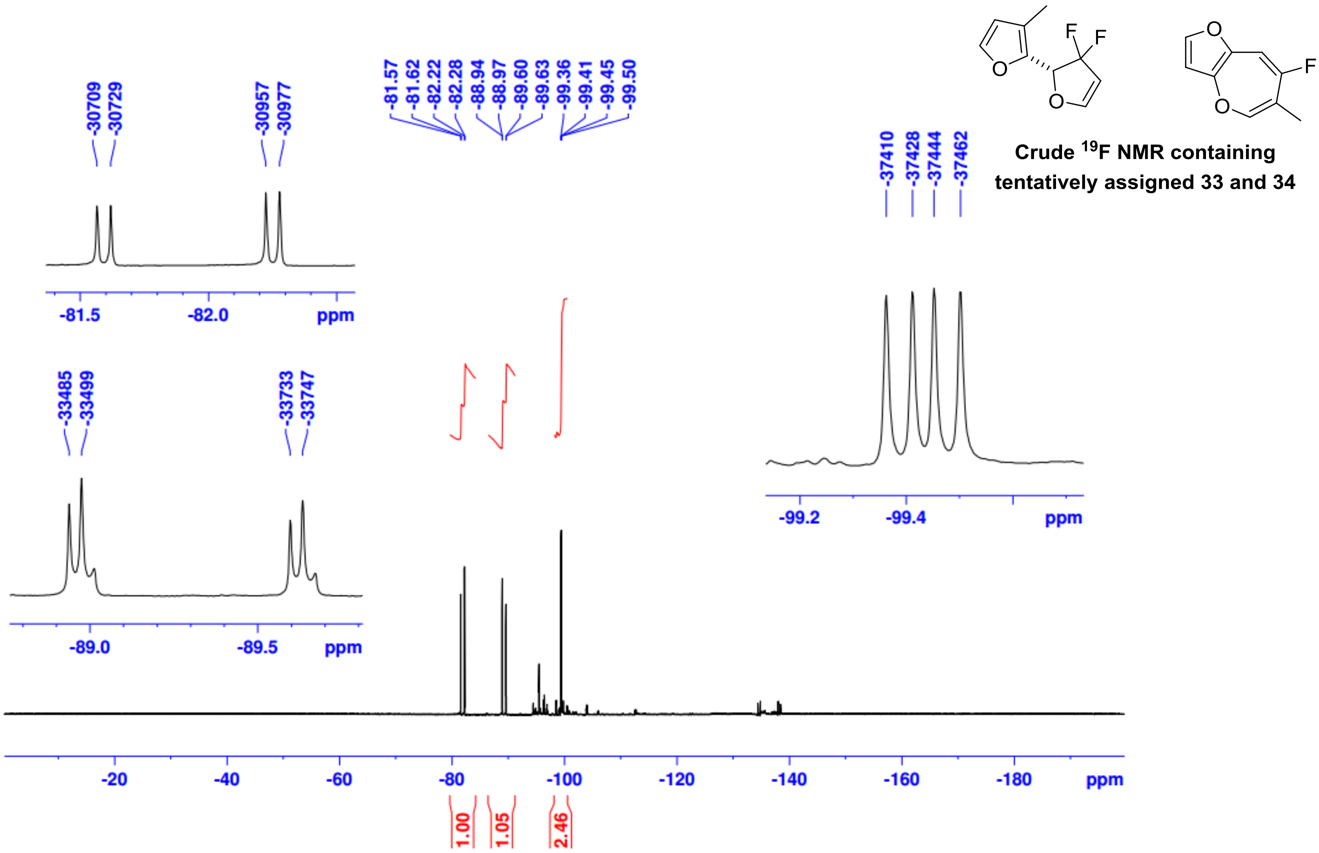


**Crude $^{19}$F NMR containing tentatively assigned 33 and 34**

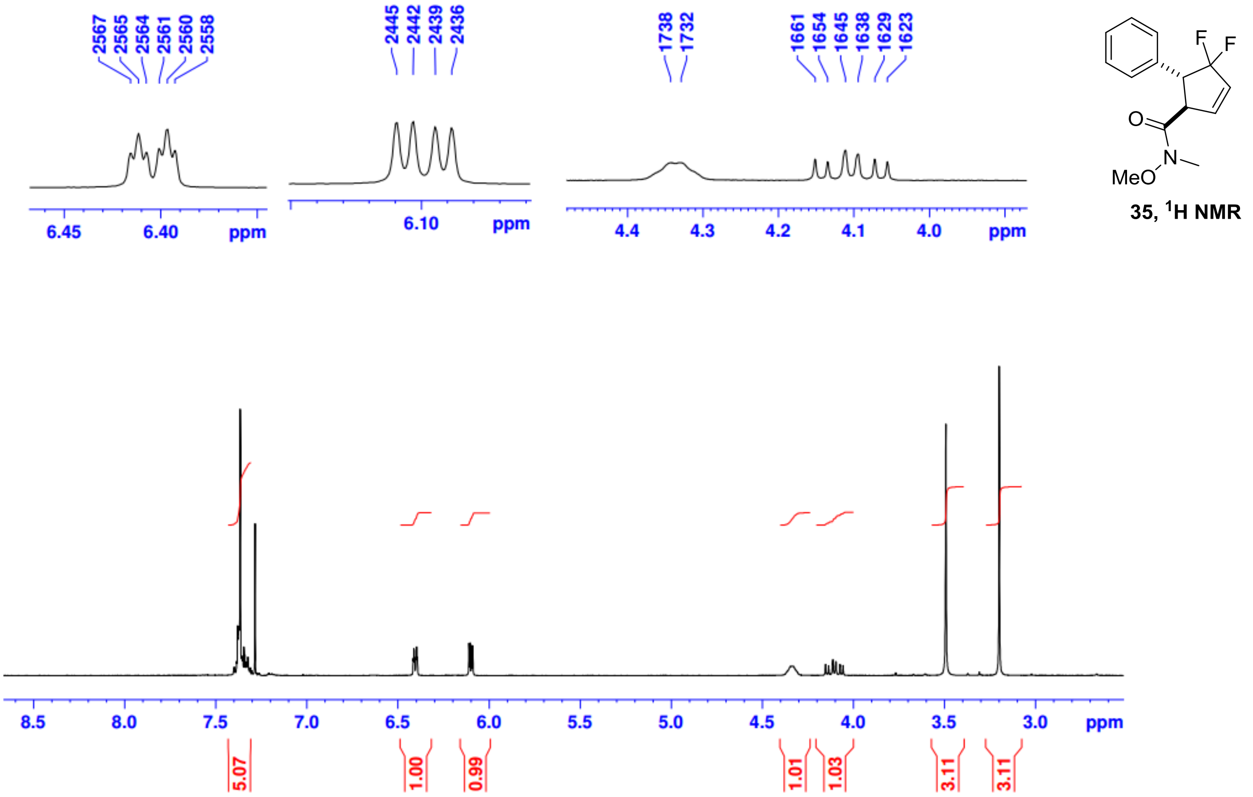


**35, $^{1}$H NMR**

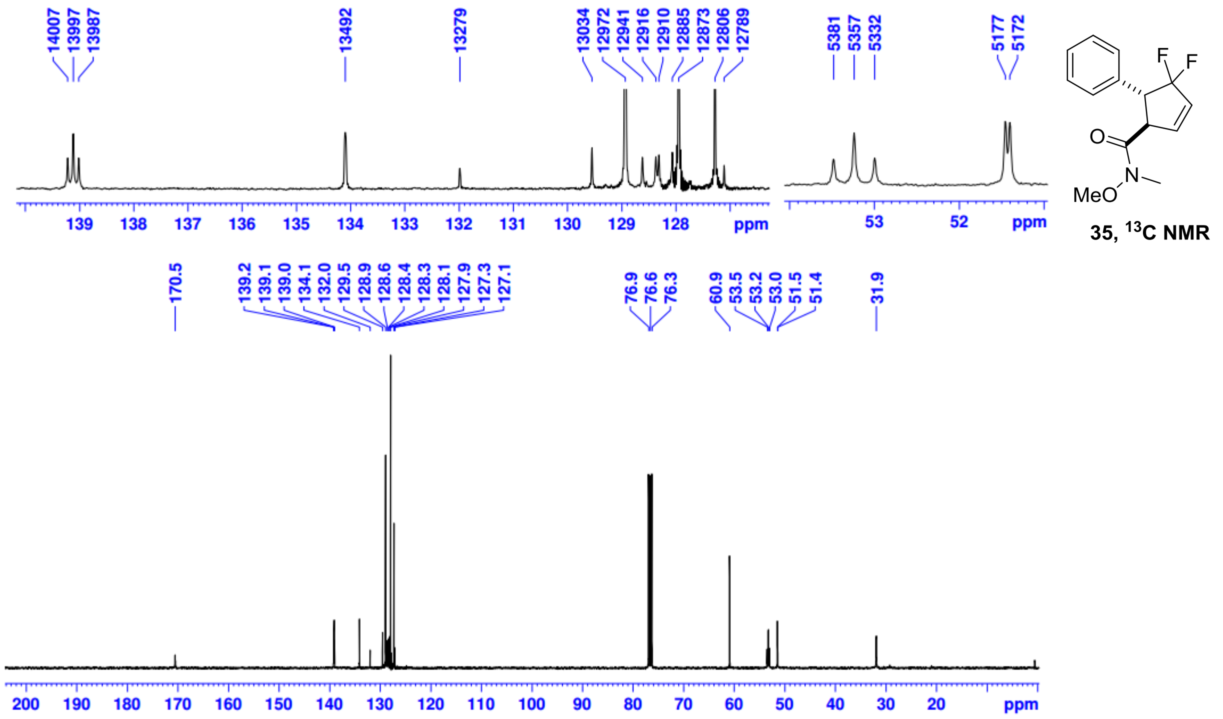

35, $^{13}C$ NMR

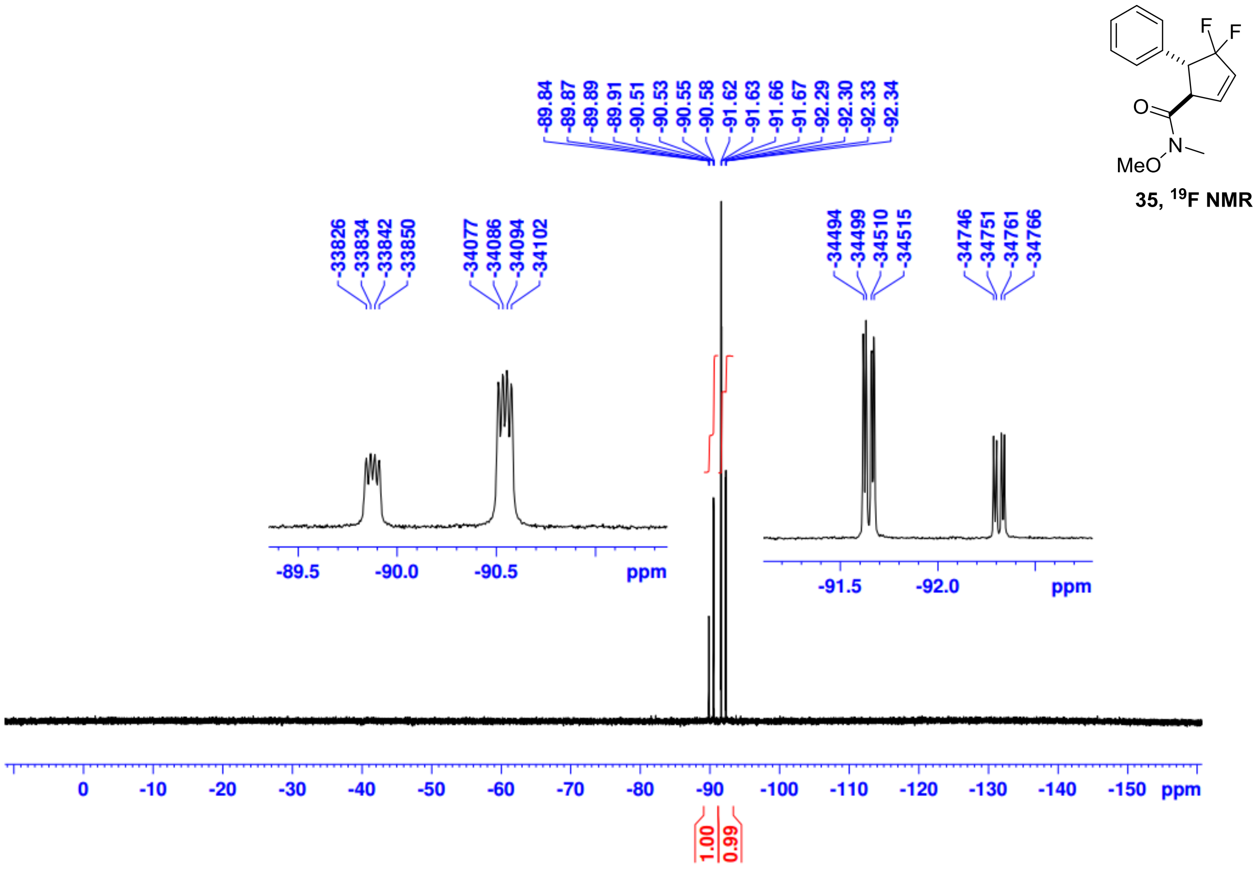

35, $^{19}F$ NMR

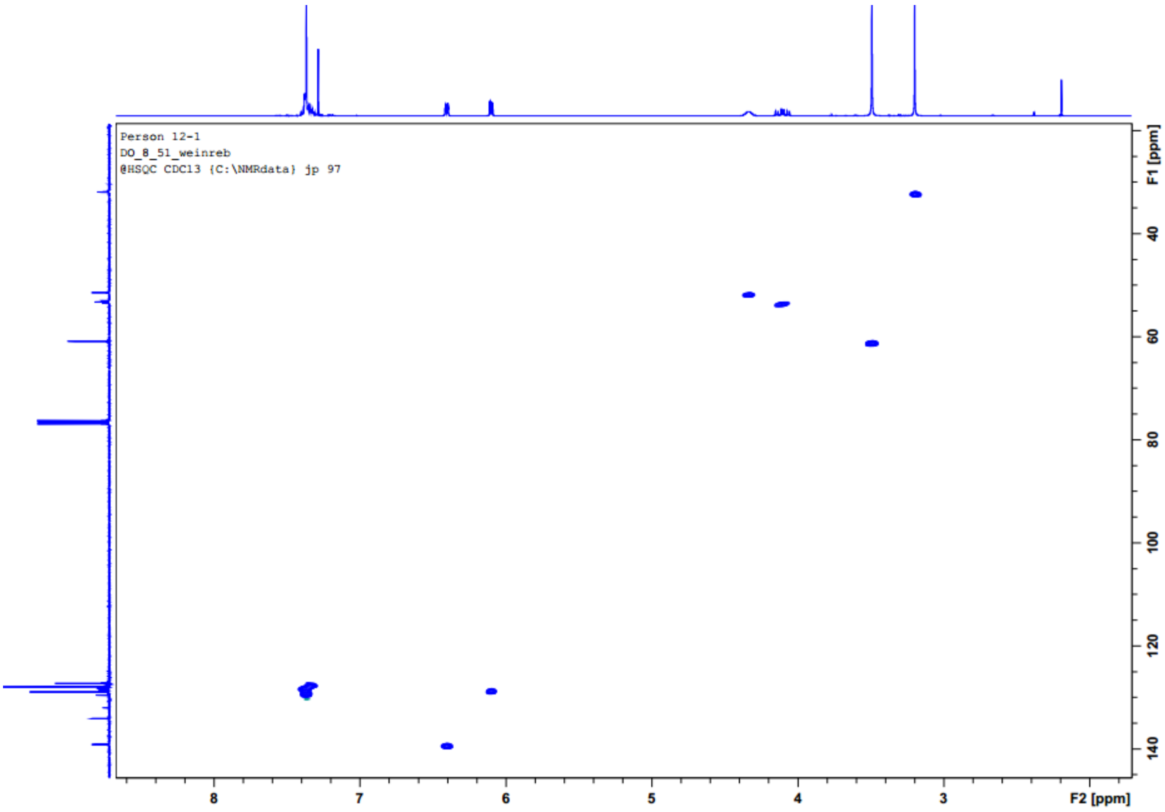


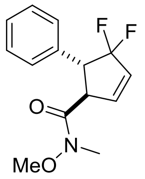


**35, HSQC**

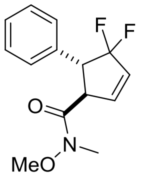


**35, HMBC**

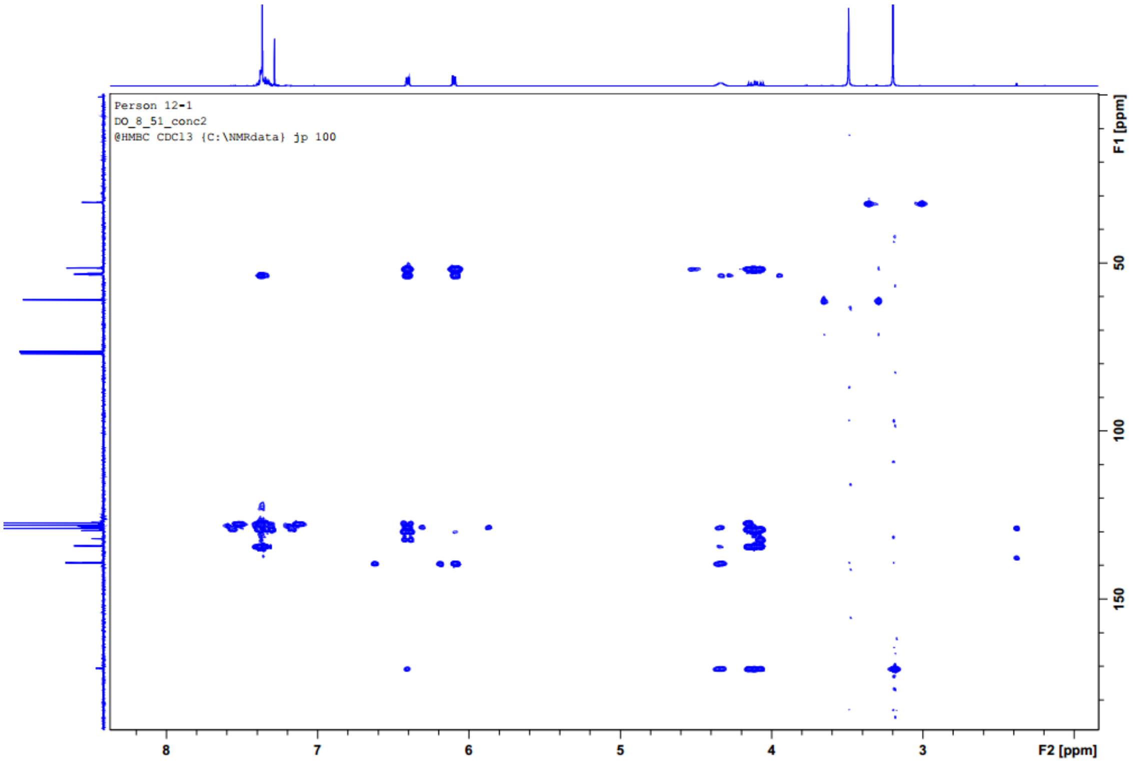

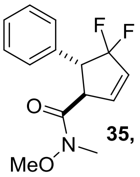


**35, NOESY**

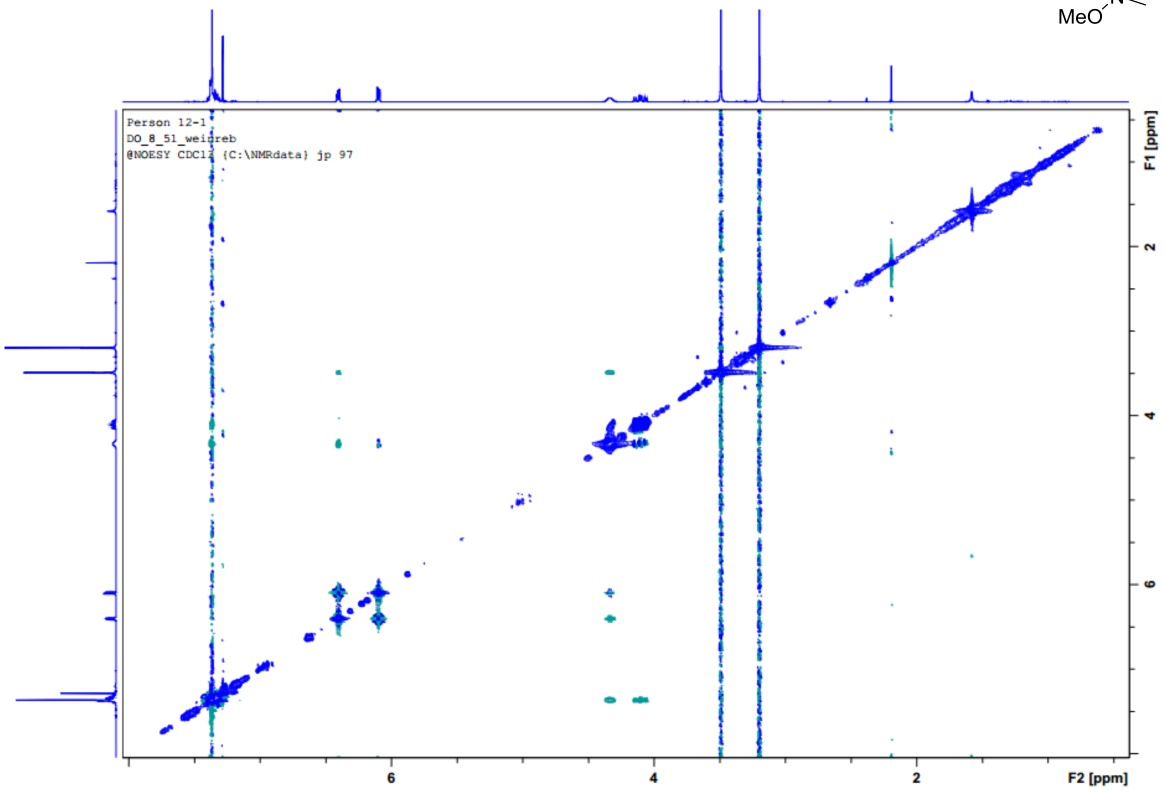


**unknown, S10a**
**$^{1}$H NMR**

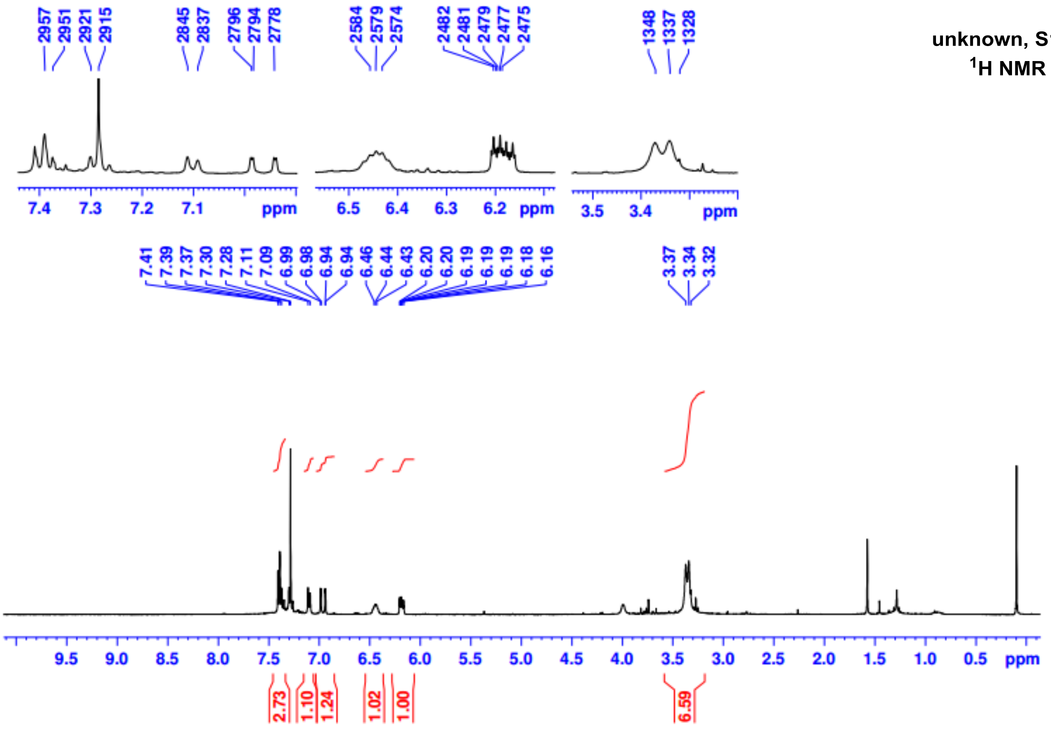

unknown, S10a
$^{19}F$ NMR

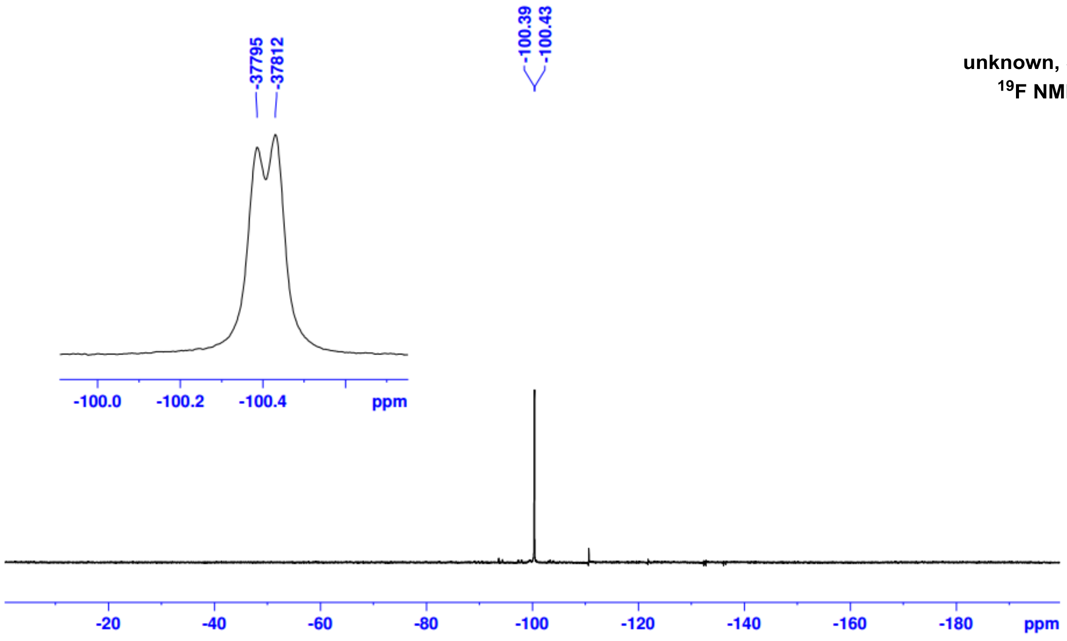


unknown, S10b
$^{1}H$ NMR

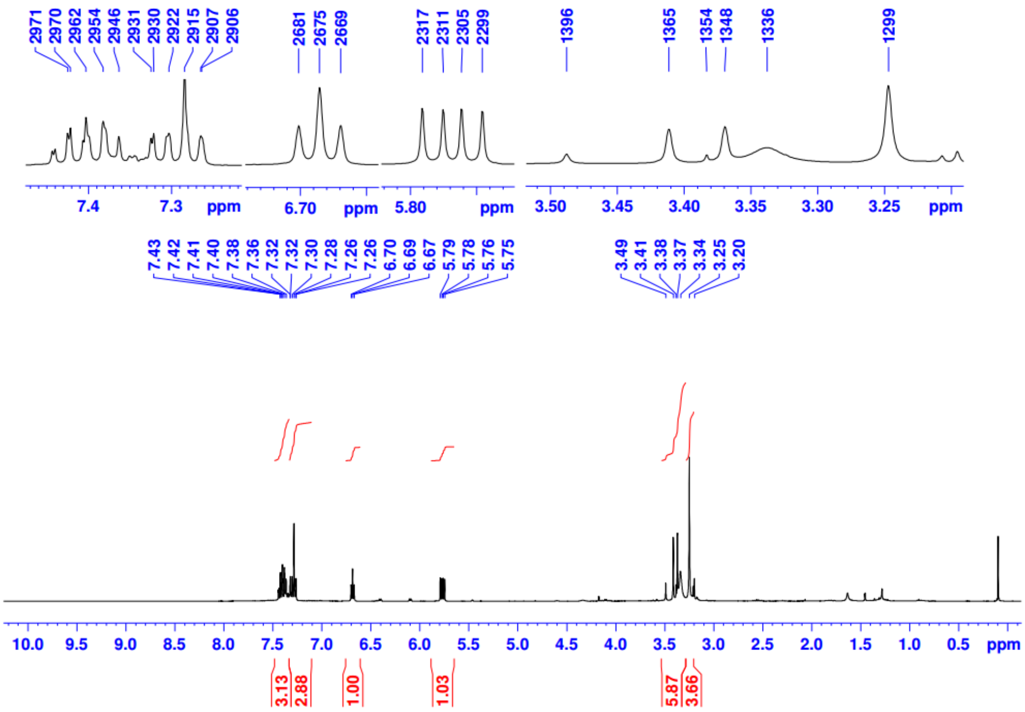

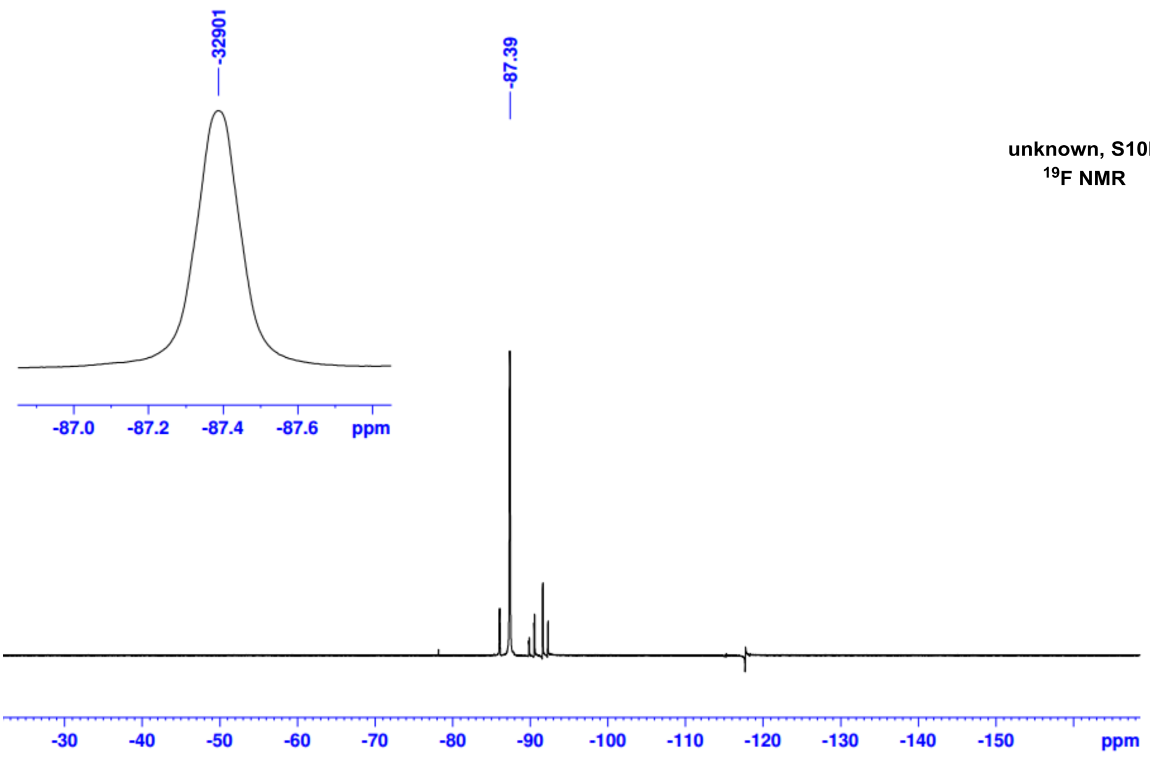


unknown, S10b
$^{19}$F NMR

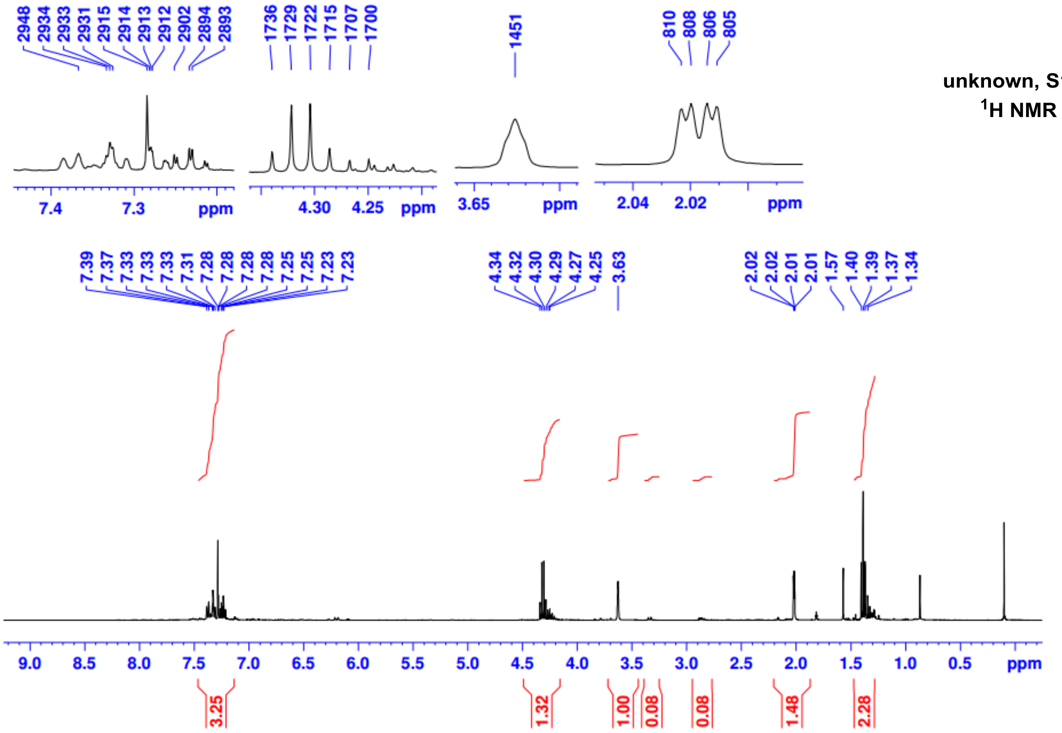


unknown, S11a
$^{1}$H NMR

unknown, S11a
$^{19}$F NMR

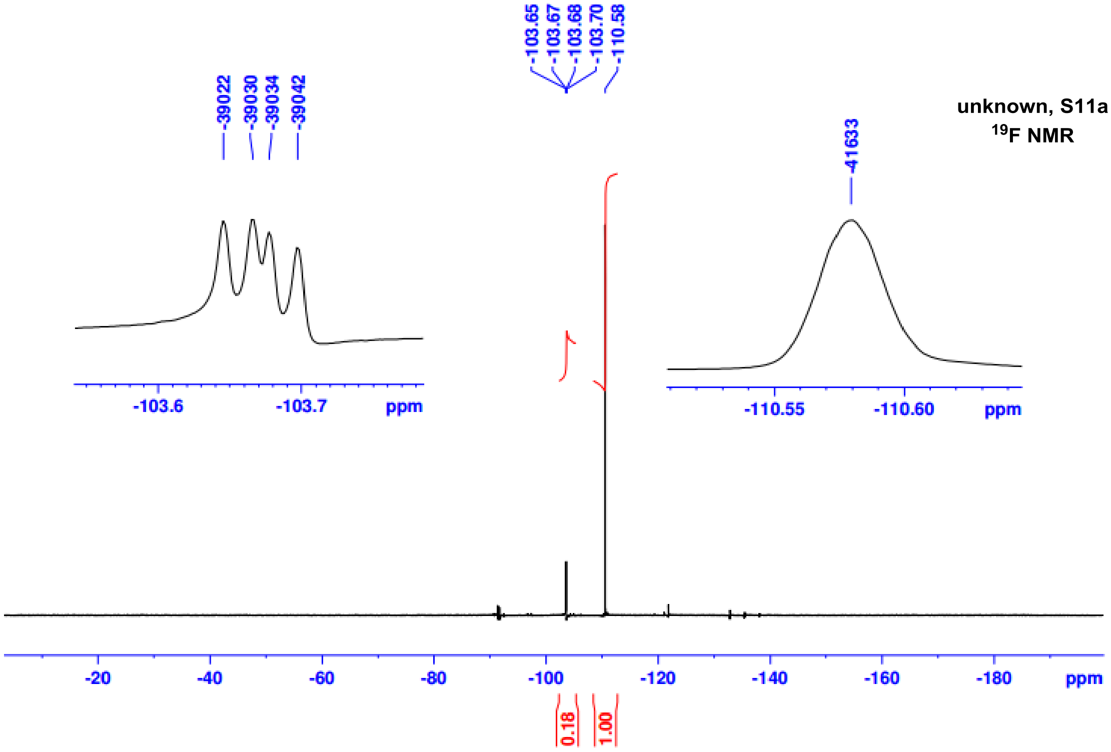

unknown, S11b
$^{1}$H NMR

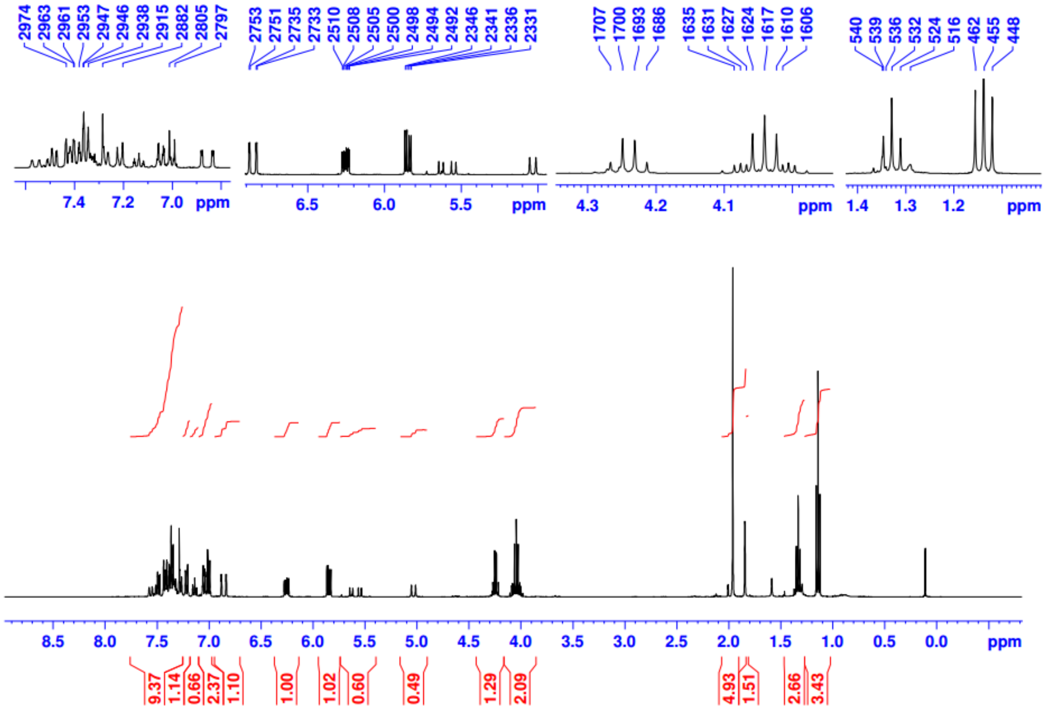

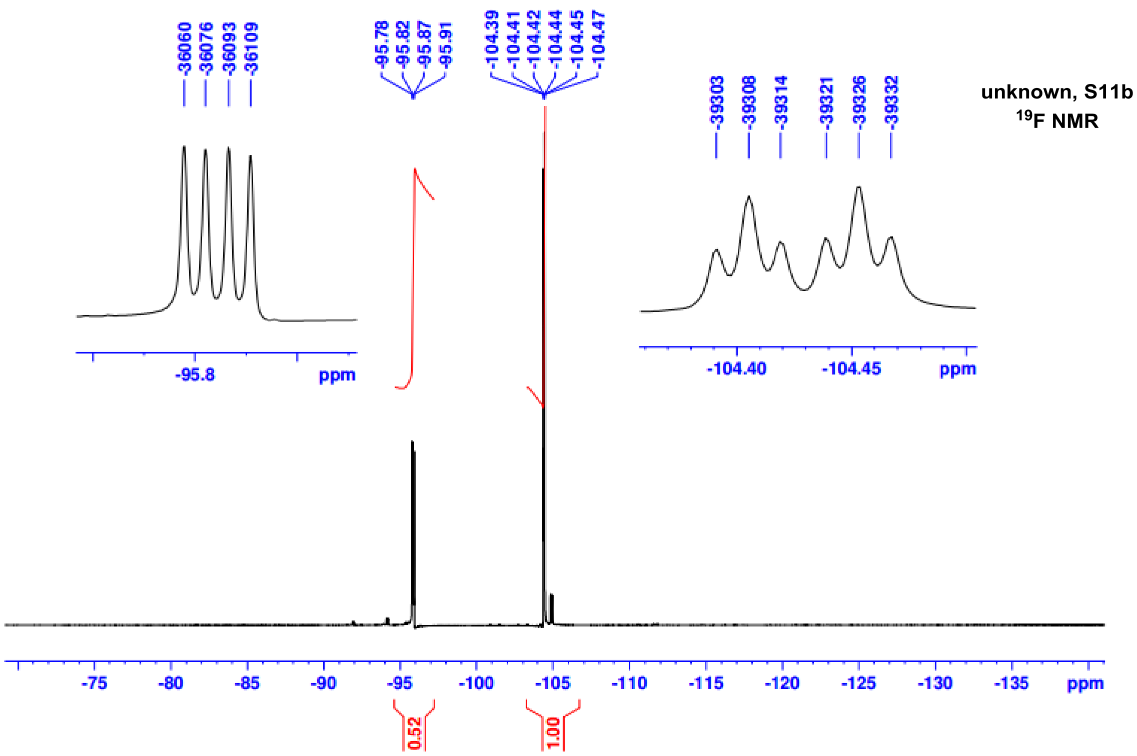

unknown, S11b
19F NMR


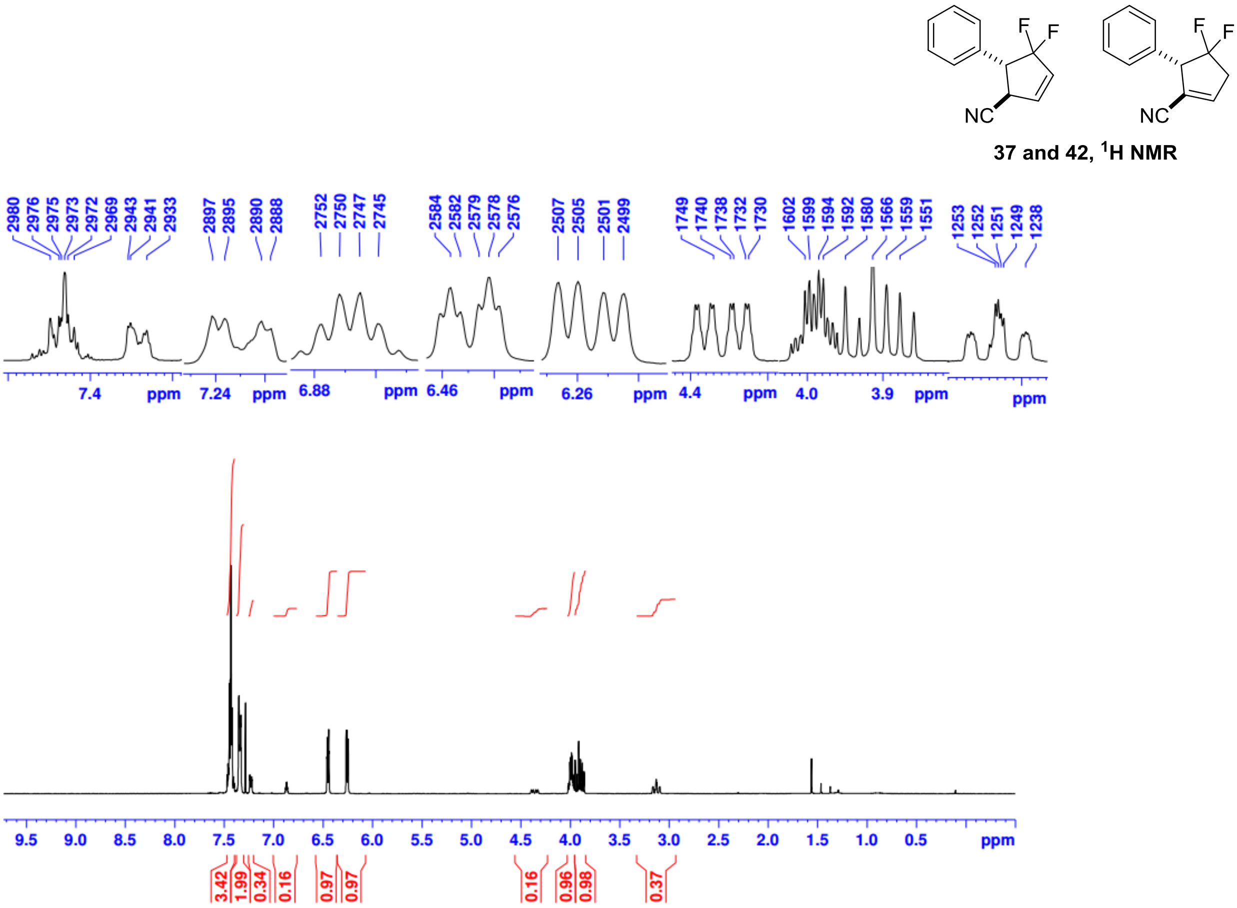

37 and 42, 1H NMR

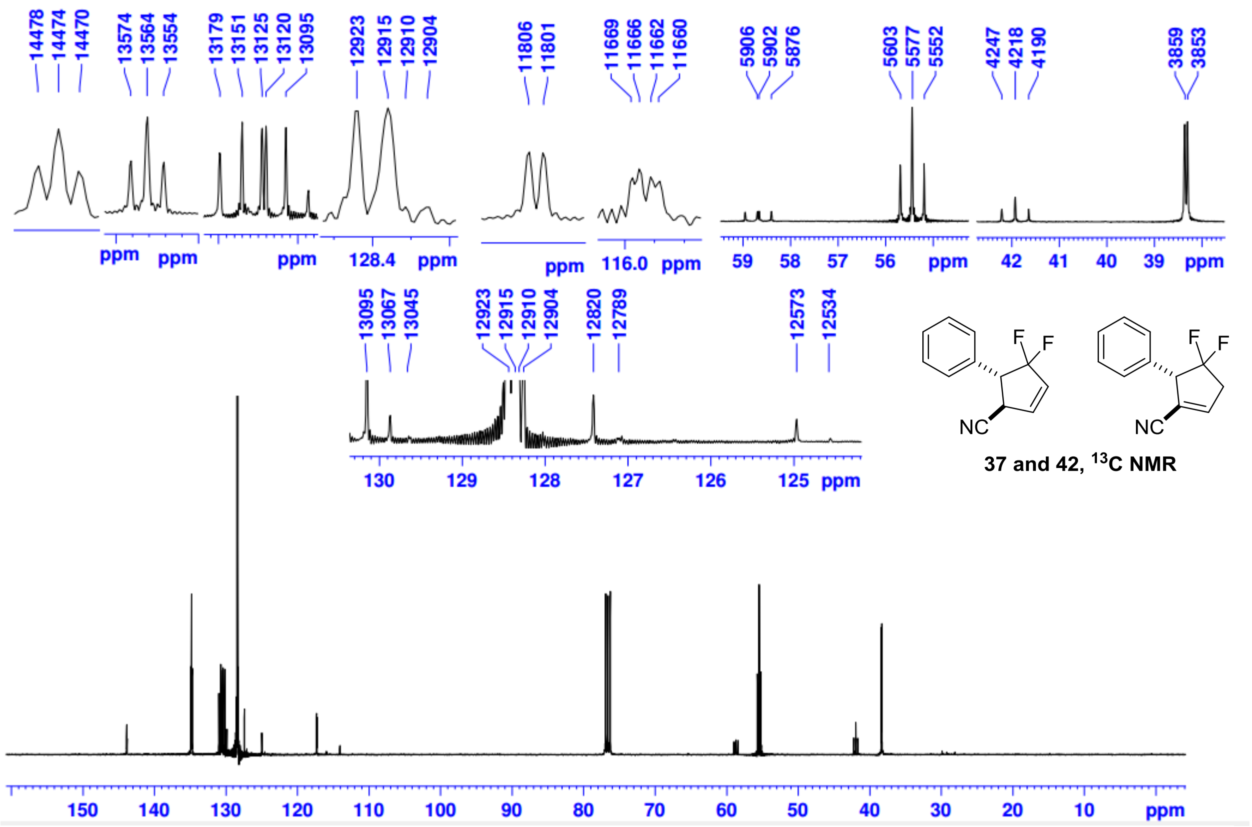
14478
14474
14470
13574
13564
13554
13179
13151
13125
13120
13095
12923
12915
12910
12904
11806
11801
11669
11666
11662
11660
5906
5902
5876
5603
5577
5552
4247
4218
4190
3859
3853
13095
13067
13045
12923
12915
12910
12904
12820
12789
12573
12534
F
F
NC
37 and 42, 13C NMR
ppm

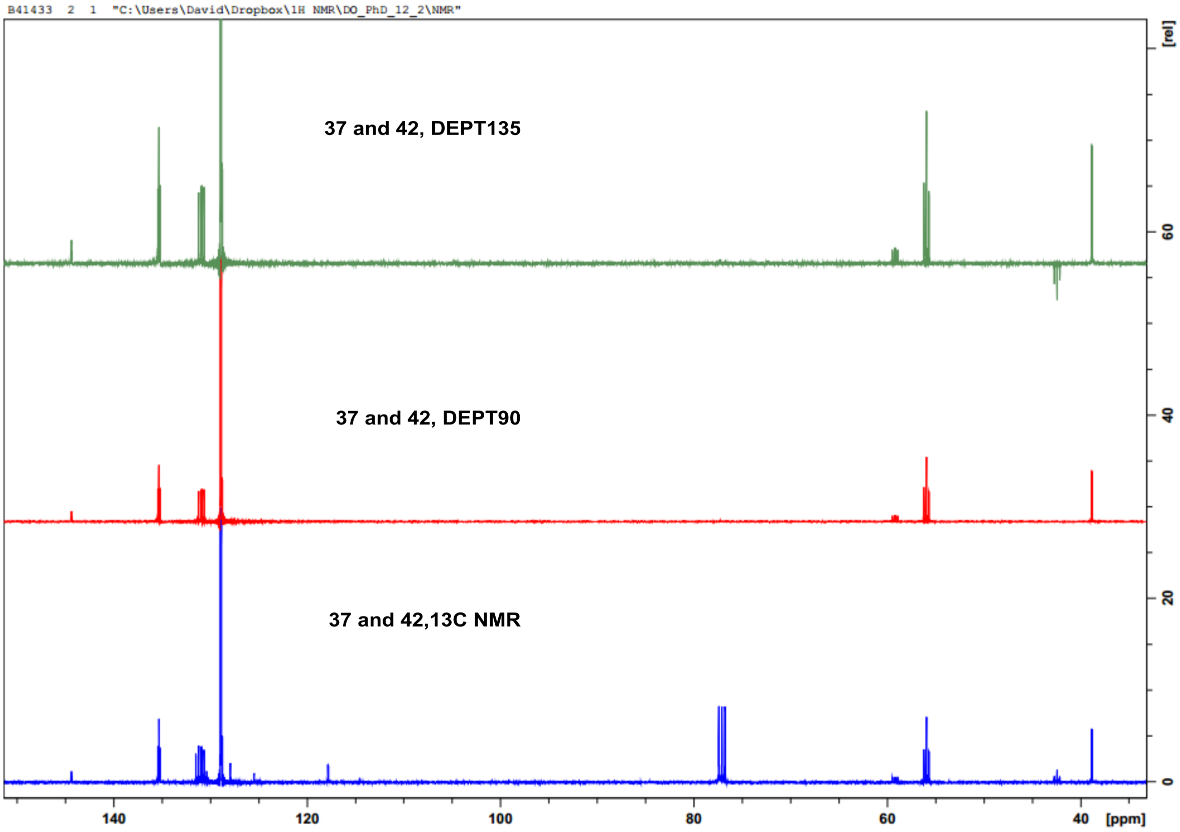
B41433 2 1 "C:\Users\David\Dropbox\1H NMR\DO_PhD_12_2\NMR"
37 and 42, DEPT135
37 and 42, DEPT90
37 and 42,13C NMR
[rel]
[ppm]

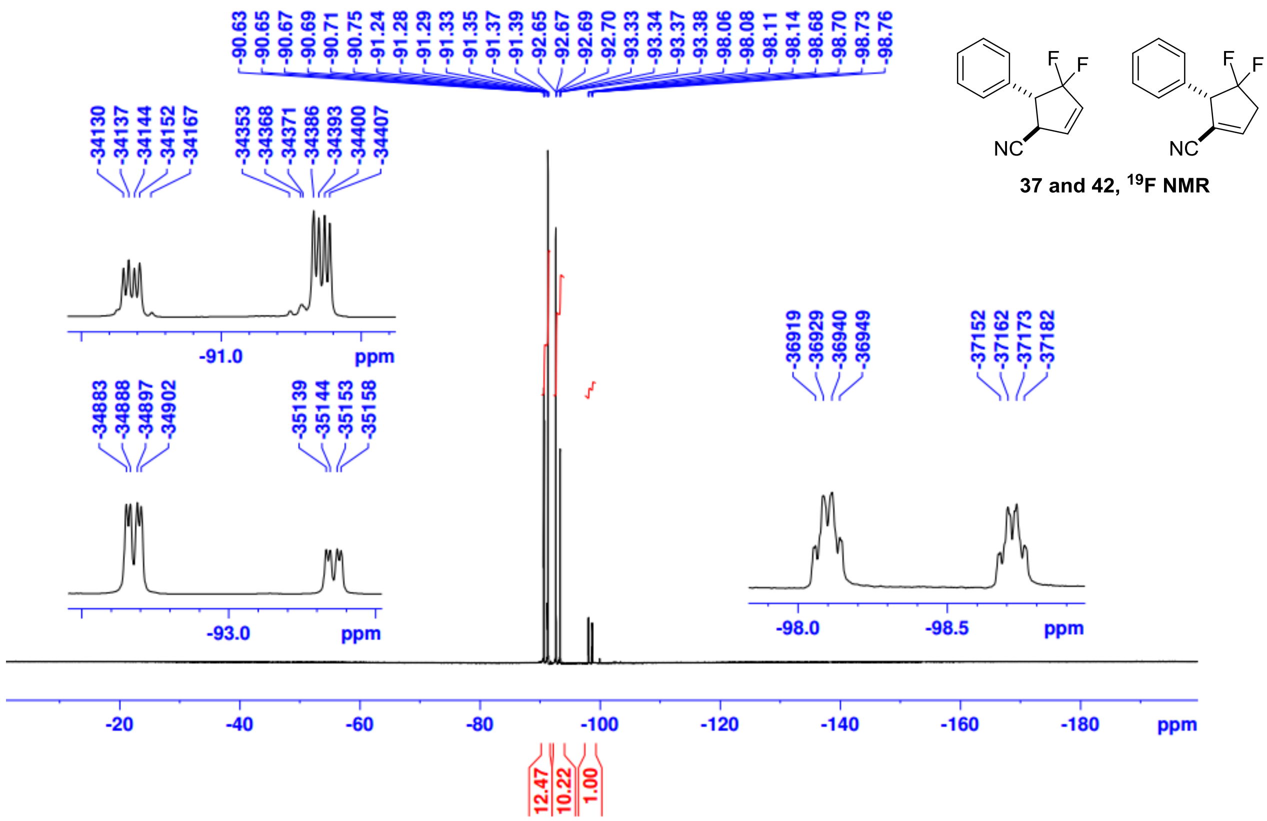
37 and 42, $^{19}$F NMR
-91.0
ppm
-93.0
ppm
-98.0
-98.5
ppm
-20
-40
-60
-80
-100
-120
-140
-160
-180
ppm
12.47
10.22
1.00

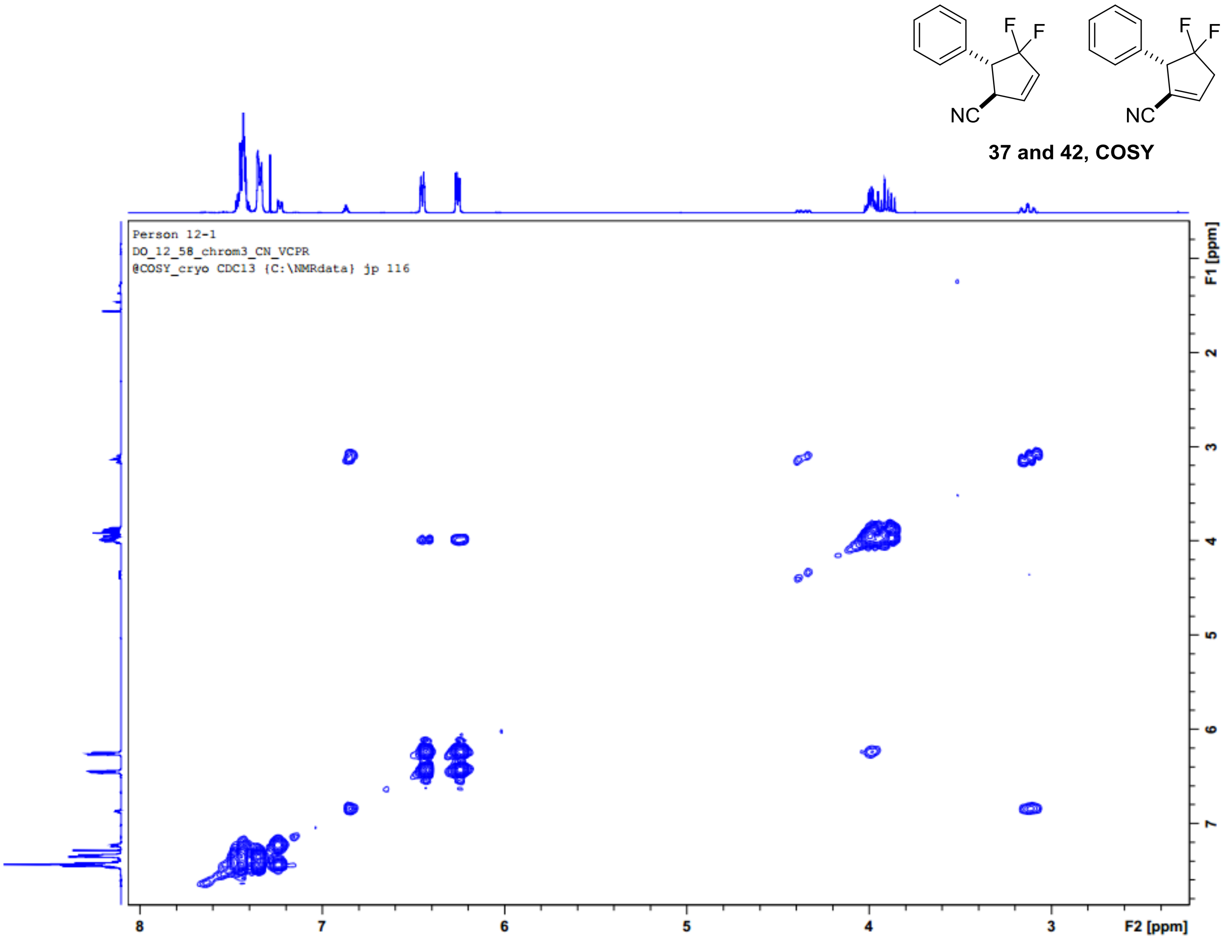
37 and 42, COSY
Person 12-1
DO_12_58_chrom3_CN_VCPR
@COSY_cryo CDCl3 {C:\NMRdata} jp 116
F1 [ppm]
F2 [ppm]

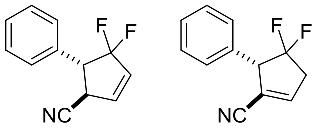


**37 and 42, HSQC**

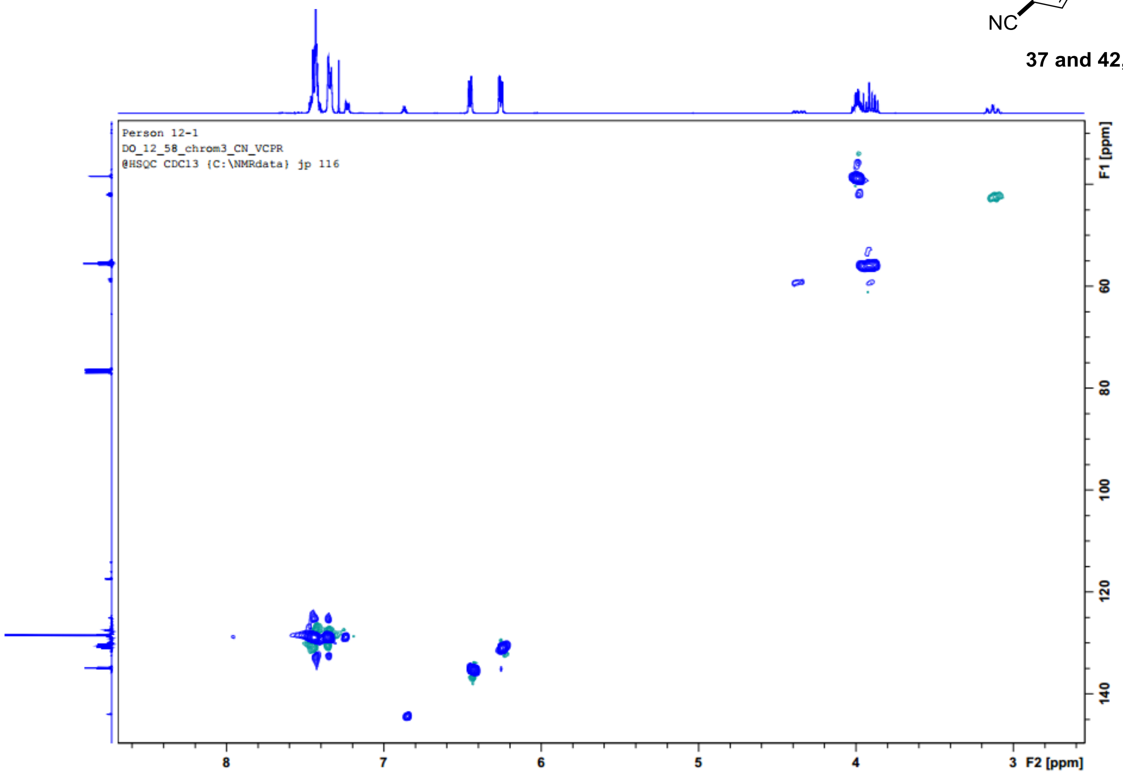


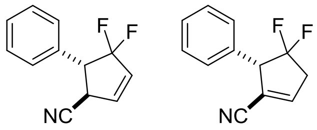


**37 and 42, HMBC**

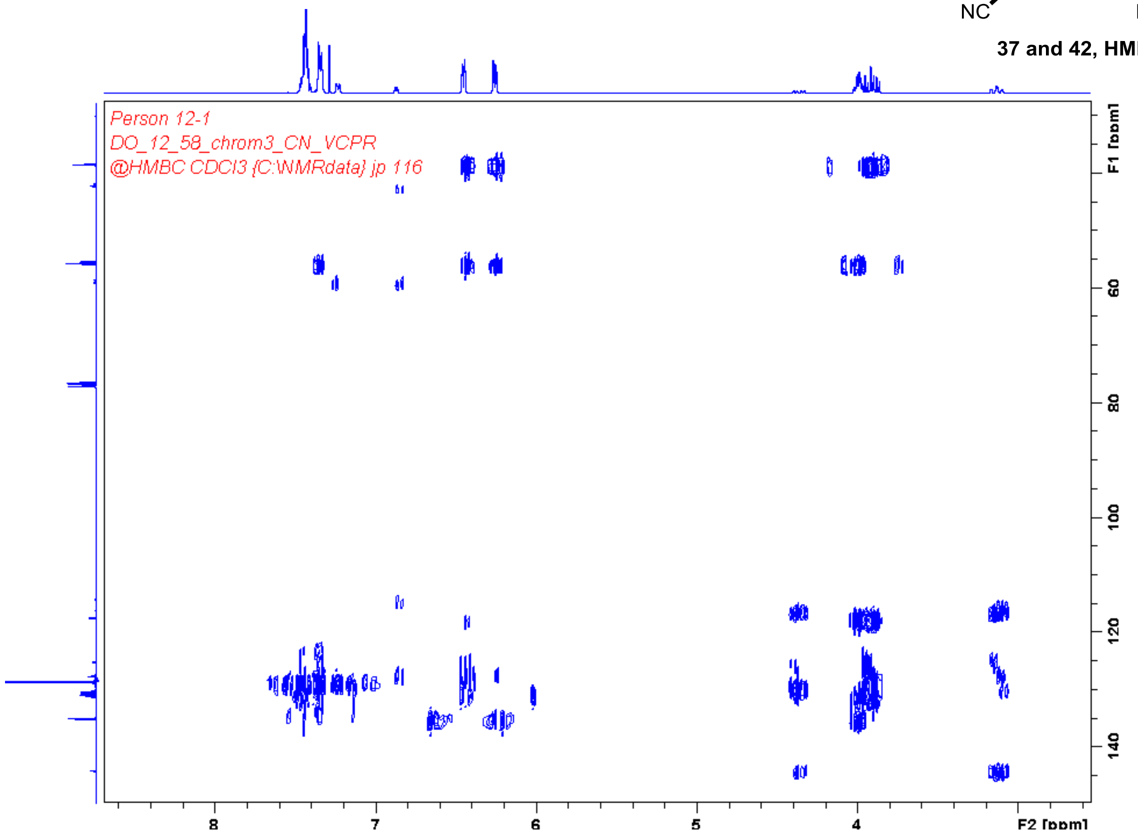

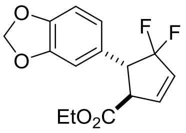


**39, $^{1}$H NMR**

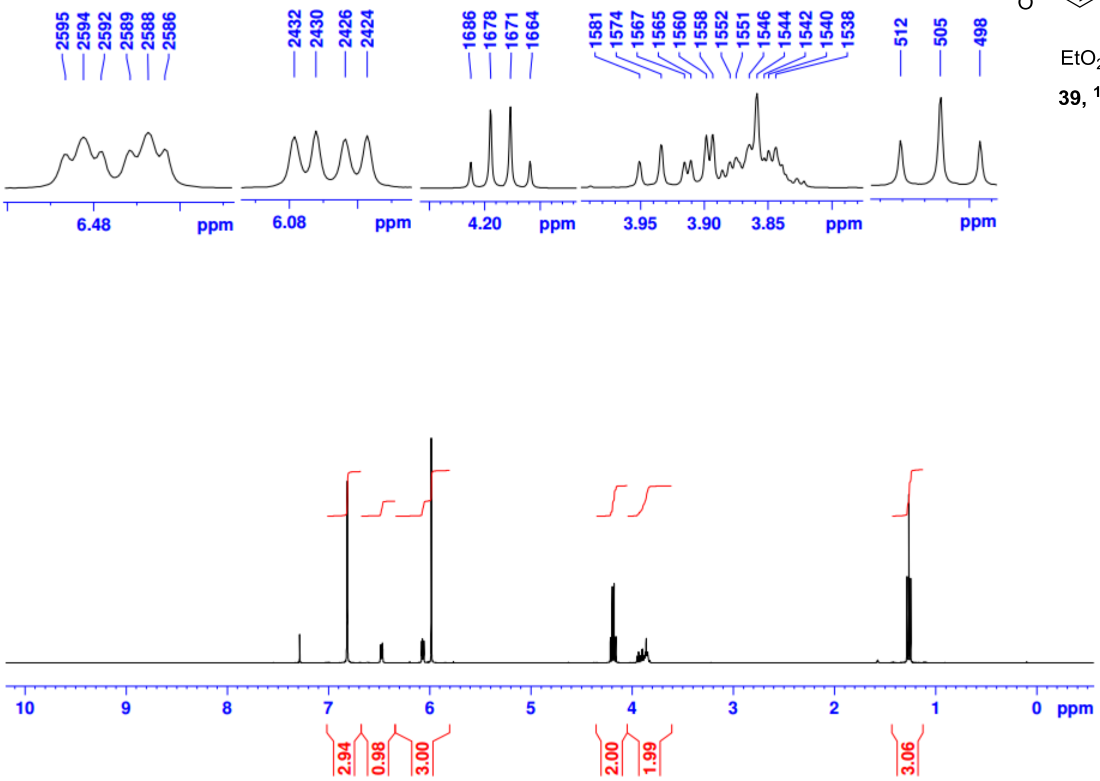


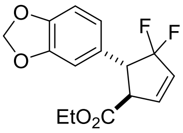


**39, $^{13}$C NMR**

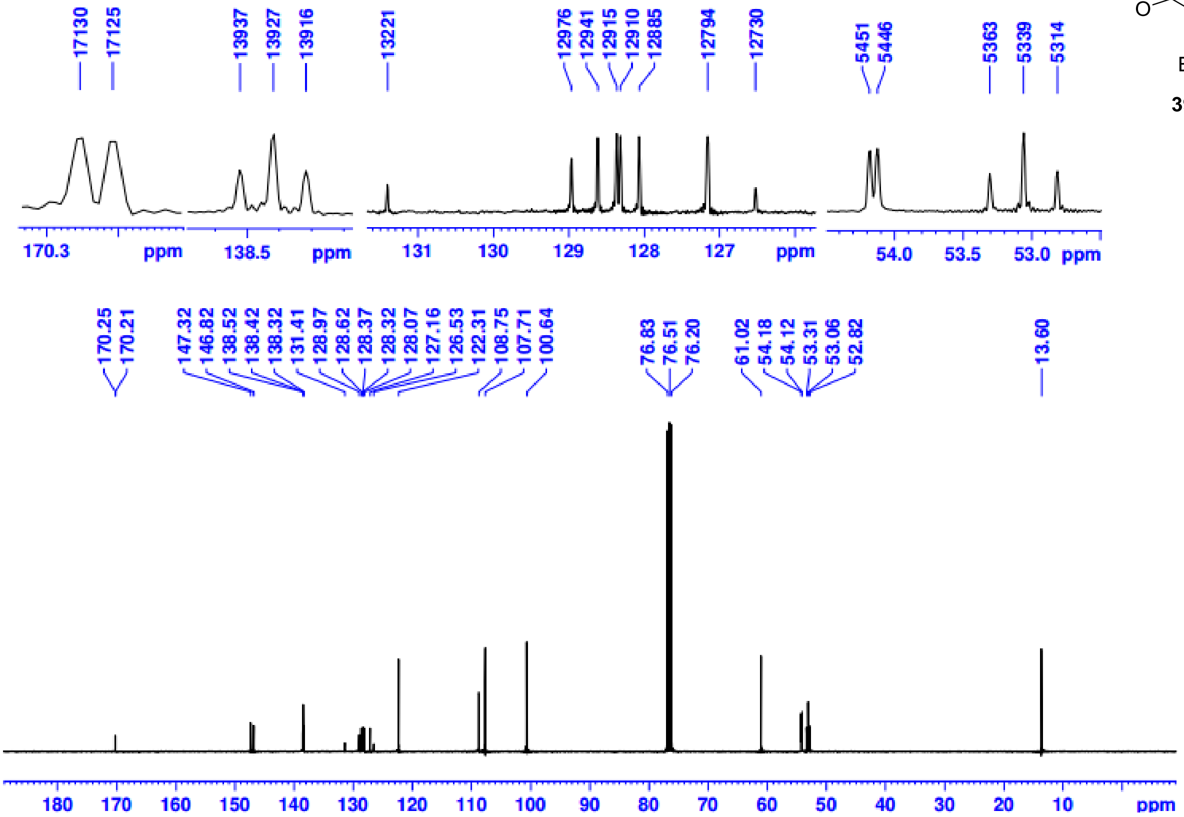

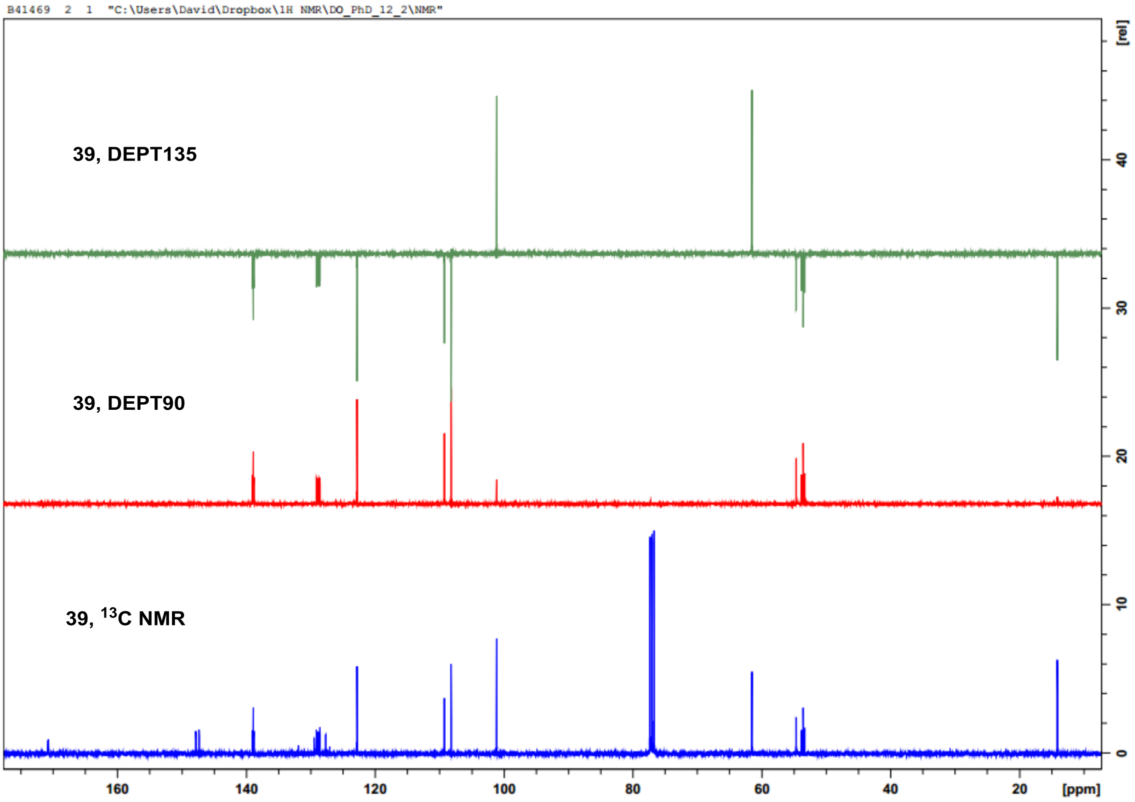
B41469 2 1 "C:\Users\David\Dropbox\1H NMR\DO_PhD_12_2\NMR"
39, DEPT135
39, DEPT90
39, 13C NMR
[rel]
[ppm]

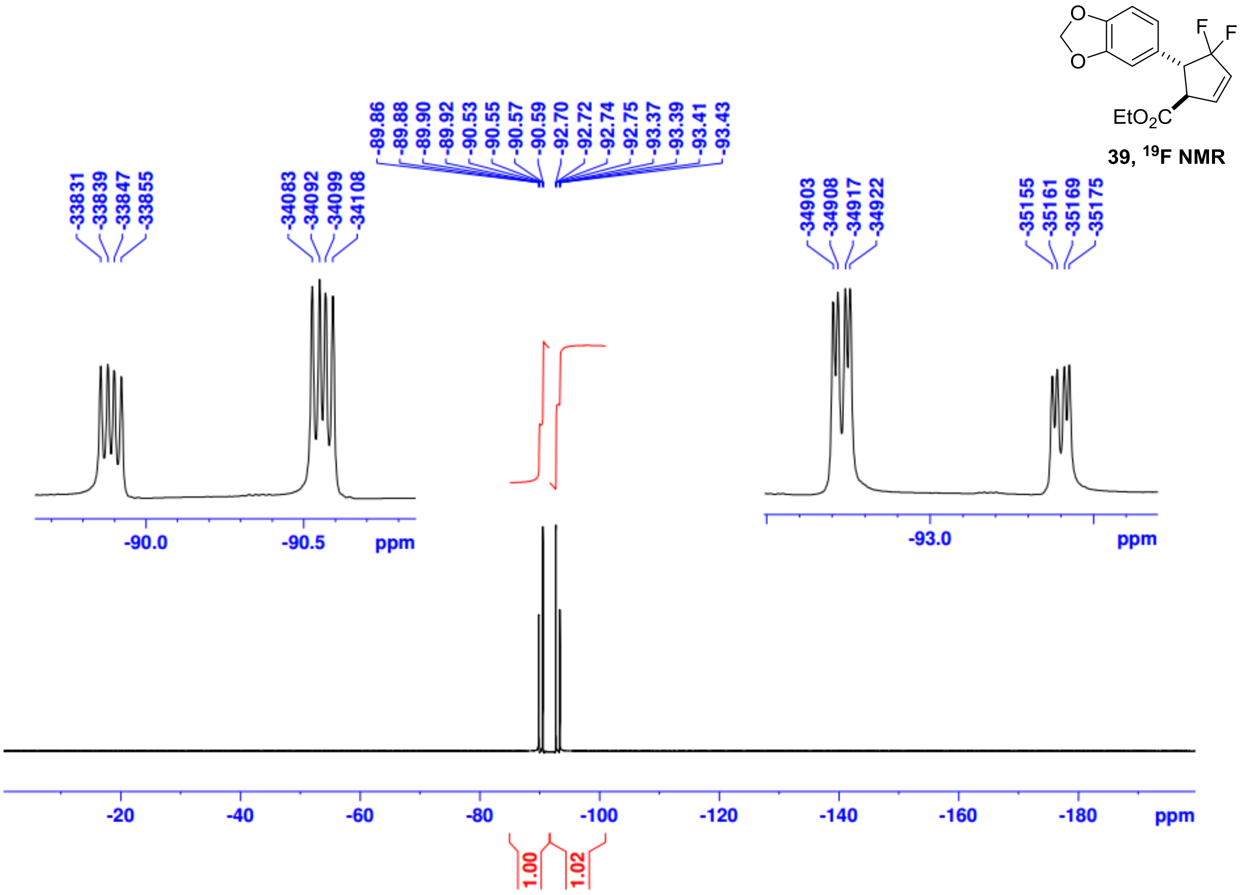
F
F
O
O
EtO2C
39, 19F NMR
-89.86
-89.88
-89.90
-89.92
-90.53
-90.55
-90.57
-90.59
-92.70
-92.72
-92.74
-92.75
-93.37
-93.39
-93.41
-93.43
-33831
-33839
-33847
-33855
-34083
-34092
-34099
-34108
-34903
-34908
-34917
-34922
-35155
-35161
-35169
-35175
ppm
1.00
1.02

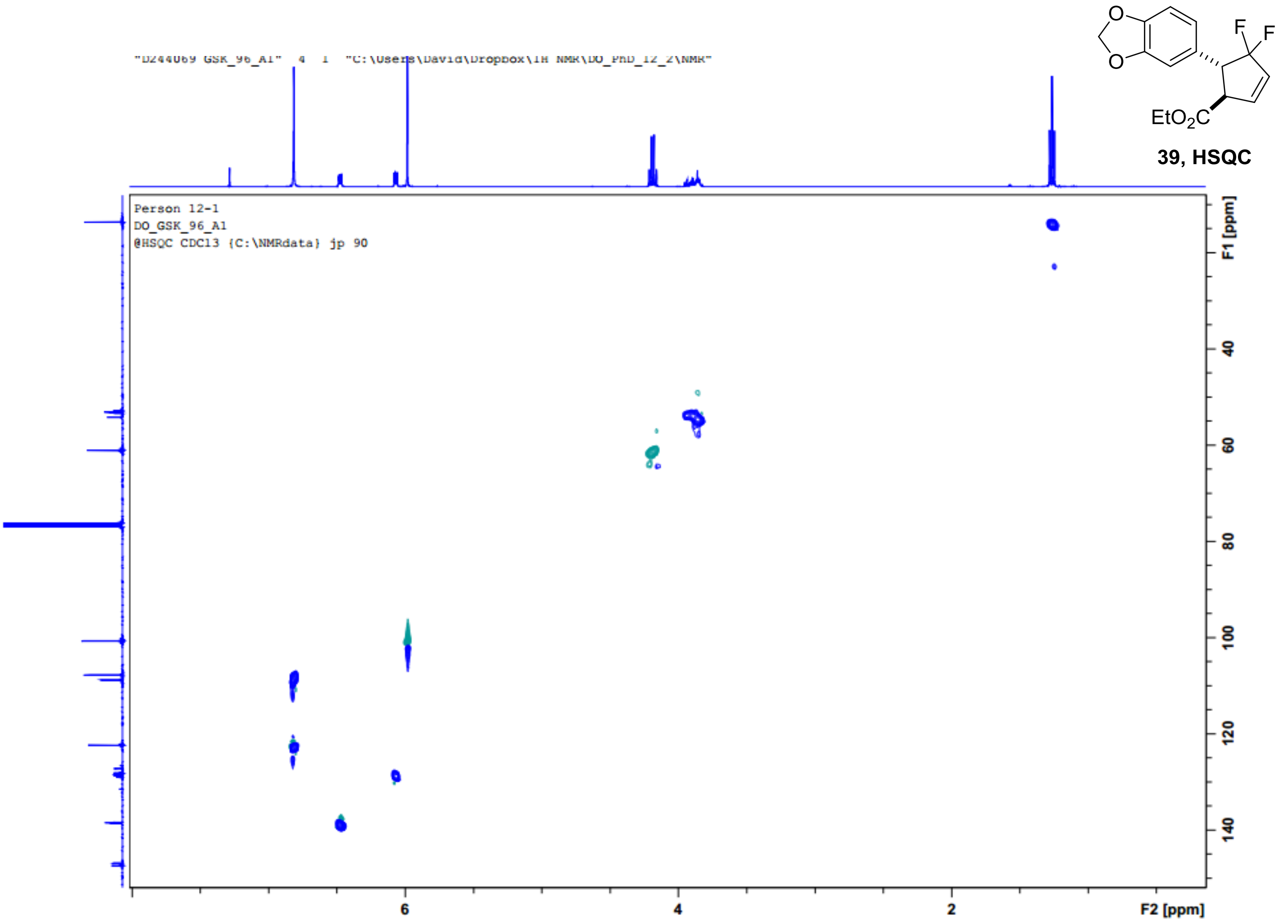


39, HSQC

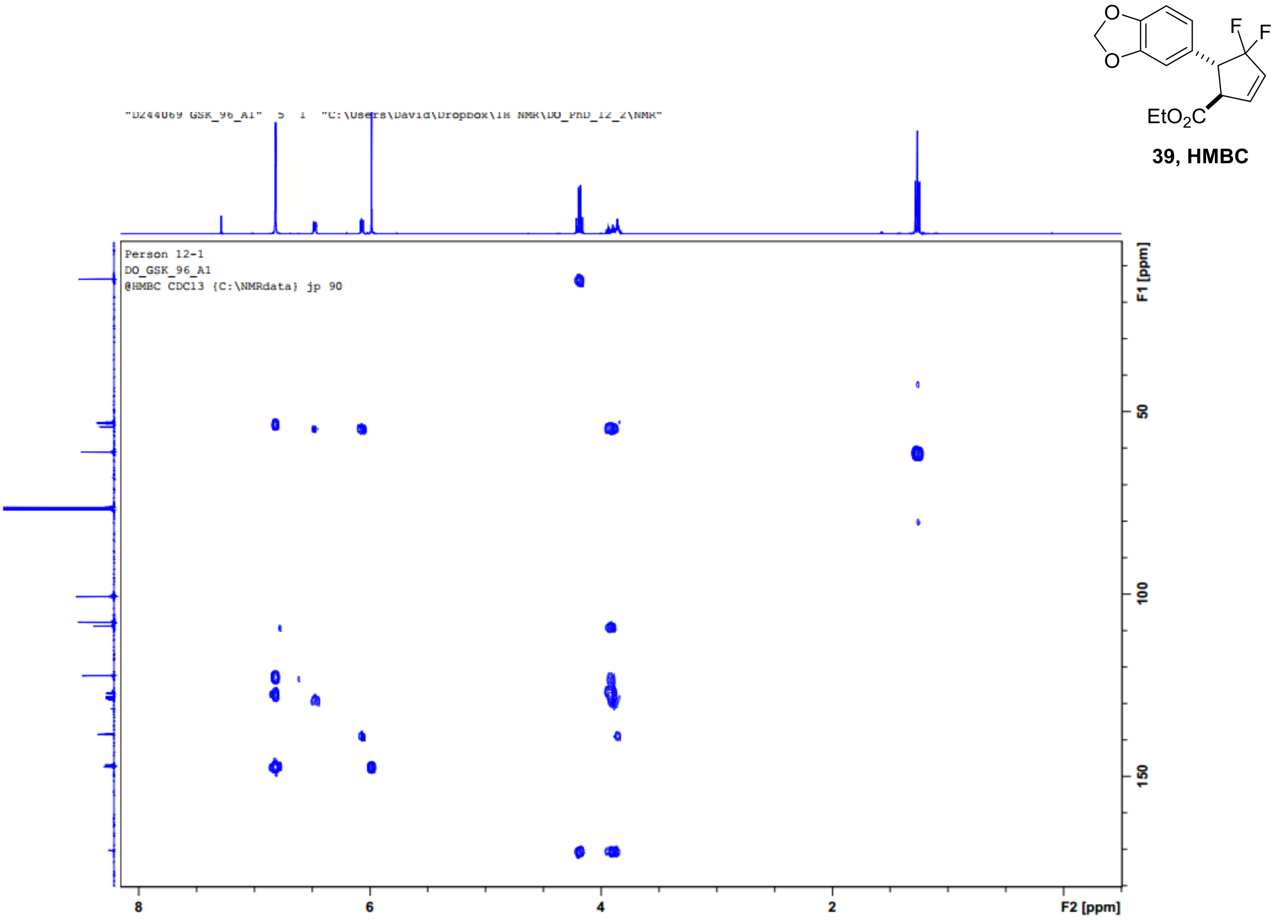


39, HMBC

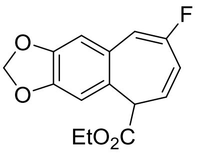


**44, $^1$H NMR**

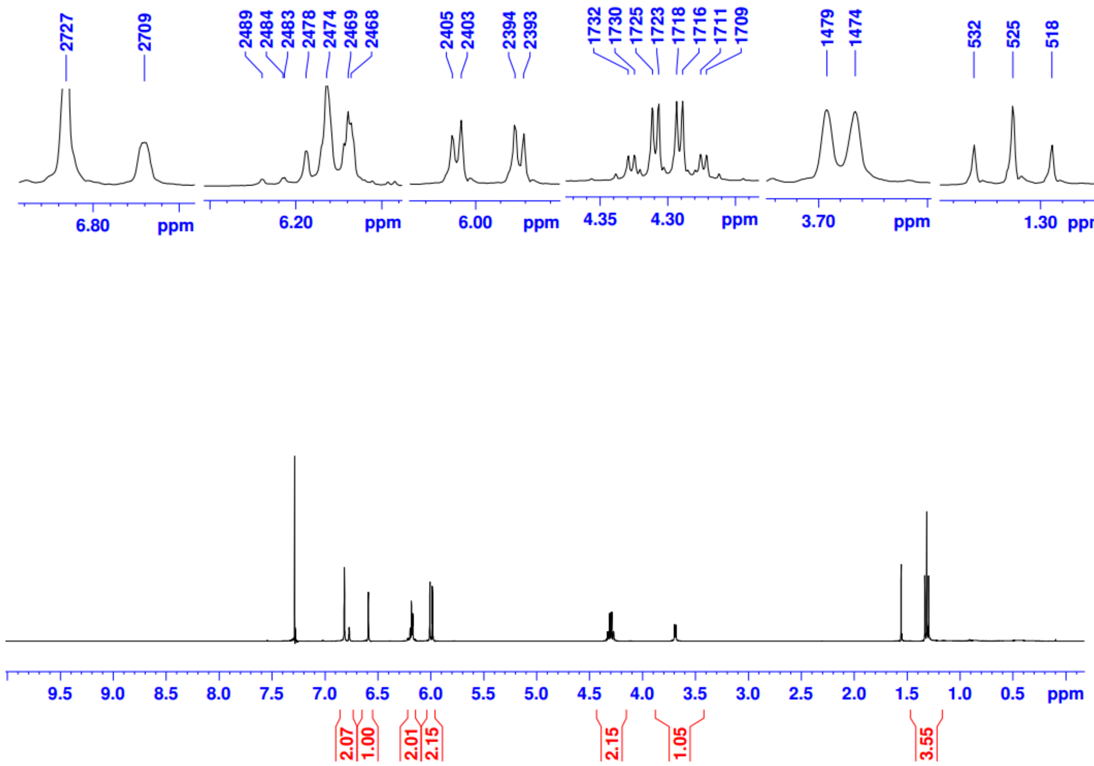


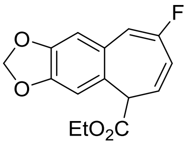


**44, $^{13}$C NMR**

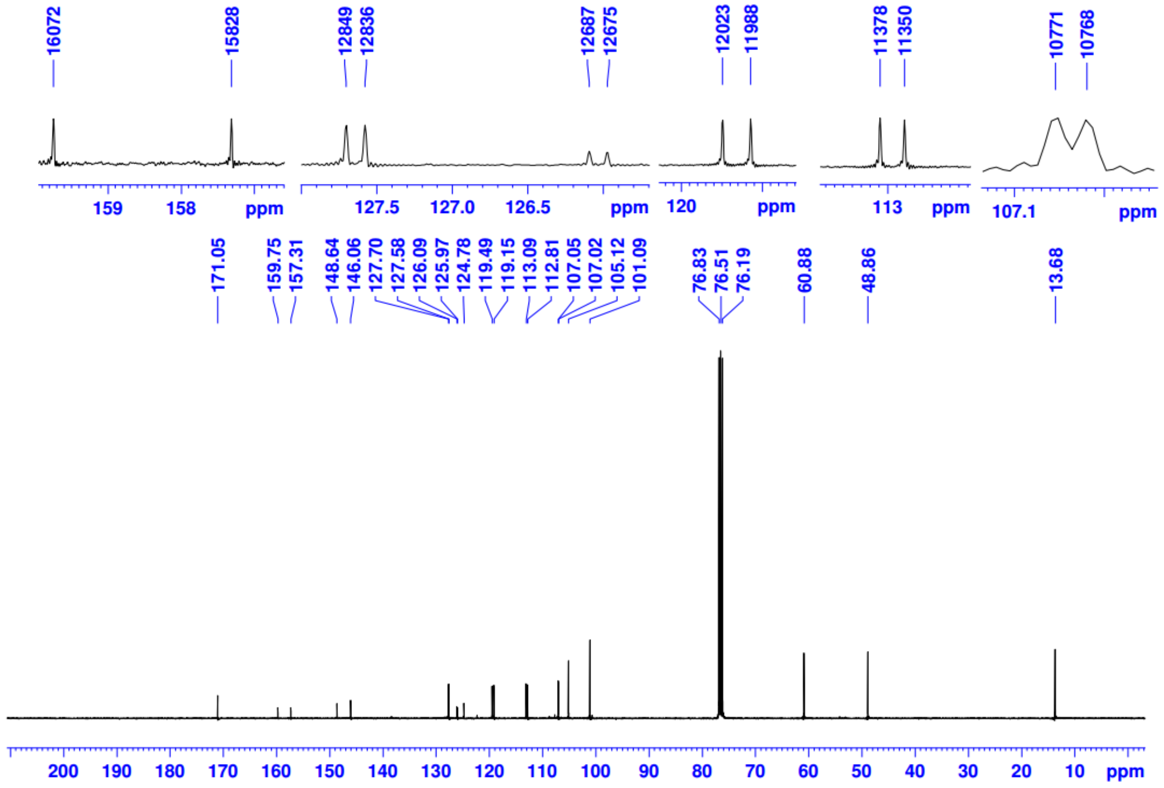

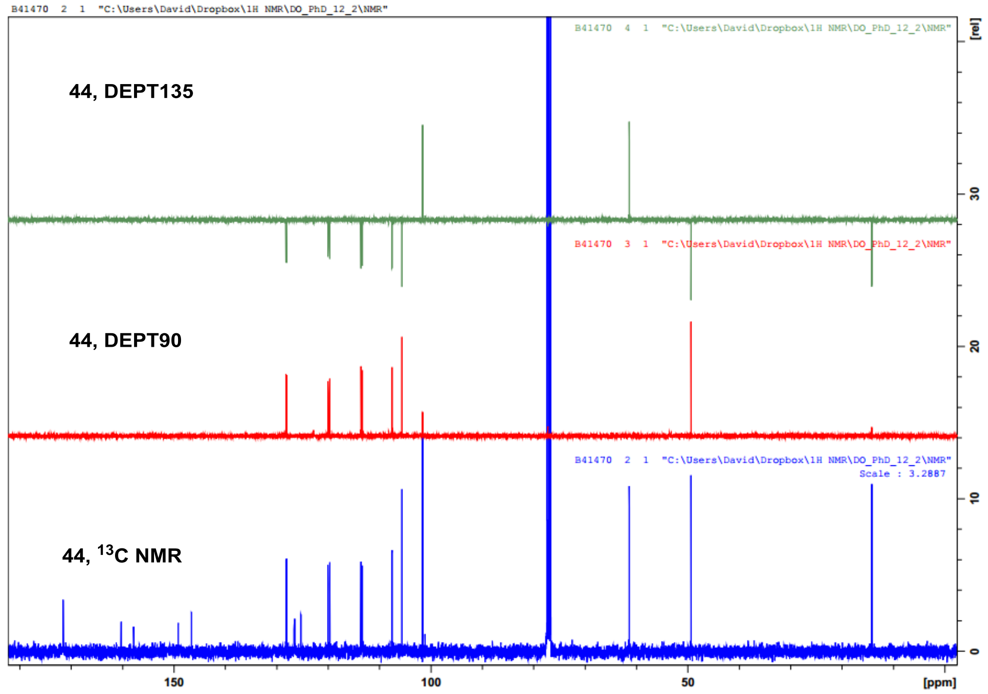

B41470 2 1 "C:\Users\David\Dropbox\1H NMR\DO_PhD_12_2\NMR"
B41470 4 1 "C:\Users\David\Dropbox\1H NMR\DO_PhD_12_2\NMR"
44, DEPT135
B41470 3 1 "C:\Users\David\Dropbox\1H NMR\DO_PhD_12_2\NMR"
44, DEPT90
B41470 2 1 "C:\Users\David\Dropbox\1H NMR\DO_PhD_12_2\NMR"
Scale : 3.2887
44, 13C NMR
150
100
50
[ppm]
[rel]
30
20
10
0


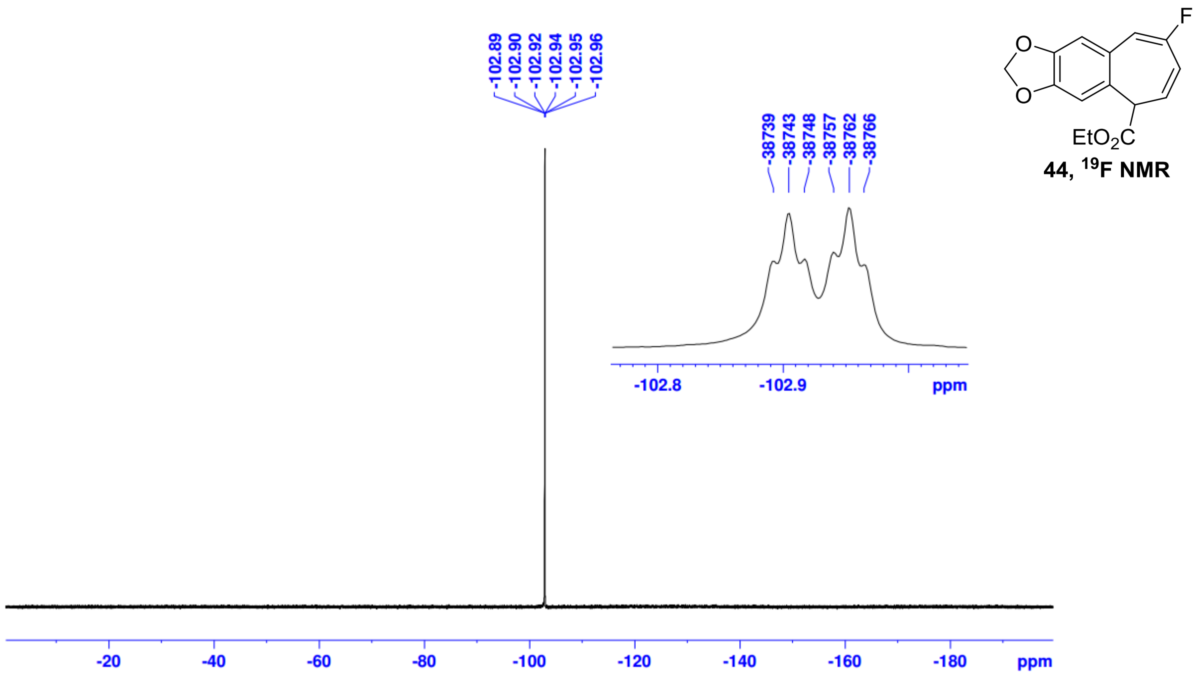

-102.89
-102.90
-102.92
-102.94
-102.95
-102.96
-38739
-38743
-38748
-38757
-38762
-38766
-102.8
-102.9
ppm
-20
-40
-60
-80
-100
-120
-140
-160
-180
ppm
F
O
O
EtO2C
44, 19F NMR

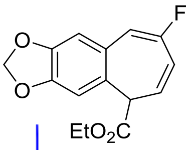


**44, HSQC**

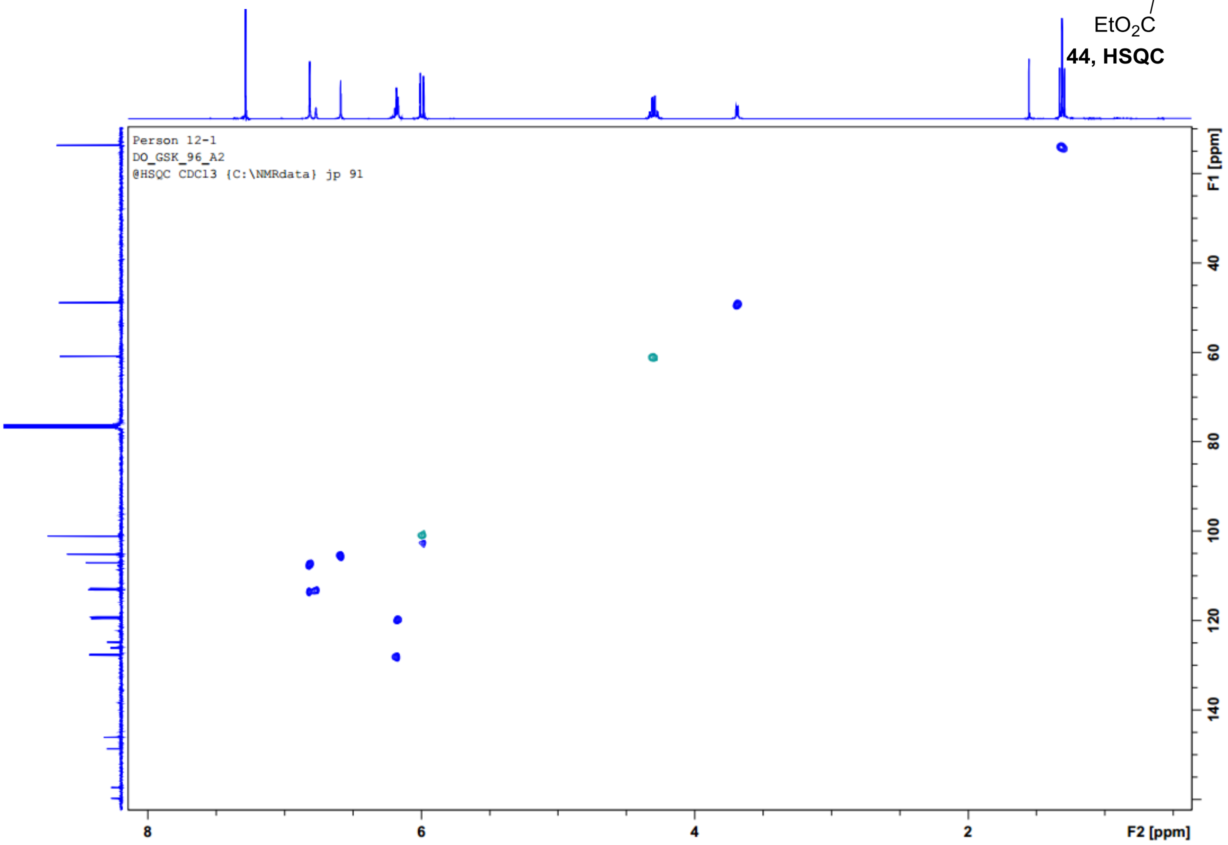


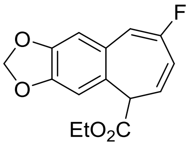


**44, HMBC**

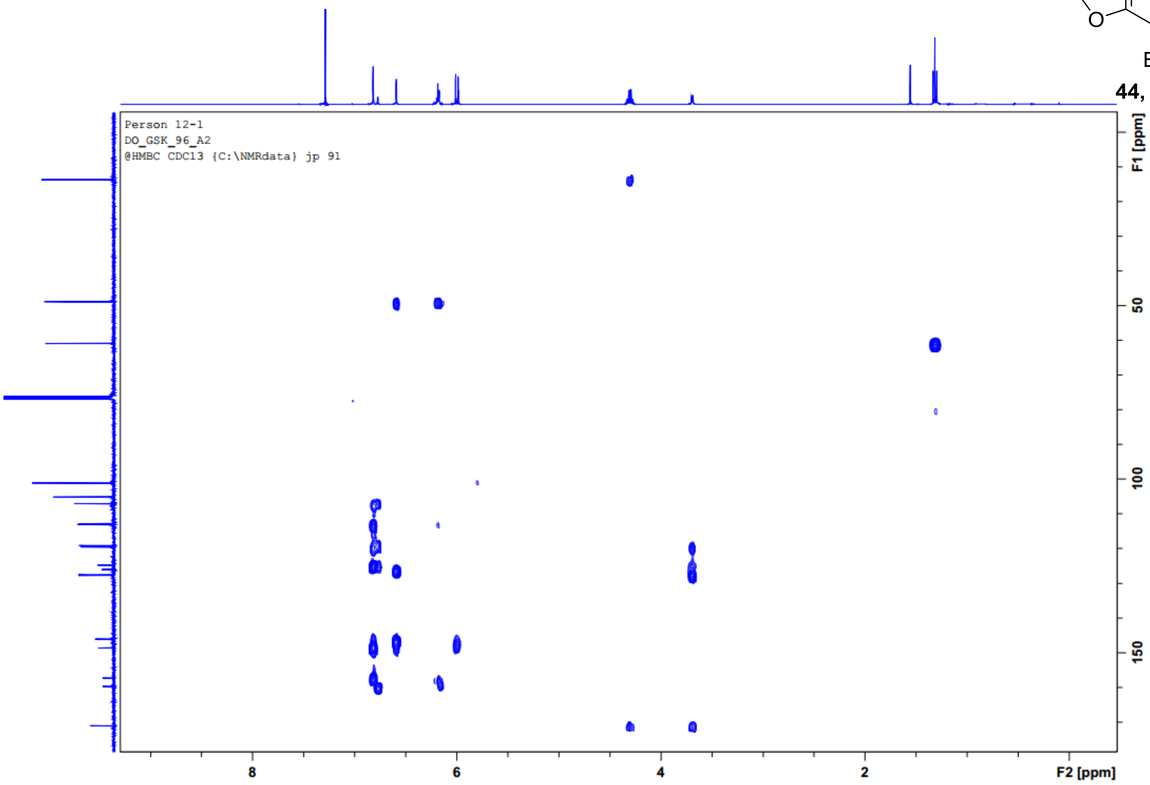

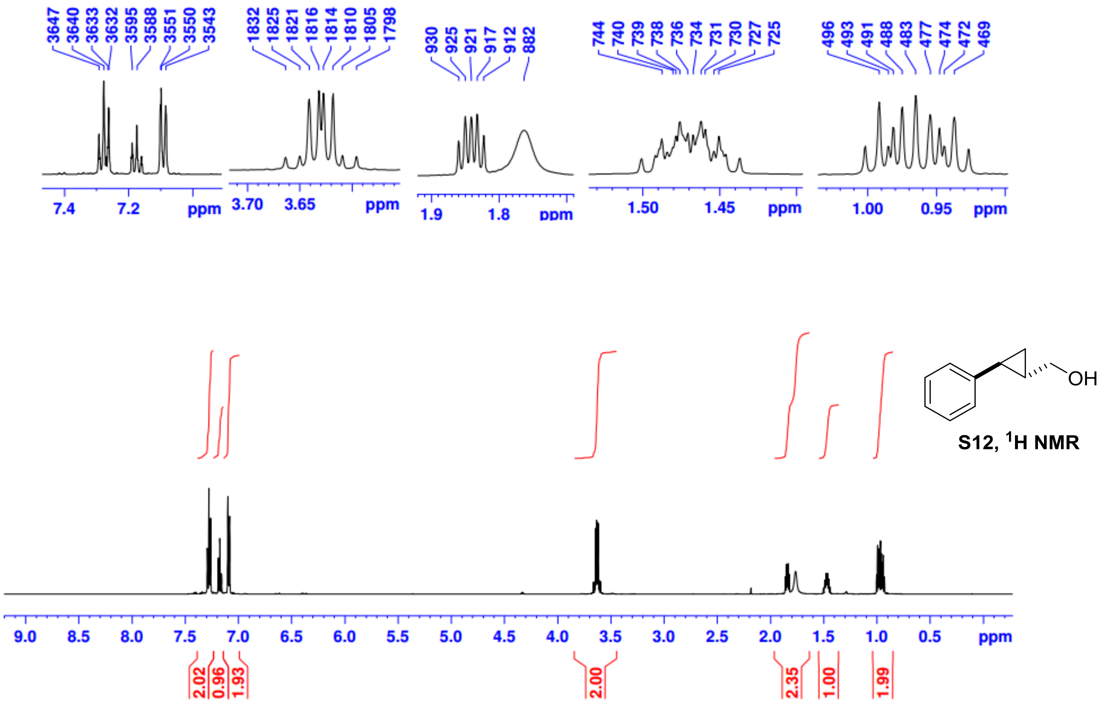

S12, $^{1}$H NMR

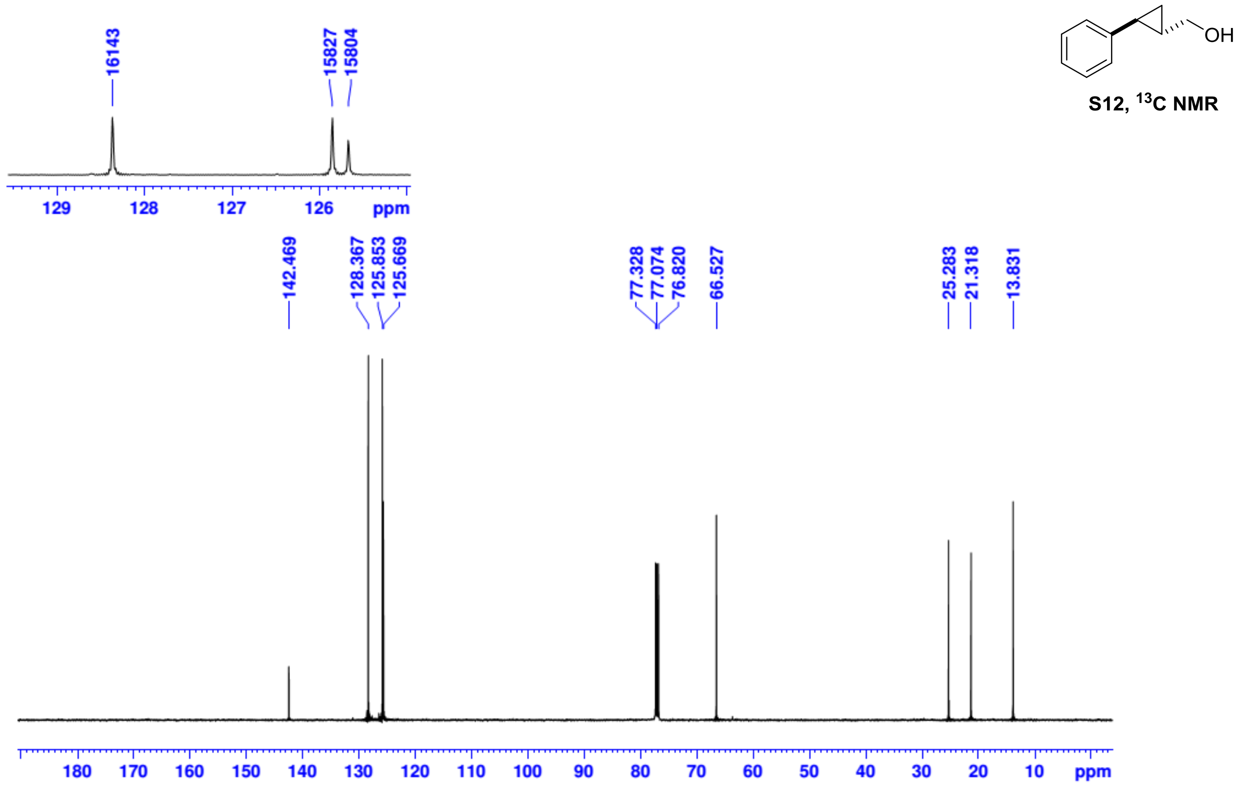

S12, $^{13}$C NMR

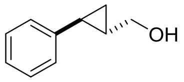


S12, HSQC

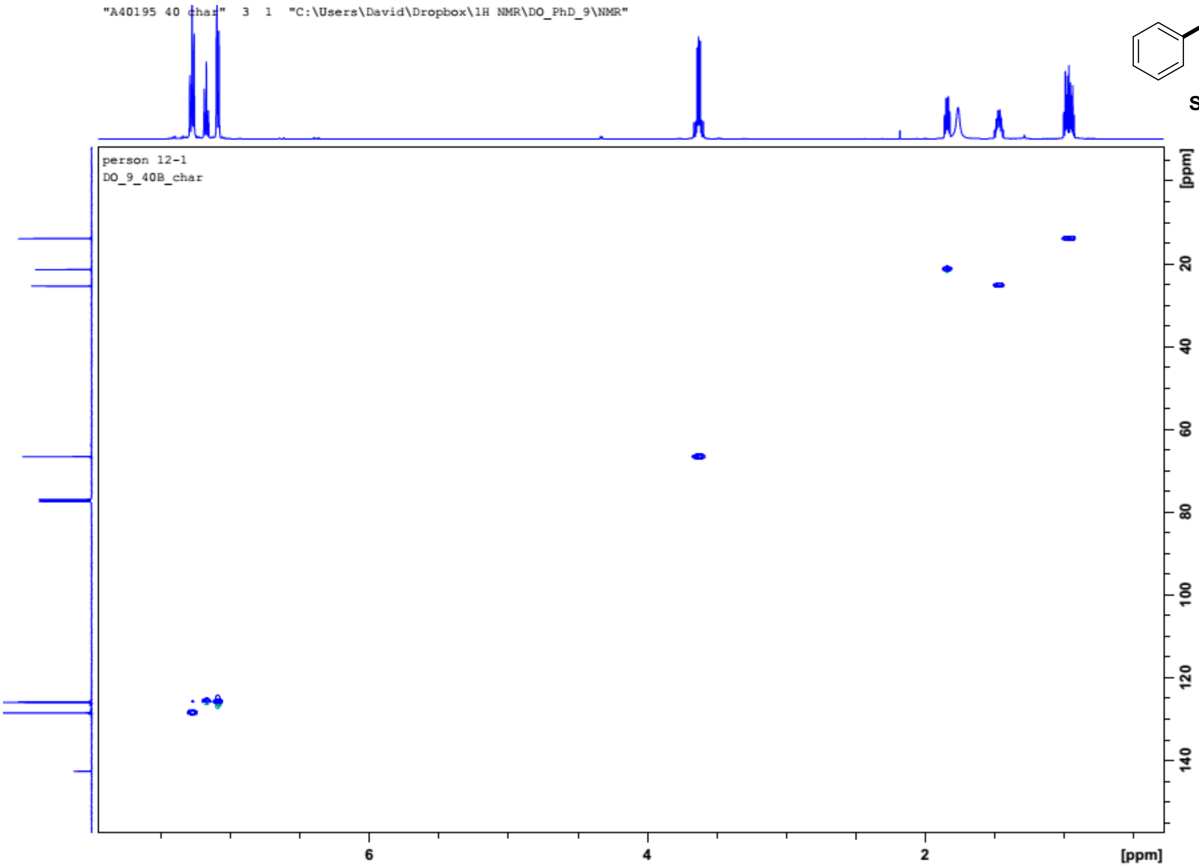


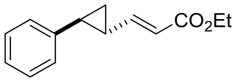


47, $^{1}$H NMR

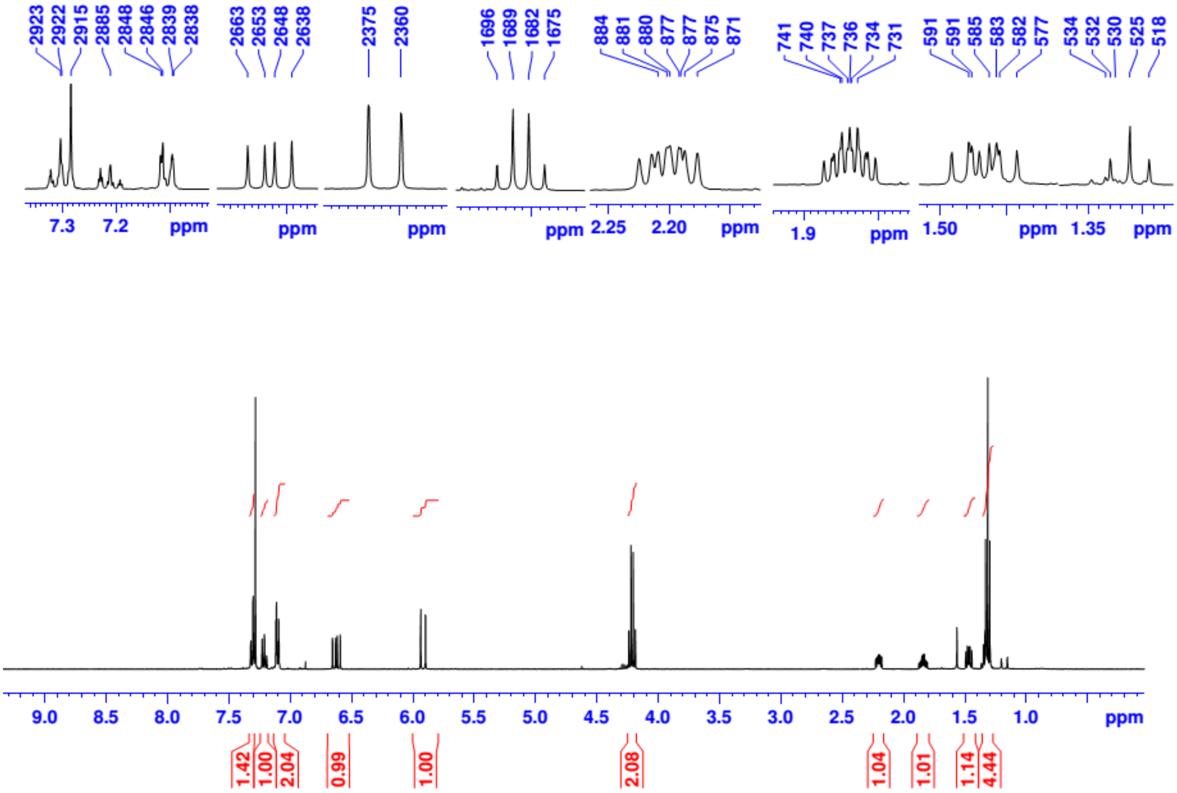

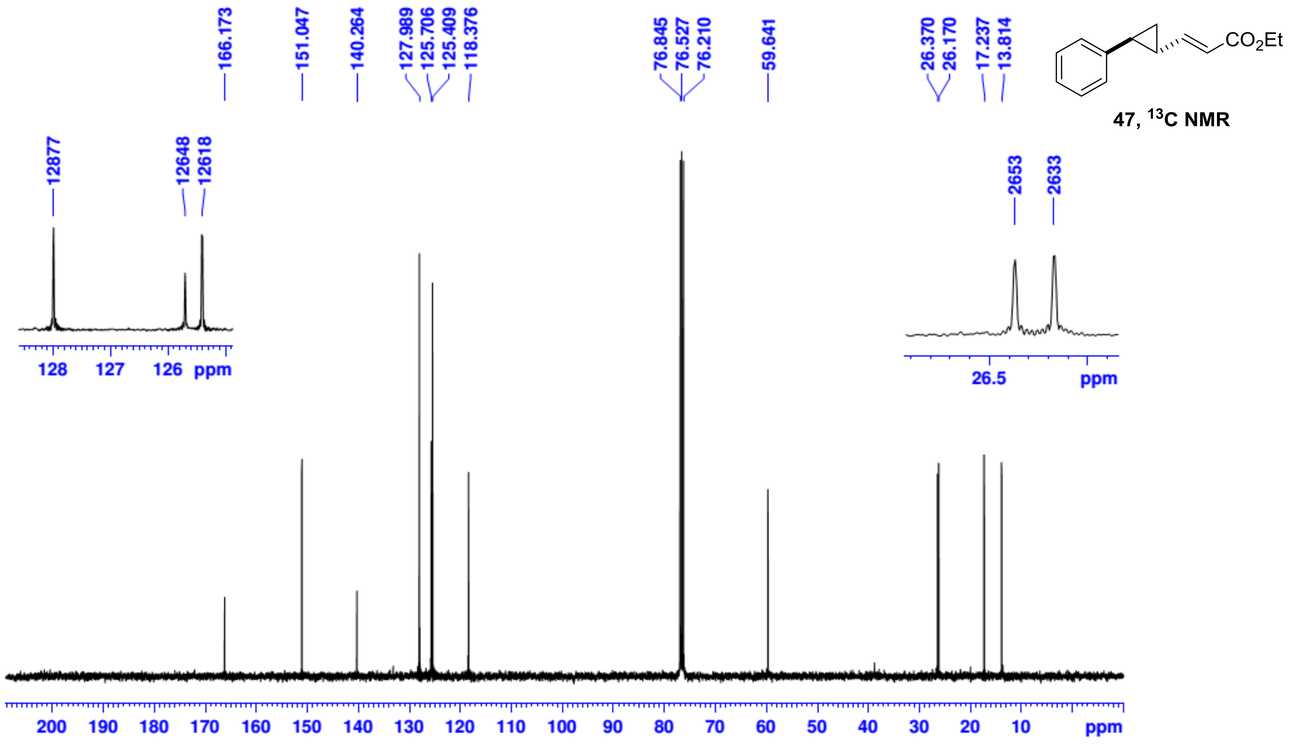


**47, $^{13}$C NMR**

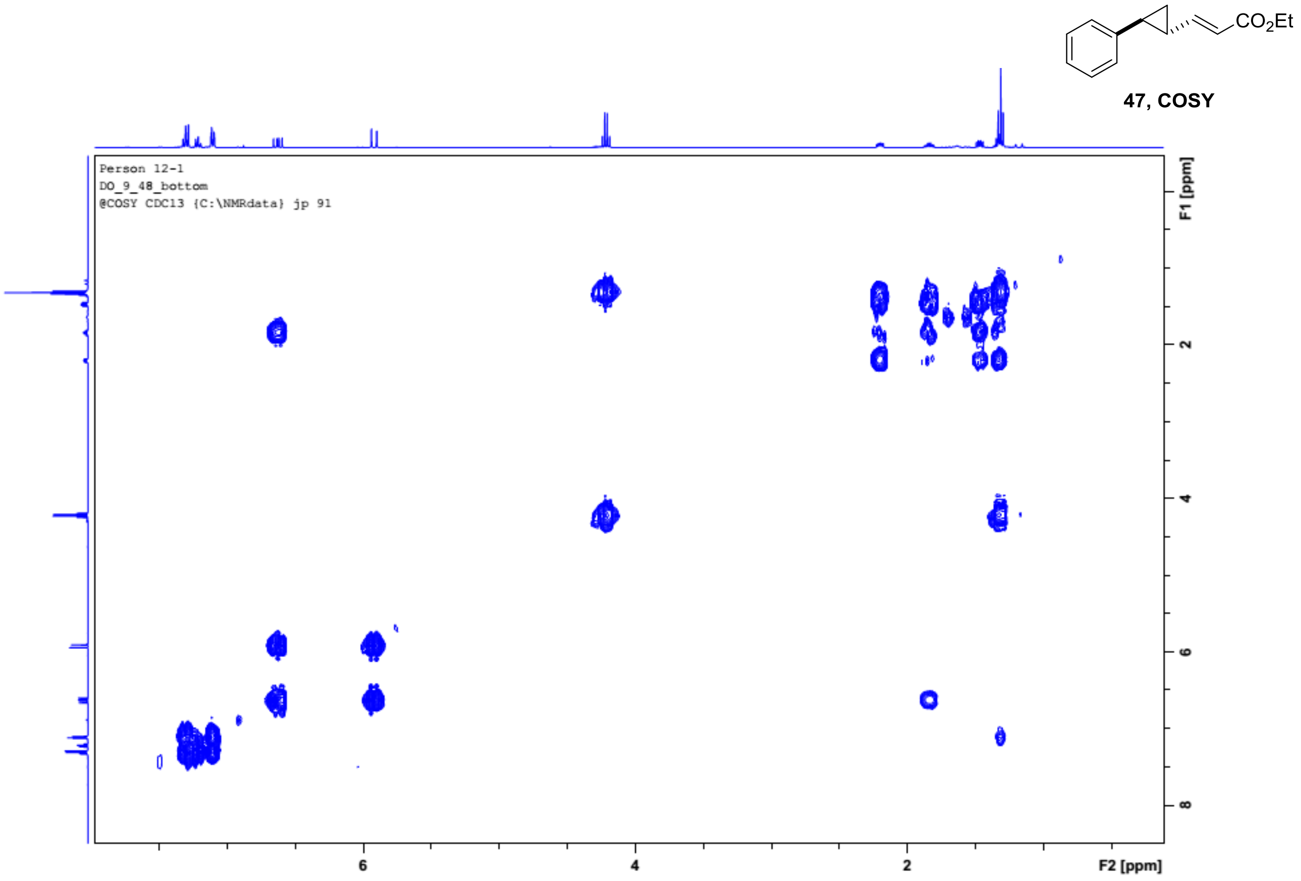


**47, COSY**

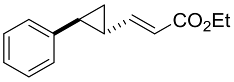


**47, HSQC**

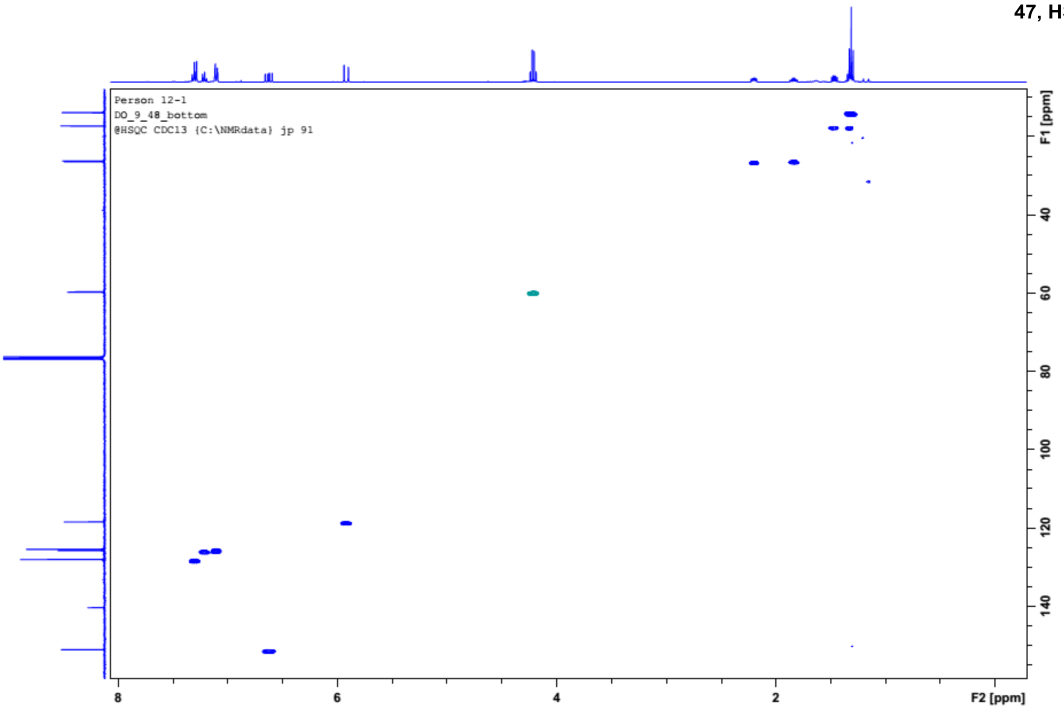


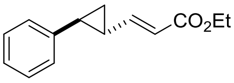


**47, HMBC**

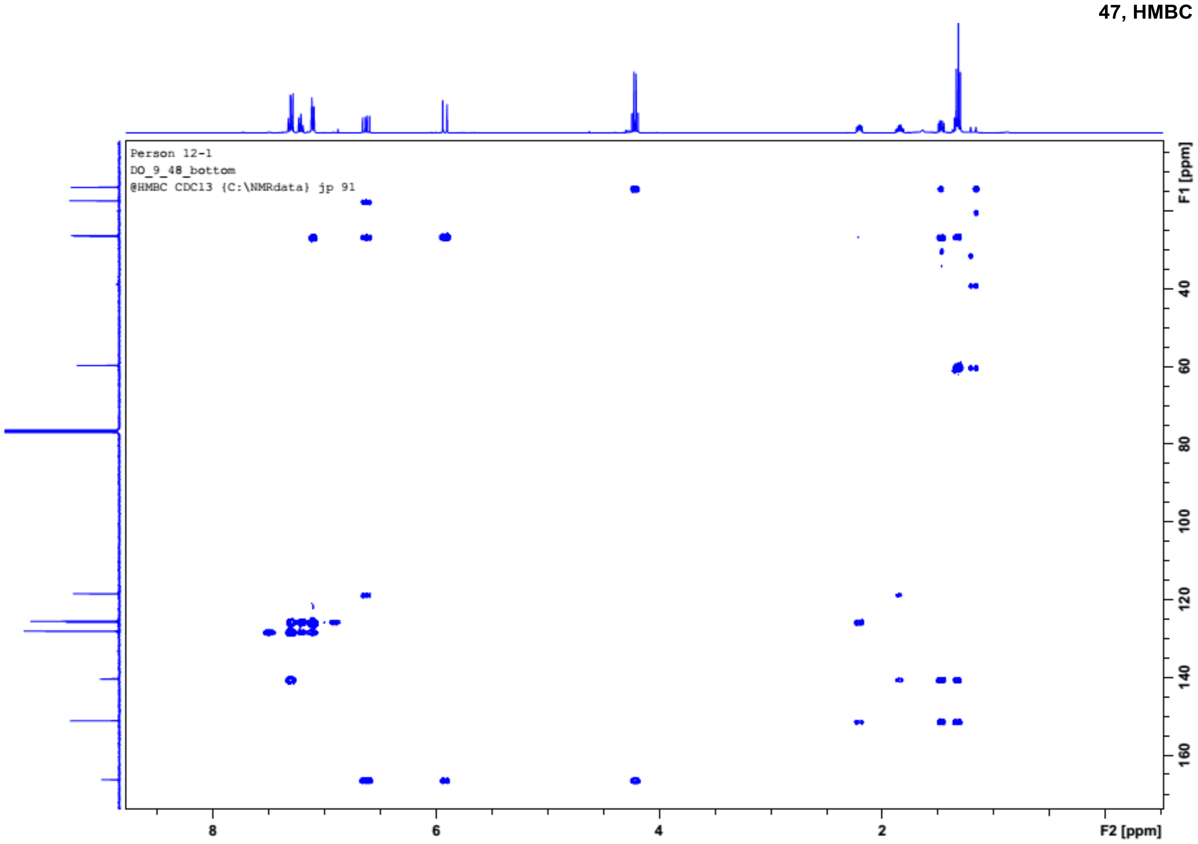

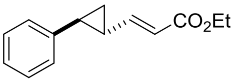


**47, NOESY**

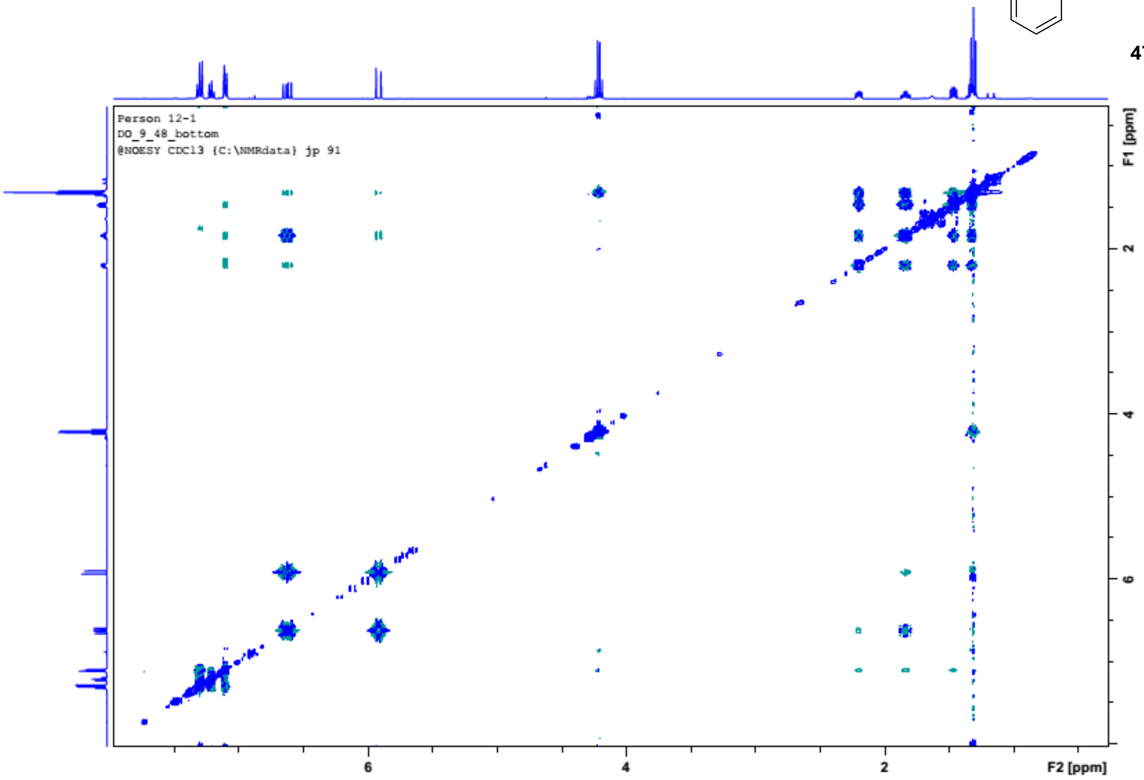


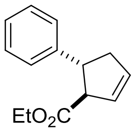


**38a, $^1$H NMR**

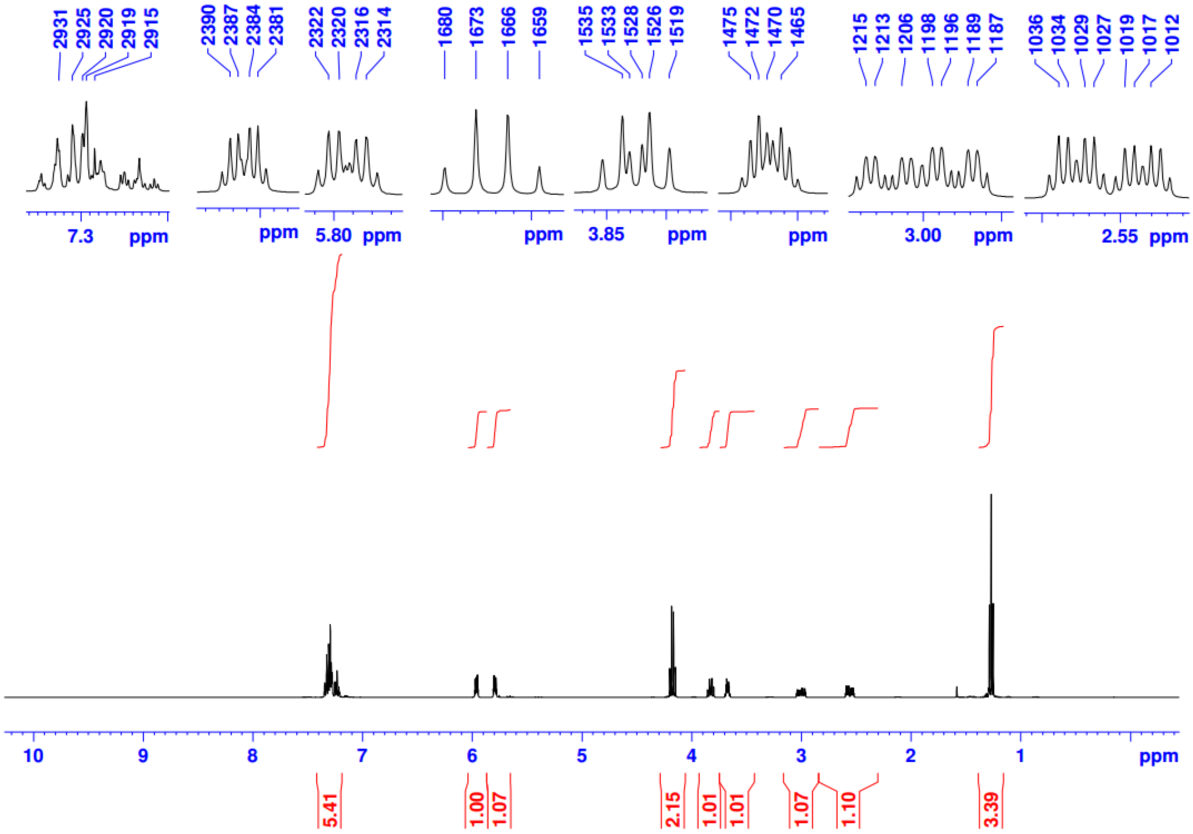

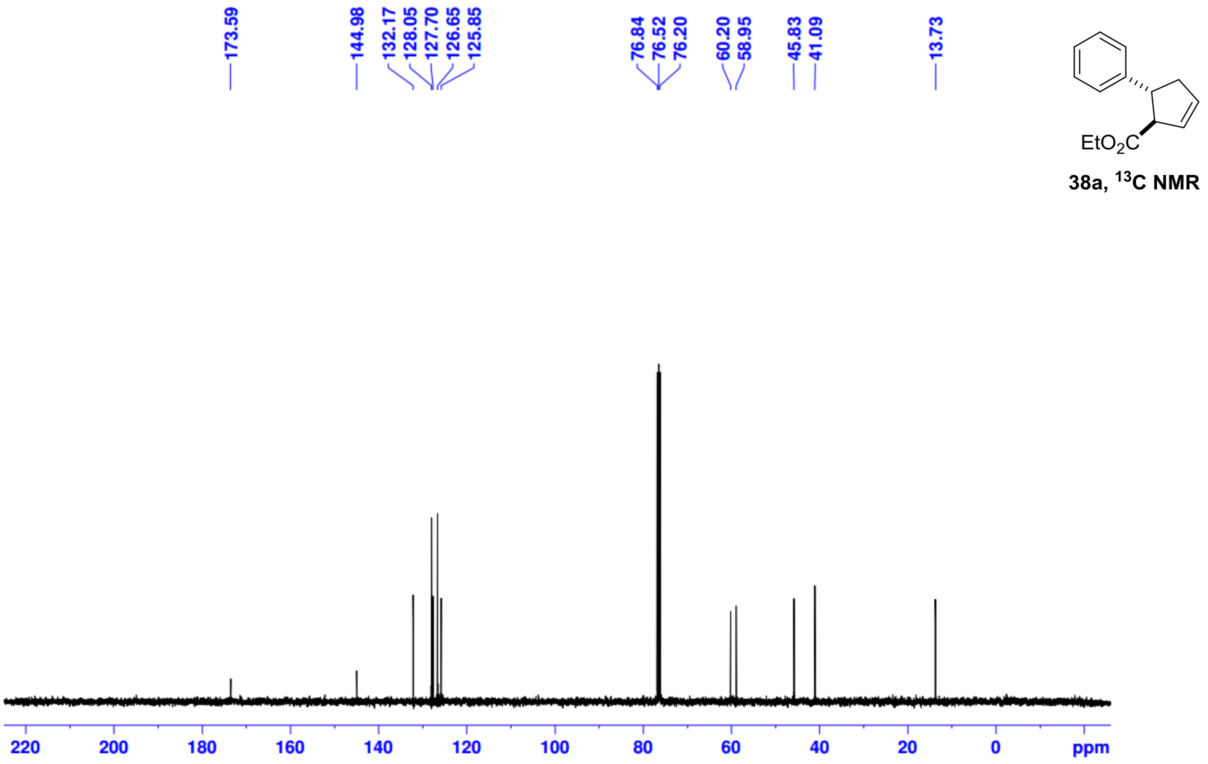


38a, $^{13}$C NMR

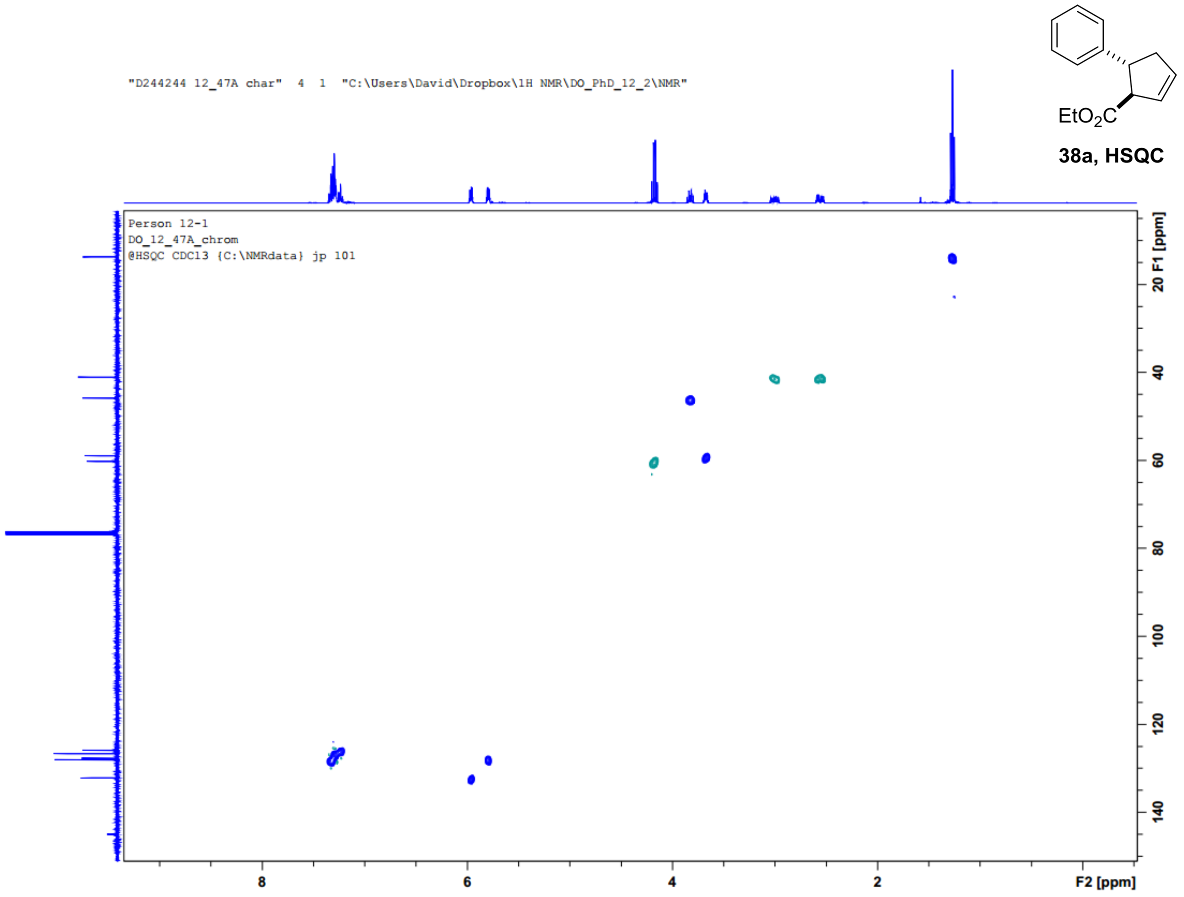


38a, HSQC

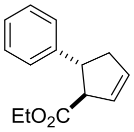


**38a, HMBC**

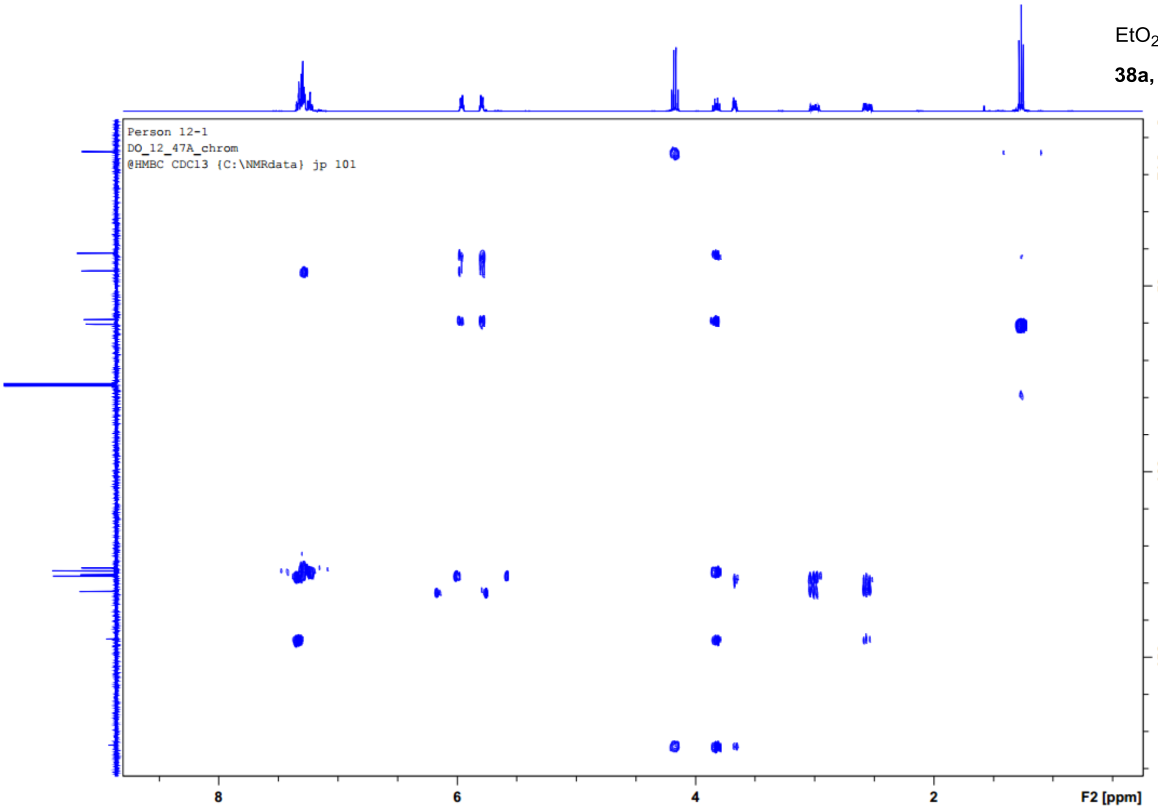


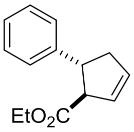


**38a, NOESY**
**no Et-Ar through**
**space interactions**
***trans*-relationship**

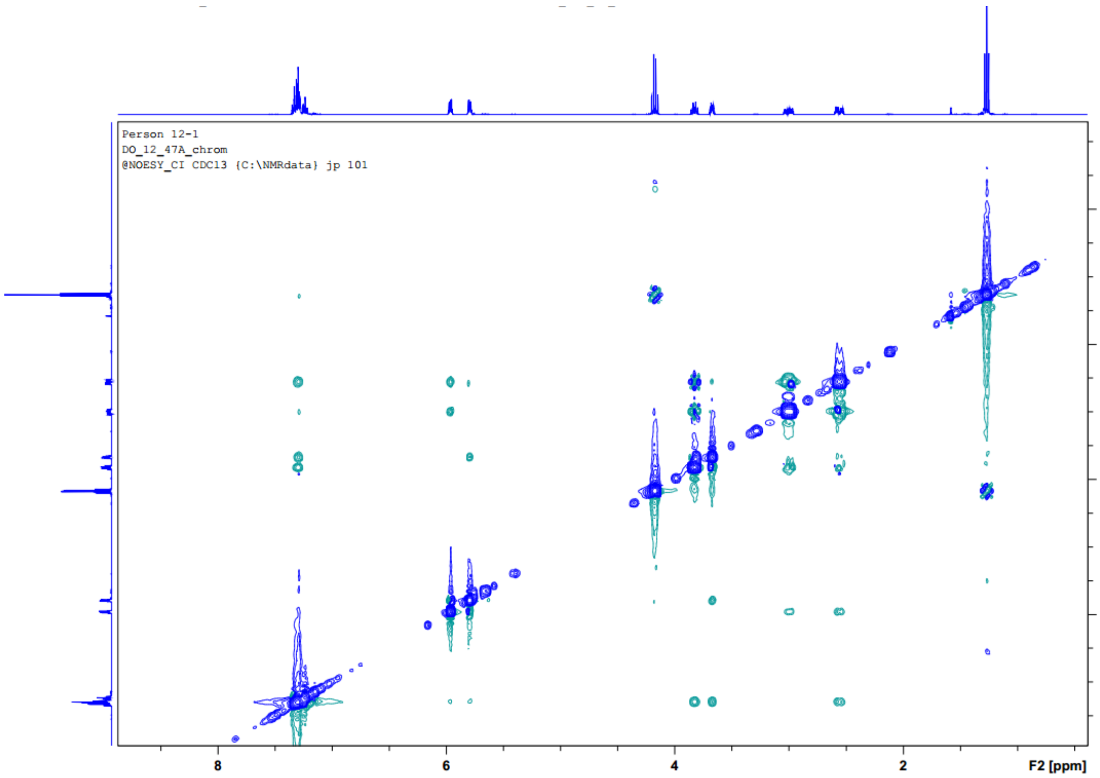

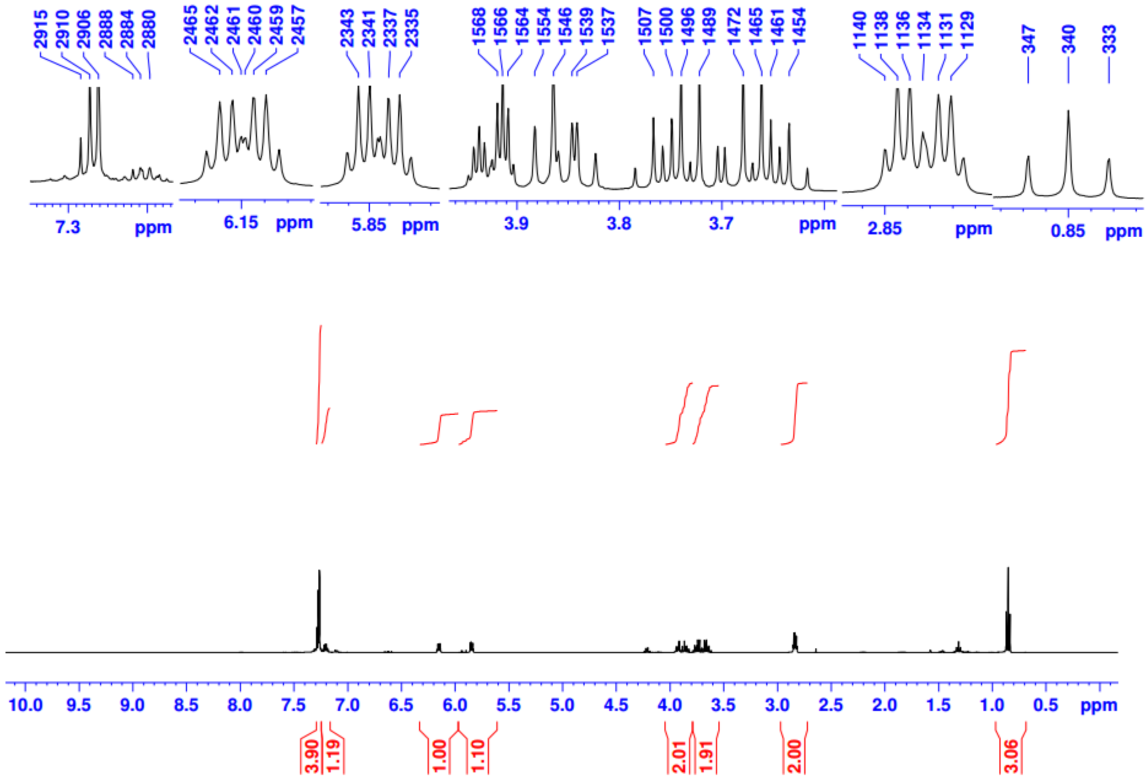


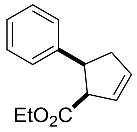


**38b, $^{1}$H NMR**

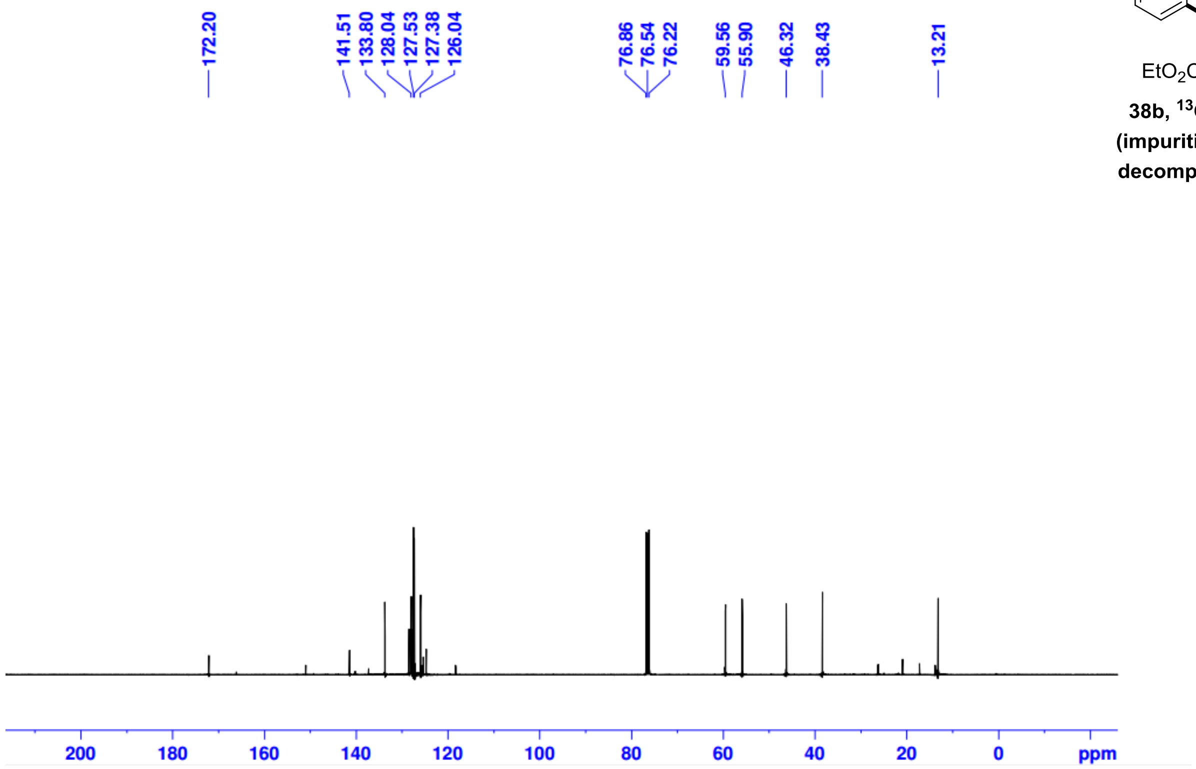


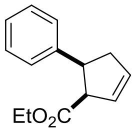


**38b, $^{13}$C NMR (impurities from decomposition)**

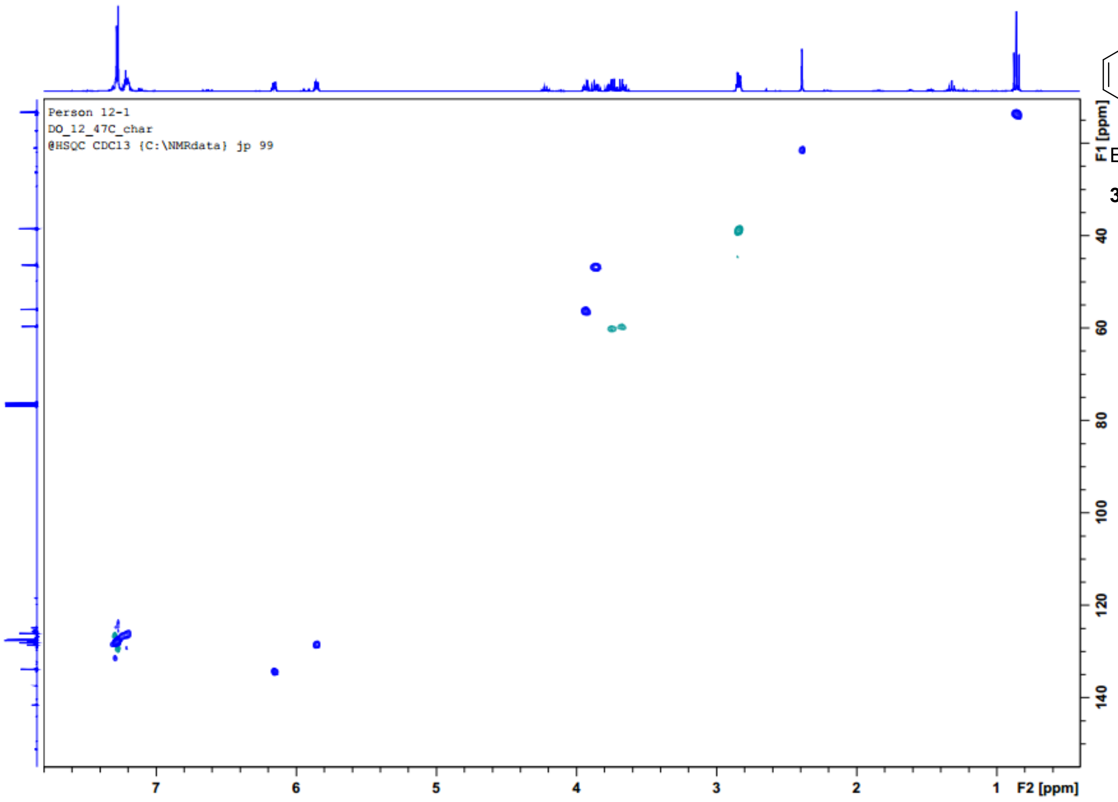


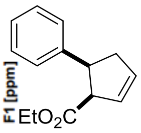


38b, HSQC

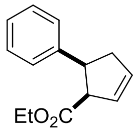


38b, HMBC

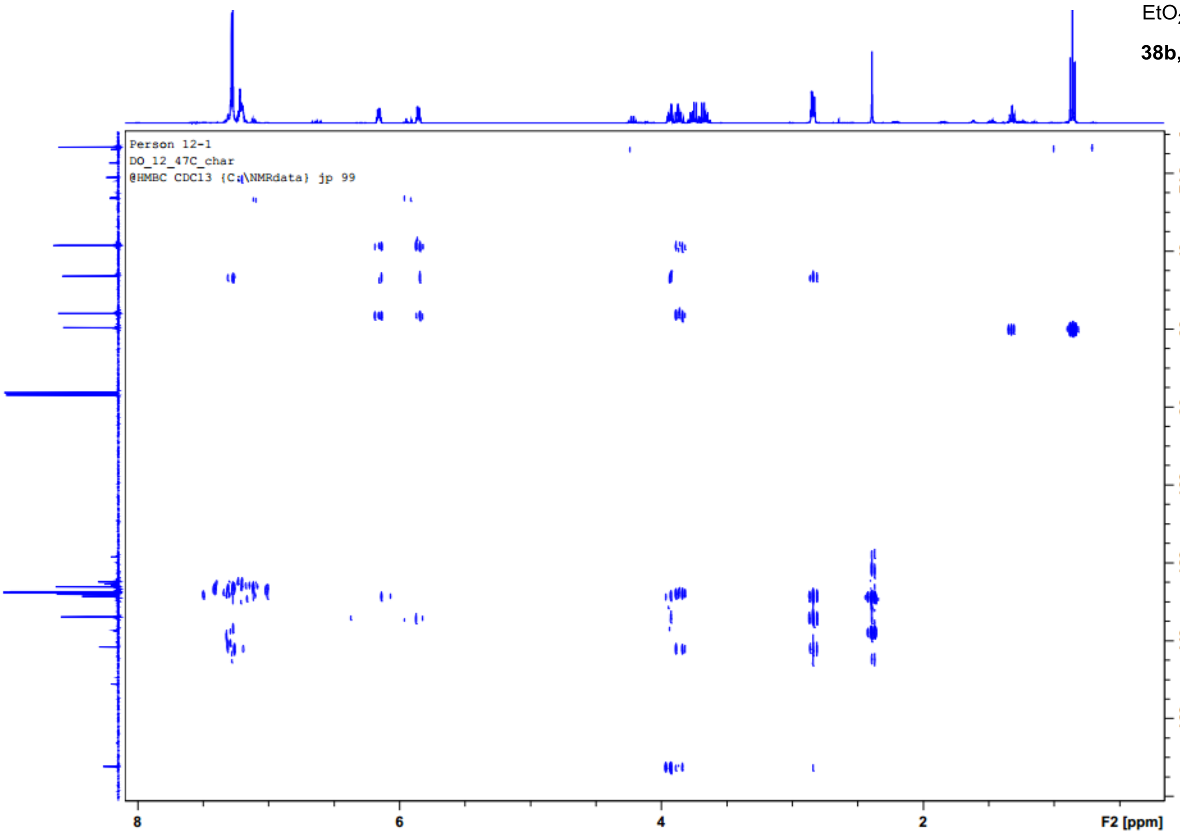

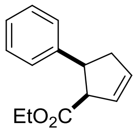


**38b, NOESY**
**observed Et-Ar through**
**space interactions**
***cis*-relationship**

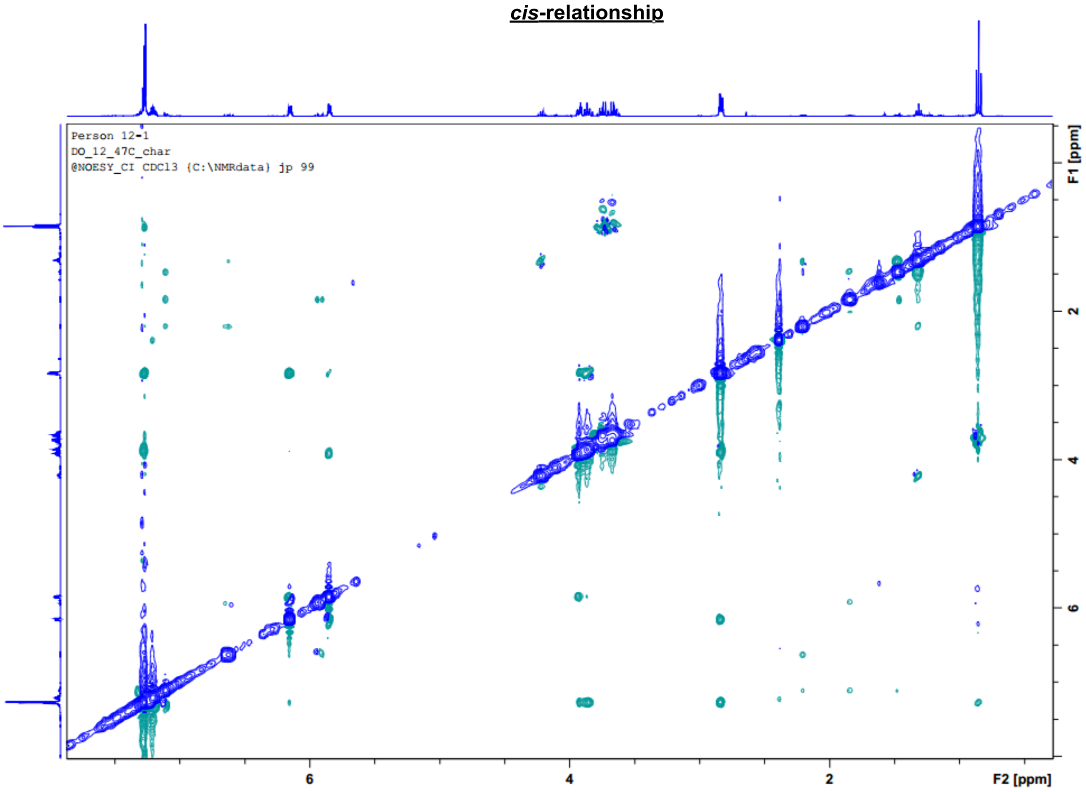

## Decomposition Evidence for Furyl Vinyl Acetate 14a

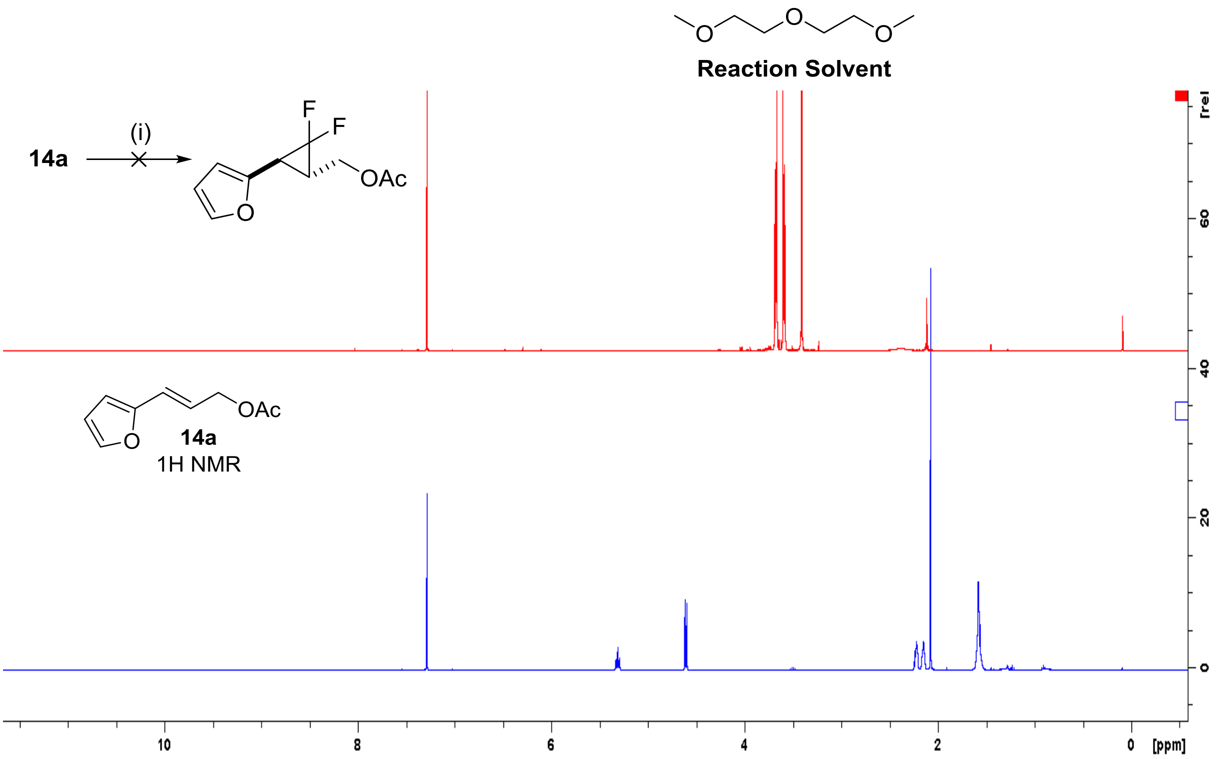


**Figure S4:** Overlay of $^{1}$H NMR spectra representing 2-furyl vinyl acetate **14a** (bottom) and the resulting crude mixture from the failed difluorocyclopropanation. Conditions: (i) MDFA (2.5 eq.), TMSCl (2.5 eq.), KI (2.8 eq.), digylme (1.17 eq.), 120 °C, 24 h.

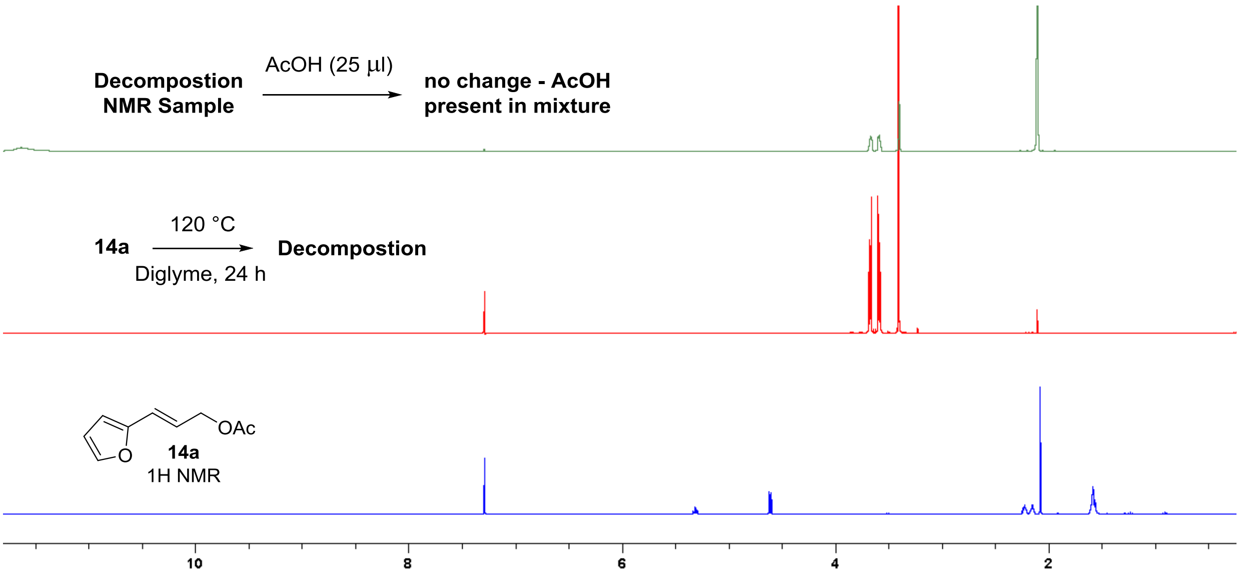


**Figure S5:** Overlay of $^{1}$H NMR spectra representing 2-furyl vinyl acetate **14a** (bottom), resulting mixture after heating **14a** at 120 °C for 24 hours (middle) and spiking the middle NMR sample with 25 μl of AcOH which showed no change suggesting that the AcOH was the only visible side product from the decomposition experiment (top).

**Difluorocyclopropanation of Ethyl Cinnamate**

Both synthesised and commercial ethyl cinnamate **13b** gave similarly low conversion after difluorocyclopropanation (28% and 29%, respectively) so we were confident that there were no side products from the previous olefination step which were responsible for the poor conversion (**Table 3**, Entries 2-3). Again, slightly higher conversions could be obtained by decreasing the reaction time to 4 hours (**Table 3**, Entry 4, 37% conversion) but **15b** could not be separated from **13b** using either chromatography or distillation. Under the same conditions, cinnamyl acetate (**14b**) had given 100% conversion to the desired difluorocyclopropane.[S20] The dramatic reactivity difference between **14b** and **13b** can be attributed to increased electron-deficiency of the alkene in **13b**. Selectivity experiments carried out by Dolbier and co-workers showed that difluorocarbene addition to a 1:1 mixture of butyl acrylate and 1-octene favoured the latter at 120 °C (product ratio of 1:3.3), consistent with the lower reactivity observed for **13b**.[S22] Other literature methods[S23,24] using commercial reagents were previously unsuccessful when used for the difluorocyclopropanation of cinnamyl acetate (**14b**)[S20] and were therefore not investigated for these less reactive alkenoates.

Dolbier and co-workers reported more success in the synthesis of **15b** using alternative MDFA conditions (64%, $^{19}$F NMR conversion);[S25] we were able to replicate their results (69% $^{1}$H NMR conversion) but felt that the longer reaction time of 48 hours would be detrimental to the product recovery of the more reactive alkenoates. Higher conversion of 81% was achieved for this substrate by the same group using trimethylsilyl 2,2-difluoro-2-(fluorosulfonyl)acetate (TFDA) as the difluorocarbene precursor but it is unclear if this is an isolated or NMR yield.[S22] This reagent is less practical than others since it is prone to hydrolysis; purification is necessary before every reaction. We observed similar conversion with TFDA (79%, determined by $^{1}$H NMR) but the isolation of synthetically useful quantities of **15b** proved difficult due to the presence of starting alkenoate **13b** and the previously unreported difluoromethyl ester side product **S13** (**Scheme S4a**, see below for a full discussion on difluorocyclopropanation of **13b** with TFDA). We currently do not have a reason for the formation of **S13** but it is proposed to proceed via a mechanism similar to that of the difluoromethylation of acids reported by Wu and Chen[S26] and driven by the elimination of ethene (**Scheme S4a**).

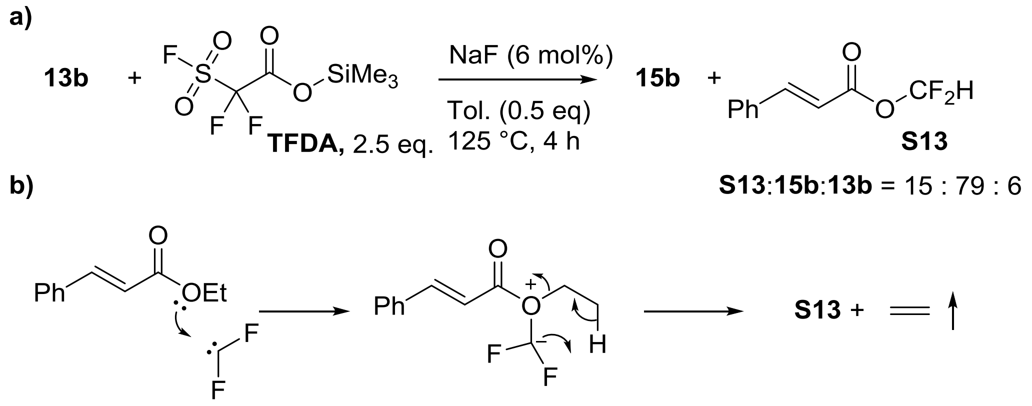

**Scheme 5:** a) Difluorocyclopropanation of **13b** using TFDA (ratios determined by $^1$H NMR). b) Proposed mechanism for the formation of side product **S13**.

Reported difluorocyclopropanation methodologies are generally screened against a set of simple alkene substrates but reactivity issues are rarely discussed. We have used alkenoates **13a** and **13b** to assess the most reactive procedures in the literature and highlighted the synthetic limitations for electron deficient olefins. For this study, the main purpose of the synthetic investigations was to obtain a range of substituted cyclopropane precursors in order to test computational predictions. No further screening of conditions was carried out, but recent synthetic advances with metal-mediated difluorocyclopropanation reported by Ichikawa and co-worker, look to be a more promising method for the synthesis of difluorocyclopropyl esters **15b**.[S27]

**Preparation of trimethylsilyl 2,2-difluoro-2-(fluorosulfonyl)acetate (S14)**

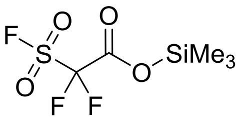

**S14**

A mixture of chlorotrimethylsilane (4.9 mL, 38.8 mmol) and commercial trimethylsilyl 2,2-difluoro-2-(fluorosulfonyl)acetate (TFDA, **S14**, 1 mL, 9.7 mmol, 80% purity determined by $^{19}$F NMR) was refluxed (110 °C) for 1 hour under an atmosphere of argon. The reaction mixture was cooled to room temperature and the water condenser was replaced with an oven dried distillation apparatus and the glassware cooled under an atmosphere of argon. Excess TMSCl was removed (90 °C, 600 mbar) and the residual **S14** was stored under argon at room temperature. The $^{19}$F NMR spectrum of the residue confirmed reagent purity at > 95% and **S14** used immediately for a difluorocyclopropanation reaction. $^{19}$F NMR (376 MHz,

CDCl$_3$): δ = -41.2 (dd, *J* = 4.6, 4.2 Hz, *F*SO$_2$CF$_2$, 1F), -103.1 ppm (d, *J* = 4.4 Hz, FSO$_2$C*F*$_2$, 2F). The data was in agreement with that reported by Tian and co-workers.[S28]

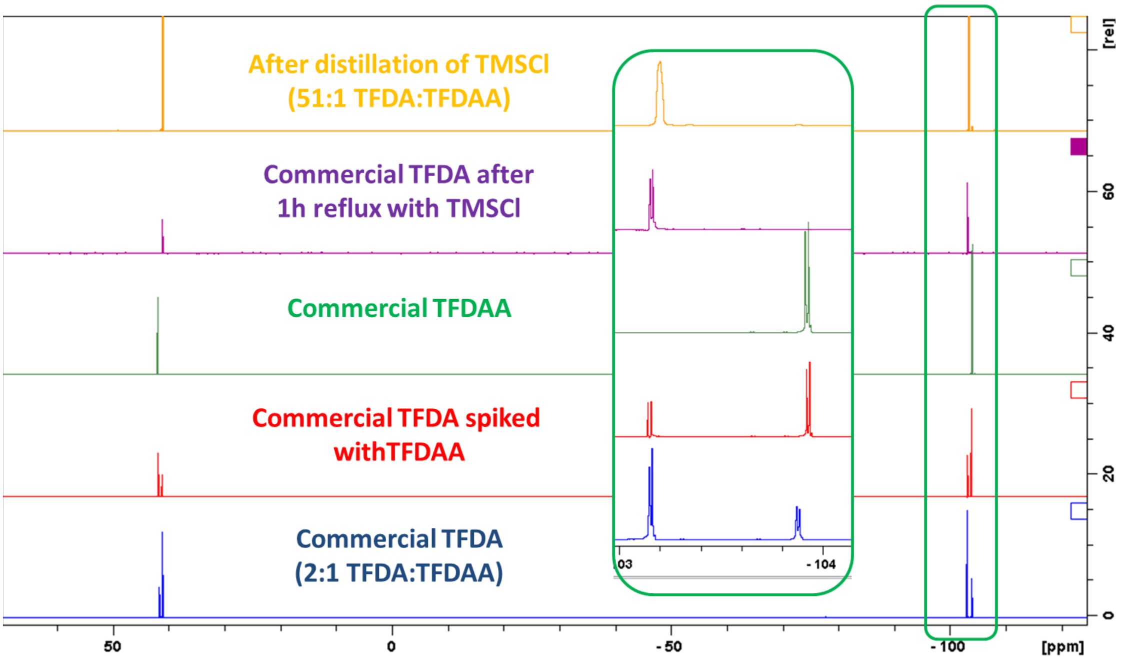


**Figure S6**: $^{19}$F NMR analysis of the synthesis and purification of TFDA.

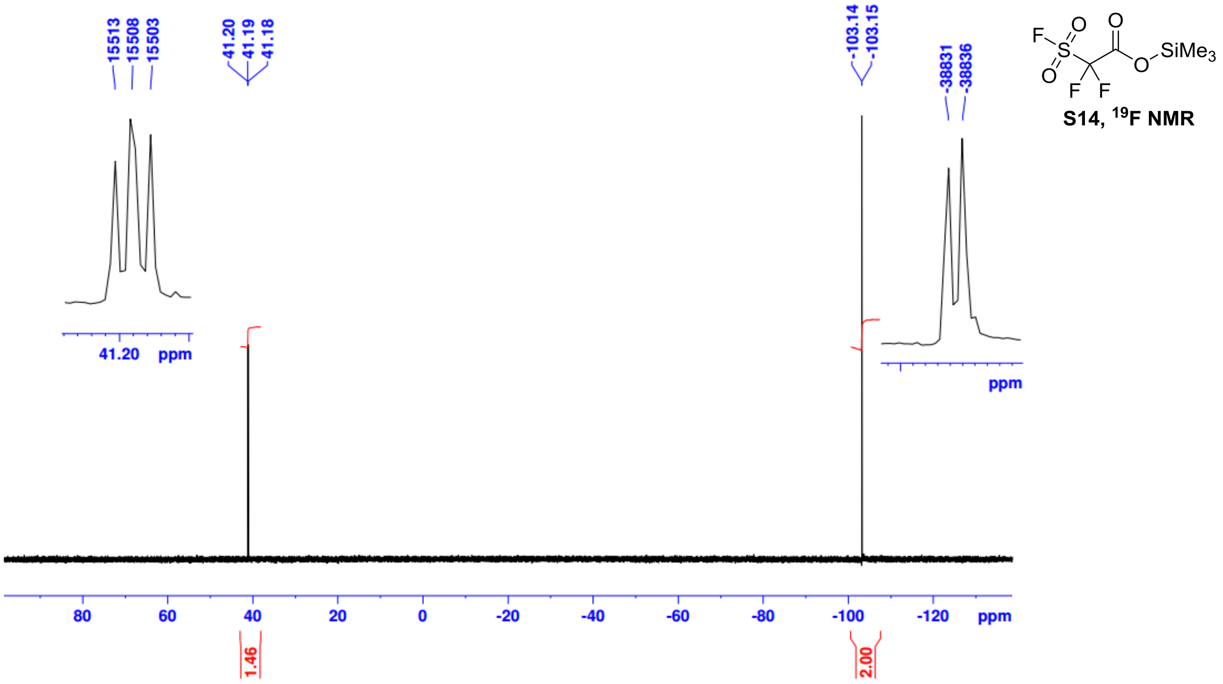


**Figure S7**: $^{19}$F NMR for clean TFDA used in difluorocyclopropanation reaction.

**Attempted difluorocyclopropanation of ethyl cinnamate with TFDA**

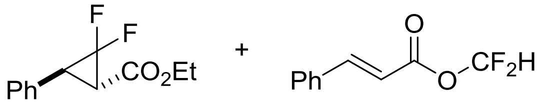


**15b** **S13**

An oven dried two-necked round bottom flask containing sodium fluoride (7 mg, 0.17 mmol) was sealed with a SubaSeal, and the salt was stirred and lightly flame dried under an atmosphere of argon. A low boiling point water condenser with a gas outlet connected to an argon/vacuum manifold was attached to the reaction flask and the atmosphere was purged three times. Ethyl cinnamate (0.48 mL, 2.84 mmol) followed by toluene (0.15 mL, 1.42 mmol) were added and the colourless suspension was heated to 125 °C. Once the reaction temperature had been reached, freshly distilled TFDA (1.4 mL, 7.1 mmol) was added dropwise over a period of 3 hours using a syringe pump. The mixture was stirred for a further 1 hour (total reaction time of 4 hours). The reaction mixture was cooled to room temperature and the reaction mixture was quenched with addition of water (10 mL) and diethyl ether (10 mL). The organic layer was separated and the aqueous layer was extracted with diethyl ether (4 x 10 mL). The original organic layer and the extracts were combined, dried ($MgSO_4$) and concentrated under reduced pressure to remove volatile materials. The $^{1}H$ NMR spectrum of the crude reaction mixture was used to determine the ratio of **13b**, **15b** and **S13**.

A similar crude reaction mixture was purified by column chromatography on silica gel (1:19 to 1:9 $Et_2O$ in hexane) and a mixture of **S13** and **13b** (4:1 ratio determined by NMR) was isolated.

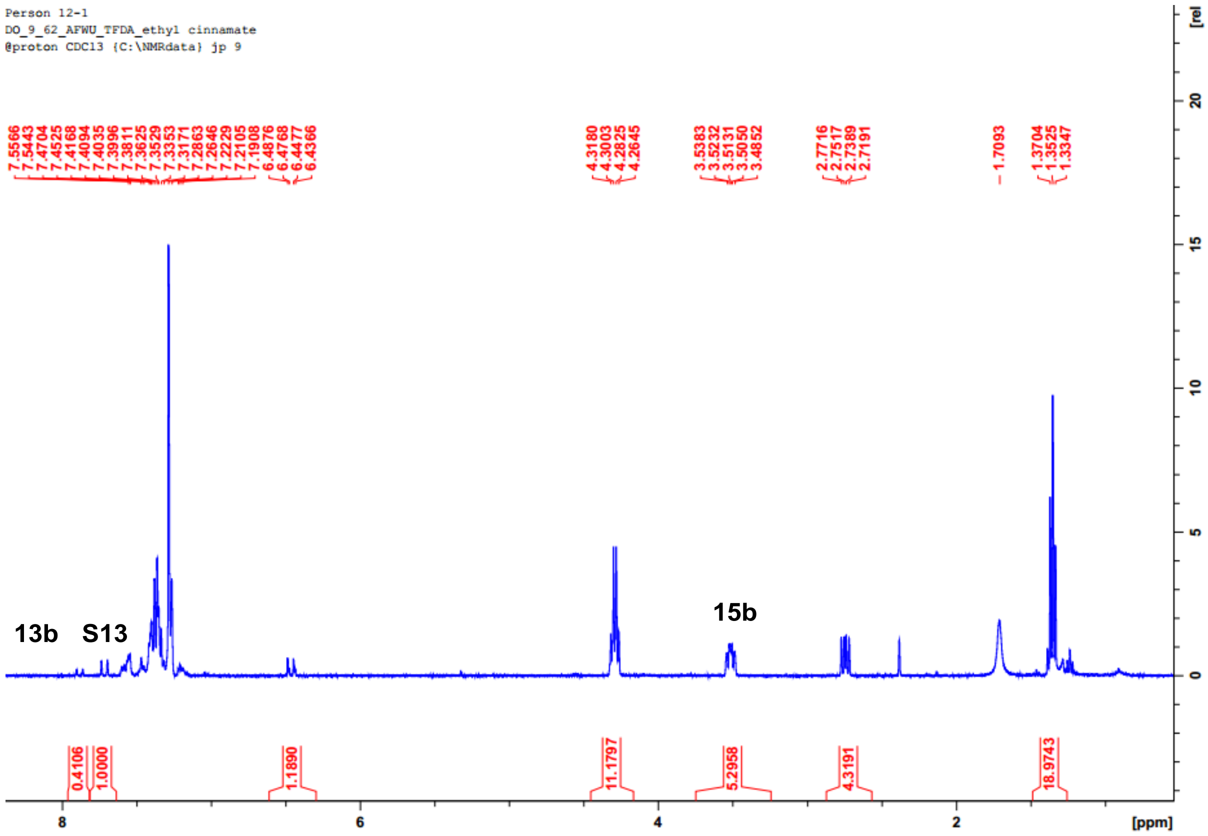


**Figure S8:** Crude $^{1}H$ NMR used to relative conversion ratio from TFDA difluorocyclopropanation.

Extracted data for **S13**: $R_f$ = 0.26 (1:4 $Et_2O$ in hexane); $^{1}H$ NMR (400 MHz, $CDCl_3$): δ = 7.88 (d, *J* = 16.0 Hz, ArC*H*=CH, 1H), 7.60-7.58 (m, Ar*H*, 3H), 7.49-7.43 (m, Ar*H*, 2H), 7.22 (t, $^{2}J_{H\text{-}F}$ = 71.2 Hz, $CF_2H$, 1H), 6.56 ppm (d, *J* = 16.0 Hz, CH=C*H*CO, 1H); $^{13}C$ NMR (100 MHz, $CDCl_3$): δ = 162.8 (t, $J_{C\text{-}F}$ = 3.4 Hz), 149.2, 133.6, 131.6, 129.2, 128.8, 115.1, 112.8 ppm (t, $^{1}J_{C\text{-}F}$ = 257.1 Hz); $^{19}F$ NMR (376 MHz, $CDCl_3$): δ = -91.4 ppm (d, $^{2}J_{F\text{-}H}$ = 71.2 Hz); $\bar{\nu}$/(film) = 1738, 1634, 1059, 980 $cm^{-1}$; MS (CI): *m/z* (%): 227 (4) $[M+C_2H_5]^+$, 199 (47) $[M+H]^+$, 157 (6), 131 (100) $[M\text{-}OCF_2H]^+$; $t_R$ (GC) = 10.45 minutes.

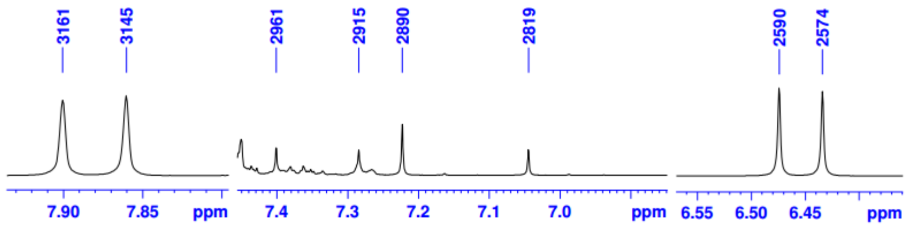


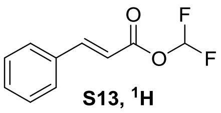


**S13, $^{1}$H**
**(contains 20% 15b)**

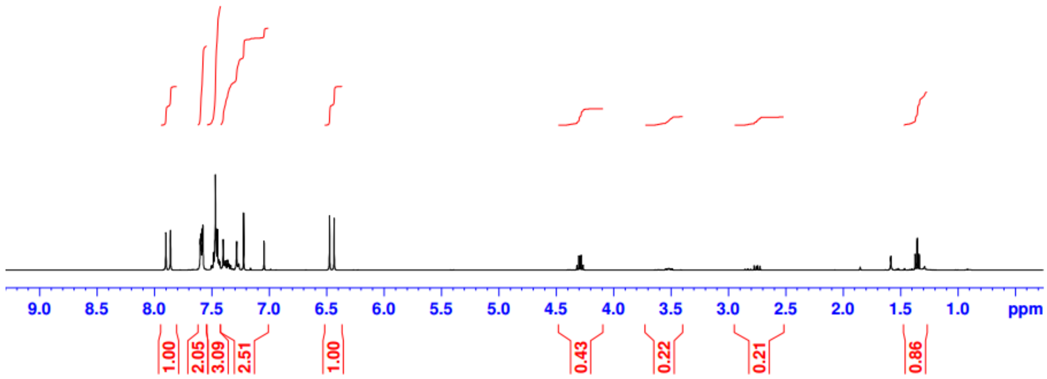


**S13, $^{19}$F**
**(contains 20% 15b)**

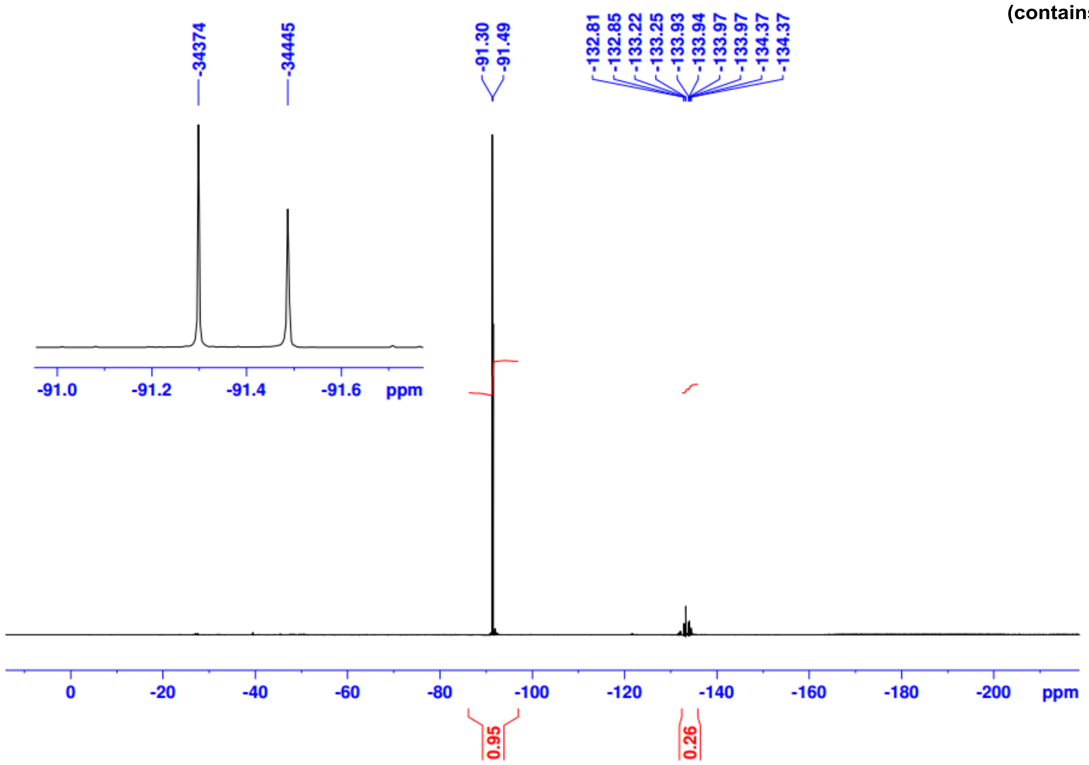

S13, $^{13}C$
(contains 20% 15b)

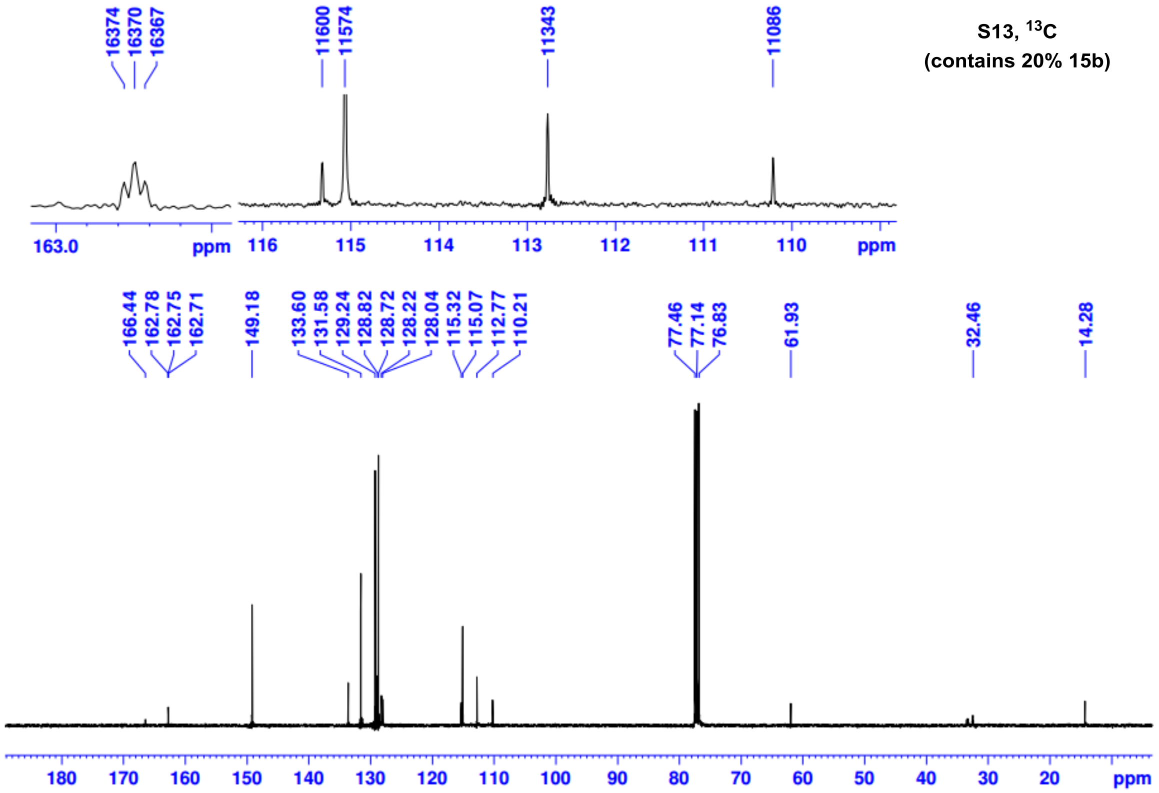


S13, HSQC
(contains 20% 15b)

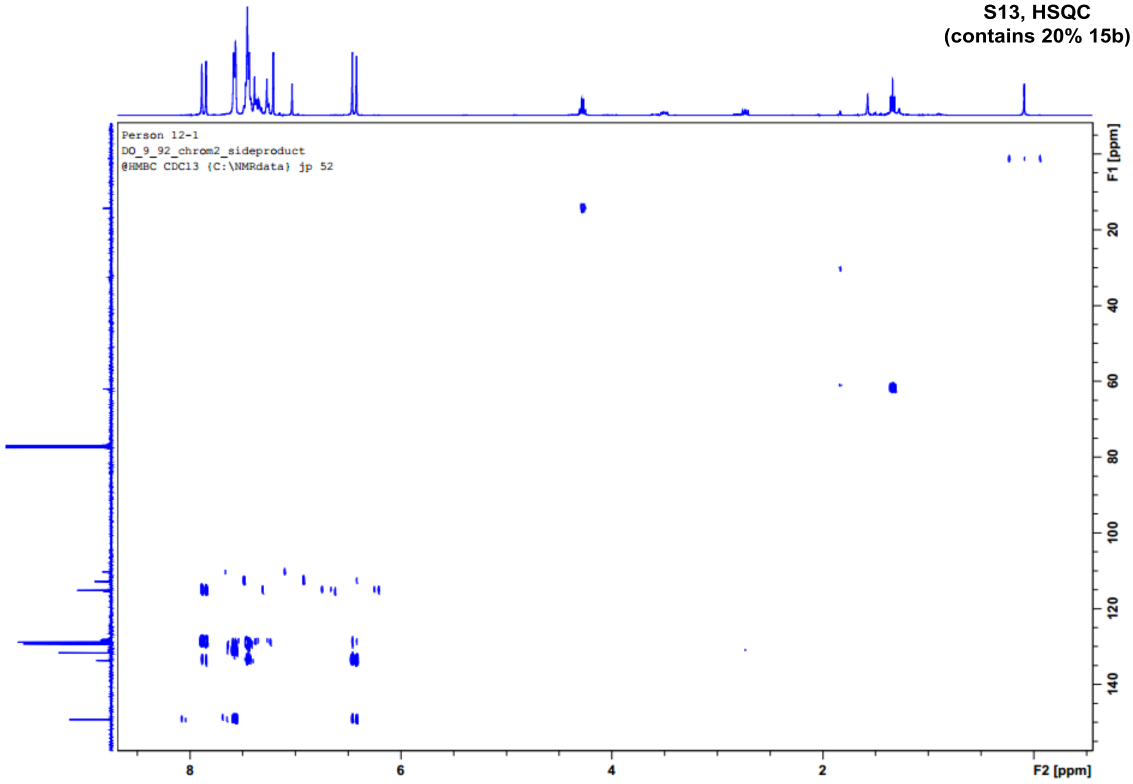

**S13, HMBC**
**(contains 20% 15b)**

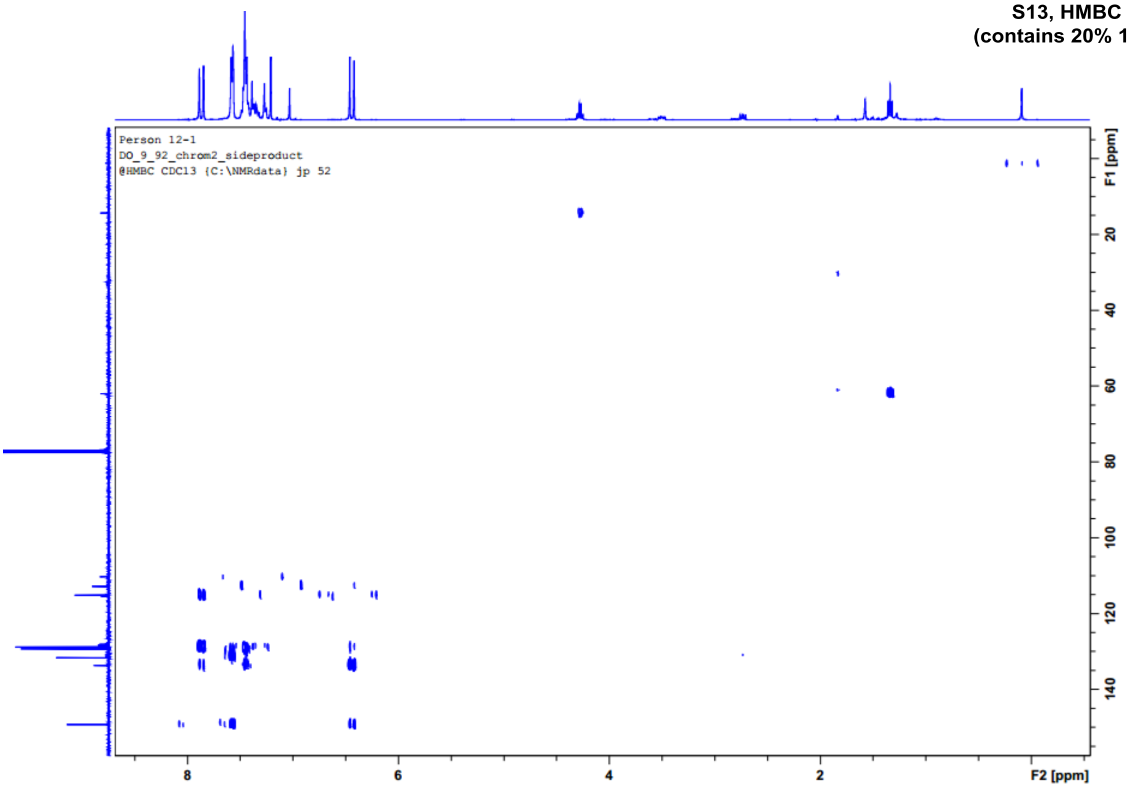

## Evidence of Difluorocarbene-Digylme Reaction

An oven dried two-necked round bottom flask containing sodium chlorodifluoroacetate (275 mg, 1.8 mmol) was sealed with a SubaSeal, and the salt lightly flame dried under vacuum. The atmosphere was replaced with nitrogen and the flask allowed to cool to room temperature. A low boiling point water condenser with a gas outlet connected to an argon/vacuum manifold was attached to the reaction flask and the atmosphere was purged three times. Diglyme (1 mL) was added and a reaction aliquot (< 0.1 mL) was taken and analysed by $^{19}F$ NMR. The saturated mixture was heated to 180 °C and reaction aliquot taken at different time points and analysed by $^{19}F$ NMR (see **Figure S9**).

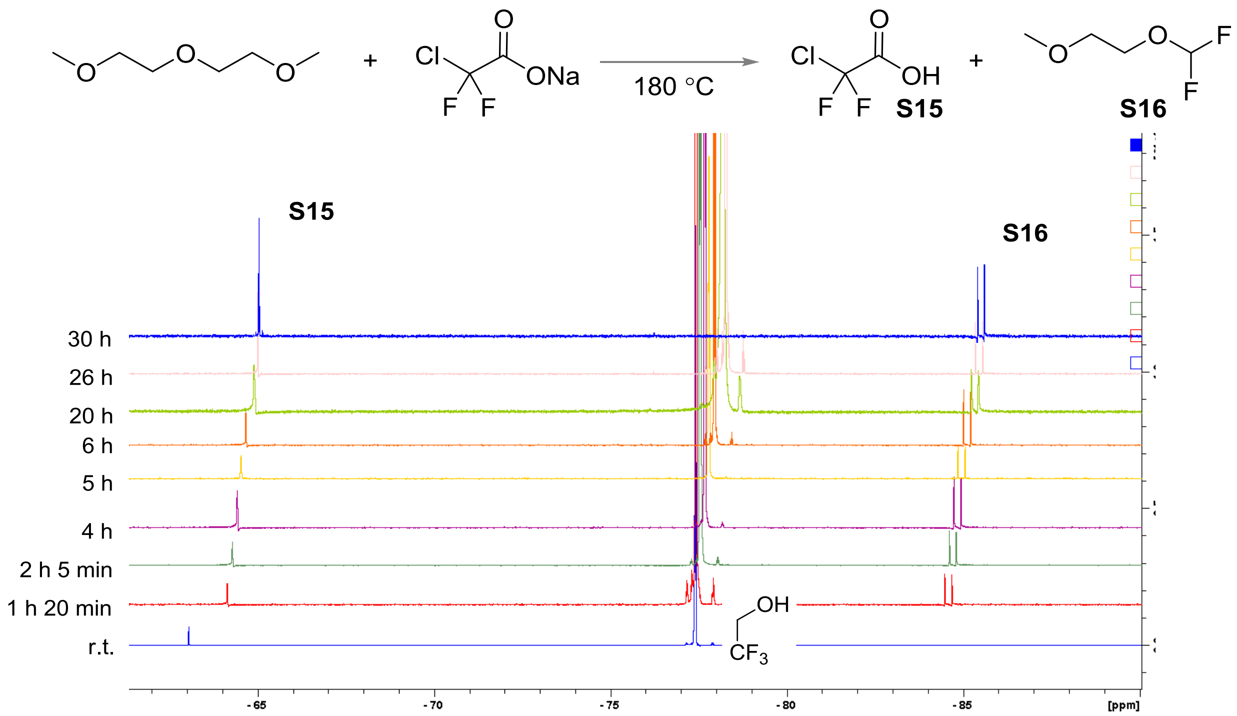


**Figure S9**: $^{19}F$ NMR ($CDCl_3$, 400 MHz) spectrum of reaction time-points showing difluorocarbene generated from sodium chlorodifluoroacetate reacting with diglmye (horizontal offset = 0.15 ppm, trifluoroethanol used as external reference).

The observed $^{19}F$ NMR experimental data for the proposed side product **S16** (-84.6 ppm, d, $^{2}J_{F\text{-}H}$ = 74.6 Hz) is extremely similar to 1,1-difluoro-3-(2-methoxyethoxy)propane reported by Rozen and co-workers[S29] (-84.6 ppm, d, $^{2}J_{F\text{-}H}$ = 75.0 Hz). Houk *et. al*. previously performed low level theoretical calculations (MP2/3-21G) showing that the attack by water on difluorocarbene resulted in a stabilisation of 9.7 kcal mol$^{-1}$ compared with the separate species, concluding that the ether stabilisation was greater than that seen with alkenes.[S30]

This side product was common when less reactive alkenoates underwent difluorocyclopropanation attempts with MDFA and thought to proceed via the attack by solvent on difluorocarbene followed by elimination of vinyl ether **S17** (**Scheme S5**).

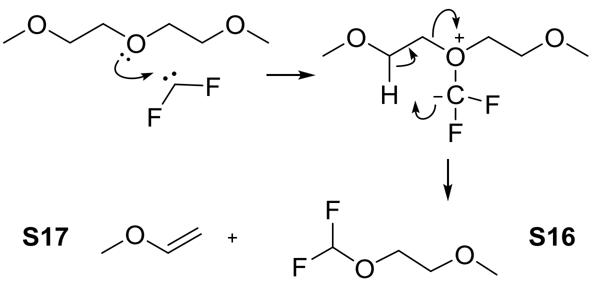


**Scheme S6:** Proposed mechanism for the reaction between diglyme and difluorocarbene.

**Trend in Difluorocyclopropanation Conversion and Calculated HOMO Energies**

The HOMO energy for a reactive species is generally considered to give a good representation of the compounds' nucleophilicity, but only a weak trend was observed between the reactivity of the alkenoates (determined by percentage conversion) and the HOMO energies of **13a-c**, **13f** and **13h** ($R^2$ = 0.8287, see Supporting Information for details). This model only assessed the reactivity of the olefin and did not take into consideration the alternative trapping reactions difluorocarbene can undergo; difluoromethylation of carboxylic acids, ketones, alcohols, thiols, heterocyclic amines and hydrophosphine oxides have all been reported.[S31-35] Compounds which reacted slowly under the difluorocyclopropanation conditions contained common side products which were attributed to difluorocarbene reacting with the reaction solvent diglyme.

Aryl alkenoates were built *de novo* using Spartan'10 software and HOMO energies obtained after geometry optimisiation using B3LYP/6-31G* methodology (gas phase, 298 K, orbitals and energies selected). Minimum energy structures were confirmed by a lack of imaginary frequency. HOMO energies are listed in **Table S2** alongside observed conversions.

**Table S2:** Difluorocyclopropanation conversion and calculated HOMO energies for (hetero)aromatic alkenoates.

| 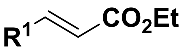 **$R^1$ =** | **Percentage Conversion (%)** | **HOMO Energy (eV)** |
|---|---|---|
| 2-Pyridyl | 0 | -6.70 |
| 2-Thiazolyl | 0 | -6.47 |
| Piperonyl | 50 | -5.68 |
| Phenyl | 37 | -6.36 |
| 2-Furyl | 66 | -5.96 |
| 2-Thiophenyl | 77 | -6.10 |
| 3-Methyl-2-furyl | 80 | -5.84 |
| 2-*N*Boc-Pyrroyl | 92 | -5.68 |

Both 2-pyridyl and 2-thiazoyl failed to show any reactivity and this could be due to their low calculated HOMO energies of -6.70 eV and -6.47 eV, respectively. However, the moderate conversion observed with piperonyl **13d** is not consistent with its higher HOMO energy of -5.68 eV, suggesting that alternative side reactions occur with these substituents. The remaining compounds show a slight trend relating percentage conversion with calculated HOMO energy but the data is not sufficient to make prediction on whether substrates will successfully undergo difluorocyclopropanation.

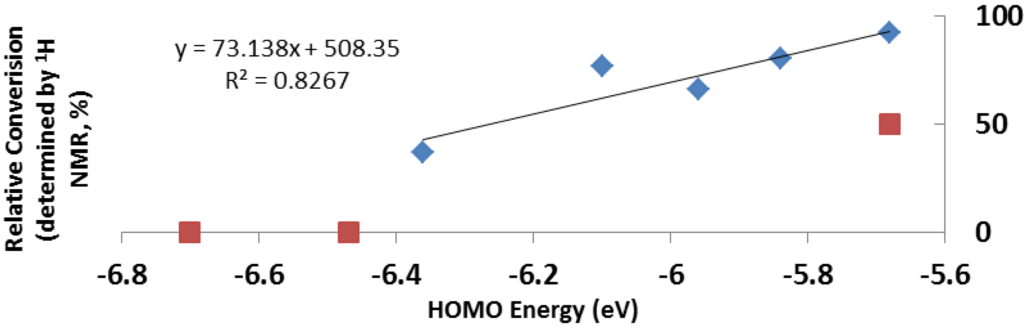


**Figure S10:** Plot of % conversion in difluorocyclopropanation reaction against calculated HOMO energies (eV) (data represented in Table SXX). ◆ = substrates which show slight trend, ■ = substrates which do not fit the trend.

**Difluorocyclopropanation Functional Group Tolerance: Additive Experiment**

We found that an indication of functional group tolerance was to measure the effects of heterocycles as additives in the previously successful difluorocyclopropanation of cinnamyl acetate **14b** (**Table S3,** Entry 1).[S20]

**Table S3:** Effect of Heterocyclic Additives on the Difluorocyclopropanation of Cinnamyl Acetate **14b**.

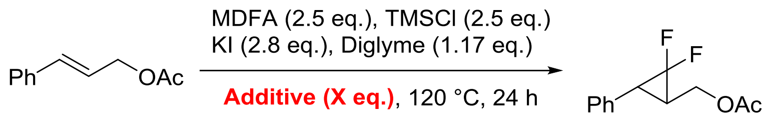


| Entry[a] | Additive | Equivalents | Conversion to S18 (%)[b] |
|---|---|---|---|
| 1[c] | none | n.a. | 100 |
| 2 | Pyridine | 1.0 | 0 |
| 3 | Pyridine | 0.5 | 4 |
| 4[c] | Pyridine | 0.1 | 85 |
| 5 | Thiazole | 0.5 | 34 |
| 6 | Pyridine-N-oxide | 0.5 | 14 |
| 7 | N-Boc-pyrrole | 0.5 | 88 |
| 8 | Methyl-3-methyl-2-furoate | 0.5 | 100 |
| 9 | 1,3-Benodioxole | 0.5 | 50 |

[a] All reactions run on an 8 mmol scale with respect to **14b** unless otherwise stated. [b] Determined by 1H NMR. [c] 16 mmol scale.

We observed that 1.0 and 0.5 equivalents of pyridine completely hindered the trapping of difluorocarbene with **14b** (**Table S3**, Entries 2-3); even small quantities of the heterocycle (10 mol%) lowered the conversion to **S18** by 15% (**Table S3**, Entry 4). Addition of thiazole to the reaction had a smaller effect, but a low 34% conversion confirmed that both of these

heterocycles do suppress the difluorocarbene-alkene reaction (**Table S3**, Entry 5). DeNinno and co-workers reported similar issues when the difluorocyclopropanation of **S19** gave a poor 22% yield, but shielding the pyridine nitrogen using *ortho*-bromo substitution gave a highly improved 78% yield from **S20** (**Scheme S6**).[S36]

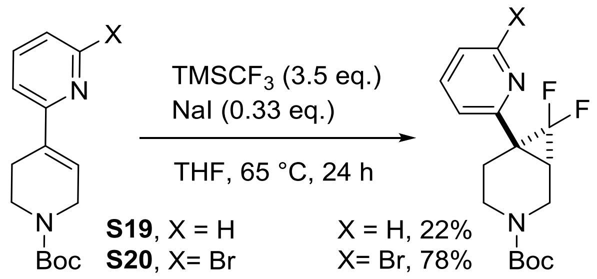


**Scheme 7:** Increased difluorocyclopropanation yields by blocking aromatic nitrogen atom.[S36]

We hoped that blocking the nitrogen atom directly via the N-oxide would help the difluorocyclopropanation reaction in a similar way and potentially give access to a more reactive VCPR precursor. However, since a poor 14% conversion to **15b** was observed when pyridine-N-oxide was used as an additive, no further synthetic investigations were carried out (**Table S3**, Entry 6). Heterocycles which were tolerated during the difluorocyclopropanation of alkenoate **13**, gave higher 88-100% conversions to **15b** when used as additives (**Table S3**, Entries 7-8). This experimental approach of assessing functional group tolerance was a more effective way to prioritising the synthesis of heteroaromatic olefins and can be used to assess which substrates will be tolerated during difluorocyclopropanation without competing side reactions.

Methodology: An oven dried two-necked round bottom flask containing potassium iodide (3.68 g, 22.2 mmol) was sealed with a SubaSeal, and the salt was stirred and lightly flame dried under an atmosphere of argon. A low boiling point water condenser with a gas outlet connected to an argon/vacuum manifold was attached and the reaction flask and the atmosphere were purged three times. Cinnamyl acetate **14b** (1.34 mL, 8.0 mmol) followed by a solution of **additive (X eq.)** in diglyme (1.3 mL) were added and the resulting suspension was heated to 120 °C. Once the reaction temperature had been reached, TMSCl (2.6 mL, 19.7 mmol) and MDFA (2.6 mL, 19.7 mmol) were added dropwise in that order. After 5 hours, the reaction mixture had evaporated to dryness and a further portion of diglyme (1.3 mL) was added. The mixture was stirred for a further 19 hours (total reaction

time of 24 hours). The resulting brown solution was cooled to room temperature and the reaction mixture was quenched with aqueous NaCl (10 mL) and diethyl ether (10 mL) was added. The organic layer was separated and the aqueous layer was extracted with diethyl ether (2 x 10 mL). The original organic layer and the extracts were combined, dried ($MgSO_4$) and concentrated under reduced pressure to remove volatiles. The $^1H$ NMR spectrum of the resulting brown oil was used to determine conversion of **13b** to difluorocyclopropyl **15b** and the results highlighted in **Table S3**.

## Crude Spectra for Low Temperature Rearrangement Precursors

Spectra below highlight the results of oxidation/Wittig chemistry on highly reactive heteroaromatic substituents.

- Furyl Alcohol **17a** with (ethoxycarbonylmethylene)triphenylphosphorane

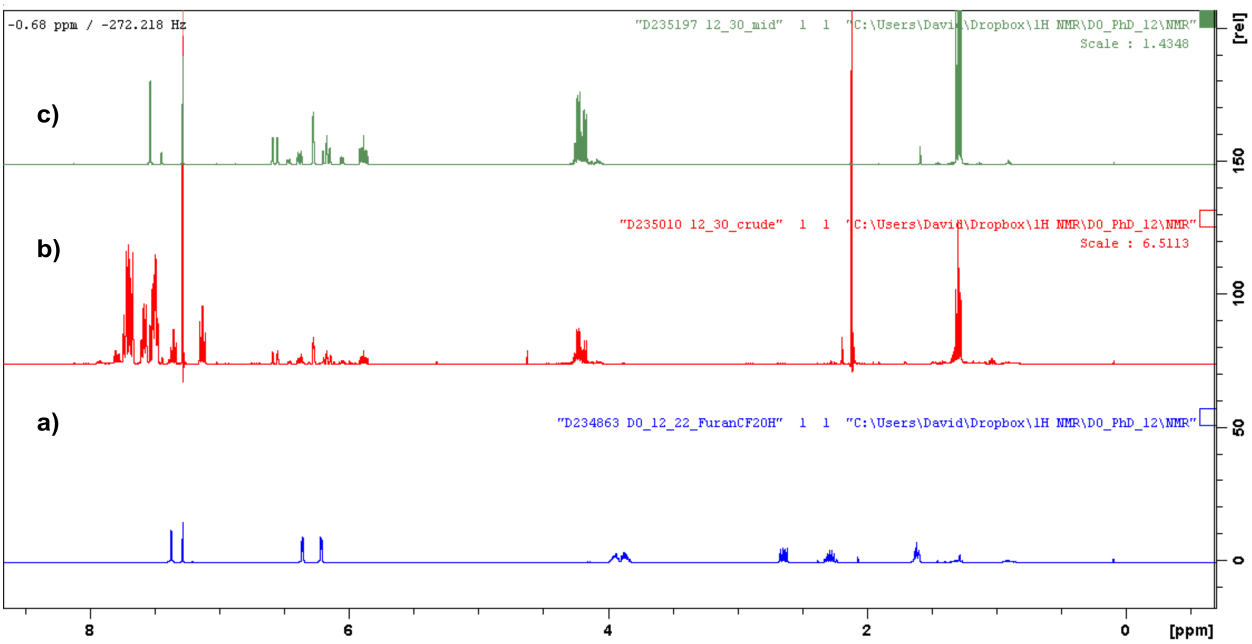


**Figure S11**: a) Isolated $^{1}$H NMR for alcohol **17a** b) Crude $^{1}$H NMR after oxidation/Wittig reaction showing no evidence of peaks relating to cyclopropane proton atoms. c) $^{1}$H NMR of main fraction isolated from first purification on silica.

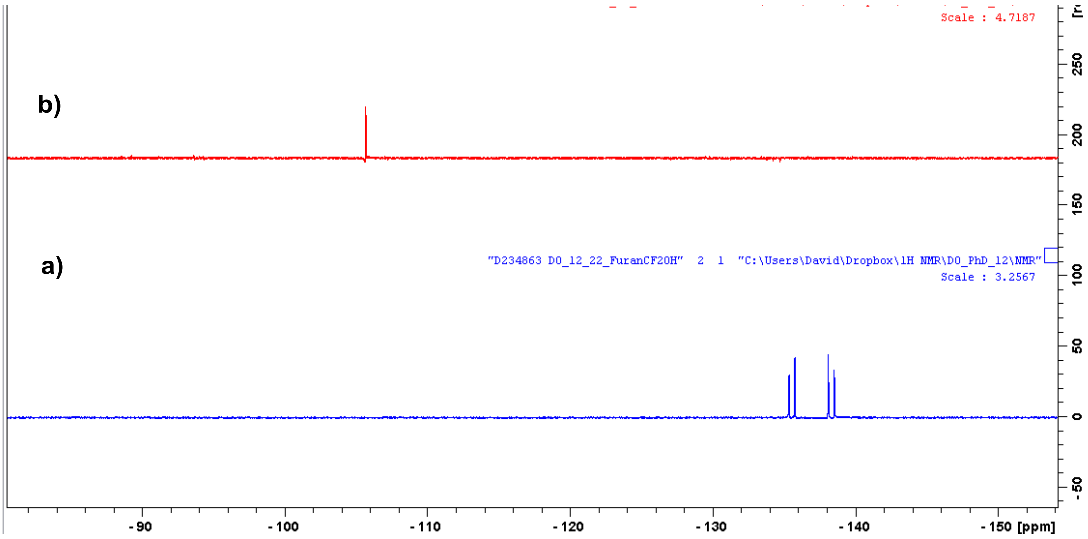


**Figure S12**: a) Isolated $^{19}$F NMR for furyl alcohol **17a** b) $^{19}$F NMR of main fraction isolated from first purification after oxidation/Wittig.

- Thiophenyl alcohol **17c** with (ethoxycarbonylmethylene)triphenylphosphorane

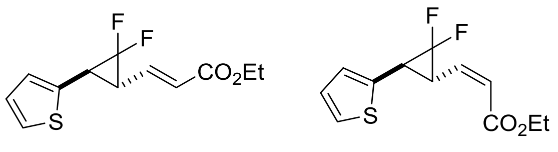


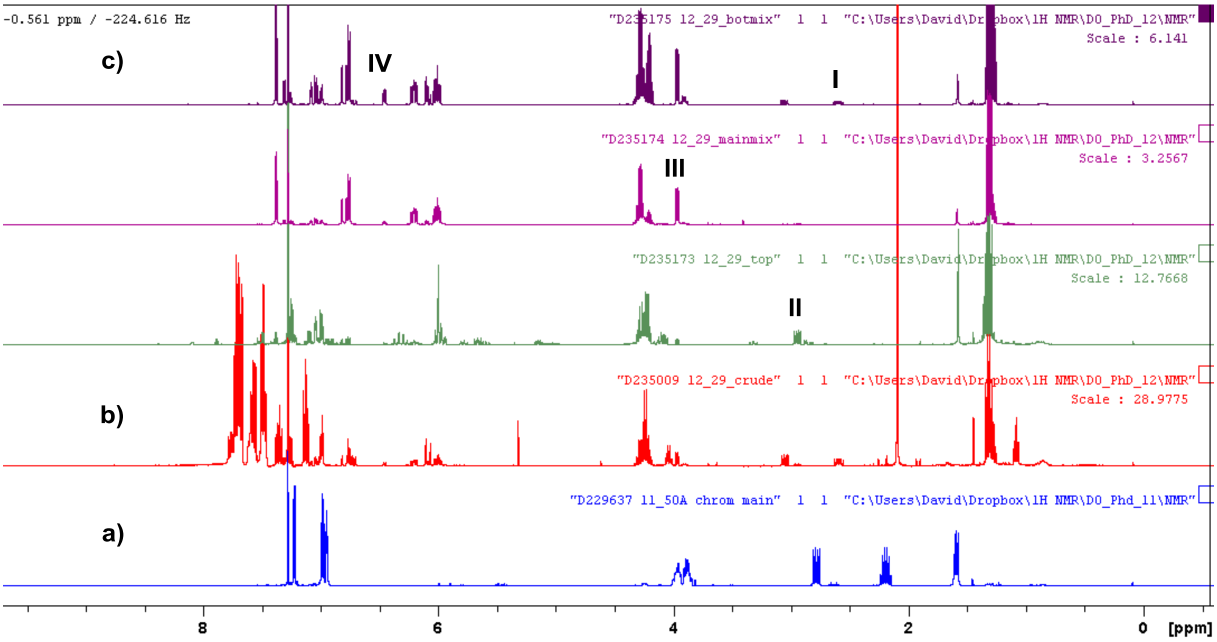


**Figure S13:** a) $^{1}$H NMR of isolated thiophenyl alcohol **17c**. b) Crude $^{1}$H NMR after oxidation/Wittig reaction. c) Three different fractions isolated after first purification on silca. (Assignment of crude spectra based on previous knowledge on *E*/*Z* cyclopropane isomers and $^{19}$F NMR data for products which was confirmed on isolation of products).

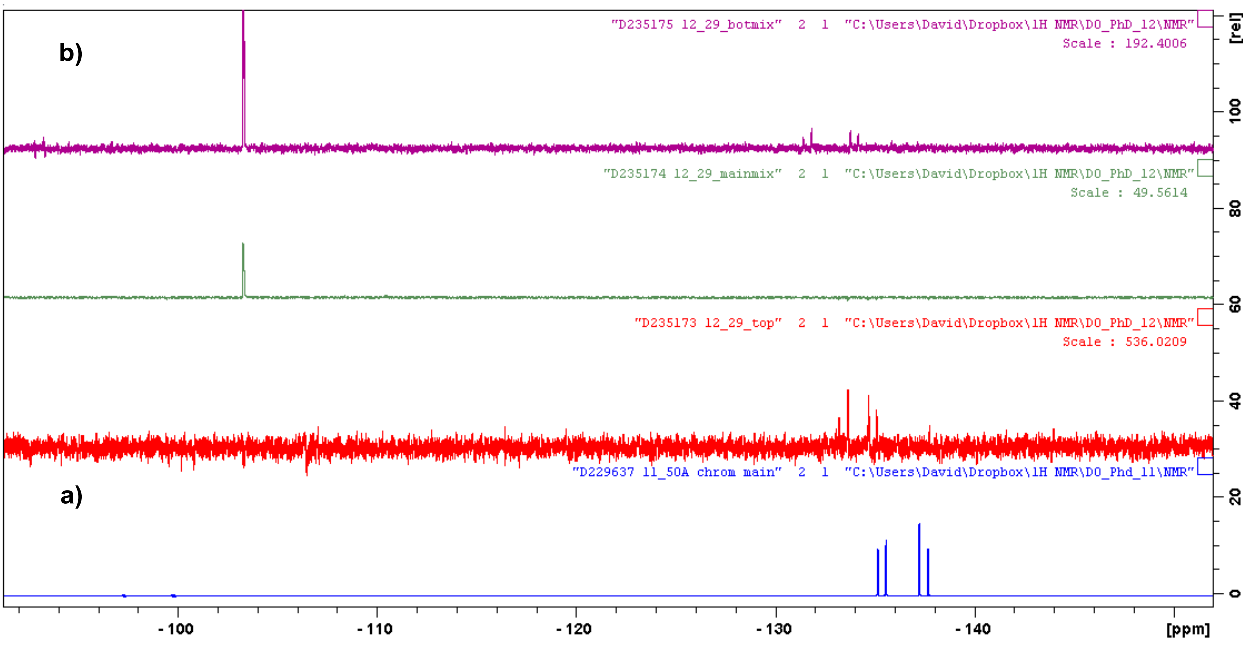


**Figure S14**: a) Crude $^{19}$F NMR for isolated thiophenyl alcohol **17c**. b) $^{19}$F NMR for isolated fractions highlighted in **Figure S13** from first purification on silica.

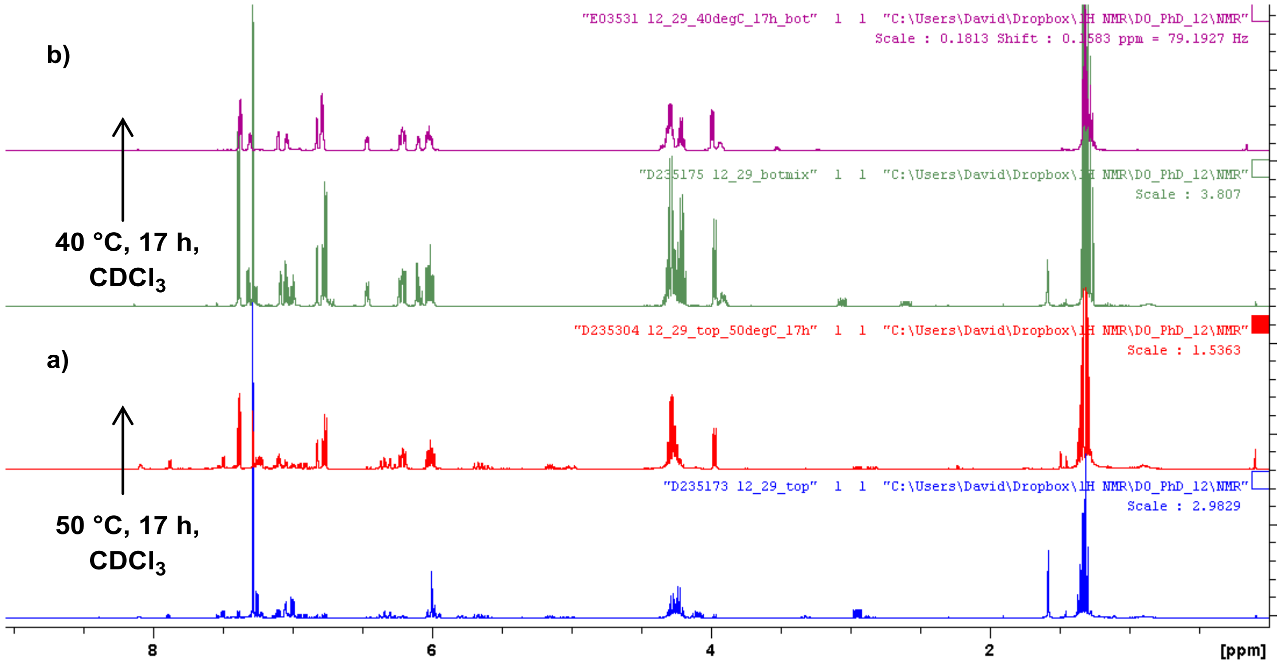


**Figure S15**: a) Thermolysis of < 10 mg sample containing *Z*-thiophenyl **24b** at 50 °C for 17 hours resulting in full conversion to heptadiene **29a** alongside other side products. b) Thermolysis of 420 mg sample containing *E*-thiophenyl **24a** at 40 °C for 17 hours resulting in full conversion to a mixture of VCPR and [3,3] product.

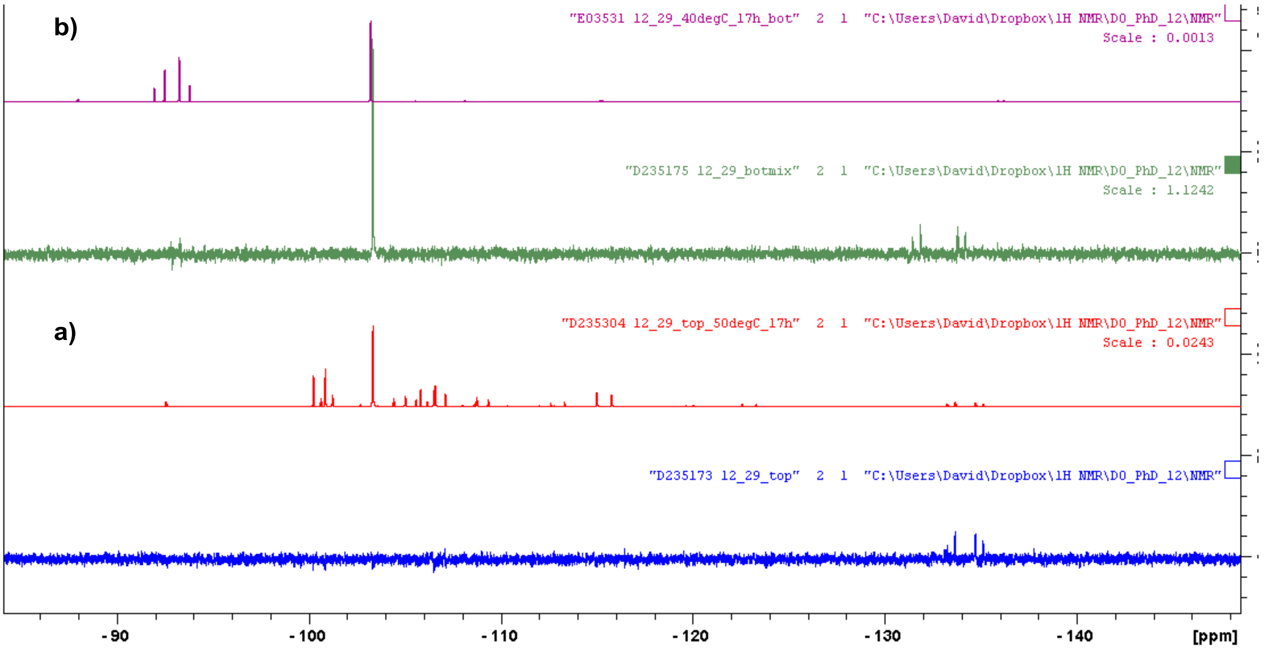


**Figure S16:** Corresponding $^{19}F$ NMR for $^{1}H$ NMR discussed in **Figure S15**.

- Furyl alcohol **17a** with (carbethoxyethylidene)triphenylphosphorane

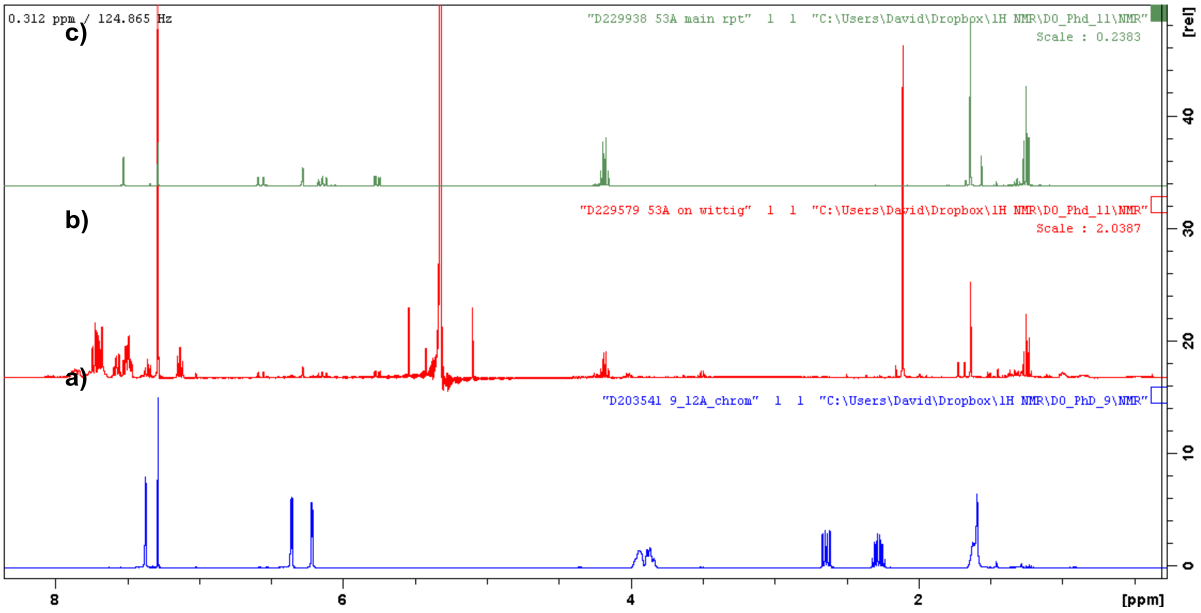


**Figure S17:** a) $^{1}H$ NMR of isolated furyl alcohol **17a**. b) Crude $^{1}H$ NMR after oxidation/Wittig with (carbethoxyethylidene)triphenylphosphorane c) $^{1}H$ NMR after 1st purification on silica.

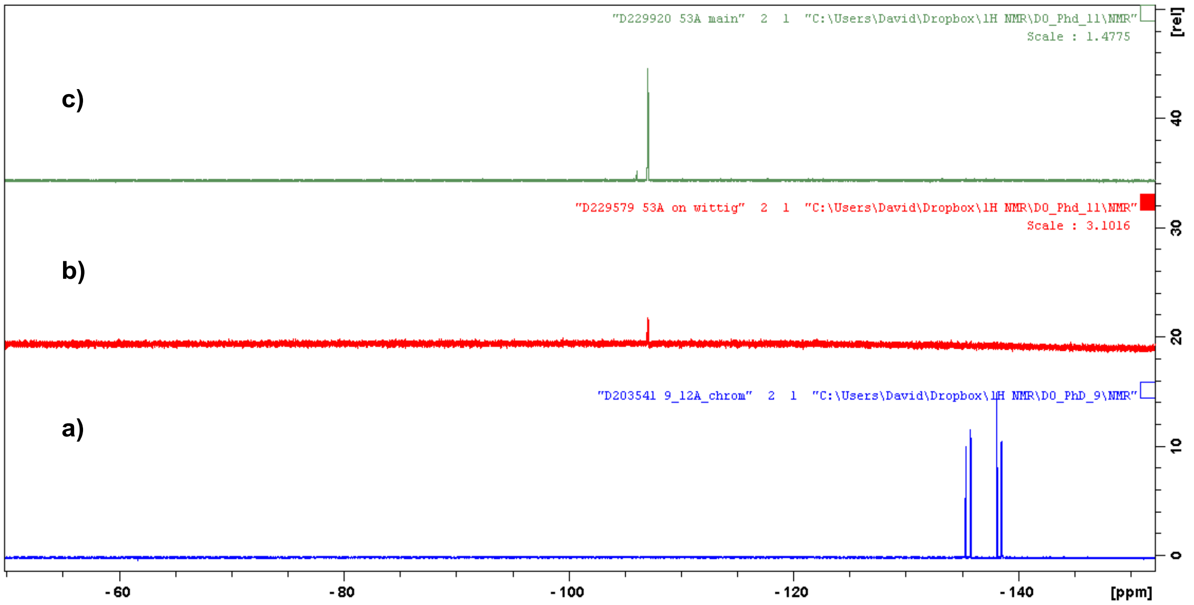


**Figure S18:** a) $^{19}$F NMR for isolated furyl alcohol **17a** b) Crude $^{19}$F NMR after oxidation/Wittig with (carbethoxyethylidene)triphenylphosphorane c) $^{19}$F NMR after 1$^{st}$ purification on silica.

- Thiophenyl alcohol **17c** with (carbethoxyethylidene)triphenylphosphorane

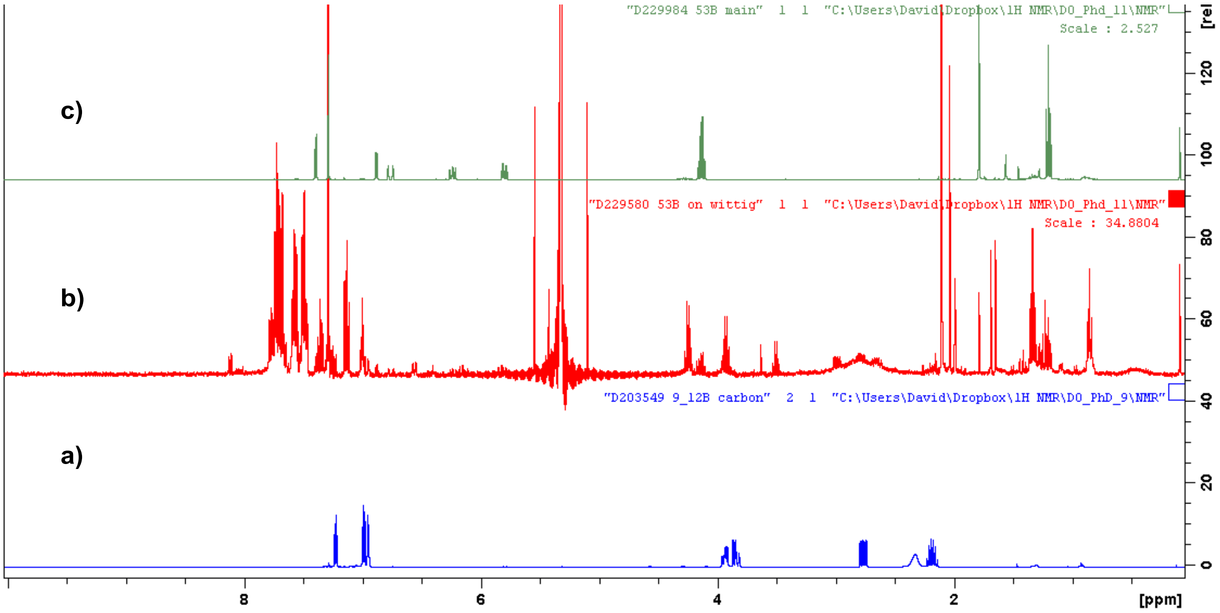


**Figure S19:** a) $^{1}$H NMR for isolated thiophenyl alcohol **17c** b) Crude $^{1}$H NMR after oxidation/Wittig with (carbethoxyethylidene)triphenylphosphorane c) $^{1}$H NMR after 1$^{st}$ purification on silica.

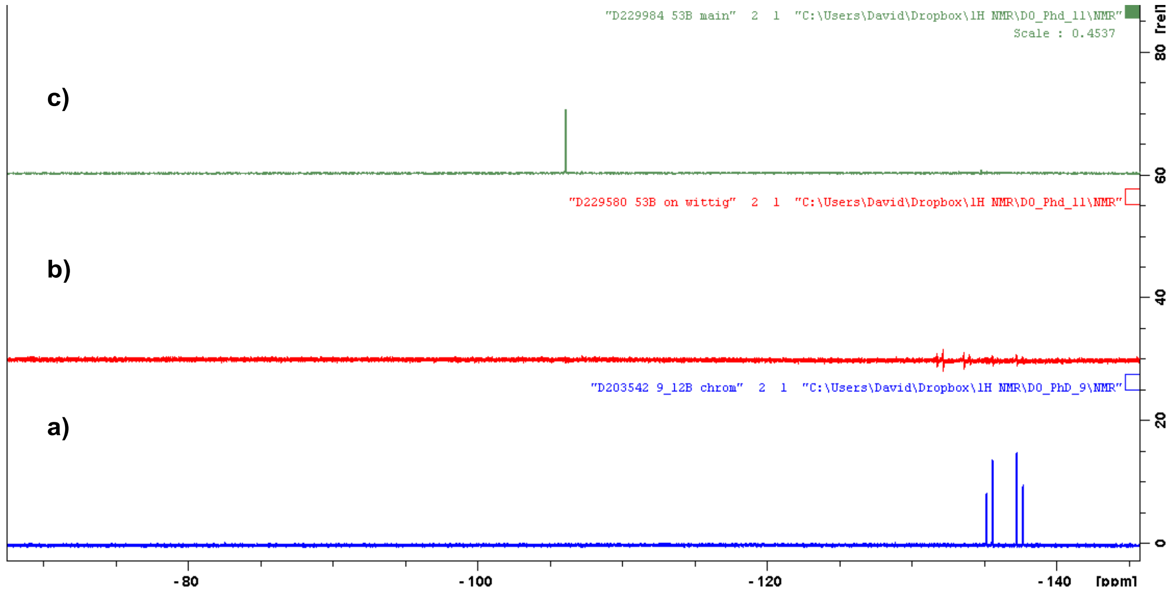


**Figure S20:** a) $^{19}$F NMR for isolated thiophenyl alcohol **17c** b) Crude $^{19}$F NMR after oxidation/Wittig with (carbethoxyethylidene)triphenylphosphorane c) $^{19}$F NMR after 1$^{st}$ purification on silica.

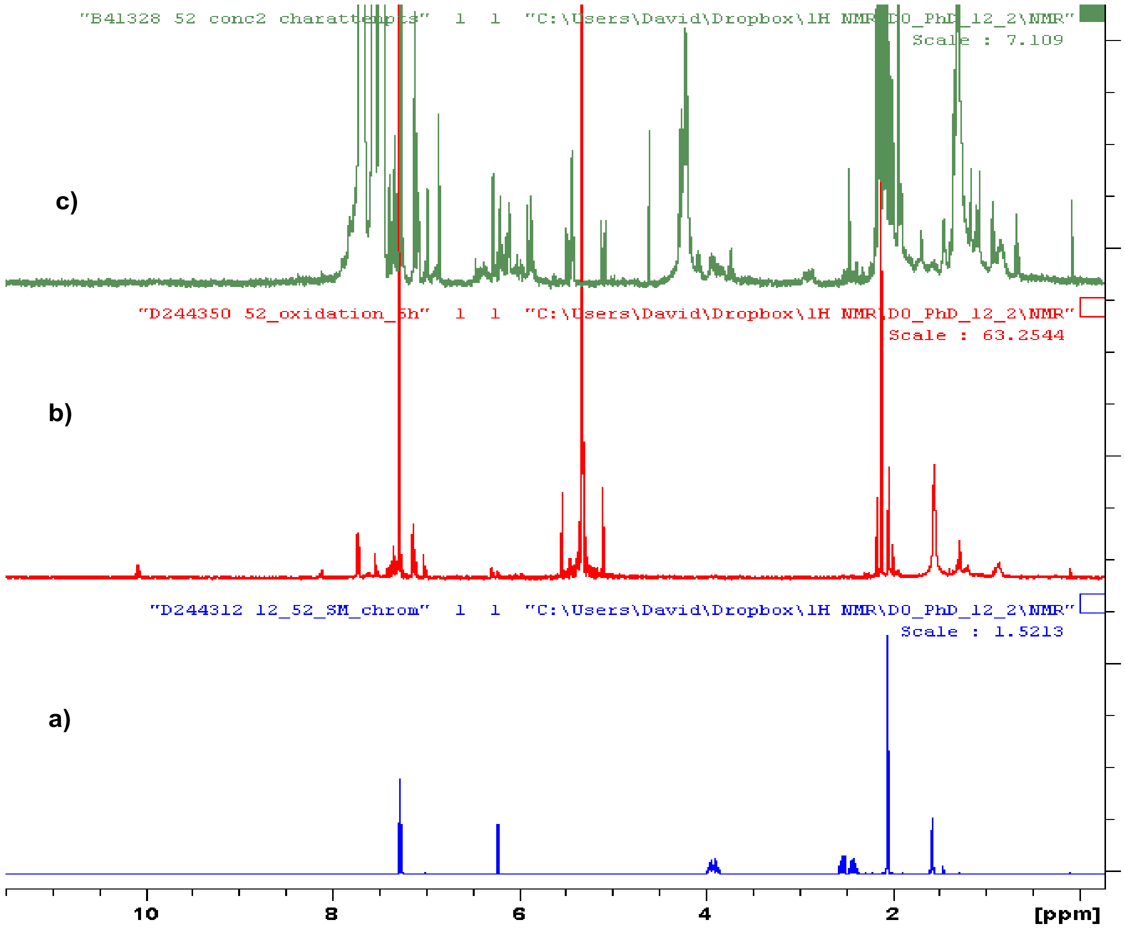


**Figure S21:** $^{1}$H NMR for isolated 3-methyl-furyl alcohol **17h** b) Crude $^{1}$H NMR after oxidation with (ethoxycarbonylmethylene)triphenylphosphorane c) $^{1}$H NMR after olefination and concentration of reaction mixture.

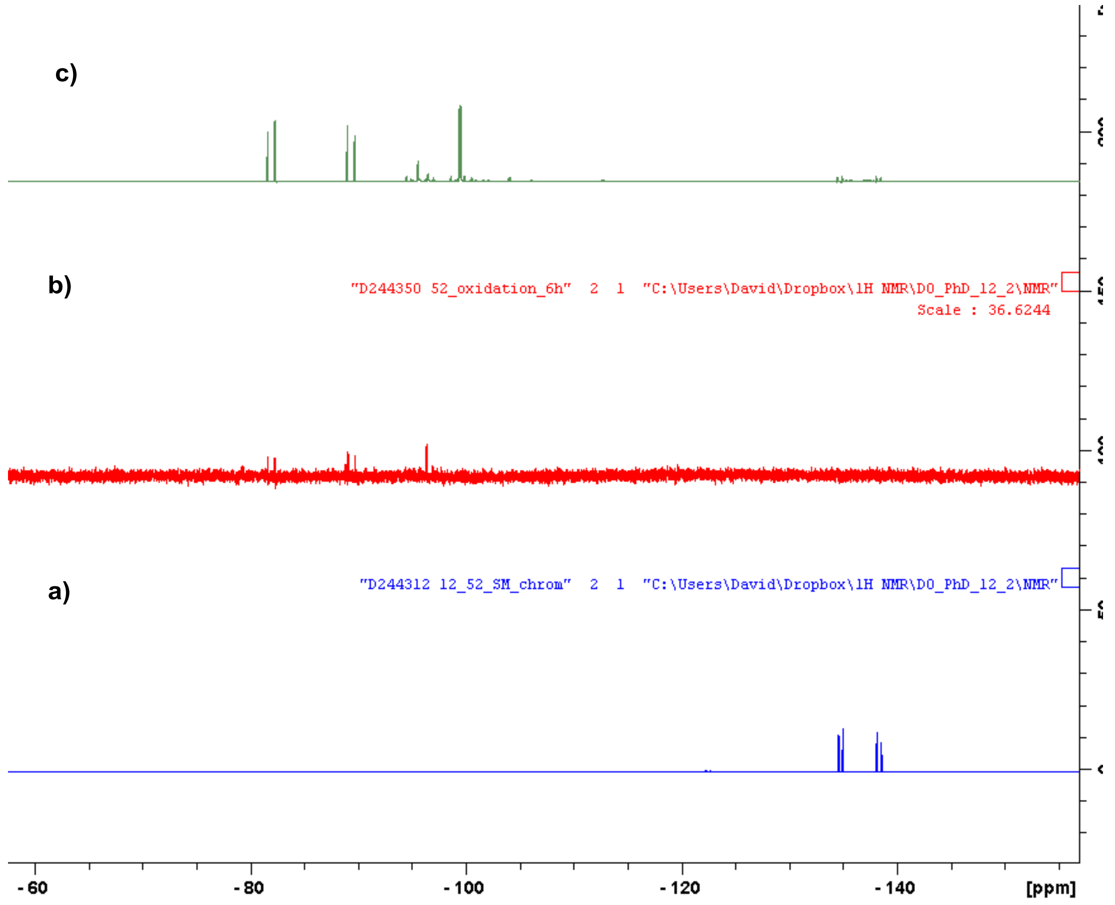


**Figure S22:** $^{19}$F NMR for isolated 3-methyl-furyl alcohol **17h** b) Crude $^{19}$F NMR after oxidation with (ethoxycarbonylmethylene)triphenylphosphorane c) $^{19}$F NMR after olefination and concentration of reaction mixture.

## Rearrangement of Isolated VCP – Crude Spectra

- **(*E*)-Weinreb Amide 19a**

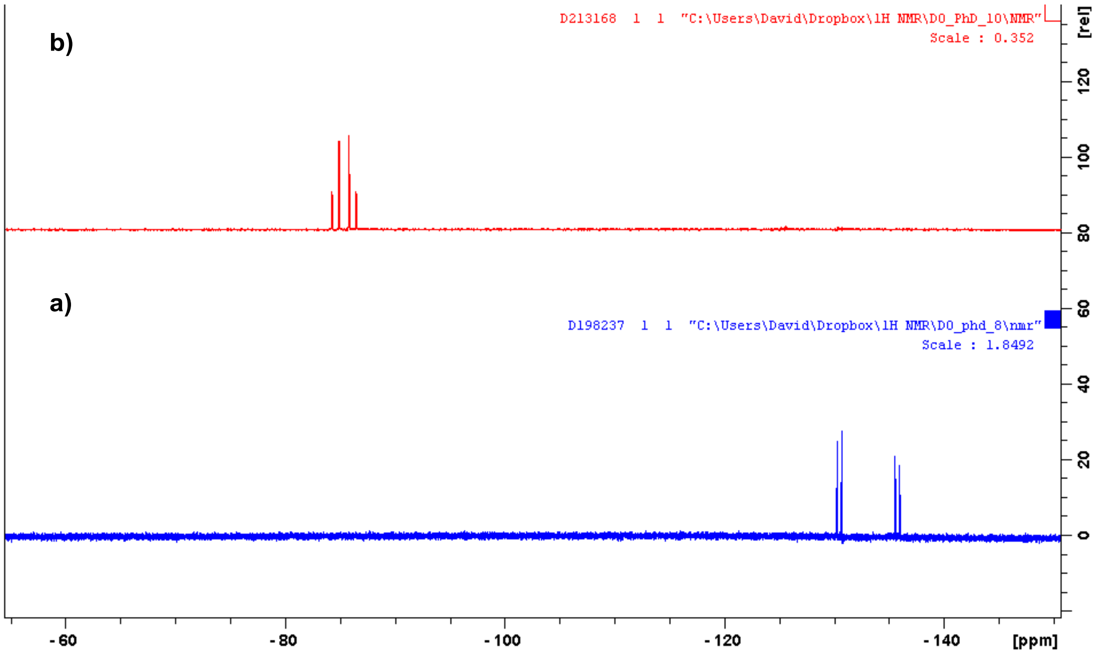


**Figure S23**: a) $^{19}$F NMR of isolated *E*-Weinreb amide VCP **19a**. b) Crude $^{19}$F NMR for rearrangement of **19a** at 95 °C for 17 hours.

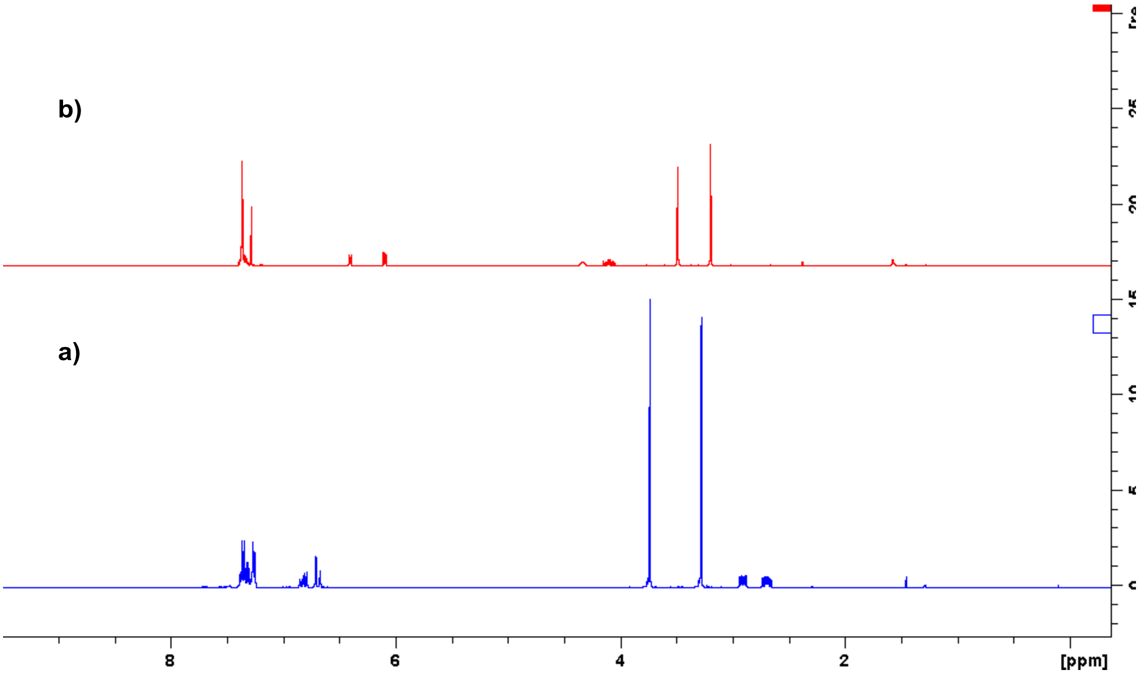


**Figure S24:** a) $^{1}$H NMR of isolated *E*-Weinreb amide VCP **19a**. b) Crude $^{1}$H NMR for rearrangement of **19a** at 95 °C for 17 hours.

- **(*Z*)-Weinreb Amide (19b)**

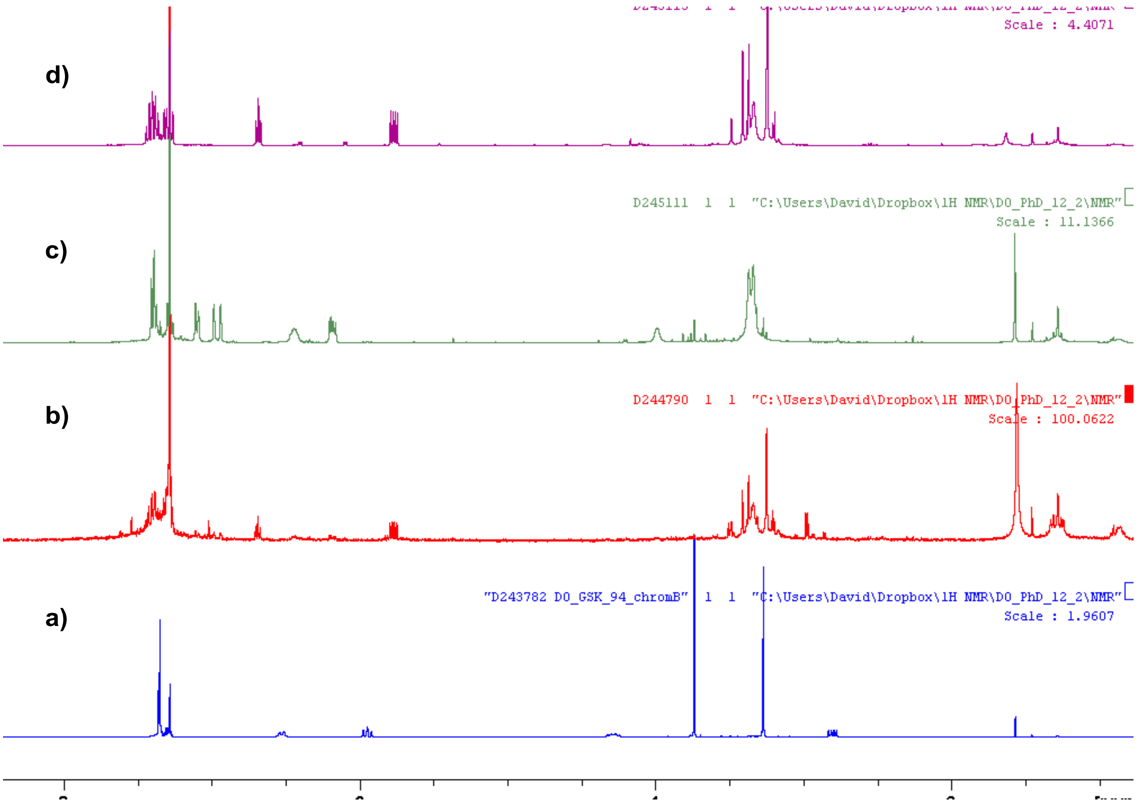


**Figure S25:** a) $^{1}$H NMR of isolated *Z*Weinreb amide VCP **19b**. b) Crude $^{1}$H NMR of **19b** after rearrangement at 165 °C for 17 hours (silica pad used to remove $Ph_2O$ solvent c) and d) $^{1}$H NMR of isolated products after chromatography.

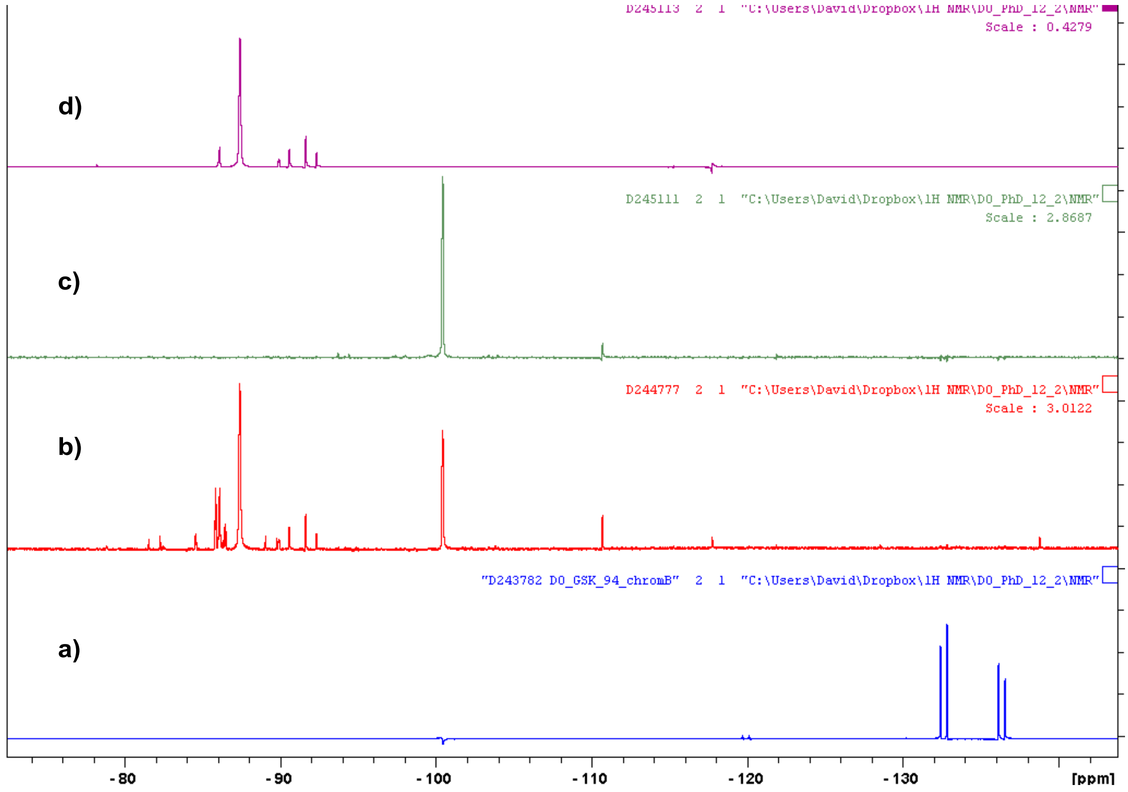


**Figure S26:** a) $^{19}$F NMR of isolated *Z*-Weinreb amide VCP **19b**. b) Crude $^{19}$F NMR of **19b** after rearrangement at 165 °C for 17 hours (silica pad used to remove $Ph_2O$ solvent. c) and d) $^{19}$F NMR of isolated products after chromatography.

- **α-Methyl Ester (20a)**

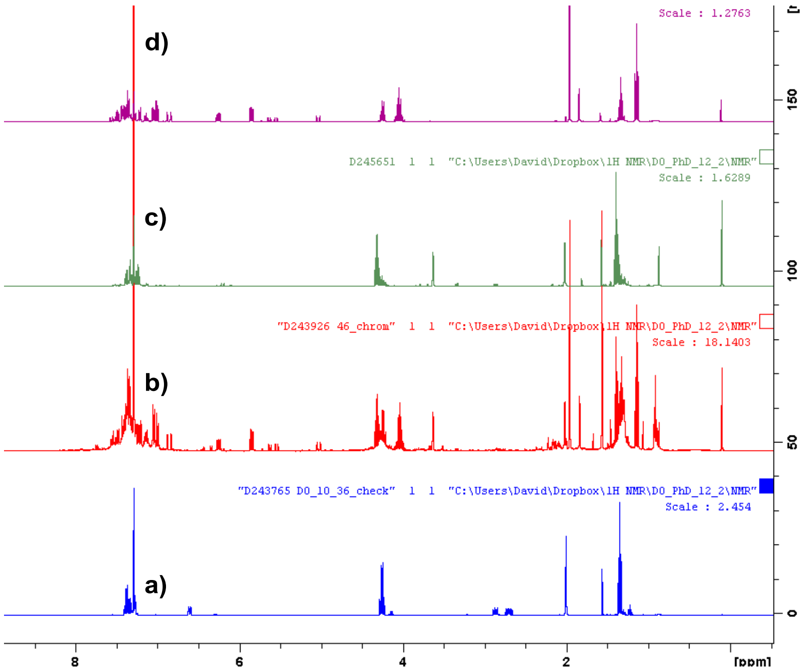


**Figure S27:** a) $^{1}$H NMR of isolated alkenoate **20a** b) Crude $^{1}$H NMR of **20a** after rearrangement at 155 °C for 17 hours (silica pad used to remove $Ph_2O$ solvent). c) and d) $^{1}$H NMR of isolated products after chromatography.

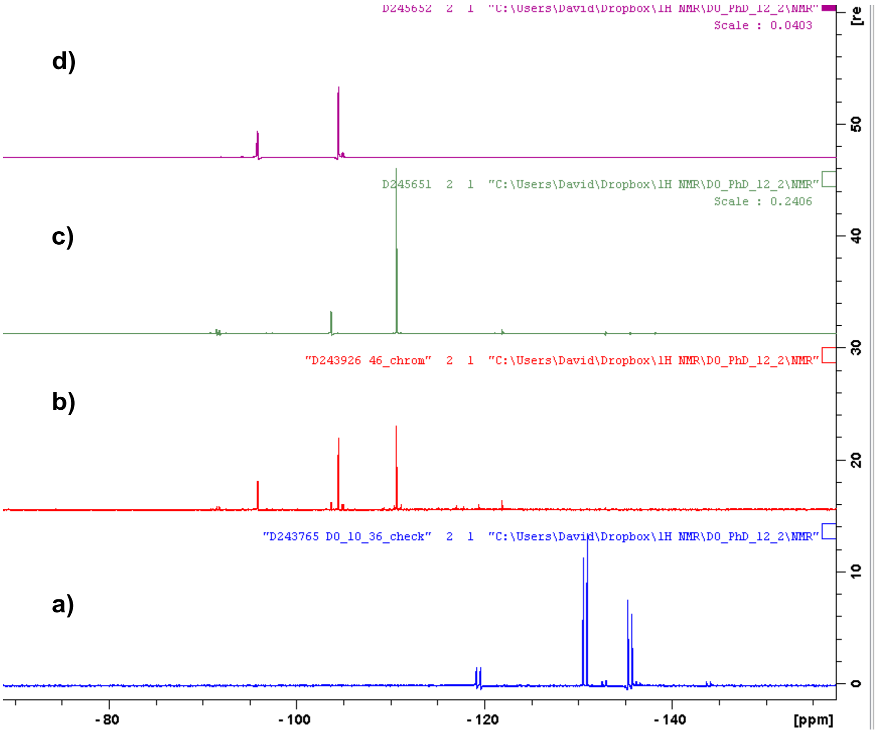


**Figure S28:** a) $^{19}$F NMR of isolated alkenoate **20a**. b) Crude $^{19}$F NMR of **20a** after rearrangement at 155 °C for 17 hours (silica pad used to remove $Ph_2O$ solvent). c) and d) $^{19}$F NMR of unknown products after chromatography.

- ***E*/*Z*-Cyano VCP mixture (21a/21b)**

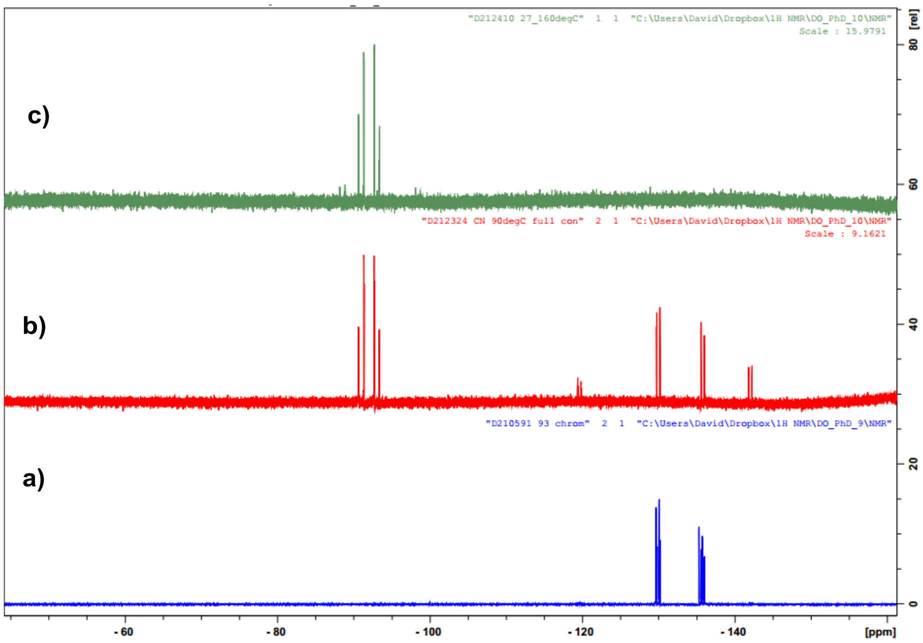


**Figure S29:** a) $^{19}$F NMR spectra of **21a/21b** mixture. b) $^{19}$F NMR spectra for the rearrangement of **21a/21b** at 90 °C for 17 h resulting in full conversion of **21a** to difluorocyclopentene **37** and only cyclopropane stereoisomeration of **21b** to **42**. c) $^{19}$F NMR spectra after the rearrangement of crude mixture in b) at 160 °C for 17 h.

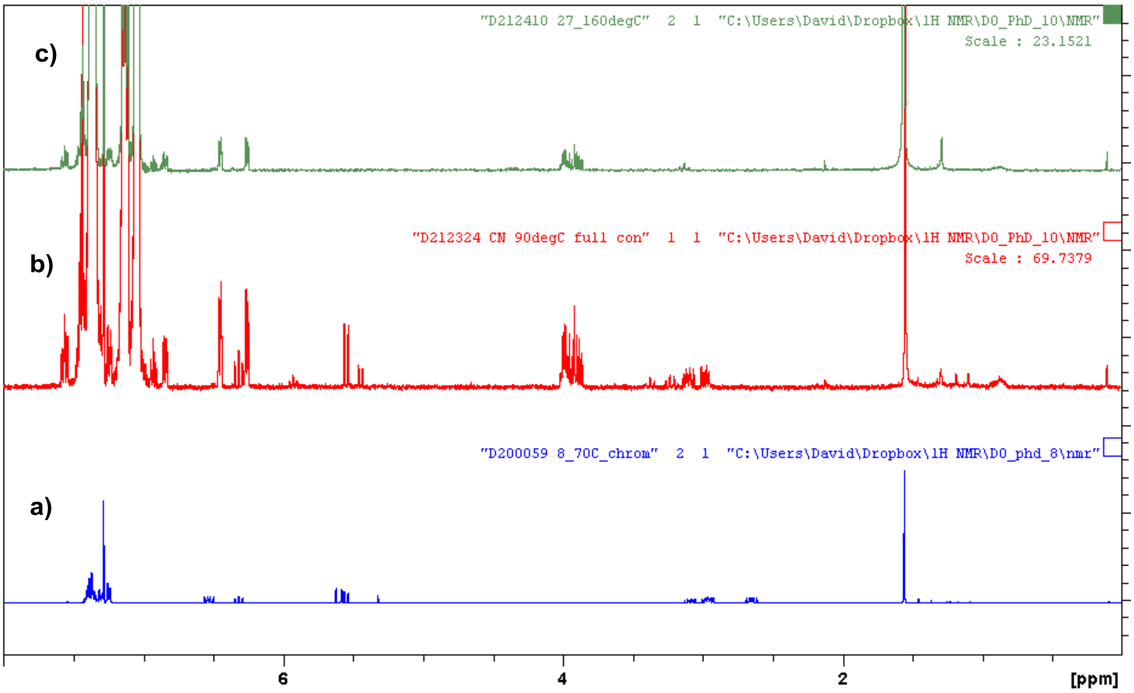


**Figure S30:** Corresponding $^{1}$H spectra for mixtures in **Figure 29.**

- **Piperonyl 22a**

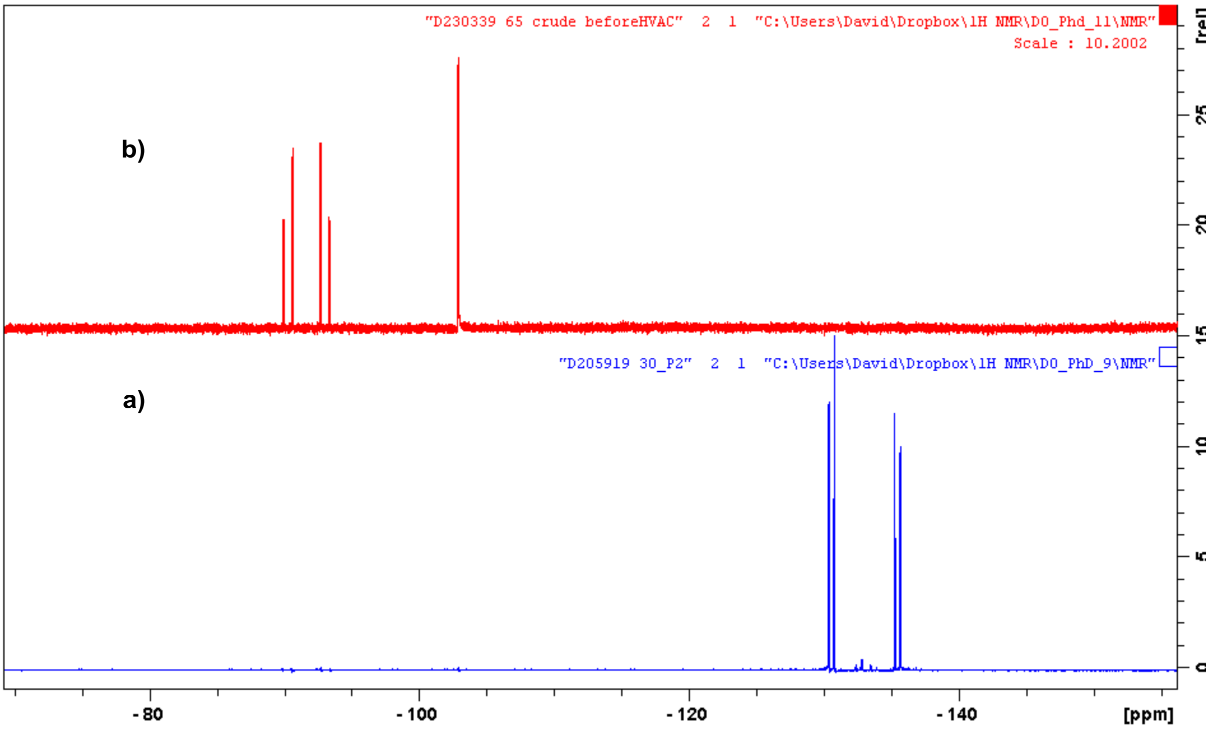


**Figure S31:** a) $^{19}$F NMR of isolated piperonyl VCP **22a**. b) $^{19}$F NMR of crude reaction mixture after rearrangement at 70 °C for 17 h.

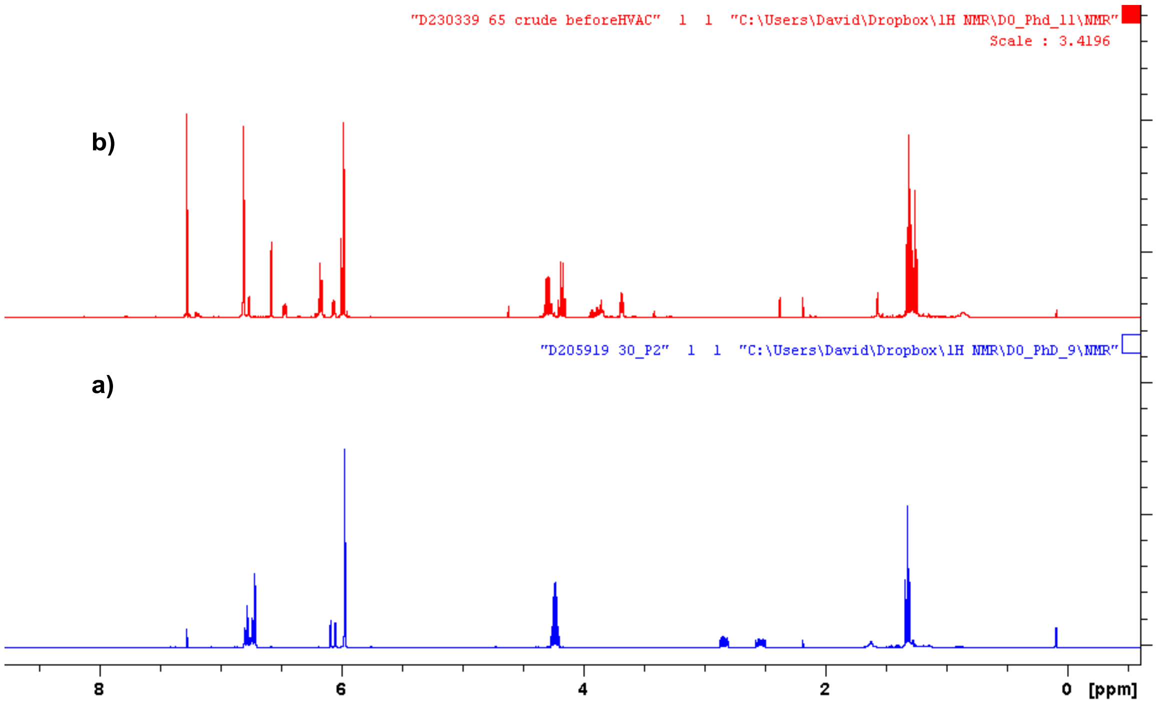


**Figure S32**: Corresponding $^{1}$H NMR spectra for $^{19}$F NMR discussed in **Figure S31**.

- **Non-fluorinated 47**

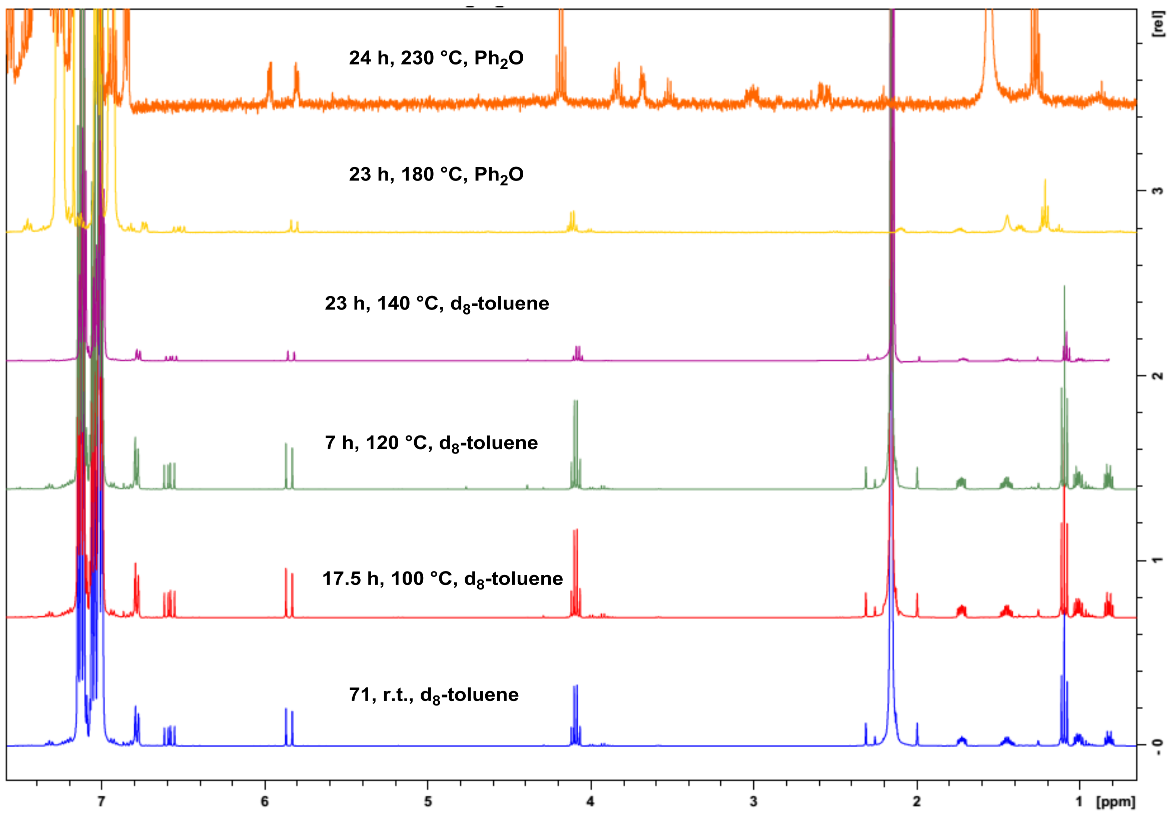


**Figure S33:** Rearrangement temperature screening show temperatures > 180 °C are required for the rearrangement of non-fluorinated VCP **47**.

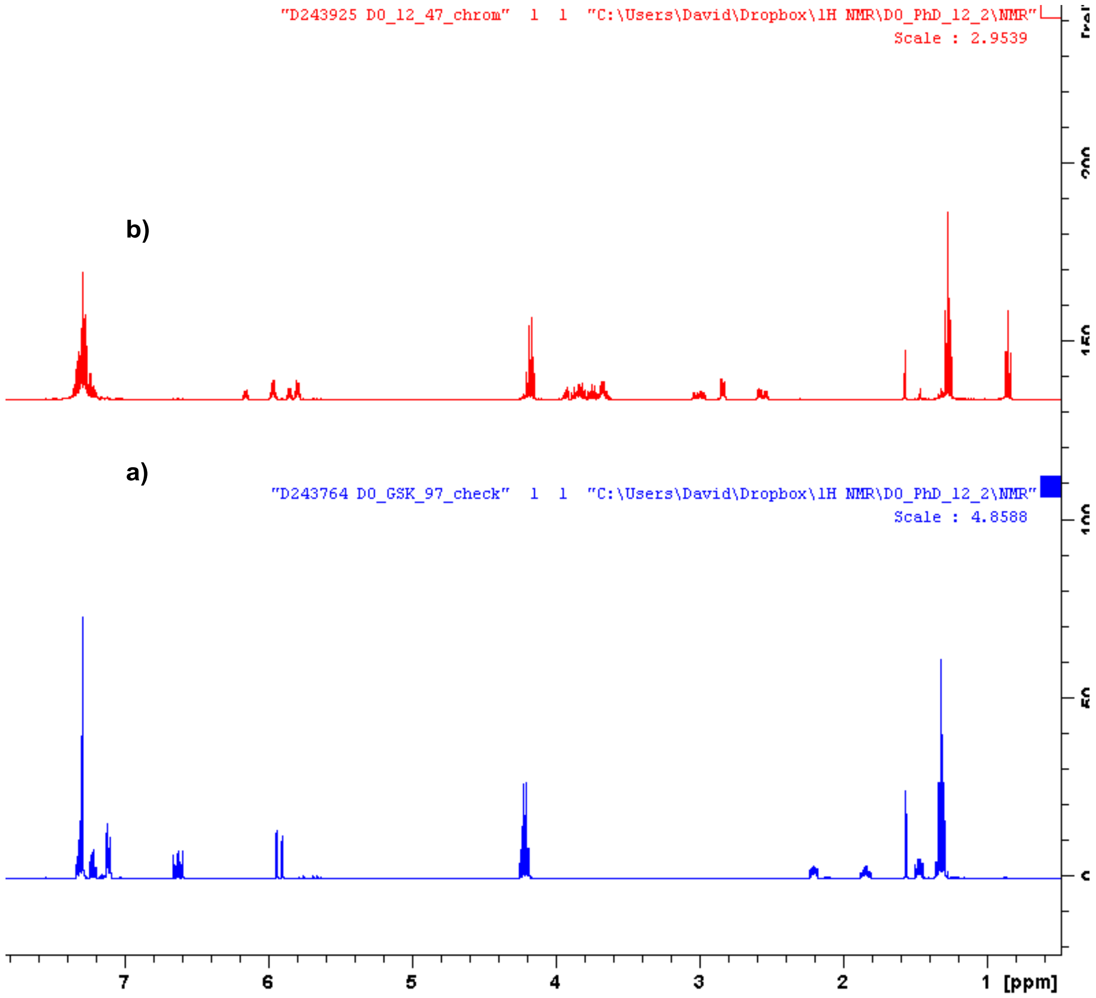


**Figure S34:** a) 1H NMR for isolated alkenoate **47** b) Crude $^{1}$H NMR for the rearrangement of isolated non-fluorinated **47** at 210 °C for 17 h (after removal of $Ph_2O$ solvent).

**Piperonyl 22a Rearrangement: Kinetic Modelling**

**VT $^{19}F$ NMR Procedure for Piperonyl 22a**

$^{19}F$ NMR spectra were acquired on a Brüker AV400 instrument equipped with a QNP-z probe and a temperature control unit. Data was collected at 376 MHz using 8 scans per data point. Settings for spectra acquisition were as follows: NS = 8 scans; D1 = 1.5 s; SW = 199.77 ppm and O1P = -100 ppm. The samples were held at reaction temperature for the duration of the experiment: 343, 353, 363 and 373 K for Arrhenius plots with **22a**. d8-Toluene was purchased from and Sigma Aldrich and used as received. A solution of VCPR precursor (typically 0.13-0.15 M) was made up in a clean, dry NMR tube and the tube was capped. The tube was inserted into the magnet and the instrument's internal temperature was set to the desired reaction temperature and allowed to equilibrate (approx. 2 minutes heating time). The sample was then analysed at appropriate intervals using a Brüker Topspin automated script, *multi_zgvd2b*. Samples were automatically shimmed using *topshim* between acquisitions. In order to obtain sufficient data points for kinetic analysis; the above procedure was altered slightly for rearrangements at temperatures of 363 and 373 K. Reactions at both temperatures used *noshim* to cancel any shimming between spectra acquisition, allowing time points to be taken every 40 or 25 seconds (respectively) instead of 60 seconds with shimming between samples. A sacrificial sample was used to shim the reaction mixture at 373 K. This was ejected and replaced with a fresh sample which was locked and followed at 25 second intervals (*multi_zgvd2b*) after solvent locking. This procedure allowed the first time point to be taken after 50 seconds instead of 180 seconds. Relative percentages were determined by averaging the manual integration for each compound on the resulting spectra using proprietary software.

**Example Spectra:**

**22a** decomposition at **373 K:** *123 seconds*

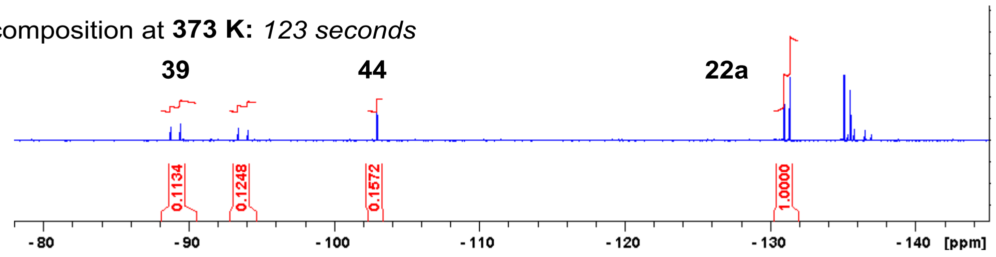


**22a** decomposition at **373 K:** *867 seconds*

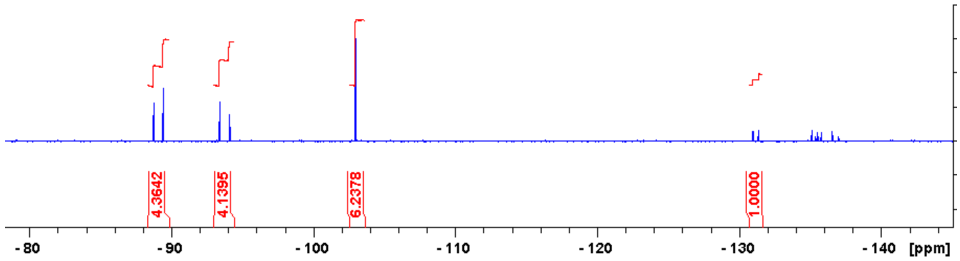

## Raw Kinetic Data for Piperonyl 22a

*$^{19}$F NMR integration data for the VCPR of **22a** in d8-toluene (0.13 M) heated at 343 K (AV400, QNP-z probe); 8 scans, D1= 1.5 s, TE = 343 K.*

| Time | Average Integrations | | | Relative Percentage | | |
|---|---|---|---|---|---|---|
| (s) | SM | VCPR | 3,3 | SM | VCPR | 3,3 |
| 0 | 1.00 | 0.03 | 0.01 | 96.4 | 3.1 | 0.5 |
| 360 | 1.00 | 0.05 | 0.02 | 93.7 | 4.6 | 1.7 |
| 480 | 1.00 | 0.07 | 0.04 | 90.0 | 6.3 | 3.7 |
| 720 | 1.00 | 0.07 | 0.06 | 88.5 | 6.2 | 5.3 |
| 960 | 1.00 | 0.11 | 0.09 | 83.5 | 9.1 | 7.5 |
| 1200 | 1.00 | 0.11 | 0.09 | 83.0 | 9.3 | 7.7 |
| 1440 | 1.00 | 0.14 | 0.15 | 77.2 | 10.9 | 11.9 |
| 1680 | 1.00 | 0.16 | 0.17 | 74.9 | 12.2 | 12.8 |
| 1920 | 1.00 | 0.19 | 0.20 | 71.8 | 14.0 | 14.2 |
| 2160 | 1.00 | 0.22 | 0.22 | 69.5 | 15.0 | 15.6 |
| 2400 | 1.00 | 0.25 | 0.27 | 66.0 | 16.4 | 17.5 |
| 2640 | 1.00 | 0.28 | 0.30 | 63.2 | 17.7 | 19.1 |
| 2880 | 1.00 | 0.28 | 0.31 | 63.0 | 17.5 | 19.5 |
| 3120 | 1.00 | 0.32 | 0.36 | 59.6 | 19.0 | 21.4 |
| 3360 | 1.00 | 0.34 | 0.36 | 58.7 | 20.1 | 21.2 |
| 3600 | 1.00 | 0.40 | 0.44 | 54.4 | 21.6 | 24.0 |
| 3840 | 1.00 | 0.38 | 0.45 | 54.4 | 20.9 | 24.7 |
| 4080 | 1.00 | 0.46 | 0.52 | 50.7 | 23.1 | 26.2 |
| 4320 | 1.00 | 0.45 | 0.51 | 50.9 | 22.9 | 26.2 |
| 4560 | 1.00 | 0.50 | 0.55 | 48.8 | 24.2 | 27.0 |
| 4800 | 1.00 | 0.57 | 0.68 | 44.5 | 25.2 | 30.3 |
| 5040 | 1.00 | 0.60 | 0.70 | 43.5 | 26.1 | 30.4 |
| 5280 | 1.00 | 0.62 | 0.72 | 42.8 | 26.4 | 30.7 |
| 5520 | 1.00 | 0.62 | 0.77 | 41.9 | 26.1 | 32.1 |
| 5760 | 1.00 | 0.69 | 0.79 | 40.2 | 27.9 | 31.9 |
| 6000 | 1.00 | 0.76 | 0.83 | 38.5 | 29.5 | 32.0 |
| 6240 | 1.00 | 0.78 | 0.95 | 36.6 | 28.5 | 34.8 |
| 6480 | 1.00 | 0.82 | 0.99 | 35.7 | 29.1 | 35.2 |
| 6720 | 1.00 | 0.88 | 1.02 | 34.5 | 30.5 | 35.0 |
| 6960 | 1.00 | 0.90 | 1.08 | 33.6 | 30.3 | 36.1 |
| 7200 | 1.00 | 0.99 | 1.15 | 31.8 | 31.7 | 36.5 |
| 7440 | 1.00 | 1.02 | 1.23 | 30.8 | 31.3 | 37.9 |
| 7680 | 1.00 | 1.09 | 1.32 | 29.3 | 32.0 | 38.8 |
| 7920 | 1.00 | 1.15 | 1.38 | 28.4 | 32.6 | 39.0 |
| 8160 | 1.00 | 1.22 | 1.51 | 26.8 | 32.6 | 40.6 |
| *8400* | *1.00* | *1.26* | *1.45* | *27.0* | *34.0* | *39.1* |

| Time | Average Integrations | | | Relative Percentage | | |
|---|---|---|---|---|---|---|
| (s) | SM | VCPR | 3,3 | SM | VCPR | 3,3 |
| 8640 | 1.00 | 1.27 | 1.56 | 26.1 | 33.2 | 40.6 |
| 8880 | 1.00 | 1.44 | 1.77 | 23.8 | 34.2 | 42.0 |
| 9120 | 1.00 | 1.54 | 1.84 | 22.8 | 35.2 | 42.0 |
| 9360 | 1.00 | 1.50 | 1.83 | 23.1 | 34.6 | 42.3 |
| 9600 | 1.00 | 1.66 | 1.96 | 21.6 | 35.9 | 42.5 |
| 9840 | 1.00 | 1.77 | 2.12 | 20.4 | 36.2 | 43.3 |
| 10080 | 1.00 | 1.81 | 2.20 | 19.9 | 36.1 | 43.9 |
| 10320 | 1.00 | 1.83 | 2.10 | 20.3 | 37.1 | 42.6 |
| 10560 | 1.00 | 1.89 | 2.37 | 19.0 | 36.0 | 45.0 |
| 10800 | 1.00 | 1.93 | 2.43 | 18.6 | 36.0 | 45.4 |
| *11280* | 1.00 | 2.09 | 2.60 | 17.6 | 36.7 | 45.7 |
| 63960 | 0.00 | 0.97 | 1.26 | 0.0 | 43.6 | 56.4 |

*$^{19}$F NMR integration data for the VCPR of **22a** in d8-toluene (0.13 M) heated at 353 K (AV400, QNP-z probe); 8 scans, D1= 1.0 s, TE = 353 K.*

| **Time** | **Average Integrations** | | | **Relative Percentage** | | |
|---|---|---|---|---|---|---|
| **(s)** | **SM** | **VCPR** | **3,3** | **SM** | **VCPR** | **3,3** |
| 0 | 1.00 | 0.04 | 0.02 | 94.0 | 3.9 | 2.2 |
| 240 | 1.00 | 0.06 | 0.05 | 90.1 | 5.8 | 4.1 |
| 420 | 1.00 | 0.12 | 0.06 | 84.4 | 10.2 | 5.4 |
| 600 | 1.00 | 0.18 | 0.18 | 73.8 | 13.1 | 13.2 |
| 780 | 1.00 | 0.23 | 0.24 | 67.6 | 15.8 | 16.5 |
| 960 | 1.00 | 0.27 | 0.31 | 63.2 | 17.1 | 19.7 |
| 1140 | 1.00 | 0.33 | 0.38 | 58.3 | 19.5 | 22.1 |
| 1320 | 1.00 | 0.43 | 0.49 | 52.1 | 22.3 | 25.6 |
| 1500 | 1.00 | 0.47 | 0.57 | 49.1 | 22.9 | 28.1 |
| 1680 | 1.00 | 0.55 | 0.65 | 45.6 | 25.0 | 29.4 |
| 1860 | 1.00 | 0.59 | 0.75 | 42.7 | 25.3 | 32.0 |
| 2040 | 1.00 | 0.73 | 0.89 | 38.2 | 27.9 | 33.8 |
| 2220 | 1.00 | 0.81 | 1.03 | 35.2 | 28.4 | 36.4 |
| 2400 | 1.00 | 0.94 | 1.15 | 32.3 | 30.5 | 37.2 |
| 2580 | 1.00 | 1.00 | 1.32 | 30.1 | 30.2 | 39.7 |
| 2760 | 1.00 | 1.20 | 1.55 | 26.7 | 32.1 | 41.3 |
| 2940 | 1.00 | 1.21 | 1.55 | 26.6 | 32.2 | 41.2 |
| 3120 | 1.00 | 1.40 | 1.78 | 23.9 | 33.5 | 42.6 |
| 3300 | 1.00 | 1.48 | 1.91 | 22.8 | 33.7 | 43.6 |
| 3480 | 1.00 | 1.71 | 2.24 | 20.2 | 34.6 | 45.2 |
| 3660 | 1.00 | 2.01 | 2.64 | 17.7 | 35.6 | 46.7 |
| 3840 | 1.00 | 2.03 | 2.59 | 17.8 | 36.1 | 46.1 |
| 4020 | 1.00 | 2.27 | 2.91 | 16.2 | 36.7 | 47.1 |
| 4200 | 1.00 | 2.85 | 3.80 | 13.1 | 37.3 | 49.6 |
| 4380 | 1.00 | 2.75 | 3.64 | 13.5 | 37.2 | 49.3 |
| 4560 | 1.00 | 3.23 | 4.31 | 11.7 | 37.8 | 50.5 |
| 4740 | 1.00 | 3.32 | 4.34 | 11.5 | 38.3 | 50.2 |
| 4920 | 1.00 | 4.35 | 5.59 | 9.1 | 39.8 | 51.1 |
| 5100 | 1.00 | 4.13 | 5.38 | 9.5 | 39.3 | 51.2 |
| 5280 | 1.00 | 4.26 | 5.65 | 9.2 | 39.1 | 51.8 |
| 5460 | 1.00 | 5.00 | 6.58 | 7.9 | 39.8 | 52.3 |
| 5640 | 1.00 | 4.81 | 6.06 | 8.4 | 40.5 | 51.1 |
| 5820 | 1.00 | 6.21 | 8.21 | 6.5 | 40.3 | 53.2 |
| 6000 | 1.00 | 6.75 | 8.85 | 6.0 | 40.7 | 53.3 |
| 6180 | 1.00 | 8.11 | 10.43 | 5.1 | 41.5 | 53.4 |
| 6360 | 1.00 | 5.79 | 7.55 | 7.0 | 40.4 | 52.7 |
| 6540 | 1.00 | 9.03 | 12.02 | 4.5 | 41.0 | 54.5 |
| 6720 | 1.00 | 9.71 | 12.64 | 4.3 | 41.6 | 54.1 |
| 6900 | 1.00 | 10.78 | 14.33 | 3.8 | 41.3 | 54.9 |
| 7080 | 1.00 | 9.40 | 12.23 | 4.4 | 41.5 | 54.0 |
| 7260 | 1.00 | 10.10 | 12.74 | 4.2 | 42.4 | 53.4 |
| 7440 | 1.00 | 12.65 | 16.46 | 3.3 | 42.0 | 54.7 |
| 7620 | 1.00 | 12.47 | 16.40 | 3.3 | 41.7 | 54.9 |
| *15120* | *0.00* | *1.01* | *1.61* | *0.0* | *38.4* | *61.6* |

$^{19}$F NMR integration data for the VCPR of **22a** in d8-toluene (0.13 M) heated at 363 K (AV400, QNP-z probe); 8 scans, D1= 1.0 s, TE = 363 K.

| Time | Average Integrations | | | Relative Percentage | | |
|---|---|---|---|---|---|---|
| (s) | SM | VCPR | 3,3 | SM | VCPR | 3,3 |
| 0 | 1.00 | 0.03 | 0.02 | 95.0 | 2.8 | 2.1 |
| 120 | 1.00 | 0.11 | 0.10 | 82.8 | 8.8 | 8.4 |
| 160 | 1.00 | 0.12 | 0.13 | 80.0 | 9.9 | 10.0 |
| 190 | 1.00 | 0.15 | 0.15 | 77.2 | 11.4 | 11.4 |
| 210 | 1.00 | 0.17 | 0.19 | 73.2 | 12.8 | 14.0 |
| 245 | 1.00 | 0.18 | 0.22 | 71.4 | 13.1 | 15.5 |
| 275 | 1.00 | 0.22 | 0.27 | 67.2 | 14.6 | 18.2 |
| 305 | 1.00 | 0.23 | 0.30 | 65.7 | 14.9 | 19.4 |
| 335 | 1.00 | 0.26 | 0.31 | 63.4 | 16.7 | 19.9 |
| 365 | 1.00 | 0.30 | 0.34 | 61.0 | 18.1 | 20.9 |
| 395 | 1.00 | 0.32 | 0.37 | 59.2 | 18.8 | 22.0 |
| 425 | 1.00 | 0.36 | 0.43 | 55.9 | 20.1 | 24.1 |
| 455 | 1.00 | 0.36 | 0.47 | 54.4 | 19.8 | 25.7 |
| 485 | 1.00 | 0.43 | 0.51 | 51.8 | 22.1 | 26.2 |
| 515 | 1.00 | 0.43 | 0.55 | 50.6 | 21.7 | 27.7 |
| 545 | 1.00 | 0.44 | 0.55 | 50.3 | 21.9 | 27.8 |
| 575 | 1.00 | 0.48 | 0.63 | 47.3 | 22.8 | 29.8 |
| 605 | 1.00 | 0.52 | 0.66 | 45.7 | 24.0 | 30.3 |
| 635 | 1.00 | 0.54 | 0.71 | 44.5 | 23.9 | 31.6 |
| 665 | 1.00 | 0.59 | 0.80 | 42.0 | 24.6 | 33.4 |
| 695 | 1.00 | 0.64 | 0.83 | 40.5 | 25.9 | 33.6 |
| 725 | 1.00 | 0.69 | 0.85 | 39.3 | 27.1 | 33.6 |
| 755 | 1.00 | 0.70 | 0.92 | 38.2 | 26.8 | 35.0 |
| 785 | 1.00 | 0.73 | 0.98 | 36.9 | 26.8 | 36.3 |
| 815 | 1.00 | 0.77 | 1.06 | 35.3 | 27.1 | 37.5 |
| 845 | 1.00 | 0.86 | 1.13 | 33.5 | 28.8 | 37.7 |
| 875 | 1.00 | 0.86 | 1.15 | 33.2 | 28.6 | 38.2 |
| 905 | 1.00 | 0.93 | 1.23 | 31.7 | 29.4 | 39.0 |
| 935 | 1.00 | 0.96 | 1.28 | 30.8 | 29.8 | 39.4 |
| 965 | 1.00 | 1.02 | 1.35 | 29.7 | 30.3 | 40.0 |
| 995 | 1.00 | 1.09 | 1.47 | 28.0 | 30.7 | 41.3 |
| 1025 | 1.00 | 1.16 | 1.64 | 26.3 | 30.5 | 43.2 |
| 1055 | 1.00 | 1.18 | 1.65 | 26.1 | 30.8 | 43.1 |
| 1085 | 1.00 | 1.30 | 1.72 | 24.9 | 32.4 | 42.7 |
| 1115 | 1.00 | 1.27 | 1.75 | 24.9 | 31.6 | 43.5 |
| 1145 | 1.00 | 1.41 | 1.91 | 23.2 | 32.6 | 44.2 |
| 1175 | 1.00 | 1.38 | 1.88 | 23.5 | 32.4 | 44.1 |
| 1205 | 1.00 | 1.56 | 2.07 | 21.6 | 33.7 | 44.7 |
| 1235 | 1.00 | 1.78 | 2.43 | 19.2 | 34.2 | 46.7 |
| 1265 | 1.00 | 1.72 | 2.35 | 19.7 | 34.0 | 46.3 |

| Time | Average Integrations | | | Relative Percentage | | |
|---|---|---|---|---|---|---|
| (s) | SM | VCPR | 3,3 | SM | VCPR | 3,3 |
| 1295 | 1.00 | 1.69 | 2.41 | 19.6 | 33.1 | 47.3 |
| 1325 | 1.00 | 1.87 | 2.51 | 18.6 | 34.8 | 46.6 |
| 1355 | 1.00 | 1.86 | 2.60 | 18.3 | 34.1 | 47.6 |
| 1385 | 1.00 | 1.95 | 2.65 | 17.9 | 34.8 | 47.3 |
| 1415 | 1.00 | 2.48 | 3.36 | 14.6 | 36.2 | 49.1 |
| 1445 | 1.00 | 2.31 | 3.18 | 15.4 | 35.6 | 49.0 |
| 1475 | 1.00 | 2.65 | 3.57 | 13.9 | 36.6 | 49.5 |
| 1505 | 1.00 | 2.31 | 3.08 | 15.6 | 36.1 | 48.2 |
| 1535 | 1.00 | 2.70 | 3.55 | 13.8 | 37.2 | 49.0 |
| 1565 | 1.00 | 2.65 | 3.68 | 13.6 | 36.2 | 50.2 |
| 1595 | 1.00 | 3.04 | 4.27 | 12.0 | 36.5 | 51.4 |
| 1625 | 1.00 | 3.07 | 4.41 | 11.8 | 36.2 | 52.0 |
| 1655 | 1.00 | 2.96 | 4.16 | 12.3 | 36.5 | 51.2 |
| 1685 | 1.00 | 3.04 | 4.26 | 12.1 | 36.6 | 51.3 |
| 1715 | 1.00 | 3.22 | 4.44 | 11.6 | 37.2 | 51.3 |
| 1745 | 1.00 | 4.05 | 5.63 | 9.4 | 37.9 | 52.7 |
| 1775 | 1.00 | 3.50 | 4.64 | 10.9 | 38.3 | 50.8 |
| 1805 | 1.00 | 3.63 | 4.95 | 10.4 | 37.9 | 51.7 |
| 1835 | 1.00 | 4.26 | 5.99 | 8.9 | 37.9 | 53.2 |
| 1865 | 1.00 | 4.10 | 5.49 | 9.4 | 38.7 | 51.8 |
| 1895 | 1.00 | 4.71 | 6.66 | 8.1 | 38.1 | 53.9 |
| 1925 | 1.00 | 5.27 | 7.43 | 7.3 | 38.4 | 54.3 |
| 1955 | 1.00 | 4.75 | 6.31 | 8.3 | 39.4 | 52.3 |
| 1985 | 1.00 | 5.47 | 7.64 | 7.1 | 38.8 | 54.1 |
| 2015 | 1.00 | 4.68 | 6.58 | 8.2 | 38.2 | 53.7 |
| 2045 | 1.00 | 4.82 | 6.57 | 8.1 | 38.9 | 53.0 |
| 2075 | 1.00 | 6.13 | 8.45 | 6.4 | 39.4 | 54.2 |
| 2105 | 1.00 | 5.63 | 7.83 | 6.9 | 38.9 | 54.2 |
| 2135 | 1.00 | 5.74 | 8.28 | 6.7 | 38.2 | 55.1 |
| 2165 | 1.00 | 6.95 | 9.38 | 5.8 | 40.1 | 54.1 |
| 2315 | 1.00 | 12.12 | 16.74 | 3.3 | 40.6 | 56.1 |
| 2465 | 1.00 | 11.96 | 16.63 | 3.4 | 40.4 | 56.2 |
| 2525 | 1.00 | 10.33 | 14.72 | 3.8 | 39.6 | 56.5 |
| 2585 | 1.00 | 10.78 | 15.52 | 3.7 | 39.5 | 56.9 |
| 2645 | 1.00 | 14.73 | 21.04 | 2.7 | 40.0 | 57.2 |
| *6360* | *1.00* | *55.62* | *98.61* | *0.6* | *35.8* | *63.5* |

$^{19}$F NMR integration data for the VCPR of **22a** in d8-toluene (0.15 M) heated at 373 K (AV400, QNP-z probe); 8 scans, D1= 1.0 s, TE = 373 K.

| Time | Average Integrations | | | Relative Percentage | | |
|---|---|---|---|---|---|---|
| (s) | SM | VCPR | 3,3 | SM | VCPR | 3,3 |
| 51 | 1.00 | 0.07 | 0.04 | 90.6 | 6.1 | 3.4 |
| 75 | 1.00 | 0.08 | 0.04 | 90.0 | 6.8 | 3.3 |
| 99 | 1.00 | 0.10 | 0.11 | 82.6 | 7.9 | 9.5 |
| 123 | 1.00 | 0.12 | 0.16 | 78.4 | 9.3 | 12.3 |
| 147 | 1.00 | 0.18 | 0.22 | 71.1 | 13.1 | 15.8 |
| 171 | 1.00 | 0.24 | 0.29 | 65.2 | 15.9 | 18.9 |
| 195 | 1.00 | 0.28 | 0.35 | 61.6 | 17.0 | 21.4 |
| 219 | 1.00 | 0.32 | 0.42 | 57.2 | 18.6 | 24.2 |
| 243 | 1.00 | 0.35 | 0.52 | 53.4 | 18.9 | 27.6 |
| 267 | 1.00 | 0.43 | 0.58 | 49.7 | 21.3 | 29.0 |
| 291 | 1.00 | 0.51 | 0.68 | 45.7 | 23.2 | 31.1 |
| 315 | 1.00 | 0.56 | 0.77 | 42.8 | 24.2 | 33.0 |
| 339 | 1.00 | 0.61 | 0.85 | 40.5 | 24.9 | 34.6 |
| 363 | 1.00 | 0.66 | 0.95 | 38.3 | 25.2 | 36.5 |
| 387 | 1.00 | 0.76 | 1.08 | 35.2 | 26.7 | 38.1 |
| 411 | 1.00 | 0.85 | 1.23 | 32.5 | 27.6 | 39.9 |
| 435 | 1.00 | 0.93 | 1.36 | 30.4 | 28.4 | 41.2 |
| 459 | 1.00 | 0.99 | 1.41 | 29.4 | 29.1 | 41.4 |
| 483 | 1.00 | 1.10 | 1.57 | 27.2 | 30.0 | 42.8 |
| 507 | 1.00 | 1.22 | 1.83 | 24.7 | 30.2 | 45.1 |
| 531 | 1.00 | 1.34 | 1.88 | 23.7 | 31.7 | 44.6 |
| 555 | 1.00 | 1.52 | 2.20 | 21.2 | 32.2 | 46.6 |
| 579 | 1.00 | 1.59 | 2.34 | 20.3 | 32.3 | 47.5 |
| 603 | 1.00 | 1.76 | 2.58 | 18.7 | 33.0 | 48.3 |
| 627 | 1.00 | 1.97 | 2.92 | 17.0 | 33.4 | 49.6 |
| 651 | 1.00 | 2.21 | 3.16 | 15.7 | 34.7 | 49.7 |
| 675 | 1.00 | 2.13 | 3.13 | 16.0 | 34.0 | 50.0 |
| 699 | 1.00 | 2.43 | 3.56 | 14.3 | 34.8 | 50.9 |
| 723 | 1.00 | 2.70 | 3.87 | 13.2 | 35.6 | 51.2 |
| 747 | 1.00 | 2.88 | 4.15 | 12.4 | 35.8 | 51.7 |
| 771 | 1.00 | 2.98 | 4.32 | 12.0 | 35.9 | 52.1 |
| 795 | 1.00 | 3.59 | 5.19 | 10.2 | 36.7 | 53.1 |
| 819 | 1.00 | 3.78 | 5.38 | 9.8 | 37.2 | 53.0 |
| 843 | 1.00 | 3.36 | 4.88 | 10.8 | 36.3 | 52.8 |
| 867 | 1.00 | 4.25 | 6.24 | 8.7 | 37.0 | 54.3 |
| 891 | 1.00 | 5.13 | 7.42 | 7.4 | 37.9 | 54.7 |
| 915 | 1.00 | 6.12 | 8.78 | 6.3 | 38.5 | 55.2 |
| 939 | 1.00 | 5.47 | 7.85 | 7.0 | 38.2 | 54.8 |
| 963 | 1.00 | 6.99 | 10.23 | 5.5 | 38.4 | 56.2 |
| 987 | 1.00 | 6.75 | 9.82 | 5.7 | 38.4 | 55.9 |
| 1011 | 1.00 | 7.67 | 10.89 | 5.1 | 39.2 | 55.7 |
| 1035 | 1.00 | 6.99 | 10.21 | 5.5 | 38.4 | 56.1 |
| 1059 | 1.00 | 6.96 | 9.74 | 5.6 | 39.3 | 55.0 |
| 1083 | 1.00 | 8.89 | 12.55 | 4.5 | 39.6 | 55.9 |
| 1107 | 1.00 | 10.62 | 14.97 | 3.8 | 39.9 | 56.3 |
| 1131 | 1.00 | 9.79 | 14.16 | 4.0 | 39.2 | 56.8 |
| 1155 | 1.00 | 14.57 | 21.05 | 2.7 | 39.8 | 57.5 |
| 1179 | 1.00 | 12.66 | 17.92 | 3.2 | 40.1 | 56.7 |
| 1203 | 1.00 | 16.53 | 23.12 | 2.5 | 40.7 | 56.9 |
| 1227 | 1.00 | 16.37 | 22.65 | 2.5 | 40.9 | 56.6 |
| 1251 | 1.00 | 16.80 | 23.88 | 2.4 | 40.3 | 57.3 |
| 1275 | 1.00 | 23.06 | 33.48 | 1.7 | 40.1 | 58.2 |
| 1299 | 1.00 | 20.64 | 28.65 | 2.0 | 41.0 | 57.0 |
| 1323 | 1.00 | 41.69 | 61.14 | 1.0 | 40.2 | 58.9 |
| 1347 | 1.00 | 22.65 | 32.25 | 1.8 | 40.5 | 57.7 |
| 1371 | 1.00 | 29.39 | 42.34 | 1.4 | 40.4 | 58.2 |
| 1395 | 1.00 | 28.58 | 40.41 | 1.4 | 40.8 | 57.7 |
| 1419 | 1.00 | 39.00 | 56.55 | 1.0 | 40.4 | 58.6 |
| 1443 | 1.00 | 38.19 | 54.13 | 1.1 | 40.9 | 58.0 |
| 1467 | 1.00 | 34.78 | 50.50 | 1.2 | 40.3 | 58.5 |
| *1635* | *1.00* | *382.68* | *547.06* | *0.1* | *41.1* | *58.8* |

**Rearrangement Reaction Profiles for 22a (343-373 K)**

The reaction profile (relative percentage conversion vs time) was plotted using the NMR data above on Excel.

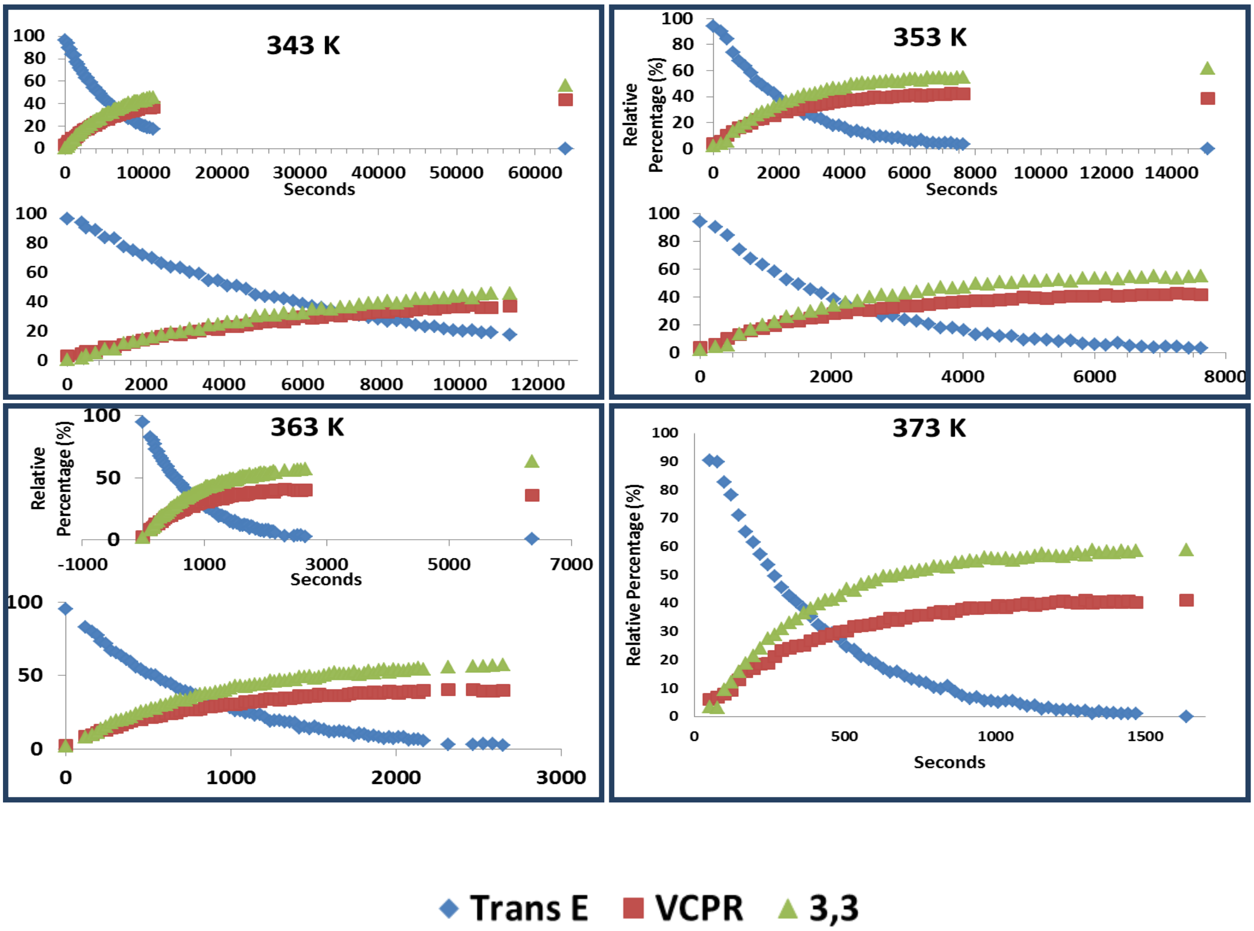


**Figure S35:** Reaction profiles for the rearrangement of **22a** at 343-373 K.

## NMR Simulations

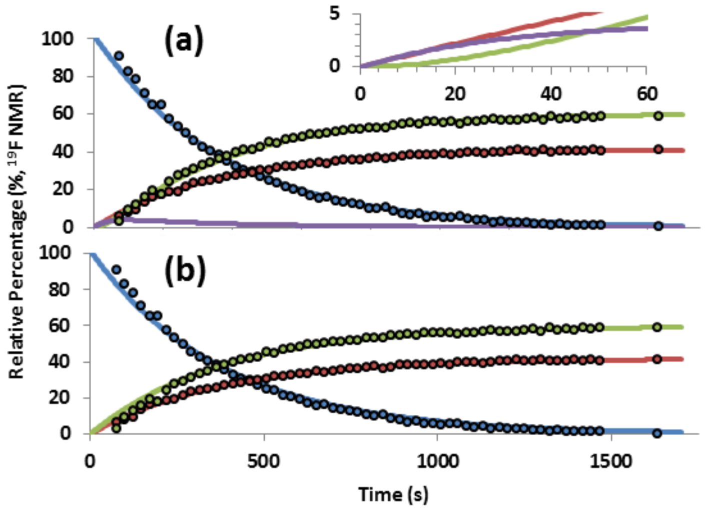


**Figure S36:** Experimental (points) and simulated (lines) concentration/time profile for thermolysis (373 K) of **22a**; a) simulation Model A takes into account *cis*-**48**; insert shows the first simulated 30 seconds of the rearrangement b) Model B utilising a steady state approximation for cis-**48** (**blue** = *trans*-**22a**, **green** = **44**, **red** = **39**, **purple** = *cis*-**48**).

The experimental concentration/time profile could be simulated successfully as far as experimental end points using numerical integration software[S37] based on two kinetic models expressed in **Scheme S7**.

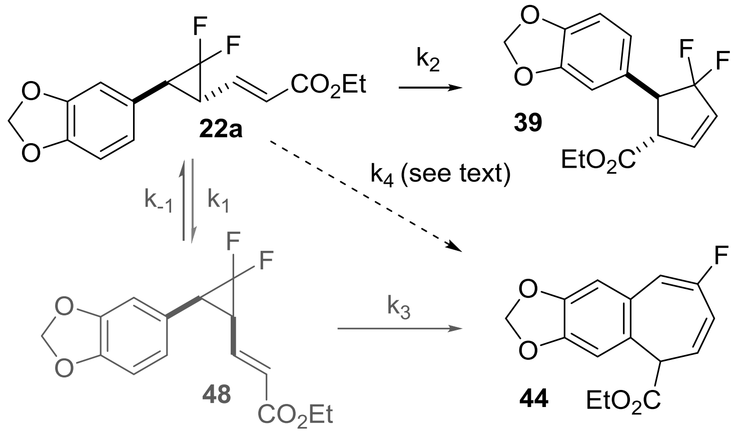


**Scheme S8:** Kinetic models used in the simulation of parallel VCPR and [3,3]-pathways.

Model A used successfully with phenyl **1a**, takes into account the equilibrium set up between *cis*-**48** and *trans*-**22a** due to cyclopropane stereoisomerisation and the subsequent first order rearrangement through to diene **44** and cyclopentene **39**, respectively. Simulated data using this model showed formation of cis-**48**, rising to a maximum at about 3% in the

reaction mixture after 30 seconds before slowly decaying (**Figure S36a**). These values are below the lower limit of experimental detection and explain why **48** was not observed. Deconvolution of rate constants predicted that rearrangement of **22a** should be selective for the [3,3]-pathway, with a rate approximately 40 times faster than VCPR (**Table S4**). This does not match experimental findings and may be due to an overestimation in rates involving *cis*-**XX** due to the lack of experimental data points to fit profiles to.

This issue was resolved by treating both rearrangement pathways as first order with respect to *trans*-**22a** (Model B), $k_4$ represents an overall rate for the conversion of **22a** to **48** and incorporates the cyclopropane stereoisomerisation and [3,3]-rearrangement rates associated with **48**. Again, simulated data showed good correlation with experimental values (**Figure S36b**) and this time the rate constants favoured the [3,3] pathway only slightly (1.5 times faster than VCPR), consistent with experimental conversions of 59% and 41% to diene **44** and cyclopentene **39**, respectively. Furthermore, the much higher rate for VCPR from **22a** (11.5 x $10^{-4}$ $s^{-1}$) than from phenyl **1a** (1.6 x $10^{-4}$ $s^{-1}$), matched the predicted differences in calculated activation energies.

**Table S4:** Rate Constants extracted from reaction simulations.

| **Rate** ($\mathbf{10^{-4}\ s^{-1}}$) | **Model** A | **Model** B |
|---|---|---|
| $k_2$ | 11.2 | 11.1 |
| $K_1$ | 21.8 | - |
| $k_{-1}$ | 153.2 | - |
| $k_3$ | 439.4 | - |
| $k_4$ | - | 15.8 |
| $k_3/k_2$ | 39.2 | - |
| $k_4/k_2$ | - | 1.4 |

**Simulation Procedure:** The NMR concentration/time data obtained for **22a** (including a measured endpoint) was imported into Berkeley Madonna software[S37] from text files and simulated using the two methods discussed below:

```
Model A

STARTTIME = 0
STOPTIME=43200
DT = 0.05

A0=100 {start concentration of trans cyclopropane}
Init A=A0
B0=0 {start concentration of cis cyclopropane}
Init B=B0
C0=0 {start concentration of cyclopentene}
Init C=C0
D0=0 {start concentration of benzoheptadiene}
Init D=D0

k1=0.0001 {k1 is isomerisation to cis}
k_1=0.0001 {k_1 is isomerisation to trans}
k2=0.0001 {k2 is the VCPR}
k3=0.0001 {k3 is the 3,3}

d/dt (A)=-k1*A-k2*A+k_1*B
d/dt (B)=-k_1*B-k3*B+k1*A
d/dt (C)=k2*A
d/dt (D)=k3*B

LIMIT A>=0
LIMIT B>=0
LIMIT C>=0
LIMIT D>=0
```

The extracted rate data (343-373 K) from Model A was not consistent with experimental observation of VCPR and [3,3] pathways having similar rates (**Table S5**).

**Table S5**: Rate data based on Model A.

| **Temp (K)** | **$k_1$ ($10^{-4}$ $s^{-1}$)** | **$k_{-1}$ ($10^{-4}$ $s^{-1}$)** | **$k_2$ ($10^{-4}$ $s^{-1}$)** | **$k_3$ ($10^{-4}$ $s^{-1}$)** |
|---|---|---|---|---|
| 343 | 5.243 | 440.186 | 0.734 | 88.296 |
| 353 | 2.667 | 0.193 | 2.028 | 114.035 |
| 363 | 11.061 | 165.75 | 5.466 | 357.913 |
| 373 | 21.794 | 153.183 | 11.246 | 439.420 |

**Model B**

```
STARTTIME = 0
STOPTIME=43200
DT = 0.05

A0=100 {start concentration of trans cyclopropane}
Init A=A0
C0=0 {start concentration of cyclopentene}
Init C=C0
D0=0 {start concentration of benzoheptadiene}
Init D=D0

k2=0.0001 {k2 is the VCPR}
k4=0.0001 {k3 is the 3,3}

d/dt (A)=- -k2*A-k4*A
d/dt (C)=k2*A
d/dt (D)=k4*A

LIMIT A>=0
LIMIT C>=0
LIMIT D>=0
```

The rate data extracted from **Model B** matched experimental results more closely, giving similar rate values for VCPR and [3,3]-rearrangement pathways (**Table S6**).

**Table S6:** Rate data extracted from Model B.

| Temp (K) | $k_2$ ($10^{-4}$ $s^{-1}$) | $k_4$ ($10^{-4}$ $s^{-1}$) |
|---|---|---|
| 343 | 0.7338 | 0.8725 |
| 353 | 2.0242 | 2.6320 |
| 363 | 5.4558 | 7.4840 |
| 373 | 11.0977 | 15.7878 |

**Arrhenius Plot for Piperonyl 22a**

Rate data from **Table S4** was changed into the desired Arrhenius parameters (**Table S7**).

**Table S7:** Arrhenius Data

| Temperature (K) | 1/T | $k_2$ ($10^{-4}$ $s^{-1}$) | Ln $k_2$ | $k_4$ ($10^{-4}$ $s^{-1}$) | Ln $k_4$ |
|---|---|---|---|---|---|
| 344.1 | 0.002906132 | 0.7338 | -9.51986 | 0.8725 | -9.34673 |
| 354.2 | 0.002823264 | 2.0242 | -8.50517 | 2.632 | -8.2426 |
| 364.2 | 0.002745744 | 5.4558 | -7.51366 | 7.484 | -7.19757 |
| 374.2 | 0.002672368 | 11.0977 | -6.8036 | 15.7878 | -6.4511 |

From Arrhenius equation **1**, a plot of Ln $k_2$ or Ln $k_4$ vs 1/T was generated on Excel and a line fitted using the trendline function (**Figure S37**). The equation of the line was calculated using the *LINEST* statistical function and the gradient used to calculate $E_a$. The errors in the values were estimated by plotting two lines which would result from the maximum errors (± 5%) in the outermost points and averaging the gradient to determine the $E_a$.

$$\ln \mathrm{k} = \frac{-E_a}{\mathrm{R}}\frac{1}{\mathrm{T}} + \ln \mathrm{A} \qquad (\mathbf{1})$$

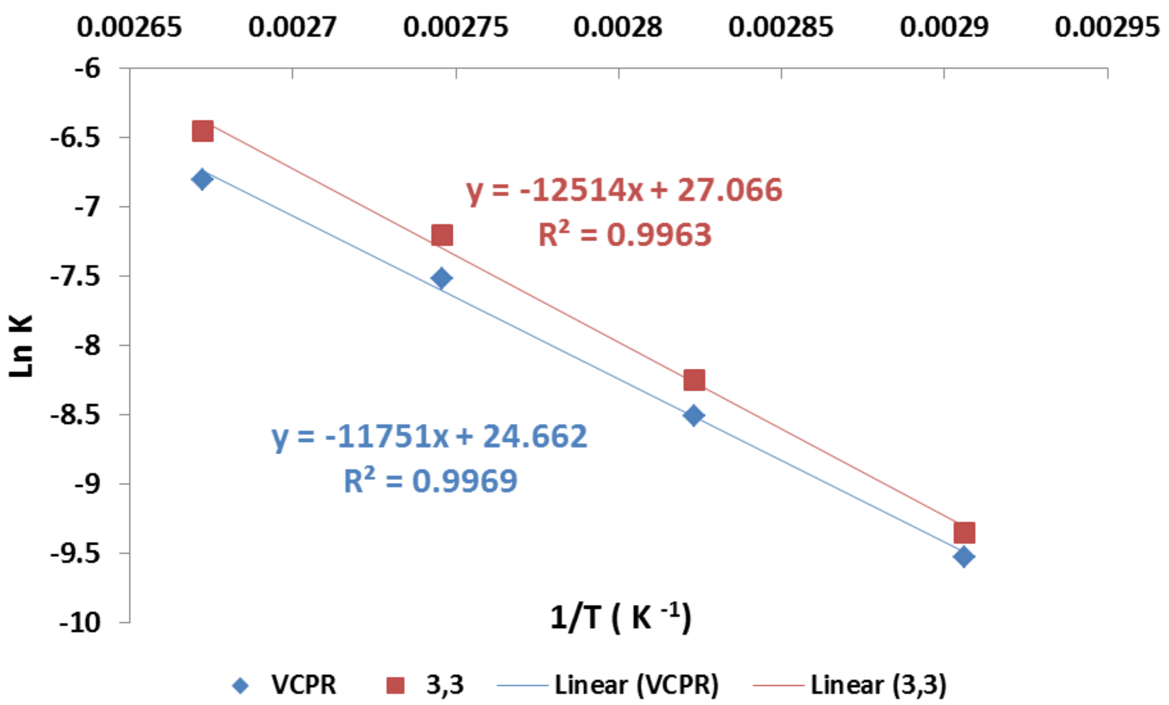


**Figure S37:** Combined Arrhenius Plots for VCPR and [3,3]-rearrangement of **22a.**

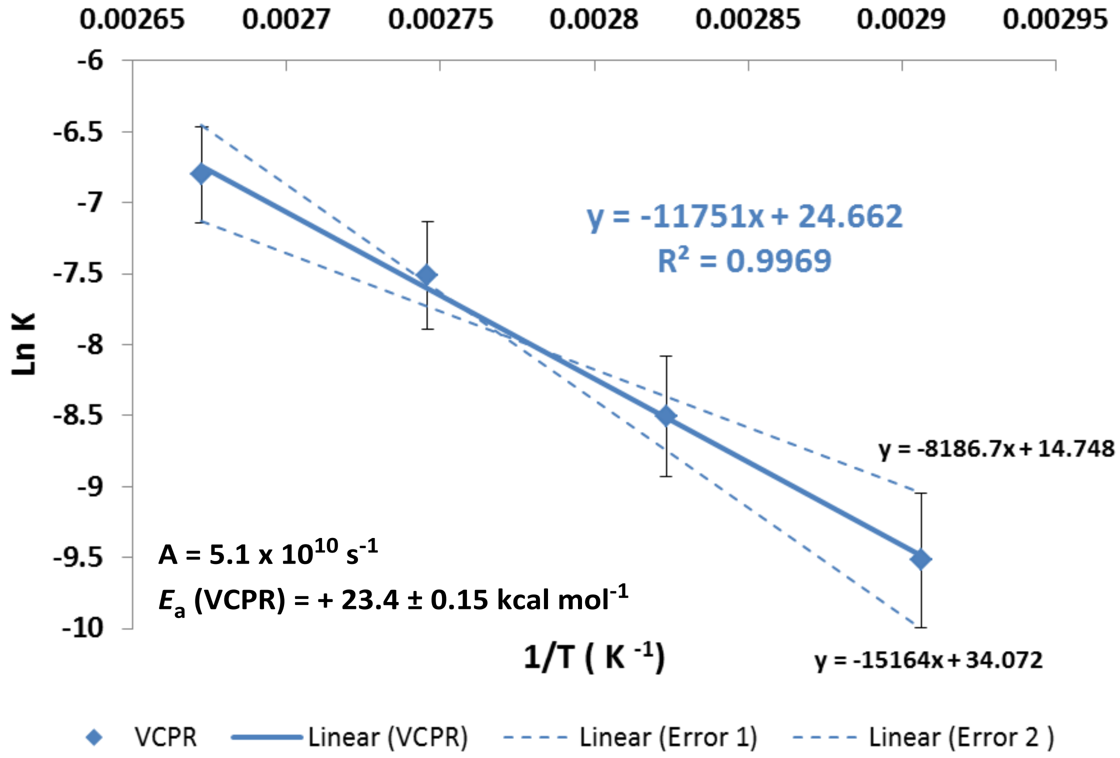


**Figure S38:** Arrhenius plot for VCPR of **22a** with error estimations.

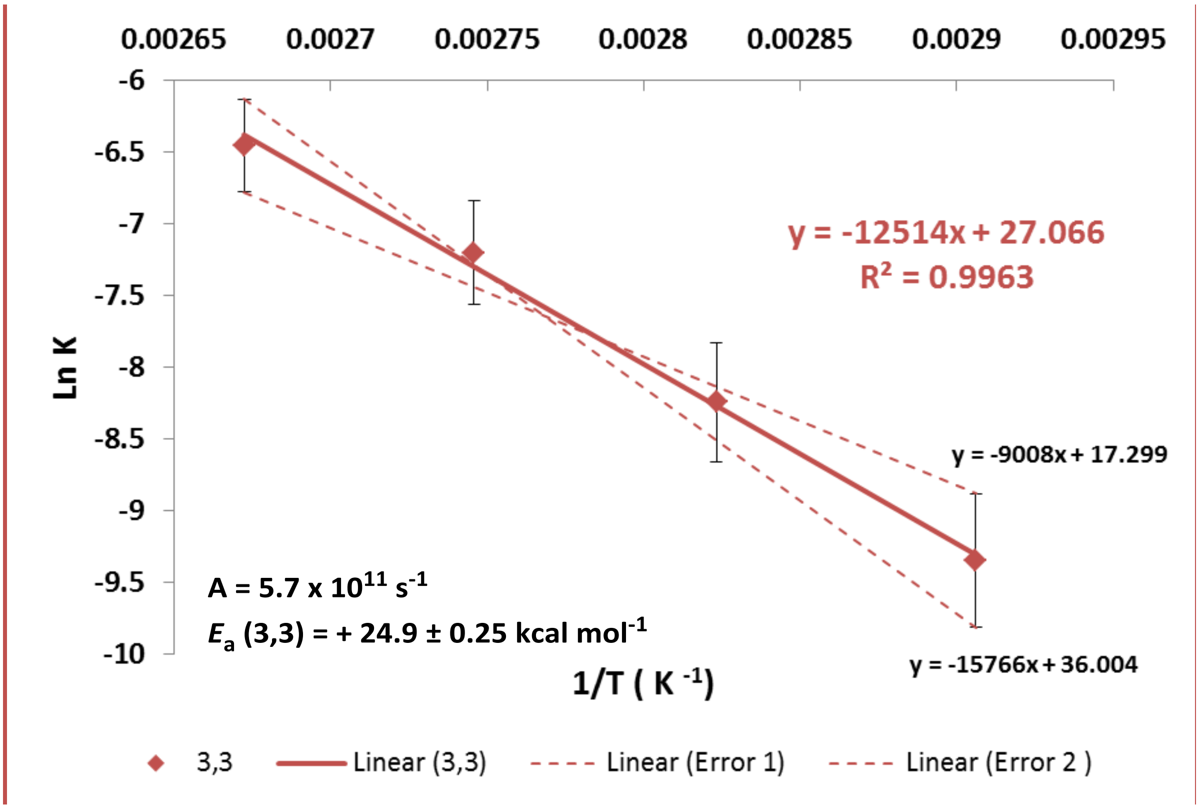


**Figure S39:** Arrhenius plot for [3,3]-rearrangement of **22a** with error estimations.

**Identification the Most Appropriate Method for Electronic Structure Calculations**

In order to assess the accuracy of the electronic structure calculations efficiently, all experimental activation energies were recalculated to 298 K using the Arrhenius data and methods described by Maskill.[S38] We previously reported that for VCPRs, changes to the basis set either had detrimental effects (with UB3LYP, UM06-2X and UB97-D functionals) or gave slightly more accurate values of $\Delta G^{\ddagger}$ when the diffuse function was introduced (6-31+G*) with the UM05-2X functional. Increasing polarisation and diffuse functions (6-31+G**) gave no improvement in the agreement between experimental and calculated values for a benchmarking set of VCPRs; these trends were also followed for piperonyl **22a** (see S182 for more details). Initial methodology screening was carried out using Spartan'10 software due to the ease of local implementation. All activation energies were then recalculated in Gaussian'09, since the software dealt with the open shell singlet character of the VCPR better giving $\langle S^2 \rangle$ values closer to 0.75, some additional valuable methods were available, and the energies obtained would then be more comparable with the literature. Investigating the barrier height for the rearrangement of VCP **9f** to **TS1f** (models representing piperonyl **22a**), gave results comparable to those observed with phenyl **9j**. The M05-2X functional[S39] with the 6-31+G* basis set still proved to be the most accurate methodology, with a $\Delta\Delta G^{\ddagger}$ = 0.2 kcal mol$^{-1}$ (**Figure S40**). Truhlar and co-workers reported that the M06-2X functional, with a similar HF-exchange (56% for M05-2X and 58% M06-2X), was slightly more accurate overall in assessing kinetic parameters than M05-2X.[S40] However in our new system (VCP-**9f**) this was not the case, with a higher (4.5 kcal mol$^{-1}$) overestimation by the M06-2X functional with the same basis set.

During the review of the manuscript of our previous publication, it was suggested that the different amounts of HF-exchange used in each method could have some role in determining the accuracy of the calculated free energies of activation for the VCPR. The M06 functional, with a lower HF-exchange of 27%, gave the closest agreement with experimental values when the 6-31G* basis set was used ($\Delta\Delta G^{\ddagger}$ = 0.7 kcal mol$^{-1}$). Electronic structure calculations using M05/6-31G* methodology were less accurate ($\Delta\Delta G^{\ddagger}$ = 2.6 kcal mol$^{-1}$), even though this functional has a similar HF-exchange value (28%) to the M06 functional. However, we observed little difference between the M05-2X and M06 functionals when the VCPR barrier heights were calculated with Gaussian'09 for piperonyl **9f**, simple precursor **8a** and phenyl **9j**. Furthermore, for the VCPR of non-fluoro **8b**, M06/6-

31+G* calculations were only within 1.6 kcal mol$^{-1}$ (underestimate) whereas M05-2X/6-31+G* methods gave ΔΔG$^{‡}$ values within 0.8 kcal mol$^{-1}$ (overestimate, see S183 for more details).

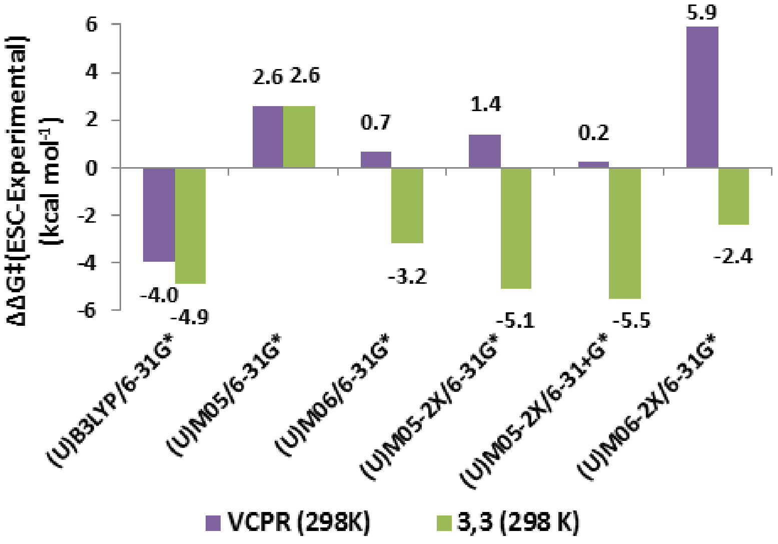


**Figure S40**: Differences between experimental ΔG$^{‡}$ (298 K, re-calculated from activation parameters) and ΔG$^{‡}$ from electronic structure calculations (298 K, gas phase, Spartan'10) plotted as ΔΔG$^{‡}$ (ΔG$^{‡}$(ESC)-ΔG$^{‡}$(experimental), kcal mol$^{-1}$) for the VCPR and [3,3]-rearrangement of VCP **9f** and **48**, respectively. Expected error associated with calculated data is ± 0.5 kcal mol$^{-1}$.

Other DFT methods either underestimated experimental barrier heights when 20% HF-exchange was used (B3LYP), or overestimated (+1.8 kcal mol$^{-1}$) when a hybrid exchange-correlation was used with 19% HF-exchange at short range and 65% at long range (CAM-B3LYP,[S41] (**Figure S41**)). These results suggest that HF-exchange alone does not determine the accuracy of VCPR activation energies.

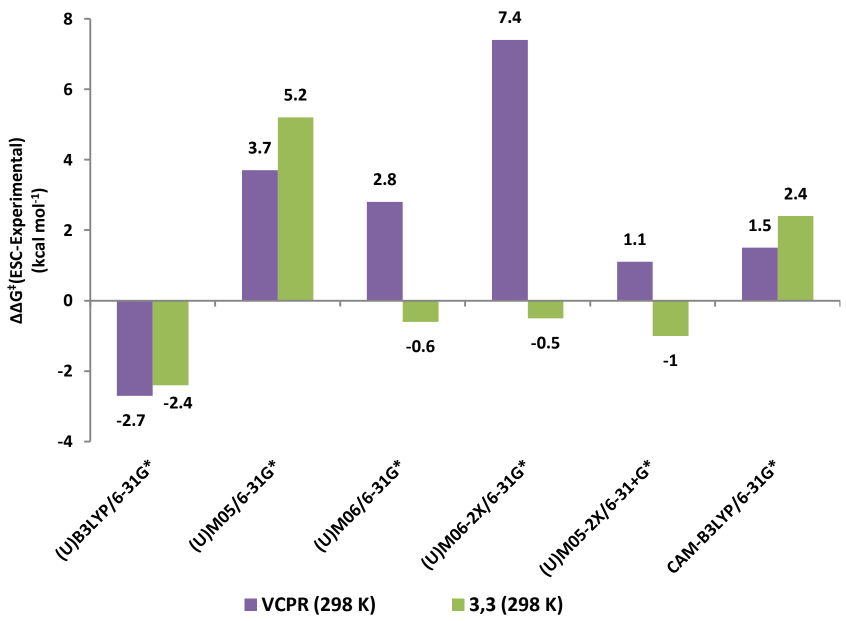


**Figure S41**: Differences between experimental ΔG$^{‡}$ (298 K, re-calculated from activation parameters) and ΔG$^{‡}$ from electronic structure calculations (298 K, gas phase, Gaussian'09) plotted as ΔΔG$^{‡}$ (ΔG$^{‡}$(ESC)-ΔG$^{‡}$(experimental), kcal mol$^{-1}$) for the VCPR and [3,3]-rearrangement of VCP **9f** and **48**, respectively. Expected error associated with calculated data is ± 0.5 kcal mol$^{-1}$.

One difference which was observed between phenyl and piperonyl substituents was the change in dipole moment calculated from precursor to transition state; **9j** showed a slight decrease in calculated polarity during VCPR and [3,3]-rearrangement (change in dipole = -0.39 and -0.22 Debyes, respectively) whereas **9f** showed a slight increase (change in dipole = +1.2 and +0.95 Debyes, respectively, UB3LYP/6-31G*, Gaussian'09, gas phase, 298 K). The calculated activation energies for the two rearrangement pathways from 9f were assessed using M05-2X/6-31+G* method with different solvation parameters which had varying dielectric constants (range of ε = 2.37-35.7), but only a slight decrease in barrier height for the [3,3]-rearrangement in acetonitrile compared with gas phase was predicted (ΔG$^{‡}$Gas-ΔG$^{‡}$MeCN = -0.9 kcal mol$^{-1}$). Maas reported that the experimental rate for the [3,3]-rearrangement of non-fluorinated 1-aryl-2-vinylcyclopropanes was more than 8 times faster in acetonitrile than benzene, but VCPR rates were not affected by reaction solvent.[S42] VCP **22a** showed no significant change in rearrangement outcome when heated in either [D3]acetonitrile (52% (**44**([3,3]) and 48% (**39**(VCPR)) conversion, determined by $^{19}$F NMR) or [D8]toluene (58% (**44**([3,3]) and 42% (**39**(VCPR)) conversion, determined by $^{19}$F NMR).

Therefore, no solvation methods were applied and all electronic structure calculations were carried out in the gas phase (see S184 for more details).

Previously, only a concerted closed-shell transition state was investigated for the [3,3]-rearrangement of *cis*-**1b**. This was consistent with Özkan and Zora's study[S43] of the divinylcyclopropane rearrangement; despite running spin-unrestricted calculations, no low energy diradical or triplet intermediates or transition states were found. We used our previously optimised triplet intermediate of ring opened phenyl VCP **9j** as a starting point to search for alternative triplet or open shell singlet pathways for the [3,3]-rearrangement ((U)B3LYP/6-31G* (gas phase, 298 K), Spartan'10). Two triplet transition states connected the alkenoate radical with alternative *ortho*-carbon centred radicals on the aromatic ring; both had uncompetitive calculated activation energies of 53.6 kcal $mol^{-1}$ and 59.2 kcal $mol^{-1}$. Only one of these optimised as a diradical, but had a much higher barrier for rearrangement (37.8 kcal $mol^{-1}$) than the closed shell pathway (27.8 kcal $mol^{-1}$). For subsequent computational methodology screening, the [3,3]-rearrangement was treated using closed shell methodology (see S187 for more details).

Irrespective of the basis set used, the M05-2X functional was found to be the least accurate of those examined for calculating the free energy of activation of **48** to **TS6a** (6-31G*, $\Delta\Delta G^{\ddagger}$ = - 5.1 kcal $mol^{-1}$ and 6-31+G*, $\Delta\Delta G^{\ddagger}$ = -5.5 kcal $mol^{-1}$). Instead, the M06-2X/6-31G* combination was found to be the best at predicting barrier height with a $\Delta\Delta G^{\ddagger}$ = -2.4 kcal $mol^{-1}$ (**Figure S40**). This is opposite to what was observed for the VCPR and gives a strong indication of the functionals' differing abilities in dealing with open shell singlet character or the loss of aromaticity at transition states. The (U)B3LYP/6-31G* method gave a similar underestimation in barrier height for both pathways ($\Delta\Delta G^{\ddagger}$ values were within error of ± 0.9 kcal $mol^{-1}$) but (U)M05/6-31G* gave exactly the same overestimate of 2.6 kcal $mol^{-1}$. If required, these two combinations of functions and basis sets provide a universal method for assessing both rearrangement pathways in Spartan'10.

Electronic structure calculations for VCP **9f** which were carried out in Gaussian'09 dealt with the open shell singlet character of the VCPR better ($S^2$ value closer to 0.75) than Spartan'10, resulting in more accurate barrier heights; M05/6-31G* was the key exception, with higher $\Delta\Delta G^{\ddagger}$ values for both VCPR and [3,3]-pathways (+3.7 kcal $mol^{-1}$ and +5.2 kcal $mol^{-1}$, respectively). DFT methodology using B3LYP/6-31G* still gave consistent underestimation in barrier height for both pathways; $\Delta\Delta G^{\ddagger}$ values were within 0.3 kcal $mol^{-1}$ of each other

(**Figure S41**). Of the Minnesota functionals, the M06-2X/6-31G* method remained the most accurate for the [3,3]-rearrangement ($\Delta\Delta G^{\ddagger}$ = -0.5 kcal mol$^{-1}$) and M05-2X/6-31+G* for the VCPR ($\Delta\Delta G^{\ddagger}$ = +1.1 kcal mol$^{-1}$).

We looked to explore the effectiveness of the B3LYP/6-31G* and M06-2X/6-31G* calculations of barrier heights for divinylcyclopropane[S11,S44] and heteroarene-vinylcyclopropane rearrangements[S45] reported in the literature but the reported experimental errors were so big that it made it impossible to distinguish between accurate and inaccurate calculated barriers (see S189 for more details).

An absolutely critical event in the [3,3]-rearrangement is the temporary disruption of aromaticity; the enthalpic consequences of this event must be handled well if any method is to provide realistic free energies of activation. The isodesmic reactions represented in **Scheme S9** were used to assess how well the B3LYP and M06-2X methods dealt with this temporary dearomatisation in the transition state.

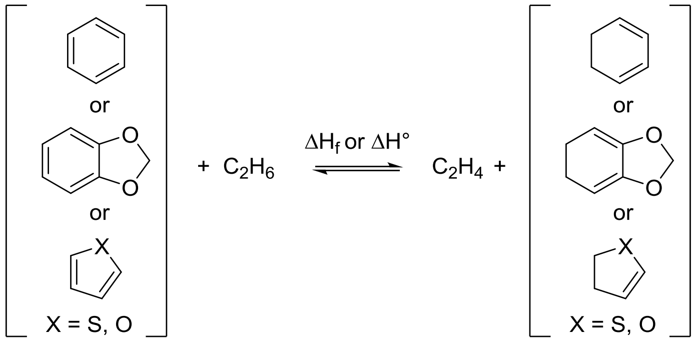


**Scheme S9**: Isodesmic reactions used to model the disruption of aromaticity.

We therefore benchmarked the DFT calculations against well-known thermochemical recipes using these isodesmic reactions. There was little difference between the calculated heats of formation for either T1[S46] or G3(MP2)[S47] thermochemical recipes (within computational error of ±0.5 kcal mol$^{-1}$), but when compared with experimental values,[S48,S49] T1 was more accurate for ethane and ethylene whereas G3(MP2) was slightly more accurate for aromatic compounds (**Figure S42**).

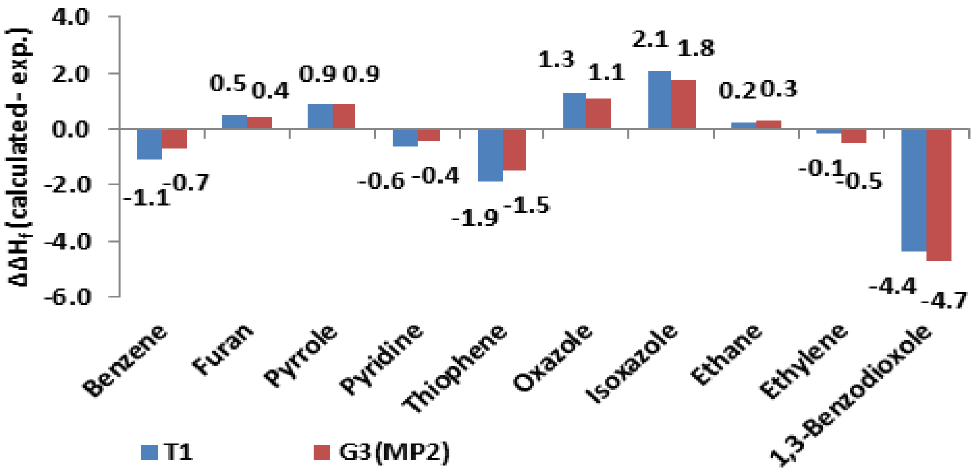


**Figure S40**: Differences between calculated and experimental heats of formation (Spartan'10, values in kcal $mol^{-1}$).

The average error associated with the calculated heats of formation for benzene, furan, pyrrole, pyridine, thiophene, oxazole and isoxaxole were ± 1.21 kcal $mol^{-1}$ and ± 0.95 kcal $mol^{-1}$ for T1 and G3(MP2), respectively. Calculated data for 1,3-benzodioxole had an underestimation greater than 4.0 kcal $mol^{-1}$ for both methods when compared with experimental values.[S50]

The G3(MP2) values were used to assess the accuracy of the calculated enthalpy change for the dearomatisation of benzene, benzodioxole, furan and thiophene using either B3LYP/6-31G* or M06-2X/6-31G* methodology (**Figure S43**, see S193, S194 for more details). The B3LYP method had an average overprediction in enthalpy of 5.3 kcal $mol^{-1}$ for benzene, furan, thiophene and 1,3-benzodioxole, and M06-2X consistently over-estimated the enthalpic cost of dearomatisation, ranging from 3.6 kcal $mol^{-1}$ to 6.4 kcal $mol^{-1}$ (total average error of 4.8 kcal $mol^{-1}$).

This thermochemical analysis suggests that the two most desirable methods for assessing the [3,3]-rearrangement do not deal with the loss of aromaticity at the transition state well. This is important since M06-2X/6-31G* calculations were perceived to be the most accurate, but are likely to over-predict the free energy ($\Delta G^{\ddagger}$) for rearrangements involving benzene, furan, thiophene and 1,3-benzodioxole.

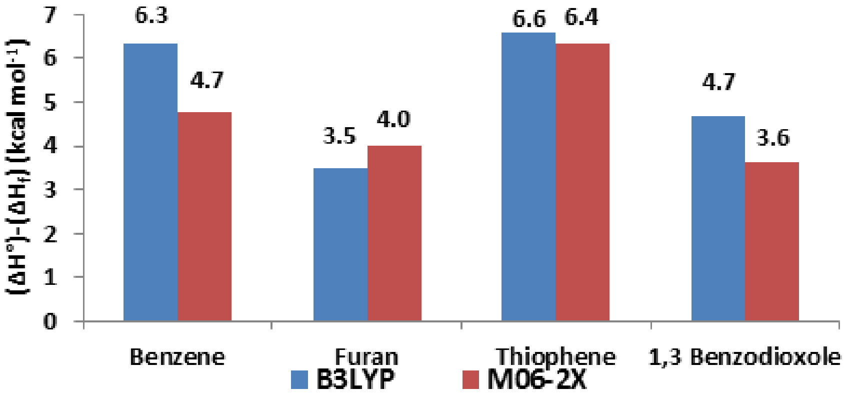


**Figure S43**: Difference in change in enthalpy (ΔH°) calculated using B3LYP or M06-2X (6-31G*) methods and G3(MP2) heats of formation (ΔHf) for the isodesmic reactions represented in Scheme 10 (gas phase, 298 K, Spartan'10, units are in kcal $mol^{-1}$).

## Basis Set Effect on 9f VCPR Activation Energies

M05-2X functional was the most accurate method for calculating the free energies of activation for the VCPR of phenyl-**9j** and piperonyl-**9f**. No benefit was found from increased basis sets higher than 6-31+G* for **9f** consistent with our previous studies[S20] (**Table S8** and **Figure S44**).

**Table S8:** Basis Set Effect on Accuracy of Calculated VCPR Free Energies of Activation for **9f**

| **Functional** | **Basis Set** | $\Delta G^{‡[a]}$ | $\Delta\Delta G^{‡[b]}$ |
|---|---|---|---|
| M05-2X | 6-31G* | 29.80 | 2.80 |
| M05-2X | 6-31+G* | 28.10 | 1.10 |
| M05-2X | 6-311G* | 30.30 | 3.30 |
| M05-2X | 6-311+G** | 28.30 | 1.30 |

[a] Gaussian'09, gas phase, 298 K. [b] Difference in calculated free energy of activation $\Delta G^{‡}$ and calculated activation energy ($E_a$); $\Delta\Delta G^{‡}$ = (ΔG)-($E_a$). All units are in kcal $mol^{-1}$.

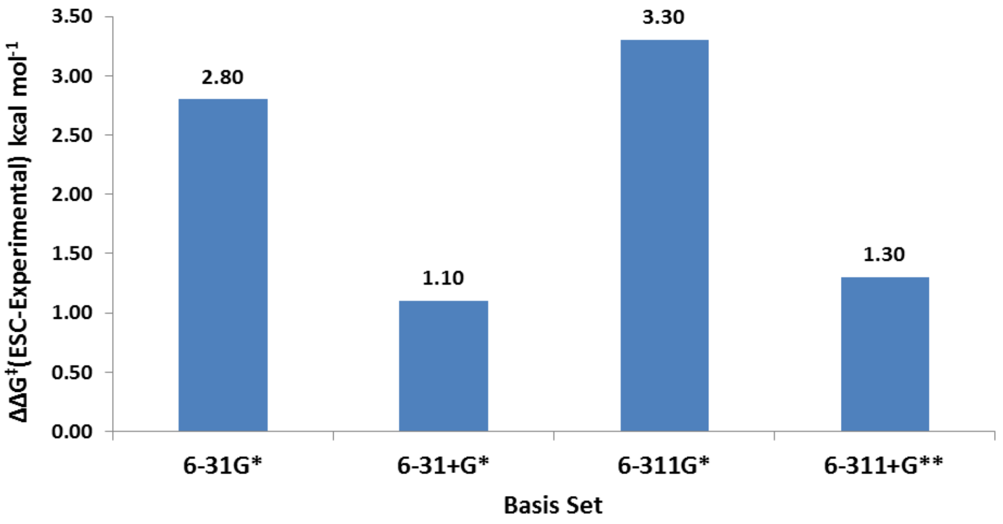


**Figure S41**: Comparison of $\Delta\Delta G^{\ddagger}$ values and basis set effects (Gaussian'09, M05-2X, gas phase, 298 K, Energies quoted in kcal mol$^{-1}$).

## Method Screening for VCPR

The calculated activation energies for the VCPR of simple VCP **8b** and difluoro-VCPs, phenyl **9j** and piperonyl **9f**, were assessed showing that the M05-2X/6-31+G* method was the most accurate for all three compounds (**Table S9** and **Figure S45**).

**Table S9:** Methodology Screening for VCPR

| Method | $\Delta\Delta G^{\ddagger}$ (ESC – Exerimental, kcal mol$^{-1}$)[a] | | |
|---|---|---|---|
| | 8b [b] | 9j[c] | 9f[d] |
| UB3LYP/6-31G* | -2.3 | -2 | -2.7 |
| UCAM-B3LYP/6-31G* | - | - | 1.5 |
| UM05/6-31G* | 2.0 | 3.3 | 3.7 |
| UM06/6-31G* | 1.4 | 2.4 | 2.8 |
| UM05-2X/6-31G* | 1.6 | 2.5 | 2.8 |
| UM05-2X/6-31+G* | 0.8 | 1.2 | 1.1 |
| UM06/6-31+G* | -1.6 | - | - |

[a] Gaussian'09, gas phase, 298 K. [b] Experimental activation energy corrected to 298 K from literature data.[S51] [c] Experimental data reported in previous publication.[S20] [d] This work.

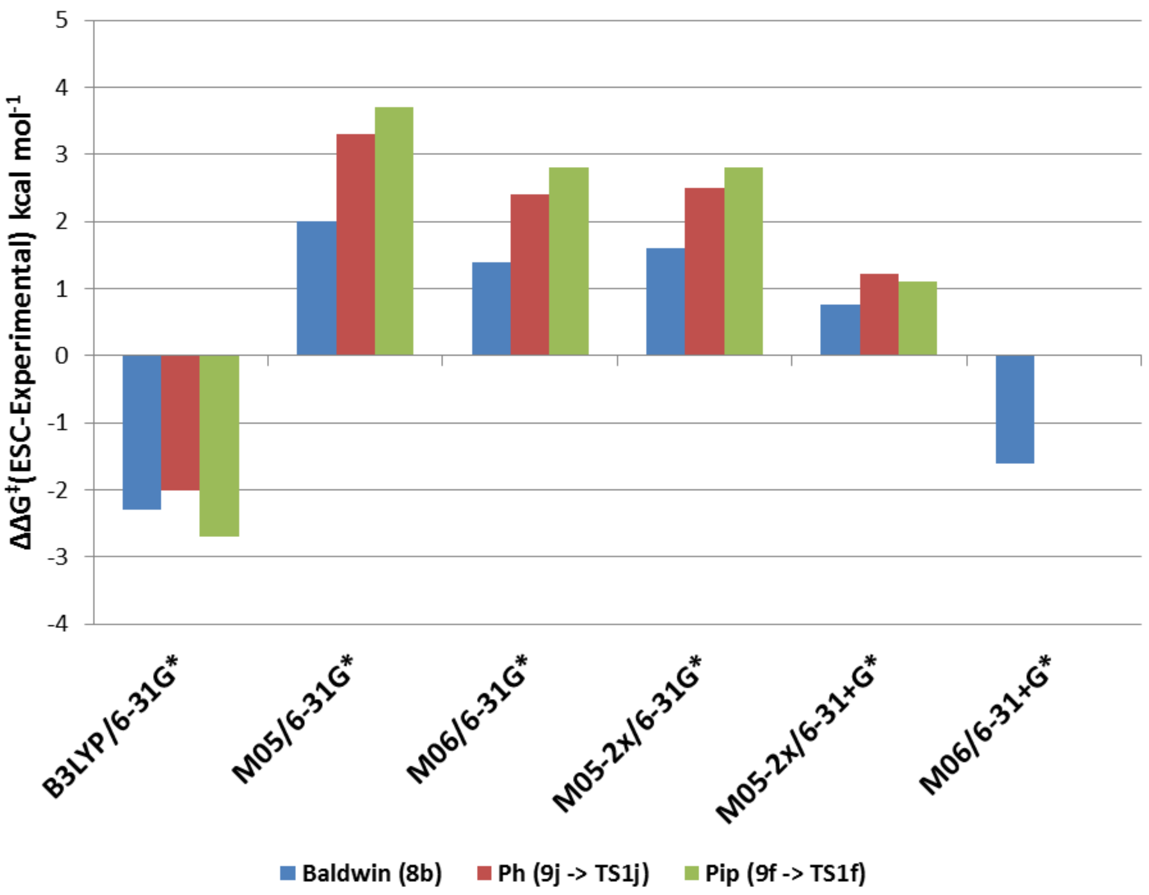


**Figure S42:** Computational methodology screening for VCPR.

**Assessment of Solvent Effects**

The difference in dipole between the VCPR and aromatic-vinylcyclopropane rearrangement transition states for the rearrangement of piperonyl-VCP precursors (*trans*-**9f** and *cis*-**9f**, respectively) was used to assess the potential effect solvent would have on electronic structure calculations (**Table S10**).

**Table S10**: Solvation Effect on VCPR and [3,3]-Rearrangement Dipole Moments

| Solvent | Solvent Properties | | Difference in Dipole Moment (Debyes)[c] | |
|---|---|---|---|---|
| | Dielectric Constant[a] | Dipole Moment (Debyes)[b] | 9f -> TS1f | 48 -> TS6a |
| Gas Phase (n.a.) | n.a. | n.a. | 0.11 | 0.74 |
| Toluene | 2.37 | 0.36 | 0.30 | 0.74 |
| Diphenyl Ether | 3.73 | 1.30 | 0.63 | 0.73 |

| | | | | |
|---|---|---|---|---|
| 1-Pentanol | 15.13 | 1.70 | 0.50 | 1.06 |
| Acetonitrile | 35.70 | 3.92 | 0.53 | 1.06 |

[a] Data reported in Gaussian'09 user's reference.[S52] [b] Data reported by National Institute of Standards and Technology (NIST).[S53] [c] Gaussian'09, (U)M05-2X/6-31+G*, 298 K, solvation parameterised using Self-Consistent Reaction Field (SCRF)[S54] using the Conductor-like Polarisable Continuum Model (CPCM).[S55]

Due to the slightly higher dipole moment for the [3,3]-pathway, more polar solvents (1-pentanol, acetonitrile) gave lower $\Delta G^{\ddagger}$ values compared with gas phase or less polar solvents (toluene, diphenyl ether); approximately 1.0 kcal mol$^{-1}$ lower energy barrier (**Table S11** and **Figure S46**).

**Table S11:** Solvent Effect on Rearrangement Free Energies of Activation ($\Delta G^{\ddagger}$)

| **Solvation** | **$\Delta G^{\ddagger}$ ((U)M05-2X/6-31+G*, 298 K, Gaussian'09)** | | |
|---|---|---|---|
| | **VCPR** | **[3,3]** | **$\Delta\Delta G^{\ddagger}$ ($\Delta G^{\ddagger}_{VCPR}$-$\Delta G^{\ddagger}_{[3,3]}$)** |
| Gas Phase | 28.1 | 25.3 | 2.8 |
| Toluene | 28.2 | 25.5 | 2.7 |
| Diphenylether | 28.3 | 25.7 | 2.6 |
| 1-Pentanol | 28.5 | 24.4 | 4.1 |
| Acetonitrile | 28.6 | 24.4 | 4.2 |

All units are in kcal mol$^{-1}$.

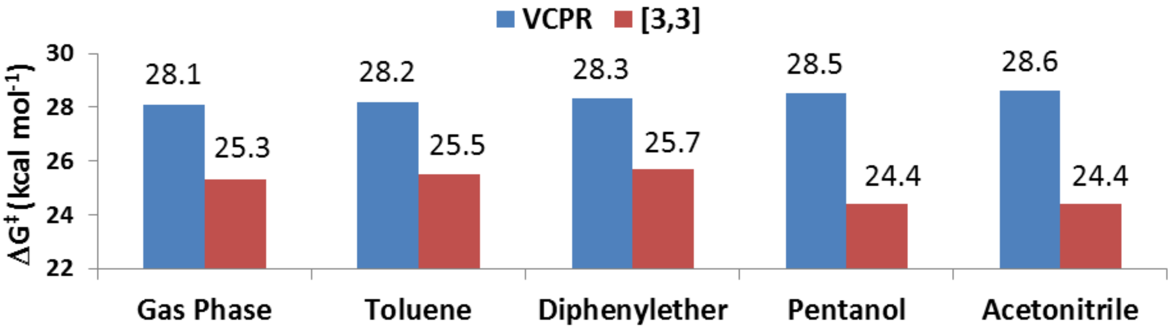


**Figure S43**: Solvent effect on calculated $\Delta G^{\ddagger}$ values for the VCPR of **9f** and [3,3]-rerrangement of **48**.

The effect of solvent was also investigated experimentally: A solution of piperonyl-**22a** (19.3 mg, 0.33 mmol) in 0.44 M [D3]acetonitrile (0.75 mL) or [D8]toluene (0.75 mL) was heated in an oil bath at 70 °C for 17 hours. Crude $^{19}$F NMR analysis of the resulting mixture was used to determine relative conversion of rearrangement products (**Table S12**), showing little difference in reaction selectivity between solvents (**Figure S47**).

**Table S12:** Solvent Effect on Thermal Rearrangement of **22a**.

| **Solvent** | **Average $^{19}$F NMR Integration** | | **Relative Percentage (%)[a]** | |
|---|---|---|---|---|
| | **VCPR (39)** | **[3,3] (44)** | **VCPR (39)** | **[3,3] (44)** |
| [D3]acetonitrile | 0.9584 | 1.0281 | 48 | 52 |
| [D6]toluene | 0.9744 | 1.3721 | 42 | 58 |

[a] Assumed NMR integration error of ± 5 %.

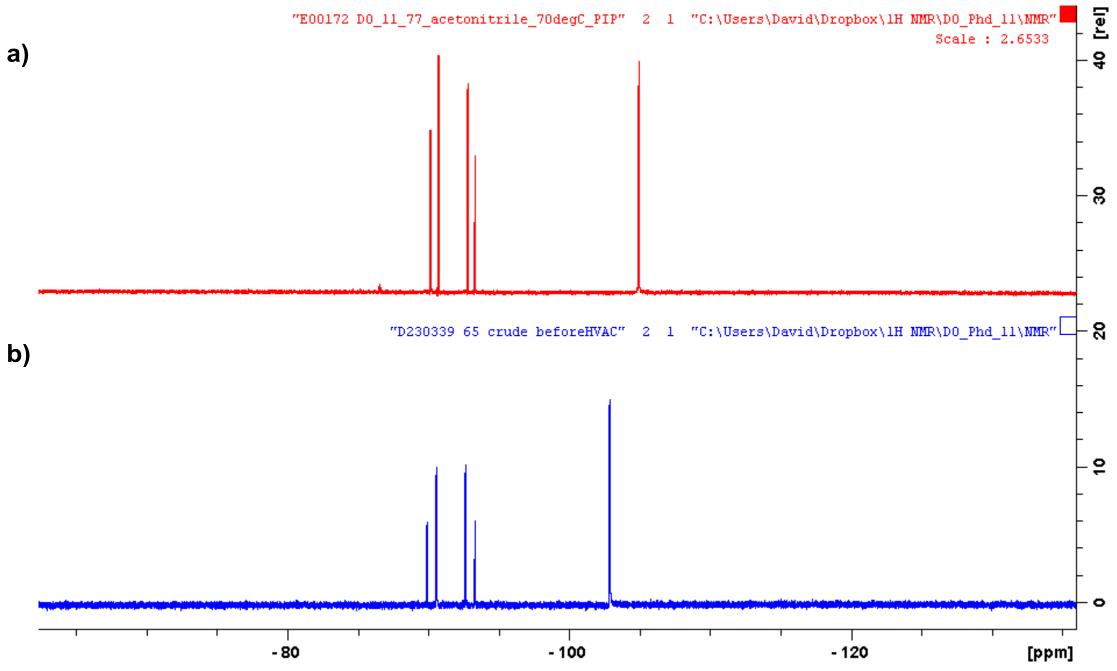


**Figure S44:** Crude $^{19}$F NMR for thermal rearrangement of **22a** in a)[D3]acetonitrile and b) [D8]toluene.

## Investigations into Alternative [3,3]-Diradical/Triplet Pathways

Attempts to use transition state **S-TS5** as a starting point to investigate triplet or diradicaloid possibilities failed when all calculations resulted in closed shell compounds ($S^2 = 0$). Instead, our previously reported *transoid* triplet intermediate **S21** was used as a starting point with atom constraints set up between alkenoate carbon and aryl C5 or C2, giving rise to *transoid* and *cisoid* structures, respectively (**Figure S42**). Molecules were manipulated in Spartan'10

to bring reaction centres within approx. 3.0 Å. Energy profiles were examined by bringing the reaction centres closer together along the constraint (3.073 Å to 1.073 Å, over 15 steps) using low level molecular mechanics (UAM1, multiplicity = 3, no MIX keyword used). Higher energy species with imaginary frequencies were examined without constraints and afforded three triplet transition states for the [3,3]-rearrangement, *transoid* **S-TS6** and *cisoid* **S-TS7a** and **S-TS7b**. These were further optimised using (U)B3LYP/6-31G* methodology with the <S2> values used to determine multiplicity of the transition state; values of 0 = closed shell, close to 0.75 = diradical and 2.0 = triplet. For triplet calculations the multiplicity was set to 3 and diradical calculations required the additional keyword MIX. For the *transoid* system (**STS-7a**) all three multiplicities gave much higher free energies of activation ($\Delta G^{\ddagger}$ = 37.8-53.6 kcal $mol^{-1}$) with respect to the previously optimised closed-shell **S-TS5** ($\Delta G^{\ddagger}$ = 27.8 kcal $mol^{-1}$). Only a triplet transition state optimised from *cisoid* **S-TS7a** ($\Delta G^{\ddagger}$ = 59.2 kcal $mol^{-1}$). Alternative *cisoid* **S-TS7b** optimised as a closed shell transition state ($\Delta G^{\ddagger}$ = 25.7 kcal $mol^{-1}$), the diradical calculations resulted a similar closed shell transition state (<S2> = 0). This newly found closed-shell **S-TS7b** and **S-TS5** were further optimised in Gaussian'09 and gave the same $\Delta G^{\ddagger}$ value of 23.9 kcal $mol^{-1}$. These investigations confirmed that the *cisoid*-closed shell **S-TS5** is the lowest energy pathway for aromatic-vinylcyclopropane rearrangements.

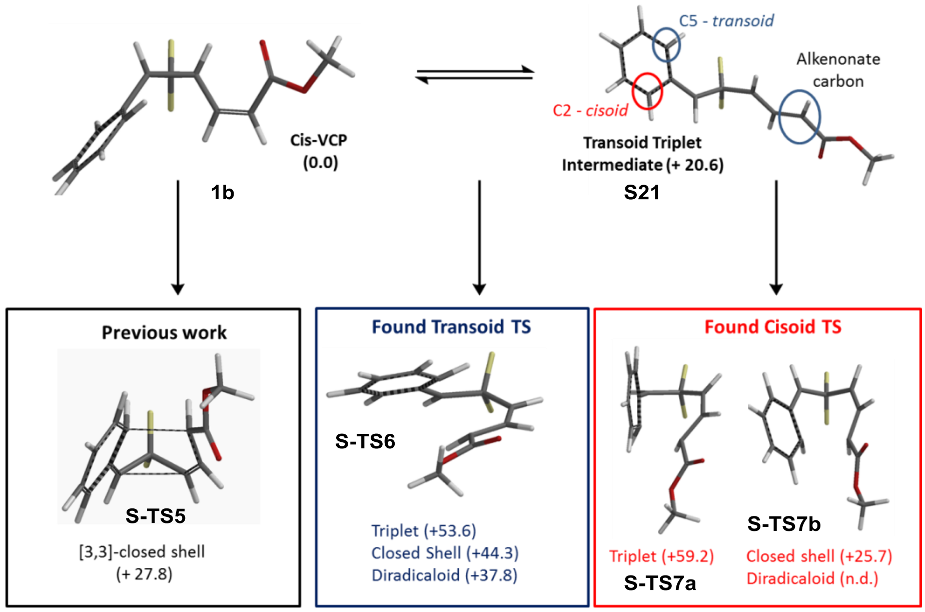


**Figure S45:** Closed-shell, diradical and triplet transition states for [3,3]-rearrangement (units in kcal $mol^{-1}$).

**Arrhenius Plot for divinyl-difluorocyclopropane S3**

The kinetic data reported by Erbes and Boland[S11] for the rearrangement of divinyl-**S3** was altered into Arrhenius parameters in Excel (**Table S13**) and plotted as described above. The resulting Arrhenius plot (**Figure S49**) and errors (**Figure S50**) gave an experimental $E_a$ value for the divinylcyclopropane rearrangements as (24.0 ± 0.17) kcal mol$^{-1}$; within error of reported value of 23.8 kcal mol$^{-1}$. The pre-exponential factor (A) was calculated as 7.4 x 10$^{16}$ s$^{-1}$. These values were used to recalculated the activation energy at 298 K.

**Table S13:** Kinetic Data used to generate Arrhenius Plot in **Figure S49**.

| T (K) | 1/T | k (x 10$^{-5}$) s$^{-1}$ | ln k |
|---|---|---|---|
| 298 | 0.003356 | 0.75 | -0.2877 |
| 313 | 0.003195 | 4.94 | 1.5974 |
| 323 | 0.003096 | 14.9 | 2.7014 |
| 333 | 0.003003 | 47.8 | 3.8670 |
| 343 | 0.002915 | 152.0 | 5.0239 |

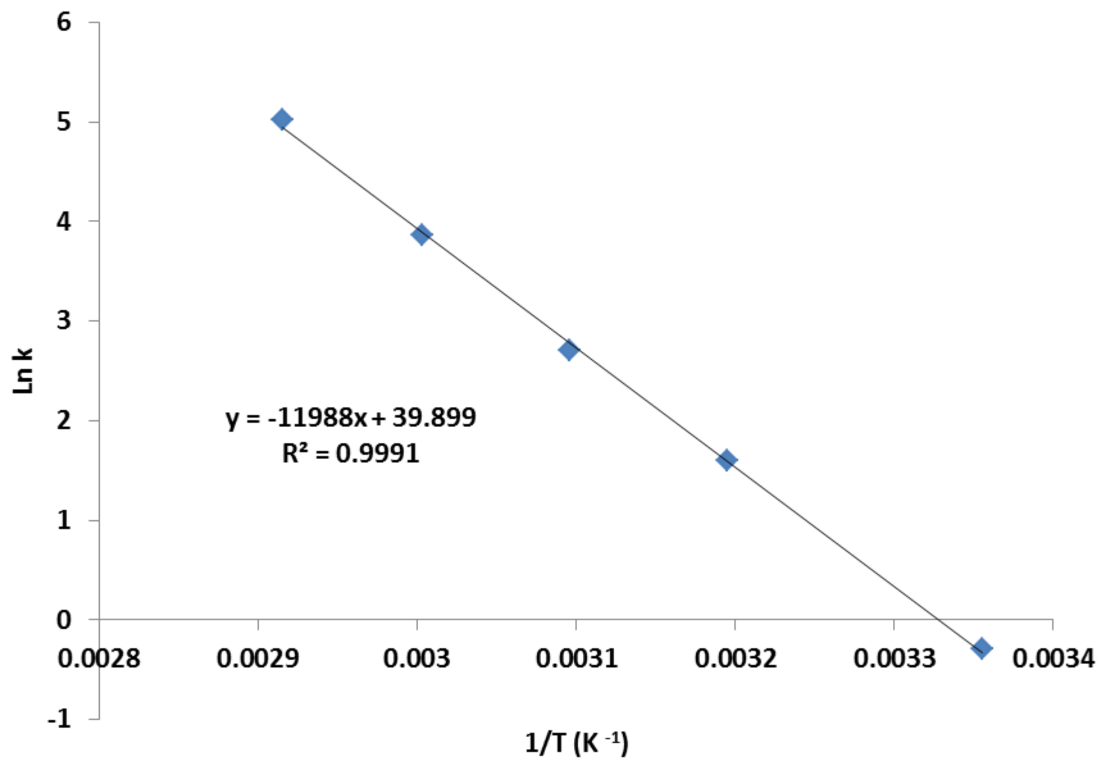


**Figure S46**: Arrhenius plot from literature kinetic data for the [3,3]-rearrangement of **S3**.

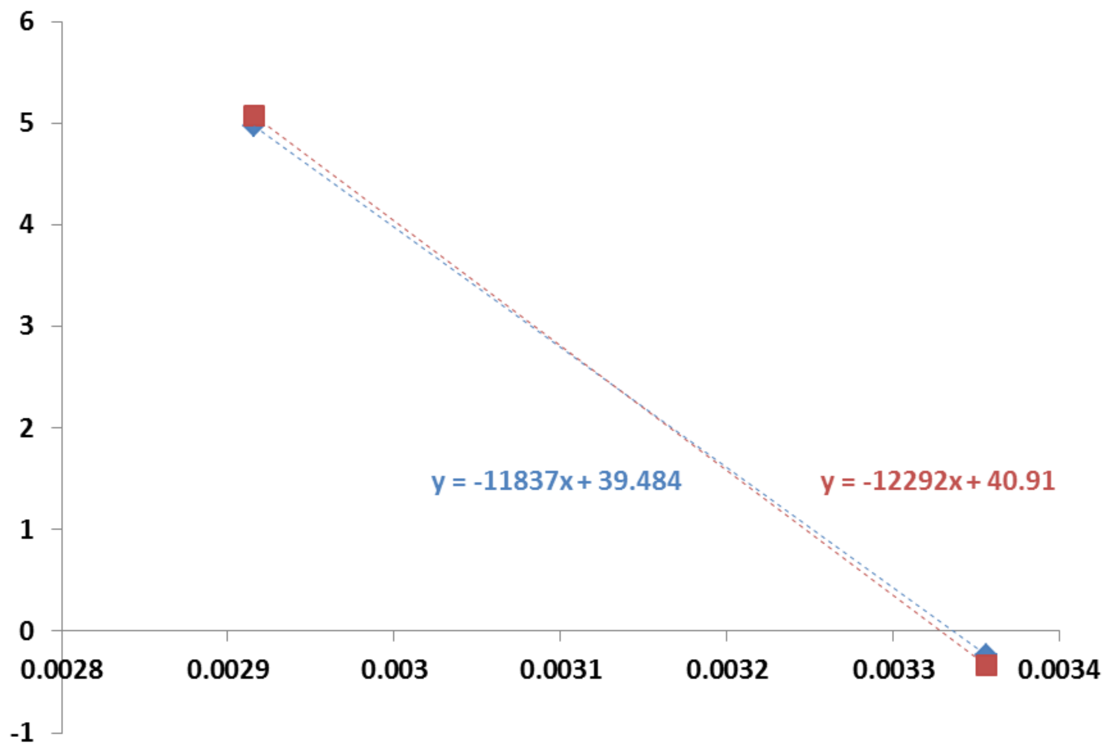


**Figure S50:** Error associated with Arrhenius plot in **Figure S49.**

## [3,3]-Sigmatropic Computational Methodology Screening

We looked to explore the effectiveness of the B3LYP/6-31G* and M06-2X/6-31G* calculations of barrier heights ($\Delta G^{\ddagger}_{B3LYP}$ and $\Delta G^{\ddagger}_{M062X}$, respectively) for divinylcyclopropane[S11,44,45] and heteroaromatic-vinylcyclopropane rearrangements[S46] reported in the literature (**Figure S51**).

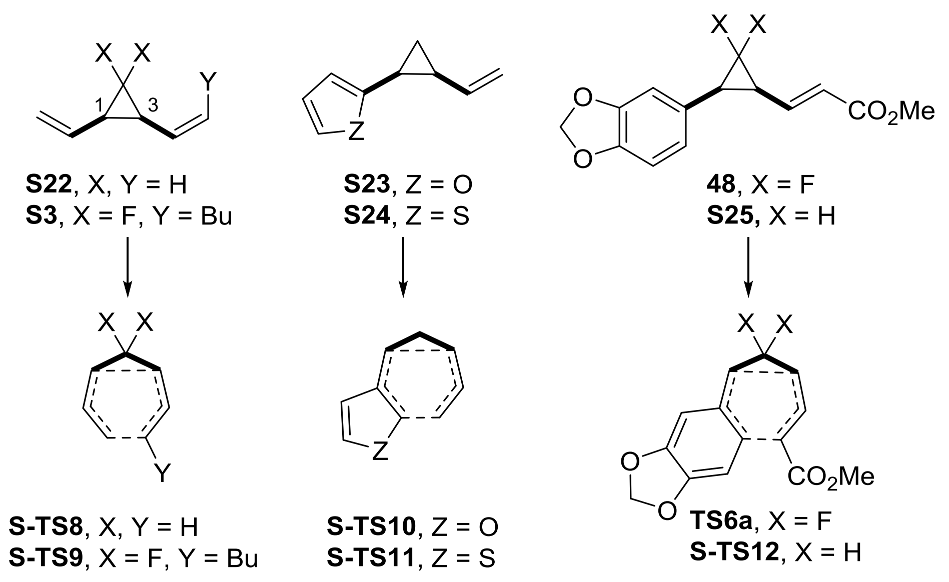


**Figure S51:** [3,3]-precursors and their corresponding transition states examined with electronic structure calculations.

Little difference was observed for the calculated $\Delta G^{\ddagger}$ values for the divinylcyclopropane rearrangement of di-VCP **S22** and fluorinated **S3** using the DFT method ($\Delta G^{\ddagger}_{B3LYP}$ difference of 0.4 kcal mol$^{-1}$) but the Minnesota method gave a $\Delta G^{\ddagger}_{M06\text{-}2X}$ difference of 4.6 kcal mol$^{-1}$ between systems (**Table S14**). The latter was more consistent with the distal bond of *gem*-difluorocyclopropane being weaker than the corresponding non-fluorinated system.[S56]

Due to the disruption of aromaticity in **S-TS10** and **S-TS11**, the aromatic-vinylcyclopropane rearrangement of furyl-**S23** and thiophenyl-**S24** showed higher $\Delta G^{\ddagger}$ values of approximately 4.0 kcal mol$^{-1}$ (B3LYP) or 10.0 kcal mol$^{-1}$ (M06-2X) than **S22**; this was less significant for piperonyl **48** (2.8 kcal mol$^{-1}$ (B3LYP) and 6.1 kcal mol$^{-1}$ (M06-2X)). In our system, the difluorocyclopropyl ring has a beneficial effect on the [3,3]-rearrangement, with $\Delta G^{\ddagger}$ values approximately 8 kcal mol$^{-1}$ lower than the corresponding non-fluorinated **S25** ($\Delta\Delta G^{\ddagger}_{B3LYP}$ = 31.8 kcal mol$^{-1}$ and $\Delta\Delta G^{\ddagger}_{M06\text{-}2X}$ = 33.2 kcal mol$^{-1}$).

**Table S14:** Barriers for [3,3]-rearrangement from electronic structure calculations (ESC) and recalculated experimental Arrhenius data.

| **Computational Methodology** | **Barriers for [3,3]-Rearrangement ($\Delta G^{\ddagger}$, kcal $mol^{-1}$, gas phase, 298 K)** | | | | | |
|---|---|---|---|---|---|---|
| | **S22 -> S-TS8** | **S3 -> S-TS9** | **S23-> S-TS10** | **S24 -> S-TS11** | **48 -> TS6a** | **S25 -> S-TS12** |
| B3LYP/6-31G* | 21.1 | 21.6 | 26.1 | 26.2 | 23.9 | 31.8 |
| M06-2X/6-31G* | 23.5 | 19.2 | 29.7 | 29.8 | 25.6 | 33.2 |
| *Experimental*[a] | *21.0* | *17.6* | *25.7* | *27.0* | *26.3* | *n.d.* |
| *(error)* | *(± 3.8)* | *(± 5.9)* | *(± 3.0)* | *(± 2.6)* | *(± 1.9)* | |

[a] All experimental values were recalculated to 298 K to allow comparison with ESC. Arrhenius determination of kinetic data for substrate **S3** provided an experimental $\Delta G^{\ddagger}$ which was recalculated to 298 K using methods described by Maskill. [S38] Calculated values of $\Delta S^{\ddagger}$ and $\Delta H^{\ddagger}$ were used for precursors **S23** and **S24** to recalculated $\Delta G^{\ddagger}$ values to 298 K.

It was promising that the majority of calculated free energies of activation were within experimental error, but the size of the errors made it difficult to assess the most accurate method for the [3,3]-rearrangement fully (**Figure S52**). The B3LYP/6-31G* was the most accurate for the divinylcyclopropane rearrangement with a $\Delta\Delta G^{\ddagger}$ value of +0.1 kcal $mol^{-1}$, comparable with Özkan and Zoras' DFT studies ($\Delta\Delta G^{\ddagger}$ = -0.3 kcal $mol^{-1}$).[S43] In contrast, the M06-2X/6-31G* calculations gave the best results with difluoro-**S3** (($\Delta\Delta G^{\ddagger}$ = +1.6 kcal $mol^{-1}$) but the extremely large error associated the experimental free energy of activation (± 5.9 kcal $mol^{-1}$) made it difficult for an effective comparison between methods.

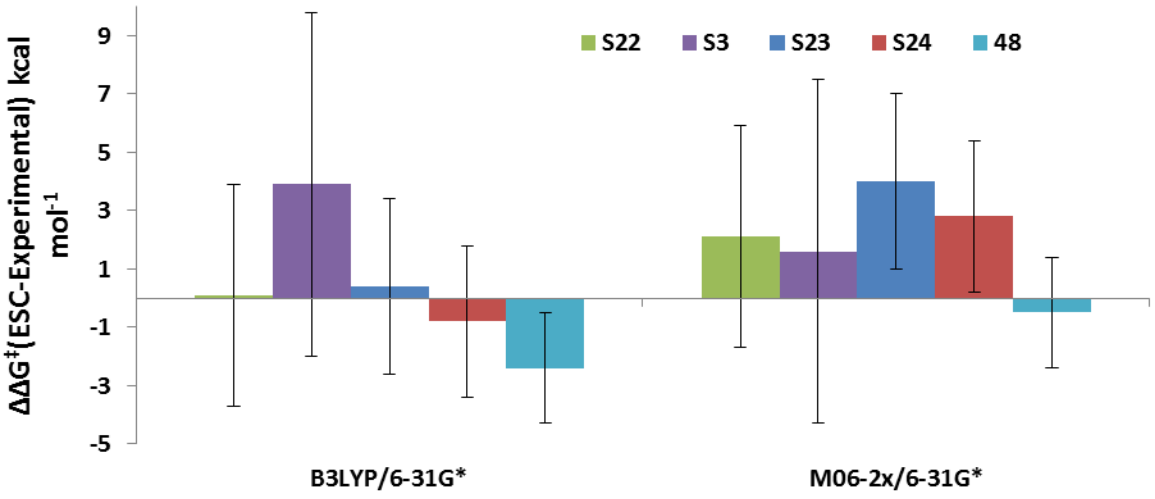


**Figure S52:** Differences between experimental ΔG$^‡$ (298 K) and ΔG$^‡$ from electronic structure calculations (298 K), plotted as ΔΔG$^‡$ (ΔG$^‡$ (ESC) - ΔG$^‡$(experimental), Gaussian'09, kcal mol$^{-1}$). Expected error associated with calculated data is ± 0.5 kcal mol$^{-1}$. Error bars represent error associated with experimental data (see **Table S12** for values).

For the aromatic-vinylcyclopropane rearrangement, the closest agreement with experimental values was observed with B3LYP/6-31G* for hetereoaromatic substituents **S23** and **S24** (ΔΔG$^‡$ values were within 0.8 kcal mol$^{-1}$) but M06-2X/6-31G* was favoured for piperonyl **48** ((ΔΔG$^‡$ values were within 0.7 kcal mol$^{-1}$).

**Error Associated with T1 and G3(MP2) Calculations**

All aromatics examined experimental were built *de novo* on Spartan'10 and minimised using low level molecular mechanics then the heat of formation calculated using the T1 and G3(MP2) thermochemical recipe. The calculated heat of formation ($\Delta H_f°$, **Table 13**) were compared with experimental values to determine the error associated with the calculations (**Figure S53**).

**Table S15:** Experimental and Calculated Heats of Formation

| Compound | Heat of Formation ($\Delta H_f°$, 298 K, kcal mol$^{-1}$) | | | | |
|---|---|---|---|---|---|
| | **Experimental[a]** | **T1** | **$\Delta\Delta H_f°$[b]** | **G3(MP2)** | **$\Delta\Delta H_f°$[b]** |
| Benzene | 19.8 | 18.7 | -1.1 | 19.1 | -0.7 |
| 1,3-Benzodixoane | -34.1[c] | -38.5 | -4.4 | -38.8 | -4.7 |
| Furan | -8.2 | -7.7 | 0.5 | -7.8 | 0.4 |
| Pyrrole | 25.9 | 26.8 | 0.9 | 26.7 | 0.8 |
| Pyridine | 33.6 | 33.0 | -0.6 | 33.2 | -0.4 |
| Thiophene | 27.5[d] | 25.6 | -1.9 | 26.0 | -1.5 |
| Oxazole | -3.7 | -2.4 | 1.3 | -2.6 | 1.1 |
| Isoxazole | 18.8 | 20.9 | 2.1 | 20.5 | 1.8 |
| Ethane | -20.2 | -20.0 | 0.2 | -19.9 | 0.3 |
| Ethylene | 12.5 | 12.4 | -0.1 | 12.0 | -0.5 |

[a] All values reported by Domalski unless otherwise stated.[S48] [b] $\Delta\Delta H_f°$ = ($\Delta H_f°$ calculated) - ($\Delta H_f°$ experimental). [c] Value reported by Cohen.[S50] [d] Value reported by Hubbard and co-workers.[S49]

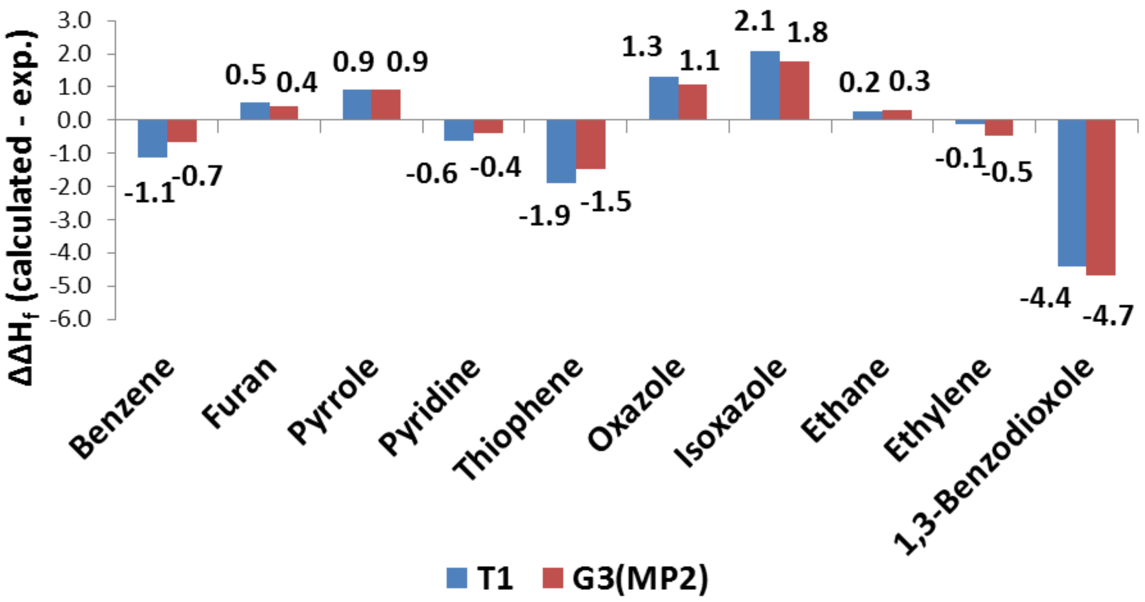


**Figure S47:** Difference in calculated and experimental heats of formation.

## Raw Data for Isodesmic Reactions

The enthalpic change (ΔH°, B3LYP/6-31G* and M06-2X/6-31G*) and heat of formation ($\Delta H_f$, T1 and G3(MP2) for the isodesmic reactions depicted in **Scheme 10** were calculated on Spartan'10 using the method described above.

Raw data for benzene, 1,3-benzodioxole, furan and thiophene are displayed in **Tables S16-S19**.

**Table S16:** Raw energy values based on isodesmic reaction with benzene.

| Method | benzene | Ethane | Total | reduced | Ethene | Total | $\Delta H_f$ or ΔH° |
|---|---|---|---|---|---|---|---|
| T1 (kJ mol$^{-1}$) | 78.22 | -83.67 | -5.45 | 106.59 | 51.70 | 158.29 | 163.74 |
| G3(MP2) (kJ mol$^{-1}$) | 80.017 | -83.366 | -3.35 | 106.35 | 50.35 | 156.71 | 160.054 |
| B3LYP (a.u.) | -232.14 | -79.75 | -311.89 | -233.29 | -78.53 | -311.82 | 0.071055 |
| M062x (a.u.) | -232.03 | -79.72 | -311.75 | -233.17 | -78.50 | -311.68 | 0.069946 |

**Table S17:** Raw energy values based on isodesmic reaction with 1,3-benzodioxole.

| Method | benzodioxole | Ethane | Total | reduced | Ethene | Total | ΔHf or ΔH° |
|---|---|---|---|---|---|---|---|
| T1 (kJ mol$^{-1}$) | -161.11 | -83.67 | -244.78 | -136.07 | 51.70 | -84.37 | 160.41 |
| G3(MP2) (kJ mol$^{-1}$) | -162.45 | -83.37 | -245.82 | -138.56 | 50.35 | -88.21 | 157.609 |
| B3LYP (a.u.) | -420.65 | -79.75 | -500.40 | -421.81 | -78.53 | -500.34 | 0.0675978 |
| M062x (a.u.) | -420.48 | -79.69 | -500.17 | -421.63 | -78.48 | -500.11 | 0.0659189 |

**Table S18:** Raw energy values based on isodesmic reaction with thiophene.

| Method | thiophene | Ethane | Total | reduced | Ethene | Total | $\Delta H_f$ or $\Delta H°$ |
|---|---|---|---|---|---|---|---|
| T1 (kJ mol$^{-1}$) | 107.11 | -83.67 | 51.70 | 76.24 | 51.70 | 127.94 | 76.24 |
| G3(MP2) (kJ mol$^{-1}$) | 108.91 | -83.37 | 50.35 | 79.29 | 50.35 | 129.64 | 79.285 |
| B3LYP (a.u.) | -552.93 | -79.75 | -632.68 | -554.11 | -78.53 | -632.64 | 0.040688 |
| M062x (a.u.) | -552.82 | -79.72 | -632.54 | -553.99 | -78.51 | -632.50 | 0.041781 |

**Table S19:** Raw energy values based on isodesmic reaction with furan.

| Method | furan | Ethane | Total | reduced | Ethene | Total | $\Delta H_f$ or $\Delta H°$ |
|---|---|---|---|---|---|---|---|
| T1 (kJ mol$^{-1}$) | -32.24 | -83.67 | -115.91 | -73.30 | 51.70 | -21.60 | 94.31 |
| G3(MP2) (kJ mol$^{-1}$) | -32.73 | -83.37 | -116.10 | -73.96 | 50.35 | -23.61 | 92.486 |
| B3LYP (a.u.) | -229.95 | -79.75 | -309.70 | -231.12 | -78.53 | -309.68 | 0.0408578 |
| M062x (a.u.) | -229.85 | -79.72 | -309.57 | -231.02 | -78.51 | -309.52 | 0.0430657 |

All raw values were converted into kcal mol$^{-1}$ for comparison (**Table S20**). Differences between $\Delta H_f$ and $\Delta H°$ values were used to assess how well the DFT and Minnesota functionals dealt with the loss of aromaticity in the isodesmic reaction (**Figures S50** and **S51**).

**Table S20:** Compiled Heats of Formation ($\Delta H_f$) and Enthalpies ($\Delta H°$) for Isodesmic Reaction (all energies are in kcal mol$^{-1}$).

| Method | | benzene | furan | thiophene | 1,3-benzodioxole |
|---|---|---|---|---|---|
| $\Delta H_f$ | T1 | 39.1 | 38.3 | 22.5 | 18.2 |
| | G3(MP2) | 38.3 | 37.7 | 22.1 | 18.9 |
| $\Delta H°$ | B3LYP | 44.6 | 42.4 | 25.6 | 25.5 |
| | M062X | 43.0 | 41.3 | 26.1 | 25.3 |

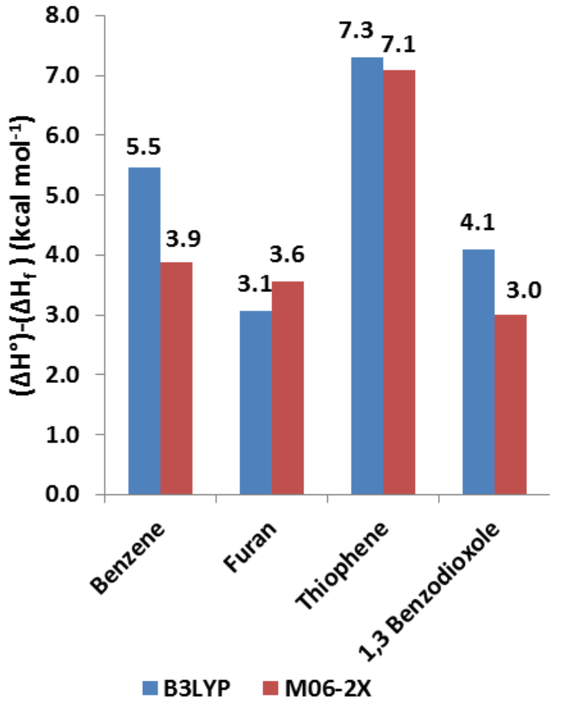


**Figure S48**: T1 differences between calculated ΔH° and $\Delta H_f$ .

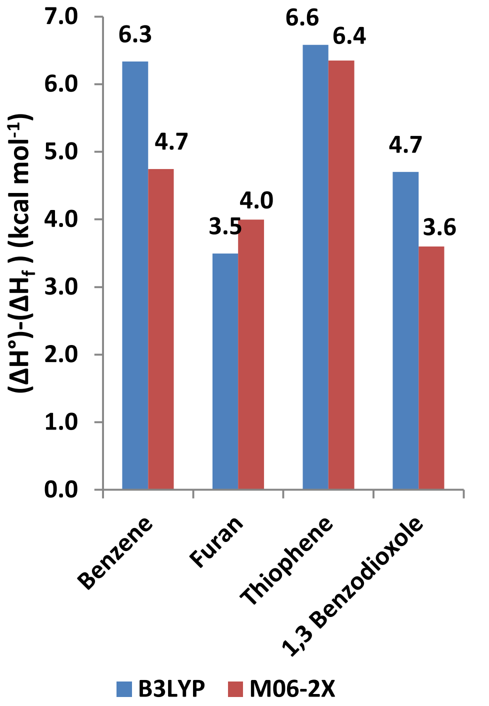


**Figure S55**: G3(MP2) differences between calculated ΔH° and $\Delta H_f$ .

## Free Energy Values for Computational Predictions

**Table S21:** Calculated free energies of activation ($\Delta G^{\ddagger}$) for VCPR and [3,3]-rearrangement.

| VCP | Calculated $\Delta G^{\ddagger}$ (Gaussian'09, gas phase, 298 K, kcal mol$^{-1}$) | | | | | | | |
|---|---|---|---|---|---|---|---|---|
| | (U)B3LYP[a] | | Corrected | | UM052X[b] | M062X[a] | Corrected | |
| | VCPR | [3,3] | VCPR[c] | [3,3] [d] | VCPR | [3,3] | VCPR[e] | [3,3] [f] |
| **1a** | 26.6 | 27.8 | 29.3 | 30.2 | 29.8 | 29.5 | 28.6 | 30.0 |
| **47** | 35.1 | 35.5 | 37.8 | 37.9 | 38.1 | 36.5 | 36.9 | 37.0 |
| **22a** | 24.3 | 23.9 | 27.0 | 26.3 | 28.1 | 27.0 | 26.9 | 27.8 |
| **20a** | 30.6 | 33.9 | 33.3 | 36.3 | 32.7 | 35.0 | 31.5 | 35.5 |
| **23a** | 22.1 | 17.5 | 24.8 | 19.9 | 25.7 | 20.7 | 24.5 | 21.2 |
| **25a** | 25.3 | 24.2 | 28.0 | 26.6 | 32.7 | 26.1 | 26.7 | 26.6 |
| **27a** | 22.5 | 21.8 | 25.2 | 24.2 | 26.2 | 24.2 | 25.0 | 24.7 |
| **27a** | 26.3 | 32.6 | 29.0 | 35.0 | 30.4 | 33.0 | 29.2 | 33.5 |
| **24a** | 22.5 | 18.5 | 25.2 | 20.9 | 26.0 | 21.2 | 24.8 | 21.7 |
| **26a** | 25.7 | 25.0 | 28.4 | 27.4 | 29.2 | 27.5 | 28.0 | 28.0 |
| **19a** | 26.2 | 33.3 | 28.9 | 35.7 | 28.0 | 35.6 | 27.6 | 36.1 |
| **21a** | 24.7 | 27.9 | 27.4 | 30.3 | 28.8 | 30.8 | 27.6 | 31.3 |
| **21b** | 29.7 | 32.7 | 32.4 | 35.1 | 34.0 | 36.7 | 32.8 | 37.2 |

[a] Using 6-31G* basis set. [b] Using 6-31+G* basis set. [c] $\Delta G^{\ddagger}$ + 2.7 kcal mol$^{-1}$ (UB3LYP/6-31G*) [d] Calculated $\Delta G^{\ddagger}$ + 2.4 kcal mol$^{-1}$ (B3LYP/6-31G*) [e] Calculated $\Delta G^{\ddagger}$ - 1.2 kcal mol$^{-1}$ (UM05-2X/6-31+G*) [f] Calculated $\Delta G^{\ddagger}$ + 0.5 kcal mol$^{-1}$ (M06-2X/6-31G*). All intermediates and transition states are for conformationally simpler Me ester.

## VCPR Temperature Predictions

**Table S22** represents the data used to generate temperature prediction graphs for M05-2x/6-31+G* and B3LYP/6-31+G* methods (**Figure 12** and **Figure S56**, respectively).

**Table S22:** Temperature and $\Delta G^{\ddagger}$ data for prediction graphs.

| VCP | Opt. Reaction Temp. | | VCPR $\Delta G^{\ddagger}$ | |
|---|---|---|---|---|
| | °C | K | B3LYP/6-31G* | M05-2X/6-31+G* |
| 22a | 70 | 343 | 27.0 | 26.9 |
| 21a | 90 | 363 | 27.4 | 27.6 |
| 19a | 95 | 368 | 28.9 | 28.0 |
| 1a | 100 | 373 | 29.3 | 28.6 |
| 21b | 160 | 433 | 32.4 | 32.8 |
| 47 | 220 | 493 | 37.8 | 36.9 |

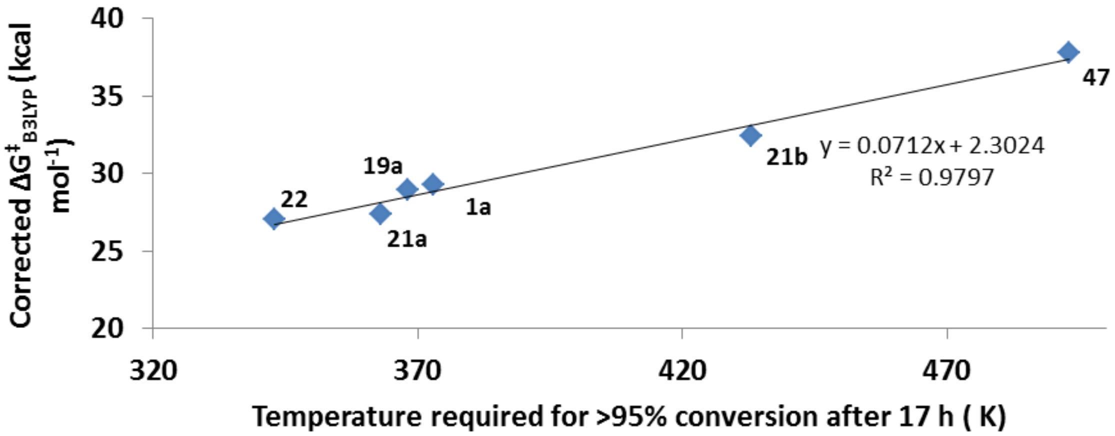


**Figure S56:** Trend between corrected $\Delta G^{\ddagger}$ values for VCPR (UB3LYP/6-31G*) and rearrangement temperature.

**References**


(S1) Waser, J.; Gaspar, B.; Nambu, H.; Carreira, E. M. *J. Am. Chem. Soc*. **2006**, *128*, 11693.
(S2) Lohre, C.; Fröhlich, R.; Glorius, F. *Synthesis* **2008**, 2221.
(S3) Hansch, C.; Leo, A.; Taft, R. W. *Chem. Rev*. **1991**, *91*, 165.
(S4) Fischer, E. O.; Maasböl, A. *Angew. Chem. Int. Ed*. **1964**, *3*, 580.
(S5) Agirbas, H.; Jackson, R. A. *J. Chem. Soc., Perkin Trans. 2* **1983**, 739.
(S6) Jiang, X.; Ji, G. *J. Org. Chem*. **1992**, *57*, 6051.
(S7) Dust, J. M.; Arnold, D. R. *J. Am. Chem. Soc*. **1983**, *105*, 1221.
(S8) Creary, X. *Acc. Chem. Res*. **2006**, *39*, 761.
(S9) Zipse, H. In *Radicals in Synthesis* I; Gansäuer, A., Ed.; Springer Berlin Heidelberg: **2006**; Vol. *263*, p 163.
(S10) Henry, D. J.; Parkinson, C. J.; Mayer, P. M.; Radom, L. *J. Phys. Chem. A* **2001**, *105*, 6750.
(S11) Erbes, P.; Boland, W. *Helv. Chim. Acta* **1992**, *75*, 766.
(S12) Creary, X.; Wolf, A.; Miller, K. *Org. Lett*. **1999**, *1*, 1615.
(S13) Schneider, T. F.; Werz, D. B. *Org. Lett*. **2011**, *13*, 1848.
(S14) Schneider, T. F.; Kaschel, J.; Werz, D. B. *Angew. Chem. Int. Ed*. **2014**, *53*, 5504.
(S15) Bruno, I.; Cole, J.; Lommerse, J. M.; Rowland, R. S.; Taylor, R.; Verdonk, M. *J. Comput. Aided Mol. Des*. **1997**, *11*, 525.
(S16) Lebel, H.; Davi, M. *Adv. Synth. Catal*. **2008**, *350*, 2352.
(S17) Iwasaki, M.; Kobayashi, Y.; Li, J. P.; Matsuzaka, H.; Ishii, Y.; Hidai, M. *J. Org. Chem*. **1991**, *56*, 1922.
(S18) Acharya, A.; Eickhoff, J. A.; Jeffrey, C. S. *Synthesis* **2013**, *45*, 1825.
(S19) Arimitsu, S.; Hammond, G. B. *J. Org. Chem*. **2007**, 72, 8559.
(S20) Orr, D.; Percy, J. M.; Tuttle, T.; Kennedy, A. R.; Harrison, Z. A. *Chem. Eur. J.* **2014**, *20*, 14305.
(S21) Charette, A. B.; Juteau, H.; Lebel, H.; Molinaro, C. *J. Am. Chem. Soc*. **1998**, *120*, 11943.
(S22) Dolbier Jr, W. R.; Tian, F.; Duan, J.-X.; Li, A.-R.; Ait-Mohand, S.; Bautista, O.; Buathong, S.; Marshall Baker, J.; Crawford, J.; Anselme, P.; Cai, X. H.; Modzelewska, A.; Koroniak, H.; Battiste, M. A.; Chen, Q.-Y. *J. Fluorine Chem*. **2004**, *125*, 459.
(S23) Wang, F.; Luo, T.; Hu, J.; Wang, Y.; Krishnan, H. S.; Jog, P. V.; Ganesh, S. K.; Prakash, G. K. S.; Olah, G. A. *Angew. Chem. Int. Ed*. **2011**, *50*, 7153.
(S24) Oshiro, K.; Morimoto, Y.; Amii, H. *Synthesis* **2010**, *12*, 2080.
(S25) Eusterwiemann, S.; Martinez, H.; Dolbier, W. R. *J. Org. Chem*. **2012**, *77*, 5461.
(S26) Chen, Q.; Wu, S. J. Org. Chem. 1989, 54, 3023.
(S27) Aono, T.; Sasagawa, H.; Fuchibe, K.; Ichikawa, J. Org. Lett. 2015, *17*, 5736.
(S28) Tian, F.; Kruger, V.; Bautista, O.; Duan, J.-X.; Li, A.-R.; Dolbier Jr, W. R.; Chen, Q.-Y. *Org. Lett*. **2000**, *2*, 563.
(S29) Hagooly, Y.; Cohen, O.; Rozen, S. *Tetrahedron Lett*. **2009**, *50*, 392.
(S30) Houk, K. N.; Rondan, N. G.; Mareda, J. *Tetrahedron* **1985**, *41*, 1555.
(S31) Fuchibe, K.; Koseki, Y.; Aono, T.; Sasagawa, H.; Ichikawa, J. *J. Fluorine Chem*. **2012**, *133*, 52.
(S32) Thomoson, C. S.; Dolbier, W. R. *J. Org. Chem*. **2013**, *78*, 8904.
(S33) Thomoson, C. S.; Wang, L.; Dolbier, W. R. *J. Fluorine Chem*. **2014**, *168*, 34.
(S34) Li, L.; Wang, F.; Ni, C.; Hu, J. *Angew. Chem. Int. Ed*. **2013**, *52*, 12390.
(S35) Deng, X.-Y.; Lin, J.-H.; Zheng, J.; Xiao, J.-C. *Chem. Commun*. **2015**, *51*, 8805.

(S36) Zhou, J.; Campbell-Conroy, E. L.; Silina, A.; Uy, J.; Pierre, F.; Hurley, D. J.; Hilgraf, N.; Frieman, B. A.; DeNinno, M. P. *J. Org. Chem*. **2015**, *80*, 70.
(S37) Zahnley, T.; Macey, R.; Oster, G. Berkeley Madonna; 8.3.18 ed.; University of California at Berkeley: Berkeley, California, **2010**.
(S38) Maskill, H., *The Physical Basis of Organic Chemistry*, OUP, Oxford, **1985**, Chapter 6, 242.
(S39) Zhao, Y. ; Schultz, N. E.; Truhlar, D. G. *J. Chem. Phys*., **2005**, *123*, 161103.
(S40) Zhao, Y. ; Truhlar, D. G. *Theor. Chem. Acc*., **2008**, *120*, 215.
(S41) Yanai, T.; Tew, D. P.; Handy, N. C. *Chem. Phys. Lett*., **2004**, *393*, 51. It is clear that the extent of HF exchange does have an effect on the sign of difference between experimental and calculated values; a wider set of systems would have to be examined before the levels of accuracy of (U)B3LYP and CAM-B3LYP could be assessed more fairly. We proceeded with (U)B3LYP because of the similarity of outcomes between the open-shell singlet and {3,3]-rearrangement pathways; they are more alike than those for for CAM-B3LYP.
(S42) Maas, G. *Chem. Ber*., **1979**, *112*, 3241.
(S43) Özkan, I.; Zora, M. *J. Org. Chem*., **2003**, *68*, 9635.
(S44) Brown, J. M.; Golding, B. T.; Stofko, J. J. *J. Chem. Soc., Chem. Commun*., **1973**, 319.
(S45) Maas, G.; Hummel, C. *Chem. Ber*., **1980**, *113*, 3679.
(S46) Ohlinger, W. S.; Klunzinger, P. E. ; Deppmeier, B. J.; Hehre, W. J. *J. Phys. Chem. A*, **2009**, *113*, 2165.
(S47) Curtiss, L. A. ; Raghavachari, K.; Pople, J. A. *J. Chem. Phys*., **1993**, *98*, 1293.
(S48) Domalski, E. S. *J. Phys. Chem. Ref. Data*, **1972**, *1*, 221.
(S49) Hubbard, W. N.; Scott, D. W.; Frow, F. R.; Waddington, G. *J. Am. Chem. Soc*., **1955**, *77*, 5855.
(S50) Cohen, N. J. Phys. Chem. Ref. Data, 1996, 25, 1411-1481.
(S51) Lewis, D. K.; Charney, D. J.; Kalra, B. L.; Plate, A.-M.; Woodard, M. H.; Cianciosi, S. J.; Baldwin, J. E. *J. Phys. Chem. A* **1997**, *101*, 4097.
(S52) http://www.gaussian.com/g_tech/g_ur/k_scrf.htm, Accessed on 9th Nov. 2015.
(S53) Nelson, R. D., Lide, D. R., Maryott, A. A., & United States. National Bureau of Standards. (1967). Selected values of electric dipole moments for molecules in the gas phase. Washington, D.C.: U.S. Dept. of Commerce, National Bureau of Standards. (http://www.nist.gov/data/nsrds/NSRDS-NBS-10.pdf, Accessed 9th Nov. 2015).
(S54) Tomasi, J.; Mennucci, B.; Cammi, R. *Chem. Rev*. **2005**, *105*, 2999.
(S55) Cossi, M.; Rega, N.; Scalmani, G.; Barone, V. *J. Comput. Chem*. **2003**, *24*, 669.
(S56) Dolbier, W. R. *Acc. Chem. Res*. **1981**, *14*, 195.

**Cartesian Coordinates**

Cartesian Coordinates used in electronic structure calculations – Spartan’10 structures (Gaussian’09 energy values provided if obtained). All energies are in a.u..

**Triage: Cyclopropane Substitution**

## Pyyrole SM (9a)

G = -830.0446014 a.u.
vi = none

Cartesian Coordinates (Angstroms)

| Atom | | | X | Y | Z |
|---|---|---|---|---|---|
| 1 | C | C1 | 1.7222269 | 1.2445885 | -0.9729440 |
| 2 | C | C2 | 0.6041819 | 1.6925847 | -0.1197857 |
| 3 | C | C3 | 0.9304149 | 0.1385579 | -0.3669379 |
| 4 | H | H3 | -0.2634018 | 2.0265621 | -0.6880495 |
| 5 | H | H6 | 1.3972895 | -0.3352988 | 0.4928236 |
| 6 | C | C10 | 0.0096991 | -0.6684673 | -1.1704285 |
| 7 | H | H11 | -0.3761963 | -0.2280125 | -2.0883867 |
| 8 | C | C11 | -0.4005248 | -1.9064327 | -0.8511647 |
| 9 | H | H12 | -0.0564018 | -2.4218961 | 0.0407895 |
| 10 | C | C12 | -1.3494274 | -2.6233645 | -1.7272019 |
| 11 | O | O1 | -1.8337068 | -2.1932897 | -2.7572939 |
| 12 | O | O2 | -1.6283020 | -3.8532978 | -1.2286266 |
| 13 | C | C13 | -2.5405183 | -4.6357388 | -2.0122175 |
| 14 | H | H5 | -3.4923969 | -4.1115587 | -2.1331000 |
| 15 | H | H13 | -2.6804404 | -5.5652310 | -1.4591559 |
| 16 | H | H14 | -2.1218863 | -4.8377263 | -3.0022856 |
| 17 | F | F1 | 1.6247087 | 1.4521976 | -2.3073350 |
| 18 | F | F2 | 3.0024035 | 1.4205751 | -0.5768409 |
| 19 | C | C4 | 0.7970878 | 2.2851547 | 1.2148360 |
| 20 | C | C5 | 1.8863411 | 2.8913793 | 1.8170170 |
| 21 | C | C6 | 1.4964665 | 3.2807468 | 3.1275918 |
| 22 | C | C7 | 0.1816452 | 2.9031882 | 3.2976489 |
| 23 | H | H1 | 2.8510224 | 3.0395555 | 1.3549197 |
| 24 | H | H8 | 2.1102781 | 3.7860042 | 3.8606918 |
| 25 | H | H9 | -0.4878149 | 3.0144079 | 4.1384141 |
| 26 | N | N1 | -0.2348714 | 2.3028757 | 2.1350296 |
| 27 | H | H2 | -1.1478764 | 1.9019360 | 1.9819925 |

## Pyyrole TS2 (TS1aa)

G = -830.419263
vi = i78
S^2 = 0.457

Cartesian Coordinates (Angstroms)

| Atom | | | X | Y | Z |
|---|---|---|---|---|---|
| 1 | C | C | -0.9297737 | 2.5748429 | 0.6568401 |

| | | | | | |
|---|---|---|---|---|---|
| 2 | C | C2 | -1.9194433 | 1.6312626 | 0.6608880 |
| 3 | C | C3 | -1.6411990 | 0.2517920 | 0.7737971 |
| 4 | C | C4 | 0.4710368 | 2.1238864 | 0.5119996 |
| 5 | C | C5 | 0.6265790 | 1.0214606 | -0.4882548 |
| 6 | H | H | -1.1171696 | 3.6410222 | 0.7171927 |
| 7 | H | H7 | -2.9595671 | 1.9256767 | 0.5328070 |
| 8 | H | H8 | -0.7662823 | -0.1029137 | 1.3009722 |
| 9 | H | H12 | -0.1240240 | 0.9537771 | -1.2623941 |
| 10 | F | F1 | 1.2877910 | 3.1931977 | 0.1719855 |
| 11 | F | F2 | 1.0287529 | 1.6979289 | 1.7555366 |
| 12 | C | C11 | -2.6538475 | -0.7310434 | 0.3954601 |
| 13 | O | O1 | -3.7440986 | -0.4774870 | -0.0947453 |
| 14 | O | O2 | -2.2384745 | -2.0004546 | 0.6602510 |
| 15 | C | C12 | -3.1717980 | -3.0280382 | 0.3079669 |
| 16 | H | H1 | -4.1123292 | -2.9029289 | 0.8523623 |
| 17 | H | H9 | -2.6929097 | -3.9684920 | 0.5846646 |
| 18 | H | H10 | -3.3842153 | -3.0079672 | -0.7652038 |
| 19 | C | C1 | 1.8411946 | 0.3622857 | -0.6905360 |
| 20 | C | C6 | 2.2631551 | -0.4716555 | -1.7562523 |
| 21 | C | C7 | 3.5614344 | -0.9103603 | -1.4767628 |
| 22 | C | C8 | 3.9309200 | -0.3557429 | -0.2484364 |
| 23 | H | H3 | 1.6666996 | -0.7030954 | -2.6285180 |
| 24 | H | H5 | 4.1791181 | -1.5561366 | -2.0853420 |
| 25 | H | H11 | 4.8492544 | -0.4533396 | 0.3134152 |
| 26 | N | N1 | 2.9011554 | 0.3967289 | 0.2113611 |
| 27 | H | H6 | 2.8480404 | 0.8957936 | 1.0889455 |

## pyyrole_higher_energyTS2 (TS1ab)

G = -830.414696
iv = i86
S^2 = 0.5102

Cartesian Coordinates (Angstroms)

| | Atom | | X | Y | Z |
|---|---|---|---|---|---|
| 1 | C | C | -0.6158183 | 2.7256632 | 0.6820107 |
| 2 | C | C2 | -1.5971922 | 2.1028680 | -0.0343527 |
| 3 | C | C3 | -1.6947996 | 0.6924256 | -0.0975346 |
| 4 | C | C4 | 0.4992112 | 1.9015225 | 1.2220471 |
| 5 | C | C5 | 0.9748909 | 0.8813229 | 0.2312319 |
| 6 | H | H | -0.5893481 | 3.7953210 | 0.8584969 |
| 7 | H | H7 | -2.3084203 | 2.6842247 | -0.6185155 |
| 8 | H | H8 | -1.3530473 | 0.0711430 | 0.7187428 |
| 9 | H | H12 | 0.8034239 | 1.1175807 | -0.8108546 |
| 10 | F | F1 | 1.5520191 | 2.7254997 | 1.5953164 |
| 11 | F | F2 | 0.1389744 | 1.2638429 | 2.4199903 |
| 12 | C | C11 | -2.4700560 | 0.0560454 | -1.1581920 |
| 13 | O | O1 | -3.0479337 | 0.6343226 | -2.0667899 |
| 14 | O | O2 | -2.4755496 | -1.3002263 | -1.0228217 |
| 15 | C | C12 | -3.2164866 | -2.0059029 | -2.0242332 |
| 16 | H | H1 | -4.2783074 | -1.7449992 | -1.9780600 |
| 17 | H | H9 | -3.0760518 | -3.0653510 | -1.8034635 |
| 18 | H | H10 | -2.8431902 | -1.7687789 | -3.0250429 |
| 19 | C | C1 | 1.9005424 | -0.1117304 | 0.5589328 |

| | | | | | |
|---|---|---|---|---|---|
| 20 | C | C6 | 2.3766330 | -0.5907480 | 1.8048456 |
| 21 | C | C7 | 3.2738964 | -1.6361421 | 1.5563537 |
| 22 | C | C8 | 3.3447797 | -1.8097260 | 0.1746747 |
| 23 | H | H3 | 2.0712456 | -0.1937930 | 2.7607488 |
| 24 | H | H5 | 3.8158363 | -2.2172635 | 2.2895826 |
| 25 | H | H11 | 3.9124580 | -2.5151602 | -0.4157203 |
| 26 | N | N1 | 2.5264033 | -0.8948515 | -0.4121237 |
| 27 | H | H6 | 2.3758869 | -0.7971094 | -1.4052698 |

## Furan trans-SM (9b)

Spartan'10: G = -850.314059 vi = none
Gaussian'09: B3LYP/6-31G*, G =-850.319618 vi = none
Gaussian'09: M05-2X/6-31G*, G = -850.262612, vi = none

Cartesian Coordinates (Angstroms)

| Atom | | | X | Y | Z |
|---|---|---|---|---|---|
| 1 | C | C1 | 1.5992207 | 1.3955614 | -0.9776466 |
| 2 | C | C2 | 0.5194974 | 1.8052430 | -0.0580500 |
| 3 | C | C3 | 0.8775038 | 0.2658666 | -0.3303277 |
| 4 | H | H3 | -0.4002896 | 2.1204185 | -0.5444083 |
| 5 | H | H6 | 1.4087112 | -0.1989848 | 0.4965651 |
| 6 | C | C10 | -0.0692639 | -0.5633929 | -1.0807464 |
| 7 | H | H11 | -0.5451553 | -0.1204950 | -1.9541452 |
| 8 | C | C11 | -0.3946896 | -1.8258644 | -0.7635527 |
| 9 | H | H12 | 0.0391353 | -2.3439339 | 0.0867471 |
| 10 | C | C12 | -1.3721319 | -2.5700978 | -1.5853767 |
| 11 | O | O1 | -1.9604997 | -2.1384533 | -2.5585899 |
| 12 | O | O2 | -1.5371983 | -3.8286142 | -1.1099537 |
| 13 | C | C13 | -2.4677534 | -4.6398049 | -1.8414309 |
| 14 | H | H5 | -3.4717553 | -4.2079556 | -1.7983632 |
| 15 | H | H13 | -2.4506879 | -5.6161272 | -1.3558111 |
| 16 | H | H14 | -2.1651118 | -4.7245612 | -2.8886582 |
| 17 | F | F1 | 1.4275165 | 1.6056448 | -2.3021574 |
| 18 | F | F2 | 2.8925579 | 1.6160802 | -0.6434690 |
| 19 | C | C4 | 0.7705242 | 2.3994701 | 1.2599089 |
| 20 | C | C5 | 1.8869409 | 2.7841676 | 1.9494628 |
| 21 | C | C6 | 1.4335592 | 3.2644071 | 3.2204022 |
| 22 | C | C7 | 0.0786439 | 3.1404409 | 3.2120022 |
| 23 | H | H1 | 2.9039660 | 2.7376631 | 1.5907183 |
| 24 | H | H8 | 2.0403843 | 3.6562081 | 4.0247172 |
| 25 | H | H9 | -0.6983851 | 3.3741271 | 3.9236340 |
| 26 | O | O3 | -0.3452394 | 2.6129866 | 2.0285292 |

## Furan TS2 (TS1bb)

Spartan'10: G = -850.28681, vi = i125, S^2 = 0.5808
Gaussian'09: B3LYP/6-31G*, G = -850.284463, vi = i137, S^2 = 0.57
Gaussian'09: M05-2X/6-31G*, G = -850.221609, vi = i163, S^2 = 0.75

Cartesian Coordinates (Angstroms)

| Atom | X | Y | Z |
|---|---|---|---|

| | | | | | |
|---|---|---|---|---|---|
| 1 | C | C | -0.8561031 | 2.7015691 | 0.3283079 |
| 2 | C | C2 | -1.8381630 | 1.8024358 | 0.0383813 |
| 3 | C | C3 | -1.6444484 | 0.4102119 | 0.2272503 |
| 4 | C | C4 | 0.5072269 | 2.1790487 | 0.6288748 |
| 5 | C | C5 | 0.8856784 | 1.0394530 | -0.2712286 |
| 6 | H | H | -0.9995893 | 3.7763684 | 0.3174435 |
| 7 | H | H7 | -2.7826728 | 2.1292823 | -0.3926797 |
| 8 | H | H8 | -1.0099284 | 0.0361089 | 1.0187846 |
| 9 | H | H12 | 0.4705459 | 1.0412851 | -1.2696769 |
| 10 | F | F1 | 1.4360242 | 3.2007638 | 0.5162551 |
| 11 | F | F2 | 0.6134274 | 1.7738239 | 1.9674583 |
| 12 | C | C11 | -2.4989795 | -0.5525386 | -0.4685442 |
| 13 | O | O1 | -3.3684446 | -0.2715912 | -1.2784601 |
| 14 | O | O2 | -2.2061722 | -1.8278254 | -0.1011751 |
| 15 | C | C12 | -2.9862758 | -2.8407750 | -0.7484711 |
| 16 | H | H1 | -4.0493679 | -2.7126694 | -0.5253266 |
| 17 | H | H9 | -2.6237993 | -3.7896870 | -0.3511872 |
| 18 | H | H10 | -2.8484901 | -2.8009163 | -1.8330240 |
| 19 | C | C1 | 2.0038465 | 0.2433660 | -0.0273076 |
| 20 | C | C6 | 2.8291747 | 0.0348828 | 1.0837613 |
| 21 | C | C7 | 3.7855008 | -0.9333375 | 0.7106043 |
| 22 | C | C8 | 3.4978427 | -1.2782298 | -0.5873676 |
| 23 | H | H3 | 2.7222504 | 0.5366346 | 2.0329496 |
| 24 | H | H5 | 4.5837052 | -1.3373896 | 1.3175395 |
| 25 | H | H11 | 3.9437590 | -1.9756074 | -1.2810134 |
| 26 | O | O3 | 2.4334523 | -0.5846670 | -1.0521485 |

## furan_higher_energyTS2 (TS1ba)

G = -850.277257
iv = i117
S^2 = 0.600

Cartesian Coordinates (Angstroms)

| Atom | | | X | Y | Z |
|---|---|---|---|---|---|
| 1 | C | C | -0.9502546 | 2.6495991 | 0.6501376 |
| 2 | C | C2 | -1.8908863 | 1.6636452 | 0.6340407 |
| 3 | C | C3 | -1.5252413 | 0.2997528 | 0.7679050 |
| 4 | C | C4 | 0.4866883 | 2.2452973 | 0.5815975 |
| 5 | C | C5 | 0.6994623 | 1.1466886 | -0.4230640 |
| 6 | H | H | -1.1843317 | 3.7083129 | 0.6705550 |
| 7 | H | H7 | -2.9410498 | 1.8946944 | 0.4667669 |
| 8 | H | H8 | -0.6672285 | 0.0082292 | 1.3580427 |
| 9 | H | H12 | 0.0014180 | 1.1086166 | -1.2480338 |
| 10 | F | F1 | 1.2635235 | 3.3417125 | 0.2455922 |
| 11 | F | F2 | 0.9578758 | 1.8500449 | 1.8288746 |
| 12 | C | C11 | -2.4336857 | -0.7569674 | 0.3243831 |
| 13 | O | O1 | -3.5074436 | -0.5836509 | -0.2312859 |
| 14 | O | O2 | -1.9309470 | -1.9884494 | 0.6061859 |
| 15 | C | C12 | -2.7538814 | -3.0880991 | 0.1987920 |
| 16 | H | H1 | -3.7287557 | -3.0463091 | 0.6926392 |
| 17 | H | H9 | -2.2124283 | -3.9867342 | 0.4969680 |

| | | | | | |
|---|---|---|---|---|---|
| 18 | H | H10 | -2.9090023 | -3.0753115 | -0.8840734 |
| 19 | C | C1 | 1.8920642 | 0.4419550 | -0.5879881 |
| 20 | C | C6 | 2.3133656 | -0.4200912 | -1.6085995 |
| 21 | C | C7 | 3.5834925 | -0.9104235 | -1.2387796 |
| 22 | C | C8 | 3.8752209 | -0.3326880 | -0.0273526 |
| 23 | H | H3 | 1.7534328 | -0.6493108 | -2.5047380 |
| 24 | H | H5 | 4.2111886 | -1.5983851 | -1.7876640 |
| 25 | H | H11 | 4.7235775 | -0.4012302 | 0.6371705 |
| 26 | O | O3 | 2.8738262 | 0.4791018 | 0.3819280 |

## para-Pyridine-N-oxide SM (9c)

G = -943.714388
vi = none

Cartesian Coordinates (Angstroms)

| Atom | | | X | Y | Z |
|---|---|---|---|---|---|
| 1 | C | C1 | 1.3089641 | 1.2878086 | -1.4497207 |
| 2 | C | C2 | 0.3057078 | 1.5125208 | -0.3822889 |
| 3 | C | C3 | 0.7951372 | 0.0499645 | -0.8058908 |
| 4 | H | H3 | -0.6826092 | 1.7431318 | -0.7723904 |
| 5 | H | H6 | 1.4675209 | -0.4016004 | -0.0798213 |
| 6 | C | C10 | -0.1475861 | -0.8399612 | -1.4924370 |
| 7 | H | H11 | -0.7435160 | -0.4144786 | -2.2979791 |
| 8 | C | C11 | -0.3306841 | -2.1327832 | -1.1854696 |
| 9 | H | H12 | 0.2308402 | -2.6289737 | -0.3990166 |
| 10 | C | C12 | -1.3267131 | -2.9400203 | -1.9226782 |
| 11 | O | O1 | -2.0384947 | -2.5410180 | -2.8245489 |
| 12 | O | O2 | -1.3530146 | -4.2104131 | -1.4542092 |
| 13 | C | C13 | -2.2945397 | -5.0801688 | -2.1008460 |
| 14 | H | H5 | -3.3109551 | -4.6904395 | -1.9982644 |
| 15 | H | H13 | -2.1988666 | -6.0424089 | -1.5969908 |
| 16 | H | H14 | -2.0596651 | -5.1776157 | -3.1644068 |
| 17 | F | F1 | 0.9485834 | 1.5578329 | -2.7241156 |
| 18 | F | F2 | 2.6047735 | 1.6327810 | -1.2590384 |
| 19 | C | C4 | 0.5748704 | 2.0527265 | 0.9723227 |
| 20 | N | N1 | 0.9519961 | 3.0708098 | 3.5869269 |
| 21 | C | C6 | -0.4849677 | 2.5780627 | 1.7269249 |
| 22 | C | C7 | 1.8381277 | 2.0634026 | 1.5880374 |
| 23 | C | C8 | 2.0043379 | 2.5628860 | 2.8639135 |
| 24 | C | C9 | -0.2895778 | 3.0730703 | 3.0006694 |
| 25 | H | H2 | -1.4913534 | 2.6050752 | 1.3191932 |
| 26 | H | H1 | 2.7153381 | 1.6911372 | 1.0725650 |
| 27 | H | H4 | 2.9491794 | 2.6021993 | 3.3891100 |
| 28 | H | H7 | -1.0668534 | 3.4896658 | 3.6269169 |
| 29 | O | O3 | 1.1240199 | 3.5268064 | 4.7635328 |

## para-pyridine-N-oxide TS2 (TS1c)

G = -943.680817
vi= i173
S^2 = 0.6244

Cartesian Coordinates (Angstroms)

Atom X Y Z

| | | | X | Y | Z |
|---|---|---|---|---|---|
| 1 | C | C | -0.7442934 | 2.9911665 | -0.6529612 |
| 2 | C | C2 | -1.8633238 | 2.2189125 | -0.6559668 |
| 3 | C | C3 | -1.8768379 | 0.9614702 | 0.0125223 |
| 4 | C | C4 | 0.5056189 | 2.3730776 | -0.1201484 |
| 5 | C | C5 | 0.6538498 | 0.9460111 | -0.5710700 |
| 6 | H | H | -0.6970805 | 4.0005318 | -1.0464526 |
| 7 | H | H7 | -2.7503323 | 2.5120346 | -1.2138959 |
| 8 | H | H8 | -1.3399625 | 0.8184490 | 0.9404952 |
| 9 | H | H12 | 0.2045073 | 0.7258017 | -1.5310947 |
| 10 | F | F1 | 1.6088236 | 3.1107358 | -0.5101945 |
| 11 | F | F2 | 0.5285161 | 2.4437114 | 1.2816101 |
| 12 | C | C1 | 1.6190072 | 0.0436571 | -0.0843508 |
| 13 | N | N1 | 3.5077418 | -1.8693317 | 0.8212471 |
| 14 | C | C7 | 1.8384409 | -1.1962806 | -0.7581019 |
| 15 | C | C8 | 2.4134554 | 0.2521798 | 1.0854579 |
| 16 | C | C9 | 3.3234735 | -0.6801557 | 1.5006845 |
| 17 | C | C10 | 2.7505123 | -2.1100167 | -0.3093180 |
| 18 | H | H2 | 1.2716721 | -1.4303514 | -1.6542811 |
| 19 | H | H6 | 2.3060988 | 1.1634289 | 1.6585467 |
| 20 | H | H5 | 3.9580480 | -0.5786565 | 2.3711521 |
| 21 | H | H3 | 2.9607491 | -3.0624405 | -0.7773622 |
| 22 | C | C11 | -2.8422352 | -0.0682516 | -0.3784039 |
| 23 | O | O1 | -3.6244498 | 0.0175318 | -1.3119035 |
| 24 | O | O2 | -2.7589742 | -1.1547562 | 0.4310049 |
| 25 | C | C12 | -3.6624428 | -2.2222009 | 0.1130110 |
| 26 | H | H1 | -4.7003788 | -1.8921776 | 0.2148756 |
| 27 | H | H9 | -3.4417473 | -3.0160126 | 0.8275249 |
| 28 | H | H10 | -3.5038444 | -2.5707545 | -0.9116447 |
| 29 | O | O3 | 4.3553882 | -2.7273136 | 1.2290179 |

## 2-thiophene SM (9d)

Spartan'10: G = -1173.29592, vi = none
Gaussian'09: B3LYP/6-31G*, G = -1173.30199, vi = none
Gaussian'09: M05-2X/6-31G*, G = -1173.23053, vi = none

Cartesian Coordinates (Angstroms)

| | | Atom | X | Y | Z |
|---|---|---|---|---|---|
| 1 | C | C1 | 1.6447102 | 1.2814457 | -0.9375890 |
| 2 | C | C2 | 0.5212287 | 1.7458593 | -0.0943692 |
| 3 | C | C3 | 0.8411195 | 0.1957362 | -0.3104794 |
| 4 | H | H3 | -0.3426689 | 2.0754370 | -0.6688474 |
| 5 | H | H6 | 1.3040248 | -0.2653453 | 0.5585792 |
| 6 | C | C10 | -0.0785972 | -0.6272681 | -1.1025299 |
| 7 | H | H11 | -0.4640952 | -0.2034661 | -2.0283529 |
| 8 | C | C11 | -0.4802431 | -1.8616494 | -0.7637583 |
| 9 | H | H12 | -0.1388546 | -2.3582980 | 0.1397290 |
| 10 | C | C12 | -1.4198937 | -2.6011787 | -1.6329982 |
| 11 | O | O1 | -1.8957629 | -2.1937891 | -2.6757460 |
| 12 | O | O2 | -1.6963244 | -3.8212538 | -1.1117456 |
| 13 | C | C13 | -2.6006195 | -4.6235892 | -1.8848499 |
| 14 | H | H5 | -3.5553537 | -4.1076858 | -2.0178802 |

| | | | | | |
|---|---|---|---|---|---|
| 15 | H | H13 | -2.7364164 | -5.5439176 | -1.3157681 |
| 16 | H | H14 | -2.1767881 | -4.8399854 | -2.8695951 |
| 17 | F | F1 | 1.5518602 | 1.4680768 | -2.2742731 |
| 18 | F | F2 | 2.9262425 | 1.4561124 | -0.5411711 |
| 19 | C | C4 | 0.7023730 | 2.3602625 | 1.2372566 |
| 20 | C | C5 | 1.8035834 | 2.9777018 | 1.7761645 |
| 21 | C | C6 | 1.5918459 | 3.4496041 | 3.1064879 |
| 22 | C | C7 | 0.3333226 | 3.1878326 | 3.5700286 |
| 23 | H | H1 | 2.7340315 | 3.0937873 | 1.2348702 |
| 24 | H | H8 | 2.3477142 | 3.9664063 | 3.6878268 |
| 25 | H | H9 | -0.0945008 | 3.4371092 | 4.5318277 |
| 26 | S | S1 | -0.6219377 | 2.3520552 | 2.3871829 |

## 2-thiophene TS2 (TS1da)

Spartan'10: G = -1173.261131, vi = i131, S^2 = 0.5683
Gaussian'09: B3LYP/6-31G*, G = --1173.266174, vi = i334, S^2 = 0.58
Gaussian'09: M05-2X/6-31G*, G = -1173.189048, vi = i147, S^2 = 0.76

Cartesian Coordinates (Angstroms)

| | | Atom | X | Y | Z |
|---|---|---|---|---|---|
| 1 | C | C | -0.7210256 | -0.3769473 | -2.7513915 |
| 2 | C | C2 | -1.6633130 | -0.7878751 | -1.8574746 |
| 3 | C | C3 | -1.6425208 | -0.3469323 | -0.5084804 |
| 4 | C | C4 | 0.4568673 | 0.3730574 | -2.2301141 |
| 5 | C | C5 | 0.9604340 | -0.2059585 | -0.9352110 |
| 6 | H | H | -0.7617934 | -0.5913906 | -3.8136378 |
| 7 | H | H7 | -2.4261631 | -1.5096041 | -2.1435472 |
| 8 | H | H8 | -1.2822483 | 0.6385526 | -0.2471795 |
| 9 | H | H12 | 0.7937751 | -1.2673813 | -0.8066724 |
| 10 | F | F1 | 1.4705438 | 0.3718068 | -3.1716569 |
| 11 | F | F2 | 0.1531733 | 1.7304276 | -2.0604202 |
| 12 | C | C11 | -2.3398484 | -1.1152447 | 0.5241681 |
| 13 | O | O1 | -2.9346804 | -2.1662237 | 0.3423726 |
| 14 | O | O2 | -2.2429288 | -0.5139217 | 1.7384721 |
| 15 | C | C12 | -2.8807106 | -1.2132354 | 2.8149511 |
| 16 | H | H1 | -3.9557208 | -1.3034663 | 2.6345635 |
| 17 | H | H9 | -2.6914657 | -0.6153949 | 3.7074120 |
| 18 | H | H10 | -2.4588123 | -2.2161999 | 2.9276998 |
| 19 | C | C1 | 1.9188089 | 0.4334481 | -0.1364692 |
| 20 | C | C6 | 2.3884821 | 1.7569630 | -0.1691832 |
| 21 | C | C7 | 3.3512113 | 2.0350217 | 0.8188765 |
| 22 | C | C8 | 3.6266421 | 0.9527961 | 1.6286792 |
| 23 | H | H3 | 2.0271893 | 2.4727681 | -0.8952321 |
| 24 | H | H5 | 3.8264599 | 3.0018556 | 0.9428367 |
| 25 | H | H11 | 4.3129625 | 0.9081502 | 2.4642703 |
| 26 | S | S1 | 2.7146815 | -0.4450712 | 1.1823680 |

## 2_thiophene_higher_energyTS2 (TS1db)

G = -1173.26196
iv = i122
S^2 = 0.601

```
Cartesian Coordinates (Angstroms)
       Atom             X             Y             Z
    ---------    ------------- ------------- -------------

  1 C   C            -0.9278013     0.7602316    -2.6901911
  2 C   C2           -1.8672172     0.7618182    -1.7032288
  3 C   C3           -1.5086288     0.8413210    -0.3336491
  4 C   C4            0.5043701     0.6104751    -2.3065949
  5 C   C5            0.7012026    -0.3701149    -1.1866012
  6 H   H            -1.1656141     0.8285247    -3.7460605
  7 H   H7           -2.9232110     0.6526694    -1.9433104
  8 H   H8           -0.6295369     1.3839016    -0.0131775
  9 H   H12           0.0016210    -1.1964024    -1.1568814
 10 F   F1            1.2424323     0.2099791    -3.4085654
 11 F   F2            1.0677132     1.8421132    -1.9497644
 12 C   C11          -2.4546584     0.4124461     0.6970248
 13 O   O1           -3.5602301    -0.0629061     0.4887400
 14 O   O2           -1.9502468     0.6069819     1.9438422
 15 C   C12          -2.8142156     0.2091798     3.0160398
 16 H   H1           -3.7451809     0.7831206     2.9953433
 17 H   H9           -2.2589454     0.4135549     3.9324871
 18 H   H10          -3.0572818    -0.8549834     2.9429923
 19 C   C1            1.8878436    -0.5154771    -0.4587369
 20 C   C6            2.2076707    -1.5955701     0.3831959
 21 C   C7            3.4563134    -1.4720247     1.0164381
 22 C   C8            4.1089268    -0.3048071     0.6769360
 23 H   H3            1.5361313    -2.4380247     0.5102434
 24 H   H5            3.8686245    -2.2100946     1.6953957
 25 H   H11           5.0800204     0.0300601     1.0180010
 26 S   S1            3.1998985     0.6740277    -0.4199186
```

## pyrrole_N_Boc – SM (9e)

```
G = -1176.15618
iv = none

Cartesian Coordinates (Angstroms)
       Atom             X             Y             Z
    ---------    ------------- ------------- -------------

  1 C   C1            2.7535104     0.5329447    -2.5439534
  2 C   C2            1.9022163     0.9879434    -1.4270432
  3 C   C3            1.8744042    -0.5181088    -1.9545018
  4 H   H3            1.0240211     1.5436471    -1.7384084
  5 H   H6            2.3889501    -1.2127350    -1.2950455
  6 C   C10           0.6922396    -1.0140414    -2.6636953
  7 H   H11           0.2033785    -0.3375816    -3.3618949
  8 C   C11           0.1738953    -2.2418222    -2.5067290
  9 H   H12           0.6086345    -2.9725220    -1.8307627
 10 C   C12          -1.0270908    -2.6493021    -3.2620710
 11 O   O1           -1.6401087    -1.9646035    -4.0590672
 12 O   O2           -1.3777460    -3.9235125    -2.9510128
 13 C   C13          -2.5355171    -4.4200730    -3.6359457
 14 H   H5           -3.4124388    -3.8079976    -3.4065128
 15 H   H13          -2.6754402    -5.4400731    -3.2758182
 16 H   H14          -2.3762980    -4.4132677    -4.7179344
```

| | | | | | |
|---|---|---|---|---|---|
| 17 | F | F1 | 2.4675232 | 0.9968779 | -3.7830902 |
| 18 | F | F2 | 4.0957545 | 0.4116881 | -2.4057509 |
| 19 | C | C4 | 2.4334821 | 1.2902387 | -0.0860095 |
| 20 | C | C5 | 3.7232841 | 1.5276603 | 0.3230500 |
| 21 | C | C6 | 3.7171782 | 1.7451030 | 1.7362044 |
| 22 | C | C7 | 2.4272226 | 1.6350730 | 2.1677360 |
| 23 | H | H1 | 4.5820521 | 1.5408932 | -0.3309699 |
| 24 | H | H8 | 4.5748523 | 1.9620229 | 2.3583141 |
| 25 | H | H9 | 1.9875509 | 1.7354041 | 3.1455720 |
| 26 | N | N1 | 1.6202058 | 1.3551326 | 1.0643105 |
| 27 | C | C8 | 0.2226284 | 1.1991010 | 1.0943728 |
| 28 | O | O3 | -0.2133437 | 1.2468479 | 2.3591224 |
| 29 | C | C9 | -1.6575265 | 1.1251508 | 2.6728774 |
| 30 | C | C14 | -2.4290862 | 2.2782238 | 2.0251443 |
| 31 | H | H4 | -2.0009948 | 3.2405094 | 2.3261956 |
| 32 | H | H15 | -3.4707766 | 2.2507602 | 2.3630633 |
| 33 | H | H16 | -2.4136606 | 2.2073436 | 0.9367034 |
| 34 | C | C15 | -1.6721970 | 1.2482366 | 4.1979027 |
| 35 | H | H7 | -1.2663801 | 2.2142387 | 4.5152625 |
| 36 | H | H17 | -1.0770148 | 0.4526265 | 4.6573822 |
| 37 | H | H18 | -2.7006075 | 1.1669068 | 4.5639643 |
| 38 | C | C16 | -2.1679553 | -0.2495484 | 2.2317861 |
| 39 | H | H10 | -2.1369785 | -0.3572100 | 1.1464935 |
| 40 | H | H19 | -3.2033431 | -0.3743943 | 2.5670994 |
| 41 | H | H20 | -1.5664571 | -1.0443221 | 2.6862017 |
| 42 | O | O4 | -0.4520227 | 1.0465409 | 0.0974583 |

## pyyrole_N_boc_TS2 (TS1e)

G = -1176.11077
iv = i75
S^2 = 0.7190

Cartesian Coordinates (Angstroms)

| Atom | | | X | Y | Z |
|---|---|---|---|---|---|
| 1 | C | C | -2.2966442 | 2.5038283 | -0.7522378 |
| 2 | C | C2 | -3.2202595 | 1.5004654 | -0.6686955 |
| 3 | C | C3 | -2.8998157 | 0.1253002 | -0.6486436 |
| 4 | C | C4 | -0.8707789 | 2.2304137 | -1.0897300 |
| 5 | C | C5 | -0.6665939 | 1.0255474 | -1.9622623 |
| 6 | H | H | -2.5642692 | 3.5472360 | -0.6166446 |
| 7 | H | H7 | -4.2782632 | 1.7570888 | -0.6453204 |
| 8 | H | H8 | -1.9495060 | -0.2386669 | -0.2794002 |
| 9 | H | H12 | -1.3323635 | 1.0127471 | -2.8182339 |
| 10 | F | F1 | -0.3734540 | 3.3374628 | -1.7833582 |
| 11 | F | F2 | -0.0802355 | 2.1913624 | 0.0547077 |
| 12 | C | C11 | -3.9457174 | -0.8595544 | -0.9329093 |
| 13 | O | O1 | -5.1044351 | -0.6039567 | -1.2244142 |
| 14 | O | O2 | -3.4698166 | -2.1283518 | -0.8295764 |
| 15 | C | C12 | -4.4254535 | -3.1600249 | -1.0993711 |
| 16 | H | H1 | -5.2735717 | -3.0933775 | -0.4117197 |
| 17 | H | H9 | -3.8909565 | -4.1003850 | -0.9574250 |
| 18 | H | H10 | -4.7998331 | -3.0837324 | -2.1247538 |
| 19 | C | C1 | 0.5248792 | 0.2999435 | -2.1256533 |

| | | | | | |
|---|---|---|---|---|---|
| 20 | C | C6 | 1.0440565 | -0.2010755 | -3.3344732 |
| 21 | C | C7 | 2.3342710 | -0.7117037 | -3.0941433 |
| 22 | C | C8 | 2.5993629 | -0.5513321 | -1.7497834 |
| 23 | H | H3 | 0.5450331 | -0.1125159 | -4.2904793 |
| 24 | H | H5 | 3.0099156 | -1.1422242 | -3.8205963 |
| 25 | H | H11 | 3.4492929 | -0.8463660 | -1.1545859 |
| 26 | N | N1 | 1.5064027 | 0.0499485 | -1.1450145 |
| 27 | C | C9 | 1.2605679 | -0.0765626 | 0.2545129 |
| 28 | O | O3 | 2.3872856 | 0.1841217 | 0.9151857 |
| 29 | C | C10 | 2.4358210 | 0.1211324 | 2.3991176 |
| 30 | C | C13 | 1.4208616 | 1.1040404 | 2.9897361 |
| 31 | H | H13 | 1.5380529 | 2.0922187 | 2.5332578 |
| 32 | H | H14 | 0.3958318 | 0.7679561 | 2.8300664 |
| 33 | H | H15 | 1.5985712 | 1.1988611 | 4.0666651 |
| 34 | C | C14 | 3.8672117 | 0.5723141 | 2.6929274 |
| 35 | H | H4 | 4.0324401 | 1.5918928 | 2.3313039 |
| 36 | H | H16 | 4.5916532 | -0.0899223 | 2.2080669 |
| 37 | H | H17 | 4.0478971 | 0.5521972 | 3.7724520 |
| 38 | C | C15 | 2.2028363 | -1.3203461 | 2.8572202 |
| 39 | H | H6 | 1.1937030 | -1.6568408 | 2.6118931 |
| 40 | H | H18 | 2.9281275 | -1.9949784 | 2.3889698 |
| 41 | H | H19 | 2.3344957 | -1.3817917 | 3.9430077 |
| 42 | O | O4 | 0.1933966 | -0.4123696 | 0.7103349 |

## piperanal_SM (9f)

Spartan'10: G = -1041.02196, iv = none
Gaussian'09: B3LYP/6-31G*, G = -1041.030057, iv = none
Gaussian'09: M05-2X/6-31G*, G = -1040.961182, iv = none

Cartesian Coordinates (Angstroms)

| | | Atom | X | Y | Z |
|---|---|---|---|---|---|
| 1 | H | H1 | 0.3173526 | 1.8117346 | -0.4860782 |
| 2 | C | C1 | 0.0796698 | 1.9703530 | 0.5619833 |
| 3 | C | C4 | -0.5139337 | 2.3341161 | 3.2187058 |
| 4 | C | C2 | 0.2084874 | 0.9017002 | 1.4568499 |
| 5 | C | C6 | -0.3484961 | 3.2417645 | 0.9790992 |
| 6 | C | C5 | -0.6383061 | 3.3915793 | 2.3211549 |
| 7 | C | C3 | -0.0958245 | 1.0796197 | 2.8250814 |
| 8 | H | H6 | -0.4515198 | 4.0668061 | 0.2827391 |
| 9 | H | H3 | -0.0162481 | 0.2654692 | 3.5352692 |
| 10 | C | C7 | 0.7180325 | -0.4153577 | 0.9594682 |
| 11 | C | C8 | 0.1449534 | -1.7162582 | 1.3582272 |
| 12 | C | C9 | -0.1583847 | -1.2780583 | -0.0372637 |
| 13 | H | H2 | 1.7827390 | -0.4632471 | 0.7318922 |
| 14 | H | H9 | -1.1646025 | -0.8901221 | -0.1771914 |
| 15 | F | F1 | -0.8678092 | -1.7730911 | 2.2531620 |
| 16 | F | F2 | 0.9806161 | -2.7644429 | 1.5466580 |
| 17 | C | C10 | 0.4727736 | -1.9279483 | -1.1918646 |
| 18 | H | H8 | 1.4598095 | -2.3639054 | -1.0472524 |
| 19 | C | C11 | -0.0808889 | -2.0200610 | -2.4103728 |
| 20 | H | H10 | -1.0656251 | -1.6194806 | -2.6329824 |
| 21 | C | C12 | 0.6434521 | -2.6904227 | -3.5104695 |
| 22 | O | O1 | 1.7572372 | -3.1745849 | -3.4406969 |

| | | | | | |
|---|---|---|---|---|---|
| 23 | O | O2 | -0.1053900 | -2.6999901 | -4.6407824 |
| 24 | C | C13 | 0.5090769 | -3.3295069 | -5.7742877 |
| 25 | H | H7 | -0.2243694 | -3.2656527 | -6.5788836 |
| 26 | H | H11 | 0.7479377 | -4.3733616 | -5.5532355 |
| 27 | H | H12 | 1.4297941 | -2.8084978 | -6.0514828 |
| 28 | O | O3 | -0.8886457 | 2.7475398 | 4.4747810 |
| 29 | C | C14 | -1.1176431 | 4.1571614 | 4.3678767 |
| 30 | H | H13 | -0.3195540 | 4.6995025 | 4.8933865 |
| 31 | H | H14 | -2.0979599 | 4.3997452 | 4.7885223 |
| 32 | O | O4 | -1.0967313 | 4.5068976 | 2.9779849 |

## piperanal_TS2 (TS1f)

Spartan'10: G = -1040.98527, iv = i123, S^2 = 0.4875
Gaussian'09: B3LYP/6-31G*, G = -1040.991312, iv = i123 S^2 = 0.49
Gaussian'09: M05-2X/6-31G*, G = -1040.916448, iv = i154, S^2 = 0.70

Cartesian Coordinates (Angstroms)

| | | Atom | X | Y | Z |
|---|---|---|---|---|---|
| 1 | C | C | -1.9415125 | 2.8459054 | -0.1145085 |
| 2 | C | C2 | -2.8968928 | 1.8980800 | 0.1045137 |
| 3 | C | C3 | -2.5560611 | 0.6313994 | 0.6442402 |
| 4 | C | C4 | -0.5213291 | 2.4249366 | 0.0232476 |
| 5 | C | C5 | -0.2785848 | 1.0938538 | -0.6265670 |
| 6 | H | H | -2.1537181 | 3.8659395 | -0.4147181 |
| 7 | H | H7 | -3.9327982 | 2.0688843 | -0.1819285 |
| 8 | H | H8 | -1.7420745 | 0.5235062 | 1.3476045 |
| 9 | H | H12 | -0.9914780 | 0.8153500 | -1.3903371 |
| 10 | F | F1 | 0.3209903 | 3.3806811 | -0.5228531 |
| 11 | F | F2 | -0.1367627 | 2.3813647 | 1.3763306 |
| 12 | C | C1 | 0.9674345 | 0.4091213 | -0.6132428 |
| 13 | C | C6 | 3.3393187 | -1.0419376 | -0.7024803 |
| 14 | C | C7 | 1.1304562 | -0.7070957 | -1.4805967 |
| 15 | C | C8 | 2.0508659 | 0.7887275 | 0.2442712 |
| 16 | C | C9 | 3.2035145 | 0.0544406 | 0.1585045 |
| 17 | C | C10 | 2.3096788 | -1.4497963 | -1.5357170 |
| 18 | H | H2 | 0.3006140 | -0.9867083 | -2.1230616 |
| 19 | H | H6 | 1.9583437 | 1.6244971 | 0.9238368 |
| 20 | H | H3 | 2.4233575 | -2.3001809 | -2.1987607 |
| 21 | C | C11 | -3.4612854 | -0.5051960 | 0.4740267 |
| 22 | O | O1 | -4.5012178 | -0.4946765 | -0.1664901 |
| 23 | O | O2 | -3.0011475 | -1.6060197 | 1.1264093 |
| 24 | C | C12 | -3.8252873 | -2.7716733 | 1.0064332 |
| 25 | H | H1 | -4.8175953 | -2.5900216 | 1.4297484 |
| 26 | H | H9 | -3.3098968 | -3.5550517 | 1.5638819 |
| 27 | H | H10 | -3.9401814 | -3.0590914 | -0.0429790 |
| 28 | C | C16 | 5.2862127 | -0.7515467 | 0.3636972 |
| 29 | H | H16 | 5.6638544 | -1.3654739 | 1.1871797 |
| 30 | H | H17 | 6.1072607 | -0.2495450 | -0.1631722 |
| 31 | O | O3 | 4.5775514 | -1.5919137 | -0.5642076 |
| 32 | O | O4 | 4.3683699 | 0.2192409 | 0.8676951 |

## Vinyl_SM (9g)

G = -698.896923
iv = none

Cartesian Coordinates (Angstroms)

| | Atom | | X | Y | Z |
|---|---|---|---|---|---|
| 1 | C | C1 | 1.9355502 | 1.7969259 | -0.3459348 |
| 2 | C | C2 | 0.8307819 | 2.1575483 | 0.5704414 |
| 3 | C | C3 | 1.1920029 | 0.6392453 | 0.2183687 |
| 4 | H | H3 | -0.0759566 | 2.5122382 | 0.0862674 |
| 5 | H | H6 | 1.6963533 | 0.1125771 | 1.0252585 |
| 6 | C | C10 | 0.2552858 | -0.1365557 | -0.6009850 |
| 7 | H | H11 | -0.1543285 | 0.3425355 | -1.4885605 |
| 8 | C | C11 | -0.1335964 | -1.3913708 | -0.3282800 |
| 9 | H | H12 | 0.2329983 | -1.9394131 | 0.5347714 |
| 10 | C | C12 | -1.0945064 | -2.0825309 | -1.2131158 |
| 11 | O | O1 | -1.5953320 | -1.6208579 | -2.2209946 |
| 12 | O | O2 | -1.3607729 | -3.3286259 | -0.7502454 |
| 13 | C | C13 | -2.2872051 | -4.0875934 | -1.5403591 |
| 14 | H | H5 | -3.2430000 | -3.5630588 | -1.6222382 |
| 15 | H | H13 | -2.4121995 | -5.0357462 | -1.0162391 |
| 16 | H | H14 | -1.8896012 | -4.2541816 | -2.5455875 |
| 17 | F | F1 | 1.8126274 | 2.0764637 | -1.6634147 |
| 18 | F | F2 | 3.2135469 | 1.9930861 | 0.0537993 |
| 19 | C | C4 | 1.0896415 | 2.6534012 | 1.9367450 |
| 20 | H | H2 | 2.0583814 | 2.3967857 | 2.3621727 |
| 21 | C | C5 | 0.2219098 | 3.3789314 | 2.6464829 |
| 22 | H | H1 | 0.4602017 | 3.7221319 | 3.6487135 |
| 23 | H | H4 | -0.7527828 | 3.6580640 | 2.2529337 |

## Vinyl_TS2 LHS (TS1g)

G = -698.857875
iv = i198
S^2 = 0.6714

Cartesian Coordinates (Angstroms)

| | Atom | | X | Y | Z |
|---|---|---|---|---|---|
| 1 | C | C | 0.1652595 | 2.6452115 | -0.3143155 |
| 2 | C | C2 | -0.9584813 | 1.8809569 | -0.3019228 |
| 3 | C | C3 | -0.9637138 | 0.5905294 | 0.2988038 |
| 4 | C | C4 | 1.4504042 | 2.0089192 | 0.1090996 |
| 5 | C | C5 | 1.5736222 | 0.5798217 | -0.3410965 |
| 6 | H | H | 0.1907607 | 3.6766667 | -0.6489816 |
| 7 | H | H7 | -1.8694255 | 2.2159214 | -0.7940528 |
| 8 | H | H8 | -0.3786445 | 0.3846912 | 1.1849748 |
| 9 | H | H12 | 1.1669910 | 0.3487995 | -1.3194338 |
| 10 | F | F1 | 2.5134097 | 2.7425610 | -0.3921617 |
| 11 | F | F2 | 1.6046460 | 2.0673940 | 1.4925765 |
| 12 | C | C11 | -1.9729073 | -0.3973458 | -0.0922705 |
| 13 | O | O1 | -2.8112126 | -0.2389039 | -0.9652907 |

| | | | | | |
|---|---|---|---|---|---|
| 14 | O | O2 | -1.8623287 | -1.5331343 | 0.6422082 |
| 15 | C | C12 | -2.8063532 | -2.5626474 | 0.3167219 |
| 16 | H | H1 | -3.8302743 | -2.2111786 | 0.4741522 |
| 17 | H | H9 | -2.5784639 | -3.3924022 | 0.9872065 |
| 18 | H | H10 | -2.6972065 | -2.8709552 | -0.7274202 |
| 19 | C | C1 | 2.4851927 | -0.3044672 | 0.2691289 |
| 20 | H | H3 | 2.9377797 | 0.0188533 | 1.2042950 |
| 21 | C | C6 | 2.7966479 | -1.5403617 | -0.2250625 |
| 22 | H | H2 | 3.4848028 | -2.2007601 | 0.2926869 |
| 23 | H | H4 | 2.3594953 | -1.9081690 | -1.1498455 |

### 3-thiophene_SM (9h)

G = -1173.29649
iv = none

Cartesian Coordinates (Angstroms)

| Atom | | | X | Y | Z |
|---|---|---|---|---|---|
| 1 | C | C1 | 1.4442820 | 1.5404046 | -1.0097879 |
| 2 | C | C2 | 0.3927582 | 1.8764466 | -0.0237970 |
| 3 | C | C3 | 0.8288766 | 0.3719452 | -0.3204143 |
| 4 | H | H3 | -0.5607177 | 2.1328018 | -0.4786628 |
| 5 | H | H6 | 1.4362987 | -0.0670530 | 0.4676500 |
| 6 | C | C10 | -0.1107572 | -0.5159186 | -1.0134407 |
| 7 | H | H11 | -0.6603421 | -0.1039428 | -1.8581155 |
| 8 | C | C11 | -0.3435135 | -1.7926382 | -0.6731372 |
| 9 | H | H12 | 0.1711044 | -2.2773250 | 0.1516008 |
| 10 | C | C12 | -1.3258436 | -2.5989270 | -1.4276396 |
| 11 | O | O1 | -1.9984877 | -2.2113527 | -2.3641198 |
| 12 | O | O2 | -1.3897156 | -3.8597859 | -0.9339553 |
| 13 | C | C13 | -2.3181495 | -4.7285856 | -1.5989953 |
| 14 | H | H5 | -3.3350508 | -4.3325827 | -1.5279792 |
| 15 | H | H13 | -2.2432837 | -5.6872218 | -1.0845376 |
| 16 | H | H14 | -2.0553539 | -4.8368853 | -2.6549552 |
| 17 | F | F1 | 1.1825558 | 1.7359006 | -2.3226269 |
| 18 | F | F2 | 2.7405856 | 1.8349551 | -0.7532548 |
| 19 | C | C4 | 0.6386102 | 2.4940989 | 1.3012228 |
| 20 | C | C5 | 1.8650771 | 2.4391855 | 2.0495963 |
| 21 | C | C6 | 1.7847172 | 3.0813387 | 3.2512183 |
| 22 | S | S1 | 0.2168841 | 3.7791875 | 3.5053528 |
| 23 | C | C8 | -0.3357356 | 3.1901481 | 1.9737064 |
| 24 | H | H1 | 2.7672056 | 1.9564327 | 1.6953535 |
| 25 | H | H8 | 2.5584428 | 3.1962145 | 3.9983072 |
| 26 | H | H15 | -1.3504474 | 3.3831587 | 1.6514110 |

### 2-thiophene TS2 alt (TS1h)

G = -1173.25727
iv = i114
S^2 = 0.5844

Cartesian Coordinates (Angstroms)

| Atom | X | Y | Z |
|---|---|---|---|

| | | | | | |
|---|---|---|---|---|---|
| 1 | C | C | -0.3331252 | 2.7439182 | -0.5242953 |
| 2 | C | C2 | -1.4517833 | 1.9650401 | -0.5727216 |
| 3 | C | C3 | -1.5103739 | 0.7180630 | 0.0976444 |
| 4 | C | C4 | 0.9029740 | 2.1508129 | 0.0594263 |
| 5 | C | C5 | 1.1146943 | 0.7374396 | -0.3968356 |
| 6 | H | H | -0.2825728 | 3.7530236 | -0.9182330 |
| 7 | H | H7 | -2.3112866 | 2.2592107 | -1.1717697 |
| 8 | H | H8 | -0.9585452 | 0.5456332 | 1.0118178 |
| 9 | H | H12 | 0.6711376 | 0.4815216 | -1.3498593 |
| 10 | F | F1 | 2.0021802 | 2.9255137 | -0.2725910 |
| 11 | F | F2 | 0.8715525 | 2.1990947 | 1.4621430 |
| 12 | C | C11 | -2.5122966 | -0.2777141 | -0.2889431 |
| 13 | O | O1 | -3.3033639 | -0.1635621 | -1.2119161 |
| 14 | O | O2 | -2.4575935 | -1.3702203 | 0.5160657 |
| 15 | C | C12 | -3.3994409 | -2.4043657 | 0.2026418 |
| 16 | H | H1 | -4.4248487 | -2.0408846 | 0.3169501 |
| 17 | H | H9 | -3.1984520 | -3.2090659 | 0.9108671 |
| 18 | H | H10 | -3.2635384 | -2.7531014 | -0.8251504 |
| 19 | C | C1 | 2.1319687 | -0.1093191 | 0.1123631 |
| 20 | C | C6 | 2.5032706 | -1.3622827 | -0.5148334 |
| 21 | C | C7 | 3.4928028 | -2.0212654 | 0.1397115 |
| 22 | S | S1 | 4.0211354 | -1.1473099 | 1.5576573 |
| 23 | C | C9 | 2.8912283 | 0.1141277 | 1.2640703 |
| 24 | H | H3 | 2.0309517 | -1.7285710 | -1.4201138 |
| 25 | H | H5 | 3.9542148 | -2.9658622 | -0.1152083 |
| 26 | H | H14 | 2.8191103 | 0.9601255 | 1.9311122 |

## 2-thiazole SM (9i)

G = -1189.35451
iv = none

Cartesian Coordinates (Angstroms)

| | | Atom | X | Y | Z |
|---|---|---|---|---|---|
| 1 | C | C1 | 1.4013058 | 1.6826903 | -0.9862793 |
| 2 | C | C2 | 0.3481207 | 1.9349898 | 0.0400251 |
| 3 | C | C3 | 0.8159698 | 0.4777131 | -0.3397660 |
| 4 | H | H6 | 1.4365614 | 0.0346426 | 0.4351354 |
| 5 | C | C10 | -0.1119487 | -0.4004681 | -1.0663144 |
| 6 | H | H11 | -0.6646054 | 0.0283623 | -1.9004368 |
| 7 | C | C11 | -0.3216483 | -1.6902861 | -0.7672656 |
| 8 | H | H12 | 0.1965106 | -2.1904600 | 0.0457575 |
| 9 | C | C12 | -1.2854305 | -2.4901029 | -1.5544828 |
| 10 | O | O1 | -1.9512959 | -2.0857909 | -2.4883978 |
| 11 | O | O2 | -1.3389844 | -3.7630262 | -1.0940432 |
| 12 | C | C13 | -2.2476632 | -4.6264585 | -1.7931289 |
| 13 | H | H5 | -3.2687791 | -4.2396457 | -1.7340227 |
| 14 | H | H13 | -2.1742931 | -5.5940744 | -1.2957037 |
| 15 | H | H14 | -1.9635389 | -4.7121801 | -2.8456385 |
| 16 | F | F1 | 1.0897515 | 1.9454442 | -2.2761353 |
| 17 | F | F2 | 2.6798604 | 2.0163755 | -0.7391868 |
| 18 | C | C4 | 0.6871187 | 2.4484393 | 1.3825995 |
| 19 | N | N1 | 1.5569002 | 1.9082761 | 2.1889631 |

```
 20 C   C7            0.9016926     3.7544921     3.4403495
 21 H   H9            0.8278894     4.4632764     4.2532685
 22 S   S1           -0.0542307     3.9215840     2.0031029
 23 H   H3           -0.6141778     2.2362664    -0.3652607
 24 C   C6            1.6824389     2.6395753     3.3463609
 25 H   H7            2.3724759     2.3003654     4.1105000
```

## 2-thiazole TS2 (TS1ia)

```
G = -1189.31604
iv = i158
S^2 = 0.6401

Cartesian Coordinates (Angstroms)
       Atom              X             Y             Z
    ---------     ------------- ------------- -------------

  1 C   C            -0.8624600     0.6458233    -2.6809721
  2 C   C2           -1.7945142     0.6484678    -1.6885283
  3 C   C3           -1.4196477     0.7346465    -0.3224604
  4 C   C4            0.5731559     0.5015687    -2.2988314
  5 C   C5            0.7688284    -0.4679819    -1.1699536
  6 H   H            -1.1050971     0.7031453    -3.7364239
  7 H   H7           -2.8520204     0.5322726    -1.9168489
  8 H   H8           -0.5487926     1.2963848    -0.0125430
  9 H   H12           0.1063398    -1.3231256    -1.1382335
 10 F   F1            1.3102300     0.0940487    -3.3953137
 11 F   F2            1.1248412     1.7403949    -1.9531783
 12 C   C11          -2.3513200     0.2959998     0.7223360
 13 O   O1           -3.4476576    -0.2001740     0.5203581
 14 O   O2           -1.8421206     0.5130008     1.9592936
 15 C   C12          -2.6828069     0.0961690     3.0445494
 16 H   H1           -3.6349998     0.6336831     3.0225873
 17 H   H9           -2.1283804     0.3339141     3.9529242
 18 H   H10          -2.8823985    -0.9775536     2.9854094
 19 C   C1            1.9504425    -0.5849422    -0.4186831
 20 N   N1            2.1984549    -1.6348782     0.3707388
 21 C   C8            4.1034809    -0.3580502     0.7303876
 22 H   H11           5.0684951    -0.0693435     1.1245010
 23 S   S1            3.2435322     0.6444020    -0.3730350
 24 C   C7            3.3860264    -1.5066663     1.0000569
 25 H   H4            3.7183885    -2.2912055     1.6718630
```

## thiazole_higher_energyTS2 (TS1ib)

```
G = -1189.31128
iv = i172
S^2 = 0.6211

Cartesian Coordinates (Angstroms)
       Atom              X             Y             Z
    ---------     ------------- ------------- -------------

  1 C   C            -0.9124198     0.5660171    -2.6688343
  2 C   C2           -1.8409100     0.5476441    -1.6746237
  3 C   C3           -1.4526669     0.6770132    -0.3132767
```

| | | | | | |
|---|---|---|---|---|---|
| 4 | C | C4 | 0.5318908 | 0.5151246 | -2.2727045 |
| 5 | C | C5 | 0.7450768 | -0.4716982 | -1.1503779 |
| 6 | H | H | -1.1557524 | 0.5780877 | -3.7256670 |
| 7 | H | H7 | -2.8933373 | 0.3752347 | -1.8897007 |
| 8 | H | H8 | -0.6112304 | 1.2936434 | -0.0270815 |
| 9 | H | H12 | 0.0711162 | -1.3178375 | -1.1388929 |
| 10 | F | F1 | 1.2984462 | 0.1460785 | -3.3609117 |
| 11 | F | F2 | 0.9821656 | 1.7714234 | -1.9150056 |
| 12 | C | C11 | -2.3479460 | 0.2107881 | 0.7509096 |
| 13 | O | O1 | -3.4172720 | -0.3501472 | 0.5736335 |
| 14 | O | O2 | -1.8381305 | 0.4805843 | 1.9781156 |
| 15 | C | C12 | -2.6495697 | 0.0533101 | 3.0811159 |
| 16 | H | H1 | -3.6204748 | 0.5563684 | 3.0622352 |
| 17 | H | H9 | -2.0932806 | 0.3274638 | 3.9780292 |
| 18 | H | H10 | -2.8124816 | -1.0275786 | 3.0439963 |
| 19 | C | C1 | 1.9449539 | -0.5742326 | -0.4236465 |
| 20 | N | N1 | 2.9420037 | 0.3050315 | -0.3933605 |
| 21 | C | C8 | 3.7444018 | -1.3024687 | 1.0843730 |
| 22 | H | H11 | 4.4074900 | -1.7902521 | 1.7856196 |
| 23 | S | S1 | 2.2310906 | -1.9904853 | 0.6387774 |
| 24 | C | C7 | 3.9330111 | -0.1008447 | 0.4304792 |
| 25 | H | H4 | 4.8138256 | 0.5217320 | 0.5467991 |

## Ph LHS SM (control compound) (9j)

Spartan'10: G = -852.507737, iv = none
Gaussian'09: B3LYP/6-31G*, G = -852.513911, iv = none
Gaussian'09: M05-2X/6-31G*, G = -852.454583, iv = none

Cartesian Coordinates (Angstroms)

| Atom | | | X | Y | Z |
|---|---|---|---|---|---|
| 1 | H | H1 | -0.7342992 | 2.1292350 | -0.6038797 |
| 2 | C | C1 | -0.6896550 | 2.5456381 | 0.3996721 |
| 3 | C | C4 | -0.5788215 | 3.6046272 | 2.9736287 |
| 4 | C | C2 | 0.1528229 | 1.9539030 | 1.3524642 |
| 5 | C | C6 | -1.4665871 | 3.6544153 | 0.7292205 |
| 6 | C | C5 | -1.4135141 | 4.1868572 | 2.0197807 |
| 7 | C | C3 | 0.2019187 | 2.4950669 | 2.6438137 |
| 8 | H | H6 | -2.1142921 | 4.1021150 | -0.0198156 |
| 9 | H | H5 | -2.0191435 | 5.0512131 | 2.2786045 |
| 10 | H | H3 | 0.8466623 | 2.0454541 | 3.3913082 |
| 11 | H | H4 | -0.5318796 | 4.0135924 | 3.9793780 |
| 12 | C | C7 | 1.0044769 | 0.7904903 | 0.9510633 |
| 13 | C | C8 | 1.3092637 | -0.3611739 | 1.8231770 |
| 14 | C | C9 | 0.3451828 | -0.6317107 | 0.7160262 |
| 15 | H | H9 | -0.6950119 | -0.6814867 | 1.0296629 |
| 16 | F | F1 | 0.8550859 | -0.4026367 | 3.0973066 |
| 17 | F | F2 | 2.5471373 | -0.9057421 | 1.7618348 |
| 18 | C | C10 | 0.7256057 | -1.4333489 | -0.4525928 |
| 19 | H | H8 | 1.7776668 | -1.4516307 | -0.7317858 |
| 20 | C | C11 | -0.1366627 | -2.1405728 | -1.1983779 |
| 21 | H | H10 | -1.1996782 | -2.1775237 | -0.9780074 |
| 22 | C | C12 | 0.3401656 | -2.9115132 | -2.3652549 |
| 23 | O | O1 | 1.4898785 | -2.9767628 | -2.7568938 |

```
 24 O   O2          -0.6913356    -3.5547476    -2.9657551
 25 C   C13         -0.3365797    -4.3399461    -4.1129601
 26 H   H7           0.3705291    -5.1270044    -3.8365732
 27 H   H11          0.1186948    -3.7122448    -4.8840365
 28 H   H12         -1.2707537    -4.7727264    -4.4725849
 29 H   H2           1.7931229     1.0081637     0.2315763
```

## Ph_LHS_TS2 (TS1j)

```
Spartan'10: G = -852.467491, iv = i124, S^2 = 0.5609
Gaussian'09: B3LYP/6-31G*, G = -852.471539, iv = i139, S^2 = 0.72
Gaussian'09: M05-2X/6-31G*, G = -852.407064, iv = i150 S^2 = 0.73

 Cartesian Coordinates (Angstroms)
       Atom             X             Y             Z
    ---------     ------------- ------------- -------------

  1 C   C           -0.7938224     2.9281295    -0.4973567
  2 C   C2          -1.9169588     2.1548519    -0.5208385
  3 C   C3          -1.9447433     0.8899069     0.1188859
  4 C   C4           0.4554569     2.3058113     0.0257641
  5 C   C5           0.6396500     0.9126099    -0.5034969
  6 H   H           -0.7489920     3.9452945    -0.8705278
  7 H   H7          -2.7990824     2.4628835    -1.0784486
  8 H   H8          -1.3634344     0.6999835     1.0109197
  9 H   H12          0.1318702     0.7046588    -1.4362973
 10 F   F1           1.5526349     3.0842611    -0.3012959
 11 F   F2           0.4548498     2.2993475     1.4302410
 12 C   C1           1.6981159     0.0337646    -0.1244960
 13 C   C6           3.7528699    -1.7843479     0.5356695
 14 C   C7           1.9221078    -1.1367612    -0.8981062
 15 C   C8           2.5370955     0.2579721     1.0002180
 16 C   C9           3.5497938    -0.6376134     1.3129267
 17 C   C10          2.9311417    -2.0302287    -0.5711578
 18 H   H2           1.2868288    -1.3240531    -1.7604162
 19 H   H6           2.3799547     1.1370129     1.6122318
 20 H   H5           4.1864329    -0.4470045     2.1726196
 21 H   H3           3.0852933    -2.9185318    -1.1775979
 22 H   H4           4.5447429    -2.4828045     0.7915831
 23 C   C11         -2.9550719    -0.1019472    -0.2577349
 24 O   O1          -3.7794569     0.0322524    -1.1481949
 25 O   O2          -2.8655294    -1.2148241     0.5148987
 26 C   C12         -3.8127241    -2.2457971     0.2059573
 27 H   H1          -4.8355106    -1.8867747     0.3524122
 28 H   H9          -3.5920855    -3.0635919     0.8932747
 29 H   H10         -3.7014272    -2.5744602    -0.8316365
```

## bis_Ph_SM (9k)

```
G = -1083.47906
iv = none

 Cartesian Coordinates (Angstroms)
       Atom             X             Y             Z
    ---------     ------------- ------------- -------------
```

| | | | | | |
|---|---|---|---|---|---|
| 1 | C | C1 | 2.1401892 | -0.0956954 | -0.5030615 |
| 2 | C | C2 | 1.0084305 | 0.6541421 | 0.1013241 |
| 3 | C | C3 | 1.0969993 | -0.9312616 | 0.1653649 |
| 4 | H | H6 | 1.3824353 | -1.3078350 | 1.1445602 |
| 5 | C | C4 | 1.2610998 | 1.3842374 | 1.3998916 |
| 6 | C | C5 | 1.6621778 | 2.8368121 | 3.7687618 |
| 7 | C | C6 | 0.5771414 | 1.0383205 | 2.5708181 |
| 8 | C | C7 | 2.1514488 | 2.4655458 | 1.4290384 |
| 9 | C | C8 | 2.3510230 | 3.1865204 | 2.6055784 |
| 10 | C | C9 | 0.7744797 | 1.7603828 | 3.7486637 |
| 11 | H | H4 | -0.1128299 | 0.1982990 | 2.5605250 |
| 12 | H | H7 | 2.6904649 | 2.7407226 | 0.5268287 |
| 13 | H | H8 | 3.0475027 | 4.0206999 | 2.6144128 |
| 14 | H | H9 | 0.2348075 | 1.4804399 | 4.6494368 |
| 15 | C | C10 | 0.1997949 | -1.7833133 | -0.6270916 |
| 16 | H | H11 | -0.0529765 | -1.4603042 | -1.6341065 |
| 17 | C | C11 | -0.3192736 | -2.9411307 | -0.1905993 |
| 18 | H | H12 | -0.1109823 | -3.3375552 | 0.7992122 |
| 19 | C | C12 | -1.2026816 | -3.7385484 | -1.0670984 |
| 20 | O | O1 | -1.5445314 | -3.4423954 | -2.1960226 |
| 21 | O | O2 | -1.5992964 | -4.8756654 | -0.4422651 |
| 22 | C | C13 | -2.4590919 | -5.7243013 | -1.2159658 |
| 23 | H | H5 | -3.3938217 | -5.2092327 | -1.4552872 |
| 24 | H | H13 | -2.6519025 | -6.5966411 | -0.5904149 |
| 25 | H | H14 | -1.9711465 | -6.0186838 | -2.1489851 |
| 26 | F | F1 | 2.2560587 | -0.1617158 | -1.8507333 |
| 27 | F | F2 | 3.3716170 | -0.0205447 | 0.0501528 |
| 28 | H | H16 | 1.8186444 | 3.3988195 | 4.6855521 |
| 29 | C | C14 | -0.0221504 | 1.2954522 | -0.7972292 |
| 30 | C | C15 | -1.9532314 | 2.5840539 | -2.3773674 |
| 31 | C | C16 | -1.3881705 | 1.1209839 | -0.5374988 |
| 32 | C | C17 | 0.3650011 | 2.1252734 | -1.8574405 |
| 33 | C | C18 | -0.5955412 | 2.7651048 | -2.6420020 |
| 34 | C | C19 | -2.3475632 | 1.7589318 | -1.3221684 |
| 35 | H | H2 | -1.7000848 | 0.4771947 | 0.2803560 |
| 36 | H | H1 | 1.4189827 | 2.2629083 | -2.0781994 |
| 37 | H | H3 | -0.2800252 | 3.4018179 | -3.4642146 |
| 38 | H | H10 | -3.4028383 | 1.6083512 | -1.1113529 |
| 39 | H | H15 | -2.7001597 | 3.0798098 | -2.9913732 |

### bis_Ph_TS2 (TS1k)

G = -1083.43796
iv = i170
S^2 = 0.5387

Cartesian Coordinates (Angstroms)

| | | Atom | X | Y | Z |
|---|---|---|---|---|---|
| 1 | C | C | -0.4905029 | 2.9644064 | 0.2699805 |
| 2 | C | C2 | -1.6739456 | 2.3000484 | 0.1811172 |
| 3 | C | C3 | -1.8234284 | 0.9930522 | 0.7115034 |
| 4 | C | C4 | 0.7007691 | 2.2204344 | 0.7848070 |
| 5 | C | C5 | 0.8020209 | 0.7758907 | 0.2922367 |
| 6 | H | H | -0.3628539 | 4.0113164 | 0.0158601 |

| | | | | | |
|---|---|---|---|---|---|
| 7 | H | H7 | -2.5148493 | 2.7325649 | -0.3574474 |
| 8 | H | H8 | -1.3254011 | 0.6920489 | 1.6207777 |
| 9 | F | F1 | 1.8636038 | 2.8958172 | 0.4238450 |
| 10 | F | F2 | 0.7094526 | 2.2676910 | 2.1728991 |
| 11 | C | C1 | 1.4274022 | -0.2471139 | 1.1415429 |
| 12 | C | C6 | 2.6632180 | -2.2619875 | 2.7066499 |
| 13 | C | C7 | 2.3919579 | -1.1262335 | 0.5910474 |
| 14 | C | C8 | 1.1150697 | -0.4092438 | 2.5134199 |
| 15 | C | C9 | 1.7196106 | -1.4031744 | 3.2752391 |
| 16 | C | C10 | 2.9998396 | -2.1130276 | 1.3598395 |
| 17 | H | H2 | 2.6831985 | -1.0061205 | -0.4461451 |
| 18 | H | H6 | 0.3847455 | 0.2361774 | 2.9804870 |
| 19 | H | H5 | 1.4477300 | -1.5099628 | 4.3216431 |
| 20 | H | H3 | 3.7451214 | -2.7611627 | 0.9068595 |
| 21 | H | H4 | 3.1324323 | -3.0357165 | 3.3080647 |
| 22 | C | C11 | -2.8669953 | 0.1051841 | 0.2041829 |
| 23 | O | O1 | -3.6526536 | 0.3696869 | -0.6919707 |
| 24 | O | O2 | -2.8577312 | -1.0884394 | 0.8590324 |
| 25 | C | C12 | -3.8453877 | -2.0292641 | 0.4217172 |
| 26 | H | H1 | -4.8524776 | -1.6265121 | 0.5631762 |
| 27 | H | H9 | -3.6987149 | -2.9175849 | 1.0380379 |
| 28 | H | H10 | -3.7106018 | -2.2706167 | -0.6369425 |
| 29 | C | C13 | 0.7059762 | 0.5609448 | -1.1515914 |
| 30 | C | C14 | 0.4088753 | 0.1093913 | -3.9293928 |
| 31 | C | C15 | 0.9236744 | 1.5965010 | -2.0893611 |
| 32 | C | C16 | 0.3436510 | -0.7135608 | -1.6564080 |
| 33 | C | C17 | 0.1940066 | -0.9325095 | -3.0176990 |
| 34 | C | C18 | 0.7744233 | 1.3681896 | -3.4571869 |
| 35 | H | H11 | 1.2584263 | 2.5672437 | -1.7492165 |
| 36 | H | H12 | 0.1380548 | -1.5165279 | -0.9558351 |
| 37 | H | H13 | -0.1056006 | -1.9144923 | -3.3740211 |
| 38 | H | H14 | 0.9565966 | 2.1808425 | -4.1552514 |
| 39 | H | H15 | 0.2912871 | -0.0641812 | -4.9954973 |

## alkynyl_LHS_SM (9l)

G = -697.662199
iv = none

Cartesian Coordinates (Angstroms)

| Atom | | | X | Y | Z |
|---|---|---|---|---|---|
| 1 | C | C1 | 2.0424676 | 1.9684931 | -0.1391170 |
| 2 | C | C2 | 0.8725035 | 2.4962424 | 0.6162951 |
| 3 | C | C3 | 1.1493632 | 0.9340715 | 0.4498497 |
| 4 | H | H3 | 0.0609649 | 2.8669018 | -0.0070648 |
| 5 | H | H6 | 1.5244582 | 0.4683830 | 1.3570881 |
| 6 | C | C10 | 0.2426017 | 0.1419043 | -0.3899421 |
| 7 | H | H11 | -0.0646980 | 0.5667251 | -1.3437475 |
| 8 | C | C11 | -0.2303187 | -1.0678896 | -0.0571690 |
| 9 | H | H12 | 0.0360504 | -1.5606142 | 0.8732704 |
| 10 | C | C12 | -1.1469251 | -1.7848967 | -0.9704422 |
| 11 | O | O1 | -1.5497743 | -1.3718325 | -2.0409954 |
| 12 | O | O2 | -1.4935214 | -2.9877721 | -0.4535502 |
| 13 | C | C13 | -2.3834903 | -3.7677120 | -1.2660675 |

| | | | | | |
|---|---|---|---|---|---|
| 14 | H | H5 | -3.3175284 | -3.2268389 | -1.4405158 |
| 15 | H | H13 | -2.5682923 | -4.6831435 | -0.7031171 |
| 16 | H | H14 | -1.9222194 | -3.9957741 | -2.2309570 |
| 17 | F | F1 | 2.0512871 | 2.1302983 | -1.4803672 |
| 18 | F | F2 | 3.2825906 | 2.1200466 | 0.3600670 |
| 19 | C | C4 | 1.0217110 | 3.0973457 | 1.9084994 |
| 20 | H | H1 | 1.2541083 | 4.0534359 | 3.9587574 |
| 21 | C | C5 | 1.1386614 | 3.6026259 | 2.9992259 |

## alkynyl_LHS_TS2 (TS1l)

G = -697.620962
iv = i184
S^2 = 0.6758

Cartesian Coordinates (Angstroms)

| Atom | | | X | Y | Z |
|---|---|---|---|---|---|
| 1 | C | C | 0.4165731 | 2.5068791 | -0.3637114 |
| 2 | C | C2 | -0.6902111 | 1.7284846 | -0.5113690 |
| 3 | C | C3 | -0.7689538 | 0.4441358 | 0.0901598 |
| 4 | C | C4 | 1.6325214 | 1.8895419 | 0.2525525 |
| 5 | C | C5 | 1.8367364 | 0.4685151 | -0.2135836 |
| 6 | H | H | 0.4814311 | 3.5345224 | -0.7045805 |
| 7 | H | H7 | -1.5231515 | 2.0493445 | -1.1332736 |
| 8 | H | H8 | -0.2830066 | 0.2352376 | 1.0335260 |
| 9 | H | H12 | 1.5620280 | 0.2510525 | -1.2394046 |
| 10 | F | F1 | 2.7448939 | 2.6456606 | -0.0628903 |
| 11 | F | F2 | 1.5608009 | 1.9131080 | 1.6360459 |
| 12 | C | C11 | -1.7408621 | -0.5420694 | -0.3985955 |
| 13 | O | O1 | -2.5033990 | -0.3717384 | -1.3355925 |
| 14 | O | O2 | -1.6893955 | -1.6811234 | 0.3336227 |
| 15 | C | C12 | -2.6113098 | -2.7077900 | -0.0591668 |
| 16 | H | H1 | -3.6404973 | -2.3981585 | 0.1488279 |
| 17 | H | H9 | -2.3494573 | -3.5814320 | 0.5392686 |
| 18 | H | H10 | -2.5162890 | -2.9258022 | -1.1264765 |
| 19 | C | C1 | 2.6781292 | -0.3978923 | 0.4613271 |
| 20 | H | H2 | 4.0143079 | -1.8713020 | 1.5919161 |
| 21 | C | C6 | 3.3891107 | -1.1891739 | 1.0613974 |

## 4-pyridine_LHS_SM (9m)

G = -868.554947
iv = none

Cartesian Coordinates (Angstroms)

| Atom | | | X | Y | Z |
|---|---|---|---|---|---|
| 1 | C | C1 | 1.7379906 | 1.0533701 | -1.0125123 |
| 2 | C | C2 | 0.5948548 | 1.5544215 | -0.2227780 |
| 3 | C | C3 | 0.8612826 | 0.0062370 | -0.4070529 |
| 4 | H | H3 | -0.2344211 | 1.9188458 | -0.8277523 |
| 5 | H | H6 | 1.2755361 | -0.4729380 | 0.4771572 |
| 6 | C | C10 | -0.0500743 | -0.7951991 | -1.2344074 |

| | | | | | |
|---|---|---|---|---|---|
| 7 | H | H11 | -0.3979950 | -0.3587991 | -2.1691355 |
| 8 | C | C11 | -0.4826418 | -2.0238238 | -0.9158956 |
| 9 | H | H12 | -0.1760631 | -2.5312450 | -0.0058114 |
| 10 | C | C12 | -1.4082921 | -2.7431426 | -1.8178865 |
| 11 | O | O1 | -1.8477926 | -2.3196153 | -2.8698685 |
| 12 | O | O2 | -1.7178395 | -3.9617345 | -1.3139346 |
| 13 | C | C13 | -2.6120458 | -4.7454915 | -2.1180937 |
| 14 | H | H5 | -3.5544664 | -4.2138221 | -2.2744602 |
| 15 | H | H13 | -2.7778343 | -5.6670627 | -1.5592395 |
| 16 | H | H14 | -2.1631492 | -4.9616572 | -3.0915960 |
| 17 | F | F1 | 1.7223381 | 1.2232993 | -2.3539189 |
| 18 | F | F2 | 3.0003533 | 1.1868913 | -0.5460287 |
| 19 | C | C4 | 0.7315355 | 2.1769759 | 1.1301111 |
| 20 | N | N1 | 0.9105237 | 3.3755852 | 3.6778867 |
| 21 | C | C6 | -0.1933435 | 1.8824315 | 2.1393948 |
| 22 | C | C7 | 1.7489586 | 3.0877968 | 1.4319480 |
| 23 | C | C8 | 1.7909243 | 3.6507591 | 2.7088508 |
| 24 | C | C9 | -0.0613028 | 2.5028302 | 3.3802346 |
| 25 | H | H2 | -1.0006241 | 1.1775720 | 1.9604804 |
| 26 | H | H1 | 2.4979879 | 3.3500452 | 0.6929301 |
| 27 | H | H4 | 2.5765237 | 4.3602219 | 2.9639503 |
| 28 | H | H7 | -0.7709234 | 2.2872482 | 4.1774282 |

## 4-pyridine_LHS_TS2 (TS1m)

G = -868.512852
iv = i151
S^2 = 0.5934

Cartesian Coordinates (Angstroms)

| Atom | | | X | Y | Z |
|---|---|---|---|---|---|
| 1 | C | C | 0.3070064 | 2.7970608 | -0.9446703 |
| 2 | C | C2 | -0.8702346 | 2.2484882 | -1.3570235 |
| 3 | C | C3 | -1.4285757 | 1.1354692 | -0.6765308 |
| 4 | C | C4 | 1.0819644 | 2.0509262 | 0.0900012 |
| 5 | C | C5 | 1.1037370 | 0.5761160 | -0.1992953 |
| 6 | H | H | 0.7280987 | 3.7102181 | -1.3504719 |
| 7 | H | H7 | -1.3808928 | 2.6113983 | -2.2462561 |
| 8 | H | H8 | -1.2991936 | 1.0085770 | 0.3895858 |
| 9 | H | H12 | 0.9892222 | 0.3102938 | -1.2422746 |
| 10 | F | F1 | 2.3744958 | 2.5344481 | 0.1628290 |
| 11 | F | F2 | 0.5526452 | 2.2904937 | 1.3649280 |
| 12 | C | C1 | 1.6724039 | -0.4116988 | 0.6680910 |
| 13 | N | N1 | 2.7702392 | -2.4577611 | 2.2998445 |
| 14 | C | C7 | 1.9062917 | -1.7183103 | 0.1748682 |
| 15 | C | C8 | 2.0121818 | -0.1793825 | 2.0237279 |
| 16 | C | C9 | 2.5517642 | -1.2197246 | 2.7707856 |
| 17 | C | C10 | 2.4457148 | -2.6822852 | 1.0166428 |
| 18 | H | H2 | 1.6680436 | -1.9635620 | -0.8566372 |
| 19 | H | H6 | 1.8552203 | 0.7934610 | 2.4703350 |
| 20 | H | H5 | 2.8223384 | -1.0542993 | 3.8121624 |
| 21 | H | H3 | 2.6310239 | -3.6892368 | 0.6468259 |
| 22 | C | C11 | -2.4292836 | 0.2928172 | -1.3442906 |
| 23 | O | O1 | -2.7867842 | 0.4153052 | -2.5041783 |
| 24 | O | O2 | -2.9178859 | -0.6490733 | -0.5024576 |
| 25 | C | C12 | -3.9064488 | -1.5218351 | -1.0694465 |
| 26 | H | H1 | -4.7684266 | -0.9486601 | -1.4213600 |
| 27 | H | H9 | -4.1964768 | -2.1977280 | -0.2648775 |
| 28 | H | H10 | -3.4881892 | -2.0815155 | -1.9108571 |

## 2-pyridine_SM (9n)

G = -868.560284
iv = none

Cartesian Coordinates (Angstroms)

| Atom | | | X | Y | Z |
|---|---|---|---|---|---|
| 1 | C | C1 | 2.1000029 | 0.5055043 | -0.6965333 |
| 2 | C | C2 | 0.9184896 | 1.2927387 | -0.2508197 |
| 3 | C | C3 | 0.9201405 | -0.2843109 | -0.2566169 |
| 4 | H | H3 | 0.3655689 | 1.7584852 | -1.0628600 |
| 5 | H | H6 | 1.0020918 | -0.6850727 | 0.7505895 |
| 6 | C | C10 | 0.0820217 | -1.0040781 | -1.2242092 |
| 7 | H | H11 | 0.0715084 | -0.6434600 | -2.2512806 |
| 8 | C | C11 | -0.6680193 | -2.0749420 | -0.9252445 |

| | | | | | |
|---|---|---|---|---|---|
| 9 | H | H12 | -0.7026792 | -2.4985163 | 0.0742806 |
| 10 | C | C12 | -1.4832540 | -2.7315458 | -1.9687958 |
| 11 | O | O1 | -1.5706412 | -2.3850279 | -3.1315743 |
| 12 | O | O2 | -2.1432411 | -3.7999312 | -1.4572169 |
| 13 | C | C13 | -2.9636894 | -4.5145889 | -2.3923749 |
| 14 | H | H5 | -3.7374900 | -3.8605552 | -2.8038684 |
| 15 | H | H13 | -3.4129366 | -5.3306347 | -1.8252458 |
| 16 | H | H14 | -2.3588262 | -4.9055369 | -3.2152852 |
| 17 | F | F1 | 2.4062756 | 0.5330426 | -2.0166253 |
| 18 | F | F2 | 3.2294763 | 0.4892063 | 0.0356387 |
| 19 | C | C4 | 0.8835237 | 2.0151828 | 1.0526041 |
| 20 | C | C5 | 0.4862175 | 4.0200942 | 2.3217539 |
| 21 | C | C6 | 1.2149747 | 1.9755476 | 3.3252197 |
| 22 | C | C7 | 0.8534596 | 3.3152944 | 3.4674928 |
| 23 | C | C9 | 0.5011632 | 3.3634083 | 1.0942391 |
| 24 | H | H1 | 0.1920557 | 5.0644380 | 2.3820080 |
| 25 | H | H10 | 1.5038450 | 1.3864459 | 4.1940646 |
| 26 | H | H7 | 0.8566146 | 3.7858904 | 4.4457855 |
| 27 | H | H4 | 0.2238540 | 3.8813328 | 0.1805933 |
| 28 | N | N1 | 1.2294931 | 1.3315891 | 2.1542812 |

## 2-pyridine_LHS_TS2 (TS1n)

G = -868.515403
iv = i143
S^2 = 0.6016

Cartesian Coordinates (Angstroms)

| | | Atom | X | Y | Z |
|---|---|---|---|---|---|
| 1 | C | C | -1.3374758 | 2.5356748 | -1.0093448 |
| 2 | C | C2 | -2.2782936 | 1.6088466 | -0.6708224 |
| 3 | C | C3 | -1.9635131 | 0.5397633 | 0.2053482 |
| 4 | C | C4 | 0.0726353 | 2.2614527 | -0.6057535 |
| 5 | C | C5 | 0.4434383 | 0.8277131 | -0.8440428 |
| 6 | H | H | -1.5466778 | 3.4307520 | -1.5847047 |
| 7 | H | H7 | -3.2738083 | 1.6322947 | -1.1088727 |
| 8 | H | H8 | -1.2360491 | 0.6601069 | 0.9961678 |
| 9 | H | H12 | -0.1008181 | 0.3085738 | -1.6207575 |
| 10 | F | F1 | 0.9380644 | 3.0934627 | -1.2933938 |
| 11 | F | F2 | 0.2717235 | 2.6027654 | 0.7419510 |
| 12 | C | C11 | -2.8012772 | -0.6650313 | 0.2258161 |
| 13 | O | O1 | -3.7626787 | -0.8645502 | -0.4984173 |
| 14 | O | O2 | -2.3748274 | -1.5432864 | 1.1655528 |
| 15 | C | C12 | -3.1247583 | -2.7633846 | 1.2453564 |
| 16 | H | H1 | -4.1702767 | -2.5577539 | 1.4924223 |
| 17 | H | H9 | -2.6510838 | -3.3472613 | 2.0353189 |
| 18 | H | H10 | -3.0865709 | -3.3018428 | 0.2938812 |
| 19 | C | C1 | 1.6820642 | 0.2233836 | -0.4512118 |
| 20 | C | C6 | 3.0429579 | -1.6244868 | -0.6922048 |
| 21 | C | C7 | 3.7697283 | 0.1398279 | 0.7639039 |
| 22 | C | C8 | 4.0071794 | -1.1069262 | 0.1855604 |
| 23 | C | C9 | 2.5956014 | 0.8152813 | 0.4550249 |
| 24 | H | H6 | 3.1965203 | -2.5964725 | -1.1593318 |
| 25 | H | H2 | 4.4882175 | 0.5785235 | 1.4508757 |

```
 26  H   H11           2.3721687     1.7800871     0.8912999
 27  H   H15           4.9094911    -1.6702421     0.4031932
 28  N   N1            1.9183188    -0.9972714    -1.0128147
```

## mestial_LHS_SM (9o)

```
G = -931.084973
iv = none

 Cartesian Coordinates (Angstroms)
       Atom             X             Y             Z
    ---------    ------------- ------------- -------------

  1  C   C1            0.1004469     2.2220900     0.6645602
  2  C   C4            0.6179998     2.7845380     3.3602783
  3  C   C2            0.4412212     1.1795836     1.5534176
  4  C   C6           -0.0085121     3.5271508     1.1593174
  5  C   C5            0.2335058     3.8095278     2.5001810
  6  C   C3            0.7356289     1.4666546     2.9061109
  7  H   H6           -0.2769598     4.3290486     0.4757904
  8  C   C7            0.5808699    -0.2319649     1.0555541
  9  C   C8           -0.1705426    -1.3846140     1.6029641
 10  C   C9           -0.5552311    -0.9555791     0.2298027
 11  H   H2            1.5767018    -0.5169806     0.7105993
 12  H   H9           -1.4941709    -0.4095453     0.1875632
 13  F   F1           -1.0798392    -1.2110464     2.5911733
 14  F   F2            0.4777548    -2.5599177     1.7786826
 15  C   C10          -0.1959880    -1.7500721    -0.9518852
 16  H   H8            0.6860607    -2.3846041    -0.8834668
 17  C   C11          -0.8754358    -1.7385575    -2.1084376
 18  H   H10          -1.7676427    -1.1355409    -2.2504491
 19  C   C12          -0.4247943    -2.5601502    -3.2515758
 20  O   O1            0.5476472    -3.2903961    -3.2670891
 21  O   O2           -1.2497147    -2.3917060    -4.3140848
 22  C   C13          -0.9012200    -3.1455892    -5.4840422
 23  H   H7           -1.6573250    -2.8967817    -6.2294611
 24  H   H11          -0.9114799    -4.2176582    -5.2686542
 25  H   H12           0.0948902    -2.8669536    -5.8391450
 26  H   H13           0.8410760     3.0050004     4.4012792
 27  H   H14           0.1448661     4.8280735     2.8688423
 28  C   C14           1.2027439     0.3953000     3.8685409
 29  H   H3            2.0048709    -0.2166351     3.4392861
 30  H   H4            0.3904854    -0.2853752     4.1410036
 31  H   H5            1.5858001     0.8469728     4.7884605
 32  C   C15          -0.1261934     1.9824906    -0.8125576
 33  H   H1            0.6219677     1.3088903    -1.2428746
 34  H   H15          -0.0814835     2.9275316    -1.3626110
 35  H   H16          -1.1080042     1.5368148    -1.0170736
```

## mestial_LHS_TS2 (TS1o)

G = -931.03943
iv = i163
S^2 = 0.4019

Cartesian Coordinates (Angstroms)

| | Atom | | X | Y | Z |
|---|---|---|---|---|---|
| 1 | C | C | -0.9096073 | 2.9999154 | -0.6912791 |
| 2 | C | C2 | -2.0360516 | 2.2377163 | -0.7378677 |
| 3 | C | C3 | -2.1135924 | 1.0215839 | -0.0058768 |
| 4 | C | C4 | 0.2750223 | 2.4204254 | 0.0037197 |
| 5 | C | C5 | 0.4715012 | 0.9822567 | -0.3974421 |
| 6 | H | H | -0.8218177 | 3.9896965 | -1.1255369 |
| 7 | H | H7 | -2.8733542 | 2.5030118 | -1.3800533 |
| 8 | H | H8 | -1.6394225 | 0.9189243 | 0.9604468 |
| 9 | H | H12 | -0.0225180 | 0.7726982 | -1.3310704 |
| 10 | F | F1 | 1.4016049 | 3.1758374 | -0.2977579 |
| 11 | F | F2 | 0.1212585 | 2.5546778 | 1.3887792 |
| 12 | C | C1 | 1.4217506 | -0.0048547 | 0.0215955 |
| 13 | C | C6 | 3.1264852 | -2.1716909 | 0.6879449 |
| 14 | C | C7 | 1.3363574 | -1.2694638 | -0.6749484 |
| 15 | C | C8 | 2.4113891 | 0.1340256 | 1.0593259 |
| 16 | C | C9 | 3.2330401 | -0.9551468 | 1.3562590 |
| 17 | C | C10 | 2.1784890 | -2.3184261 | -0.3231740 |
| 18 | H | H5 | 3.9816581 | -0.8398030 | 2.1353523 |
| 19 | H | H3 | 2.0933610 | -3.2653645 | -0.8495429 |
| 20 | H | H4 | 3.7819432 | -2.9982625 | 0.9491787 |
| 21 | C | C11 | -3.1018826 | 0.0086805 | -0.3848636 |
| 22 | O | O1 | -3.8836344 | 0.0960370 | -1.3186036 |
| 23 | O | O2 | -3.0497525 | -1.0689426 | 0.4432732 |
| 24 | C | C12 | -3.9932816 | -2.1074128 | 0.1491285 |
| 25 | H | H1 | -5.0179906 | -1.7422433 | 0.2652941 |
| 26 | H | H9 | -3.7880318 | -2.9023816 | 0.8670705 |
| 27 | H | H10 | -3.8644584 | -2.4699381 | -0.8747308 |
| 28 | C | C13 | 0.3217102 | -1.5138343 | -1.7672206 |
| 29 | H | H2 | 0.4539469 | -0.8378948 | -2.6220265 |
| 30 | H | H11 | -0.7036854 | -1.3781792 | -1.4022584 |
| 31 | H | H13 | 0.4101087 | -2.5361182 | -2.1452878 |
| 32 | C | C14 | 2.6522116 | 1.3956379 | 1.8523865 |
| 33 | H | H6 | 3.5569560 | 1.2846271 | 2.4567706 |
| 34 | H | H14 | 1.8176205 | 1.6153149 | 2.5241571 |
| 35 | H | H15 | 2.7726663 | 2.2688901 | 1.2088587 |

## CN_LHS_SM (9p)

G = -713.768678
iv = none

Cartesian Coordinates (Angstroms)

| | Atom | | X | Y | Z |
|---|---|---|---|---|---|
| 1 | C | C1 | 2.1105281 | 2.1676364 | 0.0607816 |
| 2 | C | C2 | 0.9282908 | 2.6973176 | 0.8055329 |
| 3 | C | C3 | 1.2084693 | 1.1411205 | 0.6469596 |
| 4 | H | H3 | 0.1174426 | 3.0867177 | 0.1951720 |
| 5 | H | H6 | 1.5781365 | 0.6749817 | 1.5564852 |
| 6 | C | C10 | 0.2994338 | 0.3482683 | -0.1952820 |
| 7 | H | H11 | -0.0074648 | 0.7713752 | -1.1498698 |
| 8 | C | C11 | -0.1680119 | -0.8609948 | 0.1413430 |
| 9 | H | H12 | 0.0980057 | -1.3501675 | 1.0737703 |
| 10 | C | C12 | -1.0836522 | -1.5834987 | -0.7736110 |
| 11 | O | O1 | -1.4813821 | -1.1690509 | -1.8448390 |
| 12 | O | O2 | -1.4294060 | -2.7821559 | -0.2538465 |
| 13 | C | C13 | -2.3207190 | -3.5670072 | -1.0628304 |
| 14 | H | H5 | -3.2547926 | -3.0265207 | -1.2372134 |
| 15 | H | H13 | -2.5037297 | -4.4796588 | -0.4953127 |
| 16 | H | H14 | -1.8592968 | -3.7978324 | -2.0267518 |
| 17 | F | F1 | 2.1283668 | 2.3361226 | -1.2752149 |
| 18 | F | F2 | 3.3385408 | 2.3297532 | 0.5788564 |
| 19 | C | C4 | 1.0853552 | 3.2951414 | 2.1015202 |
| 20 | N | N1 | 1.2158852 | 3.7684526 | 3.1543505 |

## CN_TS2_LHS (TS1p)

G = -713.721964
iv = i155
S^2 = 0.7284

Cartesian Coordinates (Angstroms)

| | Atom | | X | Y | Z |
|---|---|---|---|---|---|
| 1 | C | C | 0.6241679 | 2.4031406 | -0.2738745 |
| 2 | C | C2 | -0.4805786 | 1.6202633 | -0.4326921 |
| 3 | C | C3 | -0.5716376 | 0.3395870 | 0.1665526 |
| 4 | C | C4 | 1.8411921 | 1.7973544 | 0.3558040 |
| 5 | C | C5 | 2.0667465 | 0.3810862 | -0.1131399 |
| 6 | H | H | 0.6878658 | 3.4299286 | -0.6174105 |
| 7 | H | H7 | -1.3048091 | 1.9405880 | -1.0662309 |
| 8 | H | H8 | -0.0794007 | 0.1167471 | 1.1042300 |
| 9 | H | H12 | 1.8288848 | 0.1377578 | -1.1414734 |
| 10 | F | F1 | 2.9471866 | 2.5617131 | 0.0595204 |
| 11 | F | F2 | 1.7442745 | 1.8038173 | 1.7346643 |
| 12 | C | C11 | -1.5489598 | -0.6406201 | -0.3373632 |
| 13 | O | O1 | -2.2846036 | -0.4580160 | -1.2919570 |
| 14 | O | O2 | -1.5247731 | -1.7723946 | 0.3978188 |
| 15 | C | C12 | -2.4409557 | -2.7978593 | -0.0199103 |
| 16 | H | H1 | -3.4727192 | -2.4461302 | 0.0688983 |

| | | | | | |
|---|---|---|---|---|---|
| 17 | H | H9 | -2.2617445 | -3.6389979 | 0.6500185 |
| 18 | H | H10 | -2.2500442 | -3.0821967 | -1.0583823 |
| 19 | C | C1 | 2.9068816 | -0.4760555 | 0.6003823 |
| 20 | N | N1 | 3.5730261 | -1.2197129 | 1.2145449 |

## bis_Me_SM (9q)

G = -700.111308
iv = none

Cartesian Coordinates (Angstroms)

| Atom | | | X | Y | Z |
|---|---|---|---|---|---|
| 1 | C | C1 | 2.0731041 | 1.4329992 | -0.0574773 |
| 2 | C | C2 | 0.9105710 | 2.0532084 | 0.6154089 |
| 3 | C | C3 | 1.0819624 | 0.4757247 | 0.5293786 |
| 4 | H | H6 | 1.3885151 | 0.0266553 | 1.4718288 |
| 5 | C | C10 | 0.2298959 | -0.3588462 | -0.3263205 |
| 6 | H | H11 | -0.0043230 | -0.0017723 | -1.3264940 |
| 7 | C | C11 | -0.2750917 | -1.5474524 | 0.0392841 |
| 8 | H | H12 | -0.0818387 | -1.9863458 | 1.0138698 |
| 9 | C | C12 | -1.1201020 | -2.3171720 | -0.8962823 |
| 10 | O | O1 | -1.4474721 | -1.9684929 | -2.0150925 |
| 11 | O | O2 | -1.5010940 | -3.4947810 | -0.3414644 |
| 12 | C | C13 | -2.3270747 | -4.3210829 | -1.1737455 |
| 13 | H | H5 | -3.2622960 | -3.8090614 | -1.4171567 |
| 14 | H | H13 | -2.5237829 | -5.2216600 | -0.5908583 |
| 15 | H | H14 | -1.8096520 | -4.5700095 | -2.1043086 |
| 16 | F | F1 | 2.1749089 | 1.5080913 | -1.4073872 |
| 17 | F | F2 | 3.3089790 | 1.5151021 | 0.4929244 |
| 18 | C | C4 | 1.1196354 | 2.6538467 | 1.9943286 |
| 19 | H | H2 | 1.3688246 | 3.7187737 | 1.9119609 |
| 20 | H | H1 | 1.9340872 | 2.1627737 | 2.5336219 |
| 21 | H | H4 | 0.2046912 | 2.5670947 | 2.5917509 |
| 22 | C | C5 | -0.1555183 | 2.7305982 | -0.2274199 |
| 23 | H | H3 | 0.0607406 | 3.8027064 | -0.3054149 |
| 24 | H | H7 | -1.1418916 | 2.6190904 | 0.2369124 |
| 25 | H | H8 | -0.2057783 | 2.3300117 | -1.2418472 |

## bis_Me_TS2 (TS1q)

G = -700.060667
iv = i100
S^2 = 0.7900

Cartesian Coordinates (Angstroms)

| Atom | | | X | Y | Z |
|---|---|---|---|---|---|
| 1 | C | C | 0.1237299 | 2.3869725 | -0.1597606 |
| 2 | C | C2 | -1.0083310 | 1.6232422 | -0.1860150 |
| 3 | C | C3 | -1.0802427 | 0.3424862 | 0.4046702 |
| 4 | C | C4 | 1.4031081 | 1.8012226 | 0.3541250 |
| 5 | C | C5 | 1.6811928 | 0.3858892 | -0.0908040 |
| 6 | H | H | 0.1467319 | 3.4180567 | -0.4968980 |

| | | | | | |
|---|---|---|---|---|---|
| 7 | H | H7 | -1.8932190 | 1.9779903 | -0.7108761 |
| 8 | H | H8 | -0.4412332 | 0.0606467 | 1.2306433 |
| 9 | F | F1 | 2.4620626 | 2.6105992 | -0.0533107 |
| 10 | F | F2 | 1.4223842 | 1.8616800 | 1.7422396 |
| 11 | C | C11 | -2.1665313 | -0.5719112 | 0.0454378 |
| 12 | O | O1 | -3.0467581 | -0.3398684 | -0.7678811 |
| 13 | O | O2 | -2.0779041 | -1.7366836 | 0.7382398 |
| 14 | C | C12 | -3.0991150 | -2.6990411 | 0.4427816 |
| 15 | H | H1 | -4.0897223 | -2.2910524 | 0.6629351 |
| 16 | H | H9 | -2.8872352 | -3.5573064 | 1.0817029 |
| 17 | H | H10 | -3.0645781 | -2.9878448 | -0.6119782 |
| 18 | C | C1 | 2.5335867 | -0.4762974 | 0.7855487 |
| 19 | H | H2 | 3.6034920 | -0.2525135 | 0.6302659 |
| 20 | H | H3 | 2.3934697 | -1.5349386 | 0.5393255 |
| 21 | H | H4 | 2.3220170 | -0.3237354 | 1.8460327 |
| 22 | C | C6 | 1.6772984 | 0.1237909 | -1.5629317 |
| 23 | H | H5 | 2.6195015 | 0.4789808 | -2.0145781 |
| 24 | H | H6 | 0.8634720 | 0.6478645 | -2.0733145 |
| 25 | H | H11 | 1.6028234 | -0.9482291 | -1.7756002 |

## spiro_Cy_SM (9r)

G = -816.783222
iv = none

Cartesian Coordinates (Angstroms)

| | | Atom | X | Y | Z |
|---|---|---|---|---|---|
| 1 | C | C7 | 0.8338418 | 0.2700360 | 1.6681000 |
| 2 | C | C8 | 1.3019352 | -1.0963801 | 1.9585323 |
| 3 | C | C9 | 0.1199460 | -1.0099642 | 1.0427021 |
| 4 | H | H9 | -0.8364595 | -1.2368433 | 1.5094591 |
| 5 | F | F1 | 1.1361577 | -1.6278679 | 3.1952081 |
| 6 | F | F2 | 2.4865374 | -1.5127188 | 1.4443376 |
| 7 | C | C10 | 0.2327149 | -1.3458225 | -0.3817849 |
| 8 | H | H8 | 1.2117319 | -1.2760414 | -0.8494771 |
| 9 | C | C11 | -0.7940576 | -1.7382254 | -1.1529121 |
| 10 | H | H10 | -1.8033911 | -1.8477723 | -0.7663437 |
| 11 | C | C12 | -0.5809612 | -2.0369866 | -2.5837731 |
| 12 | O | O1 | 0.4763905 | -1.9589756 | -3.1808004 |
| 13 | C | C1 | 0.0253239 | 1.0203924 | 2.7196751 |
| 14 | C | C3 | 1.6510379 | 1.1890916 | 0.7694728 |
| 15 | C | C5 | -0.0685140 | 3.0189961 | 1.1530655 |
| 16 | C | C4 | 0.7469214 | 2.2428305 | 0.1069988 |
| 17 | C | C6 | -0.8763674 | 2.0762243 | 2.0588939 |
| 18 | H | H6 | 2.4012181 | 1.6924191 | 1.3970295 |
| 19 | H | H1 | 0.6167925 | 3.6165654 | 1.7724597 |
| 20 | H | H3 | 0.0654169 | 1.7409366 | -0.5935039 |
| 21 | H | H4 | -1.6452781 | 1.5663157 | 1.4610916 |
| 22 | H | H13 | -0.5610714 | 0.3219513 | 3.3257121 |
| 23 | H | H14 | 2.2108182 | 0.6259676 | 0.0173195 |
| 24 | H | H15 | -0.7402863 | 3.7301518 | 0.6567296 |
| 25 | H | H16 | 1.3571245 | 2.9335692 | -0.4875397 |
| 26 | H | H17 | -1.4061865 | 2.6472244 | 2.8313489 |
| 27 | H | H19 | 0.7275683 | 1.5149340 | 3.4072191 |

| | | | | | |
|---|---|---|---|---|---|
| 28 | O | O2 | -1.7431661 | -2.4142474 | -3.1732278 |
| 29 | C | C2 | -1.6457089 | -2.7183003 | -4.5716870 |
| 30 | H | H2 | -0.9410616 | -3.5372764 | -4.7398430 |
| 31 | H | H5 | -1.3079479 | -1.8430298 | -5.1339370 |
| 32 | H | H7 | -2.6510199 | -3.0071538 | -4.8805256 |

## spiro_Cy_TS2 (TS1r)

G = -816.731056
iv = i97
S^2 = 0.7478

Cartesian Coordinates (Angstroms)

| | Atom | | X | Y | Z |
|---|---|---|---|---|---|
| 1 | C | C | -0.9106382 | 0.1385209 | -2.4198357 |
| 2 | C | C2 | -2.0066762 | 0.0588373 | -1.6096145 |
| 3 | C | C3 | -2.0468213 | 0.6448966 | -0.3245854 |
| 4 | C | C4 | 0.3617085 | 0.7027390 | -1.8766115 |
| 5 | C | C5 | 0.7101923 | 0.2673726 | -0.4763650 |
| 6 | H | H | -0.9127853 | -0.1936752 | -3.4526047 |
| 7 | H | H7 | -2.8809086 | -0.5071742 | -1.9252811 |
| 8 | H | H8 | -1.4417739 | 1.5058868 | -0.0739070 |
| 9 | F | F1 | 1.4122950 | 0.3522255 | -2.7348973 |
| 10 | F | F2 | 0.3253288 | 2.0931194 | -1.9280441 |
| 11 | C | C11 | -3.0625295 | 0.2215394 | 0.6410615 |
| 12 | O | O1 | -3.9253566 | -0.6174316 | 0.4373100 |
| 13 | O | O2 | -2.9288840 | 0.8840187 | 1.8196481 |
| 14 | C | C12 | -3.8809944 | 0.5254657 | 2.8300970 |
| 15 | H | H1 | -4.8911736 | 0.8195024 | 2.5290081 |
| 16 | H | H9 | -3.5784079 | 1.0691925 | 3.7260809 |
| 17 | H | H10 | -3.8678857 | -0.5531413 | 3.0093056 |
| 18 | C | C1 | 0.7278114 | -1.1969004 | -0.1483625 |
| 19 | C | C6 | 3.1970845 | -0.9642782 | 0.4242527 |
| 20 | C | C7 | 1.6949869 | 1.0935451 | 0.2973150 |
| 21 | C | C8 | 3.1336164 | 0.5342591 | 0.0967162 |
| 22 | C | C10 | 2.1569301 | -1.7667288 | -0.3710193 |
| 23 | H | H6 | 3.0191287 | -1.1074851 | 1.5005324 |
| 24 | H | H2 | 1.4469283 | 1.0280261 | 1.3671402 |
| 25 | H | H3 | 3.4328635 | 0.7002145 | -0.9452832 |
| 26 | H | H4 | 2.3923188 | -1.7226277 | -1.4412872 |
| 27 | H | H5 | -0.0026178 | -1.7533951 | -0.7426260 |
| 28 | H | H12 | 4.2045241 | -1.3485397 | 0.2199300 |
| 29 | H | H13 | 1.6477085 | 2.1463858 | 0.0092794 |
| 30 | H | H14 | 3.8312146 | 1.0989149 | 0.7277639 |
| 31 | H | H16 | 2.1753025 | -2.8235721 | -0.0765909 |
| 32 | H | H17 | 0.4675095 | -1.3297124 | 0.9114743 |

### Me_LHS_SM (9s)

G = -660.822287
iv = none

Cartesian Coordinates (Angstroms)

| | Atom | | X | Y | Z |
|---|---|---|---|---|---|
| 1 | C | C1 | 2.0231665 | 1.8219797 | -0.1009084 |
| 2 | C | C2 | 0.8591224 | 2.3957932 | 0.5973220 |
| 3 | C | C3 | 1.0653756 | 0.8373953 | 0.4948820 |
| 4 | H | H3 | 0.0828452 | 2.7793574 | -0.0623670 |
| 5 | H | H6 | 1.4065548 | 0.3683184 | 1.4155768 |
| 6 | C | C10 | 0.1794920 | 0.0384012 | -0.3610723 |
| 7 | H | H11 | -0.1057450 | 0.4597875 | -1.3234845 |
| 8 | C | C11 | -0.3015600 | -1.1721524 | -0.0405860 |
| 9 | H | H12 | -0.0568987 | -1.6648328 | 0.8959144 |
| 10 | C | C12 | -1.1930632 | -1.8907679 | -0.9742955 |
| 11 | O | O1 | -1.5745636 | -1.4815698 | -2.0545686 |
| 12 | O | O2 | -1.5477945 | -3.0970966 | -0.4663047 |
| 13 | C | C13 | -2.4130947 | -3.8792491 | -1.3015377 |
| 14 | H | H5 | -3.3460342 | -3.3437464 | -1.4979605 |
| 15 | H | H13 | -2.6068929 | -4.7973238 | -0.7456777 |
| 16 | H | H14 | -1.9280348 | -4.1029442 | -2.2558562 |
| 17 | F | F1 | 2.1083142 | 1.9187173 | -1.4481460 |
| 18 | F | F2 | 3.2560764 | 1.9357782 | 0.4474883 |
| 19 | C | C4 | 0.9961342 | 3.0828046 | 1.9380245 |
| 20 | H | H2 | 1.1842622 | 4.1541589 | 1.8037964 |
| 21 | H | H1 | 1.8310465 | 2.6675779 | 2.5100867 |
| 22 | H | H4 | 0.0812917 | 2.9696133 | 2.5296743 |

### Me_LHS_TS2 (TS1s)

G = -660.768319
iv = i131
S^2 = 0.6779

Cartesian Coordinates (Angstroms)

| | Atom | | X | Y | Z |
|---|---|---|---|---|---|
| 1 | C | C | 0.3085323 | 2.3948690 | -0.3713510 |
| 2 | C | C2 | -0.8130753 | 1.6163945 | -0.4309390 |
| 3 | C | C3 | -0.8733321 | 0.3534257 | 0.2006025 |
| 4 | C | C4 | 1.5557584 | 1.7871013 | 0.1739419 |
| 5 | C | C5 | 1.7883266 | 0.4037325 | -0.3426420 |
| 6 | H | H | 0.3500821 | 3.4137669 | -0.7408256 |
| 7 | H | H7 | -1.6791382 | 1.9321921 | -1.0087759 |
| 8 | H | H8 | -0.2831662 | 0.1347899 | 1.0804898 |
| 9 | H | H12 | 1.3603866 | 0.1697550 | -1.3099371 |
| 10 | F | F1 | 2.6477364 | 2.5869476 | -0.1376405 |
| 11 | F | F2 | 1.5407328 | 1.7597005 | 1.5709135 |
| 12 | C | C11 | -1.9181127 | -0.6061449 | -0.1751014 |
| 13 | O | O1 | -2.7455039 | -0.4375735 | -1.0556302 |
| 14 | O | O2 | -1.8572819 | -1.7243077 | 0.5904331 |

| | | | | | |
|---|---|---|---|---|---|
| 15 | C | C12 | -2.8504334 | -2.7170042 | 0.2970293 |
| 16 | H | H1 | -3.8548958 | -2.3160435 | 0.4598089 |
| 17 | H | H9 | -2.6521582 | -3.5419732 | 0.9820799 |
| 18 | H | H10 | -2.7687977 | -3.0501926 | -0.7414680 |
| 19 | C | C1 | 2.9503630 | -0.3851851 | 0.1507666 |
| 20 | H | H2 | 3.8623132 | -0.1334861 | -0.4167327 |
| 21 | H | H3 | 2.7802482 | -1.4597707 | 0.0183974 |
| 22 | H | H4 | 3.1514158 | -0.1809937 | 1.2065809 |

## Cyclohexane_LHS_SM (9t)

G = -856.068176
iv = none

Cartesian Coordinates (Angstroms)

| Atom | | | X | Y | Z |
|---|---|---|---|---|---|
| 1 | C | C1 | 1.8132546 | -0.1254555 | -1.2431369 |
| 2 | C | C2 | 0.6689606 | 0.5838044 | -0.6484235 |
| 3 | C | C3 | 0.7976203 | -0.9827691 | -0.5514934 |
| 4 | H | H6 | 1.0980521 | -1.3510941 | 0.4276266 |
| 5 | C | C10 | -0.0974759 | -1.8420595 | -1.3358473 |
| 6 | H | H11 | -0.3637252 | -1.5051304 | -2.3363689 |
| 7 | C | C11 | -0.6038909 | -3.0121600 | -0.9186998 |
| 8 | H | H12 | -0.3800074 | -3.4297584 | 0.0586900 |
| 9 | C | C12 | -1.4888097 | -3.7946546 | -1.8059083 |
| 10 | O | O1 | -1.8576938 | -3.4646708 | -2.9173734 |
| 11 | O | O2 | -1.8499558 | -4.9621632 | -1.2174127 |
| 12 | C | C13 | -2.7011198 | -5.8031835 | -2.0087260 |
| 13 | H | H5 | -3.6432313 | -5.2952692 | -2.2332015 |
| 14 | H | H13 | -2.8796215 | -6.6930651 | -1.4039693 |
| 15 | H | H14 | -2.2120140 | -6.0690561 | -2.9498777 |
| 16 | F | F1 | 1.9300157 | -0.2036574 | -2.5895176 |
| 17 | F | F2 | 3.0420992 | 0.0008356 | -0.6833144 |
| 18 | H | H27 | -0.0787439 | 0.9222028 | -1.3654762 |
| 19 | C | C4 | 0.8320289 | 1.4467615 | 0.5901217 |
| 20 | C | C5 | 1.5341502 | 3.7458363 | 1.4385367 |
| 21 | C | C6 | -0.3191983 | 2.4784660 | 2.6147853 |
| 22 | C | C7 | 0.2214645 | 3.8631329 | 2.2267911 |
| 23 | C | C8 | -0.4816357 | 1.5709344 | 1.3856840 |
| 24 | C | C9 | 1.3760635 | 2.8396635 | 0.2090836 |
| 25 | H | H1 | 2.3145050 | 3.3312512 | 2.0936405 |
| 26 | H | H10 | 0.3738144 | 2.0058396 | 3.3264914 |
| 27 | H | H7 | -0.5259304 | 4.3824974 | 1.6086663 |
| 28 | H | H2 | -1.2592787 | 1.9886008 | 0.7282212 |
| 29 | H | H3 | 0.6841610 | 3.3106055 | -0.5056947 |
| 30 | H | H4 | 1.5795893 | 0.9671361 | 1.2389613 |
| 31 | H | H8 | 1.8840046 | 4.7383857 | 1.1281029 |
| 32 | H | H9 | -1.2801993 | 2.5756065 | 3.1353638 |
| 33 | H | H15 | 0.3682606 | 4.4789489 | 3.1231632 |
| 34 | H | H16 | -0.8321850 | 0.5758432 | 1.6887860 |
| 35 | H | H17 | 2.3366723 | 2.7277945 | -0.3082738 |

### Cyclohexane_LHS_TS2 (TS1t)

G = -856.014022
iv = i85
S^2 = 0.6959

Cartesian Coordinates (Angstroms)

| | Atom | | X | Y | Z |
|---|---|---|---|---|---|
| 1 | C | C | -1.1459943 | 3.1420549 | -0.3807845 |
| 2 | C | C2 | -2.3123754 | 2.4309537 | -0.4431040 |
| 3 | C | C3 | -2.4581901 | 1.1834592 | 0.2011743 |
| 4 | C | C4 | 0.0639459 | 2.4728074 | 0.1785269 |
| 5 | C | C5 | 0.2378422 | 1.0780749 | -0.3265326 |
| 6 | H | H | -1.0443228 | 4.1549539 | -0.7551947 |
| 7 | H | H7 | -3.1534524 | 2.7964908 | -1.0285193 |
| 8 | H | H8 | -1.8743492 | 0.9306885 | 1.0759075 |
| 9 | H | H12 | -0.1990978 | 0.8539743 | -1.2939466 |
| 10 | F | F1 | 1.1937126 | 3.2292701 | -0.1245203 |
| 11 | F | F2 | 0.0351417 | 2.4568309 | 1.5756810 |
| 12 | C | C11 | -3.5671769 | 0.2930240 | -0.1557100 |
| 13 | O | O1 | -4.3893255 | 0.5049005 | -1.0319125 |
| 14 | O | O2 | -3.5758169 | -0.8171822 | 0.6256282 |
| 15 | C | C12 | -4.6388617 | -1.7415579 | 0.3566904 |
| 16 | H | H1 | -5.6111052 | -1.2696221 | 0.5252804 |
| 17 | H | H9 | -4.4906418 | -2.5695469 | 1.0508394 |
| 18 | H | H10 | -4.5931794 | -2.0931125 | -0.6779004 |
| 19 | C | C1 | 1.3281238 | 0.2014565 | 0.1880883 |
| 20 | C | C6 | 2.0361646 | -2.2017771 | 0.6649187 |
| 21 | C | C7 | 3.7606359 | -0.5154607 | -0.1164017 |
| 22 | C | C8 | 3.3347840 | -1.9902941 | -0.1272322 |
| 23 | C | C9 | 2.6408840 | 0.4018975 | -0.6276467 |
| 24 | C | C10 | 0.9096345 | -1.2879287 | 0.1613144 |
| 25 | H | H6 | 2.2214743 | -1.9930558 | 1.7287106 |
| 26 | H | H2 | 4.0288647 | -0.2203249 | 0.9085850 |
| 27 | H | H3 | 3.1802209 | -2.3140483 | -1.1672855 |
| 28 | H | H11 | 2.4375610 | 0.1804705 | -1.6856019 |
| 29 | H | H4 | 0.6471647 | -1.5634168 | -0.8713829 |
| 30 | H | H5 | 1.5382216 | 0.4850275 | 1.2284702 |
| 31 | H | H13 | 1.7187659 | -3.2504677 | 0.6040024 |
| 32 | H | H14 | 4.6613020 | -0.3721416 | -0.7272798 |
| 33 | H | H15 | 4.1354506 | -2.6202288 | 0.2804987 |
| 34 | H | H16 | 2.9425636 | 1.4522243 | -0.5736873 |
| 35 | H | H17 | 0.0014311 | -1.4283938 | 0.7603263 |

## Nosub_SM (9u)

G = -621.530722
iv = none

Cartesian Coordinates (Angstroms)

| | Atom | | X | Y | Z |
|---|---|---|---|---|---|
| 1 | C | C1 | 2.2693226 | 2.2659885 | 0.0864734 |
| 2 | C | C2 | 1.1197603 | 2.9740186 | 0.6698508 |
| 3 | C | C3 | 1.2450634 | 1.4144417 | 0.7701306 |
| 4 | H | H6 | 1.5344577 | 1.0450348 | 1.7510014 |
| 5 | C | C10 | 0.3425391 | 0.5632196 | -0.0173407 |
| 6 | H | H11 | 0.0778347 | 0.9032775 | -1.0171279 |
| 7 | C | C11 | -0.1726064 | -0.6001362 | 0.4056053 |
| 8 | H | H12 | 0.0465146 | -1.0118713 | 1.3865594 |
| 9 | C | C12 | -1.0708885 | -1.3795287 | -0.4724605 |
| 10 | O | O1 | -1.4275210 | -1.0602966 | -1.5906800 |
| 11 | O | O2 | -1.4615842 | -2.5255858 | 0.1371570 |
| 12 | C | C13 | -2.3404688 | -3.3567339 | -0.6346471 |
| 13 | H | H5 | -3.2668138 | -2.8246330 | -0.8690111 |
| 14 | H | H13 | -2.5467023 | -4.2262388 | -0.0095105 |
| 15 | H | H14 | -1.8609645 | -3.6579635 | -1.5697553 |
| 16 | F | F1 | 2.3857290 | 2.1922397 | -1.2587838 |
| 17 | F | F2 | 3.4912239 | 2.3878929 | 0.6511892 |
| 18 | H | H19 | 1.2698913 | 3.5329598 | 1.5878548 |
| 19 | H | H27 | 0.3652130 | 3.3639145 | -0.0065050 |

## Nosub_TS2 (TS1u)

G = -621.573778, iv = i192, S^2 = 0.7541

Cartesian Coordinates (Angstroms)

| | Atom | | X | Y | Z |
|---|---|---|---|---|---|
| 1 | C | C | 0.7946670 | 2.3077343 | -0.3275513 |
| 2 | C | C2 | -0.3207882 | 1.5191922 | -0.3512372 |
| 3 | C | C3 | -0.3471353 | 0.2472733 | 0.2644960 |
| 4 | C | C4 | 2.0777544 | 1.7153433 | 0.1641641 |
| 5 | C | C5 | 2.2929621 | 0.3245173 | -0.3214240 |
| 6 | H | H | 0.8063598 | 3.3332337 | -0.6810900 |
| 7 | H | H7 | -1.2132158 | 1.8362782 | -0.8866403 |
| 8 | H | H8 | 0.2852294 | 0.0174445 | 1.1119224 |
| 9 | H | H12 | 1.9390721 | 0.0455178 | -1.3051098 |
| 10 | F | F1 | 3.1381612 | 2.5196024 | -0.2259812 |
| 11 | F | F2 | 2.1241288 | 1.7200433 | 1.5552521 |
| 12 | C | C11 | -1.3975823 | -0.7187094 | -0.0857327 |
| 13 | O | O1 | -2.2725753 | -0.5339239 | -0.9149029 |
| 14 | O | O2 | -1.2755861 | -1.8575817 | 0.6374555 |
| 15 | C | C12 | -2.2574039 | -2.8663948 | 0.3575224 |
| 16 | H | H1 | -3.2646088 | -2.4873954 | 0.5511549 |
| 17 | H | H9 | -2.0236528 | -3.6957507 | 1.0256294 |
| 18 | H | H10 | -2.1949143 | -3.1843250 | -0.6871621 |
| 19 | H | H14 | 3.1091280 | -0.2420994 | 0.1192347 |

**Traige: Alkene Substition**

## CN_E_RHS_SM (10a)

```
Spartan'10: G = -716.912803, iv = none
Gaussian'09: B3LYP/6-31G*, G = -716.917313, iv = none
Gaussian'09: M05-2X/6-31G*, G = -716.875782, iv = none

Cartesian Coordinates (Angstroms)
       Atom             X             Y             Z
    ---------     ------------- ------------- -------------

   1  C   C1          1.2099133    -0.0977419    -1.6065770
   2  C   C2          0.0590977     0.4469622    -0.8600824
   3  C   C3          0.2832058    -1.1118198    -1.0155466
   4  H   H3         -0.7357640     0.8262827    -1.5022389
   5  H   H6          0.6520854    -1.5923392    -0.1124772
   6  C   C4          0.1766691     1.0873485     0.4884279
   7  C   C5          0.3166115     2.3312581     2.9990340
   8  C   C6         -0.7584871     0.7817166     1.4871255
   9  C   C7          1.1837058     2.0222044     0.7609151
  10  C   C8          1.2518592     2.6400825     2.0107107
  11  C   C9         -0.6903124     1.3999595     2.7345575
  12  H   H4         -1.5401211     0.0543296     1.2818457
  13  H   H7          1.9166062     2.2596338    -0.0034322
  14  H   H8          2.0386332     3.3621794     2.2112263
  15  H   H9         -1.4215208     1.1546333     3.5000254
  16  H   H10         0.3715235     2.8128553     3.9715426
  17  C   C10        -0.6131026    -1.9007668    -1.8689807
  18  H   H11        -0.9056200    -1.4587226    -2.8188340
  19  C   C11        -1.0781109    -3.1203258    -1.5398247
  20  H   H12        -0.7980410    -3.5941554    -0.6016563
  21  F   F1          1.2349442     0.0471189    -2.9525554
  22  F   F2          2.4623054     0.0002929    -1.1107415
  23  C   C12        -1.9519651    -3.8639691    -2.3870171
  24  N   N1         -2.6641154    -4.4870174    -3.0654468
```

## CN_E_RHS_TS2 (TS2a)

```
Spartan'10: G = -716.875779, iv = i139, S^2 = 0.5463
Gaussian'09: B3LYP/6-31G*, G = -716.878006, iv = i142, S^2 = 0.71
Gaussian'09: M05-2X/6-31G*, G = -716.822858, iv = i163, S^2 = 0.87

 Cartesian Coordinates (Angstroms)
       Atom             X             Y             Z
    ---------     ------------- ------------- -------------

   1  C   C          -2.0151459     2.2303766    -0.0973687
   2  C   C2         -2.9431550     1.3505820     0.3674799
   3  C   C3         -2.5398690     0.1873772     1.0918623
   4  C   C4         -0.5821229     1.8287807    -0.0075954
   5  C   C5         -0.3850858     0.4037320    -0.4380687
   6  H   H          -2.2614819     3.1842132    -0.5498818
   7  H   H7         -4.0012889     1.4983995     0.1605929
   8  H   H8         -1.6636352     0.2188718     1.7271340
   9  H   H12        -1.1535598     0.0087404    -1.0894109
```

| | | | | | |
|---|---|---|---|---|---|
| 10 | F | F1 | 0.2022057 | 2.6699625 | -0.7739606 |
| 11 | F | F2 | -0.1019806 | 2.0131870 | 1.2992351 |
| 12 | C | C1 | 0.8586417 | -0.2923927 | -0.3976390 |
| 13 | C | C6 | 3.2581461 | -1.7749134 | -0.3738577 |
| 14 | C | C7 | 0.9563986 | -1.5448774 | -1.0627802 |
| 15 | C | C8 | 2.0071669 | 0.1905469 | 0.2877517 |
| 16 | C | C9 | 3.1855331 | -0.5420531 | 0.2868369 |
| 17 | C | C10 | 2.1357579 | -2.2738553 | -1.0474285 |
| 18 | H | H2 | 0.0850569 | -1.9299378 | -1.5868042 |
| 19 | H | H6 | 1.9565916 | 1.1379754 | 0.8093028 |
| 20 | H | H5 | 4.0568926 | -0.1560074 | 0.8089601 |
| 21 | H | H3 | 2.1878759 | -3.2298883 | -1.5607497 |
| 22 | H | H4 | 4.1834916 | -2.3440671 | -0.3629747 |
| 23 | C | C11 | -3.3726726 | -0.9440476 | 1.2101815 |
| 24 | N | N1 | -4.0537612 | -1.8907052 | 1.2991830 |

## Weinreb_Amide_RHS_SM (10b)

Spartan'10: G = -947.067954, iv = none
Gaussian'09: B3LYP/6-31G*, G = -947.076299, iv = none
Gaussian'09: M05-2X/6-31G*, G = -947.020971, iv = none

Cartesian Coordinates (Angstroms)

| | Atom | | X | Y | Z |
|---|---|---|---|---|---|
| 1 | C | C1 | -1.4670215 | -1.8630042 | -0.2979475 |
| 2 | C | C2 | -1.7954595 | -0.7553857 | 0.6210089 |
| 3 | C | C3 | -0.5077807 | -0.7195424 | -0.3017992 |
| 4 | H | H3 | -1.5563263 | -0.9723070 | 1.6617199 |
| 5 | H | H6 | -0.6081401 | -0.0550275 | -1.1571230 |
| 6 | C | C4 | -2.9022014 | 0.2271570 | 0.4004430 |
| 7 | C | C5 | -4.9645210 | 2.1066034 | 0.0780893 |
| 8 | C | C6 | -2.7137389 | 1.5659107 | 0.7746769 |
| 9 | C | C7 | -4.1376207 | -0.1588032 | -0.1355554 |
| 10 | C | C8 | -5.1610036 | 0.7773159 | -0.2962337 |
| 11 | C | C9 | -3.7364277 | 2.4990068 | 0.6162029 |
| 12 | H | H4 | -1.7569553 | 1.8733261 | 1.1900092 |
| 13 | H | H7 | -4.2953475 | -1.1902837 | -0.4317271 |
| 14 | H | H8 | -6.1137281 | 0.4635579 | -0.7144940 |
| 15 | H | H9 | -3.5747818 | 3.5324214 | 0.9112700 |
| 16 | H | H10 | -5.7625886 | 2.8333963 | -0.0472600 |
| 17 | C | C10 | 0.8266066 | -0.8336070 | 0.2998313 |
| 18 | H | H11 | 0.9617074 | -1.5572226 | 1.1015221 |
| 19 | C | C11 | 1.8856003 | -0.0975559 | -0.0705567 |
| 20 | H | H12 | 1.8128700 | 0.6439152 | -0.8574601 |
| 21 | C | C12 | 3.1819019 | -0.2851643 | 0.6277332 |
| 22 | O | O1 | 3.3361624 | -1.0633959 | 1.5655795 |
| 23 | N | N1 | 4.2673549 | 0.4261932 | 0.1278718 |
| 24 | C | C13 | 5.4661468 | 0.6116434 | 0.9286587 |
| 25 | H | H5 | 5.5681329 | -0.2635093 | 1.5695692 |
| 26 | H | H13 | 5.3895370 | 1.5154256 | 1.5463922 |
| 27 | H | H14 | 6.3395043 | 0.6949450 | 0.2750625 |
| 28 | F | F1 | -1.0729595 | -3.0457522 | 0.2301305 |
| 29 | F | F2 | -2.2134450 | -2.0857404 | -1.4061244 |
| 30 | O | O2 | 3.9800488 | 1.5460992 | -0.6824481 |

| | | | | | |
|---|---|---|---|---|---|
| 31 | C | C14 | 4.4328597 | 1.3118606 | -2.0186236 |
| 32 | H | H2 | 4.2195193 | 2.2375591 | -2.5596953 |
| 33 | H | H1 | 3.8943077 | 0.4753809 | -2.4795787 |
| 34 | H | H15 | 5.5107114 | 1.1111478 | -2.0471366 |

## Weinreb_amide_RHS_TS2 (TS2b)

Spartan'10: G = -947.029070, iv = i127, S^2 = 0.5635
Gaussian'09: B3LYP/6-31G*, G = -947.034526, iv = i142, S^2 = 0.55
Gaussian'09: M05-2X/6-31G*, G = -716.829881, iv = i163, S^2 = 0.87

Cartesian Coordinates (Angstroms)

| Atom | | | X | Y | Z |
|---|---|---|---|---|---|
| 1 | C | C | -0.3708103 | 3.2467077 | -0.7831259 |
| 2 | C | C2 | -1.5345291 | 2.5375201 | -0.8136579 |
| 3 | C | C3 | -1.6370972 | 1.2810981 | -0.1615410 |
| 4 | C | C4 | 0.8362767 | 2.5577318 | -0.2460828 |
| 5 | C | C5 | 0.9440416 | 1.1547946 | -0.7683250 |
| 6 | H | H | -0.2640747 | 4.2559235 | -1.1654640 |
| 7 | H | H7 | -2.3928187 | 2.8872876 | -1.3828514 |
| 8 | H | H8 | -1.0886985 | 1.0873996 | 0.7477209 |
| 9 | H | H12 | 0.4333421 | 0.9712623 | -1.7046199 |
| 10 | F | F1 | 1.9794635 | 3.2721405 | -0.5629116 |
| 11 | F | F2 | 0.8234439 | 2.5560476 | 1.1595635 |
| 12 | C | C1 | 1.9584378 | 0.2243082 | -0.3835976 |
| 13 | C | C6 | 3.9199424 | -1.6910241 | 0.2942364 |
| 14 | C | C7 | 2.1243885 | -0.9626358 | -1.1474145 |
| 15 | C | C8 | 2.8072229 | 0.4142519 | 0.7398366 |
| 16 | C | C9 | 3.7737304 | -0.5287123 | 1.0609754 |
| 17 | C | C10 | 3.0871008 | -1.9039211 | -0.8110489 |
| 18 | H | H2 | 1.4847797 | -1.1228519 | -2.0121957 |
| 19 | H | H6 | 2.6945236 | 1.3061860 | 1.3428490 |
| 20 | H | H5 | 4.4196154 | -0.3619704 | 1.9188608 |
| 21 | H | H3 | 3.1977030 | -2.8027072 | -1.4116446 |
| 22 | H | H4 | 4.6764064 | -2.4256027 | 0.5560373 |
| 23 | C | C11 | -2.7109407 | 0.3574663 | -0.5597123 |
| 24 | O | O1 | -3.4753177 | 0.5889965 | -1.4986722 |
| 25 | N | N1 | -2.7996676 | -0.8457652 | 0.1359310 |
| 26 | O | O2 | -2.1985384 | -0.8783023 | 1.4133387 |
| 27 | C | C12 | -4.0180133 | -1.6373118 | 0.0779455 |
| 28 | H | H9 | -3.7798796 | -2.6879591 | 0.2658295 |
| 29 | H | H10 | -4.4370453 | -1.5272446 | -0.9217100 |
| 30 | H | H11 | -4.7482652 | -1.2934599 | 0.8216190 |
| 31 | C | C13 | -1.1294124 | -1.8306027 | 1.4283861 |
| 32 | H | H1 | -0.7534186 | -1.8191473 | 2.4549569 |
| 33 | H | H13 | -0.3318626 | -1.5429230 | 0.7352616 |
| 34 | H | H14 | -1.4900290 | -2.8369809 | 1.1812269 |

## AllylAlcohol_RHS_E_SM (10c)

```
G = -739.155508
iv = none

  Cartesian Coordinates (Angstroms)
       Atom             X             Y             Z
    ---------    ------------- ------------- -------------

  1 C   C1          0.2555785     2.1890182     0.4890142
  2 C   C4         -0.0090838     2.6009899     3.2310042
  3 C   C2          0.3676012     1.0972270     1.3622191
  4 C   C6          0.0168255     3.4717008     0.9799304
  5 C   C5         -0.1172528     3.6810733     2.3545178
  6 C   C3          0.2336460     1.3170931     2.7394558
  7 H   H6         -0.0667795     4.3074216     0.2899720
  8 C   C7          0.6624323    -0.2635615     0.8118213
  9 C   C8          0.0034628    -1.4998679     1.2885984
 10 C   C9         -0.4096150    -1.0224052    -0.0598878
 11 H   H2          1.6825928    -0.4228790     0.4619987
 12 H   H9         -1.3779573    -0.5256805    -0.0738933
 13 F   F1         -0.8920404    -1.4510192     2.3058011
 14 F   F2          0.7442514    -2.6307156     1.3926437
 15 C   C10        -0.0144213    -1.7308339    -1.2938316
 16 C   C11        -0.7356426    -1.7174309    -2.4181920
 17 H   H10        -1.6777015    -1.1702959    -2.4622000
 18 H   H13        -0.1140383     2.7552384     4.3017809
 19 H   H14        -0.3054297     4.6800810     2.7388050
 20 H   H17         0.3109915     0.4797849     3.4253868
 21 H   H3          0.3540560     2.0251906    -0.5814957
 22 H   H12         0.9182520    -2.2882847    -1.2718986
 23 C   C12        -0.3541539    -2.4248015    -3.6850703
 24 H   H1         -0.3431852    -1.6949674    -4.5151730
 25 H   H4         -1.1372756    -3.1636898    -3.9344604
 26 O   O1          0.9111033    -3.0494862    -3.5340332
 27 H   H5          1.0937834    -3.5488993    -4.3428135
```

## allylalcohol_RHS_E_TS2 (TS2c)

```
G = -739.115497
iv = i149
S^2 = 0.5638

Cartesian Coordinates (Angstroms)
       Atom             X             Y             Z
    ---------    ------------- ------------- -------------

  1 C   C          -0.9916180     2.5756204    -0.6751842
  2 C   C2         -2.1559361     1.8593542    -0.5635713
  3 C   C3         -2.2167936     0.7019090     0.2369574
  4 C   C4          0.2411986     1.9532219    -0.1246117
  5 C   C5          0.3251911     0.5046037    -0.4913438
  6 H   H          -0.9166136     3.5396422    -1.1658984
  7 H   H8         -1.5833461     0.6228962     1.1114011
  8 H   H12        -0.2252742     0.2257674    -1.3802903
  9 F   F1          1.3654598     2.6372691    -0.5628246
```

| | | | | | |
|---|---|---|---|---|---|
| 10 | F | F2 | 0.2941675 | 2.1103313 | 1.2790261 |
| 11 | C | C1 | 1.3651672 | -0.3820207 | -0.0641299 |
| 12 | C | C6 | 3.3555405 | -2.2306219 | 0.7196571 |
| 13 | C | C7 | 1.5116549 | -1.6321336 | -0.7209743 |
| 14 | C | C8 | 2.2515829 | -0.0921458 | 1.0071926 |
| 15 | C | C9 | 3.2304789 | -1.0033181 | 1.3810741 |
| 16 | C | C10 | 2.4878426 | -2.5398509 | -0.3345601 |
| 17 | H | H2 | 0.8427593 | -1.8714578 | -1.5445940 |
| 18 | H | H6 | 2.1571432 | 0.8506500 | 1.5309215 |
| 19 | H | H5 | 3.9032527 | -0.7590580 | 2.1991517 |
| 20 | H | H3 | 2.5795329 | -3.4894655 | -0.8552001 |
| 21 | H | H4 | 4.1215079 | -2.9392671 | 1.0226611 |
| 22 | H | H14 | -3.0327146 | 2.1343550 | -1.1457768 |
| 23 | C | C11 | -3.3073964 | -0.3132689 | 0.1297800 |
| 24 | H | H1 | -4.0085898 | -0.1780739 | 0.9764311 |
| 25 | H | H7 | -2.8726158 | -1.3209940 | 0.2537061 |
| 26 | O | O1 | -3.9795164 | -0.1834506 | -1.1147506 |
| 27 | H | H9 | -4.7420651 | -0.7804935 | -1.1042495 |

## Me_RHS_E_SM (10d)

G = -663.957928
iv = none

Cartesian Coordinates (Angstroms)

| | Atom | | X | Y | Z |
|---|---|---|---|---|---|
| 1 | C | C1 | 0.4009824 | 2.0386001 | 0.3731448 |
| 2 | C | C4 | -0.0441784 | 2.4200530 | 3.0959599 |
| 3 | C | C2 | 0.3917446 | 0.9285029 | 1.2304266 |
| 4 | C | C6 | 0.1932558 | 3.3240719 | 0.8705544 |
| 5 | C | C5 | -0.0312215 | 3.5181574 | 2.2355956 |
| 6 | C | C3 | 0.1679087 | 1.1329872 | 2.5983758 |
| 7 | H | H6 | 0.2045607 | 4.1739084 | 0.1930297 |
| 8 | C | C7 | 0.6594677 | -0.4356104 | 0.6749485 |
| 9 | C | C8 | -0.0604812 | -1.6558925 | 1.1030610 |
| 10 | C | C9 | -0.3990780 | -1.1480238 | -0.2537758 |
| 11 | H | H2 | 1.6880637 | -0.6259657 | 0.3677942 |
| 12 | H | H9 | -1.3489336 | -0.6193858 | -0.3044273 |
| 13 | F | F1 | -0.9952085 | -1.5969659 | 2.0833357 |
| 14 | F | F2 | 0.6411994 | -2.8117864 | 1.2177540 |
| 15 | C | C10 | 0.0217734 | -1.8588060 | -1.4791923 |
| 16 | C | C11 | -0.6265763 | -1.7904779 | -2.6471827 |
| 17 | H | H10 | -1.5257926 | -1.1758116 | -2.7142843 |
| 18 | H | H13 | -0.2193715 | 2.5626497 | 4.1591164 |
| 19 | H | H14 | -0.1950732 | 4.5194536 | 2.6249715 |
| 20 | H | H17 | 0.1518618 | 0.2821452 | 3.2714201 |
| 21 | H | H3 | 0.5698026 | 1.8869875 | -0.6903161 |
| 22 | H | H12 | 0.9179436 | -2.4736188 | -1.3967510 |
| 23 | C | C12 | -0.2172187 | -2.5078523 | -3.9001855 |
| 24 | H | H1 | 0.6855317 | -3.1076787 | -3.7429439 |
| 25 | H | H4 | -0.0183799 | -1.8012469 | -4.7175624 |
| 26 | H | H5 | -1.0125826 | -3.1783942 | -4.2528668 |

## Me_RHS_E_TS2 (TS2d)

G = -663.917301
iv = i184
S^2 = 0.5585

Cartesian Coordinates (Angstroms)

| | Atom | | X | Y | Z |
|---|---|---|---|---|---|
| 1 | C | C | -0.9151383 | 2.6513547 | -0.7786512 |
| 2 | C | C2 | -2.1077249 | 1.9853535 | -0.8954940 |
| 3 | C | C3 | -2.3582101 | 0.7899579 | -0.1888719 |
| 4 | C | C4 | 0.1849905 | 1.9441886 | -0.0714278 |
| 5 | C | C5 | 0.2556601 | 0.5076570 | -0.4856083 |
| 6 | H | H | -0.7255362 | 3.6365584 | -1.1899678 |
| 7 | H | H8 | -1.8833208 | 0.6491853 | 0.7747308 |
| 8 | H | H12 | -0.1358502 | 0.3017099 | -1.4732521 |
| 9 | F | F1 | 1.3945439 | 2.5835277 | -0.3012456 |
| 10 | F | F2 | 0.0234202 | 2.0493885 | 1.3265275 |
| 11 | C | C1 | 1.1528036 | -0.4591358 | 0.0724220 |
| 12 | C | C6 | 2.8706507 | -2.4587781 | 1.0962271 |
| 13 | C | C7 | 1.3491309 | -1.6865482 | -0.6134791 |
| 14 | C | C8 | 1.8458027 | -0.2716662 | 1.2975662 |
| 15 | C | C9 | 2.6926693 | -1.2560728 | 1.7896820 |
| 16 | C | C10 | 2.1907439 | -2.6682562 | -0.1092948 |
| 17 | H | H2 | 0.8298147 | -1.8495212 | -1.5553695 |
| 18 | H | H6 | 1.7092330 | 0.6511676 | 1.8469904 |
| 19 | H | H5 | 3.2177380 | -1.0883929 | 2.7266783 |
| 20 | H | H3 | 2.3247423 | -3.5980963 | -0.6560184 |
| 21 | H | H4 | 3.5316207 | -3.2251805 | 1.4915181 |
| 22 | H | H14 | -2.8650800 | 2.3500191 | -1.5911246 |
| 23 | C | C11 | -3.4330427 | -0.1819377 | -0.5617517 |
| 24 | H | H1 | -3.0292397 | -1.2005583 | -0.6427918 |
| 25 | H | H7 | -3.9040764 | 0.0698443 | -1.5176679 |
| 26 | H | H9 | -4.2163453 | -0.2257684 | 0.2096742 |

## nosub_RHS_SM (10e)

G = -624.665083
iv = none

Cartesian Coordinates (Angstroms)

| | Atom | | X | Y | Z |
|---|---|---|---|---|---|
| 1 | H | H1 | -0.7768006 | 1.0388829 | -1.6047737 |
| 2 | C | C1 | -0.7173554 | 1.4647263 | -0.6059672 |
| 3 | C | C4 | -0.5698059 | 2.5451006 | 1.9567121 |
| 4 | C | C2 | 0.1393784 | 0.8803837 | 0.3387670 |
| 5 | C | C6 | -1.4888334 | 2.5770693 | -0.2751774 |
| 6 | C | C5 | -1.4173630 | 3.1205023 | 1.0098283 |
| 7 | C | C3 | 0.2059673 | 1.4328222 | 1.6246422 |
| 8 | H | H6 | -2.1470385 | 3.0188171 | -1.0187338 |
| 9 | H | H5 | -2.0187004 | 3.9875695 | 1.2699744 |
| 10 | H | H3 | 0.8604990 | 0.9879185 | 2.3665809 |

| | | | | | |
|---|---|---|---|---|---|
| 11 | H | H4 | -0.5091567 | 2.9619231 | 2.9586068 |
| 12 | C | C7 | 0.9869728 | -0.2859276 | -0.0643017 |
| 13 | C | C8 | 1.2810050 | -1.4418712 | 0.8111093 |
| 14 | C | C9 | 0.3385846 | -1.7037254 | -0.3110794 |
| 15 | H | H9 | -0.7064157 | -1.7584663 | -0.0129154 |
| 16 | F | F1 | 0.8141422 | -1.4814324 | 2.0827236 |
| 17 | F | F2 | 2.5240203 | -1.9833723 | 0.7679361 |
| 18 | C | C10 | 0.7298449 | -2.5175481 | -1.4803324 |
| 19 | H | H8 | 1.7922604 | -2.5300748 | -1.7195707 |
| 20 | C | C11 | -0.1267143 | -3.2164337 | -2.2292022 |
| 21 | H | H10 | -1.1933096 | -3.2345539 | -2.0164315 |
| 22 | H | H2 | 1.7888044 | -0.0620621 | -0.7678205 |
| 23 | H | H13 | 0.2100143 | -3.8002475 | -3.0805750 |

## nosub_RHS_TS2 (TS2e)

G = -624.623111
iv = i300
S^2 = 0.5749

Cartesian Coordinates (Angstroms)

| | | Atom | X | Y | Z |
|---|---|---|---|---|---|
| 1 | C | C | -1.7874470 | 2.4421631 | -0.5045635 |
| 2 | C | C2 | -2.9103076 | 1.6546177 | -0.5179662 |
| 3 | C | C3 | -2.9306507 | 0.4028033 | 0.1255955 |
| 4 | C | C4 | -0.5366945 | 1.8213362 | 0.0097436 |
| 5 | C | C5 | -0.3654349 | 0.4249353 | -0.5081575 |
| 6 | H | H | -1.7528806 | 3.4631134 | -0.8681306 |
| 7 | H | H7 | -3.7853806 | 1.9730485 | -1.0847020 |
| 8 | H | H8 | -2.3409283 | 0.2191327 | 1.0131394 |
| 9 | H | H12 | -0.8595597 | 0.2241621 | -1.4499067 |
| 10 | F | F1 | 0.5605938 | 2.5993423 | -0.3282603 |
| 11 | F | F2 | -0.5231132 | 1.8293521 | 1.4181294 |
| 12 | C | C1 | 0.6996512 | -0.4548601 | -0.1310145 |
| 13 | C | C6 | 2.7580381 | -2.2748292 | 0.5294732 |
| 14 | C | C7 | 0.9343812 | -1.6177403 | -0.9109320 |
| 15 | C | C8 | 1.5307144 | -0.2392681 | 1.0000659 |
| 16 | C | C9 | 2.5445757 | -1.1348745 | 1.3131165 |
| 17 | C | C10 | 1.9442125 | -2.5118766 | -0.5846335 |
| 18 | H | H2 | 0.3072605 | -1.8002019 | -1.7805825 |
| 19 | H | H6 | 1.3680550 | 0.6346591 | 1.6181396 |
| 20 | H | H5 | 3.1740740 | -0.9480413 | 2.1792411 |
| 21 | H | H3 | 2.1048388 | -3.3940255 | -1.1988995 |
| 22 | H | H4 | 3.5500534 | -2.9732849 | 0.7852097 |
| 23 | H | H11 | -3.6840514 | -0.3396633 | -0.1241046 |

## AllylAlcohol_RHS_Z_SM (10f)

G = -739.152680
iv = none

Cartesian Coordinates (Angstroms)

| | | Atom | X | Y | Z |
|---|---|---|---|---|---|
| 1 | C | C1 | 0.0851050 | 1.6761281 | 0.3841732 |
| 2 | C | C4 | 0.9694170 | 2.3216998 | 2.9514251 |
| 3 | C | C2 | 0.7293453 | 0.7021926 | 1.1574226 |
| 4 | C | C6 | -0.1117169 | 2.9621284 | 0.8898064 |
| 5 | C | C5 | 0.3298641 | 3.2887861 | 2.1730192 |
| 6 | C | C3 | 1.1666918 | 1.0368216 | 2.4462655 |
| 7 | H | H6 | -0.6130479 | 3.7100865 | 0.2806242 |
| 8 | C | C7 | 0.9817207 | -0.6701300 | 0.6123821 |
| 9 | C | C8 | 0.3001452 | -1.8728140 | 1.1463146 |
| 10 | C | C9 | -0.1309859 | -1.4173229 | -0.2025879 |
| 11 | H | H2 | 1.9948860 | -0.8646646 | 0.2592903 |
| 12 | H | H9 | -1.0772748 | -0.8910040 | -0.2262031 |
| 13 | F | F1 | -0.5547505 | -1.7599330 | 2.1893523 |
| 14 | F | F2 | 1.0125521 | -3.0239942 | 1.2656796 |
| 15 | C | C10 | 0.2344924 | -2.1846184 | -1.4139185 |
| 16 | C | C11 | -0.2989358 | -2.0996594 | -2.6390618 |
| 17 | H | H10 | 0.1029440 | -2.7637087 | -3.4044095 |
| 18 | H | H13 | 1.3107232 | 2.5658500 | 3.9540720 |
| 19 | H | H14 | 0.1735724 | 4.2899330 | 2.5661627 |
| 20 | H | H17 | 1.6597545 | 0.2828651 | 3.0547075 |
| 21 | H | H3 | -0.2702189 | 1.4158108 | -0.6094971 |
| 22 | H | H12 | 1.0420510 | -2.9017490 | -1.2741580 |
| 23 | C | C12 | -1.4035451 | -1.2271609 | -3.1634237 |
| 24 | H | H1 | -2.2789188 | -1.8620928 | -3.3906656 |
| 25 | H | H4 | -1.0748721 | -0.8007048 | -4.1272752 |
| 26 | O | O1 | -1.7595442 | -0.1917930 | -2.2533476 |
| 27 | H | H5 | -2.5194536 | 0.2790478 | -2.6261489 |

## Allylalochol_RHS_Z_TS2 (TS2f)

G = -739.107323
iv = i244
S^2 = 0.6641

Cartesian Coordinates (Angstroms)

| | | Atom | X | Y | Z |
|---|---|---|---|---|---|
| 1 | C | C | -2.1259931 | 2.0740105 | -0.1662080 |
| 2 | C | C2 | -2.5424791 | 1.5174060 | 1.0086412 |
| 3 | C | C15 | -1.8016030 | 0.6027220 | 1.8031781 |
| 4 | C | C4 | -0.9168276 | 1.5576179 | -0.8649583 |
| 5 | C | C13 | -0.7384488 | 0.0873205 | -0.6639539 |
| 6 | H | H | -2.6797738 | 2.8649364 | -0.6622078 |
| 7 | H | H8 | -2.3837208 | -0.0497596 | 2.4558284 |
| 8 | H | H12 | -1.6672560 | -0.4609286 | -0.5726305 |
| 9 | F | F1 | -1.0227259 | 1.8395517 | -2.2240077 |

| | | | | | |
|---|---|---|---|---|---|
| 10 | F | F2 | 0.2272279 | 2.2801359 | -0.4820522 |
| 11 | C | C1 | 0.4300140 | -0.6726015 | -0.9764675 |
| 12 | C | C6 | 2.6656667 | -2.3058208 | -1.5618602 |
| 13 | C | C7 | 0.3417808 | -2.0913285 | -0.9412475 |
| 14 | C | C8 | 1.6894536 | -0.1010288 | -1.3069829 |
| 15 | C | C9 | 2.7777544 | -0.9110292 | -1.6028171 |
| 16 | C | C10 | 1.4388181 | -2.8908837 | -1.2260127 |
| 17 | H | H2 | -0.6111088 | -2.5494343 | -0.6868931 |
| 18 | H | H6 | 1.7894984 | 0.9759755 | -1.3396011 |
| 19 | H | H5 | 3.7265815 | -0.4526459 | -1.8683576 |
| 20 | H | H3 | 1.3419452 | -3.9726504 | -1.1924436 |
| 21 | H | H4 | 3.5248236 | -2.9305226 | -1.7894764 |
| 22 | H | H14 | -3.5589411 | 1.7394595 | 1.3403955 |
| 23 | C | C11 | -0.3624922 | 0.6962153 | 2.2256699 |
| 24 | H | H1 | 0.1722012 | 1.4506196 | 1.6412898 |
| 25 | H | H7 | 0.1431767 | -0.2710079 | 2.0799692 |
| 26 | O | O1 | -0.3830862 | 1.0317742 | 3.6228611 |
| 27 | H | H9 | 0.5255144 | 0.9418970 | 3.9503449 |

## Me_Z_RHS_SM (10g)

G = -664.955651
iv = none

Cartesian Coordinates (Angstroms)

| | Atom | | X | Y | Z |
|---|---|---|---|---|---|
| 1 | C | C1 | 0.6876844 | 1.8059308 | 0.2717546 |
| 2 | C | C4 | 0.2332772 | 2.1619593 | 2.9962718 |
| 3 | C | C2 | 0.6402783 | 0.6845481 | 1.1130431 |
| 4 | C | C6 | 0.5129610 | 3.0897137 | 0.7856787 |
| 5 | C | C5 | 0.2839789 | 3.2711096 | 2.1517316 |
| 6 | C | C3 | 0.4122952 | 0.8762936 | 2.4822057 |
| 7 | H | H6 | 0.5541235 | 3.9482377 | 0.1204040 |
| 8 | C | C7 | 0.8758488 | -0.6774199 | 0.5386953 |
| 9 | C | C8 | 0.1402163 | -1.8910747 | 0.9564978 |
| 10 | C | C9 | -0.2033816 | -1.3607236 | -0.3908768 |
| 11 | H | H2 | 1.8972229 | -0.8817126 | 0.2169921 |
| 12 | H | H9 | -1.1441406 | -0.8177792 | -0.4139133 |
| 13 | F | F1 | -0.7876269 | -1.8294679 | 1.9434392 |
| 14 | F | F2 | 0.8237780 | -3.0590947 | 1.0536399 |
| 15 | C | C10 | 0.2126043 | -2.0669876 | -1.6213846 |
| 16 | C | C11 | -0.3788681 | -2.0009782 | -2.8219581 |
| 17 | H | H13 | 0.0549811 | 2.2947349 | 4.0601739 |
| 18 | H | H14 | 0.1465586 | 4.2711907 | 2.5541931 |
| 19 | H | H17 | 0.3680571 | 0.0172382 | 3.1433062 |
| 20 | H | H3 | 0.8611049 | 1.6644457 | -0.7923559 |
| 21 | H | H5 | 0.0623687 | -2.5931285 | -3.6236434 |
| 22 | H | H12 | 1.0901639 | -2.7021730 | -1.5132039 |
| 23 | C | C12 | -1.5928016 | -1.2066712 | -3.2116891 |
| 24 | H | H1 | -1.3569106 | -0.5121795 | -4.0294335 |
| 25 | H | H4 | -2.0052938 | -0.6196340 | -2.3862491 |
| 26 | H | H7 | -2.3884795 | -1.8663773 | -3.5833190 |

## Me_Z_RHS_TS2 (TS2g)

G = -663.908400
iv = i271
S^2 = 0.7108

Cartesian Coordinates (Angstroms)

| | | Atom | X | Y | Z |
|---|---|---|---|---|---|
| 1 | C | C | -1.5504450 | 2.4776641 | -0.6191524 |
| 2 | C | C2 | -2.6238280 | 1.6386294 | -0.6718734 |
| 3 | C | C3 | -2.6681504 | 0.3067540 | -0.1672499 |
| 4 | C | C4 | -0.2008042 | 1.9546502 | -0.2616217 |
| 5 | C | C5 | 0.0096776 | 0.5600419 | -0.7584830 |
| 6 | H | H | -1.6142070 | 3.5236503 | -0.9017007 |
| 7 | H | H12 | -0.4798152 | 0.3547298 | -1.7024576 |
| 8 | F | F1 | 0.7668653 | 2.7997959 | -0.7985427 |
| 9 | F | F2 | 0.0214425 | 2.0575976 | 1.1224109 |
| 10 | C | C1 | 1.0293103 | -0.3495120 | -0.3479285 |
| 11 | C | C6 | 2.9841249 | -2.2674036 | 0.3711957 |
| 12 | C | C7 | 1.2144525 | -1.5409105 | -1.1033773 |
| 13 | C | C8 | 1.8610609 | -0.1597168 | 0.7899370 |
| 14 | C | C9 | 2.8220179 | -1.1026679 | 1.1298415 |
| 15 | C | C10 | 2.1707081 | -2.4804807 | -0.7487619 |
| 16 | H | H2 | 0.5883137 | -1.7070611 | -1.9770387 |
| 17 | H | H6 | 1.7452761 | 0.7351629 | 1.3868514 |
| 18 | H | H5 | 3.4518718 | -0.9319488 | 1.9991310 |
| 19 | H | H3 | 2.2898196 | -3.3812381 | -1.3451807 |
| 20 | H | H4 | 3.7347479 | -3.0020007 | 0.6493852 |
| 21 | H | H7 | -3.3665500 | -0.3611418 | -0.6760890 |
| 22 | H | H14 | -3.5119600 | 1.9904034 | -1.2035033 |
| 23 | C | C11 | -2.2362635 | -0.1744225 | 1.1888731 |
| 24 | H | H1 | -1.5813535 | 0.5328307 | 1.6967027 |
| 25 | H | H8 | -1.7263941 | -1.1446348 | 1.1324948 |
| 26 | H | H9 | -3.1299182 | -0.3287709 | 1.8161379 |

## CN_Z_RHS_SM (10h)

Spartan'10: G = -716.912699, iv = none
Gaussian'09: B3LYP/6-31G*, G =-716.917609, iv = none
Gaussian'09: M05-2X/6-31G*, G = -716.877091, iv = none

Cartesian Coordinates (Angstroms)

| | | Atom | X | Y | Z |
|---|---|---|---|---|---|
| 1 | C | C1 | 0.8179847 | 0.4610398 | -1.8635220 |
| 2 | C | C2 | -0.3416567 | 0.4689995 | -0.9506254 |
| 3 | C | C3 | 0.4045255 | -0.8718587 | -1.3259301 |
| 4 | H | H3 | -1.2995784 | 0.5872061 | -1.4573460 |
| 5 | H | H6 | 1.0320986 | -1.2635388 | -0.5294869 |
| 6 | C | C4 | -0.2651222 | 0.9957873 | 0.4498269 |
| 7 | C | C5 | -0.1980338 | 2.0131451 | 3.0630712 |
| 8 | C | C6 | -0.7499435 | 0.2341961 | 1.5211498 |
| 9 | C | C7 | 0.2576765 | 2.2709732 | 0.7012935 |

| | | | | | |
|---|---|---|---|---|---|
| 10 | C | C8 | 0.2898950 | 2.7763162 | 2.0014705 |
| 11 | C | C9 | -0.7179257 | 0.7399927 | 2.8203014 |
| 12 | H | H4 | -1.1462860 | -0.7610274 | 1.3358170 |
| 13 | H | H7 | 0.6466198 | 2.8635872 | -0.1213348 |
| 14 | H | H8 | 0.7007166 | 3.7655608 | 2.1843421 |
| 15 | H | H9 | -1.0945771 | 0.1378479 | 3.6425950 |
| 16 | H | H10 | -0.1699119 | 2.4064027 | 4.0757033 |
| 17 | C | C10 | -0.2505385 | -1.8537494 | -2.1986835 |
| 18 | H | H11 | -0.7665948 | -1.4637915 | -3.0729015 |
| 19 | C | C11 | -0.2592490 | -3.1874688 | -2.0083162 |
| 20 | H | H12 | -0.7683717 | -3.8396237 | -2.7121403 |
| 21 | F | F1 | 1.9994365 | 0.9831711 | -1.4741208 |
| 22 | F | F2 | 0.6103896 | 0.7185857 | -3.1784391 |
| 23 | C | C12 | 0.3781323 | -3.8268640 | -0.9027312 |
| 24 | N | N1 | 0.8903143 | -4.3548891 | 0.0000069 |

## CN_Z_RHS_TS2 (TS2h)

Spartan'10: G = -716.865358, iv = i141, S^2 = 0.6948
Gaussian'09: B3LYP/6-31G*, G = -716.870285, iv = i165, S^2 = 0.71
Gaussian'09: M05-2X/6-31G*, G = -716.822858, iv = i157, S^2 = 0.87

Cartesian Coordinates (Angstroms)

| | | Atom | X | Y | Z |
|---|---|---|---|---|---|
| 1 | C | C | -1.6959458 | 2.4322037 | -0.5853651 |
| 2 | C | C2 | -2.8320034 | 1.6728406 | -0.5751580 |
| 3 | C | C3 | -2.9807398 | 0.3998630 | 0.0375008 |
| 4 | C | C4 | -0.4175204 | 1.8559479 | -0.1169702 |
| 5 | C | C5 | -0.2444133 | 0.4520397 | -0.6140125 |
| 6 | H | H | -1.6942970 | 3.4655487 | -0.9128003 |
| 7 | H | H7 | -3.6967941 | 2.0395279 | -1.1290636 |
| 8 | H | H12 | -0.8219802 | 0.2020451 | -1.4936243 |
| 9 | F | F1 | 0.6514990 | 2.6240699 | -0.5853241 |
| 10 | F | F2 | -0.2852804 | 1.9416445 | 1.2749634 |
| 11 | C | C1 | 0.8219841 | -0.4094148 | -0.2537761 |
| 12 | C | C6 | 2.8634354 | -2.2330723 | 0.4205645 |
| 13 | C | C7 | 1.0618240 | -1.5728641 | -1.0405546 |
| 14 | C | C8 | 1.6432015 | -0.1977193 | 0.8914358 |
| 15 | C | C9 | 2.6488791 | -1.0981051 | 1.2106711 |
| 16 | C | C10 | 2.0650588 | -2.4670966 | -0.7082571 |
| 17 | H | H2 | 0.4437234 | -1.7493459 | -1.9171956 |
| 18 | H | H6 | 1.4717272 | 0.6691657 | 1.5142291 |
| 19 | H | H5 | 3.2624894 | -0.9291043 | 2.0913627 |
| 20 | H | H3 | 2.2336450 | -3.3494409 | -1.3192103 |
| 21 | H | H4 | 3.6455019 | -2.9392286 | 0.6860434 |
| 22 | H | H9 | -3.8222598 | -0.2064568 | -0.3046222 |
| 23 | C | C11 | -2.3987703 | -0.0760801 | 1.2324707 |
| 24 | N | N1 | -1.9229643 | -0.5269678 | 2.1966924 |

## Me_CO2Et_RHS_SM (10i)

Spartan'10, G = -891.796469, iv = none
Gaussian'09: B3LYP/6-31G*, G = -891.805001, iv = none
Gaussian'09: M05-2X/6-31G*, G = -891.739337, iv = none

Cartesian Coordinates (Angstroms)

| | Atom | | X | Y | Z |
|---|---|---|---|---|---|
| 1 | H | H1 | 1.0446575 | 2.2529926 | 0.3038655 |
| 2 | C | C1 | 0.6803316 | 2.4282900 | 1.3133353 |
| 3 | C | C4 | -0.2625032 | 2.8772000 | 3.8951227 |
| 4 | C | C2 | 0.2451012 | 1.3409889 | 2.0855825 |
| 5 | C | C6 | 0.6461051 | 3.7238963 | 1.8257449 |
| 6 | C | C5 | 0.1726455 | 3.9521526 | 3.1200901 |
| 7 | C | C3 | -0.2258138 | 1.5785692 | 3.3836570 |
| 8 | H | H6 | 0.9875777 | 4.5551942 | 1.2146256 |
| 9 | H | H5 | 0.1441892 | 4.9618900 | 3.5207809 |
| 10 | H | H3 | -0.5693807 | 0.7480578 | 3.9909839 |
| 11 | H | H4 | -0.6314050 | 3.0461543 | 4.9033250 |
| 12 | C | C7 | 0.3355870 | -0.0422941 | 1.5220763 |
| 13 | C | C8 | -0.6565510 | -1.1087543 | 1.7646985 |
| 14 | C | C9 | -0.6767509 | -0.5166698 | 0.3952902 |
| 15 | H | H2 | 1.3441159 | -0.4219058 | 1.3611400 |
| 16 | H | H9 | -1.4879302 | 0.1870095 | 0.2353990 |
| 17 | F | F1 | -1.7181082 | -0.8976478 | 2.5784862 |
| 18 | F | F2 | -0.2105188 | -2.3788247 | 1.9208703 |
| 19 | C | C10 | -0.1732717 | -1.2781974 | -0.7575398 |
| 20 | H | H8 | 0.5383231 | -2.0645695 | -0.5233232 |
| 21 | C | C11 | -0.5062844 | -1.0987494 | -2.0513345 |
| 22 | C | C12 | 0.0974987 | -1.9667538 | -3.1024499 |
| 23 | O | O1 | -0.1662135 | -1.8665359 | -4.2864012 |
| 24 | O | O2 | 0.9733092 | -2.8843421 | -2.6229824 |
| 25 | C | C13 | 1.5644962 | -3.7388047 | -3.6115307 |
| 26 | H | H7 | 2.2118431 | -4.4205317 | -3.0585095 |
| 27 | H | H11 | 2.1468352 | -3.1535082 | -4.3286787 |
| 28 | H | H12 | 0.7938764 | -4.2943522 | -4.1526963 |
| 29 | C | C14 | -1.4727345 | -0.0764040 | -2.5904011 |
| 30 | H | H10 | -0.9791214 | 0.5663472 | -3.3277598 |
| 31 | H | H13 | -1.8904669 | 0.5564653 | -1.8044602 |
| 32 | H | H14 | -2.2994382 | -0.5663625 | -3.1170062 |

## Me_CO2Et_RHS_TS2 (TS2i)

Spartan'10: G = -891.751323, iv = i100, S^2 = 0.7421
Gaussian'09: B3LYP/6-31G*, G = -891.756292, iv = i92, S^2 = 0.75
Gaussian'09: M05-2X/6-31G*, G = -891.68717, iv = i70, S^2 = 0.91

Cartesian Coordinates (Angstroms)

| | Atom | | X | Y | Z |
|---|---|---|---|---|---|
| 1 | C | C | -0.7492316 | 2.8416276 | -0.6069933 |
| 2 | C | C2 | -1.8256500 | 2.0339074 | -0.4681444 |
| 3 | C | C3 | -1.8311064 | 0.7379227 | 0.1677036 |
| 4 | C | C4 | 0.6373249 | 2.3487772 | -0.3422518 |
| 5 | C | C5 | 0.8153452 | 0.9098674 | -0.7192309 |
| 6 | H | H | -0.8360057 | 3.8517814 | -0.9954873 |
| 7 | H | H7 | -2.7673212 | 2.3486960 | -0.9174200 |
| 8 | H | H12 | 0.2552825 | 0.6281755 | -1.6035435 |
| 9 | F | F1 | 1.5256631 | 3.1226515 | -1.0794369 |
| 10 | F | F2 | 1.0038965 | 2.6003383 | 0.9844375 |
| 11 | C | C1 | 1.8685020 | 0.0389292 | -0.3180248 |
| 12 | C | C6 | 3.9090872 | -1.7988735 | 0.3628609 |
| 13 | C | C7 | 2.0633196 | -1.1621470 | -1.0576450 |
| 14 | C | C8 | 2.7324120 | 0.2773956 | 0.7875127 |
| 15 | C | C9 | 3.7319599 | -0.6297908 | 1.1116369 |
| 16 | C | C10 | 3.0667499 | -2.0588718 | -0.7258170 |
| 17 | H | H2 | 1.4142271 | -1.3625323 | -1.9066413 |
| 18 | H | H6 | 2.6102043 | 1.1790719 | 1.3722462 |
| 19 | H | H5 | 4.3820658 | -0.4266731 | 1.9585224 |
| 20 | H | H3 | 3.2014201 | -2.9617152 | -1.3153402 |
| 21 | H | H4 | 4.6952357 | -2.5015952 | 0.6247043 |
| 22 | C | C11 | -2.7852724 | -0.2244240 | -0.4164488 |
| 23 | O | O1 | -3.3895750 | -0.0539514 | -1.4651962 |
| 24 | O | O2 | -2.9290712 | -1.3403758 | 0.3432352 |
| 25 | C | C12 | -3.8551082 | -2.3092594 | -0.1659952 |
| 26 | H | H1 | -4.8430391 | -1.8615727 | -0.3058310 |
| 27 | H | H9 | -3.8935191 | -3.1006354 | 0.5838020 |
| 28 | H | H10 | -3.5102835 | -2.7061803 | -1.1254549 |
| 29 | C | C13 | -1.2696131 | 0.4568753 | 1.5383462 |
| 30 | H | H8 | -0.6997947 | -0.4782820 | 1.5628785 |
| 31 | H | H11 | -2.0971278 | 0.3325175 | 2.2523740 |
| 32 | H | H13 | -0.6309769 | 1.2683454 | 1.8846422 |

**Other Synthesised VCP Precursors**

## Me Furan SM Trans (S9a)

G = -697.705753
iv = none

Cartesian Coordinates (Angstroms)

| | Atom | | X | Y | Z |
|---|---|---|---|---|---|
| 1 | C | C7 | 0.1406689 | -0.9956178 | 0.5964871 |
| 2 | C | C8 | 1.3872171 | -1.1047379 | 1.3823601 |
| 3 | C | C9 | 0.4327958 | -0.0392131 | 1.8146329 |
| 4 | H | H2 | -0.5882604 | -1.7749922 | 0.8259152 |
| 5 | H | H9 | 0.7063655 | 0.9810075 | 1.5644678 |
| 6 | F | F1 | 2.5620693 | -0.7294608 | 0.8469569 |
| 7 | F | F2 | 1.5578818 | -2.1889554 | 2.1726557 |
| 8 | C | C10 | -0.3889264 | -0.2021742 | 3.0432596 |
| 9 | H | H12 | -0.6630748 | -1.2485563 | 3.2963319 |
| 10 | O | O1 | -0.7568556 | 0.7285319 | 3.7269647 |
| 11 | C | C1 | 0.1434540 | -0.4465200 | -0.7726300 |
| 12 | O | O2 | 0.9128662 | -1.1269062 | -1.6855271 |
| 13 | C | C3 | 0.7601324 | -0.4932891 | -2.8790145 |
| 14 | C | C4 | -0.0823992 | 0.5655587 | -2.7531779 |
| 15 | C | C5 | -0.4971161 | 0.6019962 | -1.3757939 |
| 16 | H | H6 | 1.3135119 | -0.9125578 | -3.7057550 |
| 17 | H | H8 | -0.3834437 | 1.2479830 | -3.5367193 |
| 18 | C | C2 | -1.4470105 | 1.5859241 | -0.7584351 |
| 19 | H | H1 | -1.0728967 | 2.6131767 | -0.8500036 |
| 20 | H | H3 | -1.6130408 | 1.3840237 | 0.3032771 |
| 21 | H | H4 | -2.4239387 | 1.5547792 | -1.2562526 |

## Me Furan SM cis (S9b)

G = -697.706140
iv = none

Cartesian Coordinates (Angstroms)

| | Atom | | X | Y | Z |
|---|---|---|---|---|---|
| 1 | C | C7 | 0.7390908 | 0.3844384 | -1.0657176 |
| 2 | C | C8 | 1.6688781 | -0.7588474 | -0.9599335 |
| 3 | C | C9 | 2.1938189 | 0.5450926 | -0.4611973 |
| 4 | H | H9 | 2.9086128 | 1.0603466 | -1.0954608 |
| 5 | F | F1 | 2.1952401 | -1.2833724 | -2.0859645 |
| 6 | F | F2 | 1.4154475 | -1.7446922 | -0.0696108 |
| 7 | C | C10 | 2.3730149 | 0.7819041 | 0.9998585 |
| 8 | H | H12 | 1.6535859 | 0.2669332 | 1.6659712 |
| 9 | O | O1 | 3.2535027 | 1.4874759 | 1.4429023 |
| 10 | C | C1 | -0.4487462 | 0.5029065 | -0.1991059 |
| 11 | O | O2 | -0.6162882 | 1.7157256 | 0.4288353 |
| 12 | C | C3 | -1.7758070 | 1.6365772 | 1.1365563 |
| 13 | C | C4 | -2.3502310 | 0.4161752 | 0.9749909 |
| 14 | C | C5 | -1.4901531 | -0.3344543 | 0.0984126 |

```
 15 H   H6          -2.0449353     2.5206780     1.6946725
 16 H   H8          -3.2754549     0.0746172     1.4194284
 17 C   C2          -1.7172756    -1.7403138    -0.3741531
 18 H   H1          -1.6172550    -2.4576887     0.4494956
 19 H   H3          -1.0063412    -2.0322880    -1.1511297
 20 H   H4          -2.7278852    -1.8545588    -0.7834508
 21 H   H5           0.6691809     0.8133453    -2.0653994
```

## Me Furan [3,3]-TS (TS3)

```
G = -697.672991
iv = i309
   Cartesian Coordinates (Angstroms)
       Atom             X             Y             Z
    ---------    ------------- ------------- -------------

  1 C   C1          -0.9655277    -0.1281625     0.5395914
  2 C   C2           0.3919177     0.1289556     0.9945501
  3 C   C5           0.2274272     0.8396342    -1.9004649
  4 H   H7           0.1609263     1.7533491    -2.5101504
  5 O   O1          -0.8684853     0.4606492    -1.3383232
  6 C   C3           1.4773684     0.2600752    -1.8088386
  7 C   C4           1.6018603    -0.1920409     0.4785488
  8 C   C11          1.8373657    -0.8215650    -0.8598650
  9 H   H2           2.3096381     0.7303558    -2.3220689
 10 H   H10          2.4830843     0.2571453     0.9257838
 11 F   F1           1.1678797    -2.0112550    -1.0464222
 12 F   F2           3.1739584    -1.1193241    -0.9750310
 13 O   O3           0.3647330     1.1247164     1.9796098
 14 C   C8          -0.9295091     1.5043356     2.1026746
 15 H   H12         -1.1124427     2.2748555     2.8394582
 16 C   C7          -1.7628534     0.8250046     1.2825503
 17 H   H11         -2.8371378     0.9257828     1.2236656
 18 C   C9          -1.5565897    -1.4572627     0.1605722
 19 H   H3          -0.8663429    -2.0669327    -0.4148454
 20 H   H4          -2.4636009    -1.2919639    -0.4275625
 21 H   H9          -1.8336694    -1.9963523     1.0765677
```

## Me Furan VCPR Ts (use NODISS keyword) (TS4)

```
G = -697.665879
iv = i234
S^2 = 0.29

Cartesian Coordinates (Angstroms)
       Atom             X             Y             Z
    ---------    ------------- ------------- -------------

  1 C   C           -1.4362337     2.2538247    -1.3566871
  2 C   C2          -2.5135153     1.5219083    -1.8423498
  3 C   C4          -0.5217870     1.5210494    -0.4698253
  4 C   C5          -0.3577867     0.0914781    -0.9001564
  5 H   H           -1.2556072     3.2975305    -1.5802847
  6 H   H12         -0.6239173    -0.1605270    -1.9144912
  7 F   F1           0.7415242     2.1148315    -0.4410394
  8 F   F2          -0.9294237     1.5682960     0.8718760
```

```
  9 H   H14          -3.1555660     1.9765040    -2.6177622
 10 O   O1           -2.7358741     0.3260619    -1.4868078
 11 C   C1            0.3479232    -0.8708234    -0.1989309
 12 O   O2            0.4898193    -2.0924328    -0.8613604
 13 C   C6            1.1870195    -2.9027068    -0.0585477
 14 C   C7            1.5357917    -2.2711910     1.1116651
 15 C   C8            1.0070982    -0.9638114     1.0508234
 16 H   H5            1.3664614    -3.9011673    -0.4315501
 17 H   H7            2.1094802    -2.6960812     1.9238066
 18 C   C3            1.1627819     0.0895214     2.0983756
 19 H   H2            1.9011221    -0.2221925     2.8422719
 20 H   H3            0.2090776     0.2796994     2.6039992
 21 H   H4            1.4716117     1.0402281     1.6569753
```

## Non-Fluoro-SM trans (47a)

```
Spartan'10: G = -654.027526, iv = none
Gaussian'09: B3LYP/6-31G*, G = -654.032624 , iv = none
Gaussian'09: M05-2X/6-31G*, G = -653.968582, iv = none

   Cartesian Coordinates (Angstroms)
       Atom            X             Y             Z
    ---------    ------------- ------------- -------------

  1 H   H1            2.4558148     1.9664977     1.3490937
  2 C   C1            1.5137206     2.2423943     1.8174568
  3 C   C4           -0.8907964     2.9604966     3.0162407
  4 C   C2            0.4921939     1.2865397     1.9171313
  5 C   C6            1.3395373     3.5362157     2.3087436
  6 C   C5            0.1349941     3.9019932     2.9103722
  7 C   C3           -0.7135294     1.6673011     2.5267193
  8 H   H6            2.1472528     4.2581074     2.2200627
  9 H   H5           -0.0036711     4.9091323     3.2937697
 10 H   H3           -1.5254456     0.9506187     2.6227930
 11 H   H4           -1.8335371     3.2322952     3.4841012
 12 C   C7            0.7180306    -0.0812305     1.3680533
 13 C   C8            0.0127924    -1.3021650     1.8856731
 14 C   C9           -0.3721006    -0.7724019     0.5197991
 15 H   H2            1.7317359    -0.2449302     1.0093980
 16 H   H9           -1.3118509    -0.2275742     0.4625525
 17 C   C10           0.0192075    -1.4923450    -0.6960636
 18 H   H8            0.9411453    -2.0733622    -0.6593643
 19 C   C11          -0.6623738    -1.4863038    -1.8532113
 20 H   H10          -1.5889776    -0.9328562    -1.9759883
 21 C   C12          -0.1648266    -2.2407264    -3.0192128
 22 O   O1            0.8483886    -2.9142415    -3.0597069
 23 O   O2           -0.9984269    -2.0894137    -4.0801680
 24 C   C13          -0.6046241    -2.7846876    -5.2704153
 25 H   H7           -1.3671695    -2.5496443    -6.0140814
 26 H   H11          -0.5623012    -3.8628738    -5.0915100
 27 H   H12           0.3786244    -2.4463030    -5.6097971
 28 H   H13          -0.7063055    -1.1776952     2.6905673
 29 H   H14           0.5724986    -2.2328375     1.9269918
```

## Non-Fluoro-SM cis (47b)

Spartan'10: G = -654.024790, iv = none
Gaussian'09: B3LYP/6-31G*, -654.030414, iv = none
Gaussian'09: M06-2X/6-31G*, -653.744967, iv = none

Cartesian Coordinates (Angstroms)

| | Atom | | X | Y | Z |
|---|---|---|---|---|---|
| 1 | H | H1 | -0.2542662 | 2.2915662 | -0.5021848 |
| 2 | C | C1 | 0.7344812 | 2.1645972 | -0.0684421 |
| 3 | C | C4 | 3.2703973 | 1.8162921 | 1.0317255 |
| 4 | C | C2 | 0.8557636 | 1.5844524 | 1.2029998 |
| 5 | C | C6 | 1.8639337 | 2.5634010 | -0.7812665 |
| 6 | C | C5 | 3.1369438 | 2.3904917 | -0.2325234 |
| 7 | C | C3 | 2.1367416 | 1.4171786 | 1.7429408 |
| 8 | H | H6 | 1.7515622 | 3.0044603 | -1.7682719 |
| 9 | H | H5 | 4.0179587 | 2.6984646 | -0.7892128 |
| 10 | H | H3 | 2.2450075 | 0.9756370 | 2.7302087 |
| 11 | H | H4 | 4.2566489 | 1.6773723 | 1.4669251 |
| 12 | C | C7 | -0.3721506 | 1.2032446 | 1.9764382 |
| 13 | C | C8 | -0.4452043 | -0.0400410 | 2.8065827 |
| 14 | C | C9 | -1.2889387 | 0.0486890 | 1.5393170 |
| 15 | H | H9 | -2.3527386 | 0.2234564 | 1.6844538 |
| 16 | C | C10 | -0.9508286 | -0.7867763 | 0.3807042 |
| 17 | H | H8 | 0.1013634 | -1.0033740 | 0.2018760 |
| 18 | C | C11 | -1.8446828 | -1.3043687 | -0.4777387 |
| 19 | H | H10 | -2.9127551 | -1.1332973 | -0.3753246 |
| 20 | C | C12 | -1.4011137 | -2.1362181 | -1.6124187 |
| 21 | O | O1 | -0.2530060 | -2.4438418 | -1.8737535 |
| 22 | O | O2 | -2.4635381 | -2.5336285 | -2.3607591 |
| 23 | C | C13 | -2.1367419 | -3.3489422 | -3.4935561 |
| 24 | H | H7 | -1.6065193 | -4.2521592 | -3.1792809 |
| 25 | H | H11 | -1.5049799 | -2.7981740 | -4.1966114 |
| 26 | H | H12 | -3.0903901 | -3.6047972 | -3.9573000 |
| 27 | H | H13 | -0.9306797 | 2.0551654 | 2.3645298 |
| 28 | H | H2 | -0.9651543 | -0.0097442 | 3.7598570 |
| 29 | H | H14 | 0.4028861 | -0.7191062 | 2.7800860 |

## non-fluoro-VCPR TS trans (TS5a)

Spartan'10: -653.972598, iv = i236, S^2 = 0.7146
Gaussian'09: B3LYP/6-31G*, G = -653.976701, iv = i246, S^2 = 0.73
Gaussian'09: M05-2X/6-31+G*, G = -653.90781, iv = i262, S^2 = 0.84

Cartesian Coordinates (Angstroms)

| | Atom | | X | Y | Z |
|---|---|---|---|---|---|
| 1 | C | C | -0.8222470 | 3.0250060 | -0.4539541 |
| 2 | C | C2 | -1.9052459 | 2.2106773 | -0.3804376 |
| 3 | C | C3 | -1.7973923 | 0.8903636 | 0.1578220 |
| 4 | C | C4 | 0.5337323 | 2.4619301 | -0.0897511 |
| 5 | C | C5 | 0.6885928 | 1.0683410 | -0.6550086 |
| 6 | H | H | -0.9020935 | 4.0478346 | -0.8109030 |

```
  7 H   H7             -2.8638439     2.5116103     -0.7984342
  8 H   H8             -1.1422527     0.6749249      0.9926968
  9 H   H12             0.2525740     0.9082878     -1.6370826
 10 C   C1              1.6275974     0.0969890     -0.1977950
 11 C   C6              3.4438317    -1.9011993      0.6552119
 12 C   C7              1.7957586    -1.1120289     -0.9276113
 13 C   C8              2.4099294     0.2652341      0.9762530
 14 C   C9              3.2978055    -0.7199932      1.3906673
 15 C   C10             2.6850935    -2.0892635     -0.5080156
 16 H   H2              1.2071244    -1.2629430     -1.8294584
 17 H   H6              2.3250788     1.1801860      1.5546033
 18 H   H5              3.8876218    -0.5662147      2.2907224
 19 H   H3              2.7948555    -3.0029020     -1.0863701
 20 H   H4              4.1438640    -2.6655275      0.9811905
 21 C   C11            -2.7745767    -0.1316268     -0.2028367
 22 O   O1             -3.6664012    -0.0019144     -1.0287858
 23 O   O2             -2.5783681    -1.2815095      0.5006099
 24 C   C12            -3.4934341    -2.3395480      0.1952483
 25 H   H1             -4.5241878    -2.0317324      0.3947253
 26 H   H9             -3.2103695    -3.1704373      0.8431085
 27 H   H10            -3.4145251    -2.6308234     -0.8566405
 28 H   H11             1.3311852     3.1187271     -0.4745862
 29 H   H13             0.6702929     2.4475518      1.0048117
```

## non-fluoro-VCPR TS cis (TS5b)

```
Spartan'10:
G = -654.971681,
iv = i237,
S^2 = 0.7337

Cartesian Coordinates (Angstroms)
       Atom             X             Y             Z
    ---------    ------------- ------------- -------------

  1 C   C               0.2453010     2.5048609      1.2953703
  2 C   C2             -0.8039811     1.7101068      1.6249380
  3 C   C3             -0.6682026     0.3122649      1.8687047
  4 C   C4              1.6121357     1.9196713      0.9916982
  5 C   C5              1.5785997     0.5091379      0.4474431
  6 H   H               0.1262525     3.5838445      1.2259920
  7 H   H7             -1.8111301     2.1230173      1.6485146
  8 H   H8              0.1915083    -0.0898749      2.3880552
  9 C   C11            -1.8097139    -0.5797378      1.6968693
 10 O   O1             -2.9124260    -0.2548200      1.2832605
 11 O   O2             -1.5012132    -1.8571654      2.0611001
 12 C   C12            -2.5659716    -2.8020317      1.9057740
 13 H   H1             -3.4406680    -2.5021625      2.4904006
 14 H   H9             -2.1715884    -3.7530990      2.2671430
 15 H   H10            -2.8605324    -2.8857106      0.8549572
 16 H   H11             2.1336028     2.5856755      0.2877626
 17 H   H13             2.2259734     1.9238677      1.9062310
 18 H   H16             2.0380922    -0.2751250      1.0425867
 19 C   C1              1.2934012     0.1999010     -0.9135336
 20 C   C6              0.6974007    -0.4795327     -3.5935287
 21 C   C7              0.8119491     1.1739275     -1.8285683
```

| | | | | | |
|---|---|---|---|---|---|
| 22 | C | C8 | 1.4690232 | -1.1260625 | -1.3953192 |
| 23 | C | C9 | 1.1765396 | -1.4563796 | -2.7094170 |
| 24 | C | C10 | 0.5172298 | 0.8326852 | -3.1429608 |
| 25 | H | H2 | 0.6605875 | 2.1940342 | -1.4909004 |
| 26 | H | H6 | 1.8339548 | -1.8874860 | -0.7099350 |
| 27 | H | H5 | 1.3199579 | -2.4769201 | -3.0548471 |
| 28 | H | H3 | 0.1450793 | 1.5932011 | -3.8244866 |
| 29 | H | H4 | 0.4688384 | -0.7400881 | -4.6233044 |

## non fluoro VCP- [3,3] TS (S-TS13)

Spartan'10 G = -653.971614, iv = i403
Gaussian'09: B3LYP/6-31G*, -653.973922, iv = i403
Gaussian'09: M06-2X/6-31G*, -653.686835, iv = i400

Cartesian Coordinates (Angstroms)

| | | Atom | X | Y | Z |
|---|---|---|---|---|---|
| 1 | C | C1 | -0.0894029 | -0.6486226 | 0.8616396 |
| 2 | H | H3 | -0.2343972 | -1.7199948 | 0.7606184 |
| 3 | C | C2 | 1.2368519 | -0.2540877 | 1.3416020 |
| 4 | C | C5 | 1.2386251 | -0.1502183 | -1.6576682 |
| 5 | H | H7 | 1.3283122 | 0.7889579 | -2.1995328 |
| 6 | C | C6 | -0.0737839 | -0.5112904 | -1.2033351 |
| 7 | H | H6 | -0.3526574 | -1.5564982 | -1.2871950 |
| 8 | C | C7 | 1.3398635 | 0.9153519 | 2.1770254 |
| 9 | H | H4 | 2.3328995 | 1.2425020 | 2.4775117 |
| 10 | C | C8 | 0.2341933 | 1.6054176 | 2.5757404 |
| 11 | H | H9 | 0.3352882 | 2.4889856 | 3.1999974 |
| 12 | C | C9 | -1.0757048 | 1.1624901 | 2.1900805 |
| 13 | H | H11 | -1.9457203 | 1.6985261 | 2.5607054 |
| 14 | C | C10 | -1.2329787 | 0.0613238 | 1.4049207 |
| 15 | H | H12 | -2.2246786 | -0.2981886 | 1.1479309 |
| 16 | C | C3 | 2.3703942 | -0.8199918 | -1.2934753 |
| 17 | C | C4 | 2.3739013 | -0.8979018 | 0.8956232 |
| 18 | C | C11 | 2.3917636 | -1.8913224 | -0.2310380 |
| 19 | H | H2 | 3.3360345 | -0.4478167 | -1.6272447 |
| 20 | H | H10 | 3.3402224 | -0.5319360 | 1.2370583 |
| 21 | C | C12 | -1.1691115 | 0.4082697 | -1.6053860 |
| 22 | O | O1 | -1.0412943 | 1.5755153 | -1.9183351 |
| 23 | O | O2 | -2.3775113 | -0.2147373 | -1.5535430 |
| 24 | C | C13 | -3.5017889 | 0.6153777 | -1.8776802 |
| 25 | H | H1 | -3.4106571 | 1.0103859 | -2.8931257 |
| 26 | H | H5 | -3.5750941 | 1.4536613 | -1.1791992 |
| 27 | H | H8 | -4.3774856 | -0.0300456 | -1.7958804 |
| 28 | H | H13 | 3.3004002 | -2.4970704 | -0.2515753 |
| 29 | H | H14 | 1.5235165 | -2.5570425 | -0.2562403 |

## piperonyl cis SM (48)

Spartan'10: G = -1041.02078, iv = none
Gaussian'09: B3LYP/6-31G* G = -1041.028189, iv = none
Gaussian'09: M06-2X/6-31G* G = -1040.629122, iv = none

Cartesian Coordinates (Angstroms)

| | Atom | | X | Y | Z |
|---|---|---|---|---|---|
| 1 | C | C1 | 3.0423844 | -0.2086799 | 0.8498853 |
| 2 | C | C2 | 1.9712958 | 0.6511022 | 1.3910393 |
| 3 | C | C3 | 1.8273286 | -0.9333291 | 1.3301227 |
| 4 | H | H6 | 1.9311472 | -1.4268921 | 2.2931801 |
| 5 | C | C10 | 0.9105989 | -1.5575832 | 0.3680707 |
| 6 | H | H11 | 0.8299722 | -1.1099408 | -0.6198009 |
| 7 | C | C11 | 0.1704137 | -2.6453405 | 0.6293754 |
| 8 | H | H12 | 0.1962986 | -3.1500943 | 1.5910290 |
| 9 | C | C12 | -0.7183097 | -3.2114163 | -0.4070501 |
| 10 | O | O1 | -0.8667601 | -2.7810948 | -1.5347917 |
| 11 | O | O2 | -1.3693752 | -4.3033018 | 0.0663863 |
| 12 | C | C13 | -2.2558576 | -4.9350251 | -0.8680243 |
| 13 | H | H5 | -3.0221314 | -4.2329708 | -1.2081799 |
| 14 | H | H13 | -2.7091715 | -5.7665514 | -0.3271395 |
| 15 | H | H14 | -1.7017856 | -5.2994578 | -1.7376918 |
| 16 | F | F1 | 3.3167039 | -0.2109170 | -0.4764377 |
| 17 | F | F2 | 4.1926516 | -0.3416594 | 1.5506246 |
| 18 | C | C4 | 1.1090415 | 1.5624446 | 0.5757094 |
| 19 | C | C5 | 0.8036928 | 3.1156355 | -1.2954926 |
| 20 | C | C6 | -0.9956292 | 2.6403948 | 0.2548302 |
| 21 | C | C7 | -0.4946950 | 3.2982780 | -0.8682452 |
| 22 | C | C9 | 1.6034720 | 2.2303373 | -0.5494306 |
| 23 | H | H1 | 1.1902327 | 3.6322954 | -2.1670758 |
| 24 | H | H4 | 2.6255347 | 2.0574505 | -0.8640583 |
| 25 | C | C8 | -0.2261551 | 1.7713216 | 0.9962538 |
| 26 | H | H2 | 2.1680653 | 0.9832609 | 2.4097456 |
| 27 | H | H3 | -0.6311615 | 1.2628101 | 1.8648840 |
| 28 | O | O3 | -2.2980178 | 3.0223465 | 0.4704360 |
| 29 | O | O4 | -1.4650229 | 4.1142322 | -1.3960229 |
| 30 | C | C14 | -2.6615555 | 3.8075867 | -0.6710824 |
| 31 | H | H7 | -3.1330517 | 4.7362777 | -0.3381130 |
| 32 | H | H10 | -3.3401538 | 3.2284802 | -1.3129356 |

## piperonyl [3,3]-TS - actual product (TS6a)

Spartan'10: G = -1040.98573, iv = i345
Gaussian'09: B3LYP/6-31G* G = -1040.990095, iv = i346
Gaussian'09: M06-2X/6-31G* G = -1040.586048, iv = i403

Cartesian Coordinates (Angstroms)

| | | Atom | X | Y | Z |
|---|---|---|---|---|---|
| 1 | C | C1 | 0.0299913 | -0.9765097 | 0.5447855 |
| 2 | H | H3 | -0.0919916 | -2.0411956 | 0.4137142 |
| 3 | C | C2 | 1.3614143 | -0.5751782 | 1.0108108 |
| 4 | C | C5 | 1.3636871 | -0.5111860 | -2.0543037 |
| 5 | H | H7 | 1.4537369 | 0.4091006 | -2.6287065 |
| 6 | C | C6 | 0.0517463 | -0.8483121 | -1.5978248 |
| 7 | H | H6 | -0.2395078 | -1.8891689 | -1.6318160 |
| 8 | C | C7 | 1.4922535 | 0.5993397 | 1.8477715 |
| 9 | H | H4 | 2.4728296 | 0.9436762 | 2.1581912 |
| 10 | C | C8 | 0.3628135 | 1.2572276 | 2.2002409 |
| 11 | C | C9 | -0.9392342 | 0.8221317 | 1.8038003 |
| 12 | C | C10 | -1.1381880 | -0.2826499 | 1.0474906 |
| 13 | H | H12 | -2.1309181 | -0.6337514 | 0.7940555 |
| 14 | C | C3 | 2.5066100 | -1.1683158 | -1.7020673 |
| 15 | C | C4 | 2.5020439 | -1.2262617 | 0.5869547 |
| 16 | C | C11 | 2.5903161 | -2.1494753 | -0.5812988 |
| 17 | H | H2 | 3.4711995 | -0.8224478 | -2.0616157 |
| 18 | H | H10 | 3.4706018 | -0.8816310 | 0.9385686 |
| 19 | C | C12 | -1.0242132 | 0.1017440 | -1.9670565 |
| 20 | O | O1 | -0.8665404 | 1.2683970 | -2.2727871 |
| 21 | O | O2 | -2.2473210 | -0.4856953 | -1.8957493 |
| 22 | C | C13 | -3.3538526 | 0.3761447 | -2.2020706 |
| 23 | H | H1 | -3.2688036 | 0.7645485 | -3.2204452 |
| 24 | H | H5 | -3.3906132 | 1.2179722 | -1.5049668 |
| 25 | H | H8 | -4.2456265 | -0.2439745 | -2.1027276 |
| 26 | F | F1 | 1.6195434 | -3.1315895 | -0.6096091 |
| 27 | F | F2 | 3.8003624 | -2.7946598 | -0.5875743 |
| 28 | O | O3 | -1.8708063 | 1.6633345 | 2.3447234 |
| 29 | O | O4 | 0.2406542 | 2.3885294 | 2.9577602 |
| 30 | C | C14 | -1.1582213 | 2.6450504 | 3.1015459 |
| 31 | H | H9 | -1.3866334 | 3.6423907 | 2.7120647 |
| 32 | H | H13 | -1.4373328 | 2.5624154 | 4.1581412 |

## piperonyl [3,3] -TS - alternative regioisomer (TS6b)

Spartan'10: G = -1040.97722
iv = i380

Cartesian Coordinates (Angstroms)

| | | Atom | X | Y | Z |
|---|---|---|---|---|---|
| 1 | C | C1 | 0.2385885 | -0.7298865 | 0.7224743 |
| 2 | H | H3 | 0.0276832 | -1.7842292 | 0.6037600 |
| 3 | C | C2 | 1.5951995 | -0.4027051 | 1.2177965 |
| 4 | C | C5 | 1.4721330 | -0.1108453 | -1.8181119 |
| 5 | H | H7 | 1.5045379 | 0.8562664 | -2.3158420 |
| 6 | C | C6 | 0.1974816 | -0.5696510 | -1.3534490 |
| 7 | H | H6 | 0.0086455 | -1.6282209 | -1.4642294 |
| 8 | C | C7 | 1.7716717 | 0.6741291 | 2.1666199 |
| 9 | H | H4 | 2.7859233 | 0.9105927 | 2.4746075 |
| 10 | C | C8 | 0.7251018 | 1.4116844 | 2.6504553 |
| 11 | H | H9 | 0.8673343 | 2.2246265 | 3.3541622 |
| 12 | C | C9 | -0.5691163 | 1.0800378 | 2.1831251 |
| 13 | C | C10 | -0.7933501 | 0.0582918 | 1.3159113 |
| 14 | C | C3 | 2.6474765 | -0.7549716 | -1.5599684 |
| 15 | C | C4 | 2.7220531 | -1.0155180 | 0.7105452 |
| 16 | C | C11 | 2.7866601 | -1.8315684 | -0.5349616 |
| 17 | H | H2 | 3.5910266 | -0.3508489 | -1.9139038 |
| 18 | H | H10 | 3.6972904 | -0.7160370 | 1.0845140 |
| 19 | C | C12 | -0.9732522 | 0.2802463 | -1.6932325 |
| 20 | O | O1 | -0.9832406 | 1.4969908 | -1.6931776 |
| 21 | O | O2 | -2.0514151 | -0.4793664 | -1.9984359 |
| 22 | C | C13 | -3.2456076 | 0.2469918 | -2.3140927 |
| 23 | H | H1 | -3.0876808 | 0.8904759 | -3.1847093 |
| 24 | H | H5 | -3.5544741 | 0.8660227 | -1.4670107 |
| 25 | H | H8 | -4.0017662 | -0.5095511 | -2.5281664 |
| 26 | F | F1 | 1.8295525 | -2.8240916 | -0.6069984 |
| 27 | F | F2 | 4.0040881 | -2.4515217 | -0.6373763 |
| 28 | O | O3 | -2.1500850 | -0.1133524 | 1.1274261 |
| 29 | O | O4 | -1.7631087 | 1.6229029 | 2.6040112 |
| 30 | C | C14 | -2.7287256 | 1.0660126 | 1.7046521 |
| 31 | H | H12 | -2.9406554 | 1.7919193 | 0.9059492 |
| 32 | H | H14 | -3.6299698 | 0.7951741 | 2.2576566 |

**Isodesmic Compounds (Energies can be found in Table S16-S19)**

Ethane

Cartesian Coordinates (Angstroms)

| | | Atom | X | Y | Z |
|---|---|---|---|---|---|
| 1 | C | C1 | 0.0000001 | 0.0000000 | 0.7637754 |
| 2 | H | H2 | 1.0121266 | -0.0000001 | 1.1565569 |
| 3 | H | H3 | -0.5060633 | -0.8765272 | 1.1565570 |
| 4 | H | H4 | -0.5060631 | 0.8765274 | 1.1565570 |
| 5 | C | C2 | -0.0000001 | 0.0000000 | -0.7637754 |
| 6 | H | H1 | 0.5060631 | -0.8765274 | -1.1565570 |
| 7 | H | H5 | 0.5060633 | 0.8765272 | -1.1565570 |
| 8 | H | H6 | -1.0121266 | 0.0000001 | -1.1565569 |

Ethene

Cartesian Coordinates (Angstroms)

| | | Atom | X | Y | Z |
|---|---|---|---|---|---|
| 1 | C | C1 | 0.0000000 | 0.0000000 | 0.6585346 |
| 2 | H | H2 | 0.0000000 | 0.9144843 | 1.2256492 |
| 3 | H | H3 | 0.0000000 | -0.9144843 | 1.2256492 |
| 4 | C | C2 | 0.0000000 | 0.0000000 | -0.6585346 |
| 5 | H | H1 | 0.0000000 | -0.9144843 | -1.2256492 |
| 6 | H | H4 | 0.0000000 | 0.9144843 | -1.2256492 |

Benzene

Cartesian Coordinates (Angstroms)

| | | Atom | X | Y | Z |
|---|---|---|---|---|---|
| 1 | H | H1 | 0.0000000 | 0.0000000 | -2.4836029 |
| 2 | C | C1 | 0.0000000 | -0.0000001 | -1.3965320 |
| 3 | C | C4 | 0.0000000 | -0.0000001 | 1.3965320 |
| 4 | C | C2 | 0.0000000 | -1.2095560 | -0.6982739 |
| 5 | C | C6 | 0.0000000 | 1.2095560 | -0.6982740 |
| 6 | C | C5 | 0.0000000 | 1.2095560 | 0.6982740 |
| 7 | C | C3 | 0.0000000 | -1.2095560 | 0.6982739 |
| 8 | H | H2 | 0.0000000 | -2.1508686 | -1.2419479 |
| 9 | H | H6 | 0.0000000 | 2.1508686 | -1.2419480 |
| 10 | H | H5 | 0.0000000 | 2.1508686 | 1.2419480 |
| 11 | H | H3 | 0.0000000 | -2.1508686 | 1.2419480 |
| 12 | H | H4 | 0.0000000 | -0.0000001 | 2.4836029 |

Benzene - reduced

Cartesian Coordinates (Angstroms)

| Atom | | | X | Y | Z |
|---|---|---|---|---|---|
| 1 | H | H1 | 1.0881541 | -0.5985062 | -2.1761678 |
| 2 | C | C1 | 0.6434959 | -0.2310204 | -1.2671432 |
| 3 | C | C3 | 0.4539005 | 1.3985299 | 0.5292146 |
| 4 | C | C5 | -0.6486740 | -0.7877853 | 0.8245360 |
| 5 | C | C4 | -0.3680277 | 0.6386557 | 1.2368443 |
| 6 | C | C6 | -0.5413287 | -0.9731704 | -0.6936467 |
| 7 | C | C2 | 1.0859599 | 0.8801419 | -0.6985849 |
| 8 | H | H10 | 0.0680039 | -1.4397351 | 1.3250990 |
| 9 | H | H7 | -0.8121612 | 1.0069181 | 2.1458111 |
| 10 | H | H11 | -1.4473510 | -0.6014106 | -1.1733836 |
| 11 | H | H3 | 1.9051309 | 1.4317046 | -1.1264837 |
| 12 | H | H5 | 0.6856329 | 2.4020851 | 0.8415236 |
| 13 | H | H9 | -1.6321903 | -1.0961221 | 1.1650105 |
| 14 | H | H12 | -0.4805450 | -2.0302851 | -0.9326293 |

Furan

Cartesian Coordinates (Angstroms)

| Atom | | | X | Y | Z |
|---|---|---|---|---|---|
| 1 | O | O1 | 0.0506086 | -0.4485978 | -1.2845127 |
| 2 | C | C2 | 1.0978809 | -0.0928593 | -0.5216394 |
| 3 | C | C3 | 0.6907284 | 0.3010616 | 0.6916180 |
| 4 | C | C4 | -0.7447007 | 0.1773514 | 0.6782675 |
| 5 | C | C5 | -1.0559874 | -0.2784870 | -0.5416720 |
| 6 | H | H3 | 2.0606797 | -0.1735618 | -0.9770217 |
| 7 | H | H5 | 1.3099502 | 0.6364801 | 1.4975195 |
| 8 | H | H7 | -1.4269491 | 0.4006048 | 1.4720643 |
| 9 | H | H9 | -1.9822107 | -0.5219919 | -1.0146236 |

Furan - reduced

Cartesian Coordinates (Angstroms)

| Atom | | | X | Y | Z |
|---|---|---|---|---|---|
| 1 | O | O1 | 0.2823937 | -0.4458870 | -1.3684354 |
| 2 | C | C2 | 1.2687481 | 0.0829888 | -0.6101449 |
| 3 | C | C3 | 0.9192139 | 0.4245706 | 0.6106088 |
| 4 | C | C4 | -0.5396163 | 0.0842859 | 0.8071793 |
| 5 | C | C5 | -0.9272401 | -0.3714167 | -0.6179478 |
| 6 | H | H3 | 2.2210539 | 0.1535510 | -1.0962173 |
| 7 | H | H5 | 1.5638401 | 0.8469238 | 1.3544034 |
| 8 | H | H7 | -1.1345112 | 0.9320998 | 1.1315137 |
| 9 | H | H8 | -0.6786505 | -0.7048947 | 1.5411980 |
| 10 | H | H9 | -1.5783534 | 0.3422369 | -1.1071110 |
| 11 | H | H10 | -1.3968781 | -1.3444585 | -0.6450469 |

thiophene

Cartesian Coordinates (Angstroms)

| Atom | | | X | Y | Z |
|---|---|---|---|---|---|
| 1 | S | S1 | 0.1167444 | -0.7253040 | -1.5997346 |
| 2 | C | C2 | 1.2671448 | -0.1588718 | -0.4449549 |
| 3 | C | C3 | 0.6707684 | 0.3240820 | 0.6597454 |
| 4 | C | C4 | -0.7621015 | 0.2433484 | 0.5917821 |
| 5 | C | C5 | -1.1936835 | -0.2975247 | -0.5616759 |
| 6 | H | H3 | 2.3162648 | -0.2167911 | -0.6518827 |
| 7 | H | H5 | 1.2070897 | 0.7252313 | 1.4987648 |
| 8 | H | H7 | -1.4167593 | 0.5773933 | 1.3743115 |
| 9 | H | H9 | -2.2054677 | -0.4715633 | -0.8663556 |

thiophene -reduced

Cartesian Coordinates (Angstroms)

| Atom | | | X | Y | Z |
|---|---|---|---|---|---|
| 1 | S | S1 | 0.2381818 | -0.1586916 | -1.8476764 |
| 2 | C | C2 | 1.4343914 | 0.1554841 | -0.5805729 |
| 3 | C | C3 | 0.9357534 | 0.2978303 | 0.6304529 |
| 4 | C | C4 | -0.5698240 | 0.2147878 | 0.7144893 |
| 5 | C | C5 | -1.0420275 | -0.4756587 | -0.5772318 |
| 6 | H | H3 | 2.4697948 | 0.2300008 | -0.8513928 |
| 7 | H | H5 | 1.5396600 | 0.5097448 | 1.4940703 |
| 8 | H | H7 | -0.9878734 | 1.2166165 | 0.7920098 |
| 9 | H | H8 | -0.9060149 | -0.3384097 | 1.5853369 |
| 10 | H | H9 | -1.9954398 | -0.1062750 | -0.9266186 |
| 11 | H | H10 | -1.1166018 | -1.5454293 | -0.4328669 |

piperonyl

Cartesian Coordinates (Angstroms)

| Atom | | | X | Y | Z |
|---|---|---|---|---|---|
| 1 | H | H1 | -2.4890973 | 0.0000000 | 0.7605630 |
| 2 | C | C1 | -1.4155844 | 0.0000000 | 0.7735268 |
| 3 | C | C4 | 1.4155844 | 0.0000000 | 0.7735268 |
| 4 | C | C2 | -0.6905232 | 0.0000000 | -0.3841316 |
| 5 | C | C6 | -0.6899151 | 0.0000000 | 1.9718652 |
| 6 | C | C5 | 0.6899151 | 0.0000000 | 1.9718652 |
| 7 | C | C3 | 0.6905232 | 0.0000000 | -0.3841316 |
| 8 | H | H6 | -1.2223019 | 0.0000000 | 2.9054279 |
| 9 | H | H5 | 1.2223019 | 0.0000000 | 2.9054279 |
| 10 | H | H4 | 2.4890973 | 0.0000000 | 0.7605630 |
| 11 | O | O1 | -1.1369414 | 0.0000000 | -1.6665113 |
| 12 | O | O2 | 1.1369414 | 0.0000000 | -1.6665113 |
| 13 | C | C7 | 0.0000000 | 0.0000000 | -2.4974534 |
| 14 | H | H3 | 0.0000000 | -0.8891458 | -3.1120134 |
| 15 | H | H8 | 0.0000000 | 0.8891458 | -3.1120134 |

piperonyl reduced

Cartesian Coordinates (Angstroms)

| | Atom | | X | Y | Z |
|---|---|---|---|---|---|
| 1 | H | H1 | -2.5329374 | 0.0000000 | 0.4013201 |
| 2 | C | C1 | -1.4590576 | 0.0000000 | 0.4432548 |
| 3 | C | C3 | 0.7815602 | 0.0000000 | 1.7990097 |
| 4 | C | C5 | 0.7299606 | 0.0000000 | -0.6478620 |
| 5 | C | C4 | 1.4590576 | 0.0000000 | 0.4432548 |
| 6 | C | C6 | -0.7299606 | 0.0000000 | -0.6478620 |
| 7 | C | C2 | -0.7815602 | 0.0000000 | 1.7990097 |
| 8 | H | H6 | 1.1341868 | 0.8637486 | 2.3556278 |
| 9 | H | H7 | 2.5329374 | 0.0000000 | 0.4013201 |
| 10 | H | H3 | -1.1341868 | -0.8637486 | 2.3556278 |
| 11 | H | H5 | 1.1341868 | -0.8637486 | 2.3556278 |
| 12 | H | H4 | -1.1341868 | 0.8637486 | 2.3556278 |
| 13 | O | O1 | 1.1332016 | 0.0000000 | -1.9415502 |
| 14 | O | O2 | -1.1332016 | 0.0000000 | -1.9415502 |
| 15 | C | C7 | 0.0000000 | 0.0000000 | -2.7666606 |
| 16 | H | H12 | 0.0000000 | 0.8882923 | -3.3820978 |
| 17 | H | H13 | 0.0000000 | -0.8882923 | -3.3820978 |

pyrrole

Cartesian Coordinates (Angstroms)

| | Atom | | X | Y | Z |
|---|---|---|---|---|---|
| 1 | C | C1 | -0.4449852 | 1.0284683 | 0.3679170 |
| 2 | H | H2 | -0.8752591 | 1.9155106 | 0.8102523 |
| 3 | C | C2 | 0.0893702 | 0.8040222 | -0.8825847 |
| 4 | H | H4 | 0.1681822 | 1.5338806 | -1.6771450 |
| 5 | N | N1 | -0.3576710 | -0.1471977 | 1.0761889 |
| 6 | H | H3 | -0.6730776 | -0.2771132 | 2.0246473 |
| 7 | C | C3 | 0.2239280 | -1.1194084 | 0.2961859 |
| 8 | H | H6 | 0.3804864 | -2.1188340 | 0.6759902 |
| 9 | C | C4 | 0.5125838 | -0.5560907 | -0.9280150 |
| 10 | H | H5 | 0.9764424 | -1.0632377 | -1.7634369 |

pyridine

Cartesian Coordinates (Angstroms)

| | Atom | | X | Y | Z |
|---|---|---|---|---|---|
| 1 | H | H1 | 0.0000000 | -0.0000007 | -2.2824770 |
| 2 | C | C1 | 0.0000000 | -0.0000003 | -1.1955420 |
| 3 | N | N1 | 0.0000000 | 0.0000004 | 1.6106501 |
| 4 | C | C2 | 0.0000000 | -1.1985966 | -0.4833139 |
| 5 | C | C6 | 0.0000000 | 1.1985963 | -0.4833146 |
| 6 | C | C5 | 0.0000000 | 1.1422376 | 0.9115526 |
| 7 | C | C3 | 0.0000000 | -1.1422371 | 0.9115532 |
| 8 | H | H2 | 0.0000000 | -2.1577589 | -0.9929579 |
| 9 | H | H6 | 0.0000000 | 2.1577583 | -0.9929590 |

| | | | | | |
|---|---|---|---|---|---|
| 10 | H | H5 | 0.0000000 | 2.0597701 | 1.4984036 |
| 11 | H | H3 | 0.0000000 | -2.0597693 | 1.4984048 |

oxazole

Cartesian Coordinates (Angstroms)

| | Atom | | X | Y | Z |
|---|---|---|---|---|---|
| 1 | N | N1 | -0.2423485 | -1.0732650 | -0.6256410 |
| 2 | C | C2 | 0.9713989 | -0.7097296 | -0.3615412 |
| 3 | C | C5 | -1.0233183 | -0.0579885 | -0.0804509 |
| 4 | H | H4 | 1.8940387 | -1.2145675 | -0.6086099 |
| 5 | H | H10 | -2.1019922 | -0.0783051 | -0.1413901 |
| 6 | O | O1 | 1.0812771 | 0.4623778 | 0.3144037 |
| 7 | C | C1 | -0.2161812 | 0.8710022 | 0.4885116 |
| 8 | H | H2 | -0.3628746 | 1.8004756 | 1.0147178 |

isoxazole

Cartesian Coordinates (Angstroms)

| | Atom | | X | Y | Z |
|---|---|---|---|---|---|
| 1 | N | N1 | -1.3579729 | -0.3166155 | 0.6680020 |
| 2 | O | O1 | -0.9688970 | -1.0478468 | -0.4596005 |
| 3 | C | C1 | -0.3749555 | 0.5260090 | 0.8839956 |
| 4 | H | H3 | -0.4490582 | 1.2097088 | 1.7205650 |
| 5 | C | C2 | 0.2337272 | -0.6094549 | -0.8719390 |
| 6 | H | H4 | 0.6405663 | -1.1029212 | -1.7432137 |
| 7 | C | C3 | 0.6770494 | 0.3923385 | -0.0660168 |
| 8 | H | H5 | 1.5995407 | 0.9487822 | -0.1317926 |

**Remaining Species for Computational Prediction**

## Ph cis SM (1b)

Spartan'10: G = -852.505870, iv = none
Gaussian'09: B3LYP/6-31G*, G = -852.511692, iv = none
Gaussian'09: M06-2X/6-31G*, G = -852.17197, iv = none

Cartesian Coordinates (Angstroms)

| | | Atom | X | Y | Z |
|---|---|---|---|---|---|
| 1 | H | H1 | -0.2566781 | 2.1773309 | -0.5418559 |
| 2 | C | C1 | 0.7326203 | 2.1016848 | -0.0979338 |
| 3 | C | C4 | 3.2705797 | 1.8884782 | 1.0330952 |
| 4 | C | C2 | 0.8683703 | 1.5838354 | 1.1975351 |
| 5 | C | C6 | 1.8530942 | 2.5100396 | -0.8195723 |
| 6 | C | C5 | 3.1260344 | 2.4054202 | -0.2547015 |
| 7 | C | C3 | 2.1488322 | 1.4797692 | 1.7561690 |
| 8 | H | H6 | 1.7326412 | 2.9064500 | -1.8241942 |
| 9 | H | H5 | 3.9999792 | 2.7221862 | -0.8173662 |
| 10 | H | H3 | 2.2673570 | 1.0735024 | 2.7558601 |
| 11 | H | H4 | 4.2579709 | 1.8001634 | 1.4782035 |
| 12 | C | C7 | -0.3508853 | 1.2086657 | 1.9816477 |
| 13 | C | C8 | -0.4570777 | -0.0224345 | 2.7885630 |
| 14 | C | C9 | -1.2947057 | 0.0093460 | 1.5504614 |
| 15 | H | H9 | -2.3531250 | 0.1988810 | 1.7111959 |
| 16 | F | F1 | -1.0722914 | 0.0238570 | 3.9930383 |
| 17 | F | F2 | 0.5874286 | -0.8816675 | 2.8417710 |
| 18 | C | C10 | -0.9504897 | -0.8346112 | 0.3975477 |
| 19 | H | H8 | 0.0925116 | -1.1108468 | 0.2613425 |
| 20 | C | C11 | -1.8431113 | -1.2883794 | -0.4950921 |
| 21 | H | H10 | -2.9028398 | -1.0588657 | -0.4270965 |
| 22 | C | C12 | -1.4068747 | -2.1361501 | -1.6249384 |
| 23 | O | O1 | -0.2668900 | -2.4927457 | -1.8526699 |
| 24 | O | O2 | -2.4647872 | -2.4769771 | -2.4025691 |
| 25 | C | C13 | -2.1481186 | -3.3039233 | -3.5313814 |
| 26 | H | H7 | -1.6796913 | -4.2367585 | -3.2058806 |
| 27 | H | H11 | -1.4645918 | -2.7848208 | -4.2092518 |
| 28 | H | H12 | -3.0995277 | -3.5034504 | -4.0257970 |
| 29 | H | H13 | -0.9257341 | 2.0420210 | 2.3838702 |

## Ph [3,3] – TS

Spartan'10: G = -852.464912, iv = i379
Gaussian'09: B3LYP/6-31G*, G = -852.467339, iv = i380
Gaussian'09: M06-2X/6-31G*, G = -852.124968, iv = i450

Cartesian Coordinates (Angstroms)

| | | Atom | X | Y | Z |
|---|---|---|---|---|---|
| 1 | C | C1 | -0.1088780 | -0.6432231 | 0.8863613 |
| 2 | H | H3 | -0.2589437 | -1.7075866 | 0.7739837 |

```
  3 C  C2          1.2220184    -0.2597465     1.3621125
  4 C  C5          1.2048982    -0.1785670    -1.6827749
  5 H  H7          1.2844671     0.7425621    -2.2565575
  6 C  C6         -0.1033175    -0.5238935    -1.2142480
  7 H  H6         -0.3862752    -1.5665343    -1.2643495
  8 C  C7          1.3310470     0.9008674     2.2111675
  9 H  H4          2.3241582     1.2134430     2.5246042
 10 C  C8          0.2299951     1.6063232     2.5951180
 11 H  H9          0.3384265     2.4867706     3.2225007
 12 C  C9         -1.0815962     1.1903990     2.1893254
 13 H  H11        -1.9465638     1.7409931     2.5492119
 14 C  C10        -1.2449905     0.0914736     1.4014086
 15 H  H12        -2.2377005    -0.2546742     1.1316479
 16 C  C3          2.3539553    -0.8303083    -1.3422934
 17 C  C4          2.3677107    -0.9013675     0.9378646
 18 C  C11         2.4527866    -1.8220421    -0.2319275
 19 H  H2          3.3133015    -0.4772245    -1.7088463
 20 H  H10         3.3345269    -0.5601096     1.2979552
 21 C  C12        -1.1878915     0.4137056    -1.6091269
 22 O  O1         -1.0362810     1.5742628    -1.9352174
 23 O  O2         -2.4035180    -0.1866668    -1.5458552
 24 C  C13        -3.5160485     0.6557767    -1.8841186
 25 H  H1         -3.4156713     1.0343754    -2.9050867
 26 H  H5         -3.5773554     1.5046847    -1.1970147
 27 H  H8         -4.4003348     0.0232509    -1.7948402
 28 F  F1          1.4839922    -2.8045753    -0.2568552
 29 F  F2          3.6640819    -2.4623688    -0.2541494
```

## furan cis SM (cis-9b)

```
Spartan'10: G = -850.312391, iv = none
Gaussian'09: B3LYP/6-31G*, G = -850.318011, iv = none
Gaussian'09: M06-2X/6-31G*, G = -849.99051, iv = none

Cartesian Coordinates (Angstroms)
       Atom             X             Y             Z
    ---------    ------------- ------------- -------------

  1 C  C1          2.4512641     1.3354068     0.2415934
  2 C  C2          1.3356127     1.7987431     1.0906077
  3 C  C3          1.6498298     0.2431782     0.8642397
  4 H  H6          2.1119688    -0.2326259     1.7254883
  5 C  C10         0.7489355    -0.5847449     0.0548152
  6 H  H11         0.2835022    -0.1279728    -0.8156985
  7 C  C11         0.4611098    -1.8668181     0.3233625
  8 H  H12         0.8845279    -2.3941822     1.1733777
  9 C  C12        -0.4570930    -2.6247006    -0.5533210
 10 O  O1         -1.0187351    -2.1938951    -1.5421198
 11 O  O2         -0.5986966    -3.8979997    -0.1092677
 12 C  C13        -1.4696957    -4.7237084    -0.8953584
 13 H  H5         -2.4872360    -4.3224924    -0.8919402
 14 H  H13        -1.4449165    -5.7075124    -0.4253723
 15 H  H14        -1.1173309    -4.7811726    -1.9288566
 16 F  F1          2.3876458     1.5065515    -1.1002996
 17 F  F2          3.7188171     1.5340913     0.6670521
```

```
 18 C   C4            0.1324177     2.4429016     0.5472554
 19 C   C5           -0.2167458     2.9711565    -0.6638453
 20 C   C6           -1.5600181     3.4501561    -0.5266274
 21 C   C7           -1.9288519     3.1814347     0.7548266
 22 H   H1            0.4101903     3.0158134    -1.5416556
 23 H   H8           -2.1620993     3.9312406    -1.2845736
 24 H   H9           -2.8285917     3.3542179     1.3254461
 25 O   O3           -0.9144789     2.5658491     1.4256099
 26 H   H2            1.6286680     2.1270843     2.0852611
```

### Furan 3,3 TS

```
Spartan'10: G = -850.287883, iv = none
Gaussian'09: B3LYP/6-31G*, G = -850.290209, iv = i284
Gaussian'09: M06-2X/6-31G*, G = -849.957473, iv = i332

                      Cartesian Coordinates (Angstroms)
       Atom             X             Y             Z
    ---------    ------------- ------------- -------------

  1 C   C1           -0.0686022    -0.6391635     1.1400999
  2 H   H3           -0.3649563    -1.6315030     0.8437269
  3 C   C2            1.2811173    -0.3378544     1.5213062
  4 C   C5            1.1576922     0.3153033    -1.4749842
  5 H   H7            1.1552839     1.3253536    -1.8803005
  6 C   C6           -0.0969354    -0.2147826    -1.0919058
  7 H   H6           -0.2642143    -1.2768469    -1.1819185
  8 C   C3            2.3686760    -0.2873355    -1.2439468
  9 C   C4            2.4762418    -0.7290840     0.9905851
 10 C   C11           2.5722832    -1.4377316    -0.3188344
 11 H   H2            3.2891460     0.2053055    -1.5429394
 12 H   H10           3.4002144    -0.3149572     1.3784504
 13 C   C12          -1.2884862     0.6414637    -1.2503270
 14 O   O1           -1.2818295     1.8382432    -1.4710164
 15 O   O2           -2.4318212    -0.0765206    -1.0850122
 16 C   C13          -3.6464005     0.6748159    -1.2190799
 17 H   H1           -3.7181560     1.1183212    -2.2162060
 18 H   H5           -3.6912395     1.4756892    -0.4752773
 19 H   H8           -4.4538401    -0.0415801    -1.0609347
 20 F   F1            1.6519208    -2.4545275    -0.4594124
 21 F   F2            3.8126397    -1.9929923    -0.4799666
 22 O   O3            1.2640133     0.6728156     2.4716370
 23 C   C8           -0.0472362     0.9682551     2.7083964
 24 H   H12          -0.2145705     1.7366318     3.4495822
 25 C   C7           -0.8906130     0.2167166     1.9638239
 26 H   H11          -1.9703284     0.2459645     1.9844542
```

## cis 2-thiophene (cis-9d)

Spartan;10: G = -1173.29413, iv = none
Gaussian'09: B3LYP/6-31G*, G = -1173.266174, iv = none
Gaussian'09: M06-2X/6-31G*, G = -1172.963766, iv = none

Cartesian Coordinates (Angstroms)

| Atom | | | X | Y | Z |
|---|---|---|---|---|---|
| 1 | C | C1 | 2.5255454 | 1.2442007 | 0.3046356 |
| 2 | C | C2 | 1.4092065 | 1.7127642 | 1.1546039 |
| 3 | C | C3 | 1.7042303 | 0.1577513 | 0.9110483 |
| 4 | H | H6 | 2.1492153 | -0.3384332 | 1.7698751 |
| 5 | C | C10 | 0.7967803 | -0.6445570 | 0.0819703 |
| 6 | H | H11 | 0.4006088 | -0.1915052 | -0.8237508 |
| 7 | C | C11 | 0.4227832 | -1.8986507 | 0.3752741 |
| 8 | H | H12 | 0.7706948 | -2.4181631 | 1.2634495 |
| 9 | C | C12 | -0.4986782 | -2.6301292 | -0.5203969 |
| 10 | O | O1 | -0.9591428 | -2.2123635 | -1.5654584 |
| 11 | O | O2 | -0.7784609 | -3.8584840 | -0.0190998 |
| 12 | C | C13 | -1.6709491 | -4.6513349 | -0.8149407 |
| 13 | H | H5 | -2.6213165 | -4.1310089 | -0.9613722 |
| 14 | H | H13 | -1.8205664 | -5.5759300 | -0.2562606 |
| 15 | H | H14 | -1.2297810 | -4.8612060 | -1.7934949 |
| 16 | F | F1 | 2.4811110 | 1.4238483 | -1.0366860 |
| 17 | F | F2 | 3.7919029 | 1.4207600 | 0.7448429 |
| 18 | C | C4 | 0.1974389 | 2.3794114 | 0.6317526 |
| 19 | C | C5 | -0.0206193 | 2.9993977 | -0.5735478 |
| 20 | C | C6 | -1.3376590 | 3.5345359 | -0.7029058 |
| 21 | C | C7 | -2.1103877 | 3.3194067 | 0.4031767 |
| 22 | H | H1 | 0.7391033 | 3.0735412 | -1.3415374 |
| 23 | H | H8 | -1.6890187 | 4.0596242 | -1.5843589 |
| 24 | H | H9 | -3.1355766 | 3.6182392 | 0.5758638 |
| 25 | S | S1 | -1.2434068 | 2.4495722 | 1.6278462 |
| 26 | H | H2 | 1.7269422 | 2.0187127 | 2.1494713 |

## cis 2-thiophene TS

Spartan'10: G = -1173.26802, iv = i300
Gaussian'09: B3LYP/6-31G*, G = -1173.27068, iv = i292
Gaussian'09: M06-2X/6-31G*, G = -1172.930053, iv = i355

Cartesian Coordinates (Angstroms)

| Atom | | | X | Y | Z |
|---|---|---|---|---|---|
| 1 | C | C1 | 0.0363919 | -0.6432743 | 1.1516885 |
| 2 | H | H3 | -0.1761859 | -1.6591940 | 0.8567293 |
| 3 | C | C2 | 1.3874465 | -0.2999837 | 1.5137127 |
| 4 | C | C5 | 1.1733536 | 0.2907682 | -1.4934262 |
| 5 | H | H7 | 1.1752585 | 1.2953169 | -1.9124989 |
| 6 | C | C6 | -0.0790655 | -0.2116921 | -1.0622572 |
| 7 | H | H6 | -0.2724851 | -1.2692479 | -1.1547817 |

```
  8 C   C3            2.3786214     -0.3339823     -1.3002843
  9 C   C4            2.5468282     -0.7424851      0.9329581
 10 C   C11           2.5890941     -1.4767566     -0.3677304
 11 H   H2            3.2976792      0.1349339     -1.6388706
 12 H   H10           3.5118299     -0.3750983      1.2683835
 13 C   C12          -1.2579048      0.6675101     -1.2124209
 14 O   O1           -1.2307474      1.8606375     -1.4478607
 15 O   O2           -2.4125233     -0.0273033     -1.0305440
 16 C   C13          -3.6139604      0.7468960     -1.1609657
 17 H   H1           -3.6960384      1.1636488     -2.1688630
 18 H   H5           -3.6249968      1.5685421     -0.4391052
 19 H   H8           -4.4331588      0.0529329     -0.9673123
 20 F   F1            1.6536572     -2.4833370     -0.4711193
 21 F   F2            3.8170567     -2.0516754     -0.5559142
 22 S   S2            1.3849854      1.0245650      2.7059169
 23 C   C8           -0.3631965      1.0161350      2.7583118
 24 H   H12          -0.8724305      1.6934226      3.4324691
 25 C   C7           -0.9252665      0.1083666      1.9231062
 26 H   H11          -1.9942427     -0.0496457      1.8406781
```

## alpha Me cis SM (*cis*-10i)

```
Spartan'10: G = -891.794682, iv = none
Gaussian'09: B3LYP/6-31G*, G = -891.802283, iv = none
Gaussian'09: M06-2X/6-31G*, G = -891.443545, iv = none

Cartesian Coordinates (Angstroms)
       Atom              X             Y             Z
    ---------    ------------- ------------- -------------

  1 H   H1            1.0605970      2.2101135      0.9795402
  2 C   C1            1.4318218      1.3411226      1.5172655
  3 C   C4            2.3865025     -0.8862409      2.8887981
  4 C   C2            0.5185848      0.4461580      2.0944014
  5 C   C6            2.8045038      1.1268614      1.6258776
  6 C   C5            3.2865377      0.0103681      2.3130865
  7 C   C3            1.0111170     -0.6707822      2.7826651
  8 H   H6            3.4977073      1.8308283      1.1731001
  9 H   H5            4.3566476     -0.1582550      2.3986995
 10 H   H3            0.3185399     -1.3740829      3.2324462
 11 H   H4            2.7523580     -1.7575112      3.4255959
 12 C   C7           -0.9468513      0.7418941      2.0124806
 13 C   C8           -2.0024763     -0.2830311      1.8928866
 14 C   C9           -1.7848029      0.5524054      0.6736209
 15 H   H9           -2.5331798      1.3236271      0.5234112
 16 F   F1           -3.1525956     -0.1268753      2.5902310
 17 F   F2           -1.6724700     -1.5967678      1.8516777
 18 C   C10          -1.1539931     -0.0304295     -0.5213977
 19 H   H8           -0.4580820     -0.8435968     -0.3434813
 20 C   C11          -1.3639456      0.3454763     -1.7988830
 21 C   C12          -0.6574363     -0.3600092     -2.9068867
 22 O   O1           -0.8167572     -0.0880527     -4.0826858
 23 O   O2            0.1855185     -1.3350438     -2.4887181
 24 C   C13           0.8788792     -2.0320139     -3.5329248
 25 H   H7            1.5263014     -2.7480706     -3.0254650
```

| | | | | | |
|---|---|---|---|---|---|
| 26 | H | H11 | 1.4701051 | -1.3364892 | -4.1346099 |
| 27 | H | H12 | 0.1715048 | -2.5502677 | -4.1865157 |
| 28 | H | H13 | -1.2694853 | 1.6294198 | 2.5548831 |
| 29 | C | C14 | -2.2831145 | 1.4423181 | -2.2707235 |
| 30 | H | H2 | -1.7367054 | 2.1587340 | -2.8934231 |
| 31 | H | H10 | -2.7448759 | 1.9845092 | -1.4427544 |
| 32 | H | H14 | -3.0804549 | 1.0336839 | -2.9021983 |

## alpha Me [3,3] - TS

Spartan'10: G = -891.746085, iv = i364
Gaussian'09: B3LYP/6-31G*, G = -891.748315, iv = i353
Gaussian'09: M06-2X/6-31G*, G = -891.387733, iv = i436

Cartesian Coordinates (Angstroms)

| Atom | | | X | Y | Z |
|---|---|---|---|---|---|
| 1 | C | C1 | -0.0100016 | -0.5463738 | 1.0292330 |
| 2 | H | H3 | -0.0273706 | -1.6176378 | 0.8919358 |
| 3 | C | C2 | 1.2581965 | 0.0056784 | 1.4941897 |
| 4 | C | C5 | 1.2777056 | -0.1356812 | -1.5540668 |
| 5 | H | H7 | 1.3712153 | 0.7638974 | -2.1600692 |
| 6 | C | C6 | -0.0625493 | -0.4626455 | -1.1428007 |
| 7 | C | C7 | 1.2247206 | 1.2095288 | 2.2856223 |
| 8 | H | H4 | 2.1708031 | 1.6498380 | 2.5907106 |
| 9 | C | C8 | 0.0431027 | 1.7927875 | 2.6374625 |
| 10 | H | H9 | 0.0404109 | 2.7063545 | 3.2256452 |
| 11 | C | C9 | -1.2045022 | 1.2028254 | 2.2521706 |
| 12 | H | H11 | -2.1324069 | 1.6587688 | 2.5871540 |
| 13 | C | C10 | -1.2272576 | 0.0556325 | 1.5131135 |
| 14 | H | H12 | -2.1709460 | -0.4165290 | 1.2572406 |
| 15 | C | C3 | 2.4490914 | -0.7115405 | -1.1506657 |
| 16 | C | C4 | 2.4742416 | -0.5391727 | 1.1292349 |
| 17 | C | C11 | 2.6692876 | -1.5584310 | 0.0591998 |
| 18 | H | H2 | 3.3809668 | -0.3191597 | -1.5489410 |
| 19 | H | H10 | 3.3945289 | -0.0738765 | 1.4714696 |
| 20 | C | C12 | -1.0180917 | 0.6571089 | -1.4359339 |
| 21 | O | O1 | -0.7001388 | 1.8127592 | -1.6418218 |
| 22 | O | O2 | -2.3096672 | 0.2444313 | -1.4290949 |
| 23 | C | C13 | -3.2803384 | 1.2756347 | -1.6612080 |
| 24 | H | H1 | -3.1226569 | 1.7401591 | -2.6382425 |
| 25 | H | H5 | -3.2131662 | 2.0462063 | -0.8880948 |
| 26 | H | H8 | -4.2502372 | 0.7774966 | -1.6258174 |
| 27 | F | F1 | 1.8593515 | -2.6707964 | 0.1829681 |
| 28 | F | F2 | 3.9592687 | -2.0250141 | 0.0765105 |
| 29 | C | C14 | -0.6043500 | -1.8668552 | -1.3905727 |
| 30 | H | H6 | 0.1631754 | -2.6169170 | -1.2093664 |
| 31 | H | H13 | -0.9247594 | -1.9509253 | -2.4368304 |
| 32 | H | H14 | -1.4776266 | -2.0875518 | -0.7703345 |

## Alpha-Me furan trans intermediate (25a)

Spartan'10: G = -889.603013, iv = none
Gaussian'09: B3LYP/6-31G*, G = -889.610117, iv = none
Gaussian'09: M05-2X/6-31+G*, G = -889.546801, iv = none

Cartesian Coordinates (Angstroms)

| | Atom | | X | Y | Z |
|---|---|---|---|---|---|
| 1 | C | C1 | 1.5534980 | 1.4511661 | -0.8407189 |
| 2 | C | C2 | 0.4917211 | 1.7903833 | 0.1279921 |
| 3 | C | C3 | 0.9244027 | 0.2753336 | -0.1820042 |
| 4 | H | H3 | -0.4628635 | 2.0583940 | -0.3175496 |
| 5 | H | H6 | 1.5218784 | -0.1559734 | 0.6148856 |
| 6 | C | C10 | -0.0116118 | -0.5872870 | -0.9142971 |
| 7 | H | H11 | -0.4795535 | -0.1381529 | -1.7855085 |
| 8 | C | C11 | -0.3444786 | -1.8574102 | -0.6079688 |
| 9 | C | C12 | -1.3239442 | -2.5939509 | -1.4571208 |
| 10 | O | O1 | -1.6466618 | -3.7502884 | -1.2574843 |
| 11 | O | O2 | -1.8309451 | -1.8576180 | -2.4768333 |
| 12 | C | C13 | -2.7730240 | -2.5429624 | -3.3135530 |
| 13 | H | H5 | -2.2981176 | -3.3916242 | -3.8139591 |
| 14 | H | H13 | -3.1050025 | -1.8051376 | -4.0450116 |
| 15 | H | H14 | -3.6177249 | -2.9086313 | -2.7238128 |
| 16 | F | F1 | 1.3171784 | 1.6717582 | -2.1548017 |
| 17 | F | F2 | 2.8454017 | 1.7427335 | -0.5556694 |
| 18 | C | C4 | 0.7592403 | 2.3888452 | 1.4405016 |
| 19 | C | C5 | 1.8814512 | 2.7972985 | 2.1068452 |
| 20 | C | C6 | 1.4445417 | 3.2683479 | 3.3872879 |
| 21 | C | C7 | 0.0927241 | 3.1158777 | 3.4074928 |
| 22 | H | H1 | 2.8913403 | 2.7738015 | 1.7262248 |
| 23 | H | H8 | 2.0596539 | 3.6732968 | 4.1787556 |
| 24 | H | H9 | -0.6741849 | 3.3331767 | 4.1351185 |
| 25 | O | O3 | -0.3444406 | 2.5796988 | 2.2324140 |
| 26 | C | C8 | 0.1868351 | -2.6638126 | 0.5478402 |
| 27 | H | H2 | 0.9275234 | -2.1143936 | 1.1329438 |
| 28 | H | H4 | 0.6441148 | -3.5929560 | 0.1908463 |
| 29 | H | H7 | -0.6289523 | -2.9599131 | 1.2171449 |

## alpha Me Furan Cis (cis-25a)

Spartan'10: G = --889.601018, iv = none
Gaussian'09: B3LYP/6-31G*, G = -889.608862, iv = none
Gaussian'09: M06-2X/6-31G*, G = -889.261077, iv = none

Cartesian Coordinates (Angstroms)

| | Atom | | X | Y | Z |
|---|---|---|---|---|---|
| 1 | C | C1 | -0.3974741 | -2.6219144 | -0.7714089 |
| 2 | C | C2 | -0.7247780 | -2.2690318 | 0.6247589 |

```
  3 C   C3           -1.1395947    -1.3394771    -0.6178839
  4 H   H6           -2.1994387    -1.4005129    -0.8440415
  5 C   C10          -0.4706465    -0.0488947    -0.8306487
  6 H   H11           0.5991262    -0.0217513    -0.6541930
  7 C   C11          -1.0889629     1.0876410    -1.2085464
  8 C   C12          -0.2992271     2.3444460    -1.3638573
  9 O   O1           -0.7798013     3.4150566    -1.6872710
 10 O   O2            1.0223717     2.1864956    -1.1093573
 11 C   C13           1.8179072     3.3713603    -1.2452303
 12 H   H5            1.7452196     3.7714566    -2.2602172
 13 H   H13           2.8408053     3.0620862    -1.0270429
 14 H   H14           1.4908954     4.1394187    -0.5386698
 15 F   F1            0.8932726    -2.5849295    -1.1823553
 16 F   F2           -1.0238371    -3.6754609    -1.3426948
 17 C   C8           -2.5694172     1.2172905    -1.4795333
 18 H   H2           -2.9498456     0.3755506    -2.0671873
 19 H   H4           -2.7689453     2.1413464    -2.0245422
 20 H   H7           -3.1435270     1.2566239    -0.5445140
 21 H   H12          -1.5836150    -2.7932609     1.0376843
 22 C   C4            0.2948733    -1.8679838     1.6026016
 23 C   C5            1.6595919    -1.9238444     1.6504779
 24 C   C6            2.0461629    -1.3645208     2.9116015
 25 C   C7            0.8917900    -1.0125971     3.5394103
 26 O   O3           -0.1860522    -1.3079138     2.7592418
 27 H   H8            2.3055975    -2.3206775     0.8820351
 28 H   H10           3.0502738    -1.2492973     3.2950736
 29 H   H15           0.6672747    -0.5667041     4.4963103
```

## alpha Me Furan VCPR TS

```
Spartan'10: G = -889.565926, iv = i88, 0.7630
Gaussian'09: B3LYP/6-31G*, G = -889.569816, iv = i85, S^2 = 0.76
Gaussian'09: M05-2X/6-31+G*, G = -889.502352, iv = i56, S^2 = 0.9266

 Cartesian Coordinates (Angstroms)
       Atom             X              Y              Z
   ---------    ------------- ------------- -------------

  1 C   C            -0.4041129     2.5914948    -0.9599034
  2 C   C2           -1.4497983     1.7333183    -0.9510775
  3 C   C3           -1.5151473     0.4795265    -0.2387479
  4 C   C4            0.9435780     2.2099149    -0.4366464
  5 C   C5            1.2510123     0.7573553    -0.6097426
  6 H   H            -0.4729379     3.5694436    -1.4267338
  7 H   H7           -2.3116161     1.9696306    -1.5753268
  8 H   H12           0.9287033     0.3061349    -1.5389267
  9 F   F1            1.8954887     2.9697258    -1.1062616
 10 F   F2            1.0946380     2.5940510     0.9005045
 11 C   C11          -2.2979979    -0.5701880    -0.9151502
 12 O   O1           -2.7696087    -0.4709860    -2.0386564
 13 O   O2           -2.4406324    -1.6860087    -0.1536856
 14 C   C12          -3.1718374    -2.7532977    -0.7712530
 15 H   H1           -4.1968192    -2.4421135    -0.9921350
 16 H   H9           -3.1665562    -3.5696404    -0.0474465
 17 H   H10          -2.6905039    -3.0619753    -1.7032552
 18 C   C1            2.1664149     0.0632262     0.1719121
```

```
 19 C   C6            2.8457301     0.3285411     1.3714154
 20 C   C7            3.6296160    -0.8085007     1.6620184
 21 C   C8            3.3922828    -1.7109159     0.6550611
 22 H   H3            2.7687563     1.2442339     1.9361133
 23 H   H5            4.2858374    -0.9543082     2.5087800
 24 H   H11           3.7547219    -2.7086143     0.4573138
 25 O   O3            2.5168398    -1.2117084    -0.2505679
 26 C   C9           -1.1929891     0.3198419     1.2245581
 27 H   H2           -0.6549729    -0.6120974     1.4253217
 28 H   H4           -2.1297930     0.2662678     1.7999525
 29 H   H6           -0.6082963     1.1576479     1.6025651
```

## alpha Me Furan [3,3] TS

```
Spartan'10: G = -889.572731, iv = i272
Gaussian'09: B3LYP/6-31G*, G = -889.570319, iv = i247
Gaussian'09: M06-2X/6-31G*, G = -889.219479, iv = i329

    Cartesian Coordinates (Angstroms)
       Atom             X             Y             Z
    ---------     ------------- ------------- -------------

  1 C   C1           -0.0507601    -0.4543224     1.0779417
  2 C   C2            1.3285473    -0.1922498     1.4254050
  3 C   C5            1.2591109     0.6796953    -1.4833306
  4 H   H7            1.2276939     1.7135085    -1.8212804
  5 C   C6            0.0194505     0.0856812    -1.1461993
  6 H   H6           -0.1045372    -0.9757655    -1.2924427
  7 C   C3            2.4871096     0.0990027    -1.2918649
  8 C   C4            2.5242029    -0.5775166     0.8877604
  9 C   C11           2.7170973    -1.1330789    -0.4856225
 10 H   H2            3.3934558     0.6452063    -1.5353547
 11 H   H10           3.4272686    -0.1629699     1.3237368
 12 C   C12          -1.1932294     0.9112987    -1.2931720
 13 O   O1           -1.2210239     2.1234481    -1.3997915
 14 O   O2           -2.3211897     0.1474670    -1.2620386
 15 C   C13          -3.5513839     0.8747359    -1.3868180
 16 H   H1           -3.5914586     1.4020560    -2.3439272
 17 H   H5           -3.6540328     1.6057142    -0.5796587
 18 H   H8           -4.3426300     0.1260135    -1.3300535
 19 F   F1            1.8935485    -2.1879088    -0.8149991
 20 F   F2            4.0033845    -1.5811108    -0.6317074
 21 O   O3            1.3742344     0.8415684     2.3582190
 22 C   C8            0.0889164     1.2342896     2.5778535
 23 H   H12          -0.0279724     2.0390440     3.2899567
 24 C   C7           -0.7988195     0.5208325     1.8549223
 25 H   H11          -1.8752294     0.6180992     1.8757905
 26 C   C9           -0.6351157    -1.8391381     0.8793952
 27 H   H3            0.0482415    -2.5028530     0.3522604
 28 H   H4           -1.5800524    -1.7834940     0.3308222
 29 H   H9           -0.8448273    -2.2772530     1.8641973
```

## alpha Me thiophene trans INT

```
Spartan'10: G = -1212.58519, iv = none
Gaussian'09: B3LYP/6-31G*, G = -1212.592277, iv = none
Gaussian'09: M05-2X/6-31+G*, G = -1212.515989, iv = none

     Cartesian Coordinates (Angstroms)
       Atom             X              Y              Z
    ---------     -------------  -------------  -------------

  1  C   C1          1.3238346      1.8053593     -1.0873313
  2  C   C2          0.3236245      2.0555461     -0.0261839
  3  C   C3          0.7947459      0.5774852     -0.4337566
  4  H   H3         -0.6629199      2.2910881     -0.4193521
  5  H   H6          1.4671961      0.1447690      0.3006432
  6  C   C10        -0.1521435     -0.2998764     -1.1306003
  7  H   H11        -0.7086025      0.1470115     -1.9516066
  8  C   C11        -0.4132408     -1.5938060     -0.8575352
  9  C   C12        -1.4436718     -2.2378082     -1.7276760
 10  O   O1         -2.0529892     -1.6948496     -2.6300681
 11  O   O2         -1.6367371     -3.5367477     -1.3911883
 12  C   C13        -2.6140969     -4.2329163     -2.1782058
 13  H   H5         -3.5922056     -3.7524431     -2.0896039
 14  H   H13        -2.6456048     -5.2463562     -1.7764506
 15  H   H14        -2.3212873     -4.2449945     -3.2316104
 16  F   F1          0.9666480      2.0709919     -2.3647945
 17  F   F2          2.6255702      2.1330297     -0.9051421
 18  C   C4          0.6368519      2.6075189      1.3066015
 19  C   C5          1.8186201      3.0845746      1.8198117
 20  C   C6          1.7098868      3.5215417      3.1739268
 21  C   C7          0.4500156      3.3767994      3.6831748
 22  H   H1          2.7336715      3.1321452      1.2437964
 23  H   H8          2.5379027      3.9365040      3.7385555
 24  H   H9          0.0885315      3.6356113      4.6692928
 25  S   S1         -0.6328297      2.6926397      2.5135808
 26  C   C8          0.2286312     -2.4216599      0.2250870
 27  H   H2          0.9683101     -1.8523869      0.7935081
 28  H   H4          0.7256047     -3.3028920     -0.1960225
 29  H   H7         -0.5233161     -2.7958786      0.9291498
```

## alpha Me thiophene cis INT

```
Spartan'10: G = -1212.58342, iv = none
Gaussian'09: B3LYP/6-31G*, G = -1212.590736, iv = none
Gaussian'09: M06-2X/6-31G*, G = -1212.235623, iv = none

   Cartesian Coordinates (Angstroms)
       Atom             X              Y              Z
    ---------     -------------  -------------  -------------

  1  C   C1         -0.3940924     -2.6141307     -0.8489641
  2  C   C2         -0.6969888     -2.3231410      0.5698131
  3  C   C3         -1.1912896     -1.3758097     -0.6243956
  4  H   H6         -2.2506758     -1.4802514     -0.8338615
  5  C   C10        -0.5785213     -0.0523812     -0.8104740
```

| | | | | | |
|---|---|---|---|---|---|
| 6 | H | H11 | 0.4985106 | -0.0069438 | -0.6890946 |
| 7 | C | C11 | -1.2258589 | 1.0922481 | -1.1062525 |
| 8 | C | C12 | -0.4554745 | 2.3600827 | -1.2657540 |
| 9 | O | O1 | -0.9745590 | 3.4315331 | -1.5179732 |
| 10 | O | O2 | 0.8816684 | 2.2138075 | -1.1053489 |
| 11 | C | C13 | 1.6495322 | 3.4162353 | -1.2480852 |
| 12 | H | H5 | 1.5244135 | 3.8375129 | -2.2492658 |
| 13 | H | H13 | 2.6864147 | 3.1213705 | -1.0834070 |
| 14 | H | H14 | 1.3404552 | 4.1617108 | -0.5102149 |
| 15 | F | F1 | 0.8754880 | -2.5058601 | -1.3074883 |
| 16 | F | F2 | -0.9959479 | -3.6751708 | -1.4339463 |
| 17 | C | C8 | -2.7101438 | 1.2581473 | -1.3035409 |
| 18 | H | H2 | -3.2576160 | 0.3225437 | -1.1691833 |
| 19 | H | H4 | -2.9256928 | 1.6449311 | -2.3055924 |
| 20 | H | H7 | -3.1088181 | 1.9955476 | -0.5985513 |
| 21 | H | H12 | -1.5180544 | -2.9178563 | 0.9655118 |
| 22 | C | C4 | 0.3183431 | -1.8915918 | 1.5540113 |
| 23 | C | C5 | 1.6851787 | -2.0166016 | 1.5284367 |
| 24 | C | C6 | 2.3232695 | -1.4802018 | 2.6874215 |
| 25 | C | C7 | 1.4390497 | -0.9528049 | 3.5858528 |
| 26 | S | S1 | -0.1970221 | -1.0952734 | 3.0276960 |
| 27 | H | H8 | 2.2159130 | -2.4811604 | 0.7070238 |
| 28 | H | H10 | 3.3963745 | -1.4948614 | 2.8431709 |
| 29 | H | H15 | 1.6461442 | -0.4916305 | 4.5424558 |

## alpha Me thiophene VCPR TS

Spartan'10: G = -1212.54641, iv = i83, S^2 = 0.7803
Gaussian'09: B3LYP/6-31G*, G = -1212.551391, iv = i86, S^2 = 0.58
Gaussian'09: M05-2X/6-31+G*, G = -1173.189048, iv = i147, S^2 = 0.76

Cartesian Coordinates (Angstroms)

| Atom | | | X | Y | Z |
|---|---|---|---|---|---|
| 1 | C | C | -0.4286277 | 2.7001455 | -0.9162575 |
| 2 | C | C2 | -1.4696176 | 1.8359440 | -0.9008575 |
| 3 | C | C3 | -1.5298555 | 0.5868081 | -0.1810509 |
| 4 | C | C4 | 0.9236504 | 2.3299843 | -0.3973802 |
| 5 | C | C5 | 1.2423567 | 0.8764220 | -0.5519030 |
| 6 | H | H | -0.5044532 | 3.6750544 | -1.3882538 |
| 7 | H | H7 | -2.3326688 | 2.0647375 | -1.5261167 |
| 8 | H | H12 | 0.9100625 | 0.4291747 | -1.4816979 |
| 9 | F | F1 | 1.8675989 | 3.0832268 | -1.0833248 |
| 10 | F | F2 | 1.0843598 | 2.7255446 | 0.9334470 |
| 11 | C | C11 | -2.3012032 | -0.4732423 | -0.8537228 |
| 12 | O | O1 | -2.7566482 | -0.3912181 | -1.9855045 |
| 13 | O | O2 | -2.4475340 | -1.5807004 | -0.0805073 |
| 14 | C | C12 | -3.1677261 | -2.6588455 | -0.6922473 |
| 15 | H | H1 | -4.1896634 | -2.3530341 | -0.9335474 |
| 16 | H | H9 | -3.1724031 | -3.4633918 | 0.0441862 |
| 17 | H | H10 | -2.6722572 | -2.9815926 | -1.6123564 |
| 18 | C | C1 | 2.1479112 | 0.1502133 | 0.2188714 |
| 19 | C | C6 | 2.5984777 | -1.1573475 | -0.0604199 |
| 20 | C | C7 | 3.5003476 | -1.6637240 | 0.8898238 |
| 21 | C | C8 | 3.7575592 | -0.7702064 | 1.9093507 |

```
 22 H   H3            2.2685549    -1.6969482    -0.9416766
 23 H   H5            3.9518922    -2.6480383     0.8325259
 24 H   H11           4.4127766    -0.9097496     2.7593308
 25 S   S1            2.8858342     0.7148177     1.7306140
 26 C   C9           -1.1945736     0.4309267     1.2788112
 27 H   H2           -0.6295144    -0.4869083     1.4728387
 28 H   H4           -2.1251082     0.3495401     1.8604097
 29 H   H6           -0.6295277     1.2824074     1.6566148
```

## alpha Me thiophene 3,3 TS

```
Spartan'10: G = -1212.54839, iv = i263
Gaussian'09: B3LYP/6-31G*, G = -1173.27068, iv = 292
Gaussian'09: M06-2X/6-31G*, G = -1172.930053, iv = i335

Cartesian Coordinates (Angstroms)
        Atom              X             Y             Z
   ---------    ------------- ------------- -------------

  1 C   C1            0.1457678    -0.4810391     1.3189222
  2 H   H3            0.1130342    -1.5256464     1.0528317
  3 C   C2            1.4068513     0.1148581     1.6544117
  4 C   C5            1.1974107     0.2862417    -1.4050061
  5 H   H7            1.1652146     1.2606365    -1.8906403
  6 C   C6           -0.0702641    -0.2276530    -1.0040764
  7 C   C3            2.4511871    -0.1973151    -1.1229512
  8 C   C4            2.6415093    -0.2034674     1.1489605
  9 C   C11           2.8414681    -1.1165508    -0.0159610
 10 H   H2            3.3075510     0.3408363    -1.5206281
 11 H   H10           3.5263145     0.3521231     1.4426269
 12 C   C12          -1.1599725     0.7907009    -1.0778121
 13 O   O1           -0.9961082     1.9873594    -1.2296738
 14 O   O2           -2.3904437     0.2366229    -0.9236220
 15 C   C13          -3.4816825     1.1680769    -0.9479221
 16 H   H1           -3.5329320     1.6767006    -1.9144631
 17 H   H5           -3.3642256     1.9185182    -0.1616101
 18 H   H8           -4.3799083     0.5719050    -0.7817891
 19 F   F1            2.1428866    -2.2998007     0.0822550
 20 F   F2            4.1653004    -1.4563063    -0.1251042
 21 S   S1            1.1568384     1.5248199     2.7115962
 22 C   C8           -0.5628487     1.2204223     2.7615546
 23 H   H12          -1.1944145     1.8597683     3.3654718
 24 C   C7           -0.9435487     0.1521811     2.0143278
 25 H   H11          -1.9682185    -0.1939008     1.9442078
 26 C   C9           -0.4705883    -1.6687664    -1.2745490
 27 H   H4            0.3836005    -2.3380882    -1.1976401
 28 H   H6           -0.8652987    -1.7402301    -2.2972913
 29 H   H9           -1.2644803    -2.0130071    -0.6064268
```

## Weinreb cis E INT (29a)

```
Spartan'10: G = -947.065987, iv = none
Gaussian'09: B3LYP/6-31G*, G = -947.073972, iv = none
Gaussian'09: M06-2X/6-31G*, G = -946.691616, iv = none

Cartesian Coordinates (Angstroms)
       Atom              X             Y             Z
    ---------    ------------- ------------- -------------

  1 C   C1         -2.3342695    -1.5454125    -1.3511858
  2 C   C2         -2.7447096    -0.4462149    -0.4551097
  3 C   C3         -1.4273573    -0.3577660    -1.3337565
  4 H   H6         -1.5152749     0.2988081    -2.1960640
  5 C   C4         -2.6988380    -0.5252874     1.0397700
  6 C   C5         -2.6905013    -0.5938805     3.8467547
  7 C   C6         -2.2668534     0.5848170     1.7792730
  8 C   C7         -3.1259504    -1.6709614     1.7235669
  9 C   C8         -3.1207600    -1.7036023     3.1190950
 10 C   C9         -2.2629843     0.5521683     3.1726594
 11 H   H4         -1.9246668     1.4736453     1.2554516
 12 H   H7         -3.4552808    -2.5399206     1.1631142
 13 H   H8         -3.4517391    -2.6000725     3.6365206
 14 H   H9         -1.9215411     1.4191895     3.7317247
 15 H   H10        -2.6846636    -0.6219162     4.9330309
 16 C   C10        -0.0989138    -0.4383716    -0.7096473
 17 H   H11         0.0410455    -1.1351713     0.1130367
 18 C   C11         0.9532007     0.2940602    -1.1057679
 19 H   H12         0.8719660     1.0115473    -1.9139824
 20 C   C12         2.2567329     0.1334208    -0.4136725
 21 O   O1          2.4230121    -0.6104655     0.5490410
 22 N   N1          3.3366603     0.8264438    -0.9512772
 23 C   C13         4.5433780     1.0426835    -0.1707411
 24 H   H5          4.6505058     0.1942966     0.5044075
 25 H   H13         4.4740857     1.9710549     0.4103888
 26 H   H14         5.4103153     1.0977487    -0.8358071
 27 F   F1         -1.8898330    -2.7129702    -0.8300171
 28 F   F2         -3.0590584    -1.7918138    -2.4682801
 29 O   O2          3.0426753     1.9122541    -1.8046925
 30 C   C14         3.4748363     1.6169464    -3.1354056
 31 H   H2          3.2608473     2.5197991    -3.7136277
 32 H   H1          2.9239356     0.7653288    -3.5521093
 33 H   H15         4.5506512     1.4065694    -3.1695807
 34 H   H16        -3.5406528     0.1730448    -0.8671103
```

## Weinreb [3,3] TS

Spartan'10: G = -947.017003, iv = i374
Gaussian'09: B3LYP/6-31G*, G = -947.020971, iv = i276
Gaussian'09: M06-2X/6-31G*, G = -946.63488, iv = i451

Cartesian Coordinates (Angstroms)

| | | Atom | X | Y | Z |
|---|---|---|---|---|---|
| 1 | C | C1 | -0.2914116 | -0.5301221 | 1.3258970 |
| 2 | H | H3 | -0.1490741 | -1.5987044 | 1.2410897 |
| 3 | C | C2 | -1.5992406 | -0.1288100 | 1.8498740 |
| 4 | C | C5 | -1.7065614 | -0.0875395 | -1.1823929 |
| 5 | H | H7 | -1.7936212 | 0.8376777 | -1.7473401 |
| 6 | C | C6 | -0.3866717 | -0.4669373 | -0.7768860 |
| 7 | H | H6 | -0.1859351 | -1.5272410 | -0.8212505 |
| 8 | C | C7 | -1.6684294 | 1.0558448 | 2.6685345 |
| 9 | H | H4 | -2.6462624 | 1.3782920 | 3.0177572 |
| 10 | C | C8 | -0.5526258 | 1.7769196 | 2.9728796 |
| 11 | H | H9 | -0.6332733 | 2.6798318 | 3.5717877 |
| 12 | C | C9 | 0.7381171 | 1.3485167 | 2.5196794 |
| 13 | H | H11 | 1.6188502 | 1.9132518 | 2.8122116 |
| 14 | C | C10 | 0.8642911 | 0.2210823 | 1.7668847 |
| 15 | H | H12 | 1.8503260 | -0.1294780 | 1.4762307 |
| 16 | C | C3 | -2.8492911 | -0.7208373 | -0.7866194 |
| 17 | C | C4 | -2.7675325 | -0.7699790 | 1.4889247 |
| 18 | C | C11 | -2.9136347 | -1.7018603 | 0.3353786 |
| 19 | H | H2 | -3.8192515 | -0.3540813 | -1.1095863 |
| 20 | H | H10 | -3.7145119 | -0.4114708 | 1.8831897 |
| 21 | C | C12 | 0.6929765 | 0.4679799 | -1.2320579 |
| 22 | O | O1 | 0.4761184 | 1.6632645 | -1.3886650 |
| 23 | N | N1 | 1.9294753 | -0.0828262 | -1.5421181 |
| 24 | F | F1 | -1.9577050 | -2.6994676 | 0.2803596 |
| 25 | F | F2 | -4.1312168 | -2.3293136 | 0.3714625 |
| 26 | C | C13 | 2.4761775 | -1.3675948 | -1.1439412 |
| 27 | H | H1 | 3.2010075 | -1.6879700 | -1.8983552 |
| 28 | H | H5 | 1.6857440 | -2.1163524 | -1.0894241 |
| 29 | H | H8 | 2.9888294 | -1.3077874 | -0.1739330 |
| 30 | O | O2 | 2.9719230 | 0.8527016 | -1.6718440 |
| 31 | C | C14 | 3.0387716 | 1.3529058 | -3.0114028 |
| 32 | H | H13 | 3.1756382 | 0.5367688 | -3.7324742 |
| 33 | H | H14 | 3.9163757 | 2.0046060 | -3.0220940 |
| 34 | H | H15 | 2.1416286 | 1.9287295 | -3.2517563 |

## CN E cis (*cis*-10a)

Spartan'10: G = -716.911798, iv = none
Gaussian'09: B3LYP/6-31G*, G = -716.91601, iv = none
Gaussian'09: M06-2X/6-31G*, G = -716.635411, iv = none

Cartesian Coordinates (Angstroms)

| | | Atom | X | Y | Z |
|---|---|---|---|---|---|
| 1 | C | C1 | 0.8384972 | -0.0293373 | -2.2221422 |
| 2 | C | C2 | -0.3885136 | 0.3527766 | -1.4965935 |
| 3 | C | C3 | 0.0569712 | -1.1649306 | -1.6406357 |
| 4 | H | H3 | -1.2259717 | 0.5801881 | -2.1550650 |
| 5 | C | C4 | -0.4016202 | 1.0552264 | -0.1737740 |
| 6 | C | C5 | -0.5390172 | 2.4067366 | 2.2840982 |
| 7 | C | C6 | -1.3816954 | 0.7197947 | 0.7719740 |
| 8 | C | C7 | 0.5083578 | 2.0774201 | 0.1262152 |
| 9 | C | C8 | 0.4386420 | 2.7474573 | 1.3492651 |
| 10 | C | C9 | -1.4511456 | 1.3901265 | 1.9918145 |
| 11 | H | H4 | -2.0912052 | -0.0732301 | 0.5488670 |
| 12 | H | H7 | 1.2735488 | 2.3463932 | -0.5943289 |
| 13 | H | H8 | 1.1521606 | 3.5368782 | 1.5694348 |
| 14 | H | H9 | -2.2152386 | 1.1173901 | 2.7146395 |
| 15 | H | H10 | -0.5905754 | 2.9289998 | 3.2354672 |
| 16 | C | C10 | 0.5670599 | -1.9031929 | -0.4780249 |
| 17 | H | H11 | 1.2154473 | -1.3671086 | 0.2095548 |
| 18 | F | F1 | 0.8806104 | 0.1048032 | -3.5664402 |
| 19 | F | F2 | 2.0528279 | 0.2561481 | -1.6955350 |
| 20 | H | H2 | -0.5309570 | -1.7369171 | -2.3540265 |
| 21 | C | C11 | 0.2694682 | -3.1931334 | -0.2351451 |
| 22 | H | H1 | -0.3753362 | -3.7579873 | -0.9051597 |
| 23 | C | C12 | 0.7710893 | -3.8997362 | 0.8981439 |
| 24 | N | N1 | 1.1665959 | -4.4947655 | 1.8173967 |

## CN E [3,3] TS

Spartan'10: G = -716.870471, iv = i381
Gaussian'09: B3LYP/6-31G*, G = -716.871513, iv = i384
Gaussian'09: M06-2X/6-31G*, G = -716.586383, iv = i459

Cartesian Coordinates (Angstroms)

| | | Atom | X | Y | Z |
|---|---|---|---|---|---|
| 1 | C | C1 | -0.8088086 | -0.5052546 | 0.4670589 |
| 2 | H | H3 | -0.9601465 | -1.5672415 | 0.3333548 |
| 3 | C | C2 | 0.5041776 | -0.1360068 | 1.0003515 |
| 4 | C | C5 | 0.6094299 | 0.0070984 | -2.0576895 |
| 5 | H | H7 | 0.7322234 | 0.9241156 | -2.6299300 |
| 6 | C | C6 | -0.7219691 | -0.3378939 | -1.6323141 |
| 7 | H | H6 | -0.9883166 | -1.3847092 | -1.7170743 |
| 8 | C | C7 | 0.5845518 | 1.0116801 | 1.8701791 |
| 9 | H | H4 | 1.5646116 | 1.3115768 | 2.2327551 |
| 10 | C | C8 | -0.5278186 | 1.7231151 | 2.2079213 |
| 11 | H | H9 | -0.4417853 | 2.5954906 | 2.8497921 |

```
 12  C   C9           -1.8226470     1.3286345     1.7312997
 13  H   H11          -2.6973987     1.8905458     2.0459019
 14  C   C10          -1.9604833     0.2386595     0.9266856
 15  H   H12          -2.9446696    -0.0819697     0.5984315
 16  C   C3            1.7357562    -0.6589915    -1.6744165
 17  C   C4            1.6614246    -0.7759015     0.6076258
 18  C   C11           1.7837623    -1.6715874    -0.5788014
 19  H   H2            2.7107493    -0.3107918    -2.0015767
 20  H   H10           2.6162454    -0.4503849     1.0114748
 21  F   F1            0.8069298    -2.6435541    -0.6632285
 22  F   F2            2.9879160    -2.3219049    -0.5697096
 23  C   C12          -1.7787176     0.5460953    -2.0362881
 24  N   N1           -2.6450171     1.2691801    -2.3218035
```

### CN Z INT (*cis*-10h)

```
Spartan'10: G = -716.911953, iv = none
Gaussian'09: B3LYP/6-31G*, G = -716.916391, iv = none
Gaussian'09: M06-2X/6-31G*, G = --716.637087, iv = none

Cartesian Coordinates (Angstroms)
       Atom            X             Y             Z
    ---------    ------------- ------------- -------------

  1  C   C1           -1.3052491     0.6534900    -1.7473256
  2  C   C2           -1.3174749    -0.1515324    -0.5107186
  3  C   C3           -0.4276179    -0.5571128    -1.7622719
  4  H   H3           -2.1888862    -0.7992368    -0.4249284
  5  C   C4           -0.7118527     0.2902570     0.7861344
  6  C   C5            0.3586852     1.0479699     3.2694727
  7  C   C6           -0.0334426    -0.6418633     1.5849899
  8  C   C7           -0.8476052     1.6066244     1.2466254
  9  C   C8           -0.3145495     1.9816313     2.4812757
 10  C   C9            0.4975196    -0.2667018     2.8179120
 11  H   H4            0.0793366    -1.6643518     1.2335346
 12  H   H7           -1.3672142     2.3390743     0.6376639
 13  H   H8           -0.4277317     3.0060297     2.8256598
 14  H   H9            1.0205771    -1.0003674     3.4254199
 15  H   H10           0.7729671     1.3412882     4.2302219
 16  C   C10           1.0319841    -0.4074183    -1.7078640
 17  H   H11           1.4187922     0.4888395    -1.2314107
 18  C   C11           1.9199131    -1.2914217    -2.2029974
 19  H   H12           2.9864701    -1.1004944    -2.1261998
 20  F   F1           -0.7198674     1.8760887    -1.7499297
 21  F   F2           -2.3991365     0.6706445    -2.5398610
 22  H   H2           -0.8069124    -1.4069014    -2.3229696
 23  C   C12           1.5417345    -2.5090008    -2.8455583
 24  N   N1            1.2395607    -3.5055343    -3.3668747
```

## CN Z [3,3] TS

Spartan'10: G = -716.863331, iv = none
Gaussian'09: B3LYP/6-31G*, G = -716.864276, iv = i384
Gaussian'09: M06-2X/6-31G*, G = -716.578656, iv = i458

Cartesian Coordinates (Angstroms)

| Atom | | | X | Y | Z |
|---|---|---|---|---|---|
| 1 | C | C1 | -0.8423313 | -0.3854496 | 0.4662477 |
| 2 | H | H3 | -0.9858849 | -1.4242021 | 0.2074645 |
| 3 | C | C2 | 0.4608799 | -0.0289211 | 1.0064344 |
| 4 | C | C5 | 0.6213220 | 0.2719571 | -2.0300150 |
| 5 | H | H7 | 0.8147920 | 1.2431589 | -2.4844903 |
| 6 | C | C6 | -0.7472213 | 0.0044337 | -1.6969464 |
| 7 | H | H8 | -1.4126024 | 0.8586842 | -1.8178538 |
| 8 | C | C7 | 0.5317204 | 1.0873507 | 1.9133168 |
| 9 | H | H4 | 1.5069625 | 1.3804196 | 2.2941124 |
| 10 | C | C8 | -0.5896368 | 1.7785552 | 2.2738744 |
| 11 | H | H9 | -0.5117713 | 2.6244792 | 2.9512872 |
| 12 | C | C9 | -1.8760834 | 1.3957891 | 1.7765733 |
| 13 | H | H11 | -2.7567043 | 1.9402548 | 2.1060845 |
| 14 | C | C10 | -1.9991775 | 0.3320961 | 0.9280028 |
| 15 | H | H12 | -2.9767536 | 0.0045472 | 0.5842892 |
| 16 | C | C3 | 1.7198049 | -0.4566810 | -1.6635881 |
| 17 | C | C4 | 1.6261879 | -0.6463821 | 0.5838551 |
| 18 | C | C11 | 1.7519420 | -1.5222970 | -0.6186132 |
| 19 | H | H2 | 2.7069898 | -0.1011847 | -1.9440974 |
| 20 | H | H10 | 2.5791735 | -0.3257808 | 0.9957299 |
| 21 | F | F1 | 0.7885549 | -2.4881404 | -0.7296507 |
| 22 | F | F2 | 2.9645099 | -2.1596987 | -0.6208029 |
| 23 | C | C12 | -1.3931786 | -1.2146902 | -2.0820448 |
| 24 | N | N1 | -1.9814943 | -2.1682982 | -2.3991697 |

**Literature Predictions**

## Erbes and Boland trans SM (trans-49)

Gaussian'09: B3LYP/6-31G*, G = -628.208979, iv = none

Cartesian Coordinates (Angstroms)

| | | Atom | X | Y | Z |
|---|---|---|---|---|---|
| 1 | C | C1 | 2.1824440 | 2.4440161 | -0.9529439 |
| 2 | C | C2 | 0.8731754 | 3.0361150 | -0.5198276 |
| 3 | C | C3 | 1.0267060 | 1.5332680 | -0.6077047 |
| 4 | H | H6 | 1.0998863 | 1.0140289 | 0.3462317 |
| 5 | C | C10 | 0.3221516 | 0.7643655 | -1.6561767 |
| 6 | H | H11 | 0.2760916 | 1.2185969 | -2.6460780 |
| 7 | C | C11 | -0.2358471 | -0.4350329 | -1.4712997 |
| 8 | H | H15 | -0.7345043 | -0.9617367 | -2.2797294 |
| 9 | C | C4 | 0.7568048 | 3.6565147 | 0.8060946 |
| 10 | C | C6 | 0.0066983 | 4.7264722 | 1.1004036 |
| 11 | H | H1 | -0.5936315 | 5.2214553 | 0.3431933 |
| 12 | H | H13 | -0.0211473 | 5.1271845 | 2.1088408 |
| 13 | H | H17 | 1.3404568 | 3.2048235 | 1.6081830 |
| 14 | F | F1 | 3.2781083 | 2.4714643 | -0.1094657 |
| 15 | F | F2 | 2.4980711 | 2.5871280 | -2.2918194 |
| 16 | C | C5 | -0.2048685 | -1.1384911 | -0.1118337 |
| 17 | H | H3 | 0.8356307 | -1.2967445 | 0.1939984 |
| 18 | H | H5 | -0.7140301 | -0.5163774 | 0.6331126 |
| 19 | C | C7 | -0.9161152 | -2.4899150 | -0.2201975 |
| 20 | H | H4 | -0.4069540 | -3.1120293 | -0.9651436 |
| 21 | H | H7 | -1.9566144 | -2.3316614 | -0.5260294 |
| 22 | C | C8 | -0.8851375 | -3.1933728 | 1.1392687 |
| 23 | H | H8 | 0.1553615 | -3.3516268 | 1.4451012 |
| 24 | H | H10 | -1.3942988 | -2.5712584 | 1.8842147 |
| 25 | C | C9 | -1.5963850 | -4.5447962 | 1.0309051 |
| 26 | H | H9 | -1.0872241 | -5.1669111 | 0.2859594 |
| 27 | H | H12 | -2.6368840 | -4.3865419 | 0.7250728 |
| 28 | H | H14 | -1.5742096 | -5.0483816 | 2.0041091 |
| 29 | H | H16 | 0.3062651 | 3.5394442 | -1.2964399 |

## Erbes and Boland cis SM (49)

Gaussian'09: B3LYP/6-31G*, G = -628.207323, iv = none

Cartesian Coordinates (Angstroms)

| | | Atom | X | Y | Z |
|---|---|---|---|---|---|
| 1 | C | C1 | 2.3048106 | 2.4092042 | -0.5864680 |
| 2 | C | C2 | 1.0108544 | 3.0286131 | -0.1099256 |
| 3 | C | C3 | 1.1490726 | 1.4984560 | -0.2412288 |
| 4 | H | H6 | 1.2222529 | 0.9792168 | 0.7127076 |
| 5 | C | C10 | 0.4445181 | 0.7295535 | -1.2897008 |
| 6 | H | H11 | 0.3984581 | 1.1837849 | -2.2796021 |
| 7 | C | C11 | -0.1134805 | -0.4698449 | -1.1048238 |
| 8 | H | H2 | 1.0063096 | 3.3831285 | 0.9192383 |

| | | | X | Y | Z |
|---|---|---|---|---|---|
| 9 | H | H15 | -0.6121377 | -0.9965488 | -1.9132536 |
| 10 | C | C4 | 0.1658656 | 3.8275133 | -1.0234979 |
| 11 | C | C6 | -0.5814338 | 4.8690025 | -0.6480115 |
| 12 | H | H1 | -0.6234135 | 5.2003890 | 0.3875069 |
| 13 | H | H13 | -1.1781026 | 5.4289125 | -1.3622669 |
| 14 | H | H17 | 0.1764772 | 3.5403149 | -2.0750644 |
| 15 | F | F1 | 3.4004749 | 2.4366523 | 0.2570101 |
| 16 | F | F2 | 2.6204377 | 2.5523160 | -1.9253435 |
| 17 | C | C5 | -0.0825019 | -1.1733032 | 0.2546422 |
| 18 | H | H3 | 0.9579973 | -1.3315565 | 0.5604743 |
| 19 | H | H5 | -0.5916635 | -0.5511894 | 0.9995885 |
| 20 | C | C7 | -0.7937486 | -2.5247270 | 0.1462784 |
| 21 | H | H4 | -0.2845874 | -3.1468413 | -0.5986678 |
| 22 | H | H7 | -1.8342478 | -2.3664734 | -0.1595536 |
| 23 | C | C8 | -0.7627709 | -3.2281848 | 1.5057446 |
| 24 | H | H8 | 0.2777281 | -3.3864388 | 1.8115770 |
| 25 | H | H10 | -1.2719322 | -2.6060704 | 2.2506905 |
| 26 | C | C9 | -1.4740185 | -4.5796082 | 1.3973810 |
| 27 | H | H9 | -0.9648575 | -5.2017231 | 0.6524352 |
| 28 | H | H12 | -2.5145175 | -4.4213540 | 1.0915487 |
| 29 | H | H14 | -1.4518430 | -5.0831936 | 2.3705850 |

## Erbes and Boland VCPR TS

Gaussian'09: B3LYP/6-31G*, G = -628.163404, iv = i262, S^2 = 0.7753

Cartesian Coordinates (Angstroms)

| Atom | | | X | Y | Z |
|---|---|---|---|---|---|
| 1 | C | C | 0.1797769 | 2.4851927 | -1.7655285 |
| 2 | C | C2 | -0.9439639 | 1.7209381 | -1.7531359 |
| 3 | C | C3 | -0.9491965 | 0.4305106 | -1.1524093 |
| 4 | C | C4 | 1.4649215 | 1.8489004 | -1.3421135 |
| 5 | C | C5 | 1.5881396 | 0.4198029 | -1.7923096 |
| 6 | H | H | 0.2052781 | 3.5166479 | -2.1001947 |
| 7 | H | H7 | -1.8549081 | 2.0559026 | -2.2452659 |
| 8 | H | H12 | 1.1815084 | 0.1887807 | -2.7706469 |
| 9 | F | F1 | 2.5279271 | 2.5825422 | -1.8433748 |
| 10 | F | F2 | 1.6191634 | 1.9073752 | 0.0413634 |
| 11 | C | C1 | 2.4997100 | -0.4644860 | -1.1820841 |
| 12 | H | H3 | 2.9522971 | -0.1411655 | -0.2469181 |
| 13 | C | C6 | 2.8111653 | -1.7003805 | -1.6762756 |
| 14 | H | H2 | 3.4993201 | -2.3607789 | -1.1585262 |
| 15 | H | H4 | 2.3740126 | -2.0681877 | -2.6010585 |
| 16 | H | H16 | -1.7373684 | -0.2135955 | -1.5598184 |
| 17 | C | C7 | -0.4063762 | -0.1645800 | 0.0803931 |
| 18 | H | H1 | 0.3309212 | 0.4487369 | 0.5844297 |
| 19 | H | H5 | 0.0334044 | -1.1397334 | -0.1532436 |
| 20 | C | C8 | -1.5525354 | -0.3853769 | 1.0822626 |
| 21 | H | H8 | -2.3197989 | -1.0295050 | 0.6341871 |
| 22 | H | H9 | -2.0358990 | 0.5747139 | 1.3037845 |
| 23 | C | C9 | -1.0461147 | -1.0194032 | 2.3778715 |
| 24 | H | H6 | -0.2876426 | -0.3746243 | 2.8368189 |
| 25 | H | H10 | -0.5660855 | -1.9803051 | 2.1590619 |
| 26 | C | C10 | -2.1775044 | -1.2408641 | 3.3689390 |

```
 27 H  H11          -1.7931010    -1.6945214     4.2878153
 28 H  H13          -2.9384534    -1.9088238     2.9528676
 29 H  H14          -2.6585977    -0.2937128     3.6331091
```

## Erbes and Boland [3,3] TS

Gaussian'09: B3LYP/6-31G*, G = -628.172483, iv = i284

```
Cartesian Coordinates (Angstroms)
       Atom             X             Y             Z
    ---------    ------------- ------------- -------------

  1 C  C1           -0.7635387     1.2387820     2.6379056
  2 H  H3           -0.9712143     0.1953323     2.4383484
  3 C  C2            0.5214959     1.6455185     2.9610528
  4 C  C5            0.5149436     1.7746469    -0.0281305
  5 H  H7            0.6413982     2.7611400    -0.4746427
  6 C  C6           -0.7712610     1.3484632     0.2653336
  7 C  C3            1.6749619     1.1534769     0.4440985
  8 C  C4            1.6800959     1.0703481     2.4308735
  9 C  C11           1.6956791    -0.0067176     1.3906396
 10 H  H2            2.6396795     1.5699725     0.1427050
 11 H  H10           2.6460830     1.4328050     2.7921912
 12 H  H17          -1.6195833     1.9563442    -0.0370334
 13 H  H1           -1.6149759     1.8093846     2.9985664
 14 H  H11           0.6397727     2.5236634     3.5908744
 15 F  F1            0.5830745    -0.8275205     1.3577380
 16 F  F2            2.8511190    -0.7659216     1.3551840
 17 C  C7           -1.2869475    -0.1133111    -0.2617409
 18 H  H5           -2.3310062     0.0417838    -0.5682532
 19 H  H6           -1.3322165    -0.8273506     0.5612621
 20 C  C8           -0.4980649    -0.6330584    -1.4586575
 21 H  H4            0.5484327    -0.7963460    -1.1815678
 22 H  H8           -0.5024572     0.1240830    -2.2517814
 23 C  C9           -1.0898539    -1.9387801    -1.9875949
 24 H  H12          -2.1338189    -1.7826954    -2.2833101
 25 H  H13          -1.0864397    -2.6963103    -1.1951802
 26 C  C10          -0.3067223    -2.4618498    -3.1811563
 27 H  H9            0.7352312    -2.6601956    -2.9103080
 28 H  H14          -0.3167139    -1.7397327    -4.0038203
 29 H  H15          -0.7471530    -3.3959545    -3.5435962
```

## Gaich trans VCP SM (50)

Gaussian'09: B3LYP/6-31G*, G = -1145.573154, iv = none

```
Cartesian Coordinates (Angstroms)
       Atom             X             Y             Z
    ---------    ------------- ------------- -------------

  1 C  C1            1.6197059    -0.8005199    -2.6549743
  2 C  C2            0.5016609    -0.3525236    -1.8018160
  3 C  C3            0.8278940    -1.9065505    -2.0489682
  4 H  H3           -0.3659227    -0.0185463    -2.3700799
  5 H  H6            1.2947686    -2.3804072    -1.1892069
  6 C  C10          -0.0928219    -2.7135757    -2.8524589
```

| | | | | | |
|---|---|---|---|---|---|
| 7 | H | H11 | -0.4787172 | -2.2731209 | -3.7704171 |
| 8 | C | C11 | -0.5030457 | -3.9515410 | -2.5331951 |
| 9 | H | H12 | -0.1589228 | -4.4670044 | -1.6412409 |
| 10 | C | C4 | 0.6945669 | 0.2400464 | -0.4671944 |
| 11 | C | C5 | 1.7838202 | 0.8462710 | 0.1349866 |
| 12 | C | C6 | 1.3939455 | 1.2356385 | 1.4455614 |
| 13 | C | C7 | 0.0791243 | 0.8580797 | 1.6156185 |
| 14 | H | H1 | 2.7485014 | 0.9944472 | -0.3271107 |
| 15 | N | N1 | -0.3373923 | 0.2577674 | 0.4529992 |
| 16 | H | H4 | 2.6578182 | -0.6578100 | -2.3337691 |
| 17 | H | H7 | 1.5407672 | -0.6324651 | -3.7351328 |
| 18 | H | H10 | -1.1996851 | -4.4813017 | -3.1751735 |
| 19 | C | C8 | -0.5815492 | 1.1320459 | 2.8351548 |
| 20 | C | C12 | 1.4364990 | 2.1607410 | 3.6885985 |
| 21 | C | C13 | 2.0869119 | 1.8917182 | 2.4846385 |
| 22 | C | C14 | 0.1140458 | 1.7824750 | 3.8598686 |
| 23 | H | H13 | 3.1242060 | 2.1867092 | 2.3504244 |
| 24 | H | H14 | -0.3886491 | 1.9853945 | 4.8031395 |
| 25 | H | H16 | -1.6118828 | 0.8380517 | 3.0161146 |
| 26 | H | H18 | 1.9693372 | 2.6622921 | 4.4922558 |
| 27 | S | S1 | -2.0091027 | -0.0544889 | 0.1539555 |
| 28 | O | O1 | -2.1126963 | -0.8254080 | -1.0678739 |
| 29 | O | O2 | -2.5459969 | -0.5894233 | 1.3920482 |
| 30 | C | C9 | -2.7561756 | 1.5265115 | -0.1429219 |
| 31 | H | H2 | -2.2740572 | 1.9858205 | -1.0079086 |
| 32 | H | H5 | -2.6400932 | 2.1531404 | 0.7424699 |
| 33 | H | H8 | -3.8168623 | 1.3675366 | -0.3483918 |

## Gaich cis VCP SM (cis-50)

Gaussian'09: B3LYP/6-31G*, G = -1145.574428, iv = none

Cartesian Coordinates (Angstroms)

| Atom | | | X | Y | Z |
|---|---|---|---|---|---|
| 1 | C | C1 | 0.5772912 | -2.0168209 | -2.6912849 |
| 2 | C | C2 | -0.4755920 | -1.4227026 | -1.8065477 |
| 3 | C | C3 | 0.1486847 | -0.6115404 | -2.9341770 |
| 4 | H | H6 | -0.5652451 | -0.4887302 | -3.7446853 |
| 5 | C | C10 | 1.0297737 | 0.5175099 | -2.6283467 |
| 6 | H | H11 | 1.8128888 | 0.3591332 | -1.8886099 |
| 7 | C | C11 | 0.9343620 | 1.7352010 | -3.1859273 |
| 8 | H | H12 | 0.1886167 | 1.9741007 | -3.9385868 |
| 9 | C | C4 | -0.1797073 | -1.0976278 | -0.3127173 |
| 10 | C | C5 | 0.8118500 | -1.6934701 | 0.4355737 |
| 11 | C | C6 | 0.7786294 | -1.1265360 | 1.7382202 |
| 12 | C | C7 | -0.2780851 | -0.2076168 | 1.7758048 |
| 13 | H | H1 | 1.5091388 | -2.4445218 | 0.0876724 |
| 14 | N | N1 | -0.8679872 | -0.2463571 | 0.5217833 |
| 15 | H | H4 | 0.3076245 | -2.7846842 | -3.4253706 |
| 16 | H | H7 | 1.5763829 | -2.1850111 | -2.3024834 |
| 17 | H | H10 | 1.6182629 | 2.5265533 | -2.8958751 |
| 18 | C | C8 | -0.5639331 | 0.5245644 | 2.9441336 |
| 19 | C | C12 | 1.2864664 | -0.5905565 | 4.0494810 |
| 20 | C | C13 | 1.5661520 | -1.3194300 | 2.8908299 |

| | | | | | |
|---|---|---|---|---|---|
| 21 | C | C14 | 0.2348618 | 0.3177570 | 4.0731321 |
| 22 | H | H13 | 2.3878390 | -2.0303952 | 2.8816971 |
| 23 | H | H14 | 0.0211906 | 0.8691525 | 4.9865939 |
| 24 | H | H16 | -1.3946649 | 1.2226822 | 3.0002371 |
| 25 | H | H18 | 1.8924320 | -0.7413470 | 4.9396372 |
| 26 | S | S1 | -2.1152702 | 0.8215448 | 0.0447368 |
| 27 | O | O1 | -2.5640956 | 0.3965081 | -1.2676831 |
| 28 | O | O2 | -3.0458025 | 0.8515416 | 1.1593617 |
| 29 | C | C9 | -1.3897601 | 2.4303800 | -0.1089514 |
| 30 | H | H2 | -0.5860389 | 2.3920360 | -0.8420049 |
| 31 | H | H5 | -1.0075041 | 2.7484702 | 0.8619686 |
| 32 | H | H8 | -2.1659855 | 3.1214365 | -0.4444398 |
| 33 | H | H9 | -1.4827759 | -1.8012238 | -1.9731721 |

Gaich cis VCPR TS 50
Gaussian'09: B3LYP/6-31G*, G = -1145.512932, iv = i451, S^2 = 0.7540

Cartesian Coordinates (Angstroms)

| | | Atom | X | Y | Z |
|---|---|---|---|---|---|
| 1 | C | C | -3.2306126 | 2.4446878 | 0.6697624 |
| 2 | C | C2 | -4.2202822 | 1.5011075 | 0.6738103 |
| 3 | C | C3 | -3.9420378 | 0.1216369 | 0.7867193 |
| 4 | C | C4 | -1.8298021 | 1.9937313 | 0.5249219 |
| 5 | C | C5 | -1.6742598 | 0.8913056 | -0.4753325 |
| 6 | H | H | -3.4311433 | 3.5154477 | 0.6530915 |
| 7 | H | H7 | -5.2604059 | 1.7955217 | 0.5457293 |
| 8 | H | H8 | -3.0671211 | -0.2330688 | 1.3138945 |
| 9 | H | H12 | -2.4248628 | 0.8236220 | -1.2494718 |
| 10 | C | C1 | -0.4596443 | 0.2321306 | -0.6776137 |
| 11 | C | C6 | -0.2005191 | -0.4626763 | -1.8531098 |
| 12 | C | C7 | 1.1697130 | -0.8601541 | -1.8616532 |
| 13 | C | C8 | 1.7273980 | -0.4742062 | -0.6391548 |
| 14 | H | H3 | -0.8602812 | -0.6009484 | -2.7062280 |
| 15 | N | N1 | 0.6809631 | 0.0224647 | 0.0816724 |
| 16 | H | H2 | -1.4017182 | 1.6667809 | 1.4794185 |
| 17 | H | H4 | -1.1848019 | 2.8381787 | 0.2564089 |
| 18 | H | H13 | -4.7016903 | -0.6156509 | 0.5029046 |
| 19 | C | C9 | 3.0889678 | -0.6561729 | -0.3579910 |
| 20 | C | C11 | 1.9799289 | -1.4934810 | -2.8204477 |
| 21 | C | C12 | 3.3318649 | -1.6992840 | -2.5376187 |
| 22 | C | C13 | 3.8768732 | -1.2773033 | -1.3304490 |
| 23 | H | H1 | 3.5714265 | -0.3184691 | 0.5560382 |
| 24 | H | H14 | 1.5919290 | -1.8166411 | -3.7863838 |
| 25 | H | H15 | 3.9740948 | -2.1817240 | -3.2767897 |
| 26 | H | H17 | 4.9426785 | -1.4328413 | -1.1485794 |
| 27 | S | S1 | 0.5609192 | 0.0277857 | 1.7628150 |
| 28 | O | O1 | 0.7534589 | 1.4339937 | 2.0410944 |
| 29 | O | O2 | -0.8212567 | -0.4545720 | 1.8865347 |
| 30 | C | C10 | 1.7027156 | -1.0271505 | 2.6098716 |
| 31 | H | H5 | 2.7004349 | -0.5918645 | 2.5708941 |
| 32 | H | H6 | 1.6764925 | -2.0178412 | 2.1536728 |
| 33 | H | H9 | 1.3805806 | -1.0943451 | 3.6515687 |

## Gaich cis [3,3] TS

Gaussian'09: B3LYP/6-31G*, G = -1145.535705, iv = i388

Cartesian Coordinates (Angstroms)

| | | Atom | X | Y | Z |
|---|---|---|---|---|---|
| 1 | C | C1 | -0.5354495 | -0.9954063 | -0.6005013 |
| 2 | H | H3 | -0.6066520 | -2.0631530 | -0.7723441 |
| 3 | C | C2 | 0.7837370 | -0.5430534 | -0.1823152 |
| 4 | C | C5 | 0.5332369 | -0.1362140 | -3.0944368 |
| 5 | H | H7 | 0.5127026 | 0.8715480 | -3.5066809 |
| 6 | C | C6 | -0.6930158 | -0.6444662 | -2.5707256 |
| 7 | H | H6 | -0.9282083 | -1.6827987 | -2.7742534 |
| 8 | C | C3 | 1.7525154 | -0.7429426 | -2.9246180 |
| 9 | C | C4 | 1.9459117 | -1.0599260 | -0.7440129 |
| 10 | C | C11 | 1.9810344 | -1.8681167 | -1.9859001 |
| 11 | H | H2 | 2.6585646 | -0.2828496 | -3.3108351 |
| 12 | H | H10 | 2.9153312 | -0.6781863 | -0.4285817 |
| 13 | C | C9 | -1.5088898 | -0.2812568 | 0.1619200 |
| 14 | C | C8 | -0.8226449 | 0.6792747 | 0.7617455 |
| 15 | N | N2 | 0.5383226 | 0.6398357 | 0.6569621 |
| 16 | H | H11 | -1.5487914 | 0.0329326 | -2.6707005 |
| 17 | H | H16 | 1.2454242 | -2.6784578 | -2.0445588 |
| 18 | H | H17 | 2.9656358 | -2.3250472 | -2.1375266 |
| 19 | C | C7 | -2.9003677 | -0.4601979 | 0.3202661 |
| 20 | C | C12 | -1.5704315 | 1.6188409 | 1.5337430 |
| 21 | C | C13 | -2.9562851 | 1.4943808 | 1.6866126 |
| 22 | C | C14 | -3.6226375 | 0.4435674 | 1.0905411 |
| 23 | H | H1 | -3.4073908 | -1.2784191 | -0.1831761 |
| 24 | H | H13 | -1.0869852 | 2.4573567 | 2.0308196 |
| 25 | H | H14 | -3.5021339 | 2.2237180 | 2.2799495 |
| 26 | H | H18 | -4.6966160 | 0.3318542 | 1.2055523 |
| 27 | S | S1 | 1.6919267 | 1.3908105 | 1.6402702 |
| 28 | O | O1 | 2.9810173 | 1.1549805 | 1.0178166 |
| 29 | O | O2 | 1.2241193 | 2.7472334 | 1.8558173 |
| 30 | C | C10 | 1.6640816 | 0.5328646 | 3.1908993 |
| 31 | H | H4 | 2.4112996 | 0.9875830 | 3.8445906 |
| 32 | H | H5 | 1.9092720 | -0.5166522 | 3.0180000 |
| 33 | H | H8 | 0.6723665 | 0.6303628 | 3.6356613 |

## Sustmann VCP INT (51)

Gaussian'09: B3LYP/6-31G*, G = -888.147718, iv = none

Cartesian Coordinates (Angstroms)

| | | Atom | X | Y | Z |
|---|---|---|---|---|---|
| 1 | C | C1 | 2.3755578 | 0.2955231 | -0.2584893 |
| 2 | C | C2 | 1.2437991 | 1.0453606 | 0.3458963 |
| 3 | C | C3 | 1.3323679 | -0.5400432 | 0.4099371 |
| 4 | H | H6 | 1.6293956 | -0.9742177 | 1.3626546 |
| 5 | C | C4 | 1.4964683 | 1.7754558 | 1.6444639 |
| 6 | C | C5 | 1.8975463 | 3.2280305 | 4.0133341 |

| | | | | | |
|---|---|---|---|---|---|
| 7 | C | C6 | 0.8125100 | 1.4295389 | 2.8153903 |
| 8 | C | C7 | 2.3868173 | 2.8567642 | 1.6736107 |
| 9 | C | C8 | 2.5863915 | 3.5777389 | 2.8501506 |
| 10 | C | C9 | 1.0098482 | 2.1516013 | 3.9932360 |
| 11 | H | H4 | 0.1036468 | 0.6053497 | 2.8103845 |
| 12 | H | H7 | 2.9165295 | 3.1509523 | 0.7712183 |
| 13 | H | H8 | 3.2746411 | 4.4187474 | 2.8602973 |
| 14 | H | H9 | 0.4670704 | 1.8787003 | 4.8943076 |
| 15 | C | C10 | 0.4351634 | -1.3920948 | -0.3825193 |
| 16 | H | H11 | 0.2058968 | -1.0547255 | -1.3920468 |
| 17 | C | C11 | -0.0839051 | -2.5499123 | 0.0539730 |
| 18 | H | H12 | 0.1640927 | -2.9173582 | 1.0465816 |
| 19 | H | H16 | 2.0497456 | 3.7930719 | 4.9286282 |
| 20 | C | C14 | 0.2132182 | 1.6866706 | -0.5526569 |
| 21 | C | C15 | -1.7178629 | 2.9752724 | -2.1327952 |
| 22 | C | C16 | -1.1528020 | 1.5122024 | -0.2929265 |
| 23 | C | C17 | 0.6003696 | 2.5164918 | -1.6128682 |
| 24 | C | C18 | -0.3601727 | 3.1563232 | -2.3974298 |
| 25 | C | C19 | -2.1121947 | 2.1501502 | -1.0775961 |
| 26 | H | H2 | -1.4740149 | 0.8847489 | 0.5348762 |
| 27 | H | H1 | 1.6540170 | 2.6825218 | -1.8205484 |
| 28 | H | H3 | -0.0491263 | 3.8026883 | -3.2139870 |
| 29 | H | H10 | -3.1683130 | 2.0104767 | -0.8629331 |
| 30 | H | H15 | -2.4648964 | 3.4766167 | -2.7418699 |
| 31 | H | H17 | 3.3513352 | 0.3428039 | 0.2164551 |
| 32 | H | H18 | 2.4593056 | 0.2393659 | -1.3395042 |
| 33 | C | C12 | -0.9860671 | -3.3988915 | -0.7362677 |
| 34 | C | C13 | -2.7262649 | -5.0860924 | -2.1560505 |
| 35 | C | C20 | -1.9629416 | -2.8637967 | -1.5869450 |
| 36 | C | C21 | -0.9080837 | -4.7900142 | -0.5908611 |
| 37 | C | C22 | -1.7697516 | -5.6296747 | -1.3013609 |
| 38 | C | C23 | -2.8248758 | -3.7035835 | -2.2971076 |
| 39 | H | H5 | -2.0707841 | -1.7874104 | -1.6994879 |
| 40 | H | H13 | -0.1679214 | -5.2335634 | 0.0711705 |
| 41 | H | H14 | -1.6932297 | -6.7074940 | -1.1856945 |
| 42 | H | H19 | -3.5754602 | -3.2756175 | -2.9563243 |
| 43 | H | H20 | -3.3970656 | -5.7386774 | -2.7082956 |

## Sustmann VCPR TS

Gaussian'09: B3LYP/6-31G*, G = -888.098339, iv = i201, S^2 = 0.6849

Cartesian Coordinates (Angstroms)

| Atom | | | X | Y | Z |
|---|---|---|---|---|---|
| 1 | C | C | -0.0269135 | 3.0322724 | 0.3651644 |
| 2 | C | C2 | -1.2103563 | 2.3679144 | 0.2763010 |
| 3 | C | C3 | -1.3598391 | 1.0609182 | 0.8066873 |
| 4 | C | C4 | 1.1643584 | 2.2883003 | 0.8799909 |
| 5 | C | C5 | 1.2656103 | 0.8437567 | 0.3874205 |
| 6 | H | H | 0.1194959 | 4.0502385 | 0.0037539 |
| 7 | H | H7 | -2.0512600 | 2.8004308 | -0.2622636 |
| 8 | H | H8 | -0.8618118 | 0.7599148 | 1.7159615 |
| 9 | C | C1 | 1.8909915 | -0.1792479 | 1.2367267 |
| 10 | C | C6 | 3.1268072 | -2.1941215 | 2.8018338 |

| | | | | | |
|---|---|---|---|---|---|
| 11 | C | C7 | 2.8555473 | -1.0583675 | 0.6862312 |
| 12 | C | C8 | 1.5786590 | -0.3413779 | 2.6086037 |
| 13 | C | C9 | 2.1831999 | -1.3353085 | 3.3704229 |
| 14 | C | C10 | 3.4634290 | -2.0451617 | 1.4550234 |
| 15 | H | H2 | 3.2210392 | -0.9799177 | -0.3384494 |
| 16 | H | H6 | 0.8868435 | 0.2999722 | 3.1447167 |
| 17 | H | H5 | 1.9531260 | -1.4530058 | 4.4328034 |
| 18 | H | H3 | 4.2224089 | -2.7040790 | 1.0248655 |
| 19 | H | H4 | 3.6127696 | -2.9629314 | 3.4072836 |
| 20 | C | C13 | 1.1695655 | 0.6288107 | -1.0564075 |
| 21 | C | C14 | 0.8724646 | 0.1772572 | -3.8342089 |
| 22 | C | C15 | 1.3872637 | 1.6643669 | -1.9941773 |
| 23 | C | C16 | 0.8072404 | -0.6456949 | -1.5612241 |
| 24 | C | C17 | 0.6575959 | -0.8646435 | -2.9225151 |
| 25 | C | C18 | 1.2380126 | 1.4360556 | -3.3620031 |
| 26 | H | H11 | 1.8179670 | 2.6327972 | -1.7364651 |
| 27 | H | H12 | 0.6492528 | -1.5164453 | -0.9209106 |
| 28 | H | H13 | 0.4119018 | -1.8580422 | -3.3112913 |
| 29 | H | H14 | 1.4735600 | 2.2149849 | -4.0950196 |
| 30 | H | H15 | 0.7975208 | -0.0117358 | -4.9092232 |
| 31 | H | H16 | 1.1167981 | 2.3143977 | 1.9749587 |
| 32 | H | H17 | 2.0788858 | 2.8248769 | 0.5980375 |
| 33 | C | C11 | -2.5255896 | 0.0640594 | 0.3526260 |
| 34 | C | C12 | -4.4846969 | -1.7852412 | -0.3841672 |
| 35 | C | C19 | -3.3685840 | 0.3320269 | -0.7365100 |
| 36 | C | C20 | -2.6731429 | -1.1355713 | 1.0686093 |
| 37 | C | C21 | -3.6530577 | -2.0555931 | 0.6977398 |
| 38 | C | C22 | -4.3461298 | -0.5980304 | -1.0985063 |
| 39 | H | H1 | -3.3329778 | 1.2302020 | -1.3474036 |
| 40 | H | H9 | -2.0583888 | -1.3923795 | 1.9312546 |
| 41 | H | H10 | -3.7896071 | -2.9892860 | 1.2506341 |
| 42 | H | H18 | -5.0219184 | -0.4118401 | -1.9386429 |
| 43 | H | H19 | -5.2580407 | -2.5055313 | -0.6682614 |

## Sustmann [3,3] TS

Gaussian'09: B3LYP/6-31G*, G = -888.090641, iv = i385

Cartesian Coordinates (Angstroms)

| | | Atom | X | Y | Z |
|---|---|---|---|---|---|
| 1 | C | C1 | -1.2158865 | -0.8522870 | 0.6734974 |
| 2 | H | H3 | -1.3659523 | -1.9166506 | 0.5611198 |
| 3 | C | C2 | 0.1150097 | -0.4688105 | 1.1492486 |
| 4 | C | C5 | 0.0978896 | -0.3876310 | -1.8956387 |
| 5 | H | H7 | 0.1774585 | 0.5334981 | -2.4694213 |
| 6 | C | C6 | -1.2103261 | -0.7329575 | -1.4271119 |
| 7 | H | H6 | -1.4932838 | -1.7755982 | -1.4772134 |
| 8 | C | C7 | 0.2240385 | 0.6918035 | 1.9983036 |
| 9 | H | H4 | 1.2171496 | 1.0043791 | 2.3117403 |
| 10 | C | C8 | -0.8770135 | 1.3972592 | 2.3822541 |
| 11 | H | H9 | -0.7685821 | 2.2777066 | 3.0096368 |
| 12 | C | C9 | -2.1886048 | 0.9813351 | 1.9764615 |
| 13 | H | H11 | -3.0535724 | 1.5319291 | 2.3363479 |
| 14 | C | C10 | -2.3519991 | -0.1175903 | 1.1885447 |

```
 15 H   H12          -3.3447091    -0.4637382     0.9187841
 16 C   C3            1.2469468    -1.0393722    -1.5551573
 17 C   C4            1.2607021    -1.1104314     0.7250007
 18 C   C11           1.3457780    -2.0311061    -0.4447914
 19 H   H2            2.2062929    -0.6862884    -1.9217102
 20 H   H13           0.5763900    -2.8114049    -0.4645882
 21 H   H14           2.3145953    -2.5432517    -0.4625649
 22 C   C14           2.6763274    -0.7228923     1.2349276
 23 C   C15           5.2516079    -0.0650837     2.1853548
 24 C   C16           3.2742115    -1.4487114     2.2797424
 25 C   C17           3.4072101     0.3442956     0.6815639
 26 C   C18           4.6813961     0.6713438     1.1505058
 27 C   C19           4.5482476    -1.1250240     2.7515672
 28 H   H10           2.7323243    -2.2771313     2.7313969
 29 H   H15           2.9668480     0.9359623    -0.1181157
 30 H   H16           5.2249259     1.5026153     0.7094284
 31 H   H17           4.9883312    -1.7004903     3.5615061
 32 H   H18           6.2423913     0.1887231     2.5524460
 33 C   C12          -2.3385948     0.1661957    -1.9295166
 34 C   C13          -4.4027784     1.7976193    -2.8176402
 35 C   C20          -2.2398596     1.5472552    -1.7738149
 36 C   C21          -3.4616944    -0.4095593    -2.5177089
 37 C   C22          -4.4965870     0.4116445    -2.9657211
 38 C   C23          -3.2759898     2.3659111    -2.2200140
 39 H   H1           -1.3679619     1.9698680    -1.2846959
 40 H   H5           -3.5283929    -1.4856102    -2.6323173
 41 H   H8           -5.3756705    -0.0270590    -3.4301205
 42 H   H19          -3.2076139     3.4431710    -2.0960297
 43 H   H20          -5.2109992     2.4361639    -3.1654863
```

## Ichikawa VCP INT (52)

Gaussian'09: B3LYP/6-31G*, G = -1108.506583, iv = none

```
Cartesian Coordinates (Angstroms)
       Atom             X             Y             Z
   ---------    ------------- ------------- -------------

  1 C   C1            2.2910281     2.0104097    -1.1886598
  2 C   C2            1.1414659     2.7184398    -0.6052825
  3 C   C3            1.2667689     1.1588629    -0.5050026
  4 C   C10           0.3642447     0.3076408    -1.2924739
  5 H   H11           0.0800168     0.6873774    -2.2712185
  6 C   C11          -0.1509008    -0.8557149    -0.8695280
  7 H   H12           0.1025203    -1.2572522     0.1062422
  8 C   C4           -1.0821648    -1.6724113    -1.6593521
  9 C   C5           -2.8682629    -3.3006439    -3.0877385
 10 C   C6           -0.9100064    -1.8836438    -3.0334648
 11 C   C7           -2.1480908    -2.3047403    -1.0065605
 12 C   C8           -3.0403695    -3.1103883    -1.7182796
 13 C   C9           -1.8020244    -2.6901298    -3.7449847
 14 H   H3           -0.0731336    -1.4334427    -3.5615838
 15 H   H4           -2.2975245    -2.1679229     0.0620295
 16 H   H5           -3.8678956    -3.5900222    -1.2022587
 17 H   H7           -1.6594109    -2.8458606    -4.8111041
 18 H   H8           -3.5610375    -3.9289132    -3.6413769
```

```
 19 H   H9             1.3255102     3.3309280     0.2670203
 20 H   H10            0.3916141     3.1226335    -1.2753837
 21 O   O1             1.7880427     0.6696859     0.7730615
 22 Si  Si1            0.9911971     0.9088461     2.2335392
 23 C   C12            1.0619693    -0.6762027     3.2549177
 24 H   H13            2.1006089    -0.9713172     3.4299155
 25 H   H15            0.5540851    -1.4914534     2.7316314
 26 H   H16            0.5751629    -0.5302304     4.2235639
 27 C   C13           -0.8176967     1.3790454     2.0350855
 28 H   H6            -1.2795211     1.5279189     3.0159549
 29 H   H17           -1.3734971     0.5922526     1.5178984
 30 H   H18           -0.9244139     2.3079912     1.4682959
 31 C   C14            1.8461939     2.2653271     3.2230294
 32 H   H14            2.8988511     2.0148413     3.3836261
 33 H   H19            1.3676965     2.3868878     4.1992625
 34 H   H20            1.7962275     3.2212932     2.6945525
 35 F   F1             3.5239867     2.1531621    -0.5786402
 36 F   F2             2.3887598     1.9467462    -2.5667325
```

## Ichikawa VCPR TS

Gaussian'09: B3LYP/6-31G* using scf=(qcverytight) and guess=(mix,always,densitymix) in input line, G = -1108.460735, iv = i109, S^2 = 0.79

```
Cartesian Coordinates (Angstroms)
       Atom             X             Y             Z
    ---------    ------------- ------------- -------------

  1 C   C              1.9137391     1.1672811     0.4623618
  2 C   C2             0.7982840     0.3787391     0.4386759
  3 C   C3             0.7719369    -0.8931799     1.0544091
  4 C   C4             3.1968266     0.5748901     0.9540772
  5 C   C5             3.4120343    -0.8159359     0.4684891
  6 H   H              1.9254320     2.1927805     0.1088231
  7 H   H8             1.4043016    -1.1230086     1.9018355
  8 H   H12            3.0581443    -1.0949354    -0.5151967
  9 H   H4             4.2350300    -1.3872942     0.9128354
 10 O   O3            -0.3890101     0.7109659    -0.1542352
 11 Si  Si1           -0.7786309     2.1508656    -0.9389403
 12 C   C1            -0.6701825     3.5884769     0.2476550
 13 H   H7            -1.3026780     3.4129458     1.1232011
 14 H   H11           -1.0122807     4.5094809    -0.2345161
 15 H   H13            0.3508105     3.7575846     0.5985019
 16 C   C6             0.3729825     2.4152865    -2.3902891
 17 H   H5             0.3692404     1.5399622    -3.0469016
 18 H   H14            1.4026811     2.5957345    -2.0723966
 19 H   H15            0.0509264     3.2797206    -2.9791621
 20 C   C7            -2.5316834     2.0200185    -1.5819238
 21 H   H6            -3.2343665     1.8577902    -0.7591750
 22 H   H16           -2.6260887     1.1830513    -2.2802412
 23 H   H17           -2.8233725     2.9357156    -2.1047400
 24 C   C8            -0.3347065    -2.0859988     0.8493571
 25 C   C9            -2.1173226    -4.2323675     0.6236915
 26 C   C10           -1.3177024    -2.0818909    -0.1502703
 27 C   C11           -0.2485493    -3.1829850     1.7262542
```

```
  28  C   C12          -1.1408365    -4.2489898     1.6126115
  29  C   C13          -2.2062767    -3.1567098    -0.2549064
  30  H   H1           -1.4565368    -1.3088833    -0.8971375
  31  H   H9            0.4835660    -3.2459843     2.5313231
  32  H   H10          -1.0931269    -5.1014248     2.2961903
  33  H   H18          -2.9836290    -3.1763755    -1.0247698
  34  H   H19          -2.8191322    -5.0675515     0.5381456
  35  F   F1            4.2306300     1.3778540     0.6270610
  36  F   F2            3.1095464     0.5543711     2.3093024
```

**Hammett Plot**

## p_NMe2_SM (S1a)

```
G = -986.405584
iv = none

Cartesian Coordinates (Angstroms)
       Atom              X             Y             Z
    ---------    ------------- ------------- -------------

   1  C   C1            1.5584825     0.1025224    -2.3886327
   2  C   C2            0.4103350     0.6021148    -1.6087786
   3  C   C3            0.6679862    -0.9553396    -1.8292907
   4  H   H3           -0.3986704     0.9730780    -2.2377576
   5  H   H6            1.0586619    -1.4515965    -0.9442274
   6  C   C4            0.5001002     1.1869267    -0.2380093
   7  C   C5            0.5732065     2.3190323     2.3824332
   8  C   C6           -0.5615688     1.0151570     0.6614430
   9  C   C7            1.6000107     1.9356895     0.1982475
  10  C   C8            1.6435293     2.4858832     1.4755522
  11  C   C9           -0.5345924     1.5585553     1.9391770
  12  H   H4           -1.4287926     0.4329341     0.3574701
  13  H   H7            2.4449454     2.0864268    -0.4648848
  14  H   H8            2.5221955     3.0502945     1.7635900
  15  H   H9           -1.3797019     1.3873351     2.5950487
  16  C   C10          -0.2340745    -1.7296817    -2.6860464
  17  H   H11          -0.5575400    -1.2745917    -3.6205935
  18  C   C11          -0.6941664    -2.9563111    -2.3925762
  19  H   H12          -0.4109535    -3.4788213    -1.4834960
  20  C   C12          -1.6147289    -3.6494885    -3.3153280
  21  O   O1           -2.0354075    -3.2116057    -4.3698009
  22  O   O2           -1.9500367    -4.8718127    -2.8302762
  23  C   C13          -2.8426018    -5.6310552    -3.6570646
  24  H   H5           -3.7770573    -5.0862991    -3.8173613
  25  H   H13          -3.0270436    -6.5595776    -3.1153547
  26  H   H14          -2.3854629    -5.8382771    -4.6289032
  27  F   F1            1.5632782     0.3054300    -3.7284662
  28  F   F2            2.8228242     0.2003465    -1.9124621
  29  N   N1            0.6028731     2.8829986     3.6510640
  30  C   C14           1.8395244     3.4710273     4.1367540
  31  H   H1            2.1484069     4.3141581     3.5067625
  32  H   H10           2.6710701     2.7489363     4.1695523
  33  H   H15           1.6805189     3.8558945     5.1459552
  34  C   C15          -0.4124265     2.5103263     4.6210870
  35  H   H2           -0.2419652     3.0633957     5.5468705
```

| | | | | | |
|---|---|---|---|---|---|
| 36 | H | H16 | -0.4061960 | 1.4336214 | 4.8563569 |
| 37 | H | H17 | -1.4149619 | 2.7723735 | 4.2619463 |

## NMe2_TS2 (S-TS1a)

G = -986.371766
iv = i75
S^2 = 0.4500

| Atom | | | X | Y | Z |
|---|---|---|---|---|---|
| 1 | C | C | -1.7257165 | 3.6608005 | -1.0708224 |
| 2 | C | C2 | -2.8870564 | 2.9448482 | -1.1483783 |
| 3 | C | C3 | -3.0524220 | 1.7261259 | -0.4506724 |
| 4 | C | C4 | -0.5565938 | 3.0194769 | -0.4159069 |
| 5 | C | C5 | -0.3836554 | 1.5979954 | -0.8610707 |
| 6 | H | H | -1.6022001 | 4.6557693 | -1.4838963 |
| 7 | H | H7 | -3.6991650 | 3.2728347 | -1.7948143 |
| 8 | H | H8 | -2.5315502 | 1.5364955 | 0.4779677 |
| 9 | H | H12 | -0.8743979 | 1.3410869 | -1.7900136 |
| 10 | F | F1 | 0.5982312 | 3.7432144 | -0.6825366 |
| 11 | F | F2 | -0.6580277 | 3.0804251 | 0.9893451 |
| 12 | C | C1 | 0.6067414 | 0.7159363 | -0.3745757 |
| 13 | C | C6 | 2.5419146 | -1.1846315 | 0.5589313 |
| 14 | C | C7 | 0.8259172 | -0.5246276 | -1.0369708 |
| 15 | C | C8 | 1.4021316 | 0.9650054 | 0.7809334 |
| 16 | C | C9 | 2.3374050 | 0.0555558 | 1.2252386 |
| 17 | C | C10 | 1.7498539 | -1.4449934 | -0.5932123 |
| 18 | H | H2 | 0.2369406 | -0.7532016 | -1.9219840 |
| 19 | H | H6 | 1.2651172 | 1.8922670 | 1.3235701 |
| 20 | H | H5 | 2.9141905 | 0.2991633 | 2.1095642 |
| 21 | H | H3 | 1.8633518 | -2.3748420 | -1.1375105 |
| 22 | C | C11 | -4.0928551 | 0.7828418 | -0.8453818 |
| 23 | O | O1 | -4.8594126 | 0.9119027 | -1.7888919 |
| 24 | O | O2 | -4.1147561 | -0.3020953 | -0.0188599 |
| 25 | C | C12 | -5.0906560 | -1.2947775 | -0.3485181 |
| 26 | H | H1 | -6.1040563 | -0.8931400 | -0.2514844 |
| 27 | H | H9 | -4.9354454 | -2.1081869 | 0.3623164 |
| 28 | H | H10 | -4.9537390 | -1.6503536 | -1.3741766 |
| 29 | N | N1 | 3.4629040 | -2.0964084 | 1.0116655 |
| 30 | C | C13 | 3.6322520 | -3.3693753 | 0.3261360 |
| 31 | H | H4 | 4.4017447 | -3.9509484 | 0.8352652 |
| 32 | H | H13 | 3.9474534 | -3.2280999 | -0.7162523 |
| 33 | H | H14 | 2.7040599 | -3.9568964 | 0.3266268 |
| 34 | C | C14 | 4.2471670 | -1.8172132 | 2.2061765 |
| 35 | H | H11 | 4.9151404 | -2.6574804 | 2.3998742 |
| 36 | H | H15 | 3.6075847 | -1.6789864 | 3.0885939 |
| 37 | H | H16 | 4.8616043 | -0.9154872 | 2.0837247 |

Cartesian Coordinates (Angstroms)

## pNH2_SM (S1b)

G = -907.846024
iv = none

Cartesian Coordinates (Angstroms)

| | Atom | | X | Y | Z |
|---|---|---|---|---|---|
| 1 | C | C1 | 1.6851610 | 0.1319418 | -1.5815304 |
| 2 | C | C2 | 0.6384108 | 0.9359312 | -0.9216993 |
| 3 | C | C3 | 0.6730599 | -0.6463225 | -0.8066263 |
| 4 | H | H3 | -0.1322477 | 1.2860880 | -1.6088658 |
| 5 | H | H6 | 1.0203339 | -0.9992078 | 0.1615377 |
| 6 | C | C4 | 0.8805428 | 1.7764293 | 0.2902526 |
| 7 | C | C5 | 1.2586871 | 3.4160653 | 2.5710386 |
| 8 | C | C6 | -0.0743025 | 1.8211479 | 1.3164424 |
| 9 | C | C7 | 2.0281729 | 2.5685850 | 0.4268111 |
| 10 | C | C8 | 2.2176801 | 3.3742094 | 1.5456268 |
| 11 | C | C9 | 0.1049242 | 2.6230367 | 2.4372474 |
| 12 | H | H4 | -0.9719102 | 1.2120028 | 1.2378336 |
| 13 | H | H7 | 2.7894641 | 2.5492299 | -0.3465061 |
| 14 | H | H8 | 3.1164852 | 3.9816222 | 1.6268973 |
| 15 | H | H9 | -0.6525406 | 2.6419044 | 3.2180763 |
| 16 | C | C10 | -0.3479446 | -1.4519089 | -1.4842468 |
| 17 | H | H11 | -0.6428478 | -1.1489419 | -2.4876272 |
| 18 | C | C11 | -0.9455504 | -2.5303317 | -0.9540096 |
| 19 | H | H12 | -0.7023801 | -2.9010621 | 0.0376269 |
| 20 | C | C12 | -1.9715553 | -3.2674690 | -1.7192850 |
| 21 | O | O1 | -2.3657116 | -2.9917879 | -2.8367407 |
| 22 | O | O2 | -2.4387865 | -4.3209899 | -1.0030180 |
| 23 | C | C13 | -3.4418486 | -5.1077992 | -1.6601821 |
| 24 | H | H5 | -4.2967329 | -4.4869973 | -1.9414600 |
| 25 | H | H13 | -3.7395441 | -5.8681781 | -0.9370796 |
| 26 | H | H14 | -3.0349725 | -5.5754868 | -2.5614979 |
| 27 | F | F1 | 1.6792664 | 0.0435328 | -2.9332927 |
| 28 | F | F2 | 2.9618784 | 0.1596004 | -1.1329688 |
| 29 | N | N1 | 1.4187425 | 4.2650383 | 3.6670814 |
| 30 | H | H2 | 0.9101470 | 3.9942771 | 4.4993782 |
| 31 | H | H1 | 2.3759190 | 4.5158404 | 3.8807859 |

## pNH2_TS2 (S-TS1b)

G = -907.811148
iv = i93
S^2 = 0.4444

Cartesian Coordinates (Angstroms)

| | Atom | | X | Y | Z |
|---|---|---|---|---|---|
| 1 | C | C | -1.0033632 | 3.1014530 | -0.7660046 |
| 2 | C | C2 | -2.1485900 | 2.3610330 | -0.8316443 |
| 3 | C | C3 | -2.2708494 | 1.1339810 | -0.1359989 |
| 4 | C | C4 | 0.1839831 | 2.4738482 | -0.1310559 |
| 5 | C | C5 | 0.3705269 | 1.0576570 | -0.5915524 |

| | | | | | |
|---|---|---|---|---|---|
| 6 | H | H | -0.9033214 | 4.1003023 | -1.1761130 |
| 7 | H | H7 | -2.9780636 | 2.6709029 | -1.4646857 |
| 8 | H | H8 | -1.7499928 | 0.9640771 | 0.7962412 |
| 9 | H | H12 | -0.1191565 | 0.8072066 | -1.5228758 |
| 10 | F | F1 | 1.3244981 | 3.2147033 | -0.4056469 |
| 11 | F | F2 | 0.0981061 | 2.5204954 | 1.2754407 |
| 12 | C | C1 | 1.3963136 | 0.1950264 | -0.1345531 |
| 13 | C | C6 | 3.4137602 | -1.6272165 | 0.7115376 |
| 14 | C | C7 | 1.6470497 | -1.0214553 | -0.8308863 |
| 15 | C | C8 | 2.2002934 | 0.4568797 | 1.0128273 |
| 16 | C | C9 | 3.1820342 | -0.4235911 | 1.4158791 |
| 17 | C | C10 | 2.6207714 | -1.9087996 | -0.4239561 |
| 18 | H | H2 | 1.0484108 | -1.2537662 | -1.7080921 |
| 19 | H | H6 | 2.0335083 | 1.3660344 | 1.5770487 |
| 20 | H | H5 | 3.7873072 | -0.1954740 | 2.2905431 |
| 21 | H | H3 | 2.7895711 | -2.8287797 | -0.9791327 |
| 22 | C | C11 | -3.2961996 | 0.1690414 | -0.5228418 |
| 23 | O | O1 | -4.0604403 | 0.2788837 | -1.4702046 |
| 24 | O | O2 | -3.3086894 | -0.9043195 | 0.3167099 |
| 25 | C | C12 | -4.2820913 | -1.9058321 | 0.0036247 |
| 26 | H | H1 | -5.2961855 | -1.5047171 | 0.0952275 |
| 27 | H | H9 | -4.1241115 | -2.7069217 | 0.7274931 |
| 28 | H | H10 | -4.1449068 | -2.2768805 | -1.0163953 |
| 29 | N | N1 | 4.4201155 | -2.4844004 | 1.0969438 |
| 30 | H | H4 | 4.7824353 | -2.3941872 | 2.0359887 |
| 31 | H | H11 | 4.3872762 | -3.4351844 | 0.7561341 |

## pOH_SM (S1c)

G = -927.720966
iv = none

Cartesian Coordinates (Angstroms)

| | Atom | | X | Y | Z |
|---|---|---|---|---|---|
| 1 | C | C1 | 1.7176635 | 0.7049715 | -1.2705558 |
| 2 | C | C2 | 0.5244052 | 1.2429854 | -0.5888208 |
| 3 | C | C3 | 0.8191476 | -0.3143685 | -0.6521193 |
| 4 | H | H3 | -0.2658900 | 1.5445537 | -1.2763492 |
| 5 | H | H6 | 1.1813468 | -0.7248364 | 0.2875717 |
| 6 | C | C4 | 0.5664812 | 1.9608276 | 0.7219435 |
| 7 | C | C5 | 0.5510370 | 3.3453453 | 3.1697163 |
| 8 | C | C6 | -0.4413795 | 1.7357997 | 1.6732258 |
| 9 | C | C7 | 1.5661187 | 2.8935809 | 1.0212313 |
| 10 | C | C8 | 1.5625457 | 3.5815466 | 2.2345678 |
| 11 | C | C9 | -0.4569616 | 2.4159899 | 2.8847103 |
| 12 | H | H4 | -1.2238110 | 1.0124494 | 1.4576288 |
| 13 | H | H7 | 2.3594454 | 3.0816508 | 0.3057250 |
| 14 | H | H8 | 2.3489012 | 4.3021003 | 2.4524849 |
| 15 | H | H9 | -1.2354174 | 2.2422225 | 3.6207104 |
| 16 | C | C10 | -0.0256227 | -1.1835437 | -1.4787692 |
| 17 | H | H11 | -0.3308351 | -0.8084850 | -2.4543328 |
| 18 | C | C11 | -0.4506697 | -2.4033908 | -1.1162162 |
| 19 | H | H12 | -0.1849613 | -2.8529013 | -0.1637986 |
| 20 | C | C12 | -1.3099918 | -3.1921371 | -2.0232351 |

| | | | | | |
|---|---|---|---|---|---|
| 21 | O | O1 | -1.7058173 | -2.8384995 | -3.1178977 |
| 22 | O | O2 | -1.6165460 | -4.3919981 | -1.4708236 |
| 23 | C | C13 | -2.4504622 | -5.2374544 | -2.2758548 |
| 24 | H | H5 | -3.4026611 | -4.7454437 | -2.4925444 |
| 25 | H | H13 | -2.6102318 | -6.1404204 | -1.6854493 |
| 26 | H | H14 | -1.9556246 | -5.4785865 | -3.2207919 |
| 27 | F | F1 | 1.7827542 | 0.7845676 | -2.6206418 |
| 28 | F | F2 | 2.9518510 | 0.8833571 | -0.7438053 |
| 29 | O | O3 | 0.4915019 | 3.9861025 | 4.3747873 |
| 30 | H | H15 | 1.2436836 | 4.5940145 | 4.4477026 |

## pOH_TS2 (S-TS1c)

G = -927.683551
iv = i104
S^2 = 0.5058

Cartesian Coordinates (Angstroms)

| | Atom | | X | Y | Z |
|---|---|---|---|---|---|
| 1 | C | C | -0.8343384 | 3.0005875 | -0.7407077 |
| 2 | C | C2 | -1.9829047 | 2.2662349 | -0.8020632 |
| 3 | C | C3 | -2.1042135 | 1.0372641 | -0.1082562 |
| 4 | C | C4 | 0.3542186 | 2.3643336 | -0.1116000 |
| 5 | C | C5 | 0.5256601 | 0.9448303 | -0.5683545 |
| 6 | H | H | -0.7301742 | 3.9984166 | -1.1522788 |
| 7 | H | H7 | -2.8147603 | 2.5799453 | -1.4296835 |
| 8 | H | H8 | -1.5836454 | 0.8652785 | 0.8238757 |
| 9 | H | H12 | 0.0492697 | 0.7031459 | -1.5090688 |
| 10 | F | F1 | 1.4973858 | 3.0949055 | -0.3957734 |
| 11 | F | F2 | 0.2712326 | 2.4172782 | 1.2927841 |
| 12 | C | C1 | 1.5376800 | 0.0644986 | -0.0982657 |
| 13 | C | C6 | 3.5070141 | -1.7720166 | 0.7576334 |
| 14 | C | C7 | 1.7747574 | -1.1543337 | -0.7946901 |
| 15 | C | C8 | 2.3301349 | 0.3200217 | 1.0546892 |
| 16 | C | C9 | 3.2991461 | -0.5778997 | 1.4669917 |
| 17 | C | C10 | 2.7337107 | -2.0572561 | -0.3797899 |
| 18 | H | H2 | 1.1816258 | -1.3754413 | -1.6783337 |
| 19 | H | H6 | 2.1723372 | 1.2324451 | 1.6157909 |
| 20 | H | H5 | 3.8997598 | -0.3626211 | 2.3492904 |
| 21 | H | H3 | 2.9095243 | -2.9851554 | -0.9146080 |
| 22 | C | C11 | -3.1320862 | 0.0717056 | -0.4974130 |
| 23 | O | O1 | -3.8953467 | 0.1895222 | -1.4437008 |
| 24 | O | O2 | -3.1415021 | -1.0023819 | 0.3367300 |
| 25 | C | C12 | -4.1134268 | -2.0068523 | 0.0224721 |
| 26 | H | H1 | -5.1277049 | -1.6073436 | 0.1168668 |
| 27 | H | H9 | -3.9514965 | -2.8086909 | 0.7442256 |
| 28 | H | H10 | -3.9754656 | -2.3741969 | -0.9985851 |
| 29 | O | O3 | 4.4384058 | -2.6900073 | 1.1254616 |
| 30 | H | H4 | 4.9052025 | -2.3762166 | 1.9163605 |

## pOMe_SM (S1d)

G = -966.999935
iv = none

Cartesian Coordinates (Angstroms)

| | Atom | | X | Y | Z |
|---|---|---|---|---|---|
| 1 | C | C1 | 1.5684705 | 0.2206178 | -1.7265240 |
| 2 | C | C2 | 0.3767325 | 0.7586473 | -1.0428449 |
| 3 | C | C3 | 0.6711354 | -0.7986142 | -1.1054805 |
| 4 | H | H3 | -0.4146782 | 1.0603301 | -1.7291412 |
| 5 | H | H6 | 1.0361460 | -1.2083128 | -0.1665828 |
| 6 | C | C4 | 0.4192798 | 1.4751382 | 0.2683520 |
| 7 | C | C5 | 0.3995578 | 2.8646981 | 2.7183402 |
| 8 | C | C6 | -0.5826736 | 1.2374397 | 1.2250323 |
| 9 | C | C7 | 1.4069566 | 2.4176329 | 0.5652301 |
| 10 | C | C8 | 1.4062191 | 3.1116747 | 1.7788282 |
| 11 | C | C9 | -0.5978226 | 1.9188432 | 2.4327813 |
| 12 | H | H4 | -1.3580613 | 0.5051265 | 1.0139794 |
| 13 | H | H7 | 2.1951714 | 2.6143151 | -0.1540665 |
| 14 | H | H8 | 2.1883617 | 3.8356514 | 1.9764159 |
| 15 | H | H9 | -1.3698877 | 1.7390233 | 3.1742316 |
| 16 | C | C10 | -0.1777455 | -1.6675893 | -1.9280107 |
| 17 | H | H11 | -0.4818819 | -1.2951250 | -2.9049402 |
| 18 | C | C11 | -0.6097880 | -2.8832318 | -1.5593780 |
| 19 | H | H12 | -0.3457029 | -3.3298459 | -0.6051592 |
| 20 | C | C12 | -1.4759922 | -3.6705031 | -2.4608967 |
| 21 | O | O1 | -1.8727548 | -3.3192847 | -3.5560320 |
| 22 | O | O2 | -1.7885307 | -4.8663163 | -1.9026053 |
| 23 | C | C13 | -2.6297513 | -5.7098323 | -2.7019138 |
| 24 | H | H5 | -3.5815062 | -5.2147909 | -2.9137000 |
| 25 | H | H13 | -2.7893392 | -6.6115801 | -2.1095754 |
| 26 | H | H14 | -2.1413615 | -5.9538428 | -3.6494661 |
| 27 | F | F1 | 1.6301616 | 0.2985041 | -3.0770919 |
| 28 | F | F2 | 2.8034684 | 0.4007019 | -1.2029834 |
| 29 | O | O3 | 0.2963876 | 3.4834113 | 3.9292099 |
| 30 | C | C14 | 1.2722343 | 4.4550219 | 4.2726559 |
| 31 | H | H2 | 2.2778950 | 4.0167761 | 4.3222211 |
| 32 | H | H1 | 0.9909234 | 4.8256908 | 5.2598928 |
| 33 | H | H10 | 1.2783764 | 5.2896248 | 3.5592219 |

## pOMe_TS2 (S-TS1d)

G = -966.962804
iv = i120
S^2 = 0.4627

Cartesian Coordinates (Angstroms)

| | Atom | | X | Y | Z |
|---|---|---|---|---|---|
| 1 | C | C | -1.2853765 | -0.9843654 | -3.2298744 |
| 2 | C | C2 | -2.4478855 | -1.0570847 | -2.5210862 |
| 3 | C | C3 | -2.5969876 | -0.3738806 | -1.2876998 |
| 4 | C | C4 | -0.1215990 | -0.3415462 | -2.5660629 |
| 5 | C | C5 | 0.0152474 | -0.7873108 | -1.1388865 |
| 6 | H | H | -1.1548866 | -1.3895044 | -4.2272081 |
| 7 | H | H7 | -3.2700595 | -1.6867161 | -2.8552197 |
| 8 | H | H8 | -2.0992402 | 0.5680364 | -1.1019723 |
| 9 | H | H12 | -0.4471057 | -1.7370223 | -0.9060560 |
| 10 | F | F1 | 1.0444644 | -0.6188252 | -3.2626443 |
| 11 | F | F2 | -0.2163996 | 1.0621237 | -2.6311444 |
| 12 | C | C1 | 1.0108366 | -0.3040677 | -0.2465697 |
| 13 | C | C6 | 2.9925752 | 0.5632996 | 1.5807666 |
| 14 | C | C7 | 1.2470701 | -0.9924939 | 0.9779465 |
| 15 | C | C8 | 1.7997017 | 0.8480714 | -0.5034394 |
| 16 | C | C9 | 2.7768615 | 1.2693620 | 0.3848445 |
| 17 | C | C10 | 2.2087428 | -0.5715467 | 1.8713489 |
| 18 | H | H2 | 0.6540083 | -1.8744149 | 1.2063193 |
| 19 | H | H6 | 1.6379786 | 1.4053597 | -1.4172121 |
| 20 | H | H5 | 3.3622031 | 2.1496247 | 0.1452903 |
| 21 | H | H3 | 2.3890886 | -1.0999616 | 2.8021740 |
| 22 | C | C11 | -3.6490779 | -0.7776127 | -0.3544587 |
| 23 | O | O1 | -4.4312068 | -1.7000828 | -0.5247839 |
| 24 | O | O2 | -3.6601480 | 0.0151239 | 0.7508289 |
| 25 | C | C12 | -4.6736312 | -0.2900381 | 1.7162525 |
| 26 | H | H1 | -5.6501013 | 0.0616953 | 1.3679974 |
| 27 | H | H9 | -4.3875978 | 0.2408311 | 2.6251722 |
| 28 | H | H10 | -4.7307654 | -1.3660361 | 1.8947585 |
| 29 | O | O3 | 3.9193253 | 0.8926177 | 2.5124782 |
| 30 | C | C13 | 4.7696399 | 2.0076929 | 2.2673506 |
| 31 | H | H4 | 5.4324808 | 2.0698038 | 3.1316097 |
| 32 | H | H11 | 4.1950788 | 2.9381197 | 2.1803286 |
| 33 | H | H13 | 5.3667660 | 1.8607484 | 1.3588516 |

## pNHCOMe_SM (S1e)

G = -1060.47329
iv = none

Cartesian Coordinates (Angstroms)

| | Atom | | X | Y | Z |
|---|---|---|---|---|---|
| 1 | C | C1 | 1.1713988 | 0.1575743 | -2.5063816 |
| 2 | C | C2 | 0.0746468 | 0.5360569 | -1.5940816 |
| 3 | C | C3 | 0.4551586 | -0.9803119 | -1.8554379 |

| | | | | | |
|---|---|---|---|---|---|
| 4 | H | H3 | -0.8364244 | 0.8316940 | -2.1138577 |
| 5 | H | H6 | 0.9977653 | -1.4393910 | -1.0322157 |
| 6 | C | C4 | 0.2848449 | 1.1340345 | -0.2398241 |
| 7 | C | C5 | 0.5967967 | 2.2851907 | 2.3182726 |
| 8 | C | C6 | -0.5347055 | 0.7412212 | 0.8278222 |
| 9 | C | C7 | 1.2582649 | 2.1111000 | -0.0000613 |
| 10 | C | C8 | 1.4233990 | 2.6872720 | 1.2588706 |
| 11 | C | C9 | -0.3842648 | 1.3064663 | 2.0872504 |
| 12 | H | H4 | -1.2963958 | -0.0181440 | 0.6698103 |
| 13 | H | H7 | 1.9085981 | 2.4281561 | -0.8084713 |
| 14 | H | H8 | 2.1795727 | 3.4397135 | 1.4311937 |
| 15 | H | H9 | -1.0315881 | 0.9864734 | 2.9017992 |
| 16 | C | C10 | -0.4605186 | -1.8381581 | -2.6155977 |
| 17 | H | H11 | -0.9621526 | -1.3995086 | -3.4768142 |
| 18 | C | C11 | -0.7271914 | -3.1200015 | -2.3225197 |
| 19 | H | H12 | -0.2622053 | -3.6350699 | -1.4868079 |
| 20 | C | C12 | -1.6764426 | -3.8919940 | -3.1511922 |
| 21 | O | O1 | -2.2963893 | -3.4692799 | -4.1086108 |
| 22 | O | O2 | -1.7767426 | -5.1674604 | -2.7017280 |
| 23 | C | C13 | -2.6724119 | -6.0064804 | -3.4449387 |
| 24 | H | H5 | -3.6915473 | -5.6111880 | -3.4077610 |
| 25 | H | H13 | -2.6236443 | -6.9849591 | -2.9658000 |
| 26 | H | H14 | -2.3589465 | -6.0737342 | -4.4903650 |
| 27 | F | F1 | 1.0067983 | 0.3562810 | -3.8353220 |
| 28 | F | F2 | 2.4596795 | 0.3884930 | -2.1653710 |
| 29 | N | N1 | 0.6944204 | 2.8088096 | 3.6232082 |
| 30 | H | H2 | 0.0386330 | 2.4191320 | 4.2864124 |
| 31 | C | C14 | 1.5413221 | 3.7860268 | 4.1073753 |
| 32 | C | C15 | 1.3137490 | 4.1357926 | 5.5722271 |
| 33 | H | H10 | 2.2809986 | 4.3566528 | 6.0286279 |
| 34 | H | H15 | 0.8180166 | 3.3435332 | 6.1430953 |
| 35 | H | H16 | 0.7007428 | 5.0424378 | 5.6345030 |
| 36 | O | O3 | 2.3867645 | 4.3535694 | 3.4326912 |

## pNHCOMe_TS2 (S-TS1e)

G = -1060.43552
iv = i111
S^2 = 0.5197

Cartesian Coordinates (Angstroms)

| | | Atom | X | Y | Z |
|---|---|---|---|---|---|
| 1 | C | C | -1.6566376 | 3.6562397 | -1.3429537 |
| 2 | C | C2 | -2.8091997 | 2.9353394 | -1.4526862 |
| 3 | C | C3 | -2.9883888 | 1.7254383 | -0.7364660 |
| 4 | C | C4 | -0.5135962 | 3.0231753 | -0.6280485 |
| 5 | C | C5 | -0.3433739 | 1.5860810 | -1.0289568 |
| 6 | H | H | -1.5148126 | 4.6412060 | -1.7738071 |
| 7 | H | H7 | -3.5988899 | 3.2442342 | -2.1348052 |
| 8 | H | H8 | -2.5298791 | 1.5721052 | 0.2307956 |
| 9 | H | H12 | -0.7556016 | 1.3301491 | -1.9960268 |
| 10 | F | F1 | 0.6542942 | 3.7247365 | -0.8777820 |
| 11 | F | F2 | -0.6747975 | 3.1267197 | 0.7632907 |
| 12 | C | C1 | 0.6022874 | 0.6913855 | -0.4611539 |

| | | | | | |
|---|---|---|---|---|---|
| 13 | C | C6 | 2.4405540 | -1.1914785 | 0.6027409 |
| 14 | C | C7 | 0.8445679 | -0.5530769 | -1.1050284 |
| 15 | C | C8 | 1.3194300 | 0.9470487 | 0.7398929 |
| 16 | C | C9 | 2.2230487 | 0.0367430 | 1.2593855 |
| 17 | C | C10 | 1.7360915 | -1.4694016 | -0.5875130 |
| 18 | H | H2 | 0.3128024 | -0.7833655 | -2.0245305 |
| 19 | H | H6 | 1.1551133 | 1.8812405 | 1.2618904 |
| 20 | H | H5 | 2.7621316 | 0.2510878 | 2.1710956 |
| 21 | H | H3 | 1.8992264 | -2.4146737 | -1.1015990 |
| 22 | C | C11 | -4.0012680 | 0.7610459 | -1.1655510 |
| 23 | O | O1 | -4.6951792 | 0.8583805 | -2.1659369 |
| 24 | O | O2 | -4.0858484 | -0.2853448 | -0.3010219 |
| 25 | C | C12 | -5.0537372 | -1.2823288 | -0.6490319 |
| 26 | H | H1 | -6.0577532 | -0.8501166 | -0.6890899 |
| 27 | H | H9 | -4.9935350 | -2.0375100 | 0.1359261 |
| 28 | H | H10 | -4.8235019 | -1.7226931 | -1.6237780 |
| 29 | N | N1 | 3.3253262 | -2.1722419 | 1.0681610 |
| 30 | H | H11 | 3.3779714 | -3.0037897 | 0.4949480 |
| 31 | C | C13 | 4.1130617 | -2.1734547 | 2.2098966 |
| 32 | O | O3 | 4.1640704 | -1.2460722 | 3.0004609 |
| 33 | C | C14 | 4.8948472 | -3.4621882 | 2.4184878 |
| 34 | H | H4 | 5.0617664 | -4.0281969 | 1.4960790 |
| 35 | H | H13 | 4.3512309 | -4.1022614 | 3.1235117 |
| 36 | H | H14 | 5.8581785 | -3.2141621 | 2.8692043 |

## pMe_SM (S1f)

G = -891.799548
iv = none

Cartesian Coordinates (Angstroms)

| | | Atom | X | Y | Z |
|---|---|---|---|---|---|
| 1 | C | C1 | 1.3804811 | 0.7111935 | -1.7861492 |
| 2 | C | C2 | 0.2866776 | 1.1001233 | -0.8747163 |
| 3 | C | C3 | 0.6545210 | -0.4190597 | -1.1326506 |
| 4 | H | H3 | -0.6218868 | 1.4031422 | -1.3943647 |
| 5 | H | H6 | 1.1922568 | -0.8807027 | -0.3076510 |
| 6 | C | C4 | 0.4999533 | 1.7001255 | 0.4795990 |
| 7 | C | C5 | 0.8180760 | 2.8514766 | 3.0485519 |
| 8 | C | C6 | -0.3228548 | 1.3150779 | 1.5462997 |
| 9 | C | C7 | 1.4852563 | 2.6683170 | 0.7125421 |
| 10 | C | C8 | 1.6392027 | 3.2305764 | 1.9790346 |
| 11 | C | C9 | -0.1651259 | 1.8825139 | 2.8089096 |
| 12 | H | H4 | -1.0877670 | 0.5590569 | 1.3853688 |
| 13 | H | H7 | 2.1401209 | 2.9749506 | -0.0963091 |
| 14 | H | H8 | 2.4142963 | 3.9768668 | 2.1390680 |
| 15 | H | H9 | -0.8130135 | 1.5654741 | 3.6230621 |
| 16 | C | C10 | -0.2649214 | -1.2725938 | -1.8936981 |
| 17 | H | H11 | -0.7636714 | -0.8315028 | -2.7553215 |
| 18 | C | C11 | -0.5361347 | -2.5538572 | -1.6025425 |
| 19 | H | H12 | -0.0747847 | -3.0711972 | -0.7662466 |
| 20 | C | C12 | -1.4852845 | -3.3226127 | -2.4346723 |
| 21 | O | O1 | -2.1001038 | -2.8977963 | -3.3944603 |
| 22 | O | O2 | -1.5911644 | -4.5978163 | -1.9858790 |

| | | | | | |
|---|---|---|---|---|---|
| 23 | C | C13 | -2.4873711 | -5.4339559 | -2.7316450 |
| 24 | H | H5 | -3.5055214 | -5.0358820 | -2.6967921 |
| 25 | H | H13 | -2.4426931 | -6.4127267 | -2.2526976 |
| 26 | H | H14 | -2.1715024 | -5.5017247 | -3.7763096 |
| 27 | F | F1 | 1.2180697 | 0.9082906 | -3.1158431 |
| 28 | F | F2 | 2.6714102 | 0.9320561 | -1.4462235 |
| 29 | C | C14 | 0.9777194 | 3.4838681 | 4.4113072 |
| 30 | H | H2 | 2.0336375 | 3.6363601 | 4.6612334 |
| 31 | H | H1 | 0.5308679 | 2.8644512 | 5.1956799 |
| 32 | H | H10 | 0.4912538 | 4.4675073 | 4.4535157 |

## pMe_TS2 (S-TS1f)

G = -891.760479
iv = i127
S^2 = 0.5260

Cartesian Coordinates (Angstroms)

| | | Atom | X | Y | Z |
|---|---|---|---|---|---|
| 1 | C | C | -3.2411448 | -1.2102549 | -0.6227091 |
| 2 | C | C2 | -2.4874199 | -2.3441240 | -0.6934144 |
| 3 | C | C3 | -1.2165378 | -2.4161221 | -0.0674915 |
| 4 | C | C4 | -2.5935456 | 0.0064873 | -0.0586121 |
| 5 | C | C5 | -1.1983952 | 0.1824275 | -0.5854099 |
| 6 | H | H | -4.2614524 | -1.1355115 | -0.9823186 |
| 7 | H | H7 | -2.8158369 | -3.2012848 | -1.2777478 |
| 8 | H | H8 | -1.0091756 | -1.8761448 | 0.8464066 |
| 9 | H | H12 | -1.0032239 | -0.2927791 | -1.5377288 |
| 10 | F | F1 | -3.3510705 | 1.1303709 | -0.3428770 |
| 11 | F | F2 | -2.5823967 | -0.0427127 | 1.3458421 |
| 12 | C | C1 | -0.3037842 | 1.2109320 | -0.1706401 |
| 13 | C | C6 | 1.5609839 | 3.2326926 | 0.5674210 |
| 14 | C | C7 | 0.8735575 | 1.4472643 | -0.9302234 |
| 15 | C | C8 | -0.5101283 | 2.0163608 | 0.9806499 |
| 16 | C | C9 | 0.4012146 | 3.0022437 | 1.3260025 |
| 17 | C | C10 | 1.7792142 | 2.4286188 | -0.5661451 |
| 18 | H | H2 | 1.0593694 | 0.8395307 | -1.8125398 |
| 19 | H | H6 | -1.3893384 | 1.8548016 | 1.5913664 |
| 20 | H | H5 | 0.2168835 | 3.6080511 | 2.2102775 |
| 21 | H | H3 | 2.6724990 | 2.5831353 | -1.1672359 |
| 22 | C | C11 | -0.2498418 | -3.4333873 | -0.4866398 |
| 23 | O | O1 | -0.4115242 | -4.2305043 | -1.3975294 |
| 24 | O | O2 | 0.8750528 | -3.3888637 | 0.2741351 |
| 25 | C | C12 | 1.8774345 | -4.3551328 | -0.0657145 |
| 26 | H | H1 | 1.5029010 | -5.3711438 | 0.0908148 |
| 27 | H | H9 | 2.7184454 | -4.1524296 | 0.5986839 |
| 28 | H | H10 | 2.1789390 | -4.2503344 | -1.1117792 |
| 29 | C | C13 | 2.5350013 | 4.3207831 | 0.9419171 |
| 30 | H | H4 | 3.5725585 | 3.9931121 | 0.8114697 |
| 31 | H | H11 | 2.4041978 | 4.6352923 | 1.9820438 |
| 32 | H | H13 | 2.3965637 | 5.2086255 | 0.3097265 |

## pCl_SM (S1i)

G = -1312.11478
iv = none

Cartesian Coordinates (Angstroms)

| | Atom | | X | Y | Z |
|---|---|---|---|---|---|
| 1 | C | C1 | 1.0629383 | 1.4158113 | -1.5049339 |
| 2 | C | C2 | 0.1398042 | 1.5398942 | -0.3527260 |
| 3 | C | C3 | 0.6951483 | 0.1310238 | -0.8500301 |
| 4 | H | H3 | -0.8906327 | 1.7074024 | -0.6573324 |
| 5 | H | H6 | 1.4579103 | -0.2833529 | -0.1946151 |
| 6 | C | C4 | 0.4782137 | 2.0756827 | 0.9941942 |
| 7 | C | C5 | 0.9765783 | 3.0761558 | 3.5642872 |
| 8 | C | C6 | -0.5657112 | 2.5572507 | 1.7980247 |
| 9 | C | C7 | 1.7836200 | 2.1102276 | 1.5110443 |
| 10 | C | C8 | 2.0351636 | 2.6052449 | 2.7889840 |
| 11 | C | C9 | -0.3281184 | 3.0574004 | 3.0764095 |
| 12 | H | H4 | -1.5847155 | 2.5400634 | 1.4200373 |
| 13 | H | H7 | 2.6183172 | 1.7625201 | 0.9131847 |
| 14 | H | H8 | 3.0474087 | 2.6301134 | 3.1785618 |
| 15 | H | H9 | -1.1452559 | 3.4269295 | 3.6864817 |
| 16 | C | C10 | -0.2266630 | -0.8184726 | -1.4845070 |
| 17 | H | H11 | -0.9308160 | -0.4240651 | -2.2149550 |
| 18 | C | C11 | -0.2609302 | -2.1326999 | -1.2185126 |
| 19 | H | H12 | 0.4155811 | -2.5990981 | -0.5081290 |
| 20 | C | C12 | -1.2386765 | -3.0086821 | -1.8988252 |
| 21 | O | O1 | -2.0799745 | -2.6502155 | -2.7007146 |
| 22 | O | O2 | -1.0788318 | -4.2959737 | -1.5072398 |
| 23 | C | C13 | -1.9833911 | -5.2345541 | -2.1077452 |
| 24 | H | H5 | -3.0166572 | -5.0010274 | -1.8356089 |
| 25 | H | H13 | -1.6960144 | -6.2107232 | -1.7156897 |
| 26 | H | H14 | -1.8922845 | -5.2144020 | -3.1970391 |
| 27 | F | F1 | 0.5750157 | 1.6800851 | -2.7386468 |
| 28 | F | F2 | 2.3408549 | 1.8533815 | -1.4198456 |
| 29 | Cl | Cl1 | 1.2921180 | 3.7040797 | 5.1758867 |

## pCl_TS2 (S-TS1i)

G = -1312.07449
iv = i128
S^2 = 0.5583

Cartesian Coordinates (Angstroms)

| | Atom | | X | Y | Z |
|---|---|---|---|---|---|
| 1 | C | C | -0.7144027 | 2.9378750 | -0.6746949 |
| 2 | C | C2 | -1.8511631 | 2.1867377 | -0.7221207 |
| 3 | C | C3 | -1.9368194 | 0.9513782 | -0.0304521 |
| 4 | C | C4 | 0.4928846 | 2.3128350 | -0.0633652 |
| 5 | C | C5 | 0.6661820 | 0.8922119 | -0.5199505 |
| 6 | H | H | -0.6282091 | 3.9366106 | -1.0883615 |
| 7 | H | H7 | -2.6978162 | 2.4866973 | -1.3362765 |

| | | | X | Y | Z |
|---|---|---|---|---|---|
| 8 | H | H8 | -1.4114849 | 0.7924321 | 0.9016563 |
| 9 | H | H12 | 0.2049960 | 0.6564334 | -1.4701264 |
| 10 | F | F1 | 1.6232164 | 3.0494124 | -0.3706913 |
| 11 | F | F2 | 0.4255219 | 2.3689785 | 1.3382104 |
| 12 | C | C1 | 1.6791377 | 0.0074726 | -0.0450402 |
| 13 | C | C6 | 3.6443348 | -1.8165463 | 0.7981102 |
| 14 | C | C7 | 1.9135574 | -1.2045907 | -0.7476448 |
| 15 | C | C8 | 2.4683437 | 0.2617319 | 1.1090983 |
| 16 | C | C9 | 3.4425531 | -0.6352618 | 1.5198311 |
| 17 | C | C10 | 2.8790198 | -2.1090211 | -0.3356613 |
| 18 | H | H2 | 1.3216660 | -1.4240667 | -1.6324540 |
| 19 | H | H6 | 2.3093340 | 1.1715125 | 1.6741328 |
| 20 | H | H5 | 4.0459134 | -0.4291387 | 2.3977672 |
| 21 | H | H3 | 3.0491448 | -3.0301417 | -0.8828047 |
| 22 | C | C11 | -2.9471157 | -0.0383071 | -0.4165050 |
| 23 | O | O1 | -3.7156678 | 0.0695285 | -1.3586056 |
| 24 | O | O2 | -2.9270737 | -1.1113717 | 0.4138963 |
| 25 | C | C12 | -3.8792252 | -2.1384886 | 0.1055527 |
| 26 | H | H1 | -4.9007242 | -1.7588757 | 0.2013831 |
| 27 | H | H9 | -3.6978636 | -2.9334902 | 0.8297628 |
| 28 | H | H10 | -3.7348658 | -2.5053594 | -0.9146126 |
| 29 | Cl | Cl1 | 4.8766261 | -2.9471882 | 1.3199661 |

## pBr_TS2 (S1j)

G = -3425.80123
iv = none

Cartesian Coordinates (Angstroms)

| Atom | | | X | Y | Z |
|---|---|---|---|---|---|
| 1 | C | C1 | 1.3562471 | 2.6029050 | 1.2666918 |
| 2 | C | C4 | -0.6298350 | 2.8110397 | 3.2079051 |
| 3 | C | C2 | 0.7185734 | 1.4376814 | 1.7172858 |
| 4 | C | C6 | 1.0156669 | 3.8582232 | 1.7679532 |
| 5 | C | C5 | 0.0187999 | 3.9511614 | 2.7362616 |
| 6 | C | C3 | -0.2767281 | 1.5624892 | 2.6997928 |
| 7 | H | H6 | 1.5175193 | 4.7487317 | 1.4057904 |
| 8 | C | C7 | 1.1124539 | 0.1354154 | 1.1133198 |
| 9 | C | C8 | 0.9466757 | -1.1990873 | 1.7365377 |
| 10 | C | C9 | 0.0186687 | -0.9076405 | 0.6097841 |
| 11 | H | H2 | 1.9701604 | 0.1879599 | 0.4468276 |
| 12 | H | H9 | -0.9951829 | -0.6472929 | 0.9066102 |
| 13 | F | F1 | 0.4393115 | -1.3158115 | 2.9860755 |
| 14 | F | F2 | 1.9255881 | -2.1146176 | 1.5561425 |
| 15 | C | C10 | 0.1900134 | -1.5358200 | -0.7050430 |
| 16 | H | H8 | 1.1912244 | -1.8658255 | -0.9766831 |
| 17 | C | C11 | -0.7967142 | -1.7231741 | -1.5941015 |
| 18 | H | H10 | -1.8237509 | -1.4308560 | -1.3952506 |
| 19 | C | C12 | -0.5098685 | -2.3537982 | -2.9004189 |
| 20 | O | O1 | 0.5832979 | -2.7182529 | -3.2898171 |
| 21 | O | O2 | -1.6438204 | -2.4791110 | -3.6311886 |
| 22 | C | C13 | -1.4742752 | -3.0839992 | -4.9217103 |
| 23 | H | H7 | -2.4696650 | -3.1096851 | -5.3663640 |
| 24 | H | H11 | -1.0703997 | -4.0951857 | -4.8222437 |

| | | | | | |
|---|---|---|---|---|---|
| 25 | H | H12 | -0.7925507 | -2.4909454 | -5.5376410 |
| 26 | H | H13 | -1.4009261 | 2.8927997 | 3.9661981 |
| 27 | H | H3 | 2.1299839 | 2.5312127 | 0.5064749 |
| 28 | H | H1 | -0.7762866 | 0.6814874 | 3.0855627 |
| 29 | Br | Br1 | -0.4741812 | 5.6699962 | 3.4252475 |

## pBr_TS2 (S-TS1j)

G = -3425.76075
iv = i133
S^2 = 0.5546

Cartesian Coordinates (Angstroms)

| | Atom | | X | Y | Z |
|---|---|---|---|---|---|
| 1 | C | C | -0.8155237 | 2.9537288 | -0.5005872 |
| 2 | C | C2 | -1.9361827 | 2.1779372 | -0.5224954 |
| 3 | C | C3 | -1.9545462 | 0.9087785 | 0.1112560 |
| 4 | C | C4 | 0.4367717 | 2.3287633 | 0.0126214 |
| 5 | C | C5 | 0.6140255 | 0.9352300 | -0.5191788 |
| 6 | H | H | -0.7736767 | 3.9729385 | -0.8683150 |
| 7 | H | H7 | -2.8226476 | 2.4857335 | -1.0731278 |
| 8 | H | H8 | -1.3765984 | 0.7210921 | 1.0060768 |
| 9 | H | H12 | 0.1141262 | 0.7341125 | -1.4577151 |
| 10 | F | F1 | 1.5334149 | 3.1042878 | -0.3197891 |
| 11 | F | F2 | 0.4451122 | 2.3186286 | 1.4168530 |
| 12 | C | C1 | 1.6689735 | 0.0538574 | -0.1377762 |
| 13 | C | C6 | 3.7061034 | -1.7621794 | 0.5299251 |
| 14 | C | C7 | 1.8972427 | -1.1169033 | -0.9086001 |
| 15 | C | C8 | 2.5045807 | 0.2725334 | 0.9903035 |
| 16 | C | C9 | 3.5144744 | -0.6203524 | 1.3151728 |
| 17 | C | C10 | 2.8992134 | -2.0179835 | -0.5834434 |
| 18 | H | H2 | 1.2701520 | -1.3074775 | -1.7757957 |
| 19 | H | H6 | 2.3514741 | 1.1504878 | 1.6046668 |
| 20 | H | H5 | 4.1489201 | -0.4387045 | 2.1760502 |
| 21 | H | H3 | 3.0594091 | -2.9063750 | -1.1843230 |
| 22 | C | C11 | -2.9634732 | -0.0861036 | -0.2659838 |
| 23 | O | O1 | -3.7881729 | 0.0484362 | -1.1556234 |
| 24 | O | O2 | -2.8700630 | -1.1978847 | 0.5058995 |
| 25 | C | C12 | -3.8159820 | -2.2322315 | 0.2024378 |
| 26 | H | H1 | -4.8390917 | -1.8783145 | 0.3588484 |
| 27 | H | H9 | -3.5853585 | -3.0496220 | 0.8865239 |
| 28 | H | H10 | -3.7123637 | -2.5580693 | -0.8366212 |
| 29 | Br | Br1 | 5.0896863 | -2.9943441 | 0.9927401 |

### pCF3_SM (S1k)

G = -1189.54302
iv = none

Cartesian Coordinates (Angstroms)

| | Atom | | X | Y | Z |
|---|---|---|---|---|---|
| 1 | C | C1 | 1.3905282 | 0.7055646 | -1.7814656 |
| 2 | C | C2 | 0.2860935 | 1.0987292 | -0.8829945 |
| 3 | C | C3 | 0.6555983 | -0.4194453 | -1.1289813 |
| 4 | H | H3 | -0.6187309 | 1.4030623 | -1.4078014 |
| 5 | H | H6 | 1.1862778 | -0.8794566 | -0.2982996 |
| 6 | C | C4 | 0.4892453 | 1.7050214 | 0.4698021 |
| 7 | C | C5 | 0.7850619 | 2.8541838 | 3.0090151 |
| 8 | C | C6 | -0.3390294 | 1.3145880 | 1.5311086 |
| 9 | C | C7 | 1.4671871 | 2.6831757 | 0.6967647 |
| 10 | C | C8 | 1.6144269 | 3.2544324 | 1.9584724 |
| 11 | C | C9 | -0.1953346 | 1.8829654 | 2.7939078 |
| 12 | H | H4 | -1.1003497 | 0.5572787 | 1.3640782 |
| 13 | H | H7 | 2.1162669 | 2.9943651 | -0.1141762 |
| 14 | H | H8 | 2.3696859 | 4.0159093 | 2.1260048 |
| 15 | H | H9 | -0.8429598 | 1.5783240 | 3.6088363 |
| 16 | C | C10 | -0.2602967 | -1.2750697 | -1.8940802 |
| 17 | H | H11 | -0.7489088 | -0.8381627 | -2.7635918 |
| 18 | C | C11 | -0.5380935 | -2.5527254 | -1.5965082 |
| 19 | H | H12 | -0.0868739 | -3.0667832 | -0.7526672 |
| 20 | C | C12 | -1.4830539 | -3.3227510 | -2.4344922 |
| 21 | O | O1 | -2.0871486 | -2.8970320 | -3.4003378 |
| 22 | O | O2 | -1.5959496 | -4.5945813 | -1.9822844 |
| 23 | C | C13 | -2.4876238 | -5.4324597 | -2.7329928 |
| 24 | H | H5 | -3.5059636 | -5.0348042 | -2.7029678 |
| 25 | H | H13 | -2.4440293 | -6.4107954 | -2.2533911 |
| 26 | H | H14 | -2.1657269 | -5.4996729 | -3.7757532 |
| 27 | F | F1 | 1.2442136 | 0.8987727 | -3.1118325 |
| 28 | F | F2 | 2.6753105 | 0.9304317 | -1.4225751 |
| 29 | C | C14 | 0.9962693 | 3.4407113 | 4.3786791 |
| 30 | F | F3 | 2.0580250 | 2.8770349 | 5.0005748 |
| 31 | F | F4 | 1.2410832 | 4.7691376 | 4.3188786 |
| 32 | F | F5 | -0.0752004 | 3.2600513 | 5.1810706 |

### pCF3_TS2 (S-TS1k)

G = -1189.50258
iv = i125
S^2 = 0.6218

Cartesian Coordinates (Angstroms)

| | Atom | | X | Y | Z |
|---|---|---|---|---|---|
| 1 | C | C | -3.2797245 | -1.2121588 | -0.6934350 |
| 2 | C | C2 | -2.5116752 | -2.3389138 | -0.7257791 |
| 3 | C | C3 | -1.2485162 | -2.3795873 | -0.0840931 |
| 4 | C | C4 | -2.6572962 | 0.0335206 | -0.1545598 |

| | | | | | |
|---|---|---|---|---|---|
| 5 | C | C5 | -1.2544317 | 0.2152299 | -0.6599795 |
| 6 | H | H | -4.2960100 | -1.1609020 | -1.0682988 |
| 7 | H | H7 | -2.8238888 | -3.2146668 | -1.2915952 |
| 8 | H | H8 | -1.0537595 | -1.8003942 | 0.8086027 |
| 9 | H | H12 | -1.0478156 | -0.2542267 | -1.6132811 |
| 10 | F | F1 | -3.4240074 | 1.1344557 | -0.4887828 |
| 11 | F | F2 | -2.6735928 | 0.0180651 | 1.2469293 |
| 12 | C | C1 | -0.3414879 | 1.2165497 | -0.2128568 |
| 13 | C | C6 | 1.5803490 | 3.1115657 | 0.6072974 |
| 14 | C | C7 | 0.8566129 | 1.4185644 | -0.9486864 |
| 15 | C | C8 | -0.5428462 | 2.0040522 | 0.9524360 |
| 16 | C | C9 | 0.4027038 | 2.9375492 | 1.3464393 |
| 17 | C | C10 | 1.8018631 | 2.3468040 | -0.5454231 |
| 18 | H | H2 | 1.0317660 | 0.8247198 | -1.8418622 |
| 19 | H | H6 | -1.4418121 | 1.8672061 | 1.5393060 |
| 20 | H | H5 | 0.2384953 | 3.5271246 | 2.2426382 |
| 21 | H | H3 | 2.7160970 | 2.4785463 | -1.1145699 |
| 22 | C | C11 | -0.2546706 | -3.3889999 | -0.4658764 |
| 23 | O | O1 | -0.3981751 | -4.2218233 | -1.3459778 |
| 24 | O | O2 | 0.8666229 | -3.2780076 | 0.2884928 |
| 25 | C | C12 | 1.9083338 | -4.2149400 | -0.0204087 |
| 26 | H | H1 | 1.5696190 | -5.2398952 | 0.1571965 |
| 27 | H | H9 | 2.7348424 | -3.9637807 | 0.6454817 |
| 28 | H | H10 | 2.2139805 | -4.1210829 | -1.0664875 |
| 29 | C | C13 | 2.5837831 | 4.1520212 | 1.0246198 |
| 30 | F | F3 | 3.8341185 | 3.8301821 | 0.6250771 |
| 31 | F | F4 | 2.6119925 | 4.3099328 | 2.3660114 |
| 32 | F | F5 | 2.2985296 | 5.3632898 | 0.4914248 |

## pCN_SM (S1l)

G = -944.753991
iv = none

Cartesian Coordinates (Angstroms)

| | Atom | | X | Y | Z |
|---|---|---|---|---|---|
| 1 | C | C1 | 1.0319461 | 1.2778849 | -1.6937737 |
| 2 | C | C2 | 0.0960445 | 1.4105239 | -0.5500448 |
| 3 | C | C3 | 0.6562833 | -0.0010430 | -1.0350636 |
| 4 | H | H3 | -0.9313648 | 1.5780907 | -0.8640662 |
| 5 | H | H6 | 1.4120200 | -0.4125834 | -0.3698562 |
| 6 | C | C4 | 0.4258318 | 1.9500567 | 0.7956994 |
| 7 | C | C5 | 0.9120884 | 2.9620527 | 3.3793517 |
| 8 | C | C6 | -0.6242761 | 2.4441046 | 1.5866669 |
| 9 | C | C7 | 1.7293305 | 1.9753284 | 1.3219641 |
| 10 | C | C8 | 1.9710244 | 2.4727423 | 2.5969470 |
| 11 | C | C9 | -0.3928655 | 2.9454223 | 2.8618626 |
| 12 | H | H4 | -1.6382702 | 2.4325100 | 1.1963269 |
| 13 | H | H7 | 2.5640968 | 1.6184703 | 0.7302804 |
| 14 | H | H8 | 2.9815089 | 2.4894410 | 2.9924417 |
| 15 | H | H9 | -1.2149625 | 3.3233372 | 3.4609522 |
| 16 | C | C10 | -0.2624683 | -0.9535721 | -1.6712338 |
| 17 | H | H11 | -0.9582229 | -0.5639595 | -2.4121110 |
| 18 | C | C11 | -0.3001821 | -2.2650782 | -1.3947489 |

| | | | | | |
|---|---|---|---|---|---|
| 19 | H | H12 | 0.3681632 | -2.7265686 | -0.6734562 |
| 20 | C | C12 | -1.2719021 | -3.1459489 | -2.0797017 |
| 21 | O | O1 | -2.1015530 | -2.7909135 | -2.8947798 |
| 22 | O | O2 | -1.1196444 | -4.4287818 | -1.6745291 |
| 23 | C | C13 | -2.0183944 | -5.3721133 | -2.2777354 |
| 24 | H | H5 | -3.0547174 | -5.1307052 | -2.0252943 |
| 25 | H | H13 | -1.7410356 | -6.3439496 | -1.8683700 |
| 26 | H | H14 | -1.9089291 | -5.3660210 | -3.3654563 |
| 27 | F | F1 | 0.5541761 | 1.5386864 | -2.9307855 |
| 28 | F | F2 | 2.3079413 | 1.7167013 | -1.5969534 |
| 29 | C | C14 | 1.1623931 | 3.4751953 | 4.6940105 |
| 30 | N | N1 | 1.3659400 | 3.8906899 | 5.7614562 |

### pCN_TS2 (S-TS1l)

G = -944.713152
iv = i137
S^2 = 0.6312

Cartesian Coordinates (Angstroms)

| Atom | | | X | Y | Z |
|---|---|---|---|---|---|
| 1 | C | C | -3.1090839 | -0.9324188 | -0.6288647 |
| 2 | C | C2 | -2.3299329 | -2.0510375 | -0.6568641 |
| 3 | C | C3 | -1.0677673 | -2.0729066 | -0.0120571 |
| 4 | C | C4 | -2.4941114 | 0.3212685 | -0.0957804 |
| 5 | C | C5 | -1.0923355 | 0.5099731 | -0.6068847 |
| 6 | H | H | -4.1244855 | -0.8900055 | -1.0067117 |
| 7 | H | H7 | -2.6301789 | -2.9322518 | -1.2195622 |
| 8 | H | H8 | -0.8846812 | -1.4899310 | 0.8805992 |
| 9 | H | H12 | -0.8991695 | 0.0591984 | -1.5725171 |
| 10 | F | F1 | -3.2673527 | 1.4146714 | -0.4344781 |
| 11 | F | F2 | -2.5075325 | 0.3085765 | 1.3047115 |
| 12 | C | C1 | -0.1713863 | 1.4984922 | -0.1545684 |
| 13 | C | C6 | 1.7710068 | 3.3804744 | 0.6695429 |
| 14 | C | C7 | 1.0077671 | 1.7263356 | -0.9176674 |
| 15 | C | C8 | -0.3437069 | 2.2530012 | 1.0391001 |
| 16 | C | C9 | 0.6063549 | 3.1763799 | 1.4373984 |
| 17 | C | C10 | 1.9601638 | 2.6448086 | -0.5183662 |
| 18 | H | H2 | 1.1573829 | 1.1623713 | -1.8345869 |
| 19 | H | H6 | -1.2283877 | 2.0984419 | 1.6431480 |
| 20 | H | H5 | 0.4639545 | 3.7456288 | 2.3504729 |
| 21 | H | H3 | 2.8537470 | 2.8065734 | -1.1128248 |
| 22 | C | C11 | -0.0618197 | -3.0751446 | -0.3826549 |
| 23 | O | O1 | -0.1945299 | -3.9142076 | -1.2579497 |
| 24 | O | O2 | 1.0527218 | -2.9477331 | 0.3771840 |
| 25 | C | C12 | 2.1026021 | -3.8824063 | 0.0888101 |
| 26 | H | H1 | 1.7739496 | -4.9044379 | 0.2965142 |
| 27 | H | H9 | 2.9289161 | -3.6062448 | 0.7440922 |
| 28 | H | H10 | 2.4016408 | -3.8142906 | -0.9604735 |
| 29 | C | C13 | 2.7594867 | 4.3219007 | 1.0961977 |
| 30 | N | N1 | 3.5667676 | 5.0849199 | 1.4450410 |

## pNO2_SM (S1m)

```
G = -1057.00885
iv = none

Cartesian Coordinates (Angstroms)
       Atom              X              Y              Z
    ---------     -------------  -------------  -------------

   1  C   C1          0.9606723      1.1812896     -1.8531310
   2  C   C2          0.0349246      1.3009843     -0.6988658
   3  C   C3          0.6076703     -0.1026353     -1.1926552
   4  H   H3         -0.9977203      1.4565647     -1.0014392
   5  H   H6          1.3756553     -0.5061260     -0.5366140
   6  C   C4          0.3728128      1.8414923      0.6438564
   7  C   C5          0.8678980      2.8424244      3.1975676
   8  C   C6         -0.6762743      2.3263099      1.4439078
   9  C   C7          1.6821014      1.8755084      1.1574627
  10  C   C8          1.9328487      2.3726145      2.4314691
  11  C   C9         -0.4396627      2.8281044      2.7184806
  12  H   H4         -1.6930405      2.3069563      1.0617016
  13  H   H7          2.5138430      1.5262153      0.5574401
  14  H   H8          2.9367882      2.4057915      2.8364874
  15  H   H9         -1.2428769      3.2030971      3.3406388
  16  C   C10        -0.3076445     -1.0649760     -1.8194009
  17  H   H11        -1.0183375     -0.6823453     -2.5496635
  18  C   C11        -0.3245048     -2.3775919     -1.5468055
  19  H   H12         0.3591994     -2.8329974     -0.8361436
  20  C   C12        -1.2937805     -3.2687425     -2.2226481
  21  O   O1         -2.1399540     -2.9205701     -3.0234733
  22  O   O2         -1.1171806     -4.5510993     -1.8270998
  23  C   C13        -2.0109161     -5.5043847     -2.4224850
  24  H   H5         -3.0469881     -5.2780942     -2.1555785
  25  H   H13        -1.7149043     -6.4738903     -2.0208165
  26  H   H14        -1.9155940     -5.4923632     -3.5114841
  27  F   F1          0.4660604      1.4392177     -3.0836845
  28  F   F2          2.2315837      1.6358706     -1.7681280
  29  N   N1          1.1310496      3.3684380      4.5444891
  30  O   O3          2.2963591      3.3664470      4.9414150
  31  O   O4          0.1699121      3.7784903      5.1952003
```

## pNO2_TS2 (S-TS1m)

```
G = -1056.96757
iv = i146
S^2 = 0.6042

 Cartesian Coordinates (Angstroms)
       Atom              X              Y              Z
    ---------     -------------  -------------  -------------

   1  C   C          -1.0505402      3.1613109     -0.7618680
   2  C   C2         -2.1778288      2.3962176     -0.7922475
   3  C   C3         -2.2302077      1.1537247     -0.1084434
   4  C   C4          0.1781153      2.5460614     -0.1766937
   5  C   C5          0.3521525      1.1241184     -0.6346538
```

| | | | | | |
|---|---|---|---|---|---|
| 6 | H | H | -0.9837357 | 4.1629145 | -1.1721862 |
| 7 | H | H7 | -3.0406011 | 2.6876625 | -1.3875016 |
| 8 | H | H8 | -1.6896861 | 0.9984542 | 0.8158345 |
| 9 | H | H12 | -0.1009347 | 0.8923349 | -1.5900162 |
| 10 | F | F1 | 1.2940308 | 3.2870577 | -0.5132652 |
| 11 | F | F2 | 0.1336866 | 2.6051720 | 1.2220645 |
| 12 | C | C1 | 1.3486363 | 0.2263447 | -0.1484016 |
| 13 | C | C6 | 3.2565094 | -1.6254149 | 0.7179857 |
| 14 | C | C7 | 1.5537745 | -0.9991868 | -0.8410035 |
| 15 | C | C8 | 2.1409860 | 0.4810842 | 1.0053119 |
| 16 | C | C9 | 3.0916634 | -0.4325935 | 1.4277239 |
| 17 | C | C10 | 2.4929002 | -1.9212115 | -0.4151085 |
| 18 | H | H2 | 0.9577564 | -1.2107602 | -1.7245399 |
| 19 | H | H6 | 2.0011554 | 1.4019270 | 1.5562857 |
| 20 | H | H5 | 3.7062824 | -0.2451737 | 2.2997809 |
| 21 | H | H3 | 2.6528627 | -2.8563351 | -0.9375804 |
| 22 | C | C11 | -3.2254250 | 0.1435432 | -0.4913149 |
| 23 | O | O1 | -4.0094962 | 0.2502472 | -1.4195601 |
| 24 | O | O2 | -3.1620420 | -0.9377572 | 0.3211888 |
| 25 | C | C12 | -4.0918647 | -1.9882898 | 0.0159936 |
| 26 | H | H1 | -5.1206382 | -1.6338762 | 0.1248956 |
| 27 | H | H9 | -3.8819071 | -2.7821657 | 0.7331040 |
| 28 | H | H10 | -3.9474514 | -2.3441567 | -1.0078561 |
| 29 | N | N1 | 4.2574823 | -2.5951503 | 1.1752864 |
| 30 | O | O3 | 4.9390762 | -2.2930285 | 2.1558377 |
| 31 | O | O4 | 4.3552888 | -3.6530746 | 0.5509473 |

**Phenolate Computational Attempts**

## Phenolate Intermediate - before opt (S7)

Cartesian Coordinates (Angstroms)

| | | Atom | X | Y | Z |
|---|---|---|---|---|---|
| 1 | H | H1 | -1.2586477 | 1.7626345 | -0.6723651 |
| 2 | C | C1 | -0.9593205 | 2.4202187 | 0.1371390 |
| 3 | C | C4 | -0.2979218 | 4.1741696 | 2.1509685 |
| 4 | C | C2 | 0.2137577 | 2.1675758 | 0.8567993 |
| 5 | C | C6 | -1.7765691 | 3.5228465 | 0.4322519 |
| 6 | C | C5 | -1.4837140 | 4.4444830 | 1.4487606 |
| 7 | C | C3 | 0.5323914 | 3.0761858 | 1.8689170 |
| 8 | H | H6 | -2.6812601 | 3.6767329 | -0.1548029 |
| 9 | H | H3 | 1.4327505 | 2.9470927 | 2.4620282 |
| 10 | H | H4 | -0.0103865 | 4.8530754 | 2.9532110 |
| 11 | C | C7 | 1.0948251 | 0.9939612 | 0.5158621 |
| 12 | C | C8 | 1.6506051 | 0.0771063 | 1.5469454 |
| 13 | C | C9 | 0.5283833 | -0.4138679 | 0.7043742 |
| 14 | H | H9 | -0.4263771 | -0.5138067 | 1.2125096 |
| 15 | F | F1 | 1.4564273 | 0.2726912 | 2.8952971 |
| 16 | F | F2 | 2.9162592 | -0.4510259 | 1.3985080 |
| 17 | C | C10 | 0.7675142 | -1.4081322 | -0.3507062 |
| 18 | H | H8 | 1.7384715 | -1.3585969 | -0.8450345 |
| 19 | C | C11 | -0.1058439 | -2.3552803 | -0.7279450 |
| 20 | H | H10 | -1.0813320 | -2.4452598 | -0.2647076 |
| 21 | C | C12 | 0.2326341 | -3.3145459 | -1.7973213 |

| | | | | | |
|---|---|---|---|---|---|
| 22 | O | O1 | 1.2898992 | -3.3544446 | -2.4044986 |
| 23 | O | O2 | -0.8331080 | -4.1340864 | -1.9776217 |
| 24 | C | C13 | -0.6385568 | -5.1147946 | -2.9957679 |
| 25 | H | H7 | 0.1956046 | -5.7751339 | -2.7383907 |
| 26 | H | H11 | -0.4683390 | -4.6343656 | -3.9644557 |
| 27 | H | H12 | -1.5482733 | -5.7177461 | -3.0641878 |
| 28 | H | H2 | 1.7504441 | 1.1589466 | -0.3355664 |
| 29 | O | O3 | -2.2303178 | 5.4433666 | 1.7097995 |

## Phenolate Intermediate - after geometry optimisation (Total Charge = -1) (S8)

Spartan'10: G = -927.192975, iv = none

Cartesian Coordinates (Angstroms)

| Atom | | | X | Y | Z |
|---|---|---|---|---|---|
| 1 | H | H1 | 1.2808579 | 3.0897020 | 0.6306506 |
| 2 | C | C1 | 0.7034081 | 2.9159564 | 1.5382962 |
| 3 | C | C4 | -0.7906655 | 2.3886376 | 3.8520824 |
| 4 | C | C2 | 0.4615034 | 1.5430458 | 1.9214290 |
| 5 | C | C6 | 0.2458640 | 3.9704274 | 2.2653526 |
| 6 | C | C5 | -0.5409795 | 3.7856705 | 3.4865468 |
| 7 | C | C3 | -0.3177473 | 1.3411958 | 3.1233992 |
| 8 | H | H6 | 0.4449109 | 4.9963243 | 1.9647671 |
| 9 | H | H3 | -0.5169180 | 0.3229192 | 3.4373228 |
| 10 | H | H4 | -1.3749331 | 2.2249088 | 4.7547202 |
| 11 | C | C7 | 0.9940677 | 0.5293339 | 1.1434880 |
| 12 | C | C8 | 0.7754011 | -0.9401199 | 1.1873830 |
| 13 | C | C9 | -0.3501997 | -1.1906383 | 0.2744310 |
| 14 | H | H2 | 1.5802893 | 0.8142811 | 0.2724571 |
| 15 | H | H9 | -1.3228352 | -0.8910906 | 0.6559472 |
| 16 | F | F1 | 0.5348639 | -1.4046105 | 2.4809257 |
| 17 | F | F2 | 1.9329416 | -1.5751813 | 0.7622446 |
| 18 | C | C10 | -0.1909201 | -1.6214304 | -1.0372029 |
| 19 | H | H8 | 0.8233621 | -1.8602679 | -1.3499328 |
| 20 | C | C11 | -1.1941300 | -1.8064516 | -1.9796295 |
| 21 | H | H10 | -2.2305457 | -1.5924159 | -1.7330885 |
| 22 | C | C12 | -0.9905567 | -2.2883093 | -3.3131497 |
| 23 | O | O1 | -1.8563495 | -2.4639529 | -4.1707136 |
| 24 | O | O2 | 0.3441622 | -2.5618713 | -3.6054491 |
| 25 | C | C13 | 0.5736672 | -3.0254089 | -4.9252715 |
| 26 | H | H7 | 1.6529881 | -3.1873279 | -5.0035254 |
| 27 | H | H11 | 0.2512599 | -2.2918886 | -5.6739473 |
| 28 | H | H12 | 0.0402419 | -3.9627018 | -5.1235976 |
| 29 | O | O3 | -0.9630088 | 4.7412646 | 4.1640642 |

## Phenolate VCPR Transition State - before optimisation (S-TS4)

```
Cartesian Coordinates (Angstroms)
       Atom            X             Y             Z
    ---------    ------------- ------------- -------------

  1 C   C           -0.6651935     2.9186490    -0.6746263
  2 C   C2          -1.8137598     2.1842964    -0.7359817
  3 C   C3          -1.9350686     0.9553256    -0.0421748
  4 C   C4           0.5233634     2.2823951    -0.0455186
  5 C   C5           0.6948050     0.8628918    -0.5022731
  6 H   H           -0.5610292     3.9164781    -1.0861974
  7 H   H7          -2.6456154     2.4980068    -1.3636021
  8 H   H8          -1.4145005     0.7833400     0.8899571
  9 H   H12          0.2184147     0.6212074    -1.4429874
 10 F   F1           1.6665307     3.0129670    -0.3296920
 11 F   F2           0.4403775     2.3353397     1.3588655
 12 C   C1           1.7068249    -0.0174399    -0.0321843
 13 C   C6           3.6761591    -1.8539552     0.8237148
 14 C   C7           1.9439024    -1.2362722    -0.7286087
 15 C   C8           2.4992798     0.2380831     1.1207706
 16 C   C9           3.4682911    -0.6598382     1.5330731
 17 C   C10          2.9028556    -2.1391946    -0.3137085
 18 H   H2           1.3507707    -1.4573798    -1.6122523
 19 H   H6           2.3414821     1.1505066     1.6818723
 20 H   H5           4.0689047    -0.4445596     2.4153719
 21 H   H3           3.0786691    -3.0670939    -0.8485266
 22 C   C11         -2.9629413    -0.0102329    -0.4313316
 23 O   O1          -3.7262018     0.1075837    -1.3776194
 24 O   O2          -2.9723572    -1.0843205     0.4028114
 25 C   C12         -3.9442819    -2.0887908     0.0885536
 26 H   H1          -4.9585600    -1.6892821     0.1829482
 27 H   H9          -3.7823516    -2.8906294     0.8103071
 28 H   H10         -3.8063206    -2.4561354    -0.9325037
 29 O   O3           4.6075507    -2.7719458     1.1915431
```

## Phenolate VCPR Transition State - after optimisation (Total Charge = -1) (S-TS4)

```
Spartan'10: G = -927.176731, iv = i107, S^2 = 0.0001

Cartesian Coordinates (Angstroms)
       Atom            X             Y             Z
    ---------    ------------- ------------- -------------

  1 C   C           -0.7028045     3.1038515    -0.7049473
  2 C   C2          -1.8062730     2.3228423    -0.6517956
  3 C   C3          -1.7138557     1.0591249     0.0372463
  4 C   C4           0.5346220     2.4555659    -0.2063623
  5 C   C5           0.5468191     0.9779598    -0.5446132
  6 H   H           -0.6564795     4.1166443    -1.0921863
  7 H   H7          -2.7285382     2.5909061    -1.1690556
  8 H   H8          -1.3345859     1.0349481     1.0507740
  9 H   H12          0.1757076     0.8044173    -1.5488622
 10 F   F1           1.6590641     3.0695692    -0.7550712
 11 F   F2           0.7014846     2.6522265     1.1777744
```

| | | | | | |
|---|---|---|---|---|---|
| 12 | C | C1 | 1.4400750 | 0.0208918 | -0.0467964 |
| 13 | C | C6 | 3.2567048 | -2.0801602 | 0.8438868 |
| 14 | C | C7 | 1.6307000 | -1.1953743 | -0.7939404 |
| 15 | C | C8 | 2.1821669 | 0.1323339 | 1.1826385 |
| 16 | C | C9 | 3.0387980 | -0.8459548 | 1.5968119 |
| 17 | C | C10 | 2.4790717 | -2.1830413 | -0.3899775 |
| 18 | H | H2 | 1.0690408 | -1.3098361 | -1.7214063 |
| 19 | H | H6 | 2.0580032 | 1.0296004 | 1.7780306 |
| 20 | H | H5 | 3.5991611 | -0.7413867 | 2.5237495 |
| 21 | H | H3 | 2.6118024 | -3.0895804 | -0.9767112 |
| 22 | C | C11 | -2.5691403 | -0.0267344 | -0.3637043 |
| 23 | O | O1 | -3.2566035 | -0.0943725 | -1.3823008 |
| 24 | O | O2 | -2.5362285 | -1.0691777 | 0.5430958 |
| 25 | C | C12 | -3.2875790 | -2.2130706 | 0.1628205 |
| 26 | H | H1 | -4.3600864 | -1.9901305 | 0.1056993 |
| 27 | H | H9 | -3.1030485 | -2.9638402 | 0.9357245 |
| 28 | H | H10 | -2.9691897 | -2.5938533 | -0.8138878 |
| 29 | O | O3 | 4.0411914 | -2.9743692 | 1.2233663 |

## Cartesian Coordinates for Alkenoates

2-pyridyl

Cartesian Coordinates (Angstroms)

| | Atom | | X | Y | Z |
|---|---|---|---|---|---|
| 1 | H | H1 | -0.8129841 | 0.1160651 | 2.5439774 |
| 2 | C | C1 | -0.4744235 | 1.0344220 | 2.0725097 |
| 3 | C | C4 | 0.3943767 | 3.2735988 | 0.7940912 |
| 4 | C | C2 | -0.0405019 | 1.0174219 | 0.7369124 |
| 5 | C | C6 | -0.4631111 | 2.2371419 | 2.7754328 |
| 6 | C | C5 | -0.0197364 | 3.3864943 | 2.1263621 |
| 7 | N | N1 | 0.3892637 | 2.1317818 | 0.1089558 |
| 8 | H | H4 | 0.7478073 | 4.1512814 | 0.2545482 |
| 9 | C | C7 | -0.0462173 | -0.2423610 | -0.0175247 |
| 10 | H | H7 | -0.3962029 | -1.1224421 | 0.5161015 |
| 11 | C | C8 | 0.3475029 | -0.3625529 | -1.2967223 |
| 12 | H | H8 | 0.7005955 | 0.5006879 | -1.8504334 |
| 13 | C | C9 | 0.3445492 | -1.6289205 | -2.0555207 |
| 14 | O | O1 | -0.1019638 | -2.6869584 | -1.3305635 |
| 15 | O | O2 | 0.7050368 | -1.7230232 | -3.2132418 |
| 16 | C | C10 | -0.1160287 | -3.9392129 | -2.0292282 |
| 17 | H | H2 | 0.8925777 | -4.2122539 | -2.3520599 |
| 18 | H | H9 | -0.7640376 | -3.8835557 | -2.9083329 |
| 19 | H | H10 | -0.5000801 | -4.6695127 | -1.3157786 |
| 20 | H | H5 | -0.7941878 | 2.2743412 | 3.8097505 |
| 21 | H | H14 | 0.0077656 | 4.3475572 | 2.6307644 |

2-thiazoyl

Cartesian Coordinates (Angstroms)

| | Atom | | X | Y | Z |
|---|---|---|---|---|---|
| 1 | C | C1 | -0.5454436 | 0.0226715 | 0.5437647 |
| 2 | H | H2 | -1.5032974 | 0.5360423 | 0.5852073 |
| 3 | C | C2 | 0.2303064 | 0.1838704 | -0.5432247 |
| 4 | H | H1 | 1.1980552 | -0.2977856 | -0.6521511 |
| 5 | C | C3 | -0.2472724 | -0.7859274 | 1.7085530 |
| 6 | N | N1 | -1.0663557 | -0.8859838 | 2.7293949 |
| 7 | C | C5 | -0.5603522 | -1.7087601 | 3.6968361 |
| 8 | C | C6 | 0.6653319 | -2.2605474 | 3.4372234 |
| 9 | S | S1 | 1.2388862 | -1.7305197 | 1.8953617 |
| 10 | H | H8 | -1.1365473 | -1.8812683 | 4.5989361 |
| 11 | H | H9 | 1.2465596 | -2.9321230 | 4.0541349 |
| 12 | C | C7 | -0.2190138 | 1.0410760 | -1.6602688 |
| 13 | O | O2 | -1.2669478 | 1.6552306 | -1.7114256 |
| 14 | O | O3 | 0.7078642 | 1.0543694 | -2.6485952 |
| 15 | C | C8 | 0.3693540 | 1.8522579 | -3.7929022 |
| 16 | H | H3 | 1.2024448 | 1.7372158 | -4.4868972 |
| 17 | H | H4 | 0.2476541 | 2.9011807 | -3.5085135 |
| 18 | H | H6 | -0.5612263 | 1.4990010 | -4.2454338 |

piperonyl

Cartesian Coordinates (Angstroms)

| | Atom | | X | Y | Z |
|---|---|---|---|---|---|
| 1 | H | H1 | -0.3968724 | 1.5543129 | -1.3560687 |
| 2 | C | C1 | -0.2192789 | 1.5267201 | -0.2859382 |
| 3 | C | C4 | 0.2450682 | 1.4866853 | 2.5233457 |
| 4 | C | C2 | 0.0211857 | 0.2990016 | 0.3837331 |
| 5 | C | C6 | -0.2191316 | 2.6804777 | 0.4651621 |
| 6 | C | C5 | 0.0089855 | 2.6658740 | 1.8389725 |
| 7 | C | C3 | 0.2470043 | 0.3044367 | 1.7726828 |
| 8 | H | H3 | 0.4295927 | -0.6324651 | 2.2881308 |
| 9 | H | H4 | 0.4213928 | 1.4824670 | 3.5934008 |
| 10 | C | C7 | 0.0240148 | -0.9245584 | -0.4176954 |
| 11 | H | H7 | -0.1664618 | -0.7832148 | -1.4795257 |
| 12 | C | C8 | 0.2353728 | -2.1861381 | 0.0068761 |
| 13 | H | H8 | 0.4339741 | -2.4278969 | 1.0460958 |
| 14 | C | C9 | 0.2267218 | -3.3702372 | -0.8687748 |
| 15 | O | O1 | -0.0136184 | -3.0745762 | -2.1734061 |
| 16 | O | O2 | 0.4168139 | -4.5054560 | -0.4720569 |
| 17 | C | C10 | -0.0228284 | -4.2012089 | -3.0594418 |
| 18 | H | H2 | -0.8102129 | -4.9071183 | -2.7801998 |
| 19 | H | H9 | 0.9393850 | -4.7202783 | -3.0349791 |
| 20 | H | H10 | -0.2118046 | -3.7914998 | -4.0526112 |
| 21 | O | O3 | -0.4233879 | 3.9719759 | 0.0435385 |
| 22 | O | O4 | -0.0403356 | 3.9417654 | 2.3335158 |
| 23 | C | C11 | -0.3280039 | 4.7920731 | 1.2135373 |
| 24 | H | H11 | 0.4857080 | 5.5156442 | 1.0892889 |
| 25 | H | H12 | -1.2832833 | 5.3032144 | 1.3824176 |

Ethyl cinnamate

Cartesian Coordinates (Angstroms)

| | Atom | | X | Y | Z |
|---|---|---|---|---|---|
| 1 | H | H1 | 0.8094665 | 2.1829020 | -1.8821796 |
| 2 | C | C1 | 0.6152364 | 2.3709243 | -0.8290218 |
| 3 | C | C4 | 0.1166399 | 2.8516511 | 1.8674978 |
| 4 | C | C2 | 0.2366564 | 1.2981348 | -0.0015361 |
| 5 | C | C6 | 0.7431351 | 3.6617944 | -0.3205155 |
| 6 | C | C5 | 0.4946944 | 3.9069062 | 1.0304224 |
| 7 | C | C3 | -0.0111336 | 1.5636824 | 1.3593274 |
| 8 | H | H6 | 1.0367395 | 4.4750189 | -0.9786682 |
| 9 | H | H5 | 0.5939834 | 4.9120816 | 1.4309070 |
| 10 | H | H3 | -0.3067116 | 0.7558034 | 2.0222448 |
| 11 | H | H4 | -0.0787780 | 3.0361425 | 2.9204475 |
| 12 | C | C7 | 0.1191615 | -0.0348170 | -0.5930555 |
| 13 | C | C8 | -0.2237237 | -1.1829758 | 0.0195615 |
| 14 | H | H2 | 0.3360990 | -0.1044686 | -1.6583634 |
| 15 | H | H7 | -0.4614193 | -1.2461745 | 1.0765785 |
| 16 | C | C10 | -0.2891713 | -2.4409783 | -0.7496115 |
| 17 | O | O1 | -0.0614930 | -2.5691224 | -1.9382809 |
| 18 | O | O2 | -0.6456391 | -3.4745586 | 0.0550586 |
| 19 | C | C11 | -0.7367231 | -4.7496375 | -0.5948518 |
| 20 | H | H8 | 0.2257049 | -5.0290876 | -1.0327061 |
| 21 | H | H11 | -1.4897831 | -4.7263014 | -1.3877804 |
| 22 | H | H12 | -1.0229413 | -5.4569196 | 0.1845253 |

2-furyl

Cartesian Coordinates (Angstroms)

| | Atom | | X | Y | Z |
|---|---|---|---|---|---|
| 1 | C | C1 | -0.5861778 | -0.0457215 | 0.4069199 |
| 2 | H | H2 | -1.6046844 | 0.3335817 | 0.3597083 |
| 3 | C | C2 | 0.2518868 | 0.2643737 | -0.6030869 |
| 4 | H | H1 | 1.2811268 | -0.0766997 | -0.6246977 |
| 5 | C | C3 | -0.2679092 | -0.8368358 | 1.5595421 |
| 6 | C | C4 | -1.0186753 | -1.2156629 | 2.6482011 |
| 7 | C | C5 | -0.1709988 | -2.0004238 | 3.4831726 |
| 8 | C | C6 | 1.0370148 | -2.0482303 | 2.8499015 |
| 9 | O | O1 | 1.0002162 | -1.3521308 | 1.6850159 |
| 10 | H | H5 | -2.0542990 | -0.9572724 | 2.8216408 |
| 11 | H | H8 | -0.4226338 | -2.4661278 | 4.4255788 |
| 12 | H | H9 | 1.9809179 | -2.5160889 | 3.0863099 |
| 13 | C | C7 | -0.2240321 | 1.0933730 | -1.7233965 |
| 14 | O | O2 | -1.3455036 | 1.5517945 | -1.8497766 |
| 15 | O | O3 | 0.7672365 | 1.2943735 | -2.6288477 |
| 16 | C | C8 | 0.4033583 | 2.0961908 | -3.7604271 |
| 17 | H | H3 | 1.2990927 | 2.1540682 | -4.3800789 |
| 18 | H | H4 | 0.0919452 | 3.0950270 | -3.4414956 |
| 19 | H | H6 | -0.4178814 | 1.6324117 | -4.3141840 |

2-thiophenyl

Cartesian Coordinates (Angstroms)

| | | Atom | X | Y | Z |
|---|---|---|---|---|---|
| 1 | C | C1 | -0.4535858 | -0.0243428 | 0.3412620 |
| 2 | H | H2 | -1.4645077 | 0.3808937 | 0.3148997 |
| 3 | C | C2 | 0.3535076 | 0.2789904 | -0.6953413 |
| 4 | H | H1 | 1.3778722 | -0.0748932 | -0.7661434 |
| 5 | C | C3 | -0.1423379 | -0.8297731 | 1.4961240 |
| 6 | C | C4 | -0.9971067 | -1.1209763 | 2.5413079 |
| 7 | C | C5 | -0.4150242 | -1.9414346 | 3.5445300 |
| 8 | C | C6 | 0.8834163 | -2.2741323 | 3.2616380 |
| 9 | S | S1 | 1.4183528 | -1.5900039 | 1.7624876 |
| 10 | H | H5 | -2.0159633 | -0.7489986 | 2.5761987 |
| 11 | H | H8 | -0.9355970 | -2.2706286 | 4.4369541 |
| 12 | H | H9 | 1.5598762 | -2.8829791 | 3.8468853 |
| 13 | C | C7 | -0.1471378 | 1.1227217 | -1.7944892 |
| 14 | O | O2 | -1.2619570 | 1.6062332 | -1.8750323 |
| 15 | O | O3 | 0.8116420 | 1.3030524 | -2.7380140 |
| 16 | C | C8 | 0.4213655 | 2.1165398 | -3.8527611 |
| 17 | H | H3 | 1.2929905 | 2.1560024 | -4.5071847 |
| 18 | H | H4 | 0.1455531 | 3.1209030 | -3.5190446 |
| 19 | H | H6 | -0.4313588 | 1.6728258 | -4.3742764 |

3-Me-2-furyl

Cartesian Coordinates (Angstroms)

| | | Atom | X | Y | Z |
|---|---|---|---|---|---|
| 1 | C | C1 | -0.1800432 | 0.0010486 | -0.0533776 |
| 2 | H | H2 | -1.1985498 | -0.0461630 | -0.4326809 |
| 3 | C | C2 | 0.6580214 | -1.0089582 | -0.3634728 |
| 4 | H | H1 | 1.6872614 | -1.0305690 | -0.0223994 |
| 5 | C | C3 | 0.1382254 | 1.1536708 | 0.7377366 |
| 6 | C | C4 | -0.6125407 | 2.2423298 | 1.1165637 |
| 7 | C | C5 | 0.2351358 | 3.0773013 | 1.9013247 |
| 8 | C | C6 | 1.4431494 | 2.4440302 | 1.9491312 |
| 9 | O | O1 | 1.4063508 | 1.2791446 | 1.2530316 |
| 10 | H | H8 | -0.0164993 | 4.0197075 | 2.3670287 |
| 11 | H | H9 | 2.3870525 | 2.6804386 | 2.4169897 |
| 12 | C | C7 | 0.1821025 | -2.1292679 | -1.1924721 |
| 13 | O | O2 | -0.9393690 | -2.2556479 | -1.6508937 |
| 14 | O | O3 | 1.1733711 | -3.0347191 | -1.3934727 |
| 15 | C | C8 | 0.8094929 | -4.1662985 | -2.1952900 |
| 16 | H | H3 | 1.7052273 | -4.7859503 | -2.2531674 |
| 17 | H | H4 | 0.4980798 | -3.8473669 | -3.1941262 |
| 18 | H | H6 | -0.0117468 | -4.7200553 | -1.7315109 |
| 19 | C | C9 | -2.0787718 | 2.4878848 | 0.7507357 |
| 20 | H | H5 | -2.1312767 | 3.0980072 | -0.1582252 |
| 21 | H | H7 | -2.5773364 | 3.0137329 | 1.5730088 |
| 22 | H | H10 | -2.5773364 | 1.5276999 | 0.5755379 |

N-Boc-2-pyrroyl

Cartesian Coordinates (Angstroms)

| | Atom | | X | Y | Z |
|---|---|---|---|---|---|
| 1 | C | C1 | 1.2835003 | 0.0246371 | -0.8176050 |
| 2 | H | H2 | 0.4573820 | 0.6022074 | -0.4253786 |
| 3 | C | C2 | 1.6434672 | 0.2773757 | -2.0940092 |
| 4 | H | H1 | 2.4366695 | -0.2564087 | -2.6077389 |
| 5 | C | C3 | 1.9067766 | -0.9304564 | 0.0646007 |
| 6 | C | C4 | 3.1300951 | -1.5680919 | -0.0617223 |
| 7 | C | C5 | 3.3727855 | -2.3355217 | 1.1068869 |
| 8 | C | C6 | 2.3000683 | -2.1602396 | 1.9417346 |
| 9 | N | N1 | 1.3927693 | -1.3112357 | 1.3275764 |
| 10 | H | H5 | 3.7966605 | -1.4526068 | -0.9059462 |
| 11 | H | H8 | 4.2415600 | -2.9448850 | 1.3147515 |
| 12 | H | H9 | 2.0707186 | -2.5626340 | 2.9152865 |
| 13 | C | C7 | 0.9274185 | 1.3158813 | -2.8498912 |
| 14 | O | O2 | 0.0024641 | 1.9983814 | -2.4411995 |
| 15 | O | O3 | 1.4288196 | 1.4302126 | -4.1074914 |
| 16 | C | C8 | 0.7931884 | 2.4165788 | -4.9300804 |
| 17 | H | H3 | 1.3037365 | 2.3698145 | -5.8929337 |
| 18 | H | H4 | 0.8961602 | 3.4127238 | -4.4900456 |
| 19 | H | H6 | -0.2711004 | 2.1941355 | -5.0492187 |
| 20 | C | C9 | 0.1569124 | -1.0315429 | 1.9667199 |
| 21 | O | O1 | -0.0304055 | -1.3261031 | 3.1276429 |
| 22 | O | O4 | -0.7017898 | -0.4573704 | 1.1277316 |
| 23 | C | C10 | -2.0430104 | 0.0056071 | 1.5781028 |
| 24 | C | C11 | -1.8692816 | 1.0950983 | 2.6378016 |
| 25 | H | H7 | -1.3961684 | 0.7013709 | 3.5394425 |
| 26 | H | H12 | -2.8524620 | 1.4962416 | 2.9074989 |
| 27 | H | H13 | -1.2634745 | 1.9190589 | 2.2458376 |
| 28 | C | C12 | -2.8591165 | -1.1864860 | 2.0837571 |
| 29 | H | H10 | -2.4441450 | -1.5982818 | 3.0048652 |
| 30 | H | H14 | -2.8916951 | -1.9750289 | 1.3241647 |
| 31 | H | H15 | -3.8873846 | -0.8606490 | 2.2757576 |
| 32 | C | C13 | -2.6408231 | 0.5700264 | 0.2877880 |
| 33 | H | H11 | -2.0110335 | 1.3598982 | -0.1330557 |
| 34 | H | H16 | -3.6325881 | 0.9862970 | 0.4928455 |
| 35 | H | H17 | -2.7466742 | -0.2180043 | -0.4644761 |

## Baldwin VCP INT (8b)

| | | | |
|---|---|---|---|
| H | 1.442750 | 0.921698 | -1.239800 |
| C | 1.174223 | 1.088619 | -0.199764 |
| C | -0.205397 | 1.594368 | 0.105723 |
| C | 0.151197 | 0.149790 | 0.419148 |
| H | 2.000646 | 1.417717 | 0.424305 |
| H | -0.883108 | 1.775465 | -0.724784 |
| H | -0.338621 | 2.274416 | 0.942683 |
| H | 0.296804 | -0.086999 | 1.471332 |
| C | -0.430333 | -0.944346 | -0.387160 |
| H | -0.576162 | -0.721840 | -1.446325 |
| C | -0.778212 | -2.146752 | 0.078483 |

```
H       -1.202504     -2.908299     -0.569397
H       -0.651283     -2.413837      1.125558

Baldwin VCPR TS

C       -0.215191      1.190052     -0.227090
C       -1.349112      0.421866     -0.217252
C       -1.386915     -0.875798      0.339664
C        1.093057      0.583636      0.232001
C        1.230186     -0.836001     -0.253629
H       -0.232714      2.211810     -0.596753
H       -2.244410      0.795796     -0.714968
H       -0.751599     -1.161244      1.168910
H        1.069204     -1.040072     -1.308078
H        1.867359     -1.532357      0.287554
H        1.931023      1.206053     -0.128602
H        1.174797      0.603886      1.332791
H       -2.185686     -1.567628      0.085453
```

**[3,3]-Mechanism Searching**

**(S-TS6)**

```
[3,3] Transoid Triplet Transition State
Spartan'10: G = -852.420461, iv = i613, S^2 = 2.0177
[3,3] Transoid Diradicaloid
Spartan'10: G = -852.445602, iv = i552, S^2 = 0.6134
[3,3] Transoid Closed Shell
Spartan'10: G = -852.435275, iv = i67

Cartesian Coordinates (Angstroms)
       Atom             X             Y             Z
    ---------    ------------- ------------- -------------

  1 C   C              2.3374563     0.3330489    -2.1080915
  2 F   F              2.4256384    -0.9115264    -2.7529639
  3 F   F3             3.4032088     1.0617736    -2.6006786
  4 C   C4             2.5657165     0.1320971    -0.6505621
  5 H   H              3.5622940     0.3928016    -0.3080310
  6 C   C6             1.0789034     0.9809830    -2.6058204
  7 H   H7             1.1643354     1.2641873    -3.6525420
  8 C   C8            -0.0983823     1.1552714    -1.9821114
  9 H   H9            -0.9281032     1.5043633    -2.5947118
 10 C   C10           -0.4496947     0.8566367    -0.5843182
 11 H   H11            0.0756405     1.4190667     0.1825713
 12 C   C12           -1.9129894     0.7750143    -0.3343334
 13 O   O             -2.7518708     0.4856100    -1.1684378
 14 O   O14           -2.2070567     1.0142512     0.9659606
 15 C   C15           -3.5943332     0.8855277     1.3115032
 16 H   H16           -3.9494122    -0.1297150     1.1106489
 17 H   H17           -4.2020983     1.5907992     0.7377134
 18 H   H18           -3.6522700     1.1081487     2.3777128
 19 C   C19            1.6719391    -0.5048293     0.2540782
 20 C   C20           -0.0900694    -1.7376610     2.1226550
 21 C   C21            0.3209353    -0.9075669    -0.1755517
 22 C   C22            2.0237697    -0.6627839     1.5896103
 23 C   C23            1.1433487    -1.2623996     2.5352652
```

| | | | | | |
|---|---|---|---|---|---|
| 24 | C | C24 | -0.4724439 | -1.6238969 | 0.7762227 |
| 25 | H | H25 | 0.2363543 | -1.2466427 | -1.2060124 |
| 26 | H | H26 | 3.0008465 | -0.3182240 | 1.9195639 |
| 27 | H | H27 | 1.4593765 | -1.3631237 | 3.5690190 |
| 28 | H | H28 | -1.4118475 | -2.0562905 | 0.4427653 |
| 29 | H | H29 | -0.7491923 | -2.2349208 | 2.8288763 |

## S-TS7a

[3,3] Cisoid Triplet Transition State
Spartan'10: G = -852.411611, iv = i538, S^2 = 2.0397

Cartesian Coordinates (Angstroms)

| | | Atom | X | Y | Z |
|---|---|---|---|---|---|
| 1 | C | C | 1.6839700 | 1.4112607 | -2.0466638 |
| 2 | F | F | 2.5766738 | 1.4465241 | -3.1009893 |
| 3 | F | F3 | 1.4296522 | 2.7520214 | -1.7493923 |
| 4 | C | C4 | 2.3582761 | 0.7947560 | -0.8570935 |
| 5 | H | H | 3.2596855 | 1.2951852 | -0.5073202 |
| 6 | C | C6 | 0.4237176 | 0.7233898 | -2.4994167 |
| 7 | H | H7 | 0.4089928 | 0.4155717 | -3.5405067 |
| 8 | C | C8 | -0.6096278 | 0.4606696 | -1.6793565 |
| 9 | H | H9 | -1.4616330 | -0.1043965 | -2.0492272 |
| 10 | C | C10 | -0.6553100 | 0.8631396 | -0.2727040 |
| 11 | H | H11 | -0.2399756 | 1.8467288 | -0.0730032 |
| 12 | C | C12 | -1.9414769 | 0.6218576 | 0.4290469 |
| 13 | O | O | -2.8204701 | -0.1344267 | 0.0639202 |
| 14 | O | O14 | -1.9994752 | 1.3246740 | 1.5906512 |
| 15 | C | C15 | -3.1948822 | 1.1333352 | 2.3624714 |
| 16 | H | H16 | -3.3078673 | 0.0836187 | 2.6482809 |
| 17 | H | H17 | -4.0751618 | 1.4390237 | 1.7906988 |
| 18 | H | H18 | -3.0761145 | 1.7608966 | 3.2465162 |
| 19 | C | C19 | 1.7705130 | -0.3620541 | -0.1613052 |
| 20 | C | C20 | 0.4476392 | -2.5605370 | 1.0378059 |
| 21 | C | C21 | 2.0701335 | -1.6586126 | -0.5413184 |
| 22 | C | C22 | 0.6974892 | -0.1188347 | 0.7983874 |
| 23 | C | C23 | 0.1260122 | -1.2833207 | 1.4447133 |
| 24 | C | C24 | 1.4172969 | -2.7631849 | 0.0394031 |
| 25 | H | H25 | 2.8258561 | -1.8237732 | -1.3048759 |
| 26 | H | H26 | 0.8222681 | 0.7481696 | 1.4468545 |
| 27 | H | H27 | -0.5881718 | -1.1257393 | 2.2476791 |
| 28 | H | H28 | 1.6790043 | -3.7695293 | -0.2736922 |
| 29 | H | H29 | -0.0270143 | -3.4164129 | 1.5104359 |

## S-TS7b

[3,3] Cisoid Closed Shell Transition State
Spartan'10: G = -852.464842, iv = i385

Cartesian Coordinates (Angstroms)

| | Atom | | X | Y | Z |
|---|---|---|---|---|---|
| 1 | C | C | 1.8306612 | 1.5956508 | -1.8575741 |
| 2 | F | F | 2.6870122 | 2.1184195 | -2.7906786 |
| 3 | F | F3 | 1.8069515 | 2.4827641 | -0.7996484 |
| 4 | C | C4 | 2.2410148 | 0.2100367 | -1.4905855 |
| 5 | H | H | 2.8205396 | -0.2931099 | -2.2601120 |
| 6 | C | C6 | 0.4929078 | 1.3164800 | -2.4574821 |
| 7 | H | H7 | 0.5094519 | 1.1703634 | -3.5334670 |
| 8 | C | C8 | -0.6102237 | 0.9738261 | -1.7309582 |
| 9 | H | H9 | -1.4344701 | 0.4807224 | -2.2423811 |
| 10 | C | C10 | -0.7132016 | 1.0783911 | -0.3075953 |
| 11 | H | H11 | -0.3784892 | 2.0027709 | 0.1423622 |
| 12 | C | C12 | -1.9631461 | 0.5379098 | 0.2891657 |
| 13 | O | O | -2.7047621 | -0.2721485 | -0.2306949 |
| 14 | O | O14 | -2.1783377 | 1.0574306 | 1.5250633 |
| 15 | C | C15 | -3.3528503 | 0.5724078 | 2.1939135 |
| 16 | H | H16 | -3.3040250 | -0.5128555 | 2.3185646 |
| 17 | H | H17 | -4.2514107 | 0.8221237 | 1.6232500 |
| 18 | H | H18 | -3.3648593 | 1.0714663 | 3.1636692 |
| 19 | C | C19 | 1.7137344 | -0.5284032 | -0.4510369 |
| 20 | C | C20 | 0.5579043 | -2.1472863 | 1.5830749 |
| 21 | C | C21 | 1.8689793 | -1.9623226 | -0.4539682 |
| 22 | C | C22 | 0.8996587 | 0.0751830 | 0.6064962 |
| 23 | C | C23 | 0.3968822 | -0.7962414 | 1.6475472 |
| 24 | C | C24 | 1.3030441 | -2.7412087 | 0.5104419 |
| 25 | H | H25 | 2.4373971 | -2.4126343 | -1.2642251 |
| 26 | H | H26 | 1.2378510 | 1.0456992 | 0.9401489 |
| 27 | H | H27 | -0.1236510 | -0.3391174 | 2.4835210 |
| 28 | H | H28 | 1.4204899 | -3.8209299 | 0.4779126 |
| 29 | H | H29 | 0.1549463 | -2.7853874 | 2.3652758 |

**[3,3]-Methodology Screening**

## Divinyl Species VCP INT (S22)

Spartan'10: B3LYP/6-31G*, G = -272.648654, iv = none
Gaussian'09: B3LYP/6-31G*, G = -272.569679, iv = none
M06-2X/6-31G*,G = -272.431421, iv = none

Cartesian Coordinates (Angstroms)

| | Atom | | X | Y | Z |
|---|---|---|---|---|---|
| 1 | C | C1 | 1.6922994 | 0.1392844 | 0.1378774 |
| 2 | C | C2 | 0.3988567 | 0.7574908 | 0.6151673 |
| 3 | C | C3 | 0.5377290 | -0.7716186 | 0.4834085 |
| 4 | H | H6 | 0.6119170 | -1.2877553 | 1.4356786 |
| 5 | C | C10 | -0.1664634 | -1.5377762 | -0.5636591 |
| 6 | H | H11 | -0.2166977 | -1.0847065 | -1.5512695 |

| | | | | | |
|---|---|---|---|---|---|
| 7 | C | C11 | -0.7243985 | -2.7353400 | -0.3801304 |
| 8 | H | H12 | -0.7005566 | -3.2348517 | 0.5837612 |
| 9 | H | H2 | 0.3943943 | 1.1086332 | 1.6424865 |
| 10 | H | H8 | 2.5494545 | 0.1604584 | 0.8004636 |
| 11 | H | H9 | 1.9359198 | 0.2523124 | -0.9131092 |
| 12 | H | H15 | -1.2222093 | -3.2567650 | -1.1892550 |
| 13 | C | C4 | -0.4449305 | 1.5535815 | -0.2977803 |
| 14 | C | C6 | -1.1908461 | 2.5945287 | 0.0754445 |
| 15 | H | H1 | -1.2316971 | 2.9258508 | 1.1086497 |
| 16 | H | H13 | -1.7851697 | 3.1497837 | -0.6406867 |
| 17 | H | H17 | -0.4376017 | 1.2668891 | -1.3470473 |

## Divinyl Species [3,3]-TS (S-TS8)

Spartan'10: B3LYP/6-31G*, G = -272.536339, iv = i434
Gaussian'09: B3LYP/6-31G*, G = -272.53598, iv = i419
M06-2X/6-31G*,G = -272.394561, iv = i518

Cartesian Coordinates (Angstroms)

| Atom | | | X | Y | Z |
|---|---|---|---|---|---|
| 1 | C | C1 | -1.3141431 | 0.0858792 | 1.1956138 |
| 2 | H | H3 | -1.5218186 | -0.9575706 | 0.9960566 |
| 3 | C | C2 | -0.0291084 | 0.4926157 | 1.5187609 |
| 4 | C | C5 | -0.0356607 | 0.6217441 | -1.4704223 |
| 5 | H | H7 | 0.0907939 | 1.6082371 | -1.9169345 |
| 6 | C | C6 | -1.3218653 | 0.1955604 | -1.1769583 |
| 7 | H | H6 | -1.5372630 | -0.8597391 | -1.0695242 |
| 8 | C | C3 | 1.1243576 | 0.0005740 | -0.9981933 |
| 9 | C | C4 | 1.1294915 | -0.0825547 | 0.9885817 |
| 10 | C | C11 | 1.1450748 | -1.1596204 | -0.0516523 |
| 11 | H | H2 | 2.0898886 | 0.4141807 | -1.2762521 |
| 12 | H | H10 | 2.0959052 | 0.3084491 | 1.2937960 |
| 13 | H | H17 | -2.1701876 | 0.8034414 | -1.4793252 |
| 14 | H | H21 | 0.2677384 | -1.8068587 | -0.0775966 |
| 15 | H | H22 | 2.0565693 | -1.7585354 | -0.0796222 |
| 16 | H | H1 | -2.1655803 | 0.6564818 | 1.5562745 |
| 17 | H | H11 | 0.0958078 | 1.4377156 | 2.0473972 |

## Furan basic alkene INT [3,3] (S23)

Spartan'10: B3LYP/6-31G*, G = -423.988081, iv = none
Gaussian'09: B3LYP/6-31G*, G = -423.990183, iv = none
M06-2X/6-31G*,G = -423.804524, iv = none

Cartesian Coordinates (Angstroms)

| Atom | | | X | Y | Z |
|---|---|---|---|---|---|
| 1 | C | C1 | 2.0211098 | -0.2019559 | -0.2878635 |
| 2 | C | C2 | 1.0455931 | 0.5679385 | 0.5563511 |
| 3 | C | C3 | 1.1811074 | -0.9579600 | 0.7227411 |
| 4 | H | H6 | 1.6950687 | -1.2655714 | 1.6311662 |
| 5 | C | C10 | 0.1137728 | -1.8791687 | 0.2796023 |
| 6 | H | H11 | -0.4504758 | -1.5718682 | -0.6005445 |

| | | | | | |
|---|---|---|---|---|---|
| 7 | C | C11 | -0.1862803 | -3.0316599 | 0.8841588 |
| 8 | H | H12 | 0.3516805 | -3.3717411 | 1.7665564 |
| 9 | C | C4 | -0.1463955 | 1.1835360 | -0.0514472 |
| 10 | C | C5 | -0.5099529 | 1.5057073 | -1.3288209 |
| 11 | C | C6 | -1.8161123 | 2.0934018 | -1.2526201 |
| 12 | C | C7 | -2.1512630 | 2.0926024 | 0.0654157 |
| 13 | H | H1 | 0.0865861 | 1.3586969 | -2.2183691 |
| 14 | H | H8 | -2.4149282 | 2.4695347 | -2.0705875 |
| 15 | H | H9 | -3.0151894 | 2.4311237 | 0.6170684 |
| 16 | O | O3 | -1.1518294 | 1.5413520 | 0.8119593 |
| 17 | H | H2 | 1.4658002 | 1.1388947 | 1.3828715 |
| 18 | H | H3 | 3.0821058 | -0.0798072 | -0.0895872 |
| 19 | H | H4 | 1.7797895 | -0.3460992 | -1.3375905 |
| 20 | H | H7 | -0.9801871 | -3.6769564 | 0.5195397 |

## Furan basic alkene TS [3,3] (S-TS10)

Spartan'10: B3LYP/6-31G*, G = -423.948929, iv = i375
Gaussian'09: B3LYP/6-31G*, G = -423.94862, iv = i364
M06-2X/6-31G*,G = -423.804524, iv = i501

Cartesian Coordinates (Angstroms)

| | | Atom | X | Y | Z |
|---|---|---|---|---|---|
| 1 | C | C1 | -1.0283397 | -0.4010231 | 0.7571931 |
| 2 | H | H3 | -1.3288846 | -1.4018108 | 0.4822254 |
| 3 | C | C2 | 0.3149653 | -0.0843233 | 1.1143534 |
| 4 | C | C5 | 0.1806775 | 0.5851929 | -1.8049434 |
| 5 | H | H7 | 0.1886549 | 1.6298365 | -2.1155432 |
| 6 | C | C6 | -1.0478457 | -0.0056010 | -1.4914104 |
| 7 | H | H6 | -1.1562864 | -1.0802117 | -1.5818701 |
| 8 | C | C3 | 1.4061193 | -0.0015796 | -1.5255067 |
| 9 | C | C4 | 1.5056262 | -0.4561222 | 0.5381058 |
| 10 | C | C11 | 1.5647472 | -1.2614028 | -0.7289562 |
| 11 | H | H2 | 2.3179841 | 0.5447544 | -1.7517164 |
| 12 | H | H10 | 2.4243494 | -0.0184088 | 0.9139063 |
| 13 | O | O3 | 0.3046054 | 0.9591443 | 2.0340899 |
| 14 | C | C8 | -1.0061254 | 1.2405430 | 2.2973044 |
| 15 | H | H12 | -1.1694267 | 2.0232775 | 3.0244229 |
| 16 | C | C7 | -1.8501102 | 0.4577518 | 1.5881914 |
| 17 | H | H11 | -2.9302458 | 0.4734522 | 1.6347546 |
| 18 | H | H8 | -1.9647426 | 0.5515984 | -1.6637485 |
| 19 | H | H13 | 0.7543954 | -1.9858727 | -0.8430314 |
| 20 | H | H14 | 2.5198825 | -1.7691948 | -0.8778209 |

## Thiophene basic alkene INT [3,3] (S24)

Spartan'10: B3LYP/6-31G*, G = -746.970146, iv = none
Gaussian'09: B3LYP/6-31G*, G = -746.972369, iv = none
M06-2X/6-31G*,G = -746.779248, iv = none

Cartesian Coordinates (Angstroms)

| | | Atom | X | Y | Z |
|---|---|---|---|---|---|
| 1 | C | C1 | 2.0342002 | -0.2274222 | -0.0893840 |
| 2 | C | C2 | 1.0083838 | 0.5175287 | 0.7205973 |
| 3 | C | C3 | 1.1438292 | -1.0097873 | 0.8564552 |
| 4 | H | H6 | 1.6098827 | -1.3407959 | 1.7825414 |
| 5 | C | C10 | 0.1071938 | -1.9243166 | 0.3331753 |
| 6 | H | H11 | -0.4024356 | -1.6036092 | -0.5748702 |
| 7 | C | C11 | -0.2265279 | -3.0867859 | 0.9001834 |
| 8 | H | H12 | 0.2573181 | -3.4399102 | 1.8085163 |
| 9 | C | C4 | -0.1614673 | 1.1537212 | 0.0676524 |
| 10 | C | C5 | -0.3028157 | 1.5991983 | -1.2219863 |
| 11 | C | C6 | -1.5793267 | 2.1816071 | -1.4925050 |
| 12 | C | C7 | -2.4031674 | 2.1789389 | -0.4026654 |
| 13 | H | H1 | 0.4935970 | 1.5255504 | -1.9551030 |
| 14 | H | H8 | -1.8643257 | 2.5889943 | -2.4567860 |
| 15 | H | H9 | -3.4137035 | 2.5570230 | -0.3227241 |
| 16 | S | S1 | -1.6333832 | 1.4513366 | 0.9735079 |
| 17 | H | H2 | 1.3955547 | 1.0633116 | 1.5801496 |
| 18 | H | H3 | 3.0826091 | -0.1075179 | 0.1690450 |
| 19 | H | H4 | 1.8493969 | -0.3496373 | -1.1530430 |
| 20 | H | H7 | -0.9948125 | -3.7274275 | 0.4772432 |

## Thiophene basic alkene TS [3,3] (S-TS11)

Spartan'10: B3LYP/6-31G*, G = -746.930649, iv = i388
Gaussian'09: B3LYP/6-31G*, G = -746.93057, iv = i390
M06-2X/6-31G*,G = -746.731749, iv = i513

Cartesian Coordinates (Angstroms)

| | | Atom | X | Y | Z |
|---|---|---|---|---|---|
| 1 | C | C1 | -0.9189548 | -0.4240114 | 0.7504790 |
| 2 | H | H3 | -1.1147122 | -1.4402266 | 0.4318292 |
| 3 | C | C2 | 0.4086231 | -0.0557975 | 1.1303085 |
| 4 | C | C5 | 0.2563491 | 0.6375349 | -1.7780825 |
| 5 | H | H7 | 0.2835947 | 1.6895231 | -2.0613975 |
| 6 | C | C6 | -0.9818885 | 0.0654255 | -1.4649227 |
| 7 | H | H6 | -1.1223033 | -1.0004326 | -1.6017383 |
| 8 | C | C3 | 1.4714917 | 0.0183867 | -1.5254622 |
| 9 | C | C4 | 1.5815088 | -0.4486707 | 0.5176155 |
| 10 | C | C11 | 1.6126170 | -1.2567297 | -0.7494393 |
| 11 | H | H2 | 2.3922651 | 0.5471979 | -1.7569661 |
| 12 | H | H10 | 2.5296780 | -0.0491525 | 0.8658829 |
| 13 | S | S1 | 0.3554048 | 1.2920013 | 2.2966335 |
| 14 | C | C8 | -1.3938788 | 1.2278275 | 2.3506109 |
| 15 | H | H12 | -1.9282369 | 1.8907785 | 3.0194038 |
| 16 | C | C7 | -1.9142747 | 0.2946522 | 1.5169151 |
| 17 | H | H11 | -2.9785961 | 0.0998604 | 1.4329914 |
| 18 | H | H8 | -1.8852902 | 0.6542682 | -1.5999269 |
| 19 | H | H13 | 0.7880972 | -1.9646036 | -0.8631282 |
| 20 | H | H14 | 2.5585060 | -1.7778317 | -0.9116061 |

## Non-fluoro Pip cis (S25)

Spartan'10: B3LYP/6-31G*, G = -842.539706, iv = none
Gaussian'09: B3LYP/6-31G*, G = -842.546027, iv = none
M06-2X/6-31G*,G = -842.201221, iv = none

Cartesian Coordinates (Angstroms)

| | Atom | | X | Y | Z |
|---|---|---|---|---|---|
| 1 | C | C1 | 3.0422011 | -0.2059109 | 0.8333341 |
| 2 | C | C2 | 1.9462495 | 0.6301462 | 1.4123946 |
| 3 | C | C3 | 1.7915637 | -0.9034859 | 1.3561728 |
| 4 | H | H6 | 1.8915419 | -1.4139110 | 2.3115219 |
| 5 | C | C10 | 0.8668632 | -1.5315163 | 0.4062663 |
| 6 | H | H11 | 0.7192598 | -1.0383017 | -0.5535636 |
| 7 | C | C11 | 0.1896422 | -2.6696412 | 0.6288198 |
| 8 | H | H12 | 0.2760755 | -3.2240637 | 1.5592409 |
| 9 | C | C12 | -0.7076642 | -3.2212370 | -0.4038901 |
| 10 | O | O1 | -0.9249719 | -2.7396061 | -1.5003832 |
| 11 | O | O2 | -1.2831356 | -4.3747921 | 0.0257938 |
| 12 | C | C13 | -2.1743954 | -4.9936572 | -0.9108810 |
| 13 | H | H5 | -3.0200563 | -4.3360514 | -1.1326643 |
| 14 | H | H13 | -2.5192305 | -5.9092198 | -0.4284011 |
| 15 | H | H14 | -1.6551503 | -5.2236293 | -1.8455651 |
| 16 | C | C4 | 1.0978825 | 1.5520209 | 0.5894276 |
| 17 | C | C5 | 0.7952224 | 3.0268768 | -1.3505680 |
| 18 | C | C6 | -0.9677564 | 2.7108075 | 0.2779478 |
| 19 | C | C7 | -0.4784712 | 3.2893029 | -0.8931402 |
| 20 | C | C9 | 1.5781117 | 2.1438437 | -0.5822917 |
| 21 | H | H1 | 1.1760331 | 3.4839111 | -2.2574735 |
| 22 | H | H4 | 2.5895542 | 1.9257801 | -0.9106321 |
| 23 | C | C8 | -0.2120485 | 1.8458768 | 1.0381704 |
| 24 | H | H2 | 2.1242673 | 0.9867590 | 2.4267254 |
| 25 | H | H3 | -0.6104691 | 1.3989510 | 1.9432289 |
| 26 | O | O3 | -2.2450646 | 3.1618447 | 0.5151643 |
| 27 | O | O4 | -1.4305404 | 4.1247672 | -1.4279509 |
| 28 | C | C14 | -2.6158043 | 3.8916840 | -0.6597057 |
| 29 | H | H7 | -3.0544472 | 4.8499658 | -0.3687754 |
| 30 | H | H10 | -3.3255377 | 3.2962697 | -1.2517681 |
| 31 | H | H8 | 3.9619561 | -0.3310041 | 1.3976659 |
| 32 | H | H9 | 3.1783192 | -0.2027794 | -0.2442203 |

## Non-fluoro Pip cis [3,3] TS (S-TS12)

Spartan'10: B3LYP/6-31G*, G = -842.491477, iv = i374
Gaussian'09: B3LYP/6-31G*, G = -842.495343, iv = i369
M06-2X/6-31G*,G = -842.148309, iv = i487

Cartesian Coordinates (Angstroms)

| | Atom | | X | Y | Z |
|---|---|---|---|---|---|
| 1 | C | C1 | 0.0520814 | -0.9917968 | 0.5259498 |
| 2 | H | H3 | -0.0680629 | -2.0610640 | 0.3911571 |
| 3 | C | C2 | 1.3793483 | -0.5890499 | 1.0005366 |
| 4 | C | C5 | 1.3946502 | -0.4550237 | -2.0184814 |
| 5 | H | H7 | 1.4907740 | 0.4909212 | -2.5477833 |
| 6 | C | C6 | 0.0803486 | -0.8195318 | -1.5814762 |

| | | | | | |
|---|---|---|---|---|---|
| 7 | H | H6 | -0.2016633 | -1.8639056 | -1.6606524 |
| 8 | C | C7 | 1.5052318 | 0.5844828 | 1.8403494 |
| 9 | H | H4 | 2.4855943 | 0.9358776 | 2.1449348 |
| 10 | C | C8 | 0.3724354 | 1.2214879 | 2.2165992 |
| 11 | C | C9 | -0.9278742 | 0.7683988 | 1.8341607 |
| 12 | C | C10 | -1.1222078 | -0.3267477 | 1.0639775 |
| 13 | H | H12 | -2.1142224 | -0.6851961 | 0.8171351 |
| 14 | C | C3 | 2.5223390 | -1.1324057 | -1.6537495 |
| 15 | C | C4 | 2.5117020 | -1.2325879 | 0.5429250 |
| 16 | C | C11 | 2.5316142 | -2.2105191 | -0.5981357 |
| 17 | H | H2 | 3.4917592 | -0.7607891 | -1.9772189 |
| 18 | H | H10 | 3.4793786 | -0.8679440 | 0.8810774 |
| 19 | C | C12 | -1.0121538 | 0.1108352 | -1.9457873 |
| 20 | O | O1 | -0.8861163 | 1.2902719 | -2.2159820 |
| 21 | O | O2 | -2.2234775 | -0.5100299 | -1.9038794 |
| 22 | C | C13 | -3.3475585 | 0.3342121 | -2.1883485 |
| 23 | H | H1 | -3.2619765 | 0.7689444 | -3.1882166 |
| 24 | H | H5 | -3.4161761 | 1.1444830 | -1.4567240 |
| 25 | H | H8 | -4.2243907 | -0.3122520 | -2.1268798 |
| 26 | O | O3 | -1.8664221 | 1.5764937 | 2.4190921 |
| 27 | O | O4 | 0.2391522 | 2.3310099 | 3.0111647 |
| 28 | C | C14 | -1.1535291 | 2.6447480 | 3.0458727 |
| 29 | H | H9 | -1.3334033 | 3.5774634 | 2.4962941 |
| 30 | H | H13 | -1.4795959 | 2.7345598 | 4.0862811 |
| 31 | H | H11 | 3.4385629 | -2.8191869 | -0.6170676 |
| 32 | H | H14 | 1.6638581 | -2.8761594 | -0.6371249 |
